# The seventh blind test of crystal structure prediction: structure generation methods

**DOI:** 10.1107/S2052520624007492

**Published:** 2024-12-01

**Authors:** Lily M. Hunnisett, Jonas Nyman, Nicholas Francia, Nathan S. Abraham, Claire S. Adjiman, Srinivasulu Aitipamula, Tamador Alkhidir, Mubarak Almehairbi, Andrea Anelli, Dylan M. Anstine, John E. Anthony, Joseph E. Arnold, Faezeh Bahrami, Michael A. Bellucci, Rajni M. Bhardwaj, Imanuel Bier, Joanna A. Bis, A. Daniel Boese, David H. Bowskill, James Bramley, Jan Gerit Brandenburg, Doris E. Braun, Patrick W. V. Butler, Joseph Cadden, Stephen Carino, Eric J. Chan, Chao Chang, Bingqing Cheng, Sarah M. Clarke, Simon J. Coles, Richard I. Cooper, Ricky Couch, Ramon Cuadrado, Tom Darden, Graeme M. Day, Hanno Dietrich, Yiming Ding, Antonio DiPasquale, Bhausaheb Dhokale, Bouke P. van Eijck, Mark R. J. Elsegood, Dzmitry Firaha, Wenbo Fu, Kaori Fukuzawa, Joseph Glover, Hitoshi Goto, Chandler Greenwell, Rui Guo, Jürgen Harter, Julian Helfferich, Detlef W. M. Hofmann, Johannes Hoja, John Hone, Richard Hong, Geoffrey Hutchison, Yasuhiro Ikabata, Olexandr Isayev, Ommair Ishaque, Varsha Jain, Yingdi Jin, Aling Jing, Erin R. Johnson, Ian Jones, K. V. Jovan Jose, Elena A. Kabova, Adam Keates, Paul F. Kelly, Dmitry Khakimov, Stefanos Konstantinopoulos, Liudmila N. Kuleshova, He Li, Xiaolu Lin, Alexander List, Congcong Liu, Yifei Michelle Liu, Zenghui Liu, Zhi-Pan Liu, Joseph W. Lubach, Noa Marom, Alexander A. Maryewski, Hiroyuki Matsui, Alessandra Mattei, R. Alex Mayo, John W. Melkumov, Sharmarke Mohamed, Zahrasadat Momenzadeh Abardeh, Hari S. Muddana, Naofumi Nakayama, Kamal Singh Nayal, Marcus A. Neumann, Rahul Nikhar, Shigeaki Obata, Dana O’Connor, Artem R. Oganov, Koji Okuwaki, Alberto Otero-de-la-Roza, Constantinos C. Pantelides, Sean Parkin, Chris J. Pickard, Luca Pilia, Tatyana Pivina, Rafał Podeszwa, Alastair J. A. Price, Louise S. Price, Sarah L. Price, Michael R. Probert, Angeles Pulido, Gunjan Rajendra Ramteke, Atta Ur Rehman, Susan M. Reutzel-Edens, Jutta Rogal, Marta J. Ross, Adrian F. Rumson, Ghazala Sadiq, Zeinab M. Saeed, Alireza Salimi, Matteo Salvalaglio, Leticia Sanders de Almada, Kiran Sasikumar, Sivakumar Sekharan, Cheng Shang, Kenneth Shankland, Kotaro Shinohara, Baimei Shi, Xuekun Shi, A. Geoffrey Skillman, Hongxing Song, Nina Strasser, Jacco van de Streek, Isaac J. Sugden, Guangxu Sun, Krzysztof Szalewicz, Benjamin I. Tan, Lu Tan, Frank Tarczynski, Christopher R. Taylor, Alexandre Tkatchenko, Rithwik Tom, Mark E. Tuckerman, Yohei Utsumi, Leslie Vogt-Maranto, Jake Weatherston, Luke J. Wilkinson, Robert D. Willacy, Lukasz Wojtas, Grahame R. Woollam, Zhuocen Yang, Etsuo Yonemochi, Xin Yue, Qun Zeng, Yizu Zhang, Tian Zhou, Yunfei Zhou, Roman Zubatyuk, Jason C. Cole

**Affiliations:** a The Cambridge Crystallographic Data Centre 12 Union Road Cambridge CB2 1EZ UK; b Department of Chemical Engineering, University College London, Torrington Place, London WC1E 7JE, UK; c AbbVie Inc., Research & Development, 1 N Waukegan Road, North Chicago, IL 60064, USA; d Department of Chemical Engineering, Sargent Centre for Process Systems Engineering and Institute for Molecular Science and Engineering, Imperial College London, London SW7 2AZ, UK; e Institute of Sustainability for Chemicals Energy and Environment (ISCE2) Agency for Science, Technology and Research (A*STAR) 1 Pesek Road Jurong Island Singapore 627833 Republic of Singapore; f Green Chemistry and Materials Modelling Laboratory Khalifa University of Science and Technology PO Box 127788 Abu Dhabi United Arab Emirates; g Roche Pharma Research and Early Development Therapeutic Modalities Roche Innovation Center Basel F Hoffmann-La Roche Ltd Grenzacherstrasse 124 4070 Basel Switzerland; h Department of Chemistry, Carnegie Mellon University, 4400 Fifth Avenue, Pittsburgh, PA 15213, USA; i Department of Chemistry University of Kentucky Lexington KY 40506 USA; j School of Chemistry, University of Southampton, Southampton SO17 1BJ, UK; k Department of Chemistry, Faculty of Science, Ferdowsi University of Mashhad, Mashhad, Iran; l XtalPi Inc., 245 Main Street, Cambridge, MA 02142, USA; m Department of Materials Science and Engineering, Carnegie Mellon University, 5000 Forbes Avenue, Pittsburgh, PA 15213, USA; n Catalent Pharma Solutions 160 Pharma Drive Morrisville NC 27560 USA; o University of Graz, Department of Chemistry, Heinrichstrasse 28, Graz, Austria; p Group Science and Technology Office, Merck KGaA, Frankfurter Str. 250, 64293 Darmstadt, Germany; q University of Innsbruck, Institute of Pharmacy, Innrain 52c, A-6020 Innsbruck, Austria; r Department of Chemistry New York University New York NY 10003 USA; s Curtin Institute for Computation, School of Molecular and Life Sciences, Curtin University, Perth, Western Australia 6845, Australia; t XtalPi Inc., International Biomedical Innovation Park II 3F 2 Hongliu Road, Futian District, Shenzhen, Guangdong, China; u Institute of Science and Technology Austria, Klosterneuburg 3400, Austria; v Department of Chemistry, Dalhousie University, 6274 Coburg Road, Dalhousie, Halifax, Canada; w Department of Chemistry, University of Oxford, 12 Mansfield Road, Oxford OX1 3TA, UK; x OpenEye Scientific Software, 9 Bisbee Court, Santa Fe, NM 87508, USA; y Avant-garde Materials Simulation, Alte Strasse 2, 79249 Merzhausen, Germany; z Department of Chemistry, University College London, 20 Gordon Street, London WC1H 0AJ, UK; aa Genentech, Inc., 1 DNA Way, South San Francisco, CA 94080, USA; bb Department of Chemistry University of Wyoming Laramie Wyoming 82071 USA; cc University of Utrecht (Retired), Department of Crystal and Structural Chemistry, Padualaan 8, 3584 CH Utrecht, The Netherlands; dd Chemistry Department, Loughborough University, Loughborough LE11 3TU, UK; ee Graduate School of Pharmaceutical Sciences, Osaka University, 1-6 Yamadaoka, Suita, Osaka 656-0871, Japan; ff School of Pharmacy and Pharmaceutical Sciences, Hoshi University, 2-4-41 Ebara, Shinagawa-ku, Tokyo 142-8501, Japan; gg Information and Media Center, Toyohashi University of Technology, 1-1 Hibarigaoka, Tempaku-cho, Toyohashi, Aichi 441-8580, Japan; hh CONFLEX Corporation, Shinagawa Center building 6F, 3-23-17 Takanawa, Minato-ku, Tokyo 108-0074, Japan; ii CRS4, Loc. Piscina Mana 1, 09050 Pula, Italy; jj Syngenta Ltd, Jealott’s Hill International Research Station, Berkshire, RG42 6EY, UK; kk Department of Chemistry, University of Pittsburgh, 219 Parkman Avenue, Pittsburgh, PA 15260, USA; ll Department of Physics and Astronomy, University of Delaware, Newark, DE 19716, USA; mm School of Chemistry, University of Hyderabad, Professor C.R. Rao Road, Gachibowli, Hyderabad, 500046 Telangana, India; nn School of Pharmacy, University of Reading, Whiteknights, Reading, RG6 6AD, UK; oo N. D. Zelinsky Institute of Organic Chemistry, Russian Academy of Sciences, Leninskiy Prospekt 47, Moscow 119991, Russia; pp FlexCryst, Schleifweg 23, 91080 Uttenreuth, Germany; qq Shanghai Key Laboratory of Molecular Catalysis and Innovative Materials, Key Laboratory of Computational Physical Science, Department of Chemistry, Fudan University,Shanghai 200433, China; rr Skolkovo Institute of Science and Technology, Bolshoy Boulevard 30, 121205 Moscow, Russia; ss Graduate School of Organic Materials Science, Yamagata University, 4-3-16 Jonan, Yonezawa 992-8510, Yamagata, Japan; tt Center for Catalysis and Separations, Khalifa University of Science and Technology, PO Box 127788, Abu Dhabi, United Arab Emirates; uu Department of Analytical and Physical Chemistry, Faculty of Chemistry, University of Oviedo, Julián Clavería 8, 33006 Oviedo, Spain; vv Department of Materials Science and Metallurgy, University of Cambridge, 27 Charles Babbage Road, Cambridge CB3 0FS, UK; ww Advanced Institute for Materials Research, Tohoku University 2-1-1 Katahira, Aoba, Sendai, 980-8577, Japan; xx Department of Mechanical, Chemical and Materials Engineering, University of Cagliari, Via Marengo 2, 09123 Cagliari, Italy; yy Institute of Chemistry, University of Silesia in Katowice, Szkolna 9, 40-006 Katowice, Poland; zz School of Natural and Environmental Sciences, Newcastle University, Kings Road, Newcastle NE1 7RU, UK; aaa SuRE Pharma Consulting, LLC, 7163 Whitestown Parkway - Suite 305, Zionsville, IN 46077, USA; bbb Faculty of Physics, Freie Universität Berlin, Arnimallee 14, 14195 Berlin, Germany; ccc Department of Physics and Materials Science, University of Luxembourg, 1511 Luxembourg City, Luxembourg; ddd Courant Institute of Mathematical Sciences, New York University, New York, NY 10012, USA; eee NYU-ECNU Center for Computational Chemistry at NYU Shanghai, 3663 Zhongshan Road North, Shanghai 200062, China; fff Department of Chemistry, University of South Florida, USF Research Park, 3720 Spectrum Blvd, IDRB 202, Tampa, FL 33612 USA; ggg Novartis Pharma AG, Basel 4002, Switzerland; CSIR–National Chemical Laboratory, India

**Keywords:** crystal structure prediction, polymorphism, lattice energy, Cambridge Structural Database, blind test

## Abstract

A seventh blind test of crystal structure prediction was organized by the Cambridge Crystallographic Data Centre featuring seven target systems of varying complexity: a silicon and iodine-containing molecule, a copper coordination complex, a near-rigid molecule, a cocrystal, a polymorphic small agrochemical, a highly flexible polymorphic drug candidate, and a polymorphic morpholine salt. In this first of two parts focusing on structure generation methods, many crystal structure prediction (CSP) methods performed well for the small but flexible agrochemical compound, successfully reproducing the experimentally observed crystal structures, while few groups were successful for the systems of higher complexity. A powder X-ray diffraction (PXRD) assisted exercise demonstrated the use of CSP in successfully determining a crystal structure from a low-quality PXRD pattern. The use of CSP in the prediction of likely cocrystal stoichiometry was also explored, demonstrating multiple possible approaches. Crystallographic disorder emerged as an important theme throughout the test as both a challenge for analysis and a major achievement where two groups blindly predicted the existence of disorder for the first time. Additionally, large-scale comparisons of the sets of predicted crystal structures also showed that some methods yield sets that largely contain the same crystal structures.

## Abstract 2

The results of the seventh blind test of crystal structure prediction are presented, focusing on structure generation methods.

## 1. Introduction

### 1.1. Background

Crystal structure prediction (CSP) seeks to predict the most likely crystal structures of a given compound from the chemical composition alone. This is of paramount importance for the design and discovery of new molecular materials, as well as for understanding the physicochemical properties of existing compounds. Since the early 1990s, numerous computational methods have been developed to tackle this complex problem, with varying degrees of success.

The combined use of computational modelling and experimental techniques is ideally suited for elucidating the structures and properties of crystals that cannot be isolated at ambient conditions, such as clathrates and exotic crystal structures that may form in the laboratory, or on other planets (Selent *et al.*, 2017 ▸; Maynard-Casely *et al.*, 2016 ▸; Zhang *et al.*, 2013 ▸).

Although other approaches are conceivable (Kitaigorodsky, 2012 ▸; Day & Motherwell, 2006 ▸), CSP generally consists of a computational search for all possible crystal packings and an estimation of the crystals’ (relative) thermodynamic stability (Day, 2011 ▸), often calculated as the cohesive energy of the perfect static structure, somewhat improperly called the ‘lattice energy’ (Palgrave & Tobin, 2021 ▸). Thermal contributions to the stability, of which the lattice vibrational entropy is the largest, are sometimes also considered (Dolgonos *et al.*, 2019 ▸; O’Connor *et al.*, 2022 ▸). The lowest energy structure is expected to be the thermodynamically stable form, and other structures within a few kJ mol^−1^ may be possible metastable polymorphs (Gavezzotti & Filippini, 1995 ▸; Day, 2011 ▸; Nyman & Day, 2015 ▸). The kinetics of nucleation and growth are currently not considered in a standard CSP calculation.

Every CSP method necessarily involves some algorithm for packing the molecule(s) under study into periodic three-dimensional crystal structures, that is, lattices are introduced and the molecule(s) are then placed in the unit cell. The resulting crystal structures should be ‘good enough’ that they fall within the basins of attraction of a more accurate energy method, thereby enabling subsequent geometry optimization of the structure by minimizing its energy. The generation of crystal structures ideally explores the entire search space, so that all relevant energy minima are found.

A series of blind tests evaluating and benchmarking methods of crystal structure prediction have been organized by the Cambridge Crystallographic Data Centre (CCDC). Since the inception of the first CSP blind test in 1999 (Lommerse *et al.*, 2000 ▸), six such tests have been conducted, providing valuable insights into the strengths and limitations of existing methodologies and promoting the development of more accurate and efficient algorithms.

Here we present the results of the seventh CSP blind test, organized by the CCDC. This blind test featured an unprecedented level of complexity in terms of the number, size and diversity of chemical compositions among seven target compounds, an endeavour which prompted the test itself to be conducted in two phases: structure generation and energy ranking, over the course of a year and a half. In this contribution, the results of the structure generation phase are presented, highlighting the successes and challenges in comprehensively producing putative crystal structures of ever more relevant model compounds, and matching the computed crystal packings to experimentally observed polymorphs. We assess the current state-of-the-art in crystal structure generation and structure matching methods and discuss the implications of these findings for the future development of CSP techniques.

This study includes four distinct supplementary information (SI) sections. SI-A offers more information, tables, and figures on the analysis of the generated sets of structures. In SI-B, participating groups define their approach and some provide additional analysis of their landscape and results. SI-C provides details on the experimental determination of the crystal structures considered in this test. Finally, SI-D contains the theoretically generated structures (and metadata) from each group, and any experimental structures that are not yet available through the Cambridge Structural Database (CSD) in the Crystallographic Information File (CIF; a standardized file format for crystallographic data) format.

Computational methods are often referred to by acronyms. We have therefore provided a glossary of abbreviations at the end of this paper to aid the reader.

### 1.2. Commonly used computational methods for crystal structure generation

Crystal structure generation is a crucial step in CSP, as it provides a set of candidate structures to be subsequently refined and ranked based on their relative stabilities. Several methods have been developed for generating crystal structures, each with its advantages and limitations. Here, we briefly describe some of the methods used for sampling the search space, including grid-based, pseudo-random, quasi-random, simulated annealing, parallel tempering, and genetic algorithms.

Grid-based methods may sample lattice parameters that are not constrained by symmetry as multiples of some small units of distance and angle, followed by dividing the unit cell into a regular grid of points and placing the molecule at each position, and sampling orientations by a grid of Euler angles (van Eijck *et al.*, 1998 ▸) or uniformly distributed rotation matrices (Arvo, 1992 ▸). These methods are easy to implement and can efficiently sample packing space for small rigid molecules. However, they may not be sufficient for sampling the conformational space of flexible molecules. Grid-based methods were common in the first blind tests, but have now largely been replaced by other methods.

There are also synthon-based methods involving a rational or systematic build-up of molecular dimers, chains or coordination spheres. These methods identify likely synthons, either from energy calculations or statistical estimates derived from the Cambridge Structural Database (CSD) (Groom *et al.*, 2016 ▸), and then successively construct crystal structures following a procedure inspired by an Aufbau principle (Hofmann & Lengauer, 1997 ▸; Hofmann *et al.*, 2004 ▸; Ganguly & Desiraju, 2010 ▸). One possible advantage of such methods is that they may incorporate kinetic effects, biasing the CSP towards structures that more easily nucleate and grow (Sarma & Desiraju, 2002 ▸).

Random methods use a deterministic algorithm to generate a sequence of numbers that appear statistically random. In the context of CSP, these are then employed to generate random molecular conformations, positions and orientations within a unit cell with randomly assigned lattice parameters. The commonly used pseudo-random numbers are known to not sample the multidimensional search space evenly (Hayes, 2011 ▸). Quasi-random numbers, also known as low-discrepancy sequences, generate well distributed points in the search space, leading to a more efficient sampling of crystal structures (Sobol’, 1967 ▸; Lin *et al.*, 2016 ▸; Case *et al.*, 2016 ▸).

Simulated annealing is a stochastic optimization technique inspired by the annealing process in metallurgy. In CSP, this method involves generating an initial crystal structure and then perturbing it through a series of random moves, such as changes to the molecular conformation, translations, rotations, or changes in the unit cell parameters. The new structures are accepted or rejected based on a Metropolis criterion (Metropolis *et al.*, 1953 ▸). The temperature is gradually decreased during the process, which leads to the generation of low-energy structures. Simulated annealing allows a thorough exploration of the search space and can escape shallow energy minima, leading to the identification of the most stable structures (Gdanitz, 1992 ▸; Catlow *et al.*, 1993 ▸). Simulated annealing can be improved by performing several simulations in parallel at different temperatures and swapping configurations between them, a method referred to as parallel tempering. Parallel tempering makes high-temperature configurations available to low-temperature simulations, greatly enhancing the sampling of configurational space, and it is therefore more computationally efficient overall (Earl & Deem, 2005 ▸).

Genetic algorithms are inspired by the principles of natural selection and genetic recombination. Here, the crystal structures and the degrees of freedom to be explored are represented by computational ‘genes’. The algorithm starts by randomly generating an initial population of crystal structures, which are then subjected to a series of genetic operations such as crossover, mutation, and selection. Crossover involves the recombination of two parent structures to produce offspring, while mutation introduces random perturbations in the structures. The selection process favours structures with lower energies, mimicking the survival of the fittest principle. Genetic algorithms can efficiently explore the crystal structure space and generate diverse structures, including those that correspond to experimentally observed forms (Oganov & Glass, 2006 ▸; Glass *et al.*, 2006 ▸; Abraham & Probert, 2006 ▸; Bahmann & Kortus, 2013 ▸; Curtis *et al.*, 2018 ▸).

Regardless of what sampling method is used, there is a need to efficiently score the generated structures by some metric. The lattice energy is the most common ranking metric. Dispersion-corrected density functional theory (DFT-D) is considered by the CSP community as a reliable method for evaluating the lattice energy (Hoja *et al.*, 2019 ▸; Maurer *et al.*, 2019 ▸; O’Connor *et al.*, 2022 ▸; Price *et al.*, 2023 ▸). However, owing to the high computational cost of density functional theory (DFT) there is a need for more efficient ranking methods for evaluating a large number (millions) of putative structures. These include tailor-made force fields fitted to the specific compound (Neumann, 2008 ▸; Yang *et al.*, 2020 ▸; Nikhar & Szalewicz, 2022 ▸), machine-learned potentials (Musil *et al.*, 2018 ▸; Zubatyuk *et al.*, 2019 ▸; Clements *et al.*, 2022 ▸; Unke *et al.*, 2021 ▸; Egorova *et al.*, 2020 ▸), potentials trained on the CSD (Hofmann & Kuleshova, 2023 ▸), anisotropic force fields (Stone & Price, 1988 ▸; Price *et al.*, 2010 ▸) or statistical potentials that estimate how similar the local atomic environments (Bartók *et al.*, 2013 ▸) are to experimentally observed structures in the CSD (Hofmann & Apostolakis, 2003 ▸; Cole *et al.*, 2016 ▸).

### 1.3. History of the CSP blind tests

The first blind test of crystal structure prediction (1998–1999) featured three target molecules, two small and rigid, and one slightly larger molecule (28 atoms) with two rotatable bonds. The structure generation methods included fairly simple pseudo-random sampling of molecular and unit cell degrees of freedom, simulated annealing in *Polymorph Predictor* (Leusen, 1996 ▸; Leusen *et al.*, 1999 ▸), as well as systematic build-up of close-packed coordination spheres (Lommerse *et al.*, 2000 ▸; Holden *et al.*, 1993 ▸). The first blind test provided valuable insights into the limitations of existing methodologies and promoted the development of more sophisticated algorithms. For instance, early methods were often limited to a single rigid molecule in the asymmetric unit.

The second blind test (2001) featured two small rigid molecules, for which correct crystal structures were successfully predicted by several groups, and a larger molecule with a freely rotating phenyl group, for which no group could predict the experimental structure. Many different structure generation methods were represented. Various force fields were used to calculate lattice energies. It was noted with some interest that some energy minima were found by more than one group, *i.e.* there was some overlap between predicted landscapes. The second test also included a component where the participants were supplied with experimental powder X-ray diffraction (PXRD) data to aid their predictions.

Similar to the second blind test, predicting the structure of the flexible molecule was largely unsuccessful in the third test in 2004–2005 (Day *et al.*, 2005 ▸). It was concluded that better energy models were needed, capable of simultaneously describing conformational and packing energies with high accuracy. The need for improvements to search procedures for crystals of flexible molecules, or crystals with more than one molecule in the asymmetric unit was also highlighted.

The first few blind tests allowed participants to submit only three candidate structures for each target, with the goal of predicting *the* correct crystal structure among those three. Previous tests might therefore have generated correct structures but they were not submitted. The CSP community has since moved to a viewpoint where we consider whole landscapes of predicted structures, predicting polymorphism rather than a single definite structure, and enabling us to see a wider range of crystalline behaviour, like stacking faults, configurational disorder and polytypism (Reilly *et al.*, 2016 ▸; Addicoat *et al.*, 2018 ▸).

Following the third blind test, van Eijck did a large-scale comparison between submissions where he addressed the issue of search completeness (Day *et al.*, 2005 ▸; van Eijck, 2005 ▸). The overlap between equivalent structures in the submitted sets should indicate the degree of search completeness. Bouke van Eijck found that to a large extent, the various CSP methods produced structures of hydantoin (VIII, CSD refcode: PAHYON) and azetidine (XI, CSD refcode: XATMOV) that were not produced by other methods. That is, the structures produced by one CSP method were in general not found by the other participants. This was a worrying observation and showed that the exploration of the search space was inadequate and most, if not all, methods failed to find many relevant low-energy structures. A similar conclusion was reached specifically for the highly polymorphic compound ROY (CSD refcode: QAXMEH) (Yu, 2010 ▸; Greenwell & Beran, 2020 ▸; Beran *et al.*, 2022 ▸), where it was found that two generally successful CSP methods produced largely disjoint sets of predictions (Nyman & Reutzel-Edens, 2018 ▸). The question of search completeness and whether different CSP methods yield similar structures or not is investigated Section 4.9 with improved comparison methods.

The fourth (Day *et al.*, 2009 ▸) and fifth blind tests (Bardwell *et al.*, 2011 ▸) (2007–2011) demonstrated a significant improvement in the predictive ability of CSP methods, with several groups successfully predicting the experimentally observed structures of ever larger and more complex target molecules. The successes included one participating group (Neumann *et al.*, 2008 ▸) who correctly predicted all four crystal structures as their first ranked choice, albeit at considerable computational expense. The improved success rates observed in these tests were generally attributed to more accurate energy models of putative crystal structures, going beyond classical force fields, with methods such as DFT-D or a hybrid method combining a molecular DFT-D energy with a multipole-based anisotropic intermolecular force field. The most reliable methods for CSP involve massive calculations on the order of millions of CPU-hours and are performed on high-performance computing clusters.

The size and complexity of the target compounds have steadily increased through the blind tests, from simple relatively rigid model systems in the beginning to far more complex molecules and salts selected to represent typical pharmaceuticals or functional materials in the sixth blind test (Reilly *et al.*, 2016 ▸). The sixth test featured a very wide range of structure generation methods, using practically all of the algorithms described above. One new method was presented by a group from the CCDC, which used unit cells taken from the CSD that contained molecules with similar overall shape as the conformers of the target compound (Cole *et al.*, 2016 ▸). Resources employed for predictions in the sixth test increased significantly compared with the previous reflecting the more detailed and demanding searches of conformational and structural landscapes. Additionally, the number of participating groups substantially increased demonstrating growth of the CSP community.

### 1.4. Notable developments since the sixth blind test

Besides the blind tests, the development of structure generation methods was also the subject of a 2018 Faraday Discussion in Cambridge (Addicoat *et al.*, 2018 ▸; Adjiman *et al.*, 2018 ▸). At the meeting, Sarah Price (Price, 2018 ▸), Artem Oganov (Oganov, 2018 ▸) and others discussed the maturity of zeroth order CSP, *i.e.* predictions based on lattice energy alone, the fact that CSP always generates crystal structures that are never observed, and the need for consideration of additional factors that affect polymorph appearance and stability, such as lattice dynamics, relative rates of nucleation, growth and transformation, molecular motion, different kinds of disorder, polytypism and the presence of solvents. Many methods for structure generation were discussed, including, for instance, evolutionary niching as a method to enhance the sampling of crystal structures in genetic algorithms (Curtis *et al.*, 2018 ▸).

One of the main consequences of using zeroth order CSP (Price, 2018 ▸), is the so-called overprediction problem (Price, 2013 ▸), a recurring theme in all blind tests. Thermal effects play a crucial role in this with a single free energy minimum that can correspond to a myriad of static states, in other words, a single thermodynamic ensemble corresponds to several lattice energy minima (Dybeck *et al.*, 2019 ▸). Different approaches have recently been developed to effectively reduce the number of predicted polymorphs, while still retaining those that are likely to be experimentally accessible. Large-scale molecular dynamics simulations supplemented by metadynamics showed that, at realistic temperatures, many of the predicted 0 K energy minima of urea, ibuprofen and succinic acid merge into a much smaller number of thermodynamic ensembles, some of which correspond to real polymorphs (Francia *et al.*, 2020 ▸, 2021 ▸). More recently, threshold Monte Carlo simulations were used to estimate energy barriers between putative crystals, clustering together those below a certain lattice energy cutoff on the order of *kT* (Butler & Day, 2023 ▸).

The seventh test reported here also featured a challenge to solve the structure from a powder X-ray diffractogram, a common, realistic and industrially relevant application of CSP. This kind of problem necessitates the development of robust methods to compare the computationally generated *perfect* structures to the noisy, complicated and often insufficient experimental data collected on real, imperfect materials. For analysing PXRD patterns, many methods exist (Ivanisevic *et al.*, 2005 ▸; Hofmann & Kuleshova, 2006 ▸; Hernández-Rivera *et al.*, 2017 ▸; Suzuki *et al.*, 2020 ▸), but for comparing to CSP-generated structures the similarity score based on cross correlation by de Gelder *et al.* (2001 ▸) has proven particularly useful to several participants. Adjusting the crystal structure in order to maximize the similarity score is a powerful method that allows solving the structure from routinely collected PXRD patterns without the need to determine the lattice parameters by indexing (Altomare *et al.*, 2019 ▸). Variants of the *FIt with DEviating lattice parameters* (FIDEL) algorithm featured prominently in this blind test for the first time (Habermehl *et al.*, 2014 ▸, 2022 ▸).

Experimental crystal structures are increasingly often determined to be disordered, whereas all CSP methods (with the exception of Group 20, see Section 4.5) so far generate only ideal, perfectly ordered structures. Disorder turned out to be a significant confounding factor in the analysis of the results presented in this study. Methods to anticipate and better model disorder, and to account for the associated configurational entropy may be needed (van Eijck, 2002 ▸; Woollam *et al.*, 2018 ▸; Chan *et al.*, 2021 ▸).

### 1.5. Commercial use of CSP and future outlook

The fifth blind test demonstrated that reliable CSP can be performed on molecules approaching the size and complexity of drugs in current development pipelines, and this led to the largest pharmaceutical companies adopting the use of CSP on commercial grounds. The academic curiosity-driven computational experiments of the past (Warshel & Lifson, 1970 ▸; Dzyabchenko, 1984 ▸) have been supplanted by commercially driven enterprises (Neumann *et al.*, 2015 ▸; Nyman & Reutzel-Edens, 2018 ▸; Sekharan *et al.*, 2021 ▸; Sun *et al.*, 2021 ▸; Firaha *et al.*, 2023 ▸). Several of the participants in this blind test are companies offering CSP services. Today, most of the 20 largest pharmaceutical and agrochemical companies use commercial software to perform CSP routinely as a complement to experimental form screens, helping to reduce the risk that late-appearing polymorphism poses to the production, formulation and bioavailability of drugs (Bauer *et al.*, 2001 ▸).

Besides pharmaceuticals, CSP is also applicable to a growing range of functional materials, such as optoelectronic or semiconducting organic molecular crystals (Campbell *et al.*, 2017 ▸; Tom *et al.*, 2023 ▸), microporous crystals (Pulido *et al.*, 2017 ▸; Sugden *et al.*, 2022 ▸; Yang *et al.*, 2018 ▸), energetic materials (Bier *et al.*, 2021 ▸; Arnold & Day, 2023 ▸; O’Connor *et al.*, 2023 ▸), and metal–organic frameworks (Xu *et al.*, 2023 ▸).

A problem not unlike CSP is the prediction of the folding of a protein from its amino acid sequence alone. This has been an important problem for more than 50 years. The 14th Critical Assessment of Protein Structure Prediction, a collaborative blind test of structure solution analogous to this study, showcased remarkable progress made in recent years towards solving this task (Jumper *et al.*, 2021 ▸; Moult *et al.*, 2020 ▸). It is conceivable that large machine learned models trained on crystallographic databases may result in similar breakthroughs also for molecular crystal structure prediction. However, such a model should fulfil the additional requirements of small molecule crystallography, including greater accuracy in atomic positions and the need to predict the relative stability of polymorphs.

In this article we report the results of a large-scale test of crystal structure prediction, showing what is currently possible with state-of-the-art computational methods for blind or experimentally guided prediction of organic molecular crystal structures.

## 2. Motivation, organization and approach

### 2.1. Motivation

The decision to undertake a seventh blind test was driven by two key factors. Firstly, by 2018, it was clear that new methods were appearing in the literature, and feedback from the academic and industrial community indicated that a new test was desirable, as there had been sufficient methodological progress to justify a new test. Secondly, it was clear to the CCDC that crystal structure prediction was gaining significant traction in the pharmaceutical industry on real world problems. Consequently, we decided to undertake a new test that would challenge the community with larger, more complex systems, expand to new chemistries, and introduce industrially relevant problems. New challenges were presented to ensure the test reflected how CSP is being applied in everyday use cases and to encourage further development and innovation. To mirror real-world situations, we deliberately chose to not provide information on the target structures which previously would have been provided, for example the number of formula units in the asymmetric unit (*Z*′) or stoichiometries of multicomponent structures. We also allowed the inclusion of disordered structures where the disorder was localized within a specific area of the molecule, though participants were not informed to expect disorder nor were predictions of disorder requested.

### 2.2. Organization

The format of the seventh blind test was shaped by feedback from the sixth blind test, and coordinated by Lily M. Hunnisett (CCDC). The test followed a two-phase process to reflect the two main components of CSP methodology: structure generation and structure ranking. The two phases ran from October 2020 to June 2022, and an in-person meeting was held in September 2022 (Cambridge, UK) to present and discuss the results.

Given the size and scope of the current challenge, the two stages are published as separate reports. This current publication reports on the first phase, structure generation, where the objective was to assess whether the experimental crystal structures had been generated by different CSP methods. Relative stability rankings of CSP structures were not requested for this exercise (unless stated otherwise), and in those cases where ranking data were provided, they were not considered in the assessment of successful structure predictions but were utilized to select the lowest ranked 100 structures for landscape similarity analysis between groups.

### 2.3. Choice of target compounds

In order to judge the suitability of systems provided by the pharmaceutical and agrochemical industries, CCDC reached out to active members of the CSP community who had participated in multiple previous blind tests with a selection of two-dimensional chemical structures selected from the CSD. Individuals were asked to comment whether the complexity and/or chemistry were deemed to be easy or difficult with respect to their own CSP methods currently under development. The answers guided the organizers in the subsequent selection of target compounds for this test. None of the molecules ultimately chosen were shown to any of the community as part of this exercise.

CCDC organizers then reached out to the crystallographic academic community and industry to source suitable unpublished crystal structures of a similar nature. The target compounds for the seventh test are tabulated in Table 1 ▸, and were numbered following the scheme set by previous blind tests. The targets were chosen in consultation with an external referee, Richard I. Cooper (University of Oxford), to provide challenges of a range of aspects which broadly fit into one of the two categories: methods development (molecules XXVII–XXX) and pharmaceutical/agrochemical applications (molecules XXXI–XXXIII).

The methods development category presented wider applications, diverse chemistry, and industrially relevant challenges. The pharmaceutical/agrochemical category tested the limits of computational capacity by inclusion of pharmaceutical or agrochemical-like substrates. Since information relating to crystallization conditions, aside from temperature, was not utilized by any CSP method in the sixth blind test (Reilly *et al.*, 2016 ▸), such information was not provided to participants.

Systems were sought with at least one structure determined from single crystal X-ray diffraction. While thorough experimental characterization and solid-form screening were crucial for the selection of targets in the pharmaceutical/agrochemical category, the choice of systems for the methods development category was driven by presenting relevant challenges. Subsequently, solid-form screening was carried out by experimental collaborators during the test for targets XXVII–XXIX, which had not yet undergone comprehensive screening.

### 2.4. Overview of selected target compounds

A brief description of the experimental determination of the compounds is given in this section, while detailed reports are available in SI-C.

#### 2.4.1. XXVII

**Figure 1:**
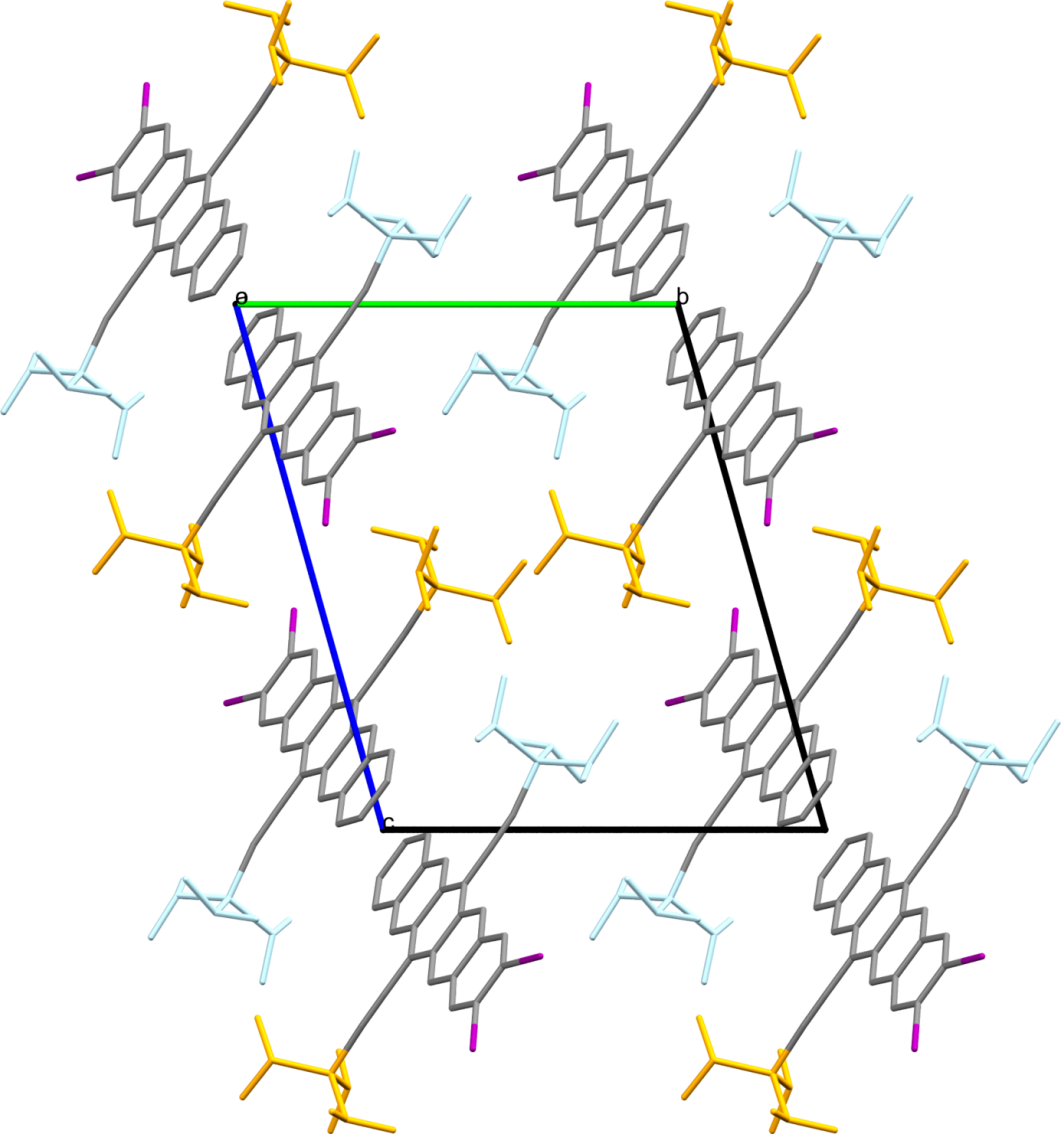
Crystal packing of XXVII Form A at 90 K excluding the bromine atoms from an impurity, and highlighting the two TIPS groups: A (orange) and B (blue). Hydrogen atoms were omitted for clarity.

Molecule XXVII [(2,3-diiodopentacene-6,13-diyl)bis(ethyne-2,1-diyl)]bis(triisopropylsilane) is a silicon and iodine-containing molecule with optoelectronic applications. The crystal packing of these compounds is crucial to their functionality. There exists one known crystal structure of XXVII, Form A, which crystallizes in the 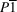 space group with a single molecule in the asymmetric unit, see packing diagram in Fig. 1 ▸.

An initial crystal structure of Form A was obtained prior to the start of the test (September 2020), collected at 90 K from a small blue plate-shaped crystal grown from a dichloromethane solution. The structure is available in SI-D, and a full report is provided in Section 1 of SI-C. In July 2022, following the submission deadline of the test, an additional crystal structure of Form A was provided by John E. Anthony and Sean Parkin (University of Kentucky). This structure was collected at 290 K and exhibited disorder of one of the triisopropylsilyl (TIPS) groups (CSD refcode: XIFZOF). In May 2023, a re-refinement of the original 90 K structure was received from the experimental providers, where the structure had been refined as having an elemental iodine/bromine disorder (CSD refcode: XIGYUL). That is, the structure has a substantial bromine contamination originating from the synthesis. To confirm that the bromine impurity does not significantly affect the overall crystal structure and the analysis of the CSP results, the structure was eventually redetermined from pure material by the providers, also in May 2023, with diffraction data collected at 100 K (CSD refcode: XIFZOF01). While this structure contained disorder in both TIPS groups, limited deviation of the overall geometry was observed.

A crystal form screen was carried out during the test by Joanna A. Bis, Stephen Carino, and Frank Tarczynski (Catalent) which was comprised of ∼150 crystallization experiments and involved 48 solvents, three crystallization modes (slurry ripening, rapid cooling, and slow evaporation), and a temperature range of 278–313 K. This resulted in an additional anhydrous form being identified via PXRD (Form B) in addition to the already known form (Form A), though the crystal structure of Form B could not be determined. Competitive ripening studies indicated Form A is more stable at 278 K and Form B is more stable between 293 K and 313 K, *i.e.* Forms A and B exhibit an enantiotropic relationship with a transition temperature between 278 and 293 K. Attempts at indexing, simulated annealing and FIDEL by both the organizers and some participant groups were unsuccessful at conclusively determining the crystal structure of Form B.

#### 2.4.2. XXVIII

**Figure 2:**
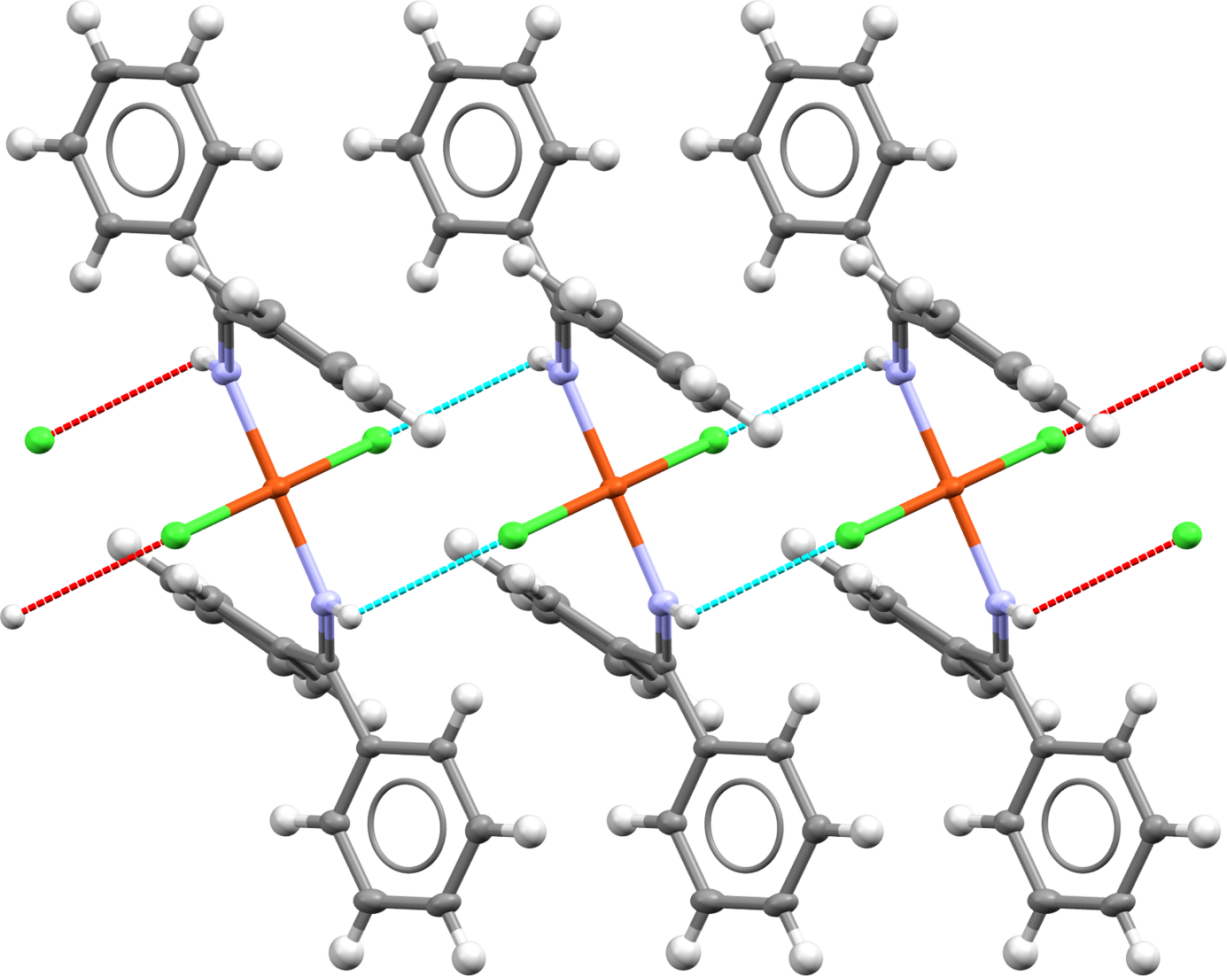
Stacked molecules of XXVIII, viewed along the crystallographic **c** axis, showing the weak intermolecular H⋯Cl hydrogen bonds detected at a distance of the sum of van der Waals radii plus 0.1 Å.

Molecule XXVIII is a copper coordination complex, dichlorido-bis(1,1-diphenylmethanimine)copper(II), with optical applications. The inclusion of copper presents an uncommon challenge for CSP methods. There exists one known crystal structure of XXVIII (Form A, CSD refcode: OJIGOG01). The molecule exists in a *trans* square-planar geometry (Fig. 2 ▸ ▸). The compound crystallizes in the triclinic crystal system, space group 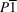, with *Z*′ = 0.5 and the copper atom on the inversion centre. Crystals of XXVIII were grown from a diethyl ether/dichloromethane solution and data collected at 150 K.

A search of the CSD identified a number of structural analogues (CSD refcodes: KAYPEG, WIFVUD, NIQXEQ, NIQXEQ01). None of the analogues exhibit any similarity in crystal packing to Form A, though the sulfur analogue (NIQXEQ) shows that this type of system can be polymorphic, existing in both square planar and non-square planar geometries.

A crystal form screen was carried out by Michael R. Probert and Jake Weatherston (Newcastle University) employing the Encapsulated Nanodroplet Crystallization (ENaCt) method (Tyler *et al.*, 2020 ▸). This comprised 20 different organic solvents in combination with four inert oils (plus no oil). Crystallization was assessed by cross-polarized optical microscopy and suitable crystals harvested for unit cell determination. All crystallization from ENaCt plates resulted in oxidative dimerization of the ligand with no observed crystallization of the desired complex.

#### 2.4.3. XXIX

Molecule XXIX (methyl 2-aminobenzoate, a liquid at room temperature (RT)) is a simple molecule with limited flexibility which possesses three symmetrically independent molecules in the only known form (Form A, CSD refcode: FASMEV). This presented a complex challenge as it is uncommon for CSP methods to search beyond *Z*′ = 2 due to computational cost. This target compound was presented as a PXRD-assisted challenge where a simulated PXRD pattern (Fig. 1 in SI-A) was provided alongside the two-dimensional chemical structure (see Section 2.6 for further details). If the PXRD pattern could be successfully indexed this would have revealed the structure to be *Z*′ = 3 at the outset. Crystals of Form A were grown by scratching the supercooled liquid sample with a needle after cooling it in a cold room. The structure crystallizes in the *P*2_1_/*c* space group and data were collected at 274 K.

Since the compound requires low temperatures for crystallization and is liquid at RT, options for high-throughput polymorph experiments are limited and less conventional methods for exploration were employed. High-pressure crystallization was carried out by Michael R. Probert and Jake Weatherston (Newcastle University), where pressure was oscillated around the initial crystallization pressure to selectively melt and grow crystals until an individual single crystal large enough for analysis was observed in the cell. The experiments resulted in no additional forms.

#### 2.4.4. XXX

**Figure 3:**
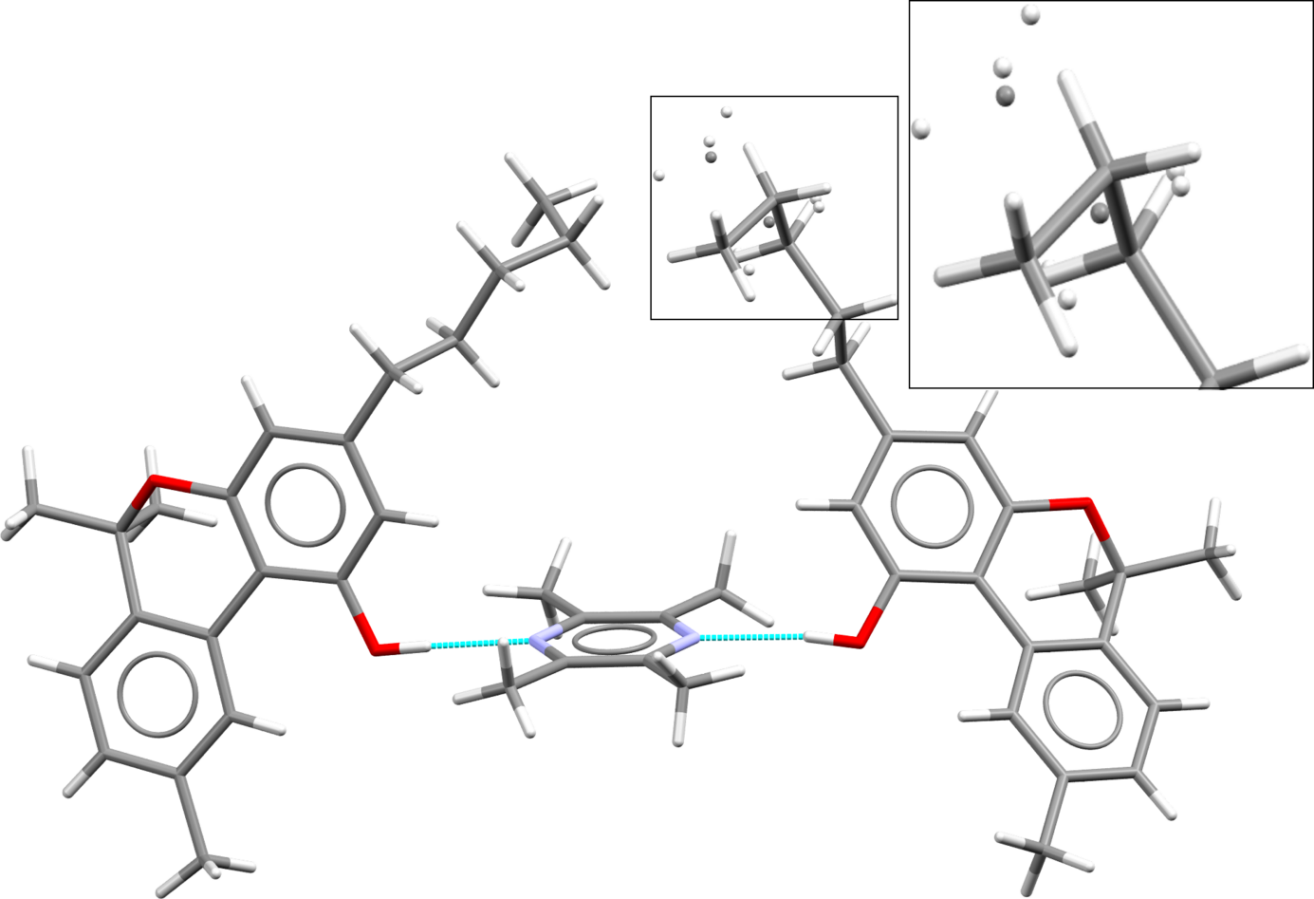
Molecules in the asymmetric unit of XXX Form A, highlighting the disorder observed in the alkyl chain.

Target system XXX consists of 6,6,9-trimethyl-3-pentyl-6*H*-benzo[*c*]chromen-1-ol, more commonly known as cannabinol (CBN), and 2,3,5,6-tetramethylpyrazine (TMP), that are known to crystallize into two different cocrystals of differing stoichiometry: Form A (2:1 CBN:TMP, CSD refcode: MIVZEA) and Form B (1:1 CBN:TMP, CSD refcode: MIVZIE) (Mkrtchyan *et al.*, 2021 ▸). Unbeknownst to the participants, Form A exhibits disorder of the cannabinol alkyl chain (Fig. 3 ▸).

Crystals of Form A were prepared by combining 20 mg cannabinol with heptane (100 µl) and tetramethylpyrazine (3 M in methanol; 87 µl; 4 molar equivalents). Solvent was removed and the sample resuspended in heptane (100 µl), then seeded with the hemicocrystal until precipitation occurred. Form A crystallizes in the *P*2_1_/*c* space group. Data were collected at 100 K. One of the molecules in the asymmetric unit contains disorder of the alkyl chain due to the rotation of the two dihedral angles located at the end of the chain, resulting in two conformational components with occupancies of 0.888 (Form A_maj_) and 0.112 (Form A_min_), respectively, see Fig. 3 ▸.

Crystals of Form B were prepared by combining cannabinol (162.1 mg) with solid tetramethylpyrazine (142.6 mg, 2.0 molar equivalents) and solvent (isooctane, 750 µl) and stirred at RT for 20 h. Form B crystallizes in space group *P*2_1_/*n*. Data were collected at 100 K.

The crystal form screens of the hemi- and monococrystals carried out by Joanna A. Bis, Stephen Carino, Ricky Couch (Catalent) were each comprised of ∼105 crystallization experiments and involved 35 solvent systems and three crystallization modes (slurry ripening, cooling, evaporation) over a temperature range of 278–298 K. The evaporative cocrystal form screens produced an additional unstable solid appearing to be a cannabinol tetramethylpyrazine cocrystal, see Section 4 in SI-C. This new solid, labelled ‘Group E’ in the solid form screen report, could not be reproduced by other methods and experimental attempts to determine the stoichiometry were unsuccessful, it was therefore not considered a target structure for this blind test. Further work by Group 20 (see Section 14 in SI-B) determined the likely stoichiometry of Group E to be 1:1 by indexing the PXRD pattern; this was confirmed independently by the organizers. The determination and comparison of the Group E PXRD data with CSP structures were beyond the scope of this test.

Due to reasons relating to an associated patent application (WO2021138610A1), an earlier deadline (June 2021) was set for participants to submit results to the organizers. In addition to submitting 1500 structures including all stoichiometries, each group submitted a list of 100 structures ranked in order of likelihood of observation. Since this requires ranking of structures containing differing stoichiometries, this was a challenging exercise for CSP methods, which are predominantly based on relative energies of crystals of the same composition.

#### 2.4.5. XXXI

Molecule XXXI, 3-((difluoro-(2-fluorophenyl)methyl)sulfonyl)-5,5-dimethyl-2l2-isoxazolidine, is a simple agrochemical compound with three rotatable bonds. There are three known crystal forms (Forms A–C), where Form A is disordered via the rotation of the *ortho*-fluorophenyl ring and Form C is a porous structure which contains void channels (likely a solvate where solvent molecules could not be resolved). It was not expected that Form C would be present in the limited sets of submitted CSP structures, since the porous host structure is likely to be relatively high in energy.

A polymorph screen was conducted by John Hone, Adam Keates and Ian Jones (Syngenta) prior to the organizers acquiring the crystallographic data. This involved performing high-throughput evaporative, drown-out, cooling and temperature cycling crystallizations in 28 different solvents and solvent mixtures. After a total of over 400 crystallizations, this screen produced three polymorphs (Forms A–C) with the resulting single-crystal structures being solved at 120 K, 200 K and 120 K, respectively.

Crystals of Form A (CSD refcode: ZEHFUR02) were grown from methanol by evaporation. The system crystallizes in space group *P*2_1_/*c* with one molecule in the asymmetric unit. The *ortho*-fluorophenyl ring is disordered over two sites with configurations, denoted Form A_maj_ and Form A_min_, in a 60:40 ratio.

Crystals of Form B (CSD refcode: ZEHFUR), crystallizing in space group *P*2_1_/*c* with one molecule in the asymmetric unit, were grown by temperature cycling an aqueous suspension.

Crystals of Form C (CSD refcode: ZEHFUR01) were grown from a surfactant/solvent mixture with temperature cycling, crystallizing in space group 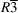. Typical of this space group, the solvent templated structure contains void channels that run parallel to the **c** lattice vector, see Fig. 2 in SI-A. A *PLATON* SQUEEZE function (Spek, 2015 ▸) was applied because no ordered solvent could be identified.

Slurry experiments were carried out to determine relative stability relationships between polymorphic forms. Equal amounts of Form A and Form B were stirred together in a water/methanol mixture over a range of temperatures (298–353 K). Form B was found to be more stable than Form A at 346 K and below. A mixture of both Form A and Form B was identified at 353 K, indicating a transition to Form A at around 353 K.

Equal amounts of Form B and Form C were suspended in a water/methanol solution and stirred at both 278 K and RT. All experiments showed conversion to Form B, showing that Form B is more stable than Form C at least over this temperature range. This is expected as only certain solvents can stabilize the porous crystal structure of Form C.

#### 2.4.6. XXXII

Molecule XXXII (*N*-(3-[2-(difluoromethoxy)-5-(methylthio)phenyl]-1-[2-(4-morpholinopiperidin-1-yl)-2-oxoethyl]-1*H*-pyrazol-4-yl)pyrazolo-[1,5-*a*]pyrimidine-3-carboxamide) is a large pharmaceutical compound with eleven rotatable bonds. There are eight claimed anhydrous forms showed through PXRD, only two crystal structures of which are resolved (Forms A and B). The crystal structure of Form A contains disorder via rotation of the difluoromethyl group. Experimental efforts throughout the course of the test to determine the remaining crystal structures were unsuccessful. With its large number of degrees of freedom, XXXII was considered the most challenging benchmark of CSP methods in terms of computational cost and efficiency.

Crystals of Form A (CSD refcode: JEKVII) were grown from an ethyl acetate solution of XXXII followed by vapour diffusion of isooctane. The structure crystallizes in space group 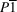 with one molecule in the asymmetric unit, and contains disorder of the difluoromethyl group. Data were collected at 90 K.

Crystals of Form B (CSD refcode: JEKVII01) were grown from the slow cooling of a hot toluene solution. The structure crystallizes in the 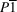 space group with two molecules in the asymmetric unit. Data were collected at 90 K.

Target XXXII was screened for polymorphs by the experimental provider, Antonio DiPasquale (Genentech), prior to the blind test. The solid form screen, surveying 80 conditions through methods of anti-solvent addition, evaporation, slow cooling, slurry conversion, and vapour diffusion, produced 25 crystal forms of this model pharmaceutical compound. Among the forms were eight anhydrous polymorphs, four hydrates, six organic solvates and seven transient or unconfirmed forms, all identified by PXRD. The crystal structures of Forms A and B have been determined by single crystal X-ray diffraction at low temperature (90 K). Further attempts by the experimentalists to determine the crystal structures of the other forms were unsuccessful. The propensity for XXXII to form solvates was high in a screen that was not designed to include desolvation experiments, so it is not certain that all anhydrous forms have been found (see Section 6 of SI-C).

Stability relationships of all the anhydrates were established via competitive slurries at different temperatures from RT (298 ± 3 K) to 373 K, where Form B was confirmed to be the stable anhydrate in this temperature range.

Further experimental exploration during the blind test resulted in an additional RT PXRD pattern of Form B, which was initially indexed by the experimental providers in the monoclinic space group *P*2_1_/*c*, *i.e.* a higher symmetry than the low-temperature (LT) variant of Form B. Through assessment of predictions and working together with the experimental provider and Group 20, the crystal structure solution of the RT form was shown to be incorrect and a redetermination obtained. Form B is a 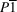 structure, however the extremely high similarity between the LT and RT structures of Form B produced ambiguous matching results and so the latter structure was not reported as a separate target for this test (see Section 4.7 for further details).

#### 2.4.7. XXXIII

Target XXXIII, a 1:1 morpholine salt of 4-amino-*N*-(5-methylisoxazol-3-yl)-benzenesulfonamide (or sulfamethoxazole for short), has two known forms: Form A (CSD refcode: ZEGWAN) and Form B (CSD refcode: ZEGWAN01). Form A is a disappearing polymorph, presenting an exercise of high relevance to industry. The site of deprotonation was made known to participants via the 2D chemical diagram provided.

**Figure 4:**
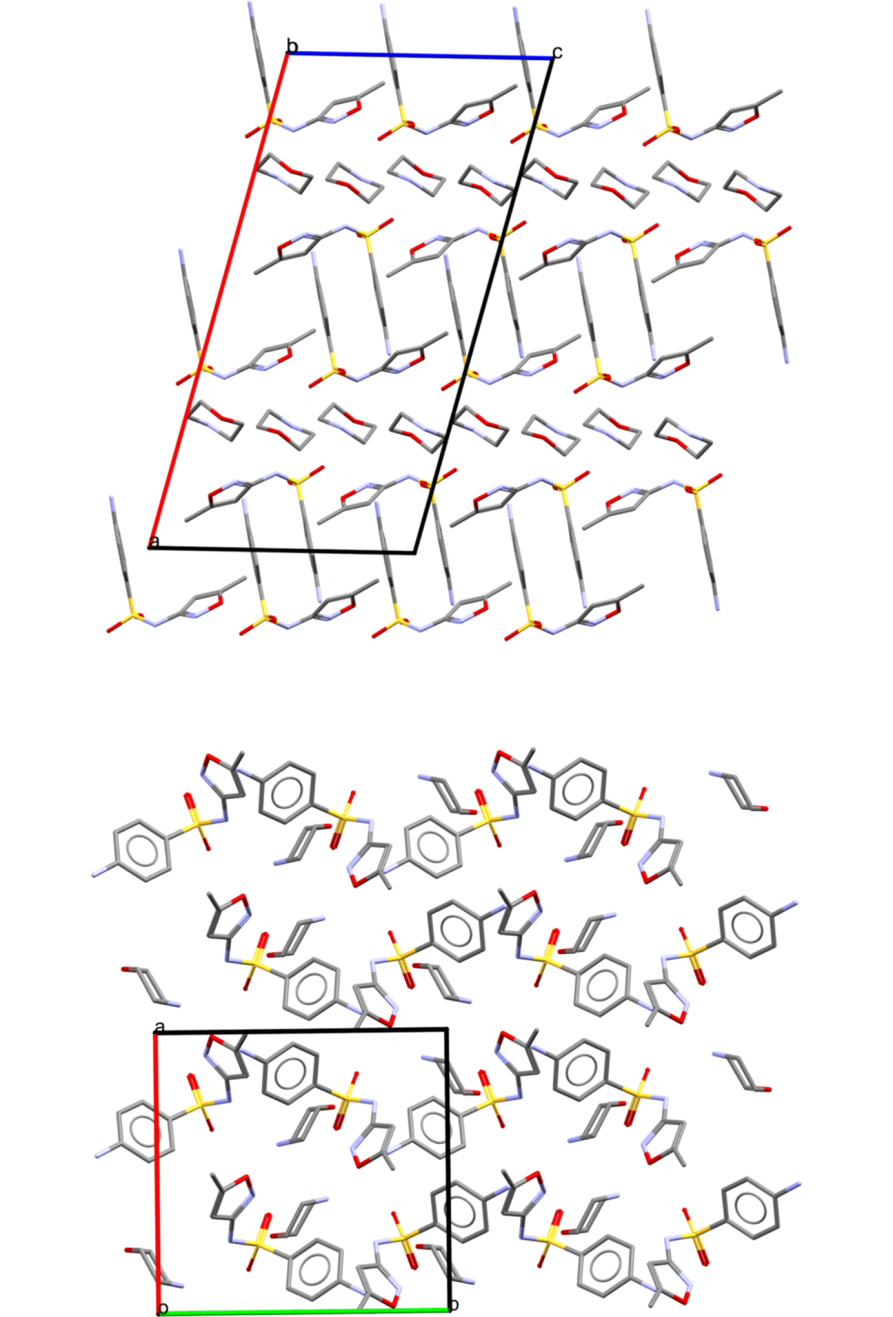
Crystal packing motif of XXXIII Form A (top) and Form B (bottom). Hydrogen atoms were omitted for clarity.

Initial crystallization in a morpholine acetonitrile solvent mixture at RT produced large block-shaped crystals, Form A, crystallizing in the monoclinic space group *C*2/*c* with one of each ion in the asymmetric unit. The proton transfer is involved in the formation of a tetrameric motif, see Fig. 4 ▸. Data were collected at 296 K. Form B belongs to the ortho­rhombic space group *Pna*2_1_ and has one formula unit (two ions) per asymmetric unit. The crystal structure of Form B contains zigzag chains of sulfamethoxazole connected via morpholine molecules. The ability of the protonated morpholine to form two separate hydrogen bonds is integral to maintaining the chains, which are arranged in a head-to-tail arrangement with neighbouring sulfamethoxazole molecules along the crystallographic *a* axis, see Fig. 4 ▸. Data were collected at 297 K.

Subsequent repeat experiments afforded large prismatic crystals, Form B, and all further attempts to reproduce Form A failed, as both repetition of the initial experiment and alternate methods yielded Form B only, that is, Form A may be a disappearing polymorph (Dunitz & Bernstein, 1995 ▸; Bučar *et al.*, 2015 ▸). In both cases, a proton transfers from the sulfon­amide nitrogen to morpholine, producing the salt form.

Polymorph diversity was investigated experimentally by Joseph Cadden, Simon Coles and Srinivasulu Aitipamula via solid-state grinding methods. Solvent-drop grinding was performed in the presence of sulfamethoxazole, morpholine and trace amounts of organic solvents of different polarity. Form B was confirmed by PXRD as the only product from all screening experiments.

### 2.5. Format of phase one: structure generation

Researchers who expressed interest in taking part in the test were first asked to provide details of the proposed methodology for the exercise to ensure all groups applied a method stemming from either published original research or previously benchmarked approaches. The two-dimensional chemical diagrams and supporting information, see Table 1 ▸, including data requested by the organizers were sent to all participants on 27th October 2020. Each group was invited to return predictions to the organizers within one year. Changes or withdrawals of submitted data were accepted only before this date. There was no requirement to attempt predictions for all target structures. For each target compound, a list of up to 1500 generated structures was submitted by each participating group to be checked by CCDC organizers for matches to the known experimental structures.

### 2.6. Pushing the boundaries: new features in this CSP blind test

The seventh blind test presented new and relevant challenges to CSP methods, the key differences to previous blind tests being:

(*a*) splitting the test into two parts; structure generation and structure ranking methods were assessed separately, the latter involving a standardized set of structures;

(*b*) the analysis of larger sets of structures (up to 1500, compared to 100 in the sixth blind test);

(*c*) the inclusion of challenging chemistry (target XXVII: an Si- and I-containing optoelectronic compound, target XXVIII: a Cu complex);

(*d*) the additional challenge: ‘Can CSP determine a crystal structure from a low-quality PXRD pattern?’;

(*e*) the additional challenge: ‘Can CSP correctly predict the most likely stable stoichiometry of a cocrystal?’

Structure XXIX was presented as a PXRD-assisted exercise; a PXRD pattern representing the known crystal structure was provided alongside the two-dimensional chemical structure and participants were asked to submit a list of ten predicted structures that could be represented by the PXRD pattern, ranked in order of likelihood of observation. The provided PXRD pattern was simulated from the experimental crystal structure of XXIX by Jason C. Cole (CCDC) and Kenneth Shankland (Reading University), and intentionally made to be of low quality by introducing complex background, background noise and broadening of the peaks to emulate a situation commonly encountered in present-day solid-form pharmaceutical projects where a crystal structure cannot be resolved from experiment. Additionally, PXRD patterns were provided in low-resolution image format only to simulate a real-world use case encountered when compounds are acquired or transferred across companies or institutions, or data are retrieved from older publications or patent documents. The purpose of this exercise was to test whether CSP methods can successfully resolve a crystal structure where experimental methods may fail.

Structure XXX was presented as a stoichiometry prediction exercise to assess the capability of CSP methods to predict the most likely observed structures among different compositions. Alongside the two-dimensional chemical structure, participants were advised that two known forms exist with different stoichiometries, and where the ratio of cannabinol to tetramethylpyrazine can be any two of the following: 1:1, 1:2, 2:1. In addition to a list of 1500 structures, participants were asked to submit a list of 100 predicted structures ranked in order of likelihood of observation, and a statement reporting the two most likely stoichiometries to be observed based on the CSP results submitted.

### 2.7. Assessment of predictions

The crystal structures submitted by participants were compared against the experimental structures using the molecular overlay method, commonly known as COMPACK (Chisholm & Motherwell, 2005 ▸), and since implemented as *Crystal Packing Similarity*, available through *Mercury* and the CSD Python API (Macrae *et al.*, 2020 ▸; Groom *et al.*, 2016 ▸). This method overlays, within given distance and angle tolerances, clusters of molecules taken from each crystal and minimizes the root mean square distance (RMSD) between atoms, typically omitting hydrogen. The method thus returns the number of molecules that could be overlaid and the RMSD. When comparing crystal structures with this method, space group symmetry and unit cell parameters are ignored, so structures with missed symmetry or unconventional unit cells are allowed and recognized as matches.

The PXRD pattern similarity measure developed by de Gelder *et al.* (2001 ▸) and available in the CSD Materials module of the *Mercury* (Macrae *et al.*, 2020 ▸) program has also been employed here to compare simulated PXRD patterns of crystal structures.

An investigation by Sacchi *et al.* (2020 ▸) into structural similarity in the CSD involving comparisons of thousands of CSD crystal structures using COMPACK and PXRD pattern similarity indicated that in the majority of cases, both methods are effective for the identification of matching structures. However, limitations were attributed to temperature and pressure effects in addition to high sensitivity to the tolerance values specified in COMPACK comparisons, highlighting the importance of considering additional structural comparison methods. Recent advances following the sixth blind test have resulted in alternative methods for efficient and accurate crystal structure comparisons (Mayo *et al.*, 2022 ▸; Nessler *et al.*, 2022 ▸; Widdowson & Kurlin, 2022 ▸).

The distance and angle tolerances applied in COMPACK comparisons to determine a match were intentionally set higher than in previous blind tests. This was to reflect the assessment of structure generation methods to produce a structure resembling that of an experimental structure prior to the utilization of more refined geometry optimization methods using higher levels of theory. Where disorder was present, the structure was split into two components and predicted structures compared against each. Comparisons were carried out in an automated fashion utilizing the CSD Python API. Each comparison followed the protocol below unless stated otherwise:

(*a*) Perform a PXRD pattern similarity comparison (patterns simulated from crystal structure). If the similarity is higher than 70%, then continue, or else the structures are considered dissimilar.

(*b*) Perform a COMPACK comparison with a molecule shell of 30 molecules and distance and angle tolerances of 35% and 35°, where hydrogen atoms were not included, and molecular differences were not allowed.

(*c*) If the number of molecules overlaid was 30, and RMSD < 1.0 Å, we consider the structures to match. The comparison was visualized in *Mercury* to confirm the structural match. Visualizations of confirmed matches were saved as images and are available in Section 1 in SI-A.

## 3. CSP methodologies submitted

Across 22 participating groups, a range of methods were applied, which follow the same general workflow: (i) Molecular conformational search, (ii) Crystal structure generation, (iii) Structure ranking. The methods are presented in Table 2 ▸.

The molecular conformational search methods included quantum mechanical (QM) torsion energy scans, the use of CSD data to inform the search, and chemical intuition. Only one group specified a rigid search method in this stage. Other methods employed systematic or genetic algorithms. Quantum chemical energy methods were used in the majority of cases in addition to force field methods.

The majority of structure generation methods employed a random or quasi-random search method. A few groups employed a grid search, and others included parallel tempering, evolutionary search, and rigid stochastic surface walking methods (Huang *et al.*, 2019 ▸).

The structure ranking methods applied in this phase of the test were most commonly force field based, either a predefined potential, or a tailor-made or machine-learned force field. A handful of groups also employed periodic QM methods to analyse energetics in this stage. Seven groups mentioned the use of both intra- and inter-molecular contributions to their energy scoring. One group also applied molecular dynamics (MD) simulations to reduce the energy landscape.

Overall, structure generation protocols applied in this test are similar to those reported in the sixth blind test. A detailed description of the methodologies applied by each group is available in SI-B.

## 4. Results and discussion

### 4.1. Submitted results

The seventh blind test saw participation from a total of 28 groups. Out of these, 22 submitted results in the first, *i.e.* structure generation, phase of the test. A summary of the participating groups for each target compound and their success rates is given in Table 3 ▸. The submitted raw data is available in SI-D.

Molecule XXIX received the most attempted predictions with 19 groups taking part in the PXRD-assisted exercise. Target molecule XXVIII received the fewest submissions with only eight groups attempting predictions, though this is likely due to the crystal structure having been published independently while the test was ongoing, which resulted in some groups stopping their efforts towards this system since it was no longer a blind test. In this case, the organizers allowed groups to still submit their predictions, and the results for this target molecule are reported here, though with full disclosure that the experimental structure was freely available prior to the submission deadline.

A description of the experimental crystal structures and results from the analyses by the organizers is reported here for each target molecule. A summary of results from the COMPACK comparisons for the methods development and pharmaceutical/agrochemical categories is provided in Tables 4 ▸, 5 ▸ and 6 ▸. Further data and information are included in Section 1 of SI-A.

### 4.2. XXVII

There is one known, experimentally resolved form of XXVII (Form A). While additional experimental structures of Form A were obtained after the test, as outlined in Section 2.4, this section reports on the analysis of the original structure determined at 90 K (structure available in SI-D), prior to the knowledge of a bromine impurity in the material and the acquisition of additional crystal structures. Evidence of an additional polymorph (Form B) emerged from a crystal form screen. However, further investigations to determine the crystal structure of Form B were beyond the scope of this study, so the analysis focused on Form A only.

The high topological symmetry of molecule XXVII resulted in large computational resource requirements for comparisons of structures using the COMPACK algorithm. Comparisons to identify predicted structures matching the experimental form were initially performed following the submission deadline resulting in one potential match, which was a structural variant of Form A differing in the conformation of an isopropyl group. However, during final analyses in August 2022, alternative crystal structure comparison methods (Widdowson *et al.*, 2022 ▸; de Gelder *et al.*, 2001 ▸) highlighted other highly similar structures present in the submitted lists. Comparisons with the variable-cell powder difference approach (Mayo *et al.*, 2022 ▸), available in the *critic2* program (Otero-de-la-Roza *et al.*, 2014 ▸), were later carried out and presented analogous results. Due to an internal limit to the maximum number of comparisons arising from topological symmetry having been exceeded with the CCDC implementation of COMPACK, the initial matching results were deemed incorrect. The *Crystal Packing Similarity* code was then updated to allow for all possible comparisons of the molecule; subsequent comparisons resulted in matching structures from six groups (10, 16, 20, 21, 24, and 25), see Table 4 ▸. It is noted that three groups (5, 21, 24) specified the use of the *Crystal Packing Similarity* code to identify and remove duplicate structures so it is possible that the limitation within the tool could have led to incorrect filtering of results. However, since the limitation resulted only in false negatives, this would not lead to a correct structure being removed. The update to the *Crystal Packing Similarity* tool has since been incorporated into recent CCDC software releases, demonstrating one of the purposes of this initiative in identifying and implementing improvements by challenging current methodologies and tools.

A search of the CSD for similar structures shows that a bromine analogue of Form A of XXVII has been published (CSD refcode: TATLOQ) (Swartz *et al.*, 2005 ▸), and a comparison of this with the initial experimental Form A (25%/25° distance/angle tolerance) suggests the crystal packing is highly similar with a 19/20 molecule match, 0.506 Å RMSD. It was expected that this available experimental structure would provide an advantage to CSP methods by providing a hint at the correct core packing of the system. Of the 14 methodologies submitted, three groups (8, 21 and 24) mentioned the use of the CSD within their workflow; Groups 8 and 21 utilized the conformation of TATLOQ, while Group 24 used only TIPS conformational information from the CSD. Two of the three groups (Groups 21 and 24) submitted the correct experimental structure.

Following the final deadline of the test, it was reported by the experimental providers of molecule XXVII that disorder of the TIPS groups was observed in the crystal structure at higher temperatures. Additionally, Group 24 reported at the time of results submission that MD simulations, performed at 300 K, had indicated dynamic disorder of the TIPS groups. This was also later reported by Group 10 from follow-up studies. It was noted during discussions with the experimental group that the desired properties of systems such as XXVII with optoelectronic applications are attributed to the crystal packing with emphasis on the orientation and distances between the core atoms of the molecule, *i.e.* the fused aromatic rings. The results of comparisons excluding the tri­isopropyl groups from both reference and comparison structures (applying a cluster of 30 molecules with 35% and 35° distance/angle tolerances) are therefore reported to indicate which methods were successful in generating a structure with the correct crystal packing (Table 4 ▸). As a result, two additional groups (5 and 6) submitted structures matching the crystal packing of Form A. There are a large number of possible conformational polymorphs due to six isopropyl groups in the molecule. Since the changes in conformation do not translate to a large change in RMSD, it is possible that in some cases the structure clustering process, if based on RMSD, may have filtered out the correct conformational polymorph matching Form A, adding further relevance to core-only comparison results.

Clustering of each submitted landscape based on the core packing, applying a standard clustering algorithm together with COMPACK, resulted in vastly different degrees of common crystal packing populations across the different groups, see Table 16 in SI-A. The presence of large clusters was likely a result of strict clustering criteria that allowed for a wider range of TIPS group conformations to be examined. On the other hand, loose clustering criteria led to smaller clusters, meaning the groups explored more diverse packings of the core atoms. However, this approach may have caused the experimental structure to be discarded as a duplicate when different TIPS conformations were not detected.

Form A was further investigated by the CCDC through molecular dynamics (MD) and enhanced sampling simulations. The focus of this study was on the disorder, being dynamic or static, related to the bending of the C—Si—C angles, the rotation of the two TIPS groups around the silicon atom, and the rotation of isopropyl groups. For this purpose, a 100 ns MD simulation followed by two 1 µs long metadynamics simulations (one for each TIPS group) were performed at room temperature and pressure. MD simulations were carried out in *GROMACS* (Lindahl *et al.*, 2020 ▸) and conducted using the General Amber Force Field (GAFF) (Wang *et al.*, 2004 ▸) with the bonded terms involving silicon atoms parameterized based on *ab initio* calculations at the MP2/6-31G(d) level (Francia, 2022 ▸). Further computational details with the description and analysis of each step are available in Section 3 of SI-A.

**Figure 5:**
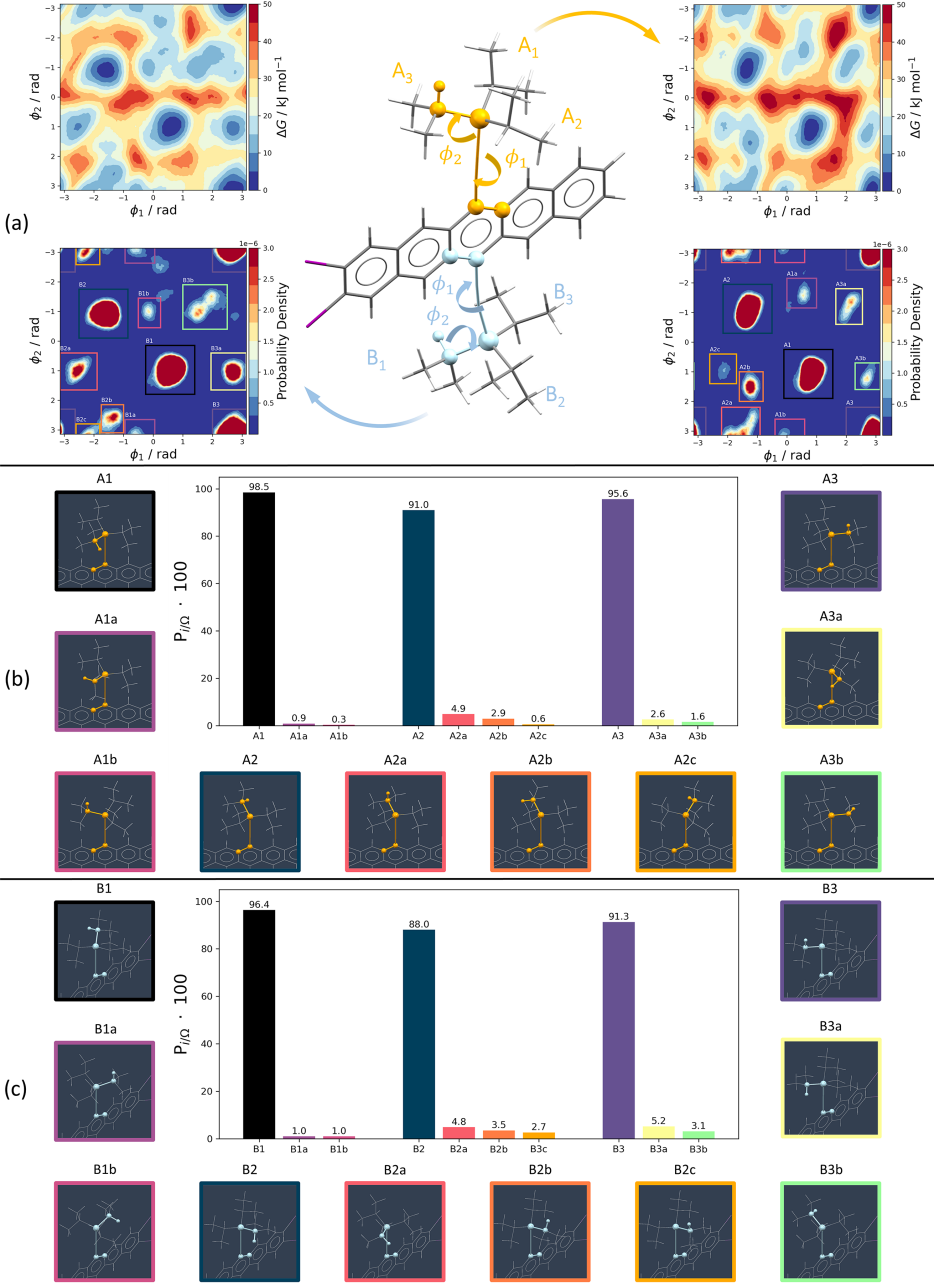
Molecular dynamics (MD)-based analysis of molecule XXVII conformational ensemble at finite temperature. (*a*, top) Free energy surfaces corresponding to TIPS A (orange) and B (blue) obtained by biasing the angles ϕ_1_ and ϕ_2_ shown in the middle. These show different behaviour in both basin shapes and locations. (*a*, bottom) Equilibrium probability distributions derived from the free energy surfaces with bounding boxes used to calculate the equilibrium probability of each conformational state. These were further divided into three regions over ϕ_1_ to account for the configuration of the three isopropyl groups. This results in equilibrium probabilities reported in panels (*b*) and (*c*), where the molecular structure of the main conformers is shown associated with the colour corresponding to the appropriate bounding box.

The MD trajectory shows a different behaviour of the two TIPS groups, with one, here labelled B, that is able to rotate more easily while the other, labelled A, is more sterically hindered by the packing. These differences in the conformational flexibility of the two TIPS groups were characterized by representing the accessible configurations as a function of two torsional angles, indicated as ϕ_1_ and ϕ_2_, and shown in Fig. 5 ▸(*a*). For each isopropyl group: ϕ_1_ detects the position of the isopropyl group with respect to the pentacene, while ϕ_2_ is the orientation of the isopropyl group in the TIPS group.

The conformational exploration obtained with unbiased MD also saw the emergence of distorted conformations, especially involving the B TIPS group, obtained from the rotation of ϕ_2_. The late appearance of such configurations suggests that timescales vastly exceeding 100 ns are needed to estimate the impact of the different conformations on the room-temperature crystal. To overcome the MD timescale limit and identify the equilibrium population of each conformer, we used well tempered metadynamics (WTMD) simulations (Barducci *et al.*, 2008 ▸).

The main output of the WTMD simulation is a free energy surface in the collective variables space, here characterized by the ϕ_1_ and ϕ_2_ torsional angles of an isopropyl group, see Fig. 5 ▸(*a*). To investigate the flexibility of the A and B groups independently, we set up two distinct simulations using the ϕ_1_ and ϕ_2_ angles of each TIPS group as the collective variables.

The broad free energy basins along ϕ_1_ suggest a dynamic disorder involving the rotation of the TIPS groups, which is more evident for the B TIPS group. The free energy surface shows that transitions from one of the three initial conformations to any other minimum exhibit energy barriers of at least 25 kJ mol^−1^. These transition barriers are several times *kT*, suggesting no dynamic disorder involving a conformational change is present. We can then calculate the equilibrium probability associated with each conformer to assess the presence of static disorder. While the six undistorted conformations dominate the probability distribution, three of them, one from the A and two from the B TIPS groups, display an approximate 10% probability of being distorted.

These simulations show the possible challenges in refining the two TIPS groups of the molecules as many concurrent phenomena are present at RT. These include the rotation of the TIPS group around the silicon atom and the presence of multiple isopropyl conformations.

The minor component of XIFZOF01 shows the B TIPS group rotating around 15° and one of the isopropyl chains in the A TIPS group being in a different conformation, corresponding to the most populated alternative conformation for that group [basin A2a in Fig. 5 ▸(*a*)].

Interestingly, XIFZOF shows a lower degree of disorder with only two isopropyl chains of the B TIPS group being displaced by around 15°. This could indicate the presence of dynamic disorder at higher temperatures (in the range where Form B becomes more stable) that converts to static disorder when the temperature is lowered.

The complex nature of disorder of the TIPS groups of XXVII indicated by the multiple crystal structure determinations and the extensive computational investigations has highlighted the handling of disorder in both theory and experiment as a major challenge to address in future research. For experimental determinations, disorder can heavily impact decisions made in the materials development process, whether that be in the pharmaceutical field or other areas of materials chemistry (Woollam *et al.*, 2018 ▸; Braun *et al.*, 2019 ▸), and this should be considered in future developments of CSP methods.

### 4.3. XXVIII

There is one known crystal structure of XXVIII (Form A, CSD refcode: OJIGOG01), with the molecule in a *trans* square-planar geometry. Unfortunately, the crystal structure of XXVIII was coincidentally published by an external group (Alshamrani *et al.*, 2021 ▸) during the test (CSD refcode: OJIGOG) and all participants were made aware of this by the organizers. It was decided to accept and analyse the results, though the exercise for this molecule cannot be considered a blind test.

Structural comparisons against the experimental Form A of XXVIII found five out of eight groups had correctly generated the known crystal structure among their submitted predicted structures (Groups 8, 10, 20, 24, and 25). Group 8 reported accessing the experimental structure where the experimental molecular conformation was used during the CSP workflow due to the CSD being utilized within their standard protocol. Group 20 disclosed that the experimental structure was utilized to continuously check it was present, but did not influence the CSP protocol. A range of geometries were considered beyond the *trans* square planar geometry observed in the experimental form (CSD refcode: OJIGOG01), with *cis* square planar, tetrahedral, and seesaw geometries also explored by some of the participating groups. Alterations to CSP workflows were also required in a small number of cases to allow for description of copper and the square planar conformation of the molecule.

### 4.4. XXIX

A single known crystal structure of XXIX exists (Form A, CSD refcode: FASMEV), containing three symmetry-independent molecules and crystallizing in the *P*2_1_/*c* space group. The experimental structure of Form A exhibits no signs of disorder. It is however composed of distinct layers, with alternating orientation of the molecules in the layers, suggesting a risk of stacking faults or polytypism. Polytypes are polymorphs where each form may be regarded as built up by stacking layers of (virtually) identical structure and composition, and where the forms differ only in their stacking sequence.

For the PXRD-assisted exercise for XXIX, simulated powder data were produced from the experimental single-crystal structure using *TOPAS* and were intentionally made to be of low quality. The simulated data were made accessible in the form of a PXRD plot (available in Fig. 1 of SI-A while the original pattern is available in Section 3 of SI-C) together with relevant metadata such as diffraction setup (transmission capillary), temperature (274 K), wavelength (Cu *K*α_1_, 1.54056 Å), and 2θ step size (0.017°).

The majority of groups who took part in this exercise converted the provided image of the PXRD pattern to a digitized file to allow for automated PXRD pattern comparisons. There was little range in methodologies employed for PXRD comparison. One group employed a PXRD fingerprint function approach.^1^ All other groups carrying out digital comparisons of PXRD patterns, including the successful prediction of Form A, employed some implementation of the FIDEL method, a highly successful approach for optimizing CSP-generated crystal structures by maximizing the agreement between simulated and observed PXRD patterns (Habermehl *et al.*, 2014 ▸). The FIDEL method relies on the calculation of a PXRD pattern similarity score using a cross-correlation function, which quantitatively evaluates the degree of congruence between the experimental and calculated patterns (de Gelder *et al.*, 2001 ▸). It is necessary to maximize the similarity by making small adjustments to the unit cell parameters. Optimizing only the unit cell is often sufficient, but molecular degrees of freedom may also be adjusted. Depending on the crystals’ morphology, and especially when the PXRD pattern has been measured in reflection geometry, it may be necessary to account for preferred orientation by, say, the March–Dollase model (Dollase, 1986 ▸). The combined or successive use of these techniques facilitates a robust and efficient optimization process, yielding high-quality crystal structures that closely resemble their experimental counterparts. One instance of this methodology is implemented in the AutoFIDEL script^2^ which was reportedly used by some of the groups for this exercise, in addition to the recently published variable-cell experimental powder difference (VC-xPWDF) method (Mayo *et al.*, 2023 ▸).

One group (Group 24) used MD simulations as the target PXRD pattern exhibited peak broadening to emulate experimental data collected close to the melting temperature.

The target crystal structure represented by the simulated PXRD pattern was successfully predicted by one group (Group 20), also ranking the structure as lowest in energy.

Of all submitted landscapes for this challenge, those of only seven of the 19 participating groups (Groups 1, 5, 10, 16, 20, 23 and 27) contained *Z*′ = 3 structures (Groups 1, 5 and 16 did not explicitly include *Z*′ = 3 in their search, see Table 19 in SI-A), which helps to explain the overall low rate of success in predicting the experimental form.

**Figure 6:**
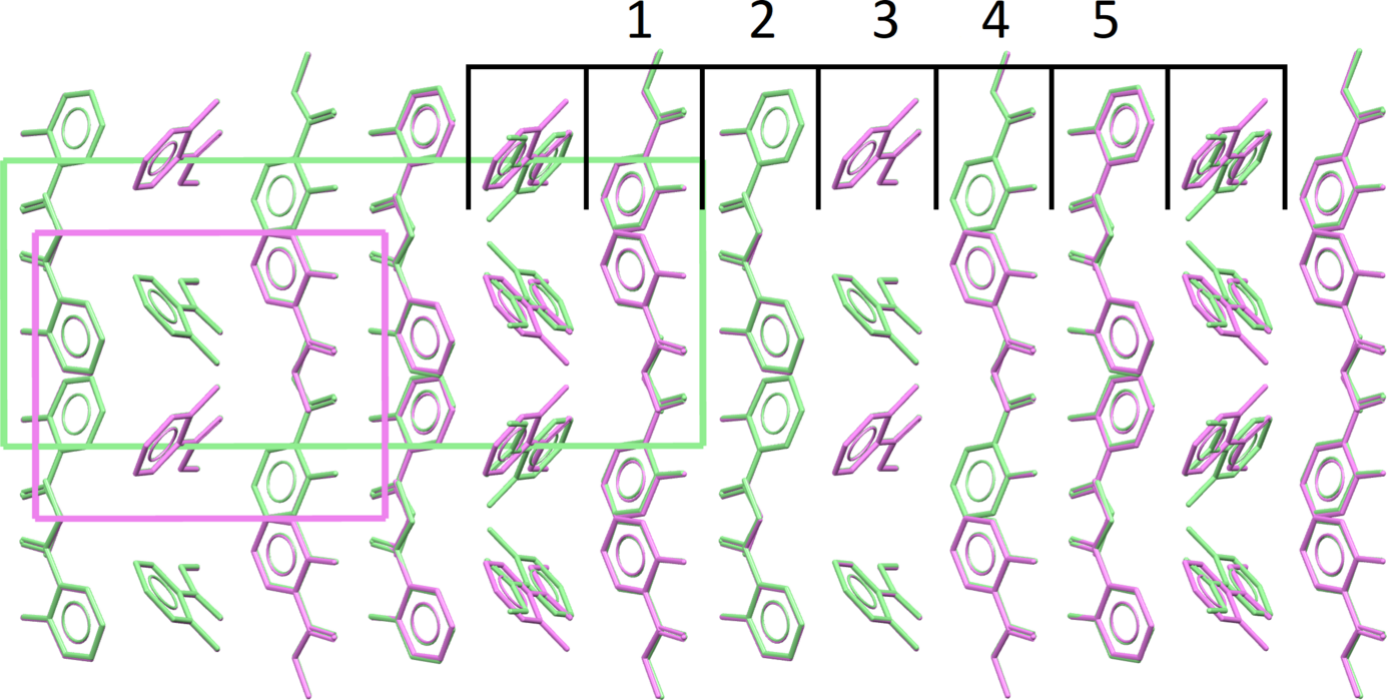
An overlay of the two polytypes of XXIX Form A; structures ranked first (light green, *P*2_1_/*c*) and second (violet, *Pc*) submitted by Group 20. Note how every sixth layer is oriented differently in the two polytypes. Hydrogen atoms are omitted for clarity.

Because of the layered structure of the target crystal, COMPACK comparisons demonstrated a large sensitivity to the number of molecules in the comparison cluster, which initially led to conflicting conclusions regarding the number of matching structures. Applying 35%/35° distance/angle tolerances, short-range structural matches were identified in submissions from nine groups (5, 6, 10, 11, 13, 16, 20, 21, 27) with a 20-molecule cluster (Tables 17 and 18 in SI-A), two groups (10 and 20) with a 30-molecule cluster, and only one group (20) with a molecular cluster of 70 and above. A visualization of the layered structure of the target Form A structure of XXIX, and unit cells of two polytypic variants are shown in Fig. 6 ▸. The unforeseen risk of polytypism may have led some groups to discard the correct structure because common clustering methods are not able to distinguish between polytypes (see individual groups’ reports in SI-B).

While one prediction (*Z*′ = 3, *Pc*) from Group 10 falls within the COMPACK matching criteria for this blind test, it is not a true structural match, but a structurally similar polytype of the experimental form, in which every 6th molecular layer is inverted (see Fig. 6 ▸). This polytype was also predicted by Group 20, in addition to the correct experimental crystal structure; the polytype was ranked as the second most stable structure and calculated to have a lattice energy within 0.1 kJ mol^−1^ of the experimental form.

**Figure 7:**
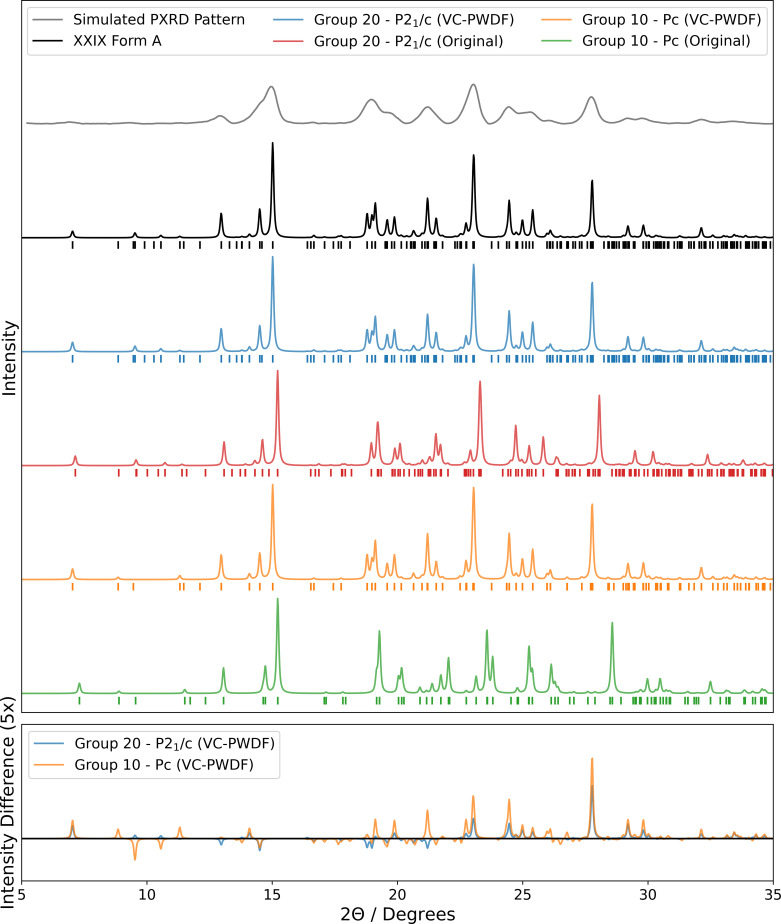
On top is the artificial target PXRD pattern given to participants, shown here without background profile. Second from the top is a pattern simulated from the experimental single-crystal X-ray diffraction (SCXRD) structure of Form A (*P*2_1_/*c*). The blue pattern, third from top, corresponds to the closest matching predicted structure after its lattice parameters have been adjusted with VC-PWDF. The red pattern, fourth from top, was simulated from the same crystal structure as found by CSP by Group 20. The bottom two patterns correspond to a polytype structure in space group *Pc* found by Group 10, as found by CSP (green), and after optimizing the PXRD similarity (orange). Note the subtle differences in Bragg peak positions and extinctions between the *Pc* and *P*2_1_/*c* structures. Inserted below is a PXRD intensity difference plot of the lattice-adjusted CSP structures relative to the SCXRD structure of Form A. The *y* axis has been scaled by a factor of 5 to aid the eye.

The target XXIX Form A and the polytype structure may be distinguished by PXRD. Comparisons between powder patterns of Form A, the noisy and artificially poor ‘experimental’ pattern provided to the participants, as well as the matching CSP structure from Group 20 are shown in Fig. 7 ▸. The ideal and noisy patterns of the experimental structure are compared to simulated powder patterns of the matching structure from Group 20, before and after the deformation of the lattice using the variable-cell powder difference method (VC-PWDF). The CSP structure nearly perfectly agrees with the powder pattern of the experimentally determined single-crystal structure. This demonstrates how CSP structures can greatly aid in indexing the poor quality powder pattern that has unusually broad peaks and substantial background noise, demonstrating its practical use in a common situation where the crystal structure is not known.

In the same Figure (Fig. 7 ▸), we show the same comparison of powder patterns for the polytypic structure predicted by Group 10. This structure, in space group *Pc*, has a powder pattern that is quite similar to the target, but it fails to correctly index the pattern. One can note the qualitative disagreement in Bragg positions (tick marks) between 10° and 11° 2θ.

The unanticipated complexity for structural comparisons in this case (in the context of both identifying structures matching experiment and clustering duplicates in a CSP workflow) may serve as warning to guide structure matching methods in future initiatives. An improvement to the selection of molecular clusters should be considered for the application of the COMPACK algorithm.

### 4.5. XXX

There exist two stable cocrystals of XXX: Form A (2:1 CBN:TMP, CSD refcode: MIVZEA) and Form B (1:1 CBN:TMP, CSD refcode: MIVZIE) (Mkrtchyan *et al.*, 2021 ▸). Form A exhibits disorder of the alkyl chain resulting in two components, Form A_maj_ and Form A_min_.

Presented as a stoichiometry prediction exercise, participants were asked to predict the two most likely stoichiometries to be observed and submit a list of 100 ranked structures in addition to the list of 1500.

A summary of the methods applied to predict stoichiometry and results from structural comparisons is provided in Table 5 ▸. Two groups (Groups 10 and 20) successfully generated Form A. Group 10 generated Form A_min_, ranked first at both 0 K and 298 K (this structure matched both disorder components under the matching criteria and was determined to match the minor component when visualized). Follow-up molecular dynamics investigations reported by Group 10 suggest that the disorder in Form A is likely dynamic, with both components of the disorder being part of the thermodynamic ensemble at RT, see Section 6 in SI-B. Independent investigation into dynamic disorder by the organizers was beyond the scope of this initiative. Group 20 generated both Form A_maj_ and Form A_min_, where the two individual structures were correctly identified as representing the major and minor components of a single disordered structure which was ranked second at 298 K (first when considering structures with 2:1 stoichiometry only). This is the first blind test where a CSP method has generated a disordered structure represented by a single Crystallographic Information File (CIF).

Structural comparisons of Form B with the submitted landscapes using COMPACK identified matches with structures from three participants: Groups 5, 10, and 20. Two of the three groups also provided Form B in their smaller ranked lists. Group 10 found the experimental structure as ranks 11 and 9 at 0 K and 298 K, including thermal contributions, respectively (ranked second at both 0 K and 298 K amongst 1:1 stoichiometry structures only). Group 20 found the experimental structure as rank 5 at 298 K (rank 2 within 1:1 stoichiometry structures only), having also accounted for thermal contributions.

The majority of ranking methods applied in this cocrystal stoichiometry challenge employed the method based on the sum of calculated energies for pure components (Cruz-Cabeza *et al.*, 2008 ▸). Additional methods included one based on a thermodynamic cycle, and the construction of convex hulls (Sun *et al.*, 2020 ▸; Hildebrandt & Glasser, 1994 ▸), which were applied by Groups 10 and 20 respectively, who successfully predicted both forms and ranked both at relatively low energy.

Seven of the 13 groups (Groups 5, 10, 19, 20, 21, 27 and 28) correctly predicted the two stoichiometries observed experimentally. The majority of groups based their prediction on the calculated ranking or energies of low-energy CSP structures. However, two groups, 19 and 28, predicted the correct stoichiometry purely based on the ratio of hydrogen bonding donors and acceptors in the two molecules.

**Figure 8:**
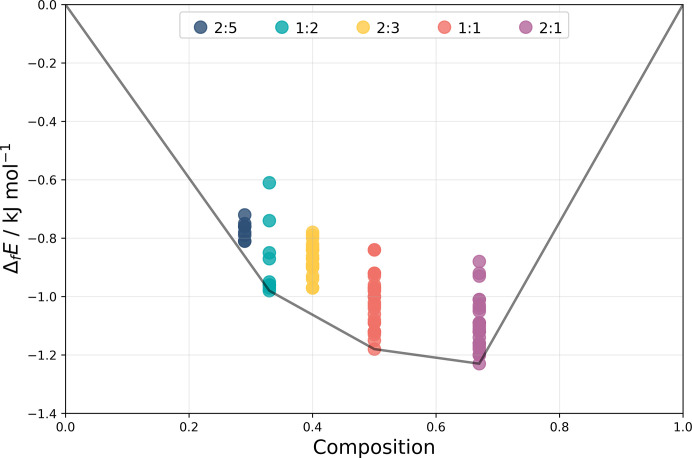
Convex hull (grey line) of the free energy of formation, approximated as the PBE-D3 lattice energy, Δ_f_*E*, of cannabinol tetramethylpyrazine cocrystals as a function of their composition. The data was provided by Group 22. Each data point corresponds to a distinct predicted cocrystal structure. Note that structures of three different stoichiometries lie on the convex hull.

Group 22 argued that a compound *A*_*x*_*B*_*y*_, where *A* and *B* may be atoms in an ordinary compound or whole molecules in a cocrystal, is thermodynamically stable if and only if its Gibbs free energy, *G*, is lower than that of any isochemical assemblage of phases. This criterion is conveniently represented graphically if one plots, as in Fig. 8 ▸, the normalized free energy of formation Δ_f_*G*(*A*_*x*_*B*_*y*_) of all possible compounds *A*_*x*_*B*_*y*_ as a function of the composition *y*/(*x* + *y*): 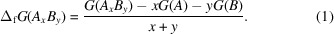
Stable structures form a convex hull. This means negative energies of all imaginable reactions of their formation from any other substances in the *A*–*B* system. Based on the convex hull method, and using DFT-D lattice energies as approximation for the true free energies, Group 22 predicted that the following three stoichiometries are stable in the cannabinol:tetramethylpyrazine system: 1:2, 1:1, 2:1. That is, they correctly predicted both of the observed stoichiometries and predicted that there should exist an additional cocrystal stoichiometry that has not yet been seen experimentally.

### 4.6. XXXI

For compound XXXI, three different forms are known (Forms A–C) where Form A exhibits disorder via the rotation of the *ortho*-fluorophenyl ring (Form A_maj_ and Form A_min_) and Form C is a porous desolvate.

Eight groups (1, 3, 5, 10, 16, 19, 20, 24) successfully generated both Form A_maj_ and Form A_min_, see the results summarized in Table 6 ▸. An additional two groups (18, 26) generated just the minor disorder component. Nine groups (1, 3, 5, 6, 10, 16, 19, 20, 24) successfully generated Form B. No structures were identified to match Form C, though considering the solvent-stabilized nature of the crystal form and that no experimental conditions or possible solvents were provided to participants to indicate this as a possibility, this was expected.

Relatively high success was observed for XXXI with eight groups (1, 3, 5, 10, 16, 19, 20, 24) successfully generating all three of Form A_maj_, Form A_min_, and Form B.

### 4.7. XXXII

There exist two known crystal structures of XXXII; Form A (*Z*′ = 1, CSD refcode: JEKVII) and Form B (*Z*′ = 2, CSD refcode: JEKVII01), both determined at low temperature (90 K). Form A exhibits disorder of the difluoromethyl group resulting in two components, Form A_maj_ and Form A_min_.

During the test, an additional crystal structure of Form B was determined from PXRD at RT, a *Z*′ = 1 structure in space group *P*2_1_/*c* (provided in SI-D), which suggested a structural difference to the 90 K form (a *Z*′ = 2 structure in space group 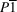). However, comparisons of this RT structure to predictions resulted in no matches. The subsequent structure ranking exercise (see Hunnisett *et al.*, 2024 ▸), requiring participating groups to apply their own local optimization methods, produced geometry-optimized structures that no longer resembled the starting structure derived from the PXRD pattern. The PXRD data was provided to all participants following the end of the initiative and a redetermination of the structure was proposed by Group 20 (provided in SI-D). Solid-state nuclear magnetic resonance (NMR) shielding calculations carried out by Antonio DiPasquale then confirmed that the redetermined crystal structure from CSP provided a better fit to experimental ^13^C and ^1^H NMR data than that previously derived from PXRD.

**Figure 9:**
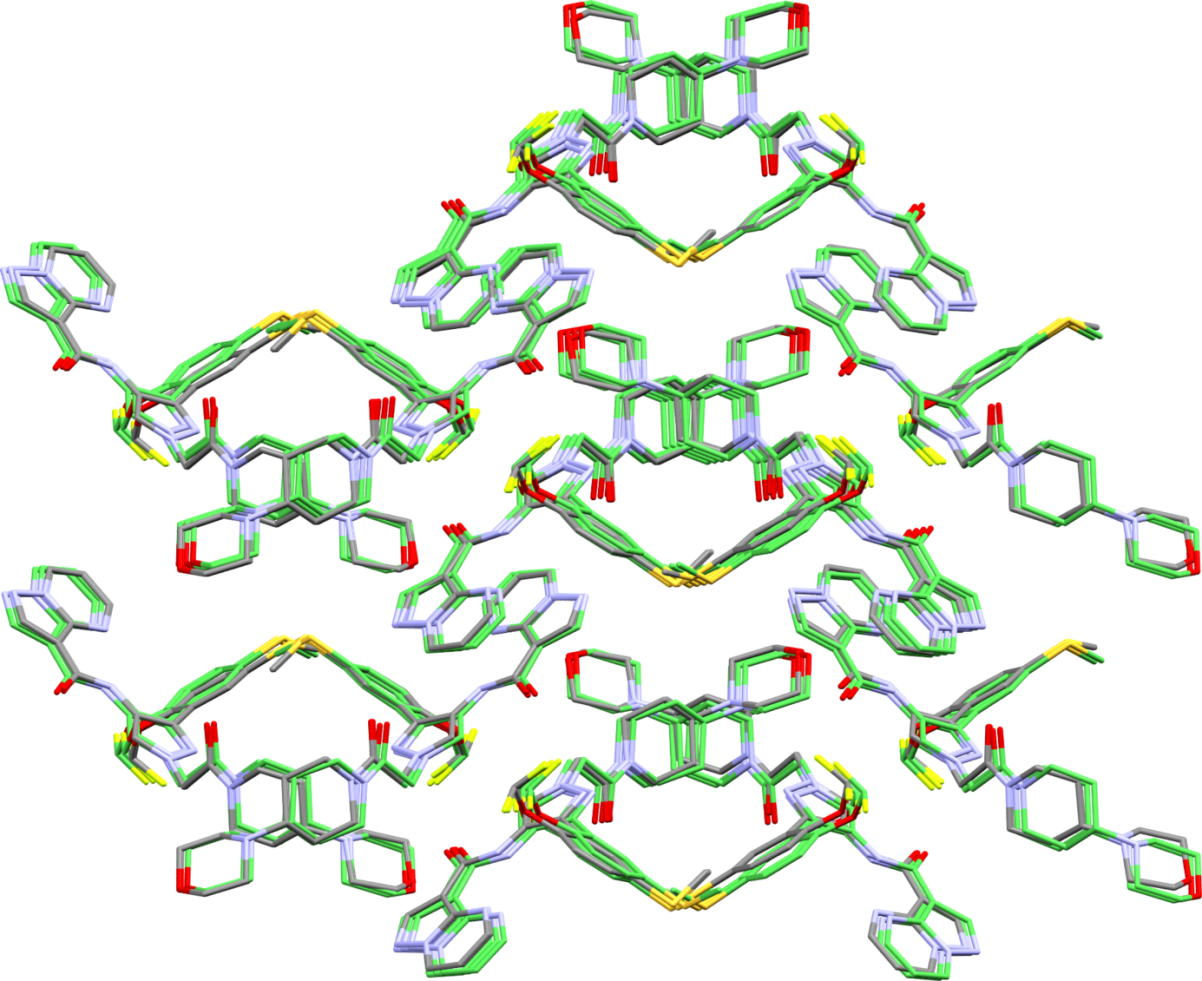
COMPACK overlay of XXXII Form B at 90 K (coloured by element) with the redetermination from PXRD of XXXII Form B (ambient temperature) by Group 20 (coloured green). Hydrogen atoms were omitted for clarity.

Further COMPACK comparisons of Form B at LT and the redetermined Form B at RT with predicted structures were unable to identify distinct matches to each form, instead resulting in matches to both forms in many cases. This is due to the minor difference between the two geometries resulting from a conformational change of the terminal thiomethyl group, see Fig. 9 ▸. Attempts were also made to identify matches to each form via manual visualization, though this also proved difficult due to there being no certain matches in each case. Results in Table 6 ▸ refer to Form B at LT only, although many of these structures were also found to match Form B at RT. Investigations into whether the LT and RT structures of Form B should be considered the same or not were beyond the scope of this blind test.

COMPACK comparisons of predicted structures with Form A identified matching structures to the major disorder component from two groups (10 and 20), with an additional possible match from Group 25, although with a high RMSD of 1.03 Å. Groups 10 and 20 were also successful in finding Form B but no predicted structures were found to match the minor disorder component of Form A.

Molecule XXXII provided a complex challenge to CSP due to the high flexibility within the molecule, though only containing one hydrogen-bond donor. Furthermore, Form B has *Z*′ = 2, posing a computationally demanding challenge, particularly for academic groups who may have limited resources and expertise to carry out the calculations. Of the 13 groups who participated, only seven extended their structural search to *Z*′ > 1, explaining why many groups did not predict Form B.

### 4.8. XXXIII

Target XXXIII was found to exist in two polymorphic forms: Form A (CSD refcode: ZEGWAN, a disappearing polymorph), and Form B (CSD refcode: ZEGWAN01).

Of the 14 participating groups, five groups (5, 10, 20, 21, 24) successfully predicted Form A, four of which (5, 10, 20, 24) also predicted Form B. No matching predicted structures were identified in the remaining groups.

### 4.9. Crystal structure landscape similarity

The convergence of structure generation methods to the same set of low-energy structures is an indication of the improvements made in crystal structure prediction. The previous attempts at structure similarity searches between different CSP sets discussed earlier for the two rigid molecules from the third blind test, hydantoin (VIII) and azetidine (XI), and ROY (van Eijck, 2005 ▸; Nyman *et al.*, 2019 ▸), reached the worrying conclusion that CSP methods largely do not yield the same structures.

To assess search completeness, we (the organizers) performed a purely geometrical crystal structure similarity comparison between the submitted structure sets, fully aware of the limitations of this approach. Different crystal structures may correspond to the same lattice energy minimum (van Eijck, 2005 ▸), and it can therefore be argued that it may be preferable to geometry-optimize all structures with a common energy method before comparisons. However, it was of interest whether different approaches yield the *same structures* or not; addressing the alternative question of whether they find the *same basins of attraction* or not would have required the re-optimization of all structures with some energy-method widely regarded as reliable, such as dispersion-corrected DFT, a prohibitively costly approach for an analysis involving tens of thousands of structures.

This similarity search aimed at evaluating whether the different groups proposed the same structures as potential observable polymorphs. It is important to note that the same structure generation method can produce different results depending on the search constraints such as available space groups, molecular conformations considered, or maximum *Z*′ used. The introduction of thermal effects and the evaluation of surface rugosity and crystallizability can further impact which structures have been submitted.

In this study, we conducted two set comparisons: one involving the first 100-ranked structures from each group, and the second comparing the first 100-ranked structures from one group with the entire set of the other (and *vice versa*), labelled as *100 versus 100* and *100 versus all*, respectively. The latter aimed at verifying if low-energy structures obtained with one method are present among the extended set of another. This approach helps reduce the impact of the energy evaluation method used as the accurate ranking was not necessary in this phase but rather the focus of the second blind test paper. Although it was not mandatory, all participants submitted the energy and rank of the generated structures and allowed us to make these comparisons. It should then be noted that the level of accuracy of the rankings may vary from group to group.

The large number of structures necessitated the use of computationally efficient algorithms for the assessment of structure similarity. To this end, we used the approach described by Widdowson *et al.* (2022 ▸), which makes use of pointwise distance distributions (PDD) as descriptors for each crystal structure. This consists of an *N* × *k* weighted matrix in which each row corresponds to an ordered list of distances between an atom in the unit cell to the *k* closest neighbours. Identical rows are then collapsed together with weights assigned based on the number of occurrences. Similar to the COMPACK algorithm, the use of atom-atom distances makes the comparison independent of the choice of the unit cell. These descriptors can then be compared with the Earth Mover’s Distance (Rubner *et al.*, 1998 ▸; Widdowson & Kurlin, 2022 ▸).

Pointwise distance descriptors were initially tested in the assessment of similarity between theoretical and experimental structures and contributed to the late identification of target XXVII matches. Section 5.2 in SI-A provides a detailed comparison between PDD and COMPACK results. When comparing two structures, an isotropic expansion of the reference structure based on their volume ratio was applied to limit thermal effects. An overestimation of similarity was observed between structures of molecule XXXI. This was due to the lack of chemical information in the PDD metric, resulting in assessing those structures that share the same packing but have molecules in different conformations (with the fluorobenzene rotated at 180°), as similar.

**Figure 10:**
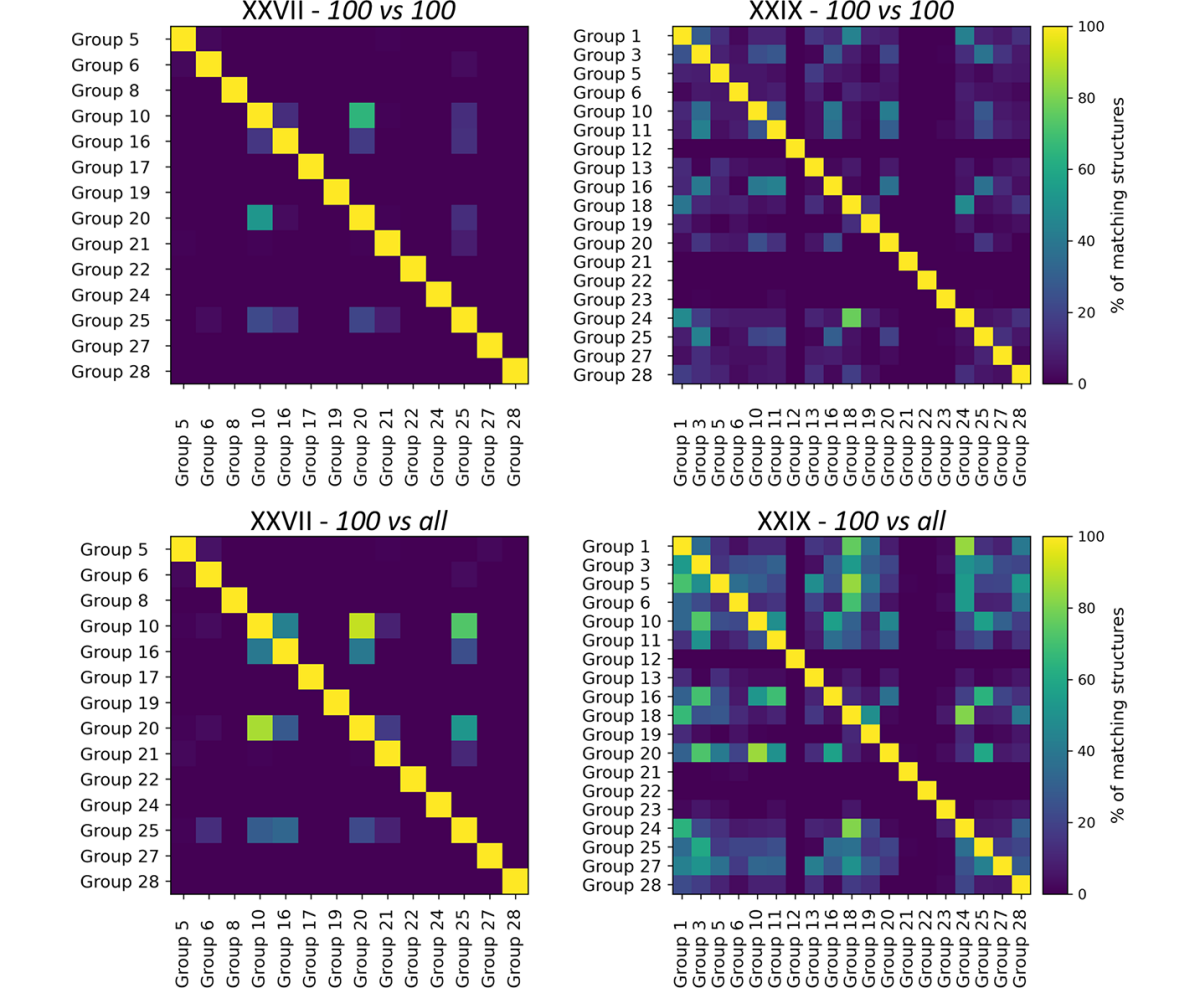
Crystal structure set similarity heat maps for molecules XXVII and XXIX showing the percentage of structures from the group on the horizontal axis that match a structure from the group on the vertical axis. Top plots show the *100 versus 100* comparisons, while those at the bottom the *100 versus all* ones. Some groups have to a large extent predicted the same crystal structures. The comparisons are not symmetric because multiple structures in one set can match a single structure from another one; this is possibly due to stricter clustering criteria.

Using *k* = 100, all matches with experimental crystals (according to the structure similarity criteria defined in Section 2.7) were found to be below 0.375 Å. Despite this, for the assessment of similarity between sets we used a much stricter cutoff of 0.225 Å to reduce the impact of false positives, exclude poorly overlapping structures and balance the missed perfect matches with the inclusion of a few partial matches (see Fig. 8 in SI-A). The comparison of structures results in a heat map which shows the percentage of structures from each group that are present in the sets by every other group. Two examples of such heat maps are shown in Fig. 10 ▸, while the remainder are available in the supplementary information (Figs. 9 and 10 in SI-A).

It is important to note that the heat maps are in general not symmetric, especially the *100 versus 100* comparisons. Although in a few cases this is due to different set sizes (as some groups have submitted less than 100 structures), this asymmetry is a consequence of the different clustering approaches adopted by each group. As a result, within the PDD distance cutoff considered, multiple structures from one group can match a single structure from another group. In general, loose clustering criteria allow for the sampling of a wider range of diverse crystal packings within the landscape. Once a subset of promising structures has been selected, closely related packings can then be retrieved by further analysis. For example, MD simulations on molecule XXVII, starting from a single crystal, were able to show a variety of possible structures which share the same packing of the pentacenes but have different conformation of the TIPS groups. In contrast, strict clustering criteria ensure that no relevant structure is being removed. This may have been crucial in the study of molecule XXIX where different structures having multiple layers in common could have been dismissed as duplicates.

Encouraging results were derived from our analysis, with some groups sharing a large proportion of their structures. Target systems XXIX and XXXI, both small molecules with few conformations available, show substantial overlap between certain groups; an example of target XXIX is shown in Fig. 10 ▸. Whilst some of the similarity could be explained by the use of the same software (for example *CrystalPredictor II* for Groups 1, 3, 18 and 24), substantial landscape overlaps also came from groups that used widely different structure generation and energy ranking methods.

As the size and flexibility of the molecule increase, the CSP sets become increasingly different, as shown in Fig. 10 ▸ for target XXVII. Low overlap is observed also in targets XXVIII and XXXIII, where challenges arise from the modelling of metal-containing molecular systems and the presence of two different molecules in the asymmetric unit. While it is not surprising that the generated structures diverge with increasing system complexity, a promising outcome is a good agreement between Groups 10 and 20 throughout the compounds. These two groups used similar methods in generating the structures with the assistance of machine learning approaches in the selection of structures on which to run dispersion-corrected DFT calculations. On average, 40% of the structures match in the *100 versus 100* comparison and 75% in the *100 versus all*.

### 4.10. Resource utilization

The sixth blind test involved an enormous expenditure of computational resources, time and money for some groups, continuing a trend established in previous tests (Reilly *et al.*, 2016 ▸). In an effort to understand the computational efficiency of the CSP methods applied in this seventh initiative, the number of CPU core hours and the hardware used were required to be reported alongside all predictions and are summarized in Table 7 ▸. It is important to note that the numbers reported here are not normalized with respect to the wide range of computational hardware utilized so should not be directly compared across groups, and challenges arising due to the high topological symmetry of XXVII may have also skewed the resources spent for some groups. Future initiatives should perhaps compare the energy expenditure in units of kWh instead.

With more than 46 million CPU core hours reportedly utilized for the structure generation phase of this seventh blind test alone, we cannot avoid commenting on the need for the community to carefully consider the economic and environmental impact of CSP. Scientific research, and possible future blind tests, should better allow for the ethical use of natural, computational and economic resources and focus on developing rational and efficient algorithms for CSP, rather than naïve brute force methods.

## 5. The seventh CSP blind test meeting

A two-day in-person meeting was held in Cambridge, UK following the final results submissions in September 2022 ▸. This provided the opportunity for participants to present their results to fellow investigators, blind test organizers, and active researchers in the CSP community from both industry and academia. A session was also held between participants and organizers to discuss any issues arising during the test and reflect on the current and possible future blind test initiative.

The comparison of crystal structures and the determination of whether two structures are the same or not can be sensitive to the method applied. The ambiguous nature of crystal structure similarity measures was raised by both organizers and participants as a significant challenge for the seventh test. It was agreed from discussions that tolerances used in COMPACK matching criteria should be looser for this phase of the test in line with recent findings (Sacchi *et al.*, 2020 ▸; Mayo *et al.*, 2022 ▸). In previous tests, these were relatively tight, which may have led to missed matches. Two missed matches from the sixth blind test, arising from the choice of COMPACK settings, are reported by Mayo & Johnson (2021 ▸). The consideration of alternative comparison methods was raised and agreed as a valuable exercise. In addition, the organizers proposed to provide greater detail on comparison results such as RMSD and applying a range of tolerances with the COMPACK method to provide a better understanding of a close or tentative match to experiment.

Ideas were proposed by participants to implement in future blind test initiatives with the focus on the assessment of structural similarity and bringing more industrial relevance to the exercises set. The use of experimental PXRD data to assess structural similarity was discussed, though the sensitivity to temperature and crystallographic disorder was highlighted and would require careful consideration on a case-by-case basis. On the other hand, this would provide clarity by accounting for cell size variation in comparisons. The use of additional experimental data in the initiative such as solid-state NMR would also help realize the industrial applications of CSP. Alternatively, incorporating the use of geometry optimization methods into the comparison assessment could help to determine whether a predicted and experimental structure represent the same basin of attraction, though this would require an enormous amount of resources, and the question of which method to apply here remains to be answered.

On reflection of the development and applications of CSP, discussions between organizers and participants raised a number of questions that remain to be answered by future research. One prominent issue that still remains is overprediction, and whether CSP has made progress towards predicting which of the hypothetical structures are experimentally accessible polymorphs. The question of how CSP is currently being applied in industry was raised, with a better connection desired between methods developers and end-users. This is difficult because proprietary CSP results obtained by pharmaceutical companies are rarely published. An understanding of how the current costs and time consumed by CSP methods compare with the experimental time needed to reach conclusions within industrial cases would be useful to guide future CSP developments.

## 6. Conclusions

The seventh blind test as a whole involved the largest number of participating groups to date with 150 researchers from 28 unique groups spanning 14 countries, and significant contributions from 18 experimentalists performing chemical synthesis, crystal structure determinations, and solid-form screening. This reflects the enormous interest and application, particularly in recent years, of CSP in academia and in industry.

The range of methods demonstrates the significant advances made in recent years, with machine learning approaches becoming more prominent, and wider adoption of quantum chemical calculations earlier in the CSP workflow. The successful CSP methods utilized in this initiative demonstrate that the accurate prediction of crystal structures requires consideration of intricate details demanding large amounts of resources and dedicated researchers, favouring commercial CSP providers or collaborations between academia and industry over purely academic researchers. Of notable achievement, Group 20 generated correct structures for all target compounds, and Group 10 generated correct structures for all except target XXIX (where a near structural match highlighted the importance of structural comparison standards). This great success can be attributed to the use of highly reliable quantum chemical calculations, cloud computing, machine learning techniques, tailor-made force fields, careful accounting of thermal effects, and efficient conformational sampling algorithms, which enabled them to effectively explore the vast configurational space and identify the most stable structures.

The two-phase format of the test has allowed the analysis and benchmarking of structure generation and ranking methods separately. This test of structure generation has provided a clearer understanding of the search space covered by each CSP method, prior to refined ranking and filtering of the landscape. In general, the overlap between structure sets generated by most CSP methods is still strikingly small. The limited success by several participants in generating the experimental structures also shows that CSP is indeed a great challenge.

In an exercise designed to push the boundaries of CSP capabilities, one group successfully determined a crystal structure represented by a low-quality PXRD pattern, a circumstance often encountered in solid-form experimental investigations. The inclusion of new chemistry in the form of compounds with copper or silicon has challenged CSP practitioners to extend their capabilities, and resulted in successful predictions of non-pharmaceutical systems.

The question of whether two crystal structures should be considered the same or not remains a challenging one with no straightforward answer. There is a need for a general standardized practice for classifying matching crystal structures within the crystallographic community. This would inform the development of structural comparison methods and structure match criteria in CSP workflows, which in this blind test likely led to lower success rates for targets XXVII, XXIX (Group 10, see Section 6 of SI-B) and XXX.

The presence and characterization of crystallographic disorder emerged as a significant challenge in the seventh CSP blind test, complicating both the prediction process and the subsequent analysis of the results. Despite the complexity, a significant milestone for CSP has been reached in this test with the first true blind prediction of disorder by Groups 20 and 24, applying methods based on symmetry-adapted ensembles on target XXX, and molecular dynamics on target XXVII, respectively. Disorder in crystal structures arises from the presence of multiple distinct conformations, orientations, or positions of atoms within the unit cell. The inherent complexity of disordered systems poses a formidable obstacle for the participating methods, as it demands a more sophisticated approach to conformational sampling and requires the consideration of multiple plausible structural candidates. Additionally, the presence of disorder can hinder the unambiguous evaluation of the predicted structures against experimental data, as it introduces an element of uncertainty in the determination of the correct crystal structure. Consequently, predicting and modelling of crystallographic disorder will be crucial for further advancements in the field of crystal structure prediction, necessitating the use of methods such as molecular dynamics or symmetry-adapted ensembles, capable of effectively handling the multifaceted nature of disordered systems and providing predictions that more accurately agree with dynamically disordered structures at crystallization, process and storage conditions.

The use of enormous computational resources in this initiative has shown that ethical considerations and a focus on the development of more computationally efficient algorithms should shape any future blind test initiatives.

The outcomes of the seventh CSP blind test emphasize the importance of continued innovation and collaboration in the field of crystal structure prediction; openly available data, published methods and open source software are key drivers to maintain and improve innovation in this thriving research community. The overall success of Groups 10 and 20 showcases the potential of current methods to accurately predict molecular crystal structures, and it serves as an inspiration for the development of more advanced and robust techniques. As the field moves forward, it will be crucial to build upon these successes and address the remaining challenges in order to fully unlock the predictive power of CSP methods for a wide range of applications in materials science, pharmaceuticals, and beyond.

## 7. Glossary

**API** Application programming interface

**B86bPBE** A GGA density functional consisting of the exchange functional proposed by Becke in 1986 and the PBE correlation functional

**CBN** Cannabinol

**CCDC** The Cambridge Crystallographic Data Centre

**CIF** Crystallographic Information File, a standardized file format for crystallographic data

**COMPACK** An algorithm for calculating crystal structure similarity based on atomic distances

**CPU** Central processing unit

**CSD** The Cambridge Structural Database

**CSP** Crystal structure prediction

**D3** Grimme’s dispersion correction, version three

**DFT** Density functional theory

**DFT-D** Dispersion-corrected density functional theory

**DFTB** Density functional tight binding

**FF** Force field, a specific set of equations and parameters for calculating interaction energies

**FIDEL** A method for matching crystal structures to PXRD patterns

**GAFF** Generalized Amber Force Field

**MD** Molecular Dynamics, a simulation method

**MMFF94s** The static force field developed by Merck

**MP2** Second-order Møller–Plesset perturbation theory

**NMR** Nuclear magnetic resonance spectroscopy

**PBE** The exchange-correlation functional by Perdew, Burke and Ernzerhof

**PBE0** A hybrid exchange-correlation functional, PBE with 25% Hartree–Fock exchange

**PXRD** Powder X-ray diffraction

**RMSD** Root-mean-square deviation

**ROY** The 5-methyl-2-[(2-nitro-phenyl)amino]-3-thiophenecarbonitrile compound

**RT** Room temperature

**SAPT** Symmetry adapted perturbation theory

**SI** Supplementary information

**TIPS** Triisopropylsilane, a functional group

**TMP** Tetramethylpyrazine

**VC-PWDF** A method for matching crystal structures by PXRD pattern similarity

**XDM** The exchange-hole dipole moment dispersion correction

## Supplementary Material

SI-A: Additional information, tables and figures. DOI: 10.1107/S2052520624007492/aw5093sup1.pdf

SI-B: Method description and further analysis per participant group. DOI: 10.1107/S2052520624007492/aw5093sup2.pdf

SI-C: Experimental reports. DOI: 10.1107/S2052520624007492/aw5093sup3.pdf

SI-D: Theoretical and experimental structures. DOI: 10.1107/S2052520624007492/aw5093sup4.zip

## Figures and Tables

**Table 1:** The information provided to participants at the start of the seventh blind test Two-dimensional chemical structures of the seven target systems, any additional information, and the data requested by the organizers to be submitted by participants. Experimental groups for both crystal structure determinations and solid-form screen experiments are also noted.

Target	Chemical diagram	Additional information	Requested predictions	Experimental groups (crystal form determination / solid-form screen experiments)
XXVII	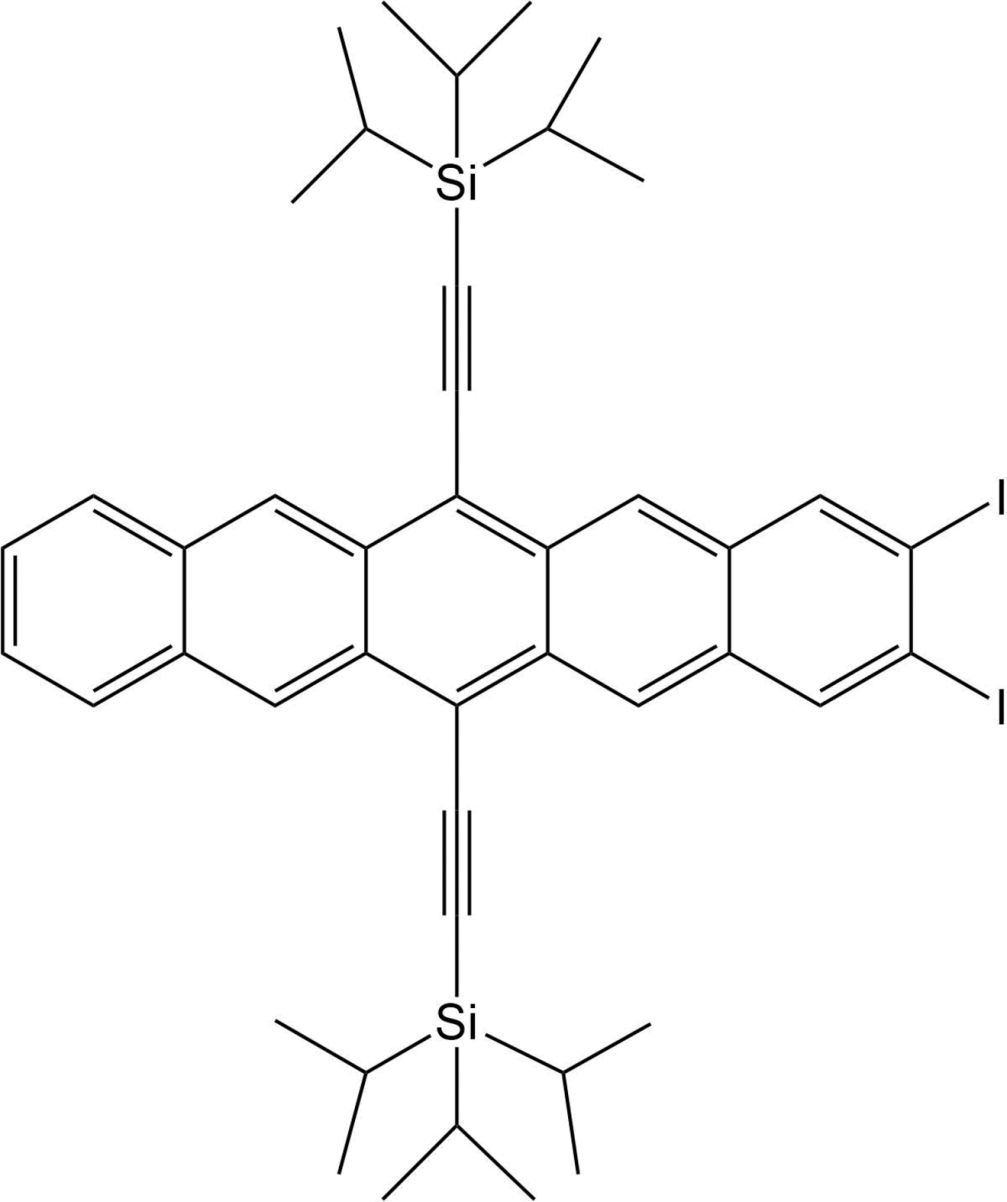	None	1500 putative structures	J. A. Anthony, S. Parkin / F. Tarczynski, J. Bis, S. Carino
XXVIII	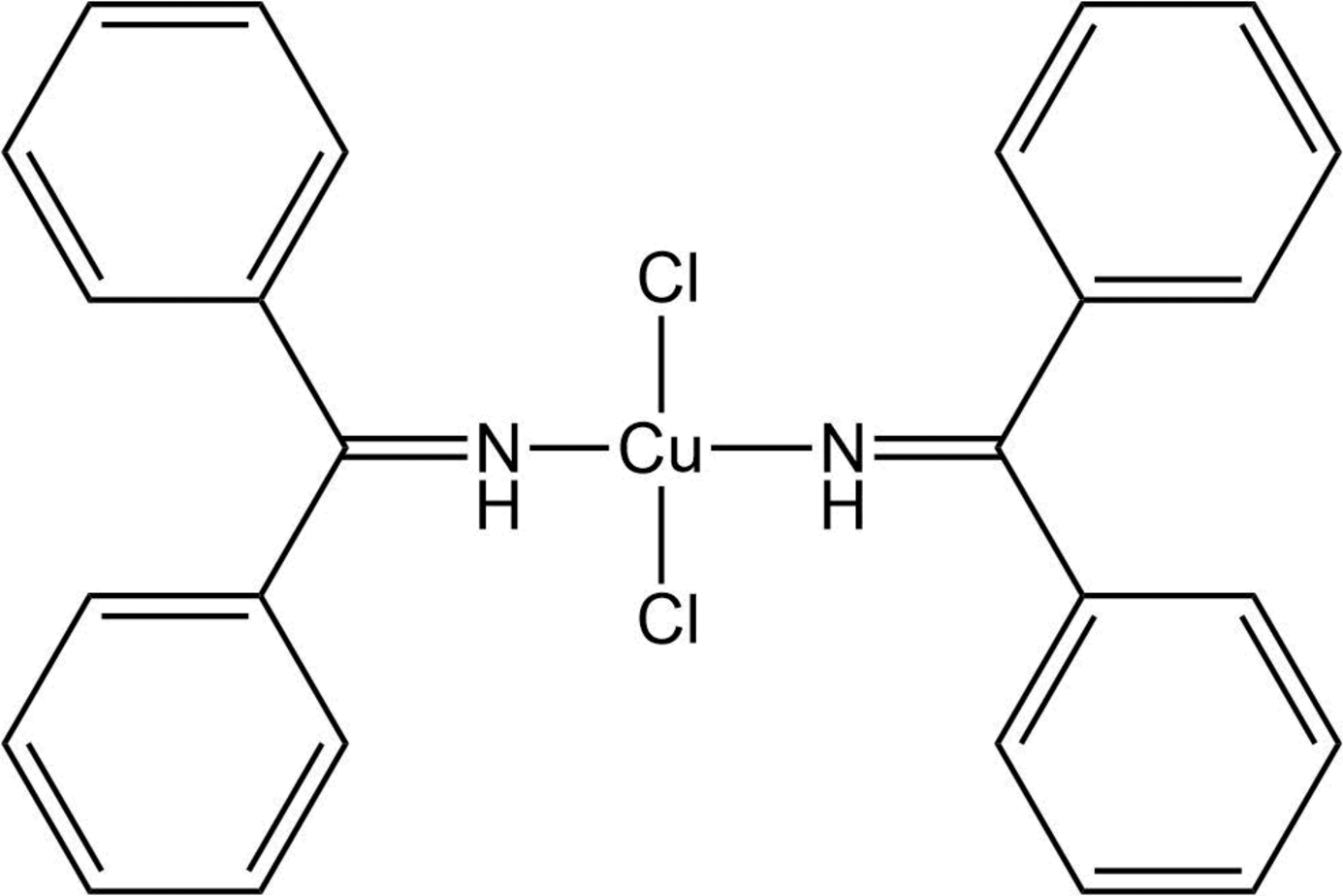	None	1500 putative structures	M. R. J. Elsegood, P. F. Kelly, L. Wilkinson / M. R. Probert, vJ. Weatherston
XXIX	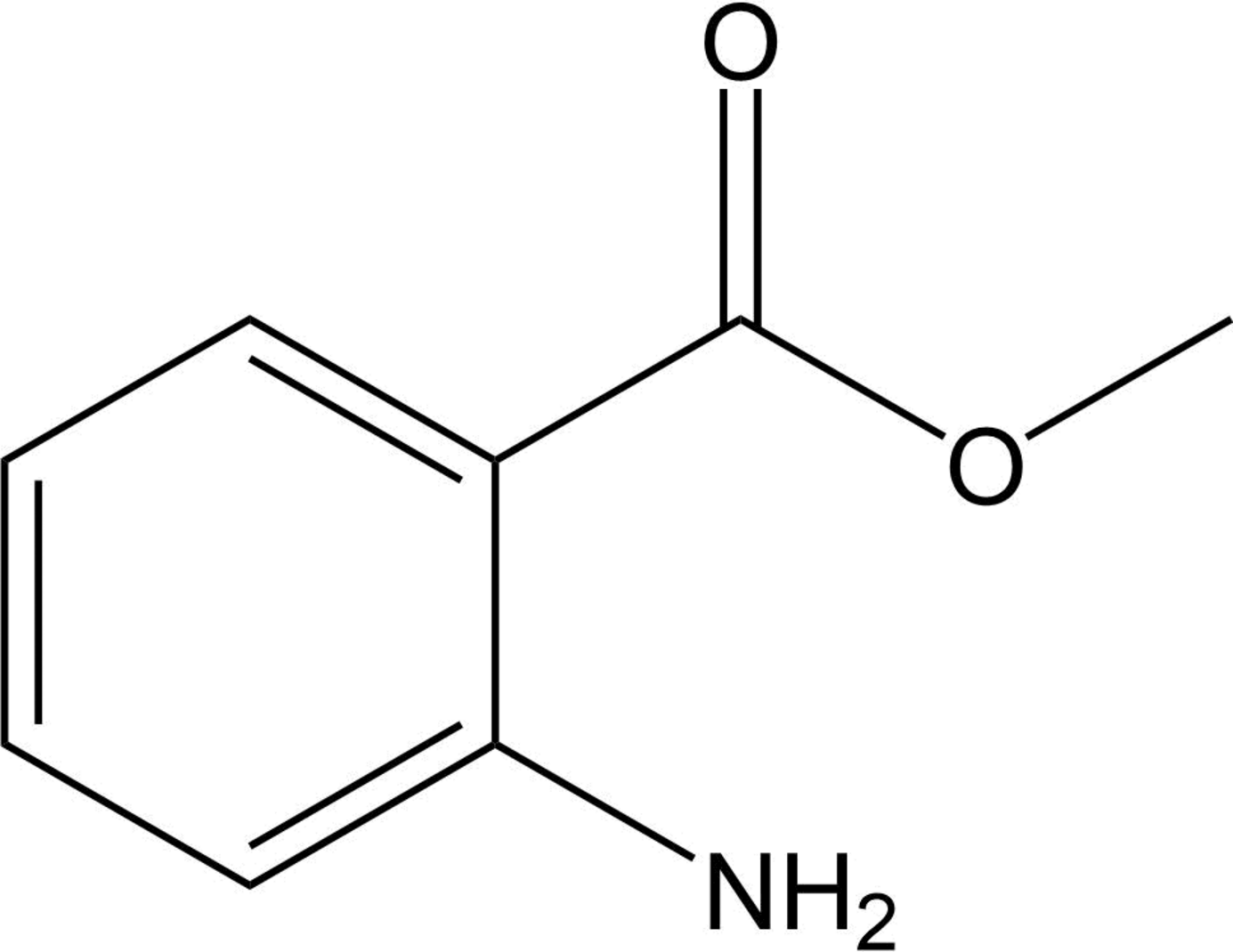	Simulated PXRD pattern	1500 putative structures, ten ranked structures matched to PXRD pattern	K. Shankland, E. Kabova, M. Ross / M. R. Probert, J. Weatherston
XXX	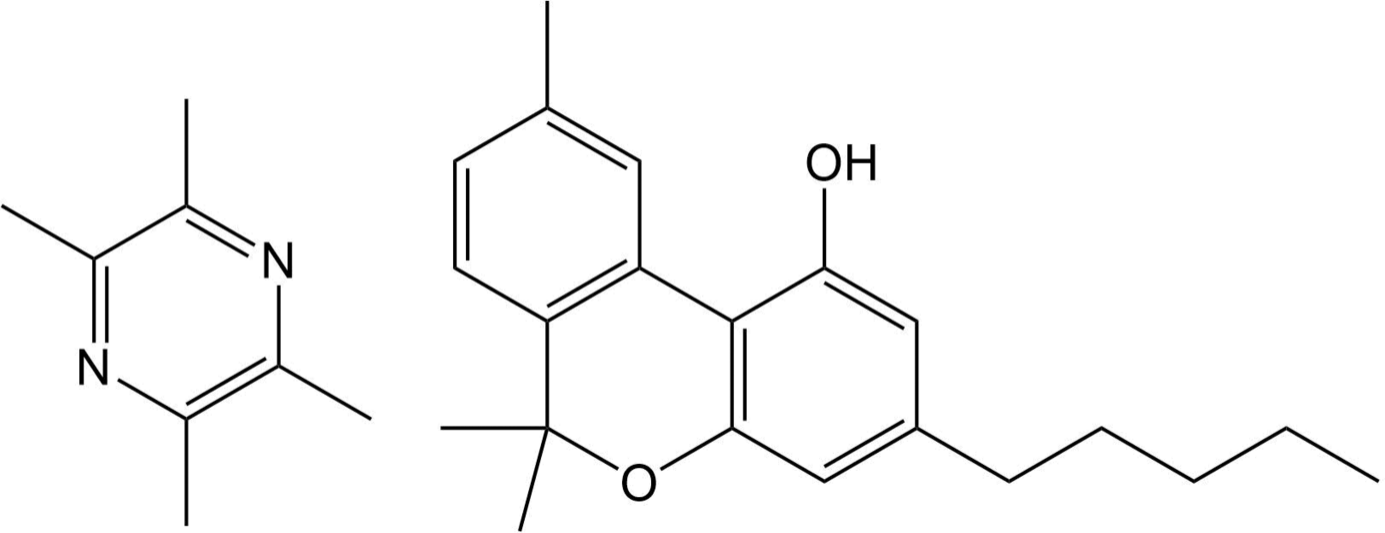	There are two forms of different stoichiometries (cannabinol:tetramethylpyrazine is two of 1:1, 2:1 or 1:2)	1500 putative structures, 100 ranked structures, and a prediction of stoichiometries	J. A. Bis, S. Carino, R. Couch, L. Wojtas
XXXI	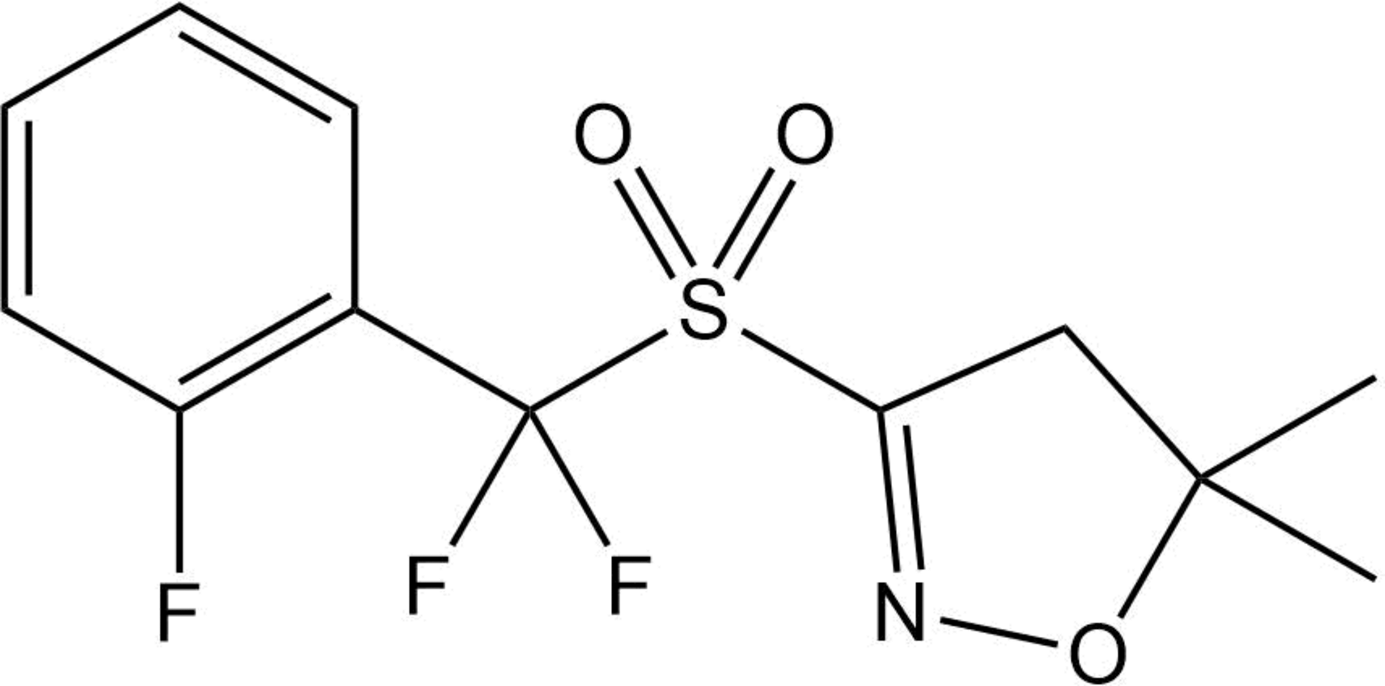	Two known polymorphs	1500 putative structures	J. Hone, A. Keates, I. Jones
XXXII	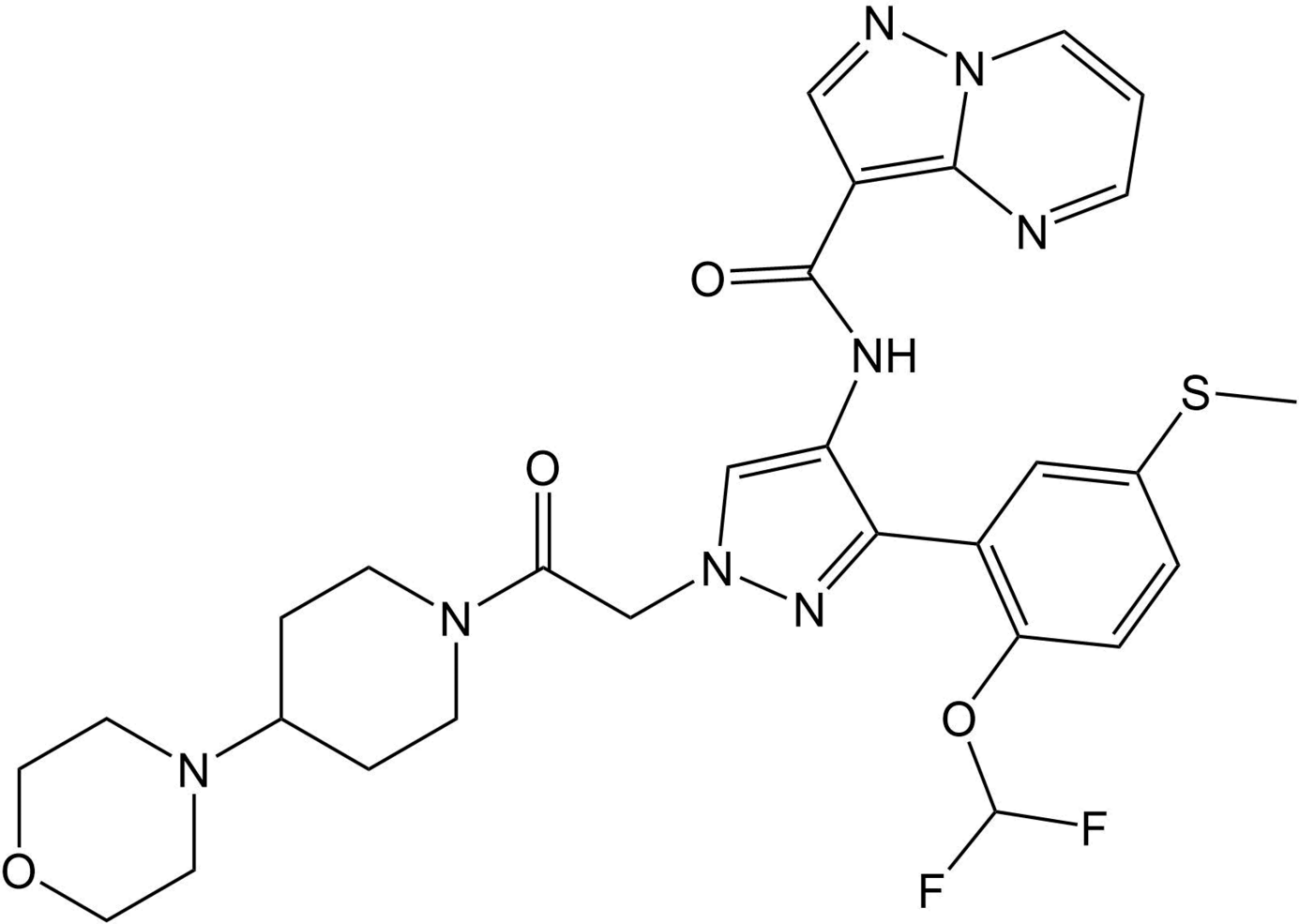	Eight known polymorphs	1500 putative structures	A. DiPasquale, J. W. Lubach
XXXIII	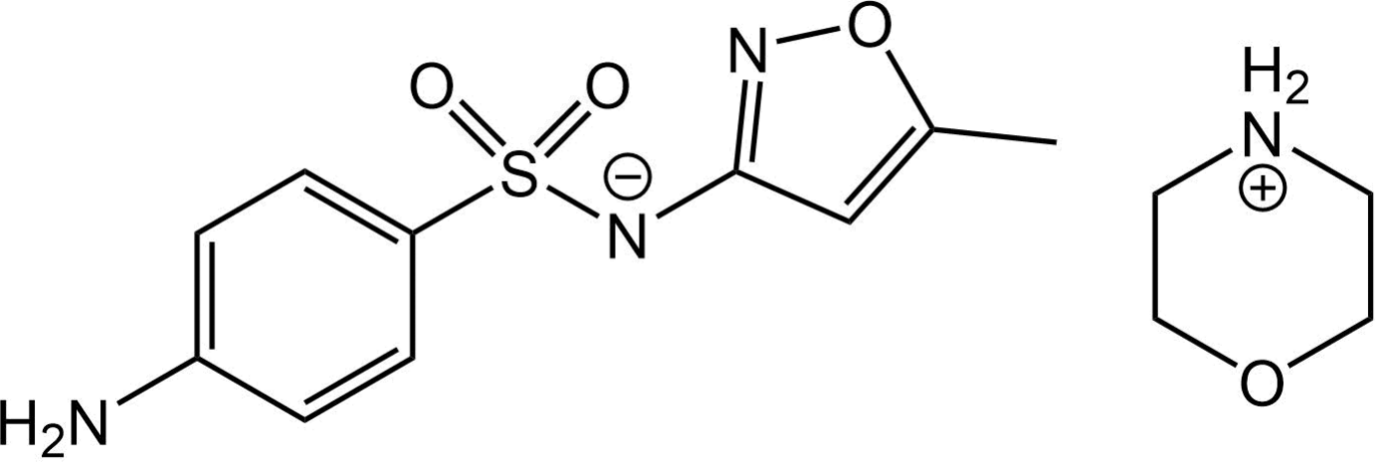	Two known polymorphs	1500 putative structures	S. Coles, S. Aitipamula, J. Cadden

**Table 2:** A summary of the structure generation component of CSP methods applied by each participant group ’–’ indicates that no submission was received by the corresponding group for this phase of the test. The * symbol denotes principal investigator.

Group	Group members	Structure generation method	Structure generation program name
1	Adjiman*, Pantelides*, Bowskill, Sugden, Sanders de Almada, Konstantinopoulos, Zhang	Quasi-random search (Sobol’)	*CrystalPredictor II* (Sugden *et al.*, 2019 ▸)
2	–	–	–
3	Boese*, List, Strasser, Hoja, Braun	Quasi-random search (Sobol’)	*CrystalPredictor II* (Sugden *et al.*, 2019 ▸)
4	–	–	–
5	Day*, Arnold, Bramley, Butler, Cuadrado, Glover, Taylor	Quasi-random search (Sobol’)	*GLEE* (Case *et al.*, 2016 ▸)
6	van Eijck*	Pseudo-random search, Price-Williams exp-6 potential; 6-31G** point charges and intramolecular energies where possible	*UPACK* (van Eijck & Kroon, 2000 ▸)
7	–	–	–
8	Hofmann*, Kuleshova, Pilia	Pseudo-random search	*FlexCryst* (Hofmann & Lengauer, 1997 ▸)
9	–	–	–
10	Jin*, Yang, L. Tan, Chang, Sun, X. Shi, C. Liu, Yue, Fu, Lin, Y. Zhou, Z. Liu, Zeng, Li, B. Shi, T. Zhou, Greenwell, Bellucci, Sekharan	AI-enhanced Self-adaptive Monte Carlo method	*XtalCSP* (Zhang *et al.*, 2018 ▸)
11	Johnson*, Otero-de-la-Roza*, Clarke, Rumson, Mayo, A. J. A. Price	Evolutionary algorithm; atomic charges; multipoles and exp-6; B86bPBE-XDM/PAW	*USPEX* (Oganov & Glass, 2006 ▸)
12	Jose*, Ramteke	Molecular electrostatic potential topography feature space-based approach	LOGOS algorithm
13	Khakimov*, Pivina	Grid search, Empirical potential	*PMC* (Dzyabchenko, 2008 ▸)
14	–	–	–
15	–	–	–
16	Marom*, Isayev*, Anstine, Bier, Hutchison, Nayal, O’Connor, Tom, Zubatyuk	Pseudo-random generation, System-specific AIMNet machine learned potentials	*Genarris* (Tom *et al.*, 2020 ▸), *AIMNet2* (Zubatyuk *et al.*, 2019 ▸)
17	Matsui*, Shinohara	Pseudo-random search, Dreiding force field	N/A
18	Mohamed*, Dhokale, Saeed, Alkhidir, Almehairbi	Quasi-random search (Sobol’)	*CrystalPredictor II* (Habgood *et al.*, 2015 ▸)
19	Muddana*, Jain, Darden, Skillman	Pseudo-random search, atomic multipole force field, IEFF optimization	N/A
20	Neumann*, Anelli, Woollam, Abraham, Dietrich, Firaha, Helfferich, Y. M. Liu, Mattei, Sasikumar, Tkatchenko, van de Streek	Monte Carlo parallel tempering, tailor-made force field	*GRACE* (Neumann, 2008 ▸; Firaha *et al.*, 2023 ▸)
21	Obata*, Goto*, Utsumi, Ikabata, Okuwaki, Fukuzawa, Nakayama, Yonemochi	Grid search, MMFF94s	*CONFLEX* (Ishii *et al.*, 2020 ▸; Goto *et al.*, 2021 ▸)
22	Oganov*, Maryewski, Momenzadeh-Abardeh, Bahrami, Salimi	Evolutionary search	*USPEX* (Glass *et al.*, 2006 ▸)
23	Pickard*, Cheng, Brandenburg	Polymorph search using a Delta-learning potential at a finite temperature	*AIRSS* ‘buildcell’ (Pickard & Needs, 2006 ▸; Pickard & Needs, 2011 ▸)
24	S. L. Price*, L. S. Price, Guo, Francia, Salvalaglio, Ding	Quasi-random (Sobol’)/Grid search; atomic multipoles + empirical exp-6	*CrystalPredictor II* (Habgood *et al.*, 2015 ▸)/*MOLPACK* (Holden *et al.*, 1993 ▸)
25	Shang*, Z.-P. Liu	Rigid-SSW+GAFF, NN potential vdw-DF2	*LASP* (Huang *et al.*, 2019 ▸)
26	Szalewicz*, Ishaque, Nikhar, Podeszwa, Rogal, Vogt-Maranto	Pseudo-random search, SAPT(DFT) fitted intermolecular potential, PBE-D3 monomer deformation energy penalties	*UPACK* (van Eijck & Kroon, 2000 ▸)
27	Tuckerman*, Szalewicz*, Bhardwaj, Chan, Hong, Ishaque, Jing, Melkumov, Nikhar, Podeszwa, Rehman, Rogal, Song, Vogt-Maranto	Pseudo-random or combined pseudo-random and parallel tempering searches using extended variable framework, SAPT(DFT) or PBE0-D3 fitted intermolecular potentials, GAFF intramolecular potentials (XXX, XXXII, XXXIII), PBE0-D3, PBE-D3, OPLS, or GAFF monomer deformation energy penalties)	*EVCCPRE* (Chan & Tuckerman, 2024 ▸) or *UPACK* (van Eijck & Kroon, 2000 ▸)
28	*(Withdrawn)*	Random/genetic search, GAFF potential, DFTB+	*PyXtal* (Fredericks *et al.*, 2021 ▸)

**Table 3:** Total number of groups who attempted predictions and who submitted a structure found to match the experimental forms of each target compound (where A, B and C refer to different polymorphs)

Target system	Attempted predictions	Number of times generated
XXVII	14	A_all atoms_: 6, A_core atoms_: 8
XXVIII†	8	5
XXIX	19	1‡
XXX	13	A: 2, B: 3
XXXI	17	A: 10, B: 9, C: 0
XXXII	13	A: 3, B: 2
XXXIII	14	A: 5, B: 4

† The experimentally known form of XXVIII was made available prior to results submission.

‡ One additional polytypic structure (every sixth molecular layer inverted) was identified, see Section 4.4.

**Table 4:** Results from structural comparisons of putative structures submitted by each group with the experimental structures of XXVII–XXX, where for XXVII, ‘all atoms’ refers to comparisons including all atoms in the structure, and ‘core atoms’ refers to comparisons excluding the triisopropyl groups A blank result indicates no attempted prediction, ‘–’ indicates an attempted prediction with no matches identified, and a number refers to the RMSD (Å) of a structural match. (Comparisons were made using COMPACK with a 30-molecule cluster, and distance and angle tolerances of 35% and 35°, respectively.)

	XXVII		XXVIII	XXIX	XXX	
Group	A_all atoms_ (RMSD_30_)	A_core atoms_ (RMSD_30_)	A (RMSD_30_)	A (RMSD_30_)	A (RMSD_30_)	B (RMSD_30_)
1				–		
3				–		
5	–	0.965		–	–	0.656
6	–	0.495	–	–	–	–
8	–	–	0.386			
10	0.647	0.258	0.234	(0.216)†	0.233_min_	0.194
11				–		
12				–	–	–
13				–	–	–
16	0.415	0.344		–		
17	–	–				
18				–	–	–
19	–	–		–	–	–
20	0.126	0.110	0.142	0.116	0.091_maj_,	0.086
					0.172_min_	
21	0.826	0.677		–	–	–
22	–	–	–	–	–	–
23				–		
24	0.661	0.559	0.460	–	–	–
25	0.210	0.183	0.194	–		
26						
27	–	–	–	–	–	–
28	–	–		–	–	–

† Polytypic structure (every sixth molecular layer inverted) identified as not a true structural match to experiment, see Section 4.4.

**Table 5:** The methods and results from the cocrystal stoichiometry prediction exercise for target XXX

			Predicted rank		
Group	Method	Predicted stoichiometry (CBN:TMP)	Form A_maj_	Form A_min_	Form B
5	Stoichiometric sum of calculated energies for pure component crystal structures obtained from CSD	2:1, 1:1 - correct	–	*n/a* †	–
6	Bespoke method (see SI-B)	1:2, 1:1 - incorrect	–	–	–
10	Thermodynamic cycle	2:1, 1:1 - correct	–	1 (0 K),	11 (0 K),
				1 (298 K)	9 (298 K)
12	None - modelled 1:1 only	1:1 only - incorrect	–	–	–
13	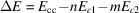	1:2, 1:1 - incorrect	–	–	–
18	Stoichiometric sum of calculated energies for pure component crystal structures obtained from CSD	1:2, 1:1 - incorrect	–	–	–
19	Guessed based on hydrogen bond donors/acceptor ratio	2:1, 1:1 - correct	–	–	–
20	Convex-hull algorithm	2:1, 1:1 - correct	2 (298 K)	2 (298 K)	5 (298 K)
21	Cohesive energy of molecules (*E*_coh_ = Δ*E*_intra_ + *E*_inter_, Δ*E*_intra_ = *E*_intra, solid_ − *E*_intra, gas_)	2:1, 1:1 - correct	–	–	–
22	Convex-hull algorithm	1:2, 2:1, 1:1‡	–	–	–
24	Δ*E*_CC_ = [*E*_latt_(*C*_*m*_:*T*_*n*_) − *nE*_latt_(*T*)]/*m* − *E*_latt_(*C*)	1:2, 1:1 - incorrect	–	–	–
27	Energies with respect to intermolecular energies of the mono-crystals for the two components	2:1, 1:1 - correct	–	–	–
28	Guessed based on hydrogen bond donors/acceptor ratio	2:1, 1:1 - correct	–	–	–

† Structure was not submitted in the ranked list.

‡ Three stoichiometries predicted to be stable, see Section 4.5.

**Table 6:** Results from structural comparisons of putative structures submitted by each group with the experimental structures of XXXI–XXXIII A blank result indicates no attempted prediction, ‘–’ indicates an attempted prediction with no matches identified, and a number refers to the RMSD (Å) of a structural match. ‘maj’ and ‘min’ refer to the major and minor components of disorder, respectively. ‘^’ refers to a tentative structural match due to high RMSD.

	XXXI			XXXII		XXXIII	
Group	A (RMSD_30_)	B (RMSD_30_)	C (RMSD_30_)	A (RMSD_30_)	B (RMSD_30_)	A (RMSD_30_)	B (RMSD_30_)
1	0.138_maj_	0.881	–	–	–	–	–
	0.274_min_						
3	0.806_maj_	0.320	–	–	–		
	0.242_min_						
5	0.347_maj_	0.633	–	–	–	0.490	0.534
	0.356_min_						
6	–	0.604	–	–	–	–	–
8	–	–	–			–	–
10	0.125_maj_	0.418	–	0.227_maj_	0.363	0.190	0.263
	0.269_min_						
11							
12	–	–	–				
13						–	–
16	0.144_maj_	0.327	–				
	0.233_min_						
17							
18	0.672_min_	–	–	–	–		
19	0.403_maj_	0.684	–	–	–	–	–
	0.432_min_						
20	0.168_maj_	0.351	–	0.148_maj_	0.191	0.114	0.215
	0.245_min_						
21	–	–	–			0.363	–
22	–	–	–	–	–	–	–
23							
24	0.856_maj_	0.866	–	–	–	0.359	0.270
	0.315_min_						
25	–	–	–	^1.029_maj_	–	–	–
26	0.857_min_	–	–				
27				–	–	–	–
28	–	–	–	–	–	–	–

**Table 7:** Summary of CPU core hours reported per target molecule for each group where predictions were attempted

Group	XXVII	XXVIII	XXIX	XXX	XXXI	XXXII	XXXIII	Total	Processors
1			652,495		840,000	1,597,000	412,000	3,501,495	AMD EPYC 7742 / Intel Xeon E5-2620,
									E5-2650 v4, Gold 6248, E5-2695
3			1,600,000		1,500,000	3,600,000		6,700,000	Intel Xeon X5650, E5-2650 v3, Silver 4214R, Platinum 8174
5	768,766		33,000	2,900,000	510,563	846,698	228,957	5,287,984	Intel Skylake 2.0 GHz
6	8,120	1,350	1,310	9,800	1,470	2,900	4,980	29,930	Various computers,
									CPU times standardized to 2.66 GHz Intel Quad 9400
8	3,200	10			4,000		1,840	9,050	Intel Xeon 2650
10	772,500	1,242,500	1,146,588	644,927	381,672	644,927	612,500	5,445,614	Intel Xeon Platinum 8124M
11			643,882					643,882	Intel Xeon E5-2683 v4
12			20,000	80,000	20,000			120,000	Intel Xeon Gold 6132
13			350	1,500			500	2,350	Intel Xeon E5450
16	1,700,000		2,128,000		630,000			4,458,000	AMD EPYC 7742 / Intel Platinum 8280
									Nvidia RTX 3090, GTX 1080, GTX 1080ti / Tesla V100S
17	95,819							95,819	Intel Xeon Gold 6154
18			1,050	36,864	632	1,561		40,107	Intel Xeon Gold 6230R
19	30,000		40,000	1,250,000	140,000	400,000	60,000	1,920,000	Intel Xeon Haswell E5-2666 v3
20	1,022,976	283,538	755,712	1,769,472	1,028,064	3,935,232	728,064	9,523,058	Intel Xeon E5-2650 v4
21	333,586		92,890	580,436	1,889,649		477,210	3,373,771	Intel Xeon Gold 6154, 6132, FUJITSU A64FX
22	20,000	2,000	15,000	180,000	20,000	25,000	25,000	287,000	Intel Xeon Gold 6230
23			10,000					10,000	Intel Xeon Scalable Processors / Apple M1
24	450,290	89,666	76,541	100,000	49,177	244,520	123,427	1,133,621	Intel Xeon E5-2650v3, L5630 & E5-2660v4 mixed clusters
25	55,150	29,691	4,784		6,476	76,161	34,648	206,910	Intel Xeon Gold 6248, Platinum 8168
26					28,332			28,332	Intel Xeon Gold
27	1,280,566	60,457	242,424	213,722		1,663,940	150,650	3,611,759	Intel Xeon Platinum, Gold-6132, Xeon E5-2695 v3
28	1,600		1,500	7,680	1,500	1,500	1,500	15,280	Intel Xeon E5-2697 A v4

## Footnotes

1 https://github.com/michelegalasso/xrpostprocessing.

2 The AutoFIDEL Python script was written by Jonas Nyman based on the FIDEL algorithm described by Habermehl *et al.* (2014 ▸) and has been copyrighted to the CCDC.
