# Supplementary material for: The seventh blind test of crystal structure prediction: structure generation methods
Source: Acta Crystallogr B Struct Sci Cryst Eng Mater. 2024 Dec 1;80(Pt 6):517–47. doi: 10.1107/S2052520624007492 (PMC11789161; doi:10.1107/S2052520624007492)
Supplement: Supplementary file 1 [file b-80-00517-sup1.pdf]

Supplementary Information A. The Seventh Blind Test of Crystal Structure Prediction: Structure Generation Methods

Lily M. Hunnisett, *et al.* *

The Cambridge Crystallographic Data Centre, 12 Union Road, Cambridge CB2 1EZ, UK. E-mail: lhunnisett@ccdc.cam.ac.uk

Contents

1 Additional figures and tables 3

2 Clustering of XXVII landscapes 31

3 Theoretical investigation of the disorder of molecule XXVII 33

3.1 Introduction 33

3.2 Structure preparation and forcefield 33

3.3 Equilibration at room temperature and pressure 34

3.4 Molecular dynamics setup and simulation's results 35

3.5 Analysis of the free energy landscape from well-tempered metadynamics 36

4 Short-range similarity predicted structures of XXIX 40

5 Crystal Structure Similarity 48

5.1 Introduction 48

5.2 Comparisons of the AMD and PDD approaches to the Crystal Packing Similarity tool 48

5.3 Assessment of similarity among submitted sets 50

6 Previous Blind Tests Target Compounds 54

7 DOI links for submitted sets of structures 58

1. Additional figures and tables

Table 1. *Summary of known crystal structures for each target system.*

Target system	Form	CSD Refcode	Description	Comments
XXVII	A	N/A*	Collected at 90 K, no disorder.	Initial crystal structure obtained prior to the start of test (September 2020). Bromine contamination not correctly included.
XXVII	A	XIGYUL	Collected at 90 K, contains bromine contamination.	Re-refinement of initial crystal structure to include known bromine contamination.
XXVII	A	XIFZOF01	Collected at 100 K, disorder of both TIPS groups.	Redetermination of initial crystal structure, no bromine contamination.
XXVII	A	XIFZOF	Collected at 290 K, disorder of one TIPS group.	Room temperature crystal structure, obtained at end of test (July 2022).
XXVIII	A	OJIGOG01	Collected at 150 K.	Original crystal structure obtained prior to start of test (September 2020).
XXIX	A	FASMEV	Collected at 274 K.	
XXX	A	MIVZEA	Collected at 100 K, disorder of cannabinol hydrocarbon chain.	Hemi-tetramethylpyrazine co-crystal of cannabinol.
XXX	B	MIVZIE	Collected at 100 K.	Mono-tetramethylpyrazine co-crystal of cannabinol.
XXXI	A	ZEHFUR02	Collected at 120 K, disorder of fluorinated ring.	
XXXI	B	ZEHFUR	Collected at 200 K.	
XXXI	C	ZEHFUR01	Collected at 120 K	Contains solvent-templated void channels.
XXXII	A	JEKVII	Collected at 90 K, disorder of the trifluoromethyl group.	
XXXII	B (LT)	JEKVII01	Collected at 90 K.	Referred to as 'Form M' in experimental report from the providers.
XXXII	B (RT)	N/A*	Collected at 297 K	Incorrect room temperature structure determined from PXRD
XXXII	B (RT)	N/A*	N/A	Structural redetermination from PXRD, originating from CSP from Group 20
XXXIII	A	ZEGWAN	Collected at 296 K.	Disappearing polymorph.
XXXIII	B	ZEGWAN01	Collected at 297 K.	

*Structures are provided in the CIF format in SI-D.

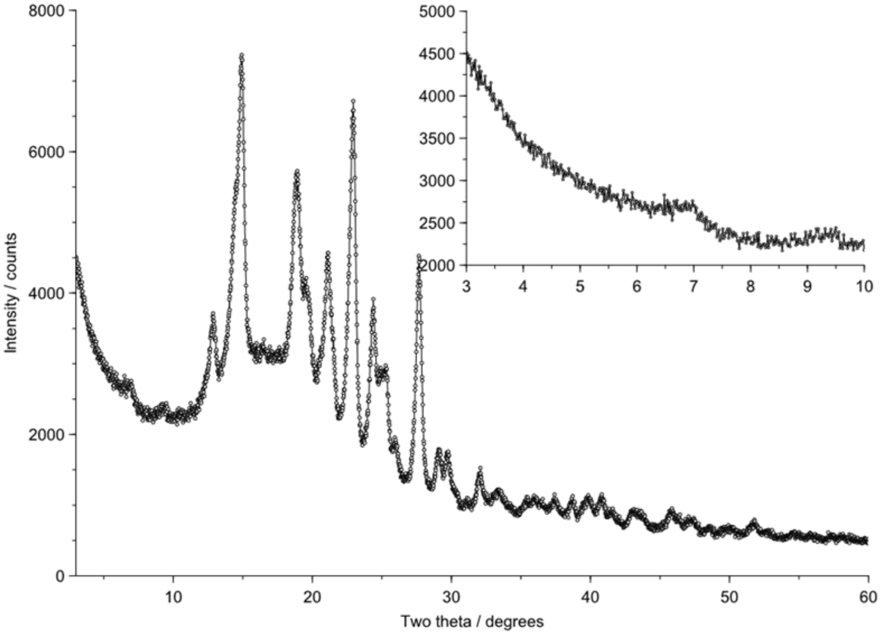

Figure: A simulated PXRD pattern of an experimentally observed structure of target XXIX (Instrument geometry = capillary transmission, data collection temperature = 273 K, step size = 0.017 degrees, incident wavelength = 1.5406 Å (monochromatic))

Fig. 1. The simulated PXRD pattern and metadata figure provided to participants for the PXRD-assisted prediction exercise of XXIX.

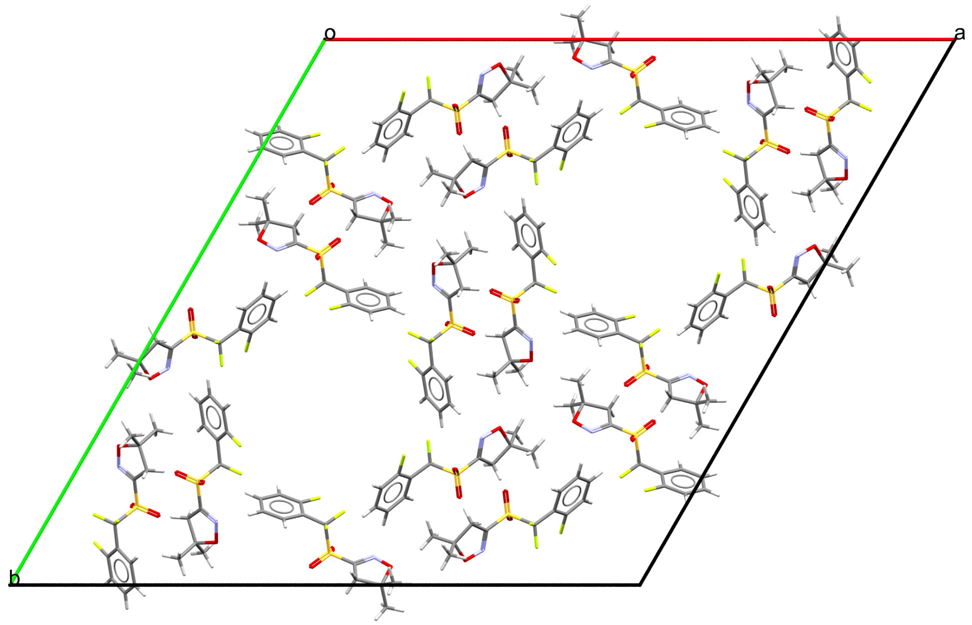

Fig. 2. Crystal packing of XXXI Form C viewed along the **c** axis showcasing the solvent-filled void channels within the structure. The solvent molecules were not refined but removed with the SQUEEZE method.

Table 2: The CIF name, RMSD, and visualisation of the comparison of predicted structures found to match the experimental form of XXVII (all atoms) for each participating group. (30 molecule cluster, 35% and 35° distance/angle tolerances.)

Group	CIF name	RMSD	Comparison visualisation
10	1266_Z1_st_YT8AefeP-XwAwmpA5.cif	0.647	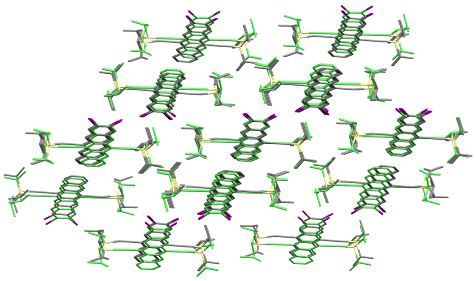
16	MI_0297_0c30f3c7.cif	0.415	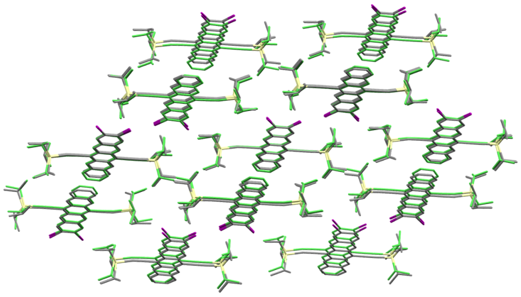
20	structure_8.cif	0.126	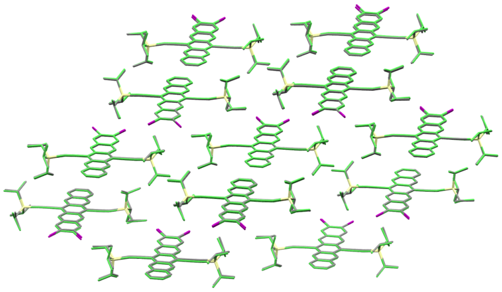
21	XXVII-0040.cif	0.826	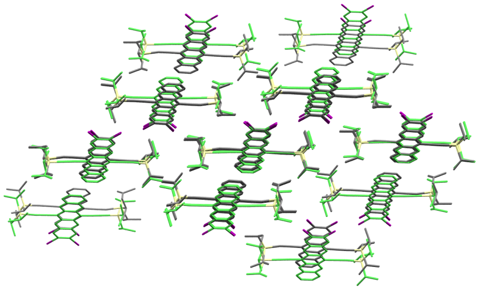

24	BXXVII_m1mp_-ca21.cif	0.661	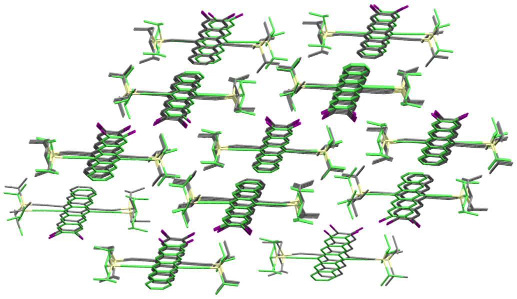
25	XXVII_4.cif	0.210	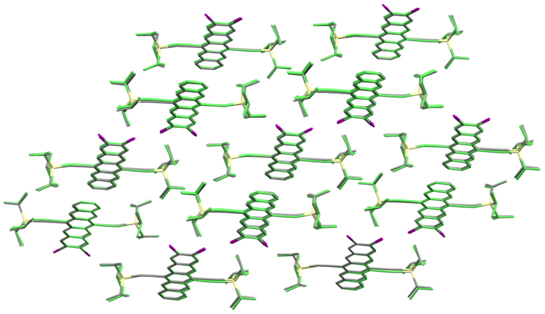

Table 3: The CIF name, RMSD, and visualisation of the comparison of predicted structures found to match the experimental form of XXVII (core atoms only) for each participating group. (30 molecule cluster, 35% and 35° distance/angle tolerances.)

Group	CIF name	RMSD	Comparison visualisation
5	27_XXVII_661_gopt-QR-2-2707-3.cif	0.965	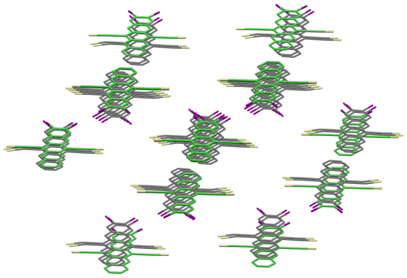
6	vaneijck-XXVII.0038.cif	0.495	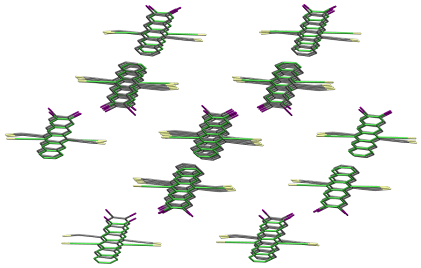
10	1026_Z2_st_YUqI9l-_5MQA_UE_C.cif	0.258	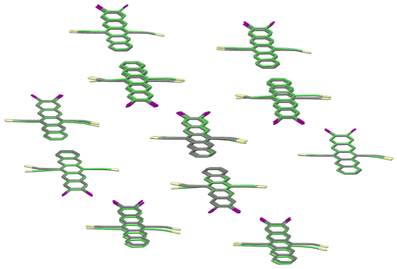
16	MI_0297_0c30f3c7.cif	0.344	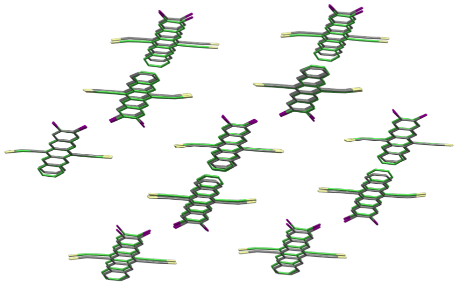
20	structure_8.cif	0.110	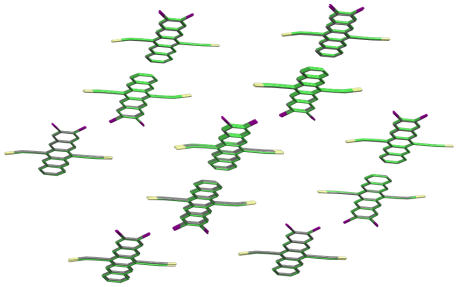

21	XXVII-0061.cif	0.677	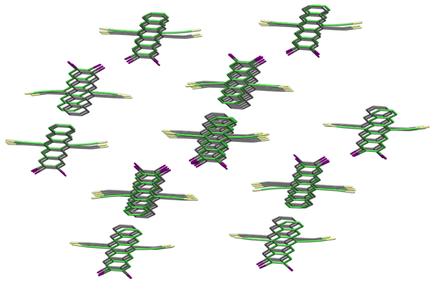
24	BXXVII_m1mp_ca-21.cif	0.559	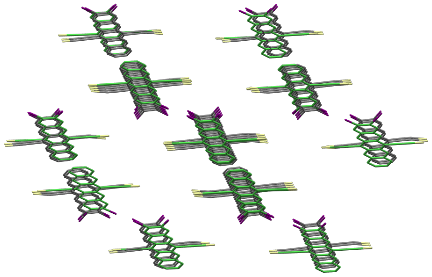
25	XXVII_108.cif	0.183	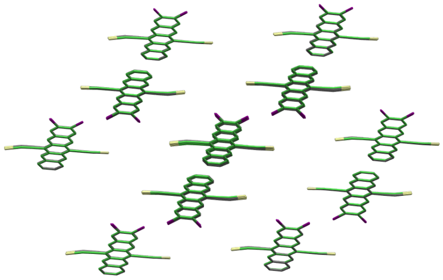

Table 4. *The CIF name, RMSD, and visualisation of the comparison of predicted structures found to match the experimental form of XXVIII for each participating group. (30 molecule cluster, 35% and 35° distance/angle tolerances.)*

Group	CIF name	RMSD	Comparison visualisation
8	00000009.cif	0.386	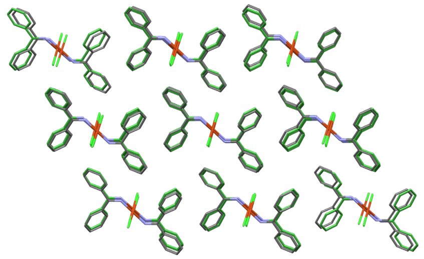
10	2_T1_st_YK2w9oA-jAAAnSf6Z.cif	0.234	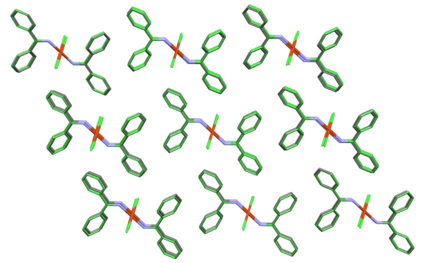
20	structure_1.cif	0.142	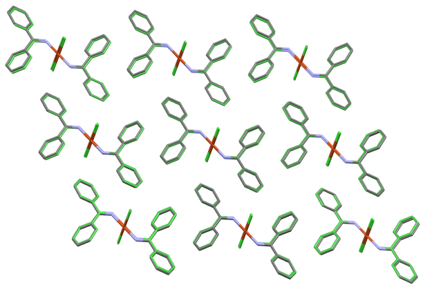
24	XXVIII_dfCB11_-CB11.cif	0.460	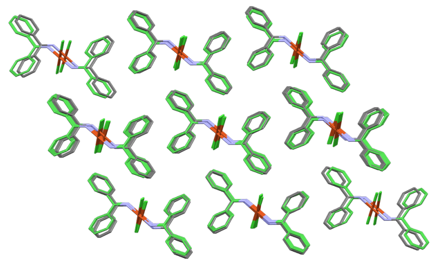
25	XXVIII_1.cif	0.194	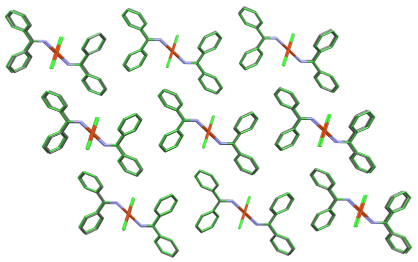

Table 5. *The CIF name, RMSD, and visualisation of the comparison of predicted structures found to match the experimental form of XXIX for each participating group. (30 molecule cluster, 35% and 35° distance/angle tolerances.)*

Group	CIF name	RMSD	Comparison visualisation
10	1_z3_st_YSx-RB1Za6zN2UQXt.cif	0.216	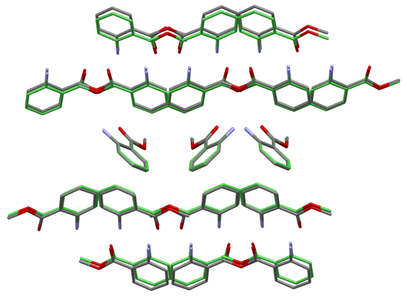
20	structure_1.cif	0.116	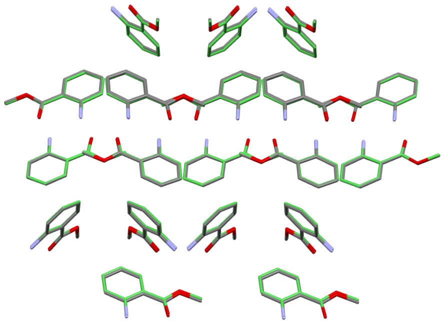

Table 6. *The CIF name, RMSD, and visualisation of the comparison of predicted structures found to match the experimental form of XXX (Form A, major disorder component) for each participating group. (30 molecule cluster, 35% and 35° distance/angle tolerances.)*

Group	CIF name	RMSD	Comparison visualisation
20	A1B2_structure_56.cif	0.091	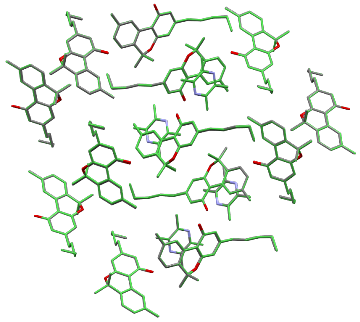

Table 7. *The CIF name, RMSD, and visualisation of the comparison of predicted structures found to match the experimental form of XXX (Form A, minor disorder component) for each participating group. (30 molecule cluster, 35% and 35° distance/angle tolerances.)*

Group	CIF name	RMSD	Comparison visualisation
10	1_A2_st_YFhl-gwufAABru26.cif	0.233	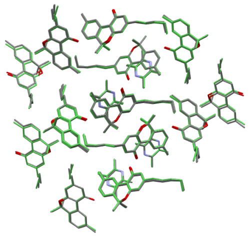
20	A1B2_structure_62.cif	0.172	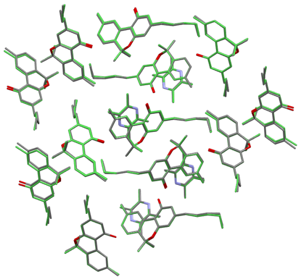

Table 8. *The CIF name, RMSD, and visualisation of the comparison of predicted structures found to match the experimental form of XXX (Form B) for each participating group. (30 molecule cluster, 35% and 35° distance/angle tolerances.)*

Group	CIF name	RMSD	Comparison visualisation
5	XXX_9d_1.1_QR-14-125731-3.cif	0.656	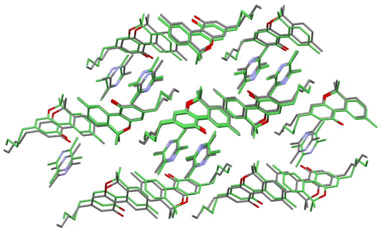
10	11_AB_st_X_fxy-C1FJgABaZL2.cif	0.194	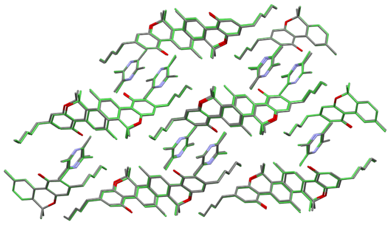
20	A1B1_structure_1.cif	0.086	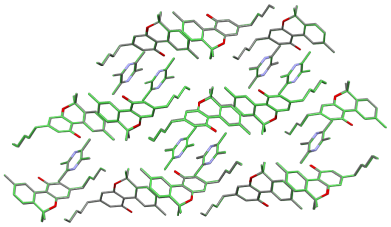

Table 9: The CIF name, RMSD, and visualisation of the comparison of predicted structures found to match the experimental form of XXXI (Form A, major disorder component) for each participating group. (30 molecule cluster, 35% and 35° distance/angle tolerances.)

Group	CIF name	RMSD	Comparison visualisation
1	VASP_MIN_3.cif	0.138	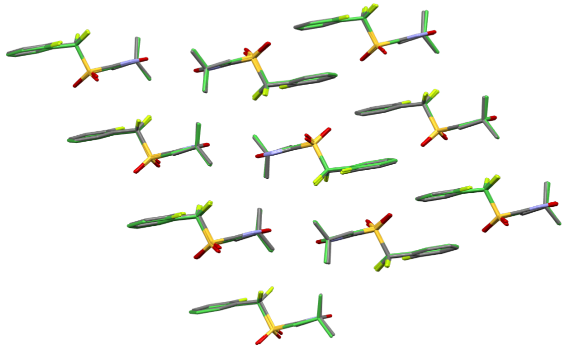
3	xxxi-ca120_e0-s1943-co-23.cif	0.806	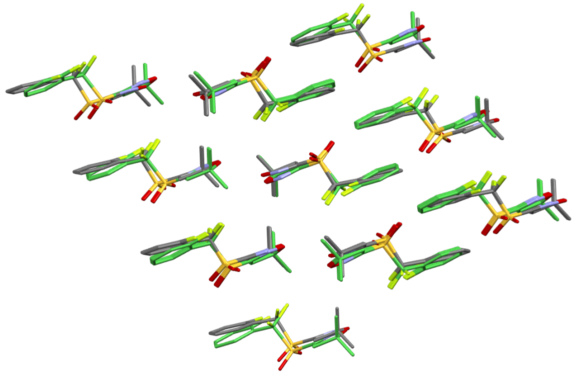
5	scan_52-QR-14-17219-3_dftbopt_final.cif	0.347	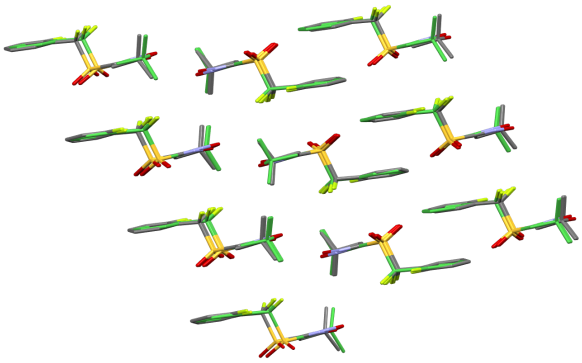

10	data_3_z1_st_YDMR-5hjUQABVngB.cif	0.125	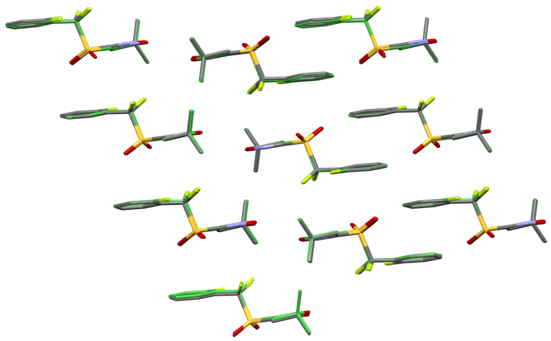
16	MI_0003_dft_-ec2f8086.cif	0.144	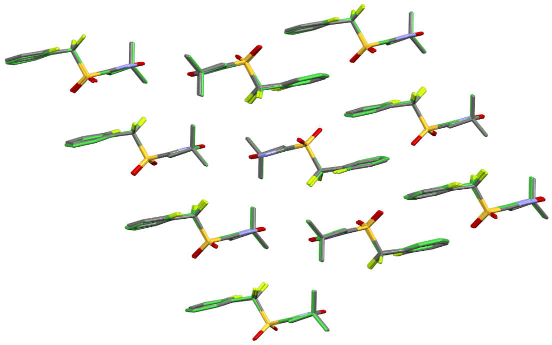
19	XXXI-1-OpenEye_structure_106.cif	0.403	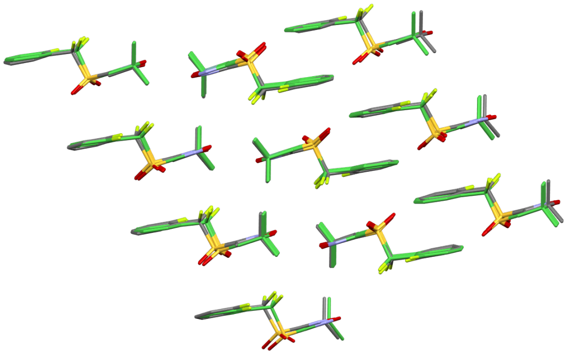

20	structure_21.cif	0.168	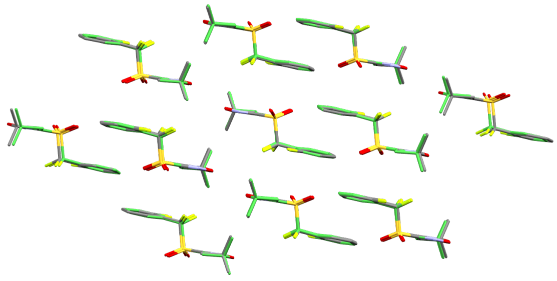
24	BTXXXI_df2_2.cif	0.856	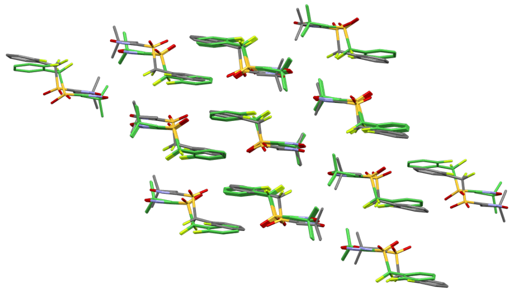

Table 10: The CIF name, RMSD, and visualisation of the comparison of predicted structures found to match the experimental form of XXXI (Form A, minor disorder component) for each participating group. (30 molecule cluster, 35% and 35° distance/angle tolerances.)

Group	CIF name	RMSD	Comparison visualisation
1	VASP_MIN_8.cif	0.274	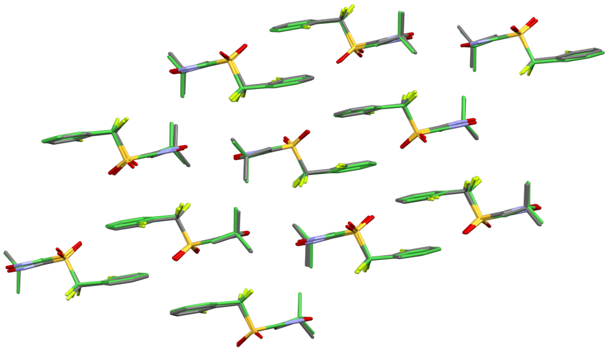
3	xxxi-ca269_e0-s5986-co-18.cif	0.242	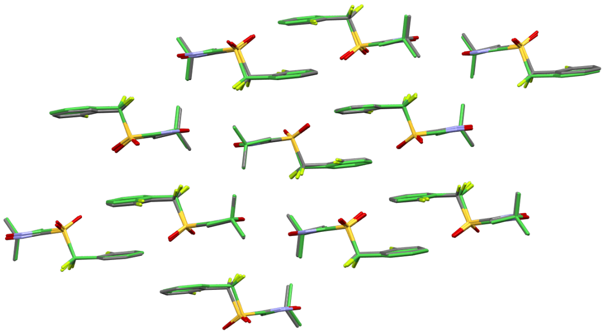
5	XXXI_conf_7_opt-QR-14-32986-3_dftbopt_final.cif	0.356	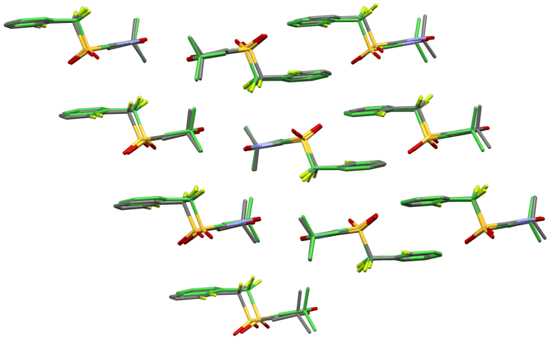

10	data_6_z1_st_YN-rfI1Za63KAghid.cif	0.269	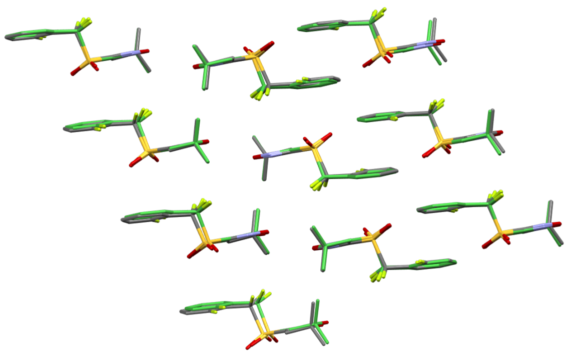
16	MI_0015_dft-_ccf83e9e.cif	0.233	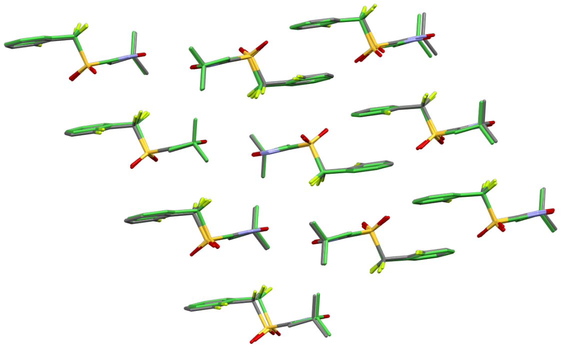
18	data_43.cif	0.672	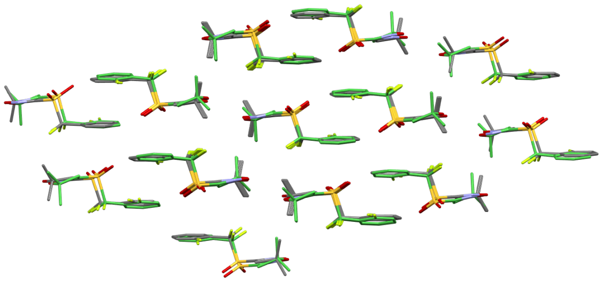

19	XXXI-1-OpenEye-_structure_56.cif	0.432	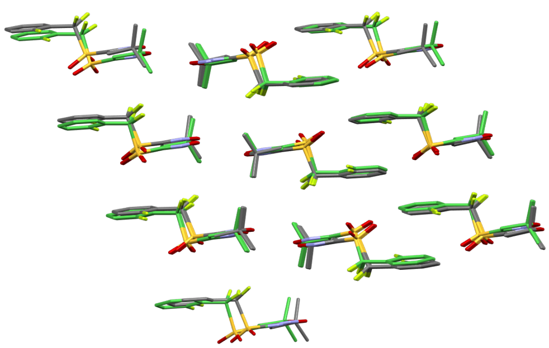
20	structure_115.cif	0.245	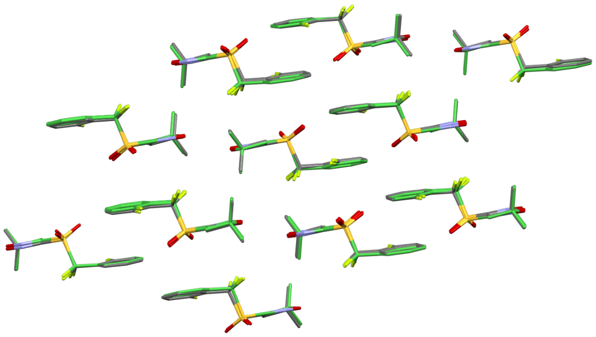
24	BTXXXI_df1_1.cif	0.315	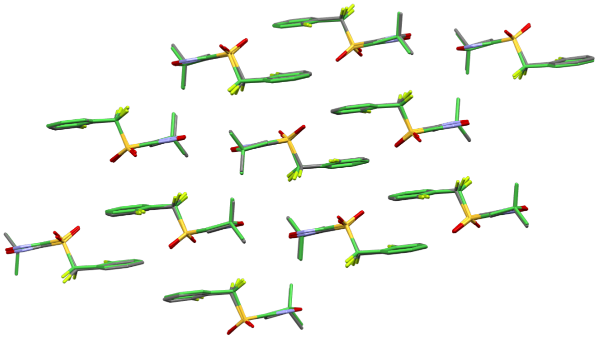

26	data_1192.cif	0.857	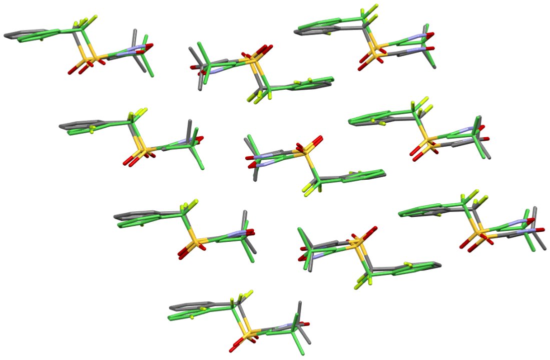

Table 11: The CIF name, RMSD, and visualisation of the comparison of predicted structures found to match the experimental form of XXXI (Form B) for each participating group. (30 molecule cluster, 35% and 35° distance/angle tolerances.)

Group	CIF name	RMSD	Comparison visualisation
1	CSOFM_00836.cif	0.881	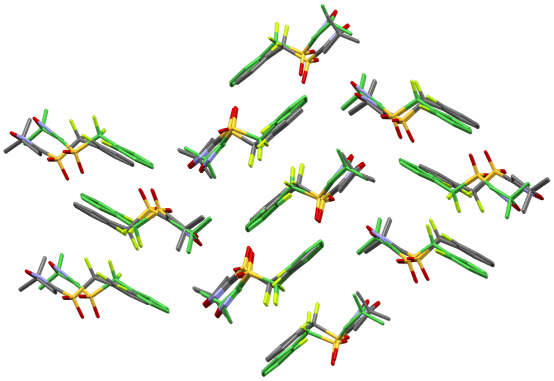
3	xxxi-ca120_e0-s4841-dma-44.cif	0.320	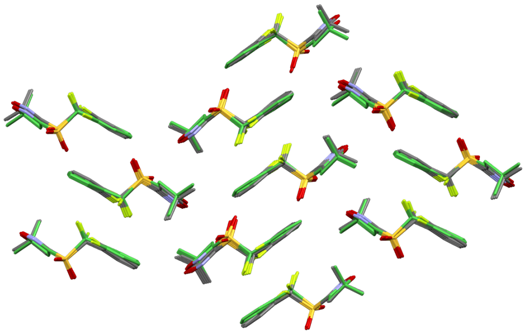
5	scan_4-QR-14-6524-3_dftbopt_final.cif	0.633	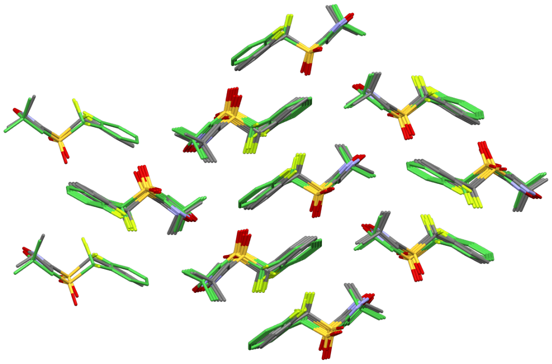

6	data_vaneijck-XXXI.0389.cif	0.604	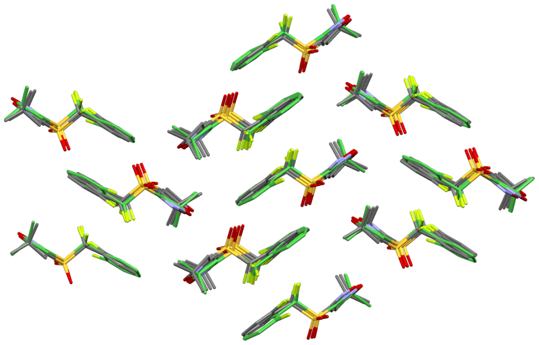
10	data_188_z1_st_YNr-fI1Za63KAghhR.cif	0.418	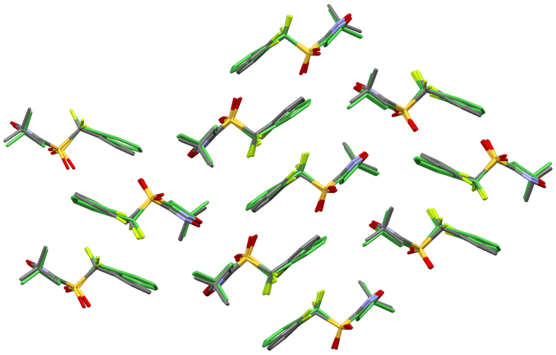
16	MI_0046_dft_-3199da20.cif	0.327	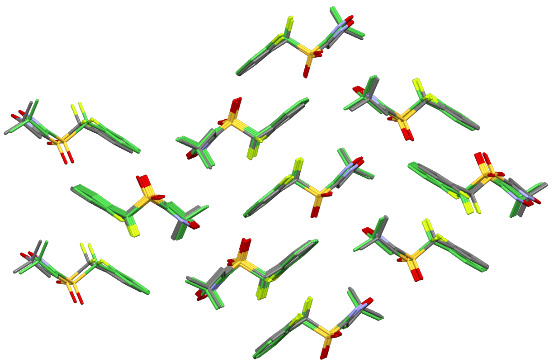

19	XXXI-1-OpenEye-_structure_1154.cif	0.684	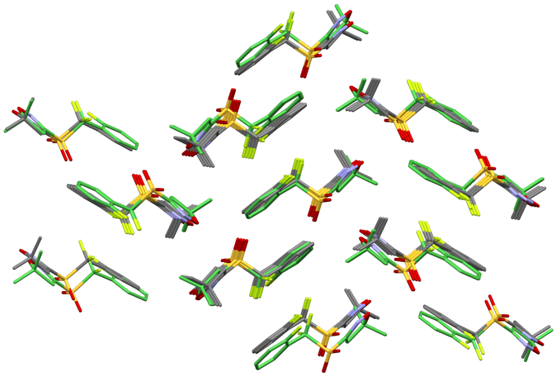
20	structure_217.cif	0.351	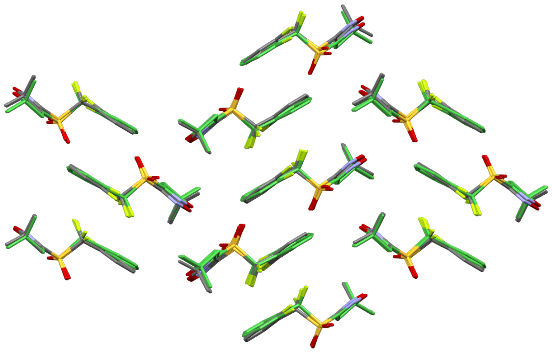
24	BTXXXI_df239_239.cif	0.866	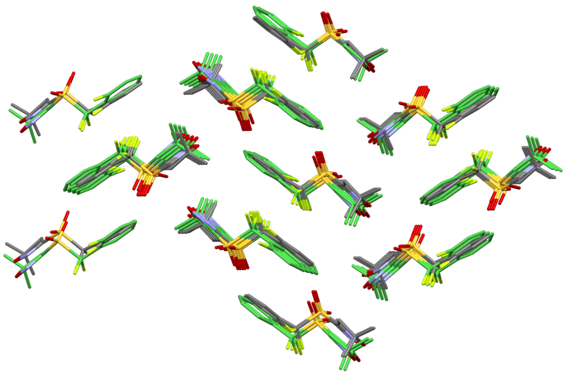

Table 12. *The CIF name, RMSD, and visualisation of the comparison of predicted structures found to match the experimental form of XXXII (Form A, major disorder component) for each participating group. (30 molecule cluster, 35% and 35° distance/angle tolerances.)*

Group	CIF name	RMSD	Comparison visualisation
10	data_815_z1_st_Y-Ul4CeqPPieGr_56.cif	0.227	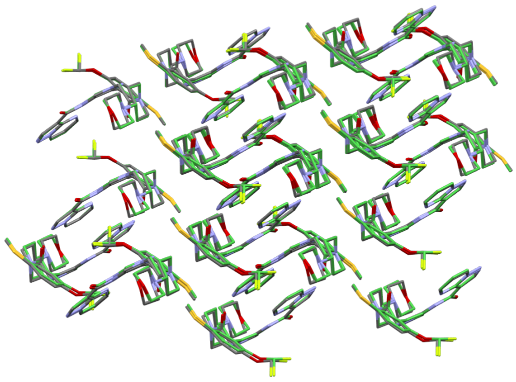
20	structure_159.cif	0.148	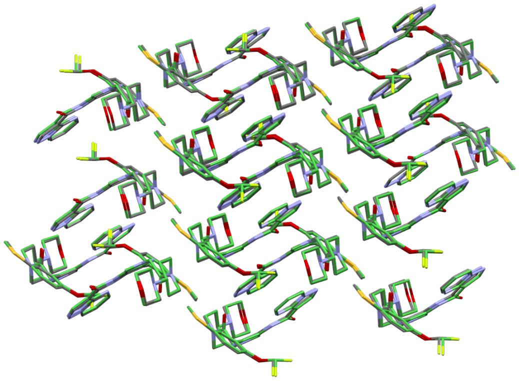
25	XXXII_592.cif	1.03	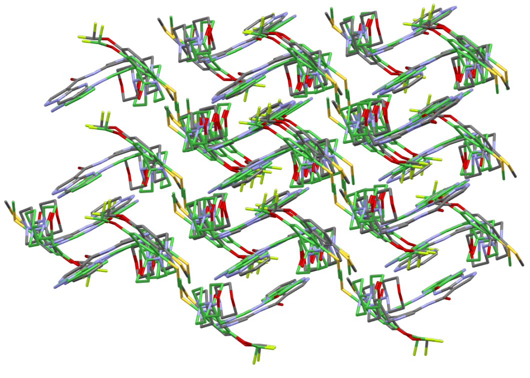

Table 13. *The CIF name, RMSD, and visualisation of the comparison of predicted structures found to match the experimental form of XXXII (Form B) for each participating group. (30 molecule cluster, 35% and 35° distance/angle tolerances.)*

Group	CIF name	RMSD	Comparison visualisation
10	data_10_z2_st_YUq-NVuqPPieGr_-B.cif	0.363	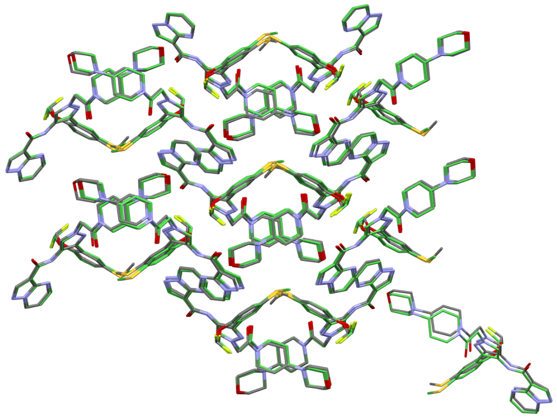
20	structure_71.cif	0.191	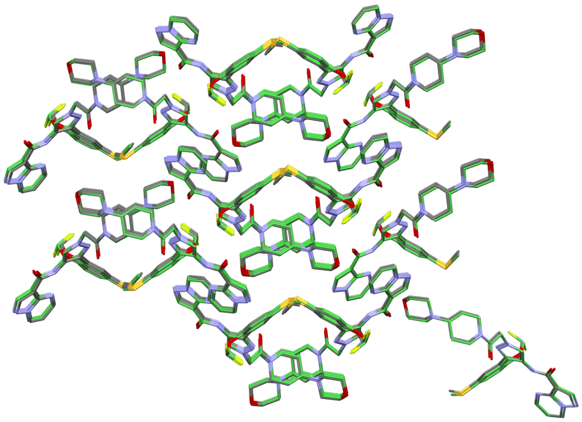

Table 14: The CIF name, RMSD, and visualisation of the comparison of predicted structures found to match the experimental form of XXXIII (Form A) for each participating group. (30 molecule cluster, 35% and 35° distance/angle tolerances.)

Group	CIF name	RMSD	Comparison visualisation
5	33_XXXIII_a_2_opt-.33_XXXIII_c_0_opt-QRBH-15-8-3-61.cif	0.490	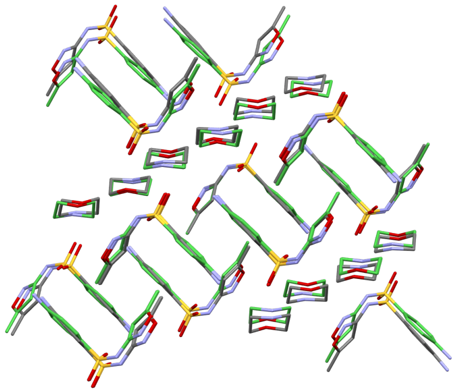
10	data_5_Z1_st_YD2-kLi1FJgABE1aQ.cif	0.190	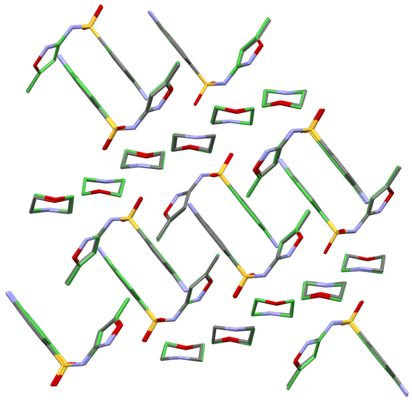
20	structure_19.cif	0.114	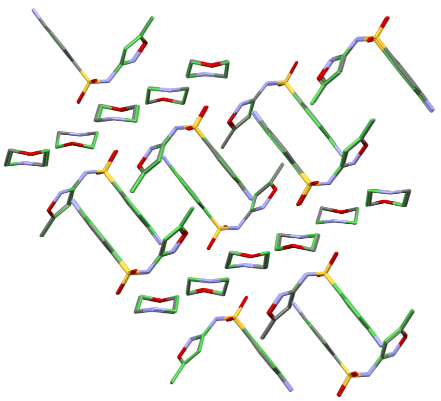

21	XXXIII-0011.cif	0.363	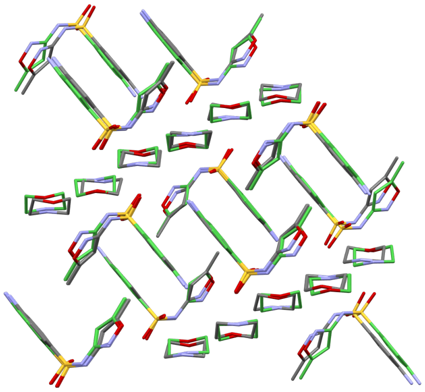
24	XXXIII_dfBa282-_Ba282.cif	0.359	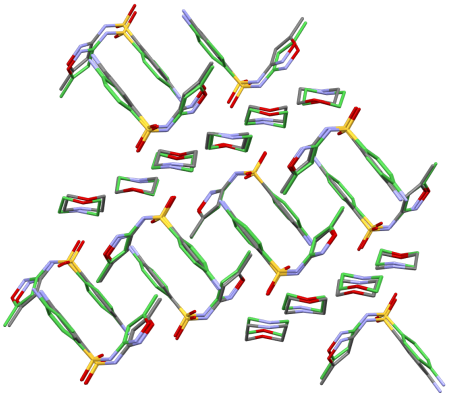

Table 15: The CIF name, RMSD, and visualisation of the comparison of predicted structures found to match the experimental form of XXXIII (Form B) for each participating group. (30 molecule cluster, 35% and 35° distance/angle tolerances.)

Group	CIF name	RMSD	Comparison visualisation
5	33_XXXIII_a_4_opt-.33_XXXIII_c_0_opt-QRBH-33-71-3-6.cif	0.534	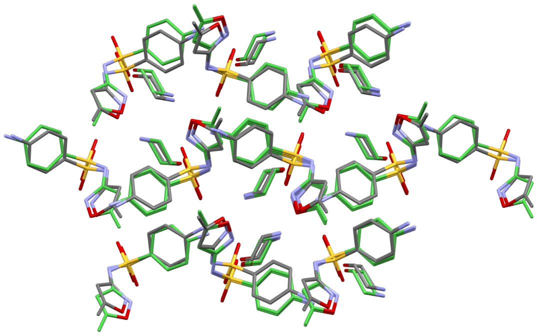
10	data_1_Z1_st_YD2-kLi1FJgABE1Iy.cif	0.263	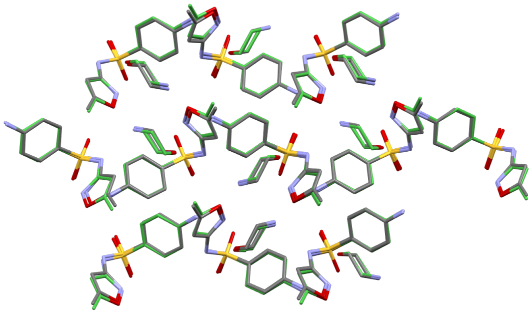
20	structure_1.cif	0.215	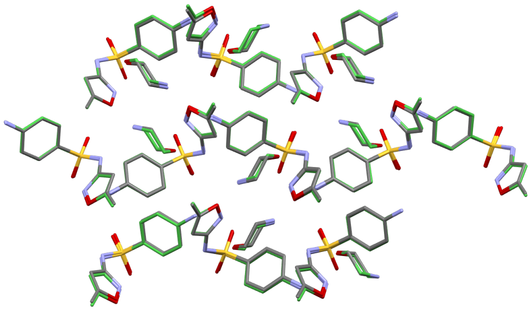

24	XXXIII_dfAa686-<br>_Aa686.cif	0.270	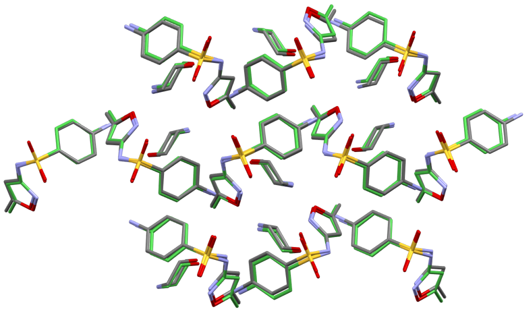

2. Clustering of XXVII landscapes

It has been observed that disorder in an experimental crystal structure often corresponds to multiple CSP-generated structures. Hence for XXVII, it was particularly interesting to analyse whether in each landscape a significant number of structures share the same core packing. It is also of interest to investigate the clustering approaches of each group by applying a standard structure similarity algorithm to every landscape. Strict clustering criteria would result in different molecular conformations while a looser approach would result in greater diversity of packings. Hence, the clustering of individual landscapes was carried out using COMPACK focusing on the core of the molecule only, excluding isopropyl groups, to identify common crystal packing motifs. To avoid an all-versus-all comparison of structures, a leaders-based clustering algorithm was adopted utilising COMPACK alone as the method of comparison (Spath, 1980). The necessarily ambiguous nature of crystal structure comparisons has previously been highlighted (van Eijck, 2005). It must therefore be noted that while the leaders-based clustering algorithm here provides an indication of similarity within the structural landscapes submitted, it is sensitive to both the tolerances specified for COMPACK and the order in which comparisons are carried out, and is not a strict representation of explicit matches otherwise obtained through pairwise comparisons. To reduce the computational intensity of the comparisons further, for each structural comparison, an initial step of a 2-molecule shell was applied to efficiently rule out non-matches, followed by a comparison of 15 molecules with 25%/25° tolerance to confirm similar structures.

For this system in particular, it was interesting to analyse: a) the number of clusters in each individual landscape, indicating how loose the clustering criteria were in each methodology, and b) for each method, whether a large majority of predicted structures represented the experimental crystal packing. Clustering was first carried out for the CSP landscape from each group to reduce the list of structures in each case. A global landscape was then constructed from the condensed list of structures from each group and clustering was then carried out to identify common structures between differing methods.

The clustering of individual landscapes indicates vastly different degrees of common crystal packing populations across the different groups, see SI-A Tab. 2, where a greater number of clusters represents a greater number of different crystal packing motifs of XXVII. This demonstrates the variation of clustering criteria applied across the different methods to exclude duplicate structures during CSP. Higher numbers of clusters (fewer structures with the same packing motif) could indicate that conformational isomers due to TIPS variation have been filtered out during the clustering process prior to the submission of results. This is likely to be the case for groups 5 and 6 where the experimental crystal packing was observed but not with the correct TIPS group conformation. None of the sets of structures contained the experimental crystal packing as the most common packing motif.

Table 16. *Results of clustering (based on the Leaders algorithm) of individual submitted landscapes of XXVII (1500 structures) using COMPACK, excluding isopropyl groups of all structures. The * symbol denotes a cluster containing a structural match to the experimental crystal packing.*

Group	Number of clusters	Size of the cluster matching experimental form	Size of the 3 highest populated clusters
5	657	3	28, 22, 17
6	438	9	77, 43, 33
8	106	-	198, 171, 126
10	240	81	131, 131, 102
16	301	18	91, 69, 44
17	1337	-	16, 15, 8
19	660	-	22, 17, 17
20	109	15	341, 272, 108
21	482	17	32, 32, 29
22	376	-	196, 166, 116
24	972	4	20, 18, 18
25	312	74	125, 118, 74*
26	863	-	12, 10, 7
28	317	-	108, 77, 55

3. Theoretical investigation of the disorder of molecule XXVII

3.1. Introduction

We used molecular dynamics and metadynamics simulations to analyse the disorder of target XXVII form A. This was initially refined at 90 K, showing no disorder and making it a perfect blind test target in the field of organic electronics. Further investigations on the same sample, conducted in May 2023, established however that Bromine contamination was present (CSD: XIGYUL). Molecular dynamics simulations, conducted by group 24 (see SI-B Sec. 18) during the first phase of the test, identified several structures in which the TIPS groups could easily change conformation. This prompted further investigations, both theoretical and experimental, and suggests that there could be dynamic disorder involving the bending of the C-Si-C angles, the rotation of the two TIPS groups, and the rotation of isopropyl groups. On the experimental side, after the end of phase 1 (July 2022), a second refinement of form A (CSD: XIFZOF) was performed using data collected at 290 K. This refinement revealed disorder in one of the triisopropylsilane (TIPS) groups (later defined as TIPS B and shown in Fig. 4A). After the I/Br elemental disorder was detected (May 2023), a third refinement at 100 K on a pure compound was made (CSD: XIFZOF01). This time, the sample was obtained by cooling down from room temperature and exhibited disorder in both TIPS groups. The TIPS previously detected as disordered has the isopropyl groups displaced, probably as a result of the rotation around the Si atom. The other TIPS group shows a change in conformation of one of the isopropyl groups instead. In this section, we provide more information about the molecular dynamics and metadynamics simulations that were carried out on XXVII form A, with respect to Section 4.2 of the main paper. We will describe how the calculations were performed and present the results obtained in detail.

3.2. Structure preparation and forcefield

We used the GAFF forcefield (Wang *et al.*, 2004) with atomtypes assigned with the AmberTools suite (Case *et al.*, 2016) and the point charges computed with the AM1-BCC model (Jakalian *et al.*, 2000). To integrate missing parameters related to the Si atoms, a series of *ad hoc* parameterisation of the bonded terms were performed using *ab initio* simulations at the MP2/6-31G(d) level. This forcefield was previously used to study the first 1000 structures of group 24 and a detail of its parametrisation can be found in Chapter 7 of Ref. Francia (2022).

A supercell of nearly cubic shape with cell edges of approximately 5 nm was generated starting from the experimental form A at 90 K. The atom positions of the resulting 144 molecules, were optimised using the steepest descent algorithm and the neighbour lists were updated every 10 steps using the Verlet cutoff scheme. Electrostatic and van der Waals interactions were calculated using a cutoff of 1.0 nm while long-range interactions were treated with the Smooth Particle Mesh Ewald (PME) and Lennard-Jones PME.

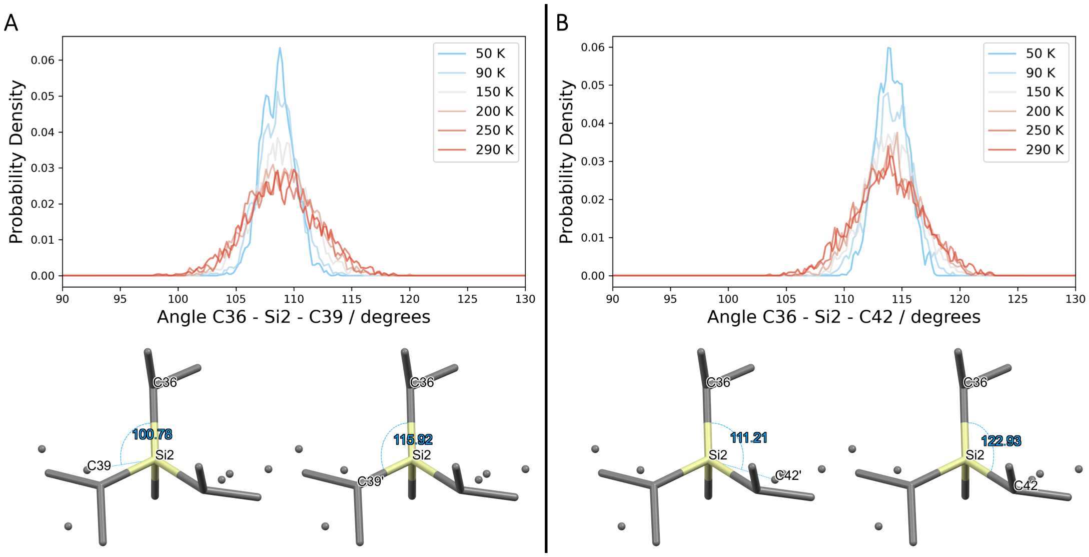

Fig. 3. The probability distributions associated with the angles C36-Si2-C39 (A) and C36-Si2-C42 (B) obtained from an MD simulation at different temperatures. On the bottom, the disorder present in the pure structure obtained at 290 K is shown with the major component depicted with solid lines and the minor one as dots.

The temperature was increased from 0 K to 290 K, leaving the system to equilibrate for 500 ps at the intermediate steps of 50, 90, 150, 200 and 250 K. The values of 90 K and 290 K were chosen to best replicate the experimental conditions at which the two patterns were taken. Between these steps, the temperature was increased by 1 K every 10 ps. This simulation was performed in the NPT ensemble using the Bussi-Donadio-Parrinello thermostat and the Parrinello-Rahman anisotropic barostat to control temperature and pressure.

We used the last 200 ps of each constant temperature step to monitor possible changes in the conformation of the molecules. With the exception of a few molecules in which a single isopropyl group rotates by 120°, no significant distortions are observed. In Fig. 3, we show how the distributions of the angles between the isopropyl groups of one of the TIPS change with temperature. In particular, we focus on angles C36 - Si2 - C39 and C36 - Si2 - C42, which at 290 K exhibit disorder with the major and minor components shown on the bottom. Noting that while well-defined peaks are present at lower temperatures, above 200 K the distributions broaden enough to include the angles experimentally observed in the minor and major components of form A. However, both TIPS show very similar distributions (two peaks around 109° and one around 115°, given by the steric interaction of methyl groups), suggesting that other factors contribute to the different behaviour of the two TIPS.

3.4. Molecular dynamics setup and simulation's results

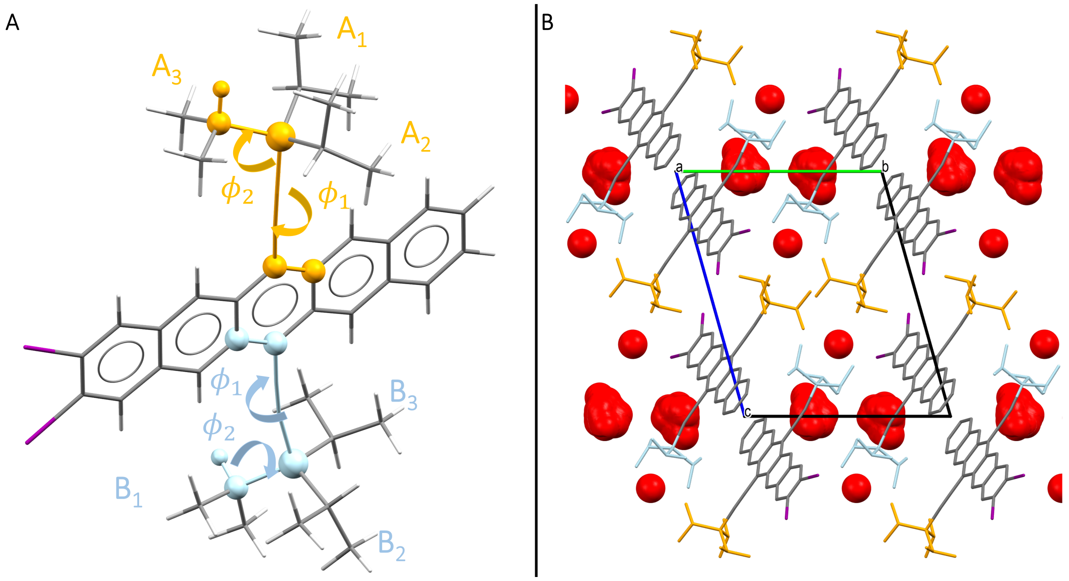

Fig. 4. (A) An example of the torsional angles ϕ_1 and ϕ_2 for groups A3 and B1. Unbiased probability distributions for the six groups are obtained from the average of the ϕ_1 and ϕ_2 angles over space (meaning over all the molecules of the simulation box) and time (meaning over all the trajectory). The ϕ_1 and ϕ_2 angles of a single molecule were also used as CVs for metadynamics simulations. (B) Voids space of the structure initially refined at 90 K using a probe of 0.95 Å and showing empty space around the B group.

In order to obtain a more accurate sampling of the atoms' positions and look for possible changes in molecules' conformation, we performed a 100 ns MD simulation using the same settings used in Sec. 3.2 but keeping the temperature at 300 K. We kept track of angles C36-Si2-C39 and C36-Si2-C42 while possible changes in the molecule conformation have been monitored by looking at the six C-C-Si-C and C-Si-C-H torsional angles, here labelled as ϕ_1 and ϕ_2, that define the conformation of the TIPS groups. While only a small broadening of distribution peaks of angles C36-Si2-C39 and C36-Si2-C42 can be seen in the 100 ns MD trajectory, the analysis of the 6 distributions of ϕ_1 and ϕ_2 shows that the two TIPS groups behave differently. These are labelled as An and Bn (shown in orange and blue, respectively, in Fig. 4B) with $n = 1, 2, 3$ to denote their three isopropyl groups in order of proximity of ϕ_1 to zero. Within this notation, the disordered isopropyl groups in structure XIFZOF (obtained at 290 K) are groups $B2$ and $B3$ while the isopropyl group that changes conformation in XIFZOF01 (at 100 K) is $A2$. An example of the torsional angles ϕ_1 and ϕ_2 for $A3$ and $B1$ is shown in Fig. 4A together with the labels of each isopropyl group.

From the 6 torsional angles distributions, $p(\phi_1, \phi_2)$, we can see that the B group shows in general more broad peaks along the ϕ_1 axis. More interestingly, different conformations arise by the end of the simulation. In particular, the B TIPS shows rotations of all three isopropyl groups and around the Silicon atom and the formation of seven distinct conformations, shown in Fig. 5. On the other hand, $A1$ and $A3$ remain stable for the entire simulation while in a few molecules the $A2$ isopropyl group rotates. However, the resulting new conformation does not correspond to the minor component of structure XIFZOF01. The reason for the different behaviour of the two groups can be seen in Fig. 4B where only one of them (B, in blue) is relatively free to rotate while the other (A, in orange) is sterically hindered. The emergence of these peaks at the end of the MD simulation suggests that much longer timescales are needed to estimate the impact of the different conformation on the room-temperature crystal. In

order to overcome the MD timescale limit and identify the probability of finding the system in each conformation, we used Well-Tempered Metadynamics (WTMD) simulations (Barducci *et al.*, 2008).

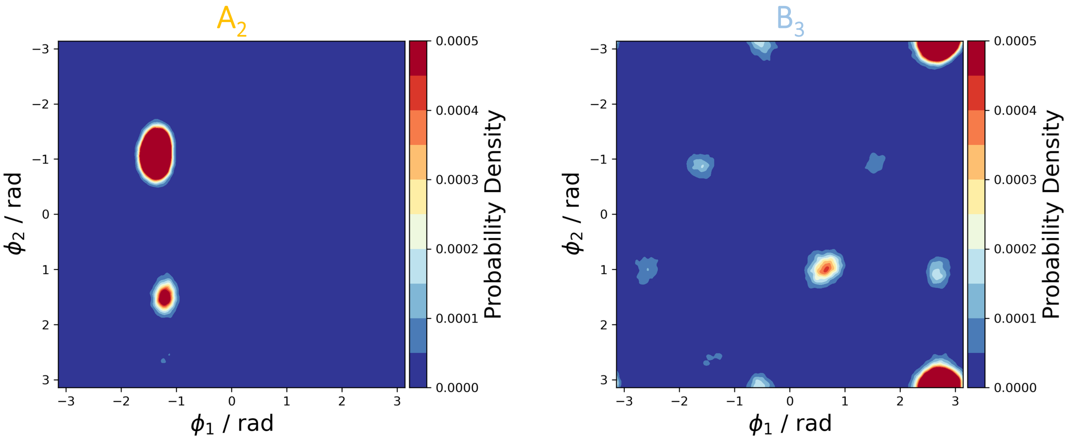

Fig. 5. Probability density obtained by sampling the ϕ_1 and ϕ_2 torsional angles of groups A2 and B3, highlighting the different behaviour of the two groups and the presence of alternative peaks.

3.5. Analysis of the free energy landscape from well-tempered metadynamics

Metadynamics introduces a repulsive history-dependent bias potential that acts in a reduced variables space, called the collective variables (CV) space, with the aim of forcing the system to escape from its free energy minima. The unbiased free energy surface (FES) in the CVs space can then be derived from the negative of the bias potential. The well-tempered variation of metadynamics decreases the deposited bias over simulation time, favouring the convergence of the FES. We set up 2 simulations using as CVs the ϕ_1 and ϕ_2 torsional angles of isopropyl groups $A1$ and $B1$ in order to study the behaviour of the two TIPS separately. The choice of the starting isopropyl groups is arbitrary as they are expected to sample all available minima during the simulation. The bias potential is updated every 500 fs with Gaussians characterised by an initial height of 1 kJ mol^{-1} and a width of 0.05 rad for each torsional angle. WTMD and the calculation of the free energy landscapes were performed using Plumed (Tribello *et al.*, 2014). A visual inspection of the final trajectory was also done to make sure that no transitions other than the molecule conformations were present. An initial 100 ns WTMD simulation was performed using the same settings as the MD simulation but this showed that larger timescales were needed to reach convergence of the FES. In order to speed up the process, the simulation was repeated using Energy and Pressure dispersion corrections instead of LJ PME, allowing us to run a 1 μs simulation for each of the two TIPS in a reasonable time.

From the FES in the CVs space, $G(\phi_1, \phi_2)$, we can calculate the related equilibrium probability distribution, $P(\phi_1, \phi_2)$, as:

$$P(\phi_1, \phi_2) = \frac{e^{-\beta G(\phi_1,\phi_2)}}{\int_\Omega e^{-\beta G(\phi_1,\phi_2)} d\phi_1 d\phi_2} \tag{1}$$

in which Ω refers to the entire configuration space. The equilibrium probability of any conformer i, P_i, can be calculated by integrating $P(\phi_1, \phi_2)$ over the subdomain Ω_i:

$$P_i = \int_{\Omega_i} P(\phi_1, \phi_2) d\phi_1 d\phi_2 \tag{2}$$

The delimiters of each subdomain Ω_i are shown in Fig. 6C and 7C. These are labelled as $A1$, $A2$, $A3$ and $B1$, $B2$, $B3$ when corresponding to one of the initial conformations, while other minima labels

are obtained by adding lowercase letters *a, b, c* to the closest one in ϕ_1. Finally, from equilibrium probabilities, we can calculate the free energy difference, $\Delta G_{i,j}$, between each conformer i and j:

$$\Delta G_{i,j} = -\beta^{-1} \log \frac{P_j}{P_i} \tag{3}$$

The values of $\Delta G_{i,j}$ were used to monitor the convergence of WTMD simulation. Plots in Fig. 6B and 7B show $\Delta G_{i,j}$ over simulation time associated with conformer $A1$ and $B1$, respectively. In both cases, no variation higher than 5 kJ mol^{-1} every 25 ns was observed after 700 ns.

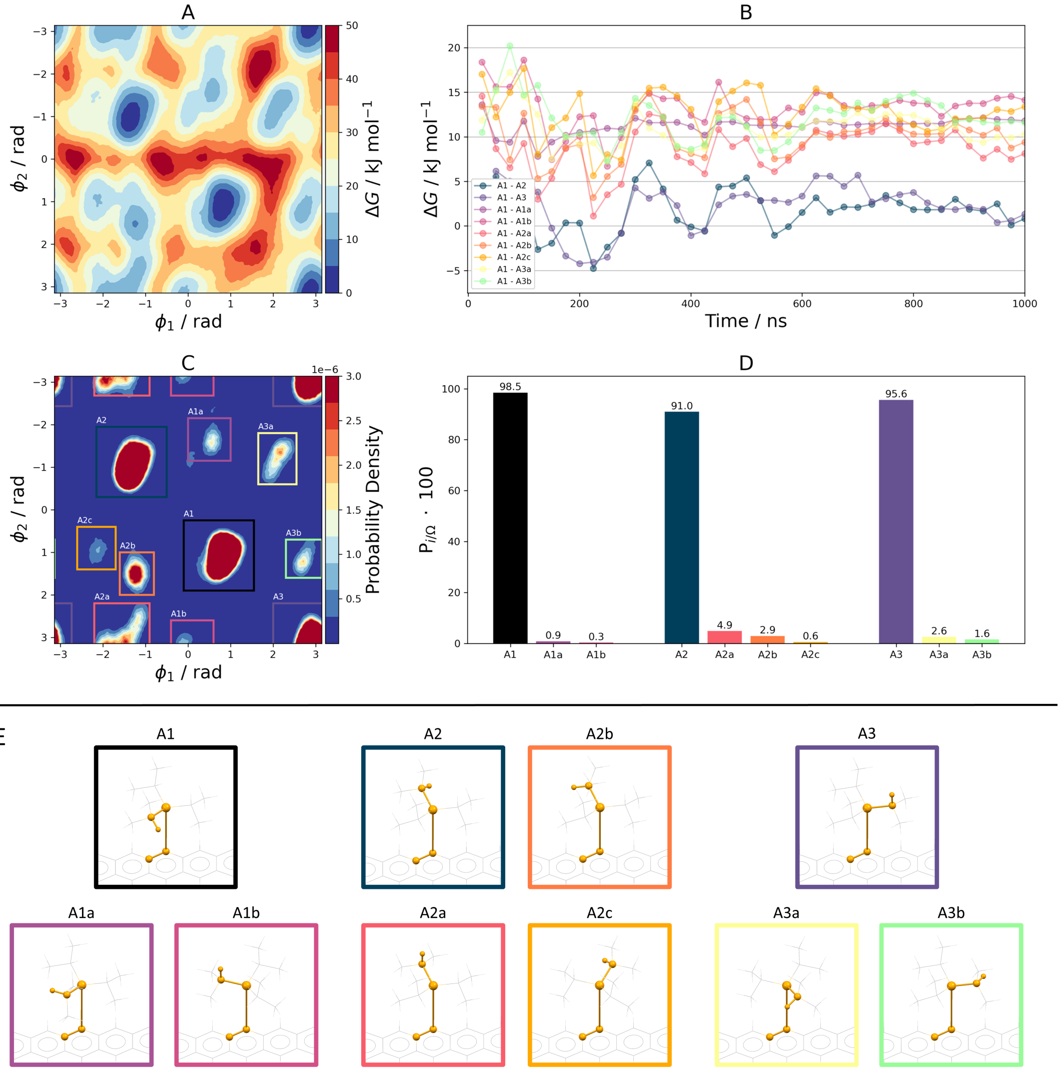

Fig. 6. (A) The free energy surface corresponding to TIPS A obtained by biasing the ϕ_1 and ϕ_2 torsional angles shown in Fig. 4 and (C) the probability distributions derived from the free energy surfaces with the bounding box used to calculate the probability, P_i, of each basin as shown in plot (D). Plot (B) shows instead the free energy difference, $\Delta G_{i,j}$, between basin A1 and all the others. These values have been used to monitor the convergence of the WTMD simulation. (E) The isopropyl conformation relative to each basin in (C) and (D).

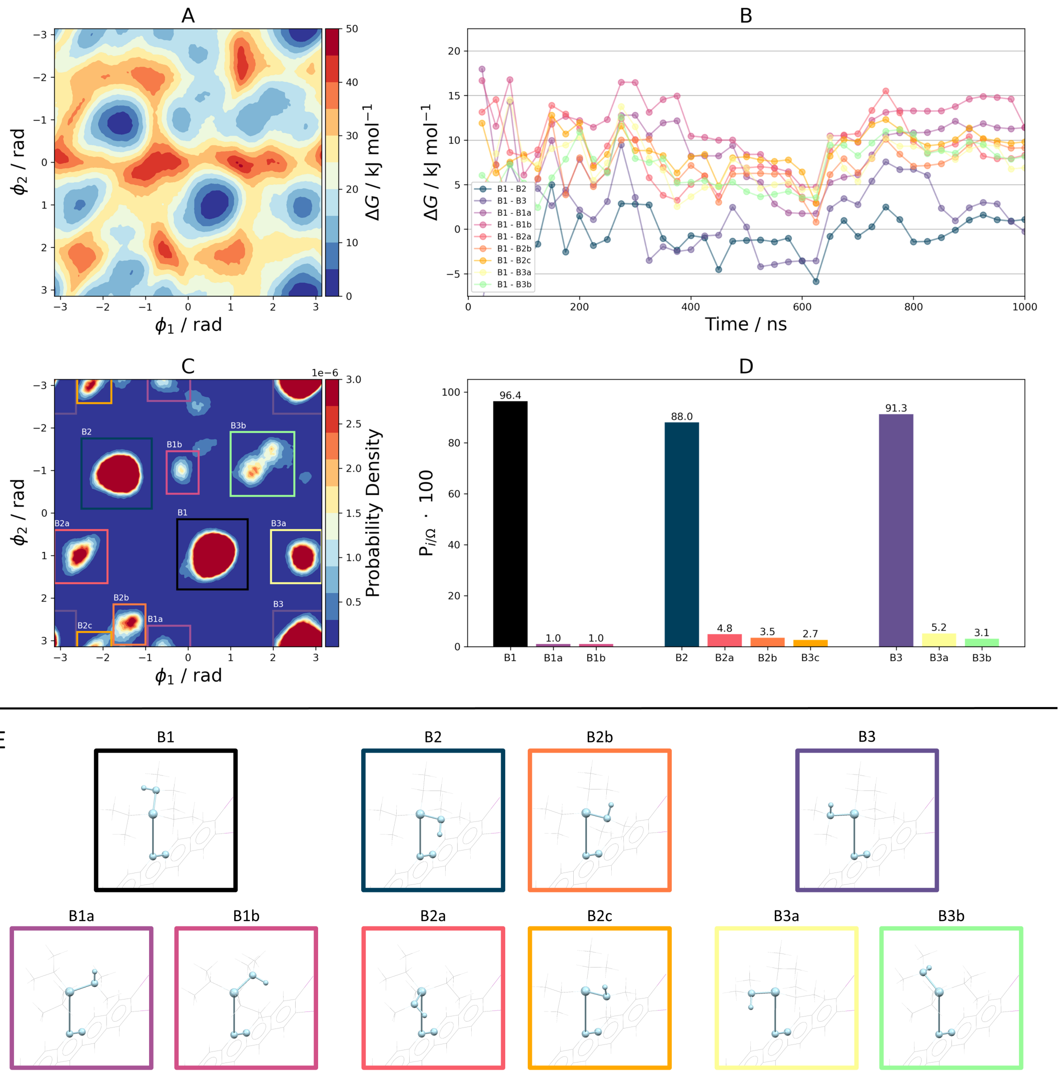

Fig. 7. (A) The free energy surface corresponding to TIPS B obtained by biasing the ϕ_1 and ϕ_2 torsional angles shown in Fig. 4 and (C) the probability distributions derived from the free energy surfaces with the bounding box used to calculate the probability, P_i, of each basin as shown in plot (D). Plot (B) shows instead the free energy difference, $\Delta G_{i,j}$, between basin B1 and all the others. These values have been used to monitor the convergence of the WTMD simulation. (E) The isopropyl conformation relative to each basin in (C) and (D).

In Fig. 6A and 7A, we show the FES of TIPS A and B, respectively. The shape of the three main conformations is again different between the two groups with B having broader free energy basins, suggesting the group can rotate about 20° at room temperature. In both cases, a series of local minima are present in the FES. However, transitions from one of the three initial conformations to any other minima exhibit energy barriers of at least 25 kJ mol^{-1}, suggesting no dynamic disorder involving a conformational change is present. In order to find the probability of one of the isopropyl groups being in a disordered state, the probability distribution $P(\phi_1, \phi_2)$ was divided into three regions over ϕ_1, each one including one of the three main conformations. The normalised probability distribution for each conformation in each region is shown in Fig. 6D for TIPS A and Fig. 7D for TIPS B. $A1$ and $A3$

from TIPS A and $B1$ from TIPS B are particularly stable with equilibrium probabilities being over 95%. In the other three cases ($A2$, $B2$, $B3$), the alternative conformations have a greater impact with around a 10% probability of not finding the system in one of the main conformations. The conformational disorder of isopropyl $A2$ observed in structure XIFZOF01 corresponds to basin $A2a$ which has the highest probability (4.9%) among the alternative conformations of TIPS A. Other populated states with probabilities around 5% are $B3a$ and $B2a$ which, however, were not detected.

WTMD simulations indicate a dynamic disorder involving the rotation of the TIPS groups, more evident for the B TIPS, and conformational static disorder given by a myriad of free energy minima, here analysed by looking at the ϕ_1 and ϕ_2 torsional angles. The presence of these two concurrent phenomena suggests difficulties in refining the structure even if the low probabilities associated with alternative conformations make it difficult to experimentally detect them. Within this analysis, the disorder in experimental structure XIFZOF01 can be related to the rotation of the TIPS and the presence of different conformations accessible at room temperature that result in static disorder when the structure is cooled down.

4. Short-range similarity predicted structures of XXIX

COMPACK comparisons demonstrated a large sensitivity to the number of molecules in the comparison cluster, which initially led to conflicting conclusions regarding the number of matching structures. Comparisons of XXIX Form A with predicted structures applying a 20 molecule cluster with 35%/35° distance/angle tolerances resulted in matches from nine groups (5, 6, 10, 11, 13, 16, 20, 21, 27), see tables 17 and 18.

Table 17. *Results of comparisons of predicted structures from participating groups with the experimental Form A of XXIX, applying a 20 molecule cluster with 35%/35° distance/angle tolerances. Results with the highest number of matched molecules are reported for each group.*

Group	CIF name	Number of matched molecules (/20)	RMSD (Å)
1	XXIX_0536.cif	14	0.702268
3	xxix-ca00_e0-s7801-z2d-dma-180.cif	14	0.597286
5	XXIX_c1x2-QR-14-38386-3.cif	20	0.195299
6	data_vaneijck-XXIX.0027.cif	20	0.159336
10	data_1_z3_st_YSxRB1Za6zN2UQXt.cif	20	0.183026
11	data_001.cif	20	0.440589
12	JJOSE_XXIX_738.cif	5	4.083
13	data_29_5.cif	20	0.322061
16	MI_0001_dft_72ba6fef.cif	20	0.388469
18	data_162.cif	15	0.912183
19	XXIX-1-OpenEye_structure_78.cif	15	2.832004
20	structure_1.cif	20	0.096875
21	XXIX-0301.cif	20	0.315199
22	data_1305.cif	10	1.259762
23	sorted-1500-n1441.cif	14	0.815571
24	BTXXIX_dfA3_A3.cif	14	0.209756
25	XXIX_558.cif	14	0.295837
27	data_937.cif	20	0.185579
28	data_112-SG-14.cif	14	0.598335

Table 18: *Visual comparisons of predicted structures from participating groups with the experimental Form A of XXIX, applying a 20 molecule cluster with 35%/35° distance/angle tolerances. Structures shown are the closest matches identified from each group. Matched molecules are shown in green, mismatched molecules shown in red.*

Group	CIF name	Visualisation of COMPACK comparison with XXIX Form A
1	XXIX_0536.cif	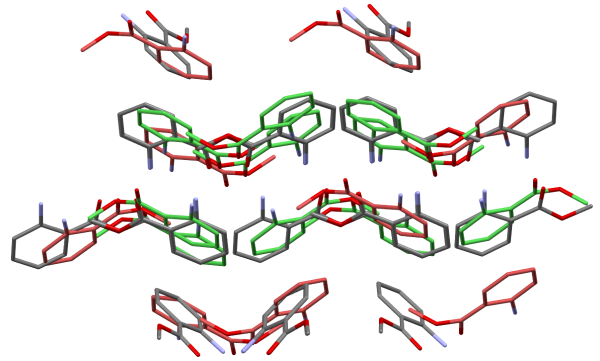
3	xxix-ca00_e0-s7801-z2d-dma-180.cif	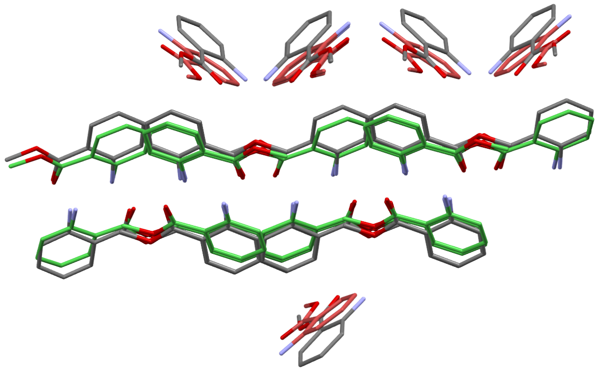
5	XXIX_c1x2-QR-14-38386-3.cif	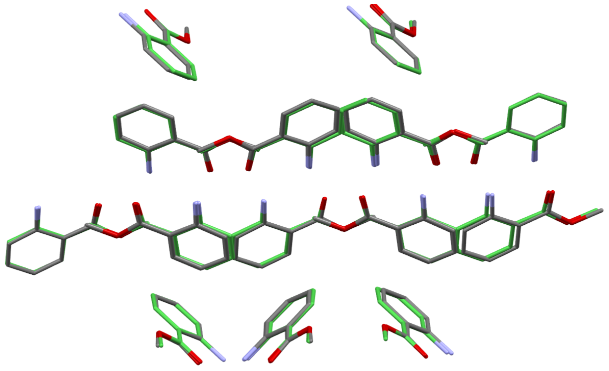

6	data_vaneijck-XXIX.0027.cif	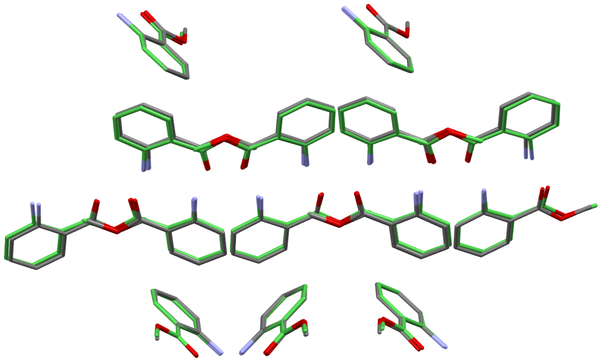
10	data_1_z3_st_YSxRB-1Za6zN2UQXt.cif	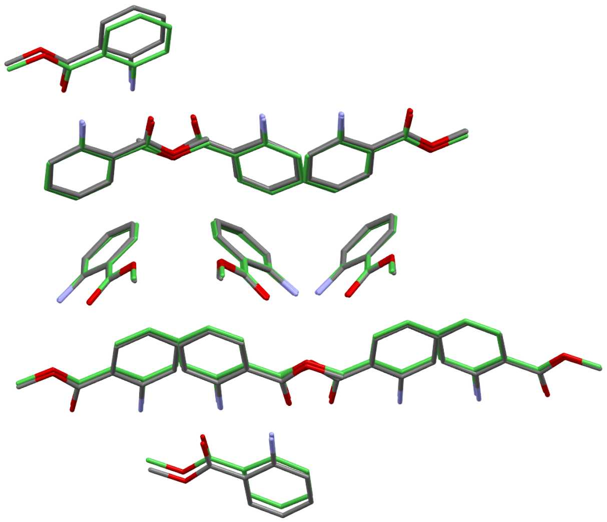
11	data_001.cif	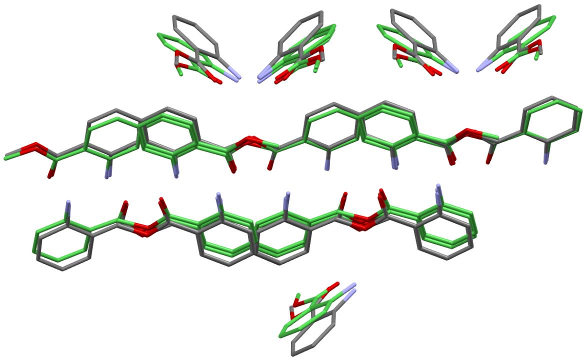

12	JJOSE_XXIX_738.cif	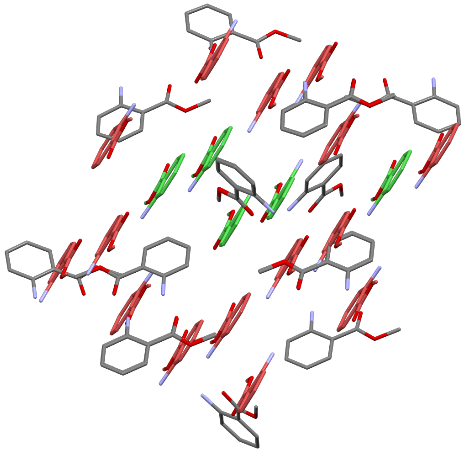
13	data_29_5.cif	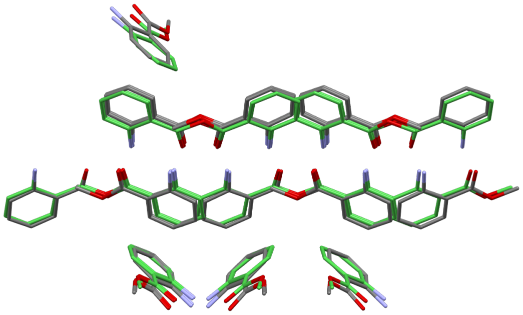
16	MI_0001_dft_72ba6-fef.cif	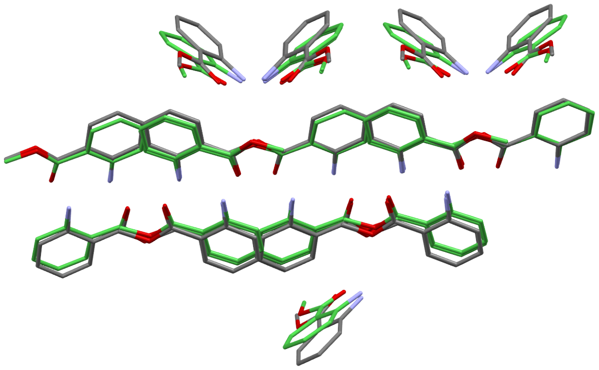
18	data_162.cif	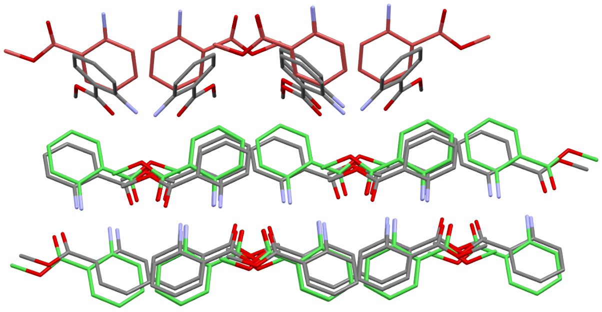

19	XXIX-1-OpenEye_structure-_78.cif	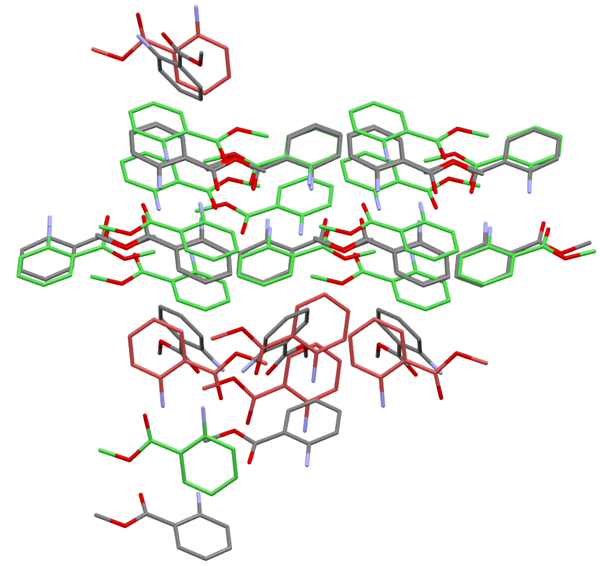
20	structure_1.cif	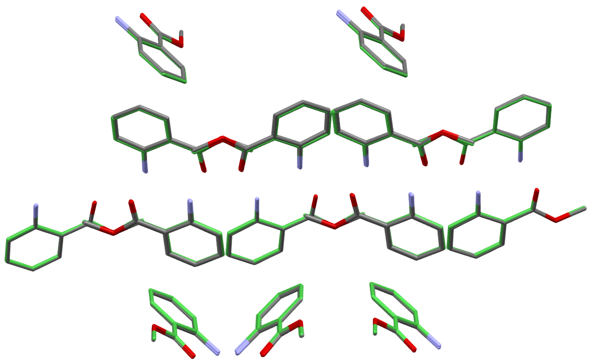
21	XXIX-0301.cif	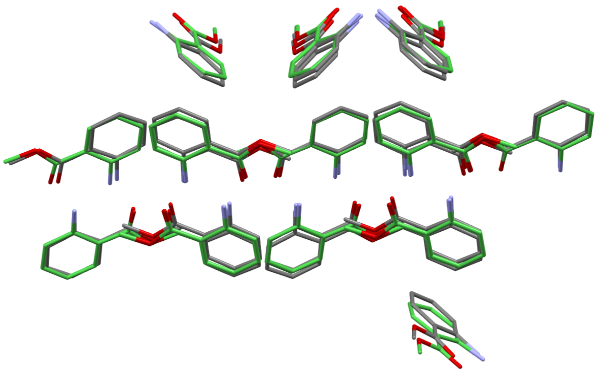

22 data_1305.cif

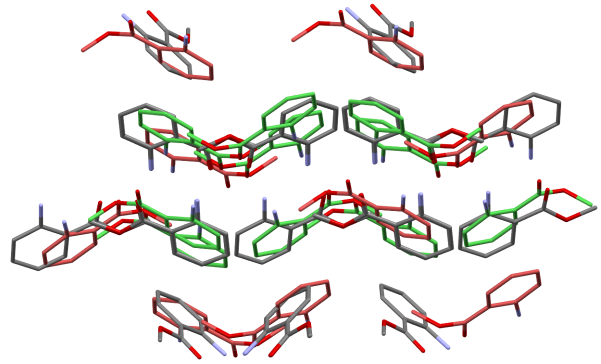

23 sorted-1500-n1441.cif

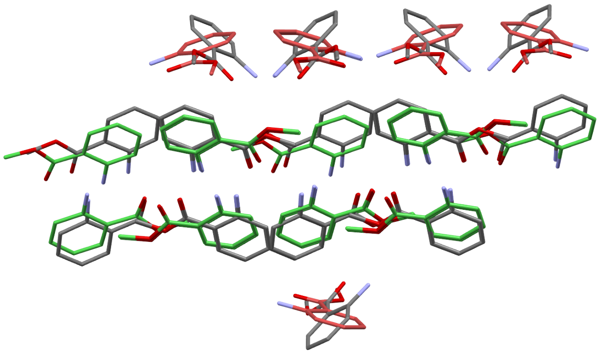

24 BTXXIX_dfA3_A3.cif

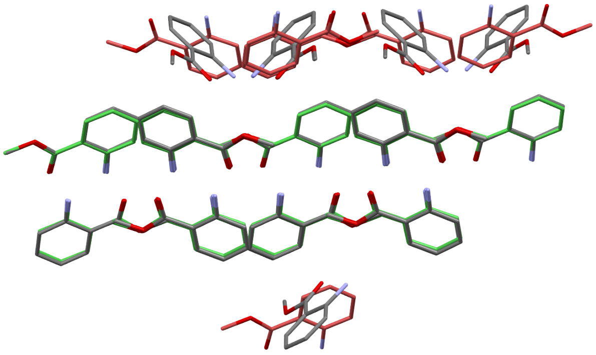

25 XXIX_558.cif

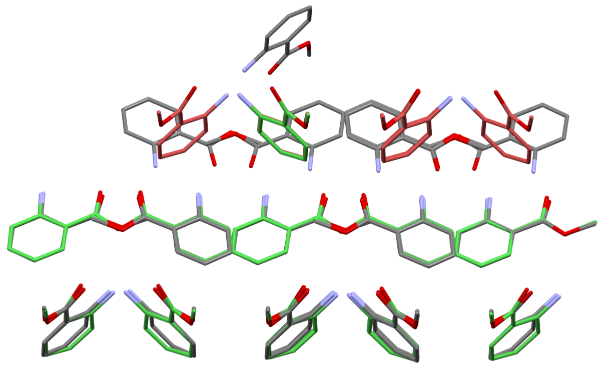

27	data_937.cif	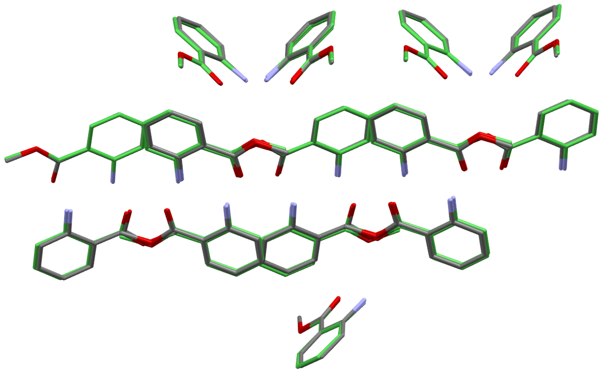
28	data_112-SG-14.cif	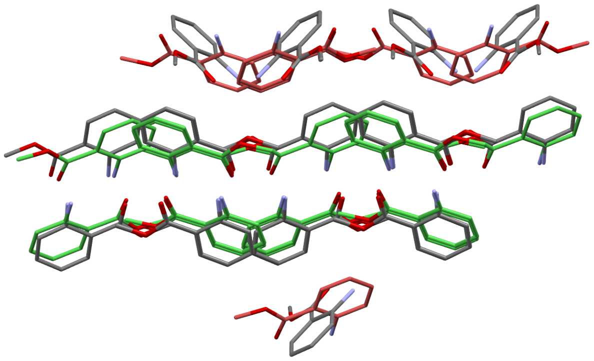

Table 19. *The number of $Z' = 3$ structures in the list of 1500 and list of 10 ranked structures submitted by each group.*

Group	Number of $Z' = 3$ structures in list of 1500	Number of $Z' = 3$ structures in list of 10
1	0	6
3	0	0
5	2	0
6	0	0
10	851	8
11	0	0
12	0	0
13	0	0
16	7	0
18	0	0
19	0	0
20	982	3
21	0	0
22	0	0
23	178	5
24	0	0
25	0	0
27	184	0
28	0	0

5. Crystal Structure Similarity

5.1. Introduction

A crystal structure similarity search was conducted on >100 000 computationally generated structures divided over 6 target molecules and produced by 28 research groups for the 7th CSP Blind Test.

Traditionally used analysis tools, such as the molecule overlay approach COMPACK (Chisholm & Motherwell, 2005) available as Crystal Packing Similarity in Mercury and the CSD Python API (Groom *et al.*, 2016), have been employed together with the computationally efficient Average Minimum Distance (AMD) and Pointwise Distance Distributions (PDD) methods, which allowed full comparison of the sets in a reasonable time. Apart from the identification of theoretical-experimental structure matches, we later explored the agreement between different structure generation methods by comparing the generated structures from each set. The latter was made possible by producing distance matrices using the PDD approach.

5.2. Comparisons of the AMD and PDD approaches to the Crystal Packing Similarity tool

The PDD descriptor consists of an $N \times k$ weighted matrix in which each one of the N rows corresponds to an ordered list of distances between an atom in the unit cell to the k closest neighbours. Identical rows are then collapsed together with weights assigned based on the number of occurrences and the different matrices are then compared using the Earth Mover's Distance.

The AMD descriptor is instead a vector obtained from the column average of the PDD matrix (Widdowson *et al.*, 2021; Widdowson & Kurlin, 2022). An advantage of both PDD and AMD metrics is that the use of atom-atom distances makes the comparison independent of the choice of the unit cell.

Both these metrics are however sensitive to the expansion or contraction of the cell, making it difficult to compare structures generated at 0 K with those that include temperature effects. For this reason, an isotropic expansion of the cell based on the ratio between the two volumes is applied when comparing structures.
AMD and PDD were initially used as an alternative approach to COMPACK to identify matches between the CSP-generated structures of the blind test and the experimental forms. This was of particular importance for the identification of theoretical structures corresponding to XXVII form A in which PDD showed a series of matches that have been ignored by COMPACK.

After confirmation by visual inspection, this led to the identification of the COMPACK limitation described in the main paper and its correction. Subsequent analyses were conducted with the PXRD similarity tool (de Gelder *et al.*, 2001) available in Mercury (MacRae *et al.*, 2020) and later on the variable-cell powder difference (Mayo *et al.*, 2022) (VC-PWDF) available in the *critic2* (Otero-de-la-Roza *et al.*, 2014) software, both confirming the presence of highly similar structures.

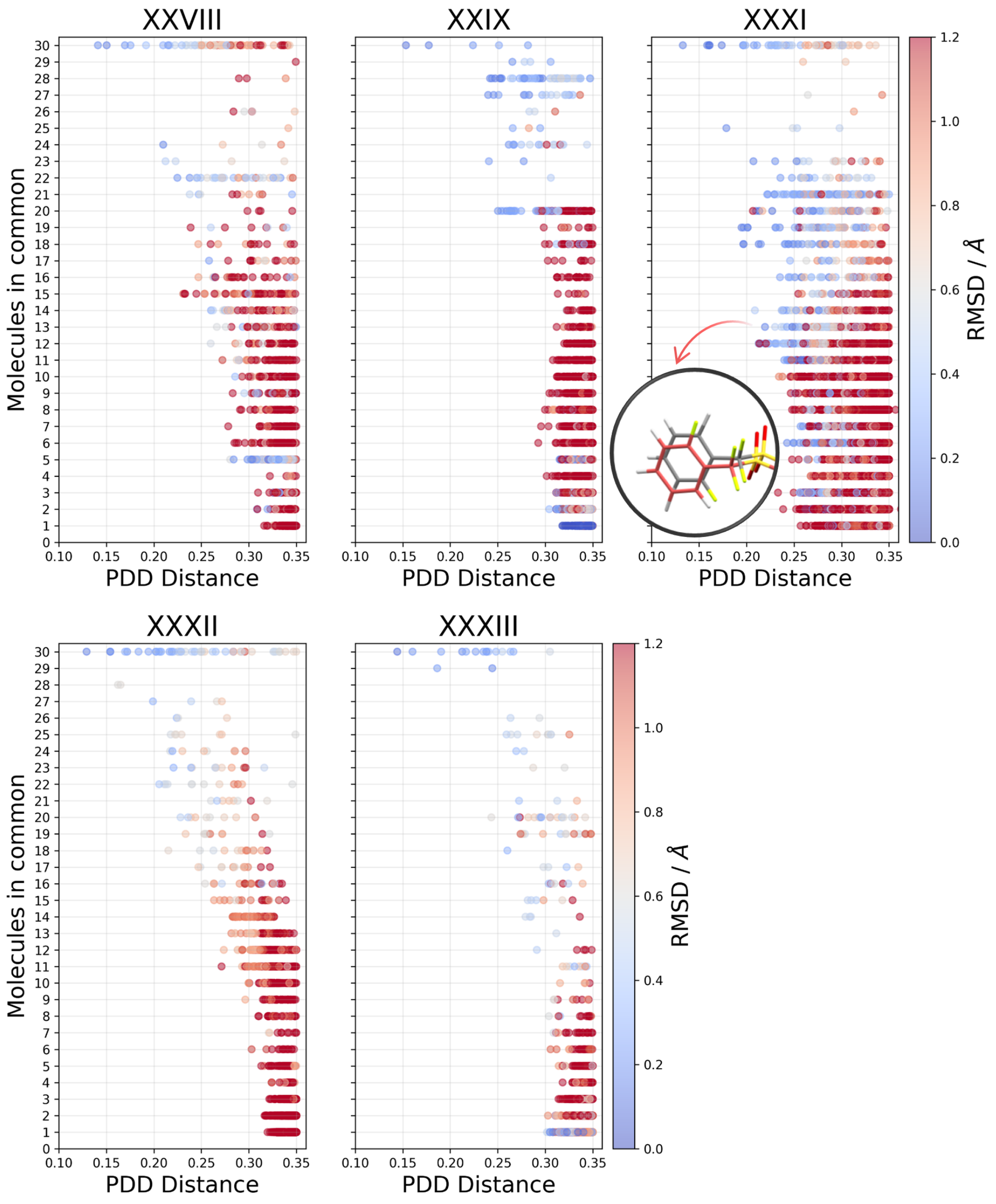

Fig. 8. Comparison between the PDD distance metric, in the abscissa, and the Crystal Packing similarity tool showing the number of molecules in common in the ordinate and the RMSD in different colours according to the colour bar on the right.

More in general, we proceeded to compare PDD distances obtained using $k = 100$ with COMPACK results using a 30-molecule shell and 0.30/30 position/angle tolerances. Among the perfect matches, *i.e.* 30/30 molecule overlap and $RMSD_{30} < 1.0$ Å, the highest PDD distance found was 0.36 Å. This value reduces to 0.32 Å when only the best matches, *i.e.* the one with the lowest $RMSD_{30}$ from each group, are considered.
In order to look at the correlation between PDD and COMPACK even among the partial matches, the search was repeated for all targets except XXVII, for which only perfect matches were calculated. To speed up the process, we focused on those structures with PDD $<$ 0.35 Å. As the (much faster

to calculate) AMD distance forms a lower bound of the PDD distance (Widdowson & Kurlin, 2022), we used it first to filter all those comparisons above the cutoff. The PDD distance is then calculated on the remaining ones and if the resulting distance remains below the cutoff, the Crystal Packing Similarity tool is used.
In Fig. 8, we show the comparison of the PDD and COMPACK metrics. In most cases, the PDD distance correlates well with both the number of molecules in common and the calculated RMSD from the COMPACK tool. In particular, PDD well distinguishes perfect matches as long as they have a low $RMSD_{30}$, with most of the comparisons having $RMSD_{30} < 0.5$ Å being below 0.25 Å PDD distance. On the other hand, as the $RMSD_{30}$ increases, false positives start to emerge.
The low level of agreement in molecule XXXI is caused by a lack of chemical information in the metric. This molecule has a series of partial matches with PDD distances below 0.25 Å. Most of these structures exhibit a 180° rotation of the fluorobenzene, which slightly affects the descriptor and results in an overestimation of the similarity between structures that have similar packing but different molecular conformation.

5.3. Assessment of similarity among submitted sets

The large number of structures imposes the use of a computationally efficient similarity metric. The PDD metric was used to compare the CSP-generated sets of each group and generate a $N_x \times N_y$ distance matrix, with N_x, N_y being the number of structures for each target. In this study, we performed two comparisons, one involving the first 100-ranked structures from each group ($N_x = N_y = 100n$, with n being the number of groups involved), and the second comparing the first 100-ranked structures from one group with the entire set of the other ($N_x = 1500n$, $N_y = 100n$). These two approaches are labelled as *100 vs 100* and *100 vs all*, respectively. This matrix was then collapsed along the x-axis by taking the minimum PDD distance from each group. If it was below 0.225 Å, the structures were considered to be the same. Looking at the plots in Fig. 8, this value was found to limit the inclusion of comparisons of poorly overlapping structures. By counting the matches, the matrix was further collapsed along the y-axis resulting in a $n \times n$ matrix, with n being the number of groups involved. These numbers were then rescaled to obtain the % of overlapping structures. The resulting heatmaps in Fig. 9 and 10 show the percentage of structures of the group on the x-axis that are present in the set of the group on the y-axis. In these plots, the upper and lower triangular matrices do not necessarily coincide. This is due to the possible different sizes of the structure sets (a few groups submitted less than 100 structures) and the presence of multiple structures in one set matching a single structure from another one. Groups 10 and 20 stand out for landscape similarity with an average of 40% structures in common across the six compounds in *100 vs 100* comparisons and 75% in *100 vs all*. The landscapes overlap for each compound are shown in Tab. 20. This includes also a COMPACK similarity analysis (limited to the first 100-ranked structures). The overestimation of similarity for compound XXXI shown in Fig. 8 is confirmed in this table too.

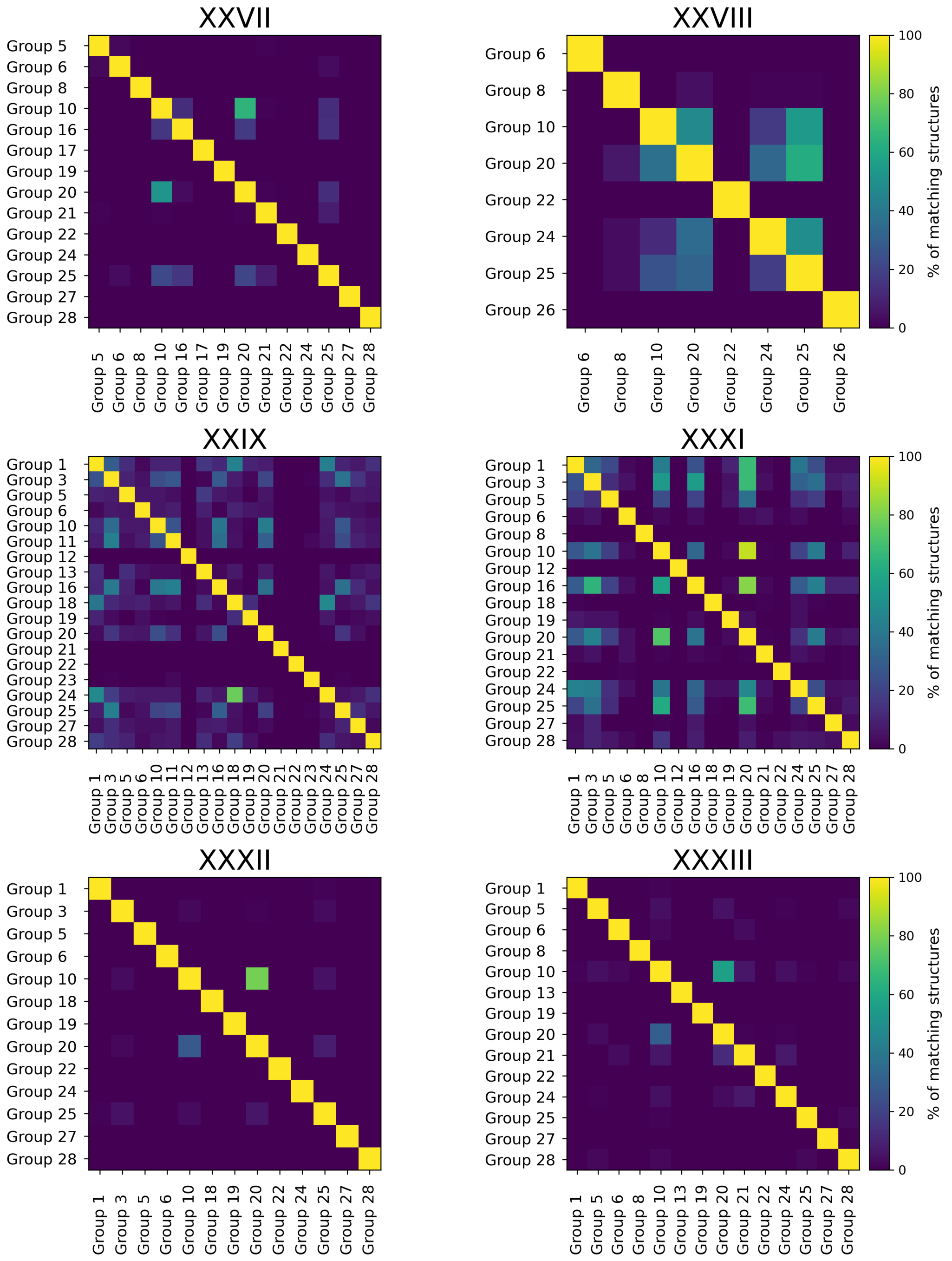

Fig. 9. The *100 vs 100* similarity heatmaps that were obtained from the procedure described in Sec. 5.3. These show the percentage of structures from the group on the x-axis that are present in the set of the group on the y-axis.

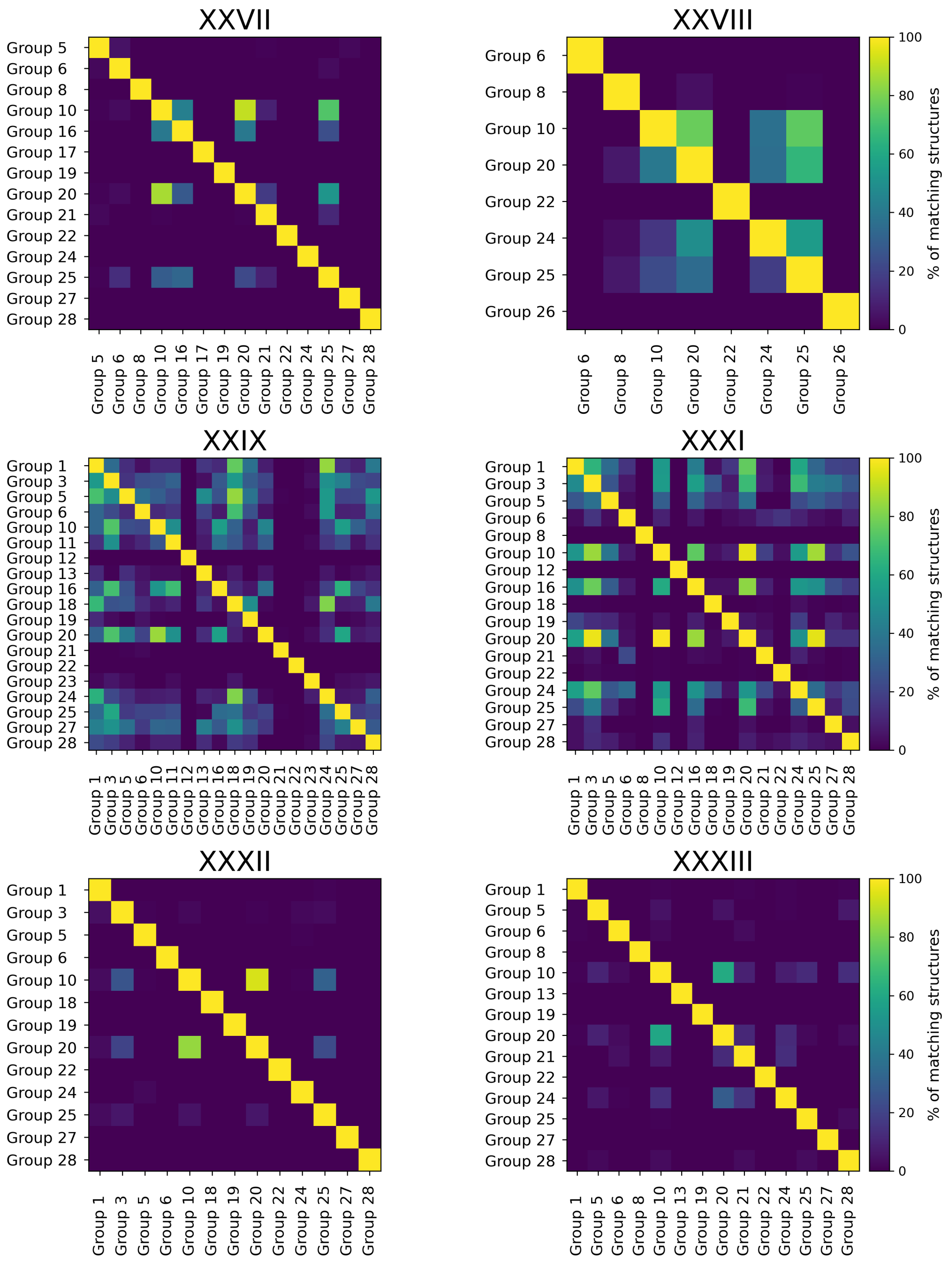

Fig. 10. The *100 vs all* similarity heatmaps that were obtained from the procedure described in Sec. 5.3. These show the percentage of structures from the group on the x-axis that are present in the set of the group on the y-axis.

Table 20. *The number of structures of the first group that are present in the set of the second group according to the PDD and COMPACK metrics, considering only the first 100 ranked structures (* 100 vs 100*). In parenthesis, the values of the* 100 vs all *comparisons. A 0.225 Å PDD distance cutoff was used to assess a match between the two structures while a 30-molecule overlap, using 35%/35° distance/angle tolerances, was used for COMPACK.*

	Structures of Group 10 in set of Group 20		Structures of Group 20 in set of Group 10	
Compound	PDD	COMPACK	PDD	COMPACK
XXVII	52 (87)	32	65 (91)	63
XXVIII	37 (40)	40	47 (77)	40
XXIX	24 (85)	25	42 (45)	43
XXXI	73 (99)	53	91 (96)	67
XXXII	28 (84)	22	79 (94)	59
XXXIII	30 (59)	30	57 (61)	60

6. Previous Blind Tests Target Compounds

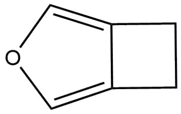

(I)

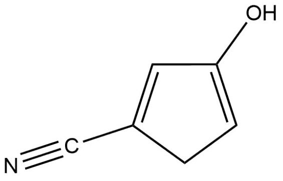

(II)

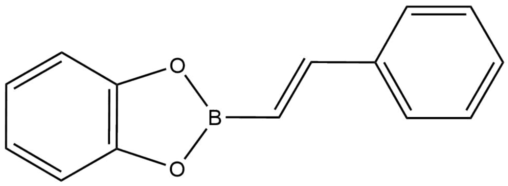

(III)

Fig. 11. Two-dimensional chemical structures of target compounds (I – III) from the first blind test of CSP methods.

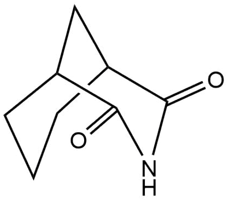

(IV)

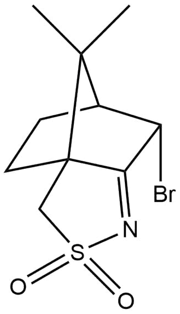

(V)

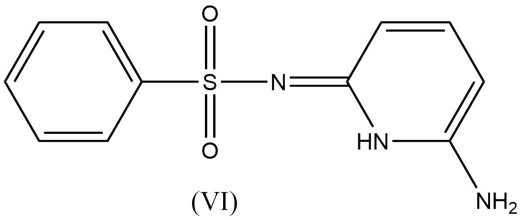

(VI)

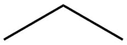

(VII)

Fig. 12. Two-dimensional chemical structures of target compounds (IV – VII) from the second blind test of CSP methods.

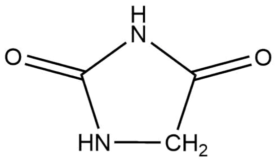

(VIII)

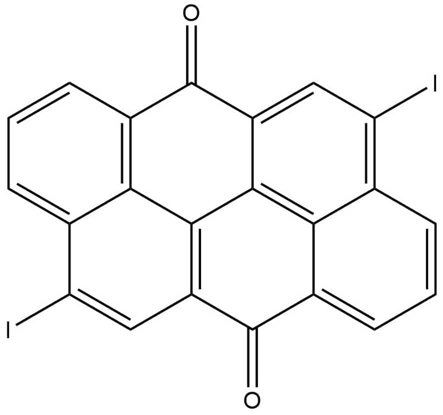

(IX)

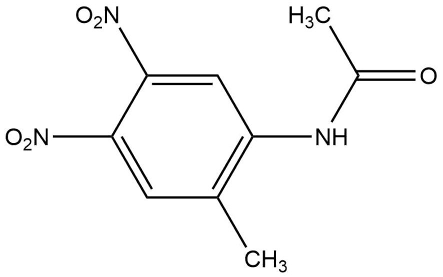

(X)

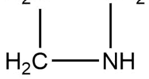

(XI)

Fig. 13. Two-dimensional chemical structures of target compounds (VIII – XI) from the third blind test of CSP methods.

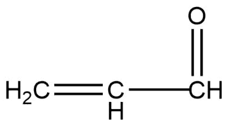

(XII)

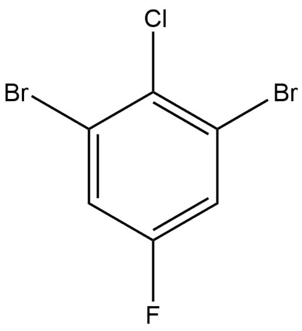

(XIII)

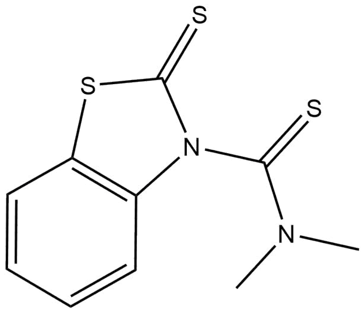

(XIV)

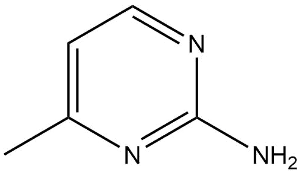

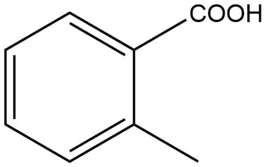

(XV)

Fig. 14. Two-dimensional chemical structures of target compounds (XII – XV) from the fourth blind test of CSP methods.

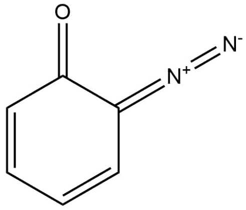

(XVI)

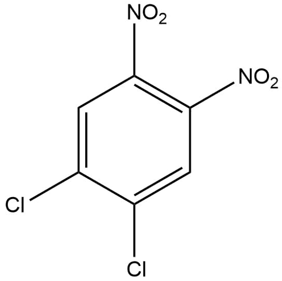

(XVII)

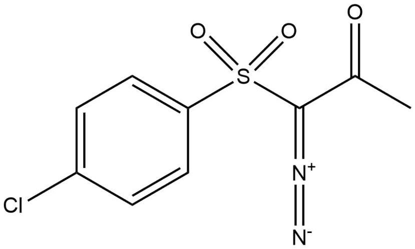

(XVIII)

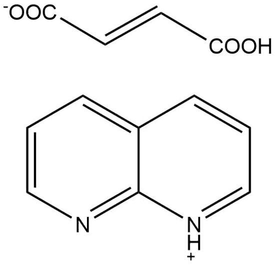

(XIX)

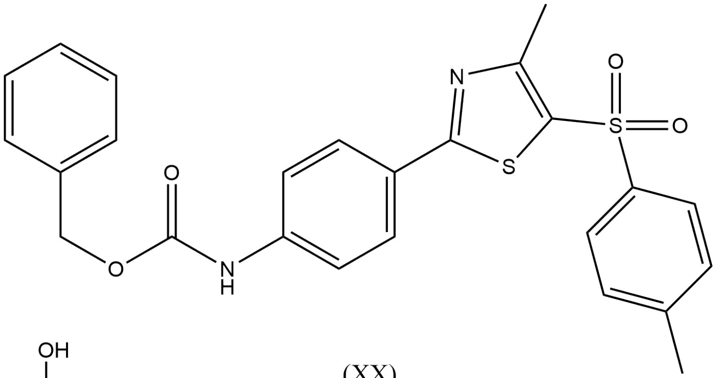

(XX)

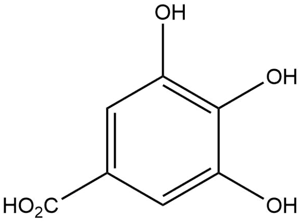

(XXI)

Fig. 15. Two-dimensional chemical structures of target compounds (XVI – XXI) from the fifth blind test of CSP methods.

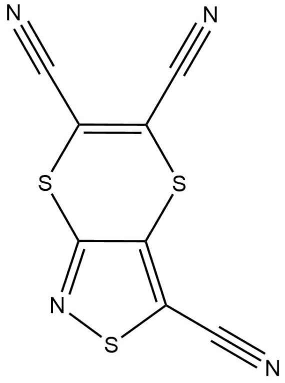

(XXII)

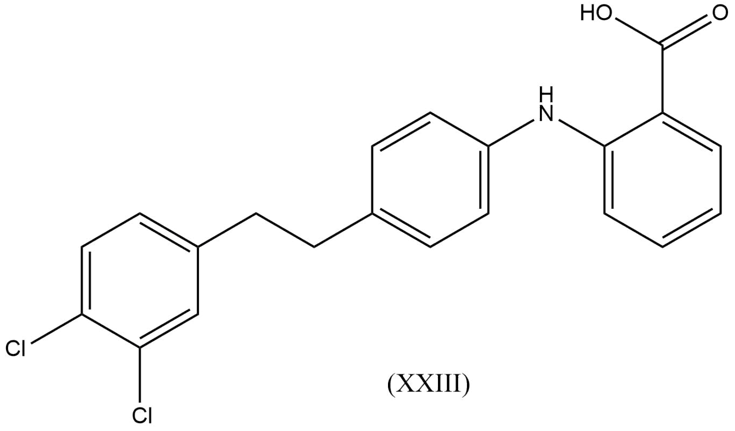

(XXIII)

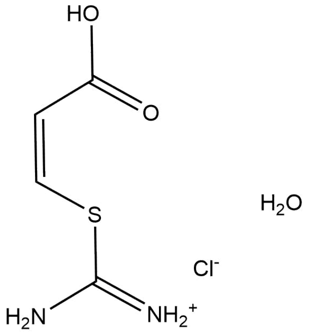

(XXIV)

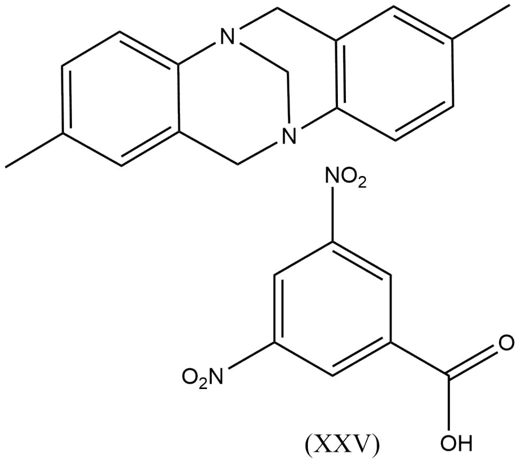

(XXV)

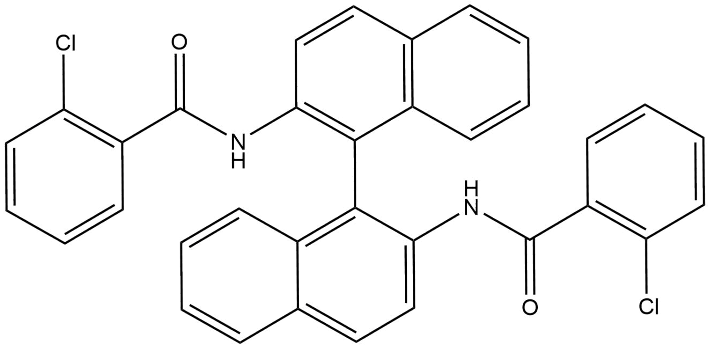

(XXVI)

Fig. 16. Two-dimensional chemical structures of target compounds (XXII – XXVI) from the sixth blind test of CSP methods.

7. DOI links for submitted sets of structures

Table 21. *DOI links for the submitted structure sets from each group for target XXVII.*

Group	DOI
5	https://doi.org/10.5517/ccdc.7bt.g5.xxvii.s1
6	https://doi.org/10.5517/ccdc.7bt.g6.xxvii.s1
8	https://doi.org/10.5517/ccdc.7bt.g8.xxvii.s1
10	https://doi.org/10.5517/ccdc.7bt.g10.xxvii.s1
16	https://doi.org/10.5517/ccdc.7bt.g16.xxvii.s1
17	https://doi.org/10.5517/ccdc.7bt.g17.xxvii.s1
19	https://doi.org/10.5517/ccdc.7bt.g19.xxvii.s1
20	https://doi.org/10.5517/ccdc.7bt.g20.xxvii.s1
21	https://doi.org/10.5517/ccdc.7bt.g21.xxvii.s1
22	https://doi.org/10.5517/ccdc.7bt.g22.xxvii.s1
24	https://doi.org/10.5517/ccdc.7bt.g24.xxvii.s1
25	https://doi.org/10.5517/ccdc.7bt.g25.xxvii.s1
27	https://doi.org/10.5517/ccdc.7bt.g27.xxvii.s1
28	https://doi.org/10.5517/ccdc.7bt.g28.xxvii.s1

Table 22. *DOI links for the submitted structure sets from each group for target XXVIII.*

Group	DOI
6	https://doi.org/10.5517/ccdc.7bt.g6.xxviii.s1
8	https://doi.org/10.5517/ccdc.7bt.g8.xxviii.s1
10	https://doi.org/10.5517/ccdc.7bt.g10.xxviii.s1
20	https://doi.org/10.5517/ccdc.7bt.g20.xxviii.s1
22	https://doi.org/10.5517/ccdc.7bt.g22.xxviii.s1
24	https://doi.org/10.5517/ccdc.7bt.g24.xxviii.s1
25	https://doi.org/10.5517/ccdc.7bt.g25.xxviii.s1
27	https://doi.org/10.5517/ccdc.7bt.g27.xxviii.s1

Table 23. *DOI links for the submitted structure sets from each group for target XXIX.*

Group	DOI
1	https://doi.org/10.5517/ccdc.7bt.g1.xxix.s1<br>https://doi.org/10.5517/ccdc.7bt.g1.xxix.s2
3	https://doi.org/10.5517/ccdc.7bt.g3.xxix.s1<br>https://doi.org/10.5517/ccdc.7bt.g3.xxix.s2
5	https://doi.org/10.5517/ccdc.7bt.g5.xxix.s1<br>https://doi.org/10.5517/ccdc.7bt.g5.xxix.s2
6	https://doi.org/10.5517/ccdc.7bt.g6.xxix.s1<br>https://doi.org/10.5517/ccdc.7bt.g6.xxix.s2
10	https://doi.org/10.5517/ccdc.7bt.g10.xxix.s1<br>https://doi.org/10.5517/ccdc.7bt.g10.xxix.s2
11	https://doi.org/10.5517/ccdc.7bt.g11.xxix.s1<br>https://doi.org/10.5517/ccdc.7bt.g11.xxix.s2
12	https://doi.org/10.5517/ccdc.7bt.g12.xxix.s1<br>https://doi.org/10.5517/ccdc.7bt.g12.xxix.s2
13	https://doi.org/10.5517/ccdc.7bt.g13.xxix.s1<br>https://doi.org/10.5517/ccdc.7bt.g13.xxix.s2
16	https://doi.org/10.5517/ccdc.7bt.g16.xxix.s1<br>https://doi.org/10.5517/ccdc.7bt.g16.xxix.s2
18	https://doi.org/10.5517/ccdc.7bt.g18.xxix.s1<br>https://doi.org/10.5517/ccdc.7bt.g18.xxix.s2
19	https://doi.org/10.5517/ccdc.7bt.g19.xxix.s1<br>https://doi.org/10.5517/ccdc.7bt.g19.xxix.s2
20	https://doi.org/10.5517/ccdc.7bt.g20.xxix.s1<br>https://doi.org/10.5517/ccdc.7bt.g20.xxix.s2
21	https://doi.org/10.5517/ccdc.7bt.g21.xxix.s1<br>https://doi.org/10.5517/ccdc.7bt.g21.xxix.s2
22	https://doi.org/10.5517/ccdc.7bt.g22.xxix.s1<br>https://doi.org/10.5517/ccdc.7bt.g22.xxix.s2
23	https://doi.org/10.5517/ccdc.7bt.g23.xxix.s1<br>https://doi.org/10.5517/ccdc.7bt.g23.xxix.s2
24	https://doi.org/10.5517/ccdc.7bt.g24.xxix.s1<br>https://doi.org/10.5517/ccdc.7bt.g24.xxix.s2
25	https://doi.org/10.5517/ccdc.7bt.g25.xxix.s1<br>https://doi.org/10.5517/ccdc.7bt.g25.xxix.s2
27	https://doi.org/10.5517/ccdc.7bt.g27.xxix.s1<br>https://doi.org/10.5517/ccdc.7bt.g27.xxix.s2
28	https://doi.org/10.5517/ccdc.7bt.g28.xxix.s1<br>https://doi.org/10.5517/ccdc.7bt.g28.xxix.s2

Table 24. *DOI links for the submitted structure sets from each group for target XXX.*

Group	DOI
5	https://doi.org/10.5517/ccdc.7bt.g5.xxx.s1<br>https://doi.org/10.5517/ccdc.7bt.g5.xxx.s2
6	https://doi.org/10.5517/ccdc.7bt.g6.xxx.s1<br>https://doi.org/10.5517/ccdc.7bt.g6.xxx.s2
10	https://doi.org/10.5517/ccdc.7bt.g10.xxx.s1<br>https://doi.org/10.5517/ccdc.7bt.g10.xxx.s2
12	https://doi.org/10.5517/ccdc.7bt.g12.xxx.s1<br>https://doi.org/10.5517/ccdc.7bt.g12.xxx.s2
13	https://doi.org/10.5517/ccdc.7bt.g13.xxx.s1<br>https://doi.org/10.5517/ccdc.7bt.g13.xxx.s2
18	https://doi.org/10.5517/ccdc.7bt.g18.xxx.s1<br>https://doi.org/10.5517/ccdc.7bt.g18.xxx.s2
19	https://doi.org/10.5517/ccdc.7bt.g19.xxx.s1<br>https://doi.org/10.5517/ccdc.7bt.g19.xxx.s2
20	https://doi.org/10.5517/ccdc.7bt.g20.xxx.s1<br>https://doi.org/10.5517/ccdc.7bt.g20.xxx.s2
21	https://doi.org/10.5517/ccdc.7bt.g21.xxx.s1<br>https://doi.org/10.5517/ccdc.7bt.g21.xxx.s2
22	https://doi.org/10.5517/ccdc.7bt.g22.xxx.s1<br>https://doi.org/10.5517/ccdc.7bt.g22.xxx.s2
24	https://doi.org/10.5517/ccdc.7bt.g24.xxx.s1<br>https://doi.org/10.5517/ccdc.7bt.g24.xxx.s2
27	https://doi.org/10.5517/ccdc.7bt.g27.xxx.s1<br>https://doi.org/10.5517/ccdc.7bt.g27.xxx.s2
28	https://doi.org/10.5517/ccdc.7bt.g28.xxx.s1<br>https://doi.org/10.5517/ccdc.7bt.g28.xxx.s2

Table 25. *DOI links for the submitted structure sets from each group for target XXXI.*

Group	DOI
1	https://doi.org/10.5517/ccdc.7bt.g1.xxxi.s1
3	https://doi.org/10.5517/ccdc.7bt.g3.xxxi.s1
5	https://doi.org/10.5517/ccdc.7bt.g5.xxxi.s1
6	https://doi.org/10.5517/ccdc.7bt.g6.xxxi.s1
8	https://doi.org/10.5517/ccdc.7bt.g8.xxxi.s1
10	https://doi.org/10.5517/ccdc.7bt.g10.xxxi.s1
12	https://doi.org/10.5517/ccdc.7bt.g12.xxxi.s1
16	https://doi.org/10.5517/ccdc.7bt.g16.xxxi.s1
18	https://doi.org/10.5517/ccdc.7bt.g18.xxxi.s1
19	https://doi.org/10.5517/ccdc.7bt.g19.xxxi.s1
20	https://doi.org/10.5517/ccdc.7bt.g20.xxxi.s1
21	https://doi.org/10.5517/ccdc.7bt.g21.xxxi.s1
22	https://doi.org/10.5517/ccdc.7bt.g22.xxxi.s1
24	https://doi.org/10.5517/ccdc.7bt.g24.xxxi.s1
25	https://doi.org/10.5517/ccdc.7bt.g25.xxxi.s1
26	https://doi.org/10.5517/ccdc.7bt.g26.xxxi.s1
28	https://doi.org/10.5517/ccdc.7bt.g28.xxxi.s1

Table 26. *DOI links for the submitted structure sets from each group for target XXXII.*

Group	DOI
1	https://doi.org/10.5517/ccdc.7bt.g1.xxxii.s1
3	https://doi.org/10.5517/ccdc.7bt.g3.xxxii.s1
5	https://doi.org/10.5517/ccdc.7bt.g5.xxxii.s1
6	https://doi.org/10.5517/ccdc.7bt.g6.xxxii.s1
10	https://doi.org/10.5517/ccdc.7bt.g10.xxxii.s1
18	https://doi.org/10.5517/ccdc.7bt.g18.xxxii.s1
19	https://doi.org/10.5517/ccdc.7bt.g19.xxxii.s1
20	https://doi.org/10.5517/ccdc.7bt.g20.xxxii.s1
22	https://doi.org/10.5517/ccdc.7bt.g22.xxxii.s1
24	https://doi.org/10.5517/ccdc.7bt.g24.xxxii.s1
25	https://doi.org/10.5517/ccdc.7bt.g25.xxxii.s1
27	https://doi.org/10.5517/ccdc.7bt.g27.xxxii.s1
28	https://doi.org/10.5517/ccdc.7bt.g28.xxxii.s1

Table 27. *DOI links for the submitted structure sets from each group for target XXXIII.*

Group	DOI
1	https://doi.org/10.5517/ccdc.7bt.g1.xxxiii.s1
5	https://doi.org/10.5517/ccdc.7bt.g5.xxxiii.s1
6	https://doi.org/10.5517/ccdc.7bt.g6.xxxiii.s1
8	https://doi.org/10.5517/ccdc.7bt.g8.xxxiii.s1
10	https://doi.org/10.5517/ccdc.7bt.g10.xxxiii.s1
13	https://doi.org/10.5517/ccdc.7bt.g13.xxxiii.s1
19	https://doi.org/10.5517/ccdc.7bt.g19.xxxiii.s1
20	https://doi.org/10.5517/ccdc.7bt.g20.xxxiii.s1
21	https://doi.org/10.5517/ccdc.7bt.g21.xxxiii.s1
22	https://doi.org/10.5517/ccdc.7bt.g22.xxxiii.s1
24	https://doi.org/10.5517/ccdc.7bt.g24.xxxiii.s1
25	https://doi.org/10.5517/ccdc.7bt.g25.xxxiii.s1
27	https://doi.org/10.5517/ccdc.7bt.g27.xxxiii.s1
28	https://doi.org/10.5517/ccdc.7bt.g28.xxxiii.s1

References

Barducci, A., Bussi, G. & Parrinello, M. (2008). *Physical Review Letters*, **100**(2), 020603.
URL: *https://link.aps.org/doi/10.1103/PhysRevLett.100.020603*

Case, D. H., Campbell, J. E., Bygrave, P. J. & Day, G. M. (2016). *Journal of Chemical Theory and Computation*, **12**(2), 910–924.

Chisholm, J. A. & Motherwell, S. (2005). *Journal of Applied Crystallography*, **38**(1), 228–231.
URL: *http://scripts.iucr.org/cgi-bin/paper?S0021889804027074*

de Gelder, R., Wehrens, R. & Hageman, J. A. (2001). *Journal of Computational Chemistry*, **22**(3), 273–289.

van Eijck, B. P. (2005). *Acta Crystallogr. B*, **61**(5), 528–535.
URL: *https://doi.org/10.1107/S010876810502611X*

Francia, N. F. (2022). *Reducing crystal structure overprediction: from small rigid molecules to conformationally complex drugs.* Ph.D. thesis, UCL (University College London).

Groom, C. R., Bruno, I. J., Lightfoot, M. P. & Ward, S. C. (2016). *Acta Crystallographica Section B: Structural Science, Crystal Engineering and Materials*, **72**(2), 171–179.
URL: *http://scripts.iucr.org/cgi-bin/paper?S2052520616003954*

Jakalian, A., Bush, B. L., Jack, D. B. & Bayly, C. I. (2000). *Journal of Computational Chemistry*, **21**(2), 132–146.

MacRae, C. F., Sovago, I., Cottrell, S. J., Galek, P. T., McCabe, P., Pidcock, E., Platings, M., Shields, G. P., Stevens, J. S., Towler, M. & Wood, P. A. (2020). *Journal of Applied Crystallography*, **53**, 226–235.
URL: *https://doi.org/10.1107/S1600576719014092*

Mayo, R. A., Otero-de-la-Roza, A. & Johnson, E. R. (2022). *CrystEngComm*, **24**(47), 8326–8338.

Otero-de-la-Roza, A., Johnson, E. R. & Luaña, V. (2014). *Computer Physics Communications*, **185**(3), 1007–1018.

Spath, H. (1980). *Cluster analysis algorithms for data reduction and classification of objects.* Ellis Horwood Chichester.

Tribello, G. A., Bonomi, M., Branduardi, D., Camilloni, C. & Bussi, G. (2014). *Computer Physics Communications*, **185**(2), 604–613.

Wang, J., Wolf, R. M., Caldwell, J. W., Kollman, P. A. & Case, D. A. (2004). *Journal of Computational Chemistry*, **25**(9), 1157–1174.
URL: *http://doi.wiley.com/10.1002/jcc.20035*

Widdowson, D. & Kurlin, V. (2022). In *Advances in Neural Information Processing Systems.*

Widdowson, D., Mosca, M. M., Pulido, A., Cooper, A. I. & Kurlin, V. (2021). *Match*, **87**(3), 529–559.
