# Supplementary material for: The seventh blind test of crystal structure prediction: structure generation methods
Source: Acta Crystallogr B Struct Sci Cryst Eng Mater. 2024 Dec 1;80(Pt 6):517–47. doi: 10.1107/S2052520624007492 (PMC11789161; doi:10.1107/S2052520624007492)
Supplement: Supplementary file 2 [file b-80-00517-sup2.pdf]

Supplementary Information B (Methods SI per group). The Seventh Blind Test of Crystal Structure Prediction: Structure Generation Methods

Lily M. Hunnisett, *et al.* *

The Cambridge Crystallographic Data Centre, 12 Union Road, Cambridge CB2 1EZ, UK. E-mail: lhunnisett@ccdc.cam.ac.uk

Contents

1 Group 1 **4**

2 Group 3 **39**

3 Group 5 **49**

4 Group 6 **56**

5 Group 8 **69**

6 Group 10 **73**

7 Group 11 **91**

8 Group 12 **97**

9 Group 13 **104**

10 Group 16 **111**

11 Group 17 **120**

12 Group 18 **122**

13 Group 19 **126**

14 Group 20 **130**

15 Group 21 **144**

16 Group 22 **163**

17 Group 23 **166**

18 Group 24 **175**

19 Group 25 **191**

20 Groups 26 and 27 **194**

21 Group 28 **237**

1. Group 1

Seventh Blind Test of Organic Crystal Structure Prediction Methods

Supporting Information for the Adjiman-Pantelides group

Isaac J. Sugden, David H. Bowskill, Stefanos Konstantinopoulos, Yizu Zhang, Leticia Sanders De Almada, Benjamin I. Tan, Claire S. Adjiman, Constantinos C. Pantelides

Department of Chemical Engineering, Sargent Centre for Process Systems Engineering and Institute for Molecular Science and Engineering, Imperial College London, London SW7 2AZ

c.adjiman@imperial.ac.uk; c.pantelides@imperial.ac.uk

1. Methodology

1.1. General methodology

The objective of the CSP studies conducted by this group was to generate lists of candidate structures for further refinement with more accurate models. Thus, emphasis was placed on the global search stage and initial refinement to produce a computationally-tractable number of candidate structures. The general methodology used for Crystal Structure Prediction (CSP) by the Adjiman-Pantelides group during the seventh blind test follows the three-stage workflow presented in (Pantelides et al., 2014) which involves analysis, global search and refinement. With further standardisation introduced for the seventh blind test, this can be further divided into an eight-stage process. In this section, the modelling framework used is first introduced, followed by a description of the eight steps.

1.1.1. Modelling framework

At the core of our approach is the minimisation of the crystal lattice energy U_{latt} at 0 K which is evaluated by combining energy contributions obtained for the specific target molecule(s) from *in vacuo* quantum-mechanical (QM) calculations and from transferable potentials. Specifically, U_{latt} is divided into an intramolecular contribution, ΔU_{intra}, which captures the energy penalty for conformational change from the most stable state *in vacuo*, and intermolecular contributions U_{inter}:

$$U_{latt} = \Delta U_{intra} + U_{inter}.$$

U_{inter} depends on variable vector X, which consists of the unit cell parameters and molecule positions in the unit cell (orientation and position of the centres of mass). It is partitioned into two terms: one that represents electrostatic interactions, U^{ele}, which are modelled either through point charges or multipoles and derived from the electrostatic potential of the isolated molecule(s) and an empirical term that represents repulsion / dispersion interactions, $U^{rep/disp}$, which is based on a transferable isotropic atom-atom exp-6 Buckingham potential. Efficient evaluations of the lattice energy are made possible by a partitioning of the conformational degrees of freedom into independent (θ) and dependent ($\bar{\theta}$) degrees of freedom and the introduction of local approximate models (LAMs) (Habgood et al., 2015,

Kazantsev et al., 2010, Sugden et al., 2016, Sugden et al., 2019) in which second-order expansions around isolated-molecule QM calculations at reference points θ_{ref} make it possible to compute the impact of conformational changes on U_{intra} and U^{ele} at very low cost. Overall, the lattice energy model is thus given by:

$$U_{latt}(\bar{\theta}, X) = \Delta U_{intra}(\bar{\theta}) + U^{ele}(\bar{\theta}, X) + U^{rep/disp}(\bar{\theta}, X),$$

The model accuracy can be iteratively improved during the course of CSP by selecting different sets of independent degrees of freedom (e.g., from a few torsions to all torsions and bond angles), using different representations of electrostatic interactions (point charges or multipoles), decreasing or changing the level of theory/basis set used in the QM calculations.

1.1.2. Detailed description of methodology

The eight steps of the systematic CSP procedure are as follows.

<u>Step 1: Initial partitioning of conformational degrees of freedom</u>

The extent of flexibility of torsion angles and their impact on the intramolecular energy are investigated with the aim to identify those degrees of freedom that are flexible <u>and</u> affect ΔU_{intra}. This is done using a combination of (i) quantum mechanical minimisations of the geometry of the target molecule *in vacuo* for different values of the torsion angles, with PBE/6-311G(d,p) as the default level of theory/basis set combination, (ii) identification of similar molecules / molecular fragments in the CSD and of the range of flexibility exhibited by the corresponding torsion angles and (iii) chemical intuition. The selected torsion angles are assigned to the set $\bar{\theta}$ of independent degrees of freedom, with associated lower and upper bounds $[\bar{\theta}^L, \bar{\theta}^U]$, while the remaining torsion angles, all bond angles and all bond lengths are assigned to the set θ of dependent degrees of freedom.

In this step, the partitioning of the independent degrees of freedom into torsional groups is also considered if there are 5 or more torsions in $\bar{\theta}$. This is done in a manner similar to CrystalPredictor I (Karamertzanis & Pantelides, 2007), but modified for CrystalPredictor II (Kazantsev et al., 2010, Habgood et al., 2015, Sugden et al., 2016, Sugden et al., 2019, Sudgen et al., in preparation).

<u>Step 2: Screen basis sets using conformational scans</u>

While our approach offers complete flexibility in terms of level of theory and basis set, most calculations for the blind test are performed using the PBE0 hybrid functional, but both level of theory and the choice of basis set can be explored at this step to make choices specific to each target. To select the specific model, we perform 1-dimensional conformational scans using CrystalPredictor II (Kazantsev et al., 2010, Habgood et al., 2015, Sugden et al., 2016, Sugden et al., 2019, Sudgen et al., in preparation) for $\bar{\theta} \in [\bar{\theta}^L, \bar{\theta}^U]$ in 30° increments using one or more levels of theory and/or basis sets (e.g., PBE, HF, 6-31G(d,p), 6-311G(d,p)). For molecules with few independent degrees of freedom, two-dimensional scans are sometimes conducted. At each point all remaining degrees of freedom are optimised, starting from their positions at the gas-phase energy minimum and a LAM is generated. Those basis sets that do not provide sufficient accuracy, as determined by comparison with the results of calculations

with the most extended basis set considered, are eliminated. Those basis sets that yield high accuracy at a comparatively high computational cost per LAM point are also eliminated. Any remaining basis set is considered in Steps 3 and 4 to finalise model selection.

Step 3: Compare model results to any known experimental data

Possible levels of theory, basis sets and transferable parameters for the repulsion / dispersion contribution are assessed based on local minimisations starting from any experimental crystal structures of the target, of individual molecules within any multicomponent target, or similar molecules that exists in the Crystal Structure Database (CSD). For flexible molecules, this is done using CrystalOptimizer (Kazantsev et al., 2011) or its newly developed equivalent Crystal Structure Optimizer for Flexible Molecules (CSO-FM) (Bowskill, 2021). For rigid molecules, Crystal Structure Optimizer for Rigid Molecules (CSO-RM) (Bowskill, 2021) is used. In all cases, the electrostatic interactions in U^{ele} are modelled using multipoles derived from isolated-molecule QM calculations at reference LAM points using GDMA (Stone, 2005). The $U^{rep/disp}$ parameters considered include FIT (Bayer & Price, 2000, Coombes et al., 1996, Williams & Cox, 1984, Hsu & Williams, 1980, Williams & Houpt, 1986, Cox et al., 1981), and DB2020 (Bowskill, 2021). The results of the local minimisations are used to eliminate unsuitable basis sets and parameter sets and to rank remaining options, based on the following questions:

- Are all crystal structures reproduced well geometrically (with $RMSD_{15} \leq 0.8$ Å)?
- If any polymorphic structures exist, are there computed lattice energies within 10 kJ/mol of each other?
- Do all crystal structures display intramolecular energy contributions within the expected range (usually 20 kJ/mol) (Cruz-Cabeza & Bernstein, 2014)?

Step 4: If required, perform CSP on similar system(s)

If elements of the target are unusual or require specific modelling choices (e.g., atom types for which transferable parameters are not available / not well tested), we perform a full CSP search for one or more similar system from the CSD. The results of the full CSP search are used to finalise the choice of basis set and transferable parameters set, using the following considerations:

- Are the relative stability rankings in good agreement with experimental data?
- What is the computational cost of the viable modelling options?

This step concludes model selection and the production phase of the CSP investigation can be initiated.

Step 5: LAM database generation

An initial LAM database is generated by carrying out isolated-molecule QM calculations with the selected basis at different values of the independent torsions. This is done using a uniform grid with a large step size (Habgood, et al., 2015), followed by the adaptive generation of reference LAM points (Sugden et al., 2016, Sugden et al., 2019) using an

intramolecular energy cut-off of 20 kJ/mol by default and a maximum LAM energy difference (Δ^*) of 5 kJ/mol by default.

Step 6: Global search and clustering

We generate putative crystal structures from an extensive search space using CrystalPredictor version 2.4.4 (Habgood et al., 2015, Kazantsev et al., 2010, Sugden et al., 2016, Sugden et al., 2019, Sudgen et al., in preparation). Only Z'=1 structures are generated for all searches undertaken during the blind test unless specified otherwise in the individual target descriptions. Normally, structures are generated within the 59 space groups most commonly found in the CSD (*P1, P-1, P21, P21/c, P21212, P212121, Pna21, Pca21, Pbca, Pbcn, C2/c, Cc, C2, Pc, Cm, P21/m, C2/m, P2/c, C2221, Pmn21, Cmc21, Aba2, Fdd2, Iba2, Pnna, Pccn, Pbcm, Pnnm, Pmmn, Pnma, Fddd, Ibam, P41, P43, I-4, P4/n, P42/n, I4/m, I41/a, P41212, P43212, P-421c, I-42d, P31, P32, R3, P-3, R-3, P3121, P3221, R3c, R-3c, P61, P63, P63/m, P213, Pa-3, Cmcm and Cmca*), with each space group searched at a frequency matching that of the space group in the CSD. Only structures within 20 kJ/mol of the best known lattice energy minimum are retained for further analysis. A clustering algorithm is used to eliminate duplicate structures by comparing the RMSD_{15} between any pair of structures (i,j) with a density difference $|\rho_\mathrm{i} - \rho_\mathrm{j}|$ ≤20 g/cm^3 and a lattice energy difference $|U_{latt,i} - U_{latt,j}|$ ≤3 kJ/mol. If the two structures in pair (i,j) have similar geometries, i.e., $|\mathrm{RMSD}_{15,i} - \mathrm{RMSD}_{15,j}| \leq 0.8$ Å, the structure with the largest lattice energy is removed.

Step 7: Refinement stage I and clustering

We perform local minimisations for the lowest energy unique structures identified in Step 6 with a multipole-based model, using CSO-RM if the target is rigid and CrystalOptimizer or CSO-FM if it is flexible. In the latter case, we expand the set of independent degrees of freedom to allow for a more detailed representation of flexibility. CrystalOptimizer and CSO-FM are based on a bi-level optimisation, with the independent degrees of freedom as variables at the outer level, and the unit cell parameters and molecular positions/orientations as variables at the inner level. The inner optimisation is performed using DMACRYS (Price et al., 2010) within CrystalOptimizer or CSO-RM within CSO-FM. At the outer level, quantum mechanical calculations on the isolated molecule are carried out on-the-fly and LAMs are then used to approximate the lattice energy, dependent degrees of freedom, and multipole moments based on these calculations. New LAM points are generated based on the distance between the current point and the nearest point available in the LAM database. For the intramolecular energy and dependent degrees of freedom, the distance is calculated based on the independent degrees of freedom only. For the electrostatics, the distance is instead calculated based on all degrees of freedom. When the distance metric exceeds some threshold, it is then deemed necessary to conduct an additional quantum mechanical calculation and add an additional LAM point to the respective database (hence 'on-the-fly'). In this way, the final LAM databases will often contain more electrostatic LAM points than energy / geometry points. The number of structures refined is based on a target of evaluating all structures from Step 6 that are found within 10 kJ/mol of the global minimum, although this is sometimes curtailed due to limited computational resources. A further clustering process is performed after the refinement, using the same settings as in Step 6.

Step 8: DFT-D refinement stage II

If time and resources are available, we perform DFT-D local minimizations for the lowest-energy structures from Step 7 within the Vienna *ab initio* Software Package (VASPv5.4.4) (Hafner, 2008). The TPSS functional and D3 dispersion correction are selected due to their good performance in multiple benchmark studies (Brandenburg & Grimme, 2016) relative to PAW pseudo-potentials. A large energy cut-off of 1000 eV is used with a tight Γ-centred *k*-point mesh of at most $0.040\times 2\pi$ Å^{-1} distance between each *k*-point. The crystal structures are relaxed allowing variation of the unit cell lengths, angles and atom positions until all forces are less than 0.01 eV/Å.

1.2. Software used

All quantum mechanical calculations are performed within Gaussian 09 package (Frisch et al., 2016). The corresponding charge density is analysed to provide atomic charges based on the HLYGAt (Hu et al., 2007) method and atomic multipoles up to hexadecapole are derived from Distributed Multipole Analysis (Stone, 2005) using GDMAv2.2. For the global search processes, CrystalPredictor v2.4.4 was used as default. For rigid-body crystal structure optimisation, CSO-RMv1.3 was used as default, while CrystalOptimizer v2.4.9 and CSO-FMv1.3 were used for structures with independent intramolecular flexibilities.

2. Summary of CSP for each target considered

Targets XXIX, XXXI, XXXII and XXXIII were investigated and specific aspects for each molecule are described in the remainder of this document. Approximate CPU times for all calculations are reported in Section 2.5.

2.1. Target XXIX (Stefanos Konstantinopoulos)

2.1.1. Step 1. Initial partitioning of conformational degrees of freedom

The three degrees of freedom to be treated as flexible during the candidate generation stage were chosen based on chemical intuition and are shown in Figure 1. This choice was made to generate sufficient conformations at the search stage, while all torsions were treated as flexible during the refinement stage.

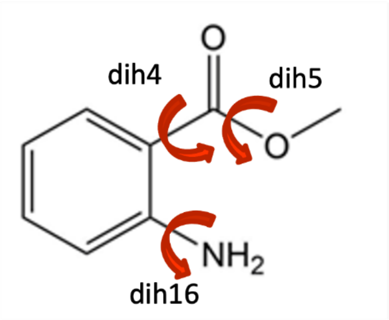

Figure 1: Molecular connectivity diagram and degrees of freedom treated as flexible for molecule XXIX

2.1.2. Step 2: Screen basis sets using conformational scans

Two-dimensional scans were performed by conducting constrained isolated quantum mechanical calculations with the PBE0/6-311+G(d,p) level of theory and basis set combination. For each 2D scan, one of the dihedrals was kept constant to its gas phase value, while the other two were varied in the range 0-360° in steps of 30°. The generated heat maps allow the identification of the low energy conformations (see Figure 1).

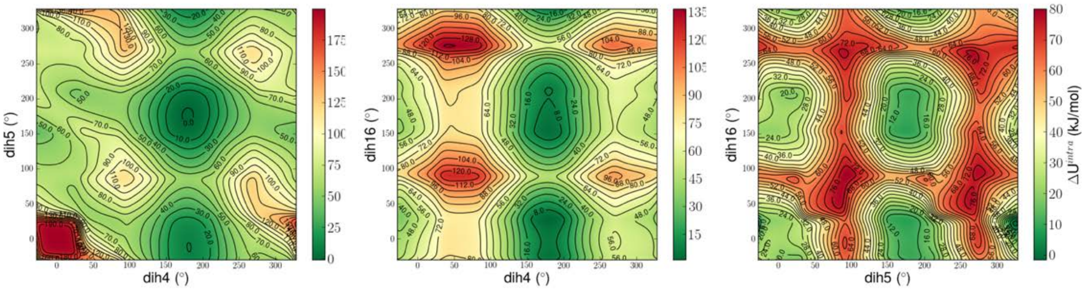

Figure 2: 2D scans for dihedrals dih4, dih5, and dih16 of Target XXXI. Calculations performed at PBE0/6-311+G(d,p) level of theory and basis set

2.1.3. Step 3: Compare model results to any known experimental data

No experimental information was available at the time of the CSP study; hence this step was skipped.

2.1.4. Step 4: If required, perform CSP on similar system(s)

The polymorphs of Anthranilic acid (CSD refcode family AMBACO) were locally minimised using CSO-FM to assess the performance of our refinement stage algorithm on a similar system. Target XXIX and Anthranilic acid are fairly similar, with the former being an ester while the latter is a carboxylic acid.

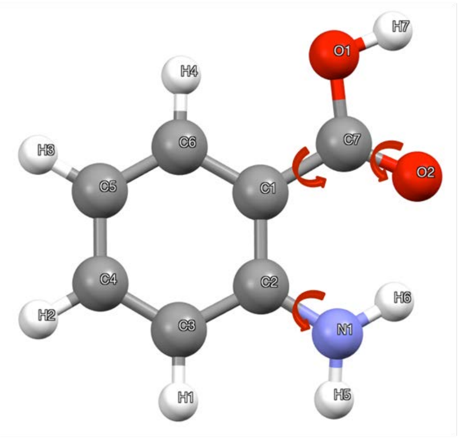

Figure 3: AMBACO molecule with the degrees of freedom chosen to be treated as flexible

All three forms were used as input for local minimisations using CSO-FM. Three torsional degrees of freedom were treated as flexible (see Figure 3). Quantum mechanical calculations were performed using PBE0/6-311+G(d,p). The parameters used to model repulsion-dispersion interactions (parameter set DB2020_PBE0_6-311+G(d,p)_mp were developed at the same level of theory/basis set (Bowskill, 2021). As can be seen in Table 1, the geometry reproduction is satisfactory for all the forms. Experimental studies suggest that form I is the most stable polymorph at 0 K, in agreement with the energy ranking suggested by our model. This model is thus retained for the next steps.

Table 1 Geometry differences between the experimental and locally minimised structures, as indicated by $RMSD_1$ and $RMSD_{15}$ and computed lattice energies for AMBACO polymorphs

	**REFCODE**	**$RMSD_1$ (Å)**	**$RMSD_{15}$ (Å)**	**U_{latt} (kJ/mol)**
**Form 1**	AMBACO07	0.039	0.235	-90.38
**Form 2**	AMBACO10	0.041	0.399	-83.48
**Form 3**	AMBACO08	0.045	0.405	-88.12

2.1.5. Step 5: LAM database generation

A total of 328 LAM points for the global search were generated at the PBE0/6-311+G(d,p) level of theory and basis set. All three flexible torsions were treated as flexible in the range of 0-360°. Further details of the LAM generation are shown in Table 2.

Table 2: Details of LAM generation for Target XXIX

Uniform Grid Interval (°)	60.0
No. of LAMs in Uniform Grid	216
Adaptive LAMs High-Energy Cut-off (kJ/mol)	30.0
Adaptive LAMs Accuracy Cut-off (kJ/mol)	5.0
No. of Adaptive LAMs Generated	112
Total LAM Database Size	328

2.1.6. Step 6: Global search and clustering

A global search for one molecule in the asymmetric unit cell (Z'=1) was performed using the LAM database generated in Step 5 and FIT potential parameters to model repulsion-dispersion interactions. Key details are presented in Table 2 and the resulting landscape is shown in Figure 4.

Table 3: Key information regarding the global search of Target XXIX

No. of Minimisations Conducted	1,000,000
No. of Unique Structures Post-clustering	16,856
Global Minimum Energy (kJ/mol)	-92.860
Energy Cut-off (kJ/mol)	20.0
Unique Structures within Cut-off	6,980

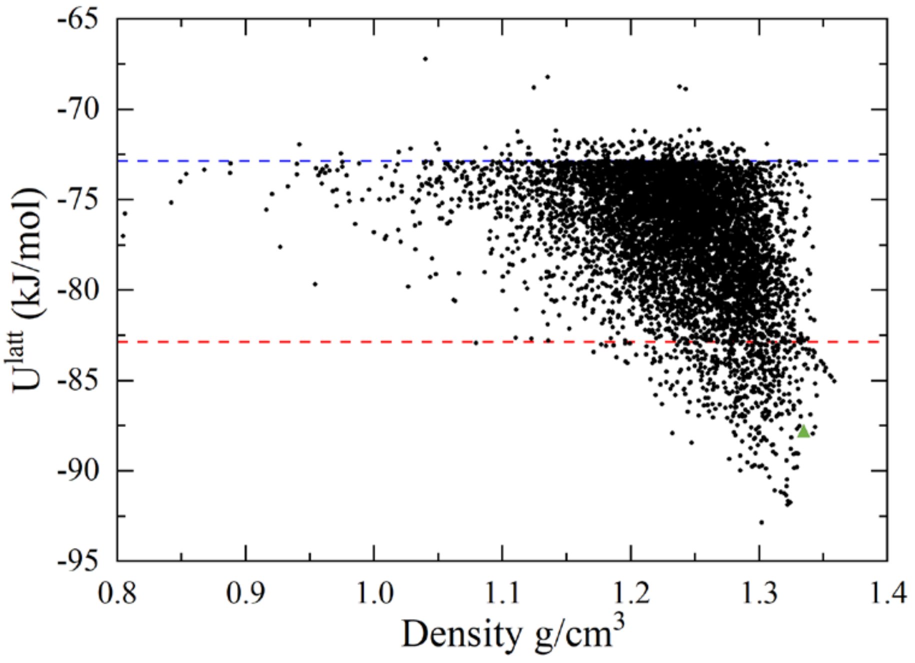

Figure 4 - Global search landscape for Target XXIX. The red line denotes the 10 kJ/mol energy cut-off, the blue line denotes the 20 kJ/mol energy cut-off, the black circles the unique Z'=1 structures identified. The Z'=3 experimental structure (FASMEV) was minimised with the global search model following its disclosure and is indicated by a green triangle

Step 7: Refinement stage I and clustering

All 6,980 Z'=1 structures within the 20 kJ/mol energy cut-off were refined with CSO-FM. All structures were locally minimised treating all torsions as independent degrees of freedom. Quantum mechanical calculations were performed with PBE0/6-311+G(d,p). The parameters used to model repulsion-dispersion interactions were developed at the same level of theory/basis set (DB2020_PBE0_6-311+G(d,p)_mp) as described by (Bowskill, 2021). Key data on this step are shown in Table 4 and the resulting landscape is shown in Figure 5.

Table 4 - Key information on refinement stage I for Target XXIX

No. of Structures Refined	6,980
No. of Unique Structures Post-clustering	4,359
Global Minimum Energy (kJ/mol)	-84.899
Energy Cut-off (kJ/mol)	10.0
Unique Structures within Cut-off	685

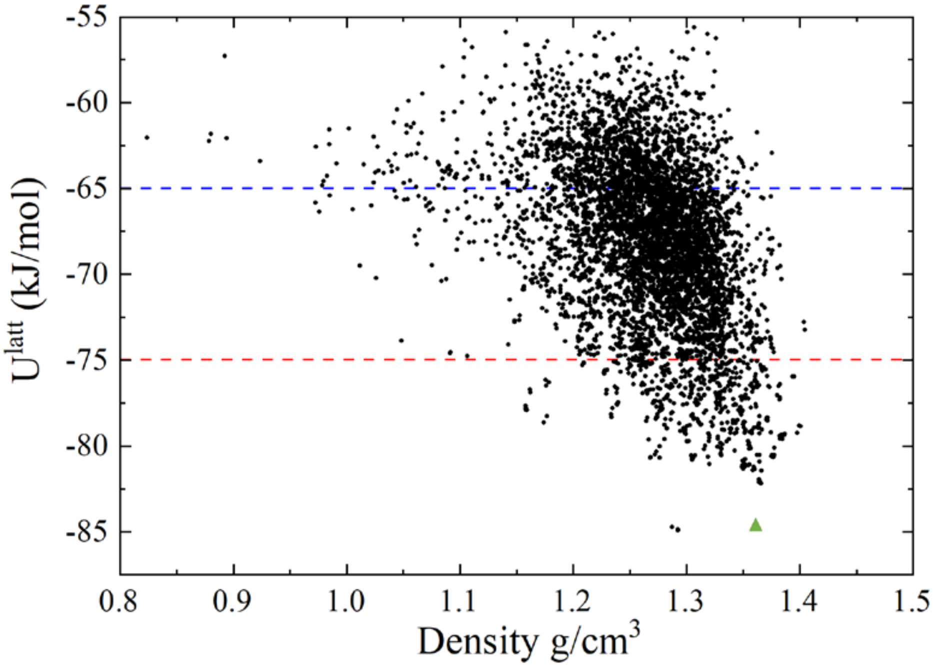

Figure 5: Refinement I landscape for Target XXIX following the use of CSO-FM and clustering. The red line denotes the 10 kJ/mol energy cut-off, the blue line the 20 kJ/mol cut-off, the black circles the unique Z'=1 structures identified. The Z'=3 experimental structure (FASMEV) was minimised with the CSO-FM model following its disclosure and is indicated by an green triangle

2.1.7. Discussion

The experimental structure was not found since it has three molecules in the asymmetric unit cell. Following the release of experimental data, the experimental structure of Target XXIX (REFCODE FASMEV) was locally minimised with CrystalPredictor and CSO-FM using the same modelling choices during the blind test. The lattice energy of FASMEV minimised with CrystalPredictor is -87.86 kJ/mol. This is well within the 20 kJ/mol energy cut-off used in the global search at just 4.99 kJ/mol above the Z’=1 global minimum. The structure obtained after minimisation is a good match for the experimental structure with a $RMSD_{20}$ of 0.194 Å.

A similar approach is applied with CSO-FM. The lattice energy of the structure resulting from local minimisation starting from FASMEV -84.65 kJ/mol and. This places the structure at just 0.25 kJ/mol above the lowest-energy Z’=1 structure, with a rank of 3. The $RMSD_{20}$ between the experimental structure (FASMEV) and its locally-minimised equivalent is 0.193 Å, indicating that a high quality match once again.

We can conclude from this that the models used in the global and local search are suitable for Target XXIX and that, had Z’>1 searches been completed, the experimental structure would have been very likely included in the submitted list due to its low rank in both landscapes generated (see Figure 4 and Figure 5).

2.2. Target XXXI (David H. Bowskill and Benjamin I. Tan)

2.2.1. Step 1. Initial partitioning of conformational degrees of freedom

An isolated gas-phase minimisation of Target XXXI was conducted using the PBE0/6-311G(d,p) level of theory and basis set. Analysis of the first-order and second-order derivatives of each torsion within the molecule suggested five flexible dihedrals, as shown in Figure 6. Chemical intuition would suggest that rotation of dih7 and dih11 (shown in blue in the figure) could disrupt the planar nature of the two rings, hence these two torsions were treated as rigid instead. The remaining three torsions (dih8, dih9, and dih10, shown in orange) were considered to be flexible in the remainder of the CSP study, i.e., $\bar{\theta} =$ (dih8,dih9,dih10). They correspond to rotation of the plane of the heterocyclic ring (dih8), rotation along the central C-S bond (dih9), and rotation of the plane of the benzene ring (dih10).

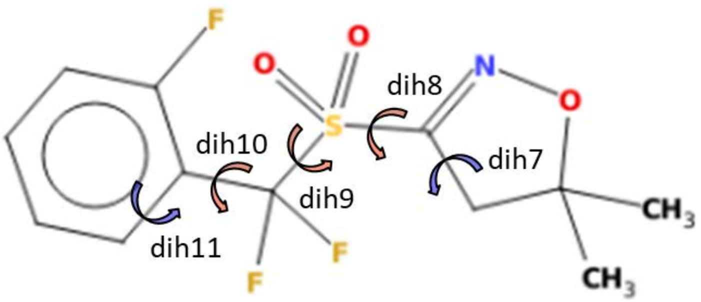

Figure 6: Flexible torsions in Target XXXI found from Step 1 analysis. Torsions in blue (dih7 and dih11) were eliminated by chemical intuition. Torsions in orange (dih8, dih9, and dih10) were treated as flexible for the remainder of the CSP study.

2.2.2. Step 2: Screen basis sets using conformational scans

1-D torsion scans were conducted on the aforementioned three dihedrals at the PBE0 level of theory with three different basis sets: 6-31G(d,p), 6-311G(d,p), 6-311+G(d,p). Each torsion was scanned over a full 360° rotation (Figure 7). The 1-D scans show regions of low conformational energy (< 20 kJ/mol), reaffirming that these torsions do indeed exhibit some degree of flexibility. All three basis sets tested exhibit nearly identical energy profiles, with

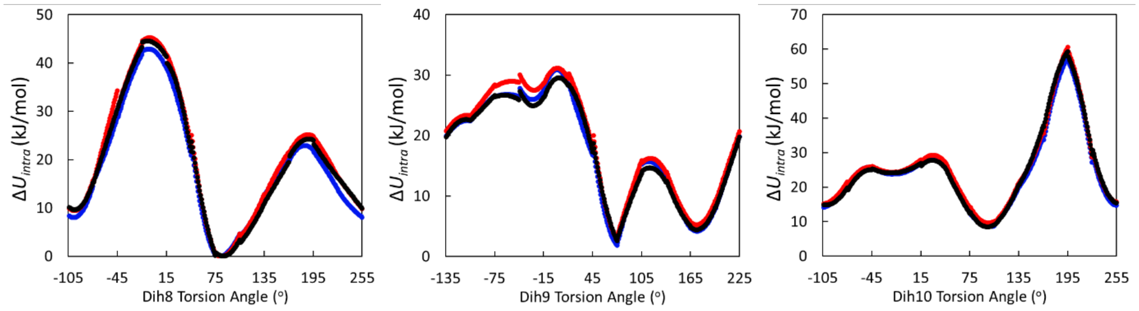

Figure 7: 1-D torsions scans for the flexible torsions of Target XXXI identified in Step 1. All scans were conducted at the PBE0 level of theory. The basis sets tested include 6-31G(d,p) (blue), 6-311G(d,p) (red), and 6-311G+(d,p) (black).

only minor variations in ΔU_{intra}. The choice of basis set was thus made based on the computational costs of each method. The average CPU hours for each LAM calculation increases with basis set size, as expected: 36.4, 40.7, and 96.7 respectively. Given the sharp rise in CPU hours for the 6-311G+(d,p) basis set, this option was discarded. The 6-311G(d,p)

basis set only costs marginally more than the 6-31G(d,p) basis set, therefore 6-311G(d,p) was ultimately selected as the QM method for the rest of the CSP study.

2.2.3. Step 3: Compare model results to any known experimental data

No experimental data was known at the time of the CSP study, hence this step was skipped.

2.2.4. Step 4: If required, perform CSP on similar system(s)

Target XXXI was not deemed to have any peculiarities in its structure, hence no testing was conducted on analogous structures.

2.2.5. Step 5: LAM database generation

The LAM generation was conducted using the PBE0/6-311G(d,p) level of theory and basis set (as selected in Step 2). All three independent torsions were assumed to be flexible over a full 360° rotation. Details of the LAM generation are summarised in Table 4.

Table 5: Details of the initial LAM generation for Target XXXI.

Uniform Grid Interval (^{o})	60.0
No. of LAMs in Uniform Grid	216
Adaptive LAMs High-Energy Cut-off (kJ/mol)	30.0
Adaptive LAMs Accuracy Cut-off (kJ/mol)	5.0
No. of Adaptive LAMs Generated	1,287
Total LAM Database Size	1,503

2.2.6. Step 6: Global search and clustering

As the Z' number of Target XXXI polymorphs was unknown *a priori*, both Z' = 1 and Z' = 2 global searches were conducted. The searches were both conducted using CrystalPredictor v2.4.4 within the standard space groups (61 most common, i.e., the 59 listed in Section 1.1.2 as well as *P2221* and *Pba2*), The LAM database developed in Step 5 and point-charge electrostatics were used in both searches. Repulsion-dispersion parameters developed for use with the selected energy model (DB2020_PBE0_6-311G(d,p)_pc) (Bowskill, 2021) were used. Key details of the searches are summarised in Table 2.

Table 6: Key information regarding the global search of Target XXXI. The energy cut-offs are defined with respect to the global minimum from each individual search.

Z'	**1**	**2**
No. of Minimisations Conducted	1,000,000	2,000,000
No. of Unique Structures Post-clustering	5,959	2,596
Global Minimum Energy (kJ/mol)	-127.013	-125.178
Energy Cut-off (kJ/mol)	15.0	14.0
Unique Clusters within Cut-off	1,503	2,596

The landscapes for each search (post-clustering) are shown in Figure 8, together with any experimental matches found. In the Z' = 1 search, two matches for $ZEHGAY_{major}$ were found,

neither of which were eliminated by the clustering algorithm. $ZEHGAY_{minor}$ and ZEHFUR were also found within the energy cut-off in the Z' = 1 search. In the Z' = 2 search, only the $ZEHGAY_{minor}$ form was found. The fourth polymorph, ZEHGEC, was not found in either of the searches done.

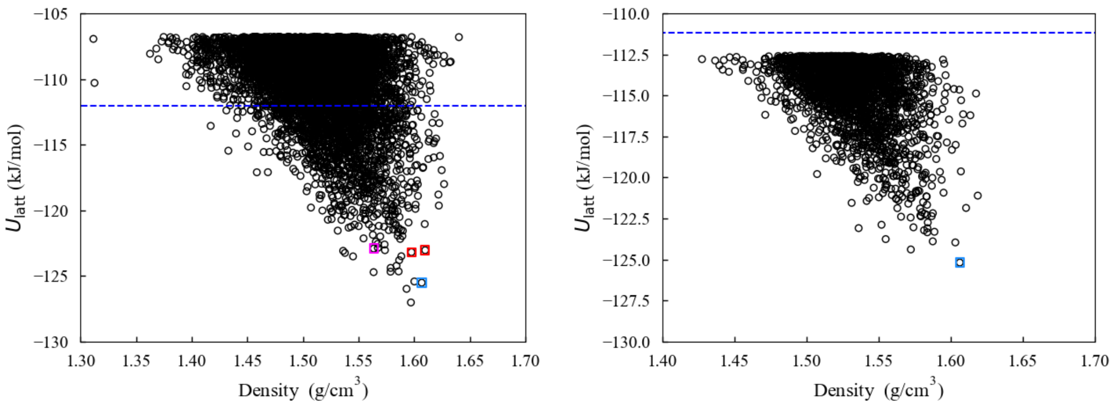

Figure 8: The crystal structure landscape for the Z' = 1 (left) and Z' = 2 (right) search conducted with CrystalPredictor v2.4.4. Unique structures found post-clustering are given by the empty black circles. The cut-off for structures used in refinement is given by the blue dashed line. Matches to the polymorphs $ZEHGAY_{major}$ (red), $ZEHGAY_{minor}$ (blue), and ZEHFUR (magenta) are given by the empty squares. ZEHGEC was not found in either search.

2.2.7. Step 7: Refinement stage I and clustering

Unique clusters within the energy cut-off from Step 6 were brought forward for further refinement of each Z' search. Multipole electrostatics were used in both refinements. Again, repulsion-dispersion parameters were developed for use with this energy model (DB2020_PBE0_6-311G(d,p)_mp) as described by (Bowskill, 2021). Using CSO-FM, LAMs are generated on-the-fly as per Table 6. The same flexible degrees of freedom used in the Global Search were employed here, without any further addition of flexible degrees of freedom. Both the Z' = 1 and Z' = 2 searches employ the same LAM databases. Key details of the refinements are summarised in Table 7. When clustering of the Z' = 2 structures, a comparison with structures found in the Z' = 1 search was also conducted, in effect removing any Z' = 2 structures which had already been found in the Z' = 1 search.

The landscapes for each search (post-clustering) are shown in Figure 9, together with any experimental matches found. The experimental matches found in the Z' = 1 search at the global search stage were all found here, although the ZEHFUR match increased substantially in energy. The fourth polymorph, ZEHGEC, was not found in either of the searches done.

Table 7: Description of LAM generation for the first refinement stage for Target XXXI. The same LAM databases were used in both the Z' = 1 and Z' = 2 searches. The same flexible degrees of freedom are identical to those used in the Global Search.

Valid Torsion Range (^{o})	5.0
Valid Angle Range (^{o})	3.5
Valid Bond Range (Å)	0.1
No. of Intramolecular Energy LAMs	5,851
No. of Electrostatic LAMs	9,056

Table 8: Key Information regarding the refinement stage of Target XXXI. When clustering Z'=2 structures, structures found in the Z'=1 search were also clustered out. The energy cut-offs are defined with respect to the Z'=1 global minimum.

Z'	**1**	**2**
No. of Structures Refined	1,503	2,161
No. of Unique Structures Post-clustering	1,328	1,930
Global Minimum Energy (kJ/mol)	-123.430	-122.183
Energy Cut-off (kJ/mol)	5.0	5.0
Unique Clusters within Cut-off	37	9

In the Z' = 2 search, the previously-identified $ZEHGAY_{minor}$ structure match was eliminated from the landscape. Analysis of the Z' = 2 minima pre-clustering reveals that the $ZEHGAY_{minor}$ match was present, hence its disappearance in the final landscape can be attributed to the clustering done with respect to the Z' = 1 structures.

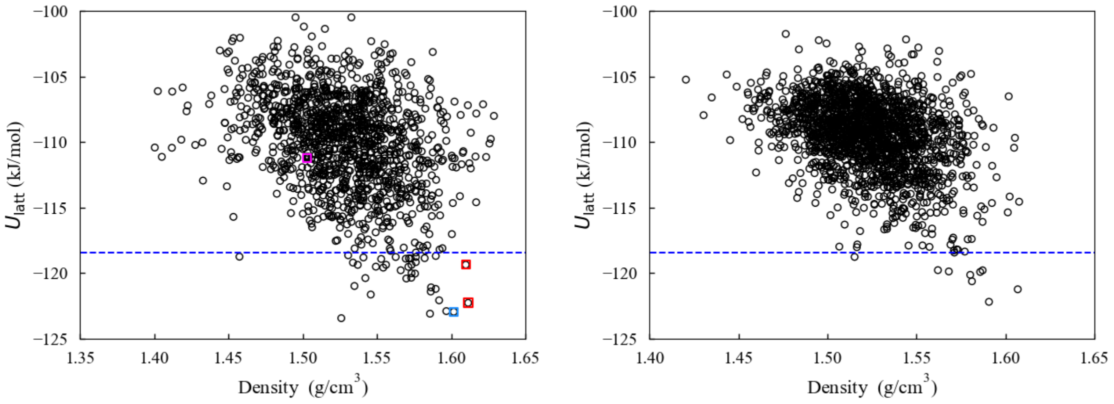

Figure 9: The crystal structure landscape for the Z' = 1 (left) and Z' = 2 (right) refinement conducted with CSO-FM for Target XXXI. Unique structures found post-clustering are given by the empty black circles. The cut-off for structures progressing to Step 8 is given by the blue dashed line. Matches to the polymorphs $ZEHGAY_{major}$ (red), $ZEHGAY_{minor}$ (blue), and ZEHFUR (magenta) are given by the empty squares. ZEHGEC was not found in either search.

2.2.8. Step 8: DFT-D refinement stage II

Forty-six structures were brought forward from Step 7 for DFT-D refinement using VASP. This includes the two $ZEHGAY_{major}$ matches and the $ZEHGAY_{minor}$ match from the Z'= 1 search. The ZEHFUR match in the Z' = 1 search was too high in energy to be brought forward. A large energy cut-off of 1000 eV is used with a tight Γ-centred *k*-point mesh of at most $0.040 \times 2\pi$ Å^{-1} distance between each *k*-point.

The structures which matched ZEHGAY in CSO-FM were still able to successfully match the experimental polymorphs post-refinement with VASP. No matches to the other experimental polymorphs were found following refinement.

2.2.9. Discussion

The fidelity of the structural predictions and the relative rankings of experimental matches at each stage of the CSP workflow are summarized in Table 8. Since all the polymorphs have

been revealed to be Z’ = 1, the results for Z’ = 2 have been omitted. With the exception of ZEHGEC, all the other polymorphs were identified at least once within the CSP workflow.

Table 9: Structural fidelity and relative rankings of experimental matches at each stage of the CSP for Target XXXI. Only the matches from the Z’ = 1 search are shown since the experimental structures are known to be Z’ = 1. Two matches to $ZEHGAY_{major}$ were identified throughout the study. ZEHGEC was not found in the original CSP study, and the results shown correspond to analysis done by minimising the experimental structure with the corresponding energy model at each stage.

**Polymorph**	**Global Search**		**Refinement I**		**Refinement II**	
	**$RMSD_{20}$ (Å)**	**U_{latt} (kJ/mol) [Rank]**	**$RMSD_{20}$ (Å)**	**U_{latt} (kJ/mol) [Rank]**	**$RMSD_{20}$ (Å)**	**U_{latt} (kJ/mol) [Rank]**
$ZEHGAY_{major}$	0.5342	-123.181 [14th]	0.3346	-119.356 [24th]	0.1337	-123.505 [3rd]
$ZEHGAY_{major}$	0.2163	-123.031 [17th]	0.1774	-122.262 [5th]	0.1665	-124.086 [1st]
$ZEHGAY_{minor}$	0.3759	-125.507 [3rd]	0.2589	-122.941 [3rd]	0.2635	-122.759 [8th]
ZEHFUR	0.5164	-122.906 [18th]	0.7027	-111.206 [450th]	NOT FOUND	
ZEHGEC*	0.7450	-98.505	0.1299	-103.481	NOT FOUND	

Relative Polymorph Stabilities

Of the three polymorphs that were found, ZEHFUR is correctly identified as the least stable of the three. However, the lattice energy of ZEHFUR is likely overstated in the first refinement stage, wherein it is ranked 450th and is about +12.4 kJ/mol from the global minimum (-123.430 kJ/mol). Energy gaps exceeding 10 kJ/mol are considered very unlikely for observable polymorphs (Nyman & Day, 2015), hence this possibly evidences an underlying error in our energy model. This large energy gap also results in ZEHFUR being excluded from the final VASP refinement. It is noted that the geometry match in the first refinement stage for ZEHFUR is notably worse than the other experimental matches.

In the first refinement stage, all the ZEHGAY matches present with good geometry fidelity. However, the relative rankings of (both) the major and the minor forms are reversed. Nevertheless, these structures were low enough in energy to be brought forward to the second refinement stage where their rankings are corrected.

Minimisation of ZEHFUR Experimental Structure

As noted above, the lattice energy of the ZEHFUR match in the first refinement stage is likely predicted to unrealistically large and does not match the experimental geometry as well as the structures predicted for the ZEHGAY polymorphs. Given that a much better match of ZEHFUR was found during the global search, this is likely due the limitations of the energy model used during Refinement I. To test this, a local minimization in CSO-FM was initiated from the ZEHFUR experimental structure using the same energy model as Refinement I.

The resultant minimum has a much lower energy (U_{latt} = -119.223 kJ/mol) than what was originally found in the CSP study, but the geometry can no longer be considered a match to the experimental structure ($RMSD_{20}$ = 1.4367 Å). Thus, the inability to obtain an energetically low-lying ZEHFUR structure in the first refinement stage is most likely a fault of the energy model. This is further reinforced by the fact that when we attempt the same minimization but with a larger basis set (6-311G+(d,p)), a better match to ZEHFUR ($RMSD_{20}$ = 0.5886 Å) is obtained. It should be noted that this latter minimization was not conducted with the DB2020

repulsion-dispersion parameter set used during the PBE0/6-311G(d,p) minimization since this was developed for specific use with PBE0/6-311G(d,p). Instead, we use the set of repulsion-dispersion parameters applicable to PBE0/6-311+G(d,p) developed in (Bowskill, 2021) (referred to as DB2020+ below).

An attempt was made to elucidate the specific fault in the energy model causing this discrepancy. To begin, we consider if the intramolecular contribution may be erroneous when using the smaller basis set. Comparing the molecular conformation obtained at the respective minima for these two energy models (PBE0/6-311G(d,p) and PBE0/6-311+G(d,p)), the molecular conformations are quite similar with respect to each other ($RMSD_1$ = 0.1511 Å). Compared to the experimental conformation, the 6-311G(d,p) minimum structure is actually a closer conformational match ($RMSD_1$ = 0.1700 Å) than the conformation at the 6-311+G(d,p) minimum structure ($RMSD_1$ = 0.2507 Å). If we compare these $RMSD_1$ values with the 'good' experimental matches obtained in Refinement I (Table 9) for $ZEHGAY_{major}$ ($RMSD_1$ = 0.2226 Å and 0.0776 Å for the 24th and 5th ranked match respectively) and $ZEHGAY_{minor}$ ($RMSD_1$ = 0.1453 Å), the minimized ZEHFUR molecular conformations are still within a range of $RMSD_1$ values that could realistically give a good match to the experimental crystal structure. A visual inspection also supports that the differences in molecular conformation relative to the experimental structure appear relatively small in both cases (Figure 10). For these two reasons, it is doubtful whether the intramolecular contributions are the root cause of the different results obtained with the two energy models. Energy-wise, as observed from the 1-D torsion scans conducted in Step 2, the choice of basis-set size (6-311G(d,p) versus 6-311+G(d,p)) should have negligible effects on ΔU_{intra}. The ΔU_{intra} contribution at these two (conformationally similar) minima is consistent with this, wherein ΔU_{intra}. = +6.591 kJ/mol and ΔU_{intra}. = +6.879 kJ/mol at the 6-311G(d,p) and 6-311+G(d,p) minima respectively.

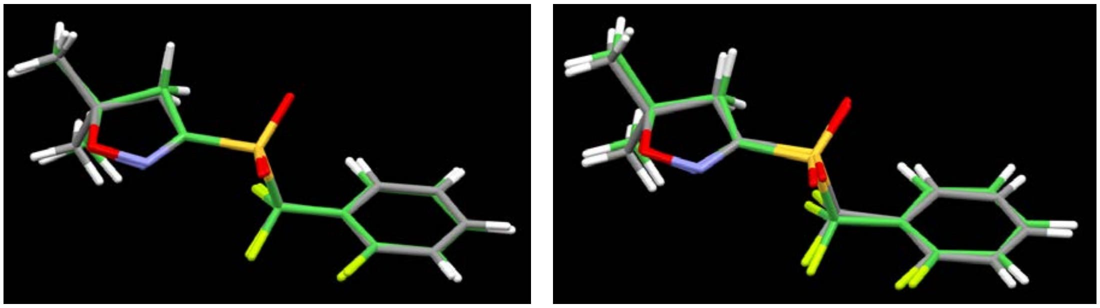

Figure 10: $RMSD_1$ comparison between the ZEHFUR experimental conformation and the conformations obtained at the minima when using PBE0/6-311G(d,p) (left) and PBE0/6-311+G(d,p) (right). The experimental conformation is given in grey whilst the minimised conformation is given in green.

The above observations suggest that the issue with the energy model may be related to the intermolecular contributions. To study this, a rigid minimization was conducted with the two energy models (PBE0/6-311G(d,p) with DB2020 and PBE0/6-311+G(d,p) with DB2020+), keeping the molecular conformation fixed at the experimental conformation. Doing so, both energy models predict a good match to the experimental structure, with $RMSD_{20}$ = 0.3183 Å and $RMSD_{20}$ = 0.2890 Å respectively.

From these findings, it would appear that the individual parts of the energy model are working well in isolation. In gas-phase molecule calculations, both models are in good agreement in terms of predicted ΔU_{intra}. When intramolecular effects are eliminated in the rigid minimization,

the intermolecular interactions correctly predict the experimental structure. However, our analysis only considers the final geometries/energies obtained at the minima in the rigid and flexible minimization, from which there appears to be little difference between the two models. It is likely that there is a more subtle interplay of both the intramolecular and intermolecular contributions, resulting in topological differences on the lattice-energy landscape when using PBE0/6-311G(d,p) with DB2020 and PBE0/6-311+G(d,p) with DB2020+.

In the context of a *blind* CSP study, it would be challenging to identify this sort of model inadequacy within our existing workflow. 1-D conformational scans can only pick up on gross errors between the predicted intramolecular properties, which as demonstrated here is insufficient. In the absence of experimental structures for comparison, there is also no opportunity to conduct flexible minimizations to assess the adequacy of the energy model. One avenue that could be considered in the early stages of CSP is the generation of several hypothetical crystal structures *in silico* using DFT-D. With these reference data, flexible minimizations may be used to assess the adequacy of the complete energy model (intra- + intermolecular components). Because our repulsion-dispersion parameters are parameterized against similar DFT-D reference data (Bowskill, 2021), such a validation study would be appropriate. In the event that the validation results prove unsatisfactory, it would be trivial to re-parameterize the repulsion-dispersion force-field with the hypothetical structures as additional reference data. A proof of concept for this workflow has been demonstrated in a previous work (Bowskill et al., 2020), albeit for a rigid molecule.

From a theoretical standpoint, diffuse basis functions are most useful for modelling systems with loosely bound electrons which can drift far from the atomic center, for instance anions (Cramer, 2004). Some studies, however, have suggested that diffuse basis functions can be highly beneficial to third-period species such as sulfur (Vandermeeren et al., 2007), especially when they are involved in highly polar bonds with oxygen. With that in mind, polarization functions are more likely to be beneficial for modelling Molecule XXXI. It is generally accepted that heavy atoms in the third-period of the periodic table should be modelled with *d*-type polarization functions (Cramer, 2004), especially if hypervalent atoms are present, such as in the sulfone group of Molecule XXXI. Whilst the PBE0/6-311G(d,p) model used in the blind test already includes one set of *d*-type polarization basis functions, it may be that this is insufficient for this particular system. Ultimately, it is unclear if the addition of the diffuse basis function is legitimately beneficial towards Molecule XXXI, or if it is fortuitously making up for an insufficiency in the polarization functions. A more thorough investigation into the nature of these heavier atoms (e.g., S and Cl) may be necessary to inform future modelling decisions with respect to the basis set requirements.

Absent ZEHGEC Structure

The ZEHGEC experimental structure was used to initiate a minimisation with an identical energy model to each stage of the original CSP study. In both energy models tested (‘Global Search’ and ‘Refinement I’) a stable minimum was found that matched the experimental structure (Table 8). Failure to identify this minimum during the Global Search is most likely because it belongs to the *R-3* space group, which is not explored extensively within our search (approximately 0.65% of all minimisations) due to its relative scarcity in the CSD. Even if it had been found in the Global Search, the ZEHGEC crystal structure exhibits an exceedingly high value of U_{latt} (+28.508 kJ/mol from the global minimum), meaning it would have been excluded from further refinement.

Analyzing the structure of ZEHGEC reveals a packing structure that is very sparse and low-density. In fact, there appear to be empty channels parallel to the *c*-axis of the unit cell (Figure 11). The low density of the structure explains its relatively high U_{latt} and is also suggestive that ZEHGEC could be a desolvated solvate.

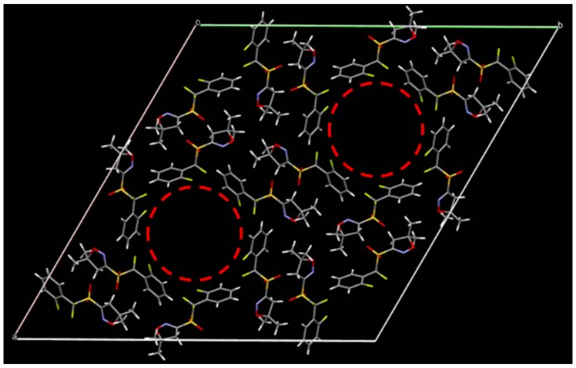

Figure 11: Crystal packing of ZEHGEC. The red dashed circles indicate the empty channels which run through the crystal structure along the c-axis.

2.3. Target XXXII (Isaac J. Sugden)

2.3.1. Step 1. Initial partitioning of conformational degrees of freedom

Through a combination of the default scanning process and chemical intuition, the 11 independent degrees of freedom shown in Figure 11 were identified.

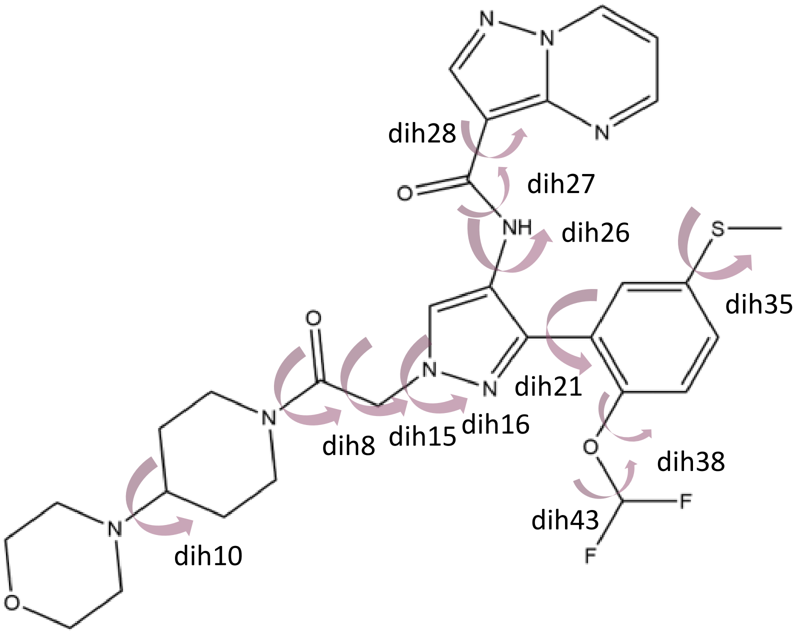

Figure 12: Independent degrees of freedom in Target XXXII

2.3.2. Step 2: Screen basis sets using conformational scans

Several levels of theory (PBE, PBE0, HF, B3LYP) were tested through scans of each torsion, with no combination offering a significant enough accuracy or cost advantage to justify deviating from PBE0/6-311G(d,p), which was the level of theory used in the parameterisation of the intermolecular repulsion/dispersion parameters. An example is shown in Figure 13, for dih16. The energy profiles for all torsions, for the selected level of theory, PBE0/6-311G(d,p), are shown in Figure 14, Figure 15 and Figure 16.

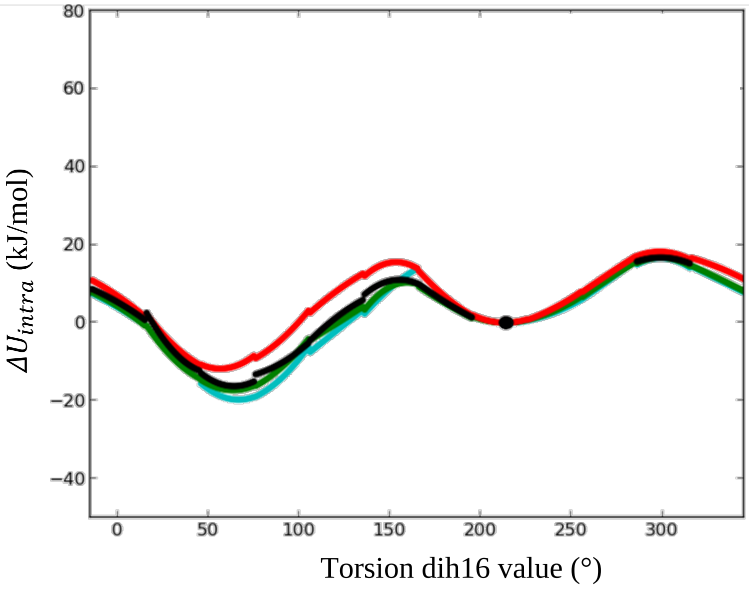

Figure 13: Torsion energy profile for dih16 in Target XXXII. Cyan is PBE, green is PBE0, red is HF, and black is B3LYP. The black dot is the minimum value in B3LYP, from the ChemDraw drawn molecule.

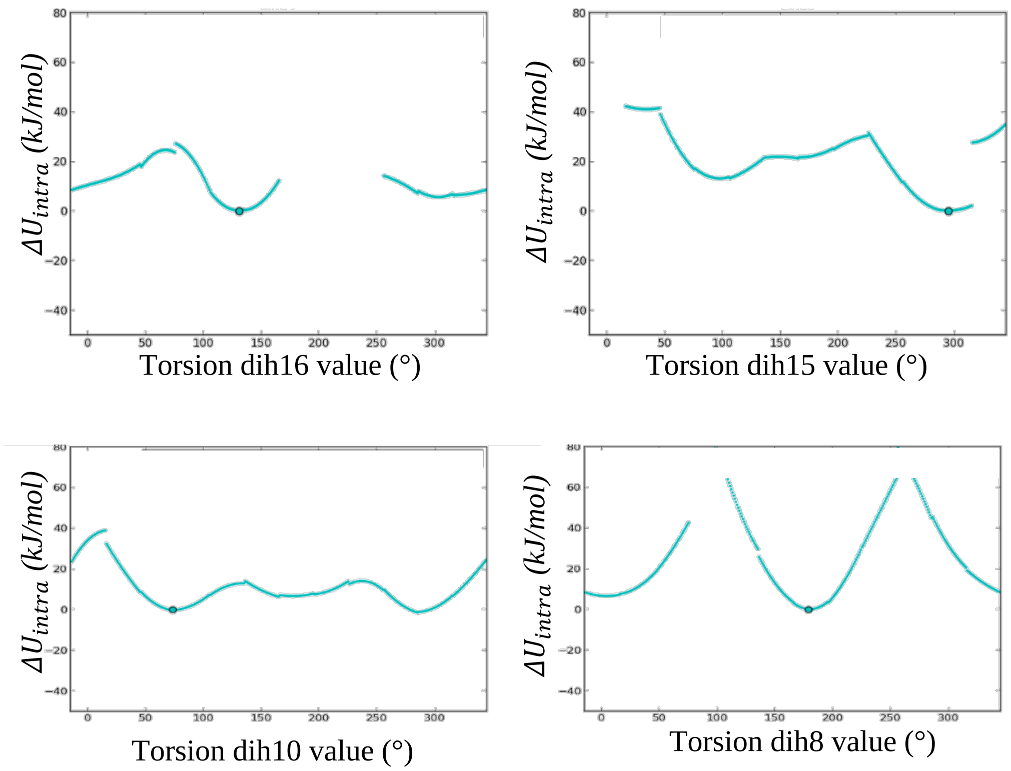

Figure 14: Torsion energy profiles for dih16, dih15, dih10 and dih8 in Target XXXII, obtained using PBE0/6-311G(d,p)

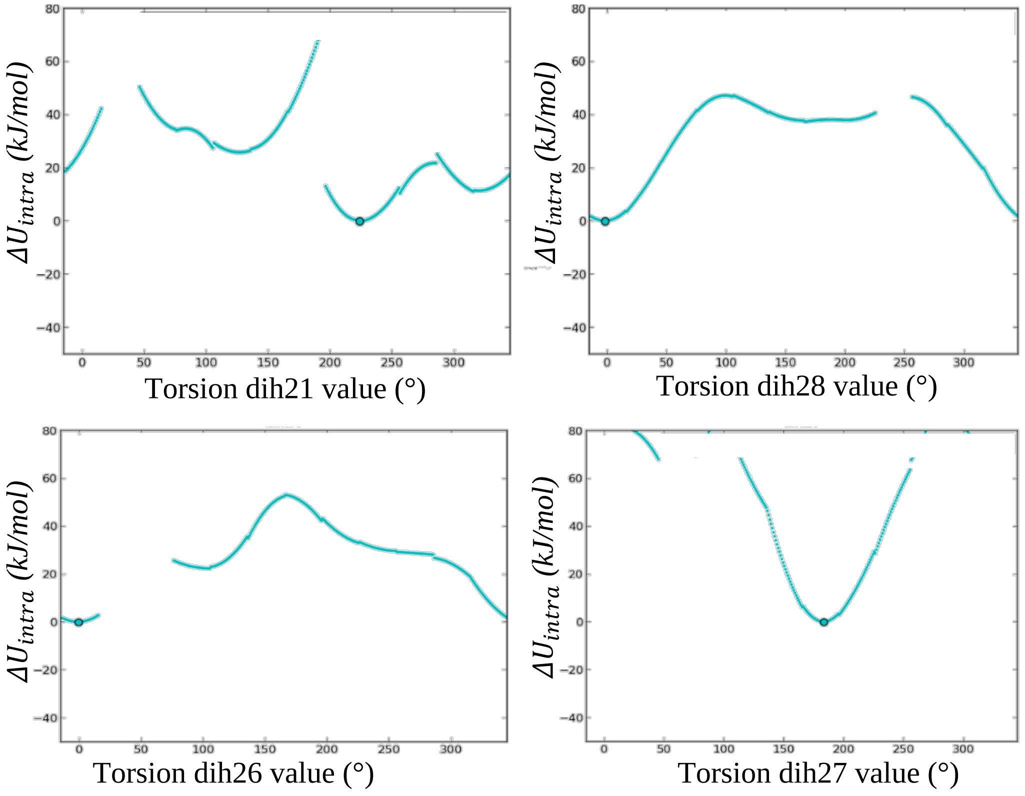

Figure 15: Torsion energy profiles for dih21, dih28, dih26 and dih27 in Target XXXII, obtained using PBE0/6-311G(d,p)

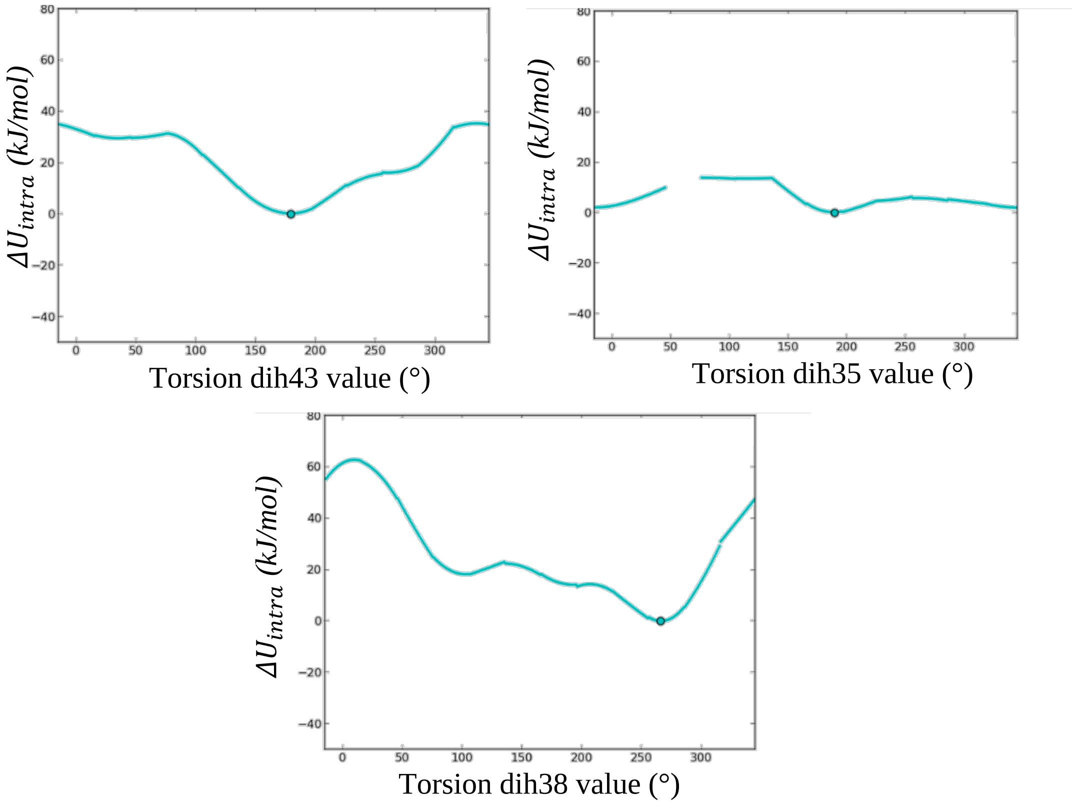

Figure 16: Torsion energy profiles for dih43, dih35 and dih38 in Target XXXII, obtained using PBE0/6-311G(d,p)

**Torsional Group**	**Torsion**	**Value *in vacuo* (º)**	**Flexibility Range**		
			**From (º)**	**To (º)**	**Initial Grid Points (º)**
1	Dih8	178.5402	0	360	[60,300,120]
1	Dih10	73.5742	0	360	[60,300,120]
1	Dih15	295.5416	0	360	[60,300,120]
1	Dih16	131.7179	-60	300	[0,240,120]
2	Dih21	-136.734	-60	300	[0,240,120]
2	Dih26	-0.9556	-60	60	[0,0,120]
2	Dih27	-177.335	120	240	[180,180,120]
2	Dih28	-2.4311	-60	60	[0,0,120]
2	Dih35	189.6541	0	360	[60,300,120]
2	Dih38	264.9522	0	360	[60,300,120]
2	Dih43	184.0758	0	360	[60,300,120]

Given the number of degrees of freedom, it was decided to separate the torsions into two groups, in order to reduce the computational effort. This is analogous to the approach developed for CrystalPredictor I (Karamertzanis & Pantelides, 2007) but extended for CrystalPredictor II (Kazantsev et al., 2010, Habgood et al., 2015, Sugden et al., 2016, Sugden et al., 2019, Sudgen et al., in preparation), which offers advantages in terms of accuracy, as well as coverage, through the use of LAMs. However, even with this separation, it was not possible to cover the 0-360° range for all torsions with the available computational resources. Approximations were made in the cases of dih26, dih27 and dih28, with the ranges fixed to ±60°, to the approximate gas phase value. As can be seen in Figure 15, with the initially generated conformation, these torsions exist in deep energy wells, with barriers of over 20 kJ/mol for rotation beyond 60°.

2.3.3. Step 3: Compare model results to any known experimental data

No experimental data was known at the time of the CSP study, hence this step was skipped.

2.3.4. Step 4: If required, perform CSP on similar system(s)

This is not applicable to this system and was skipped.

2.3.5. Step 5: LAM database generation

The adaptive LAM algorithm converged using settings as follows: a maximum LAM boundary difference of 5 kJ/mol, and a maximum energy cut-off of 20 kJ/mol. This resulted in the generation of 7221 LAMs for group 1, and 1763 LAMs for group 2.

2.3.6. Step 6: Global search and clustering

Eight hundred and thirty thousand minimisations were performed in Z'=1 using CrystalPredictor v2.4.4, using standard space groups (63 most common), and the DB2020_PBE0_6-31G(d,p)_pc parameter set (Bowskill, 2021). A cut-off for refinement of 120 kJ/mol was chosen, in order to generate at least 2000 structures, as the landscape was very sparsely populated around the global minimum. Upon closer inspection, it was revealed that there were a significant number of structures where torsional group 1 had rotated into the geometric space occupied by atoms of torsional group 2, resulting in clearly invalid conformations. A key rule of the torsional group approximation is that the approximation is only valid if atoms in the separate groups are significantly geometrically separated. This is clearly not the case, and suggests that the algorithm was spending significant effort in invalid regions of conformational space, meaning that the global search is far from converged. The results are summarised in Table 10 and Figure 17.

Table 10 - Key information regarding the global search of Target XXXII.

No. of Minimisations Conducted	830,000
No. of Unique Structures Post-clustering	4888
Energy Cut-off (kJ/mol)	120.0
Unique Structures within Cut-off	2000

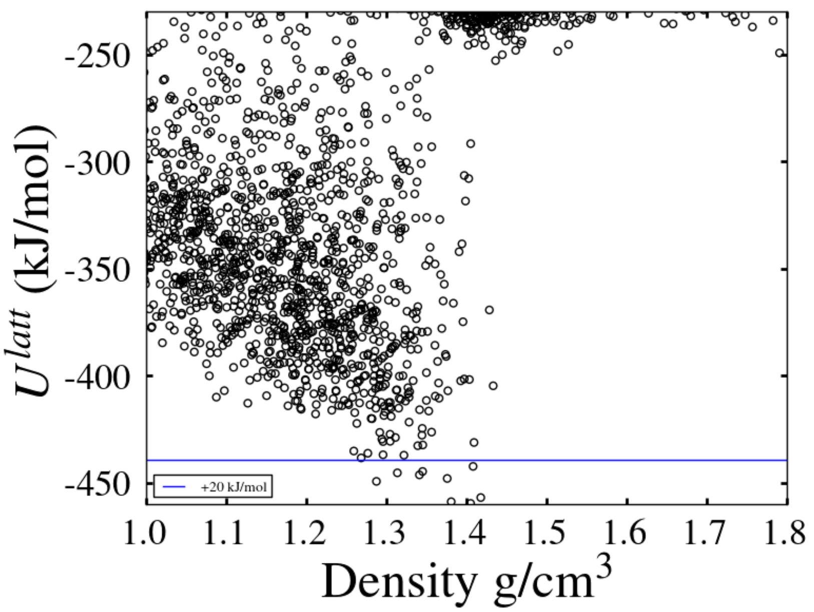

Figure 17: The crystal structure landscape for the (incomplete) Z' = 1 global search conducted with CrystalPredictor v2.4.4 for Target XXXII. The blue indicates the 20 kJ/mol.

2.3.7. Step 7: Refinement stage I and clustering

Approximately 1,500 structures were refined using CSO_FM V1.3, and the DB2020_PBE0_6-31G(d,p)_mp parameter set. The same level of theory and degrees of freedom were used as in the global search. After clustering using the CSD python API this resulted in 1,364 unique structures. An additional 135 structures were taken from the unrefined global search structures and included in the blind test submission, as time had run out to complete the refinement. The results are summarised in Table 11 and Figure 18.

Table 11 - Key information regarding the refinement stage of Target XXXII

No. of Structures Refined	1,499
No. of Unique Structures Post-clustering	1,364
Global Minimum Energy (kJ/mol)	-238.16

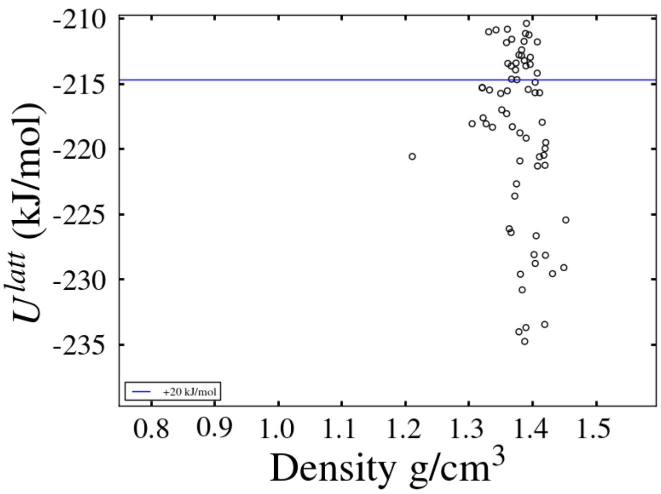

Figure 18: The partial crystal structure landscape for the Z' = 1 refinement conducted with CSO-FM for Target XXXII

2.3.8. Step 8: DFT-D refinement stage II

Not applicable

2.3.9. Discussion

There were two experimental targets for this system. JEKVOO is a Z'=2 structure, and thus was not explicitly searched for in our study. JEKVII is Z'=1, and exhibits disorder on the difluoromethyl group, which was not explicitly modelled. However, the major and minor components can be expected to be found in an exhaustive Z'=1 search, and no matches were found within the landscape. It was ascertained that the reason for these structures being missed in the search is that the molecule adopts a conformation outside of the ranges of flexible torsions searched for in this investigation, as shown in Table 12 (the disordered components are differentiated by differences in dih43, which was sampled in a 360° range).

Table 12. Key torsions in the JEKVII experimental structure and the ranges sampled in the investigation of Target XXXII

**Torsion**	**Exp value (º)**	**Lower bound (º)**	**Upper bound (º)**
**Dih26**	69	-60	60
**Dih27**	53	120	240
**Dih28**	116	-60	60

As detailed in Section 1.1.2, to make the generation of LAM databases manageable in a reasonable amount of time, the ranges of the three torsions listed in were truncated to ±60° around the gas phase minimum of the initially-generated conformation. This was justified on the basis that the one-dimensional energy profiles, with all other torsions fixed at their minimum values, showed deep energy wells (> 20kJ/mol) around the gas phase value. However, when flexibility is allowed in the other torsions (i.e. the molecule adapts its conformation to maintain favourable intramolecular interactions as the investigated torsion

rotates), the intramolecular energy cost is small enough to allow those torsion values within a viable crystal structure. In future work, efforts to allow 360° rotations will be considered.

2.4. Target XXXIII (Leticia Sanders De Almada)

2.4.1. Step 1. Initial partitioning of conformational degrees of freedom

Initially, an isolated gas-phase minimisations of the two components of Target XXXIII were carried out *in vacuo*, considering the isolated anion and the isolated cation separately, and using PBE0/6-311G(d,p). The independent degrees of freedom were chosen through analysis of the first-order and second-order derivatives of each torsion within the ions. The cation was considered rigid, and the anion exhibited four torsions to be considered flexible (dih9, dih10 and dih22) as shown in Figure 19.

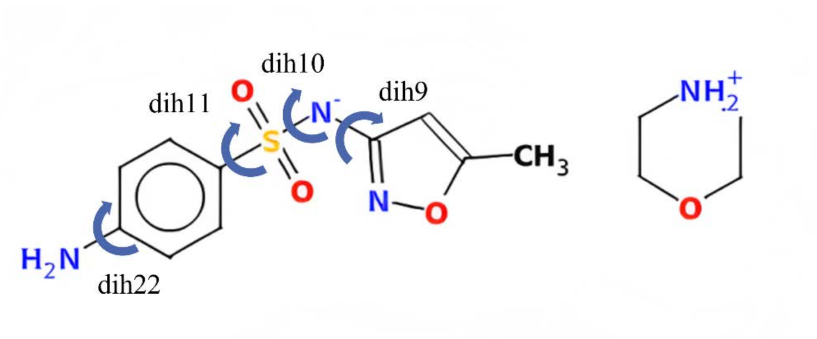

Figure 19: Flexible torsions in Target XXXIII found from Step 1 analysis

2.4.2. Step 2: Screen basis sets using conformational scans

One-dimensional scans of three of the four independent degrees of freedom (dih9, dih10, dih11) of the anion were carried out using PBE0 as the level of theory with three different basis sets: 6-31G(d,p), 6-311G(d,p), 6-311+G(d,p). The scans are shown in Figure 20. The change in intramolecular energy as a function of torsion values is similar for all three basis sets. Therefore, the choice of basis set was based on computational cost. The average cost for finding the optimal structure for 6-31G(d,p), 6-311G(d,p), 6-311+G(d,p) was, respectively, 16.5 CPU hours, 21.4 CPU hours and 50.6 CPU hours. The small increase from 6-31G(d,p) to 6-311G(d,p) meant that 6-311G(d,p) could be used without significant costs, while the more complete 6-311+G(d,p) would have more than double the cost while not showing significant changes in the 1-D scans.

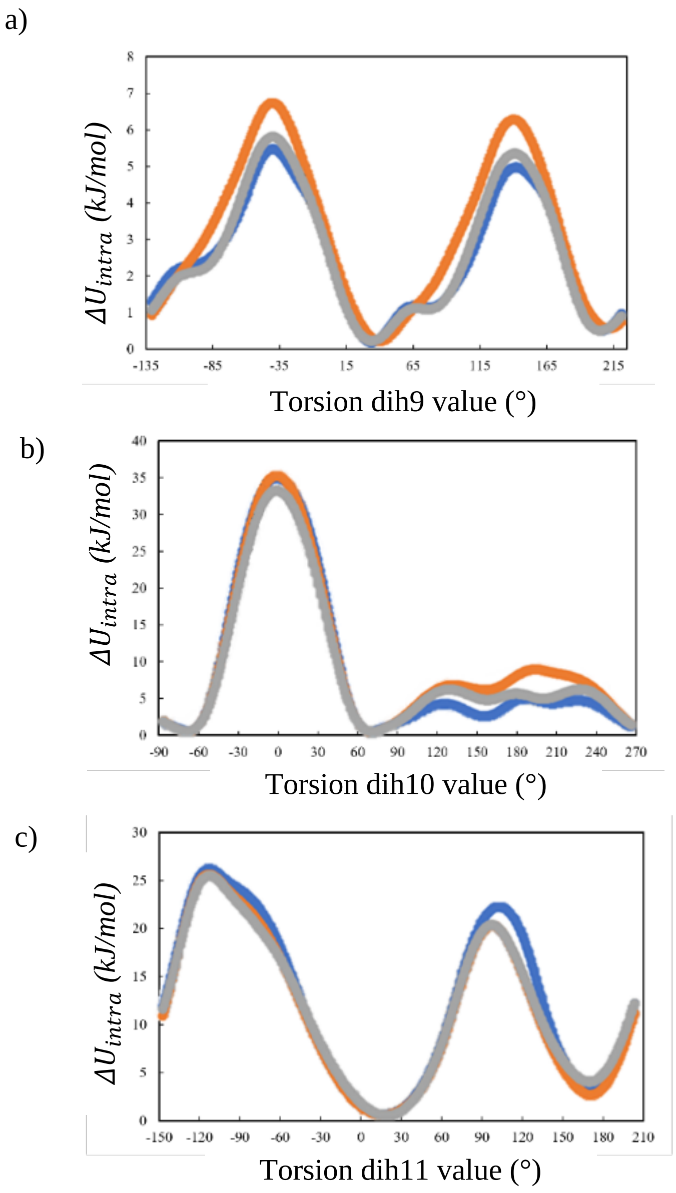

Figure 20: 1-D scans of the anion in Target XXXIII for three of the flexible torsions identified in Step 1 for a) dih9, b) dih10 and c) dih11. All scans were conducted at the PBE0 level of theory. The basis sets tested include 6-31G(d,p) (blue), 6-311G(d,p) (orange), and 6-311G+(d,p) (grey).

2.4.3. Step 3: Compare model results to any known experimental data

No experimental data was available for Target XXXIII.

2.4.4. Step 4: If required, perform CSP on similar system(s)

One system found in the CSD was evaluated, DEVGOA, which has one known experimental form (Figure 21). DEVGOA contains the same cation as Target XXXIII. It does not contain

rings, but it presents a negative charge on a nitrogen connected to a sulfone group, similarly to the anion in Target XXXIII.

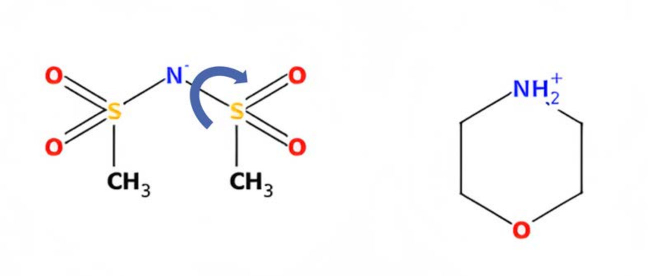

Figure 21: Salt system tested, DEVGOA, with the independent degree of freedom indicated by an arrow

Analysis of first and second-order derivatives around the gas phase optimal values indicated that DEVGOA has one independent degree of freedom, as shown in Figure 21. The experimental structure was minimised using *CrystalOptimizer* with PBE0 as the level of theory and three basis sets: 6-31G(d,p), 6-311G(d,p), 6-311+G(d,p). The level of theory PBE0/6-311G(d,p) with a PCM correction (ε=11) was also tested, and two sets of dispersion/repulsion parameters were investigated: FIT (Coombes et al., 1996) and parameters developed by David Bowskill (Bowskill, 2021) for each level of theory (denominated DB2020). For DEVGOA, local minimisations from the experimental crystal structure resulted in a match with an $RMSD_{15}$ value less than 0.3Å for most models, as indicated in Table 13.

Table 13: Results of local minimisations of experimental structure of DEVGOA. Here the $RMSD_{15}$ and $RMSD_{1}$ between the experimental structure and the final optimised structure are shown. The $RMSD_{1}$ is applied to the anion only, as this is the only flexible component in the salt.

**Model used (parameter set – level of theory/basis set)**	**RMSD15 (Å)**	**RMSD1 (Å)**
FIT - PBE0/6-31G(d,p)	0.278	0.022
FIT - PBE0/6-311G(d,p)	0.236	0.022
FIT - PBE0/6-311+G(d,p)	0.240	0.021
DB2020 - PBE0/6-31G(d,p)	0.223	0.022
DB2020 - PBE0/6-311G(d,p)	0.198	0.022
DB2020 - PBE0/6-311+G(d,p)	0.215	0.022
DB2020 - PBE0/6-311G(d,p) +PCM(ε=7)	0.143	0.021

Based on the local optimisation results, two global searches were carried out using the PBE0/6-311G(d,p) level of theory with the two basis sets, as this level of theory yielded the second best results while having a significantly lower computational costs compared to the models with PCM correction. The resulting landscapes after 300,000 minimisations are shown in Figure 22. The experimental form was found as the 9th lowest energy structure ($RMSD_{15}$ of 0.3 Å) for the FIT parameters and as the 3rd lowest energy structure for parameter set DB2020 ($RMSD_{15}$ of 0.2 Å). Refinement of the 1000 lowest energy structures using multipole electrostatics and the FIT parameters improved the rank from 9th to 4th. For the DB2020 parameter set, the ranking worsened slightly from 3rd to 4th.

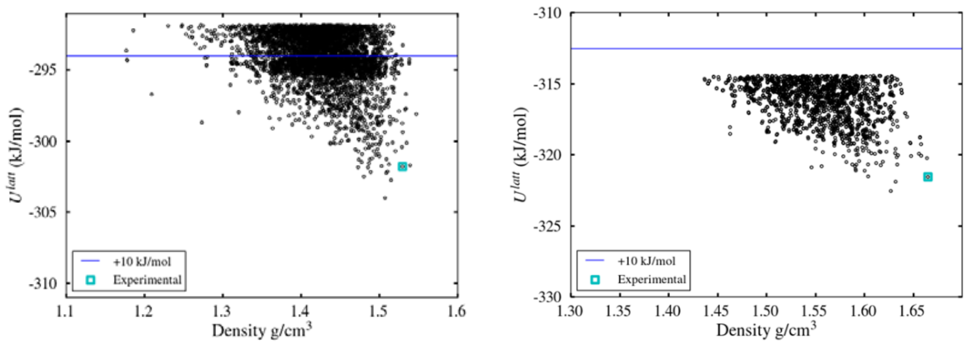

Figure 22: The crystal structure landscape for DEVGOA after global search using CrystalPredictor with a) FIT parameters and b) DB2020 parameters. Unique structures found post-clustering are given by the empty black circles. The cut-off for structures progressing to refinement is given by the blue line. Matches to the known experimental form are given by the blue squares.

Based on these results, the model DB2020 - PBE0/6-311G(d,p) was chosen for the global optimisation step of Target XXXIII, and a PCM correction was included for the refinement stage.

2.4.5. Step 5: LAM database generation

For the LAM database, it was assumed that dih9, dih10 and dih11 were flexible over a full 360° rotation, while dih22 was assumed to be flexible over 180°. With the level of theory PBE0/6-311G(d,p), the adaptive LAM algorithm, run with a maximum LAM boundary difference of 5 kJ/mol and a maximum energy cut-off of 30 kJ/mol, converged at 3,233 LAMs. As the cation was considered rigid, no LAMs were needed for this ion. A summary of the anion LAM database generation is shown in Table 14.

Table 14: Details of global search LAM generation for the anion in Target XXXIII.

Uniform Grid Interval (^{o})	60.0
No. of LAMs in Uniform Grid	864
Adaptive LAMs High-Energy Cut-off (kJ/mol)	30.0
Adaptive LAMs Accuracy Cut-off (kJ/mol)	5.0
No. of Adaptive LAMs Generated	2,639
Total LAM Database Size	3,233

2.4.6. Step 6: Global search and clustering

Global search was conducted for Z' = 1 (one cation and one anion in the asymmetric unit). As described in the LAM generation section, parameters developed by (Bowskill, 2021) were used (DB2020_PBE0_6-311G(d,p)_pc parameter set) and for this stage, point-charge electrostatics were used. One million minimisations performed using *CrystalPredictor V2.4.4*, using the 61 most common space groups, i.e., the 59 space groups listed in Section 1.1.2 as well as P2221 and PBA2. A cut-off for refinement of 16 kJ/mol was chosen, and following clustering with the CSD python API tools, this resulted in 1,406 low-energy

structures being identified. Table 15 shows a summary of the global search and Figure 24 shows the resulting landscape.

Table 15: Key information regarding the global search of Target XXXIII. The energy cut-offs are defined with respect to the global minimum from each individual search.

Z'	**1**
No. of Minimisations Conducted	1,000,000
Global Minimum Energy (kJ/mol)	-339.116
Energy Cut-off (kJ/mol)	16.0
Unique Clusters within Cut-off	1,406

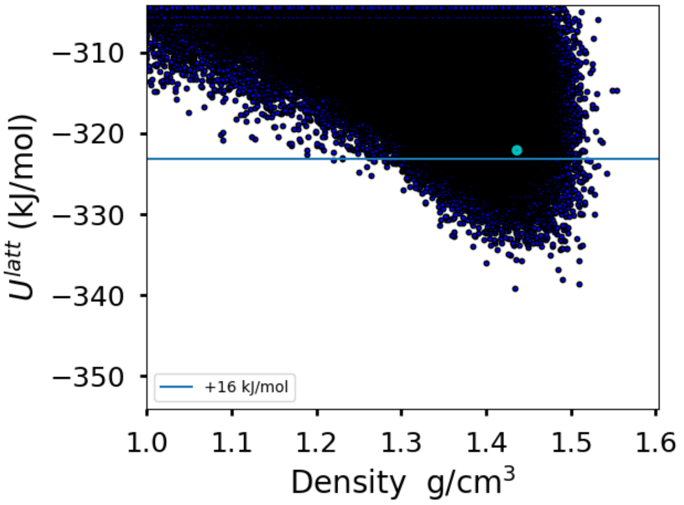

Figure 23: The crystal structure landscape for Target XXXIII after candidate search and global optimisation using CrystalPredictor with DB2020 parameters and PBE0/6-311G(d,p) level of theory. Unique structures found post-clustering are given by the empty black circles. The blue line indicates 16 kJ/mol from global minimum. The chosen cut-off was 16 kJ/mol due to computational limitations. The blue circle shows the position ZEGWER would take in the landscape if it had been found in the search. ZEGWAN is not shown as it is larger than the scale of lattice energy, being 37 kJ/mol above the global minimum.

One of the experimental forms, ZEGWER, was not found in the search, indicating that the search was not complete or that the model was not accurate enough. However, even if it was found, the energy would have been 20 kJ/mol above the global minimum, above the cut-off. ZEGWAN is not found in the search. However, it if is locally minimised using *CrystalPredictor* and the same model described, the resulting structure was found in the search, indicating that this structure could converge to ZEGWAN after refinement. However, this matching structure lied above the cut-off, 37 kJ/mol above the global minimum found.

2.4.7. Step 7: Refinement stage I and clustering

Due to practical computational reasons, the 1,058 lowest-energy structures from Step 6 were refined with local optimisation using multipoles electrostatics. Again, a parameter set developed by (Bowskill, 2021) was used for the level of theory PBE0/6-311G(d,p) with a PCM correction (ε=7). The set of independent degrees of freedom was expanded through the

addition of four bond angles and one torsion, which relates to the change in angle between the two hydrogens in the -NH_2 group, as indicated in Figure 24. After clustering using the CSD python API, this resulted in 1,048 Z'=1 structures, as shown in Figure 25.

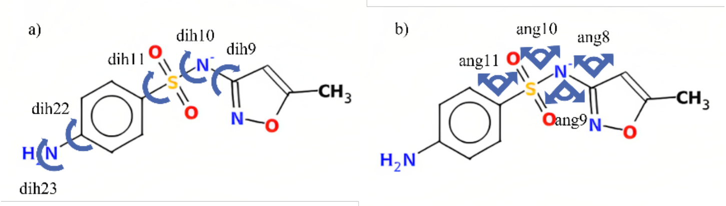

Figure 24: Independent degrees of freedom for the first refinement stage of Target XXXIII indicated by blue arrows: a) independent torsion angles; b) bond angles.

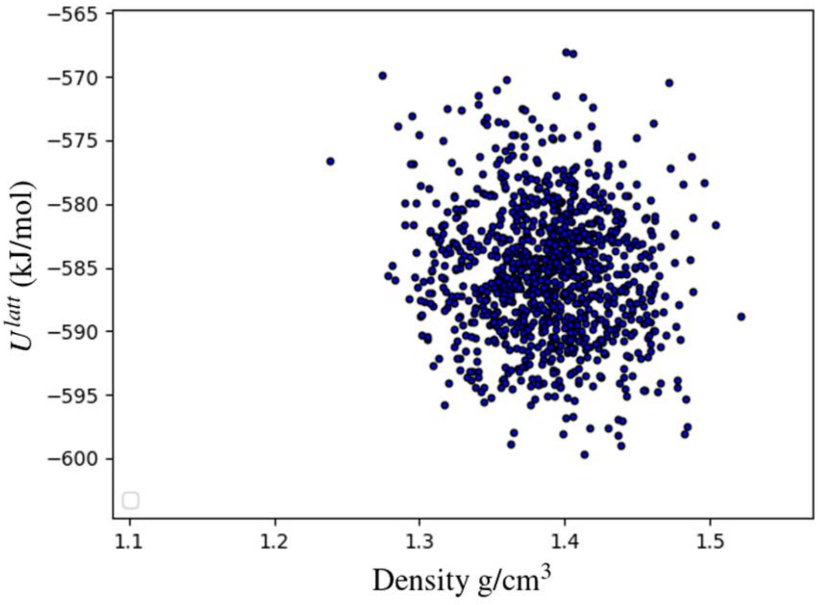

Figure 25: The crystal structure landscape for Target XXXIII after refinement sing CSO-FM with the DB2020 parameter set and PBE0/6-311G(d,p) level of theory with PCM correction. Unique structures found post-clustering are given by the blue circles.

2.4.8. Step 8: DFT-D refinement stage II

Periodic DFT optimisations were performed on the 102 lowest energy structures (approximately 7.8 kJ/mol of the global minimum) using the TPSS functional, PAW pseudopotentials, and the D3 dispersion correction, in VASP. None of the experimental structures were included in the first refinement stage and thus none of the structures considered converged to the experimental forms after the second refinement stage.

2.4.9. Discussion

None of the two experimental forms were found at the end of the CSP study. As mentioned in Step 6, ZEGWER was not found in the global search, indicating that the search was not complete. If it was found, it would have been 20 kJ/mol above the global minimum. ZEGWAN was found, but it was above the energy cut-off that was used, 37 kJ/mol above global minimum. Local minimisations of both experimental structures using the energy model, repulsion/dispersion parameters and LAM database used in Step 6 lead to poor matches between the computed structures and the original experimental structure, with fewer than 15 molecules matching, indicating that the energy model does not satisfactorily describe the interactions present in the crystal structure of Molecule XXXIII.

Several issues were encountered when trying to converge the isolated molecule quantum calculations for ZEGWAN and it was not possible to complete the refinement stage for the structure. A local minimisation of the experimental structure of ZEGWER was carried out using CSO-FM and the model described in Step 7. Despite the use of multipoles and the addition of a PCM correction (ε=7), the model still gives a poor match, with the optimised structure not matching a full 15-molecule cluster with the starting experimental structure. The optimised structure is 17 kJ/mol above the new global minimum, ranking as the 795th structure before clustering.

2.5. Computational resources used

The following resources were used to conduct the computations:

Default: Imperial HPC, AMD EPYC 7742 64-Core Processor or Intel(R) Xeon(R) CPU E5-2620 0 @ 2.00GHz Processor.

For some refinement steps, the following resources were adopted:

- Thomas (Intel(R) Xeon(R) CPU E5-2650 v4 @ 2.20GHz)
- Young (Intel(R) Xeon(R) Gold 6248 CPU @ 2.50GHz)
- Cirrus (18-core Intel Xeon E5-2695 (Broadwell) @2.1 GHz)

The CPU times for the four CSPs carried out are listed in Table 16.

Table 16: Approximate computational resource used for each CSP target considered in CPU hrs.

Target	XXIX	XXXI	XXXII	XXXIII
Steps 1-4. Modelling choices	50	2,000	1,000	1,000
Step 5. LAM generation	425	103,000	415,000	70,000
Step 6. Global search	2,020	14,500	31,000	11,000
Step 7. Refinement I	650,000	76,600	1,150,000	130,000
Step 8. Refinement II	N/A	N/A	N/A	N/A
Total	653,000	196,199	1,597,000	412,000

3. Acknowledgments

Funding for this research was provided by: Engineering and Physical Sciences Research Council (grant Nos. EP/J014958/1, EP/J003840/1, EP/P022561/1, EP/P020194, and

EP/T51780X/1), Eli Lilly and Company and Syngenta. We would like to acknowledge the Imperial College Research Computing Service, DOI: 10.14469/hpc/2232, the Cirrus UK National Tier-2 HPC Service at EPCC (http://www.cirrus.ac.uk) funded by the University of Edinburgh and EPSRC (EP/P020267/1), and the UK Materials and Molecular Modelling Hub for computational resources, which is partially funded by EPSRC (EP/P020194/1 and EP/T022213/1)

4. Bibliography

Bayer, T. & Price, S. L., 2000. Dimer or catemer? Low-energy crystal packings for small carboxylic acids. *The Journal of Physical Chemistry B,* pp. 104(12), pp.2647-2655..

Bowskill, D. H., 2021. Reliable and Efficient Parameter Estimation Methodologies for Crystal Structure Prediction. *Imperial College London, PhD Thesis.*

Bowskill, D. H. et al., 2020. *Efficient Parameterization of a Surrogate Model of Molecular Interactions in Crystals.* Milano, Elsevier B.V., pp. 493-498.

Brandenburg, J. G. & Grimme, S., 2016. Organic crystal polymorphism: a benchmark for dispersion-corrected mean-field electronic structure methods. *Acta Crystallographica Section B: Structural Science, Crystal Engineering and Materials,* pp. 72(4), pp.502-513.

Coombes, D. S., Price, S. L., Willock, D. J. & Leslie, M., 1996. Role of electrostatic interactions in determining the crystal structures of polar organic molecules. A distributed multipole study.. *The Journal of Physical Chemistry,* pp. 100(18), pp.7352-7360.

Cox, S. R., Hsu, L. Y. & Williams, D. E., 1981. Nonbonded potential function models for crystalline oxohydrocarbons.. *Acta Crystallographica Section A: Crystal Physics, Diffraction, Theoretical and General Crystallography,* pp. 37(3), pp.293-301.

Cramer, C. J., 2004. Ab Initio Implementations of Hartree–Fock Molecular Orbital Theory. In: *Essentials of Computational Chemistry.* 2nd ed. s.l.:John Wiley & Sons, pp. 165-202.

Cruz-Cabeza, A. J. & Bernstein, J., 2014. Conformational polymorphism.. *Chemical reviews,* pp. 114(4), pp.2170-2191.

Frisch, M. J. et al., 2016. *Gaussian 09, Revision A.02.* Wallingford CT: Gaussian, Inc..

Habgood, M. et al., 2015. Efficient handling of molecular flexibility in ab initio generation of crystal structures.. *Journal of chemical theory and computation,,* pp. 11(4), 1957-1969.

Hafner, J., 2008. Ab-initio simulations of materials using VASP: Density-functional theory and beyond. *Journal of computational chemistry,* pp. 29(13), 2044-2078.

Hsu, L. Y. & Williams, D. E., 1980. Intermolecular potential-function models for crystalline perchlorohydrocarbons. *Acta Crystallographica Section A: Crystal Physics, Diffraction, Theoretical and General Crystallography,* pp. 36(2), pp.277-281.

Hu, H., Lu, Z. & Yang, W., 2007. Fitting molecular electrostatic potentials from quantum mechanical calculations. *Journal of chemical theory and computation,* pp. 3(3), 1004-1013.

Karamertzanis, P. G. & Pantelides, C. C., 2007. Ab initio crystal structure prediction. II. Flexible molecules. *Molecular Physics,* pp. 105(2-3), pp.273-291.

Karamertzanis, P. G. & Pantelides, C. C., 2007. Ab initio crystal structure prediction. II. Flexible molecules.. *Molecular Physics,* pp. 105(2-3), pp.273-291..

Kazantsev, A. V., Karamertzanis, P. G., Adjiman, C. S. & Pantelides, C. C., 2011. Efficient handling of molecular flexibility in lattice energy minimization of organic crystals.. *Journal of Chemical Theory and Computation,* pp. 7(6), 1998-2016.

Kazantsev, A. V., Karamertzanis, P. G., Adjiman, C. S. & Pantelides, C. S., 2011. Efficient handling of molecular flexibility in lattice energy minimization of organic crystals. *Journal of Chemical Theory and Computation,* pp. 7(6), pp.1998-2016.

Kazantsev, A. V., Karamertzanis, P. G., Pantelides, C. C. & Adjiman, C. S., 2010. Ab initio crystal structure prediction for flexible molecules. *Computer Aided Chemical Engineering,* pp. Vol. 28, pp. 817-822.

Nyman, J. & Day, G. M., 2015. Static and lattice vibrational energy differences between polymorphs. *CrystEngComm,* 17(28), pp. 5154-5165.

Pantelides, C. C., Adjiman, C. S. J. & Kazantsev, A. V., 2014. General computational algorithms for ab initio crystal structure prediction for organic molecules. *Prediction and Calculation of Crystal Structures,* pp. 25-58.

Price, S. L. et al., 2010. Modelling organic crystal structures using distributed multipole and polarizability-based model intermolecular potentials. *Physical Chemistry Chemical Physics,* pp. 12(30), 8478-8490.

Stone, A. J., 2005. Distributed multipole analysis: Stability for large basis sets. *Journal of Chemical Theory and Computation,* pp. 1(6), 1128-1132.

Stone, A. J., 2005. Distributed multipole analysis: Stability for large basis sets.. *Journal of Chemical Theory and Computation,* pp. 1(6), pp.1128-1132.

Sudgen, I. J. et al., in preparation. Accurate and efficient representation of intramolecular energy in ab initio generation of crystal structures. Part III: Torsional Group Partitioning. *in preparation.*

Sugden, I. J., Adjiman, C. S. & Pantelides, C. C., 2016. Accurate and efficient representation of intramolecular energy in ab initio generation of crystal structures. I. Adaptive local approximate models.. *Acta Crystallographica Section B: Structural Science, Crystal Engineering and Materials,* pp. 72(6), 864-874.

Sugden, I. J., Adjiman, C. S. & Pantelides, C. C., 2019. Accurate and efficient representation of intramolecular energy in ab initio generation of crystal structures. II. Smoothed

intramolecular potentials.. *Acta Crystallographica Section B: Structural Science, Crystal Engineering and Materials,* pp. 75(3), 423-433.

Vandermeeren, L., Leyssens, T. & Peeters, D., 2007. Theoretical study of the properties of sulfone and sulfoxide functional groups. *Journal of Molecular Structure: THEOCHEM,* pp. 1-8.

Williams, D. E. & Houpt, D. J., 1986. Fluorine nonbonded potential parameters derived from crystalline perfluorocarbons. *Acta Crystallographica Section B: Structural Science,* pp. 42(3), pp.286-295.

Williams, D. T. & Cox, S. R., 1984. Nonbonded potentials for azahydrocarbons: the importance of the Coulombic interaction.. *Acta Crystallographica Section B: Structural Science,* pp. 40(4), pp.404-417.

2. Group 3

Supplementary Information of Group 3 (DBoese) for Phase One

Alexander List[a], Nina Strasser[a], Johannes Hoja[a], Doris E. Braun[b], and A. Daniel Boese*[a]

[a] Department of Chemistry, University of Graz, Heinrichstrasse 28/IV, 8010 Graz, Austria
[b] Institute of Pharmacy, University of Innsbruck, Innrain 52c, 6020 Innsbruck, Austria

* Correspondence to: adrian_daniel.boese@uni-graz.at

In phase one, we attempted crystal structure predictions for target systems XXIX, XXXI, and XXXII.

1 General Procedure

1.1 Conformer Generation

The initial structure for the conformer generation was obtained by performing a B3LYP+D3(BJ)/def2-TZVPPD[1–10] geometry optimization using TURBOMOLE 7.4.1.[11] Conformers were then generated with COSMOConf 4.1,[12] utilizing TURBOMOLE 7.4.1, and employing the following hierarchical procedure:

1. Generation of up to 2000 conformers using Balloon 1.6.7[13,14]
2. All structures: PBE+D3(BJ)/def2-SV(P)[9,15,16] single point calculation
3. Top 1000 structures within 150 kcal/mol: PBE+D3(BJ)/def2-SV(P) geometry optimization
4. Top 500 structures within 100 kcal/mol: B3LYP+D3(BJ)/def2-SV(P) single point calculation
5. Top 300 structures within 75 kcal/mol: B3LYP+D3(BJ)/def2-SV(P) geometry optimization
6. Top 200 structures within 50 kcal/mol: B3LYP+D3(BJ)/def2-TZVP[9] geometry optimization
7. Top 100 structures within 40 kcal/mol: B3LYP+D3(BJ)/def2-TZVPPD geometry optimization

Clustering of the conformers was performed between the steps, for which conformers were assumed to be identical if their root mean square deviation (RMSD) was less than 0.1 Å. The conformer generation step was carried out in the gas phase as well as for several dielectric constants (2, 21, 78, 150, 999) using COSMO.[17] From the resulting set of structures, the important unique conformers were identified by further clustering with respect to the RMSD and visual inspection using Mercury 4.0.[18]

Prior to the crystallographic landscape generation, the conformers were optimized again with PBE0/6-31G(d,p)[19–24] within Gaussian 09 Revision A. 02.[25] For the so obtained XXIX and XXXI conformer geometries, PBE0/aug-cc-pVTZ[26–28] single-point calculations were performed to obtain the CHELPG atomic charges needed for subsequent force field optimizations of generated crystal structures.[29] For XXXII, PBE0/6-31G(d,p) single-point calculations were performed instead.

A total of 2 conformers for target XXIX, 11 conformers for target XXXI, and 115 conformers for target XXXII were taken to the landscape generation step.

1.2 Landscape Generation

The generation of the crystallographic landscape was performed using CrystalPredictor v.2.0.1.[30–32] Z' = 1 searches were carried out in 61 space groups (cell angles between 50° and 130°, XXIX and XXXI: cell lengths between 3 Å and 40 Å, XXXII: cell lengths between 3 Å and 65 Å, minimum cell density of 500 kg/m^3, maximum relative intermolecular energy of 30 kJ/mol, deformation parameter of 0.15), wherein the conformers were treated as rigid molecules. The included space groups are shown in Table S1. Additionally, Z' = 2 searches were carried out in the 18 following space groups for XXIX: $P\bar{1}$, $P2_1$, $P2_1/c$, $C2/c$, Cc, $C2$, Pc, Cm, $P2_1/m$, $C2/m$, $P2/c$, $P2_12_12$, $P2_12_12_1$, $Pna2_1$, $Pca2_1$, $Pbca$, $Pbcn$, $P222_1$. For the lattice energy optimization within CrystalPredictor the FIT exp-6 atom-atom potential and the CHELPG atomic charges were used.[33,34]

Table S1: Space groups for which searches in the crystallographic landscape were conducted.

Crystal system	Space groups used
Triclinic	$P1$, $P\bar{1}$
Monoclinic	$P2_1$, $P2_1/c$, $C2/c$, Cc, $C2$, Pc, Cm, $P2_1/m$, $C2/m$, $P2/c$
Orthorhombic	$P2221$, $P2_12_12$, $P2_12_12_1$, $Pna2_1$, $Pca2_1$, $Pbca$, $Pbcn$, $C222_1$, $Pmn2_1$, $Cmc2_1$, $Aba2$, $Fdd2$, $Iba2$, $Pnna$, $Pccn$, $Pbcm$, $Pnnm$, $Pmmn$, $Pnma$, $Pba2$, $Cmcm$, $Cmca$, $Fddd$, $Ibam$
Tetragonal	$P4_1$, $P4_3$, $I\bar{4}$, $P4/n$, $P4_2/n$, $I4/m$, $I4_1/a$, $P4_12_12$, $P4_32_12$, $P\bar{4}2_1c$, $I\bar{4}2d$
Trigonal	$P3_1$, $P3_2$, $R3$, $P\bar{3}$, $R\bar{3}$, $P3_121$, $P3_221$, $R3c$, $R\bar{3}c$,
Hexagonal	$P6_1$, $P6_3$, $P6_3/m$
Cubic	$P2_13$, $Pa\bar{3}$

A total of 10,000,000 crystal structures were generated for target XXIX, 6,500,000 for target XXXI, and 142,500,000 for target XXXII. The crystal structures were then clustered to remove duplicates using CrystalPredictor's internal method.

1.3 Re-optimization and Re-ranking

The structures obtained from CrystalPredictor were subject to rigid-molecule lattice energy minimization using DMACRYS v.2.3.0.[35] The atomic charges of the conformers were replaced with distributed multipoles, which were calculated with GDMA2 from the PBE0/aug-cc-pVTZ (XXIX and XXXI) and PBE0/6-31G(d,p) (XXXII) calculations.[36] The repulsion/dispersion term was described by the FIT exp-6 atom-atom potential.

The DMACRYS-structures with the lowest energy where then optimized with CrystalOptimizer,[37] allowing for molecular flexibility within the molecules by allowing rotation around the following single bonds:

- XXIX: C1-O1, O1-C2, C2-C3, N1-C8
- XXXI: S1-C1, C1-C2, S1-C8, C9-C11, C9-C12

- XXXII: C1-C4, C2-N3, N3-C12, C12-C13, S1-C6, C9-O1, O1-C11, N1-C19, C19-C20, C20-N7, C23-N8

PBE0/6-31G(d,p) was used for the isolated molecule constrained geometry optimizations and the calculation of the distributed multipoles (XXXI and XXXII). For the XXIX structures, PBE0/aug-cc-pVTZ was used for the calculation of the distributed multipoles. Identically to DMACRYS, the FIT exp-6 atom-atom potential was used.

After the DMACRYS and CrystalOptimizer minimization steps, duplicates were identified and removed with COMPACK[38] in the following way: for structures which differed by energy and volume less than 0.2 kJ/mol and 1 Å^3, respectively, their powder patterns were calculated ($2\theta_{min}$ = 5°, $2\theta_{max}$ = 40°, triangle parameter of 2.0) and compared. If the powder patterns were sufficiently similar (97 % agreement or higher), the $RMSD_{20}$ between the two crystal structures was calculated, for which the structures were assumed to be identical if all 20 molecules could be matched (distance tolerance of 20 %) and the $RMSD_{20}$ was less than 0.1 Å.

Next, for all remaining CrystalOptimizer as well as for additional structures from the DMACRYS optimization level, single points were calculated with PBE+MBD[39–41] (light species default settings) using FHI-aims[42–45] (version 210226) together with ASE version 3.21.1.[46] Convergence criteria were set to 10^{-6} eV, 10^{-3} eV, 10^{-5} electrons/Å^3, and 10^{-4} eV/Å for the total energy, sum of eigenvalues, charge density, and forces, respectively. The k-grid for the PBE calculations was determined in such a way that for each direction $k \cdot l \geq 10$ Å, where k is the number of k-points and l is the cell length in that direction. For the MBD calculations the k-points were determined according to $k_{MBD} \cdot l \geq 15$ Å.

The top structures were subsequently optimized with PBE+MBD/light, using the same settings for the SCF convergence criteria and the k-grids as described above, and ensuring that all forces were less than 0.05 eV/Å. Initially, no symmetry constraint was imposed, so that crystal structures could transition to a different space group when it was energetically favorable. The structures were then symmetrized and optimized again, this time with a symmetry constraint.

Finally, another (more accurate) PBE+MBD/light geometry optimization with a symmetry constraint was performed, but the k-grids were now determined according to $k \cdot l \geq 18$ Å (PBE) and $k_{MBD} \cdot l \geq 25$ Å (MBD) and the maximum value for the forces was set to 0.005 eV/Å.

A final clustering step was performed, wherein crystal structures were assumed to be identical if the difference in their energies was less than 1 kJ/mol and if the $RMSD_{20}$ between the structures was less than 0.15 Å (distance and angle tolerances of 20 %). The structures were symmetrized and converted to CIFs using pymatgen.[47] Table S2 provides an overview of the number of calculated structures and considered energy ranges at each level.

Table S2: Number of structures calculated at each step of the re-optimization and re-ranking procedure as well as the lattice energy range of the structures at the previous level in kJ/mol. For target XXIX, separate lattice energy ranges are given at the DMACRYS and CrystalOptimizer level for $Z' = 1$ and $Z' = 2$ structures, respectively.

Level	XXIX		XXXI		XXXII	
	No.	Range	No.	Range	No.	Range
DMACRYS opt.	106,473	16 / 7.5[a]	73,988	20-25[b]	123,624	30
CrystalOptimizer opt.	1,826	10 / 6[a]	8,072	20-30[b]	4,622	30
PBE+MBD/light sp. (red. conv. crit.)	37,701	25	38,535	30	5,739	30-55[b]
PBE+MBD/light opt. (red. conv. crit.)	-	-	2,490	18	1,678	34
PBE+MBD/light opt.	2,001	10	382	10	-	-

[a] $Z' = 1$ / $Z' = 2$; [b] Depending on the conformer.

2 Remarks to the Individual Targets

2.1 Target XXIX

2.1.1 Landscape generation

1,000,000 $Z' = 1$ and 9,000,000 $Z' = 2$ crystal structures were generated, giving a total of 10,000,000 crystal structures.

2.1.2 Re-optimization and re-ranking

The PBE+MBD/light optimization step with reduced convergence criteria was skipped. For the final clustering step a RMSD cutoff of 0.2 Å was applied instead of 0.15 Å.

2.2 Target XXXI

2.2.1 Conformer generation

The conformer generation procedure with dielectric constants of 150 and 999 was omitted.

An additional set of conformers was obtained by randomly adsorbing the previously generated conformers on a periodic graphene sheet at a distance of 3.0 Å and optimizing them using density functional tight binding (DFTB3+TS[48]) within DFTB+.[49] For each conformer, 25 of such arrangements were created. The resulting conformers which – on the surface – were less than 20 kJ/mol higher in energy than the surface structure of lowest energy were checked for uniqueness by the means of RMSD values and visual inspection. During the subsequent optimization with PBE0/6-31G(d,p), these conformer geometries were partially frozen. However, the conformers generated near the graphene sheet did not contribute to any meaningful crystal structures later on.

A total of 11 conformers were taken to the landscape generation step – 8 from the standard procedure and 3 surface conformers.

2.2.2 Re-optimization and re-ranking

As less than 1500 crystal structures were calculated at the last step, the crystal structures of the last two optimizations steps were combined, duplicates removed, and the top 1500 structures were included in the submitted landscape.

2.3 Target XXXII

2.3.1 Conformer generation

Step 7 of the conformer generation procedure was omitted.

As the number of conformers was too large, additional clustering steps were performed. The following conformers were taken to the landscape generation step: (1) conformers with a RMSD value larger than 1 Å, (2) conformers with RMSD values between 0.5 and 0.8 Å were retained in case rotations around the bonds O1-C9, S1-C6, or C12-C13 were greater than 90°, around the O1-C11 bond greater than 120°, or around the N8-C23 bond greater than 180°. Only the lower energy conformer was considered for conformers with RMSD values below 0.5 Å and rotations around the S1-C10 bond were ignored completely.

2.3.2 Re-optimization and re-ranking

No symmetry constraint was applied during the second to last optimization level and the final, fully converged geometry optimization with PBE+MBD/light was omitted. The final clustering step was omitted as well.

3 Experimentally Assisted Challenge – Target XXIX

The choice of the 10 most likely structures was based on energy ranking and powder pattern similarity matching.

3.1 Energy Ranking

PBE+MBD/tight as well as PBE0+MBD/light single-point calculations were performed on top of the 2001 PBE+MBD/light-optimized structures obtained in phase one, and the final lattice energies of the structures were calculated by approximating the PBE0+MBD/tight level energies with the following scheme[50]:

$$E_{\text{PBE0+MBD/tight}} \approx E_{\text{PBE+MBD/tight}} + E_{\text{PBE0+MBD/light}} - E_{\text{PBE+MBD/light}}$$

Finally, for the top 117 structures, harmonic vibrational free energies (at 300 K) were calculated at the PBE+MBD/light level.

3.2 Powder Pattern Similarity Matching

The provided figure was converted to xy data using GetData Graph Digitizer.[51] The PXRD patterns for all structures provided in submission 1 were simulated using Mercury (FWHM peak shape: 0.1). AutoFIDEL v.0.4,[52] was used to compare the experimental and simulated data. No satisfactory match was identified.

3.3 Selection of 10 Most Likely Structures

The top 10 structures most likely to be represented by the experimental PXRD pattern were chosen based on a combination of the AutoFIDEL similarity scores, visual inspection and comparison of all patterns, and the ranking of the crystal structure based on their Helmholtz free energies.

4 Acknowledgments

The computational results presented have been achieved using the Vienna Scientific Cluster (VSC) as well as the HPC facilities at the University of Graz and the University of Innsbruck.

References

[1] P. A. M. Dirac, *Proc. R. Soc. London, Ser. A*, 1929, **123**, 714–733.

[2] J. C. Slater, *Phys. Rev.*, 1951, **81**, 385–390.

[3] A. D. Becke, *Phys. Rev. A*, 1988, **38**, 3098–3100.

[4] Lee, Yang and Parr, *Phys. Rev. B*, 1988, **37**, 785–789.

[5] A. D. Becke, *J. Chem. Phys.*, 1993, **98**, 5648–5652.

[6] S. Grimme, J. Antony, S. Ehrlich and H. Krieg, *J. Chem. Phys.*, 2010, **132**, 154104.

[7] A. D. Becke and E. R. Johnson, *J. Chem. Phys.*, 2006, **124**, 221101.

[8] S. Grimme, S. Ehrlich and L. Goerigk, *J. Comput. Chem.*, 2011, **32**, 1456–1465.

[9] F. Weigend and R. Ahlrichs, *Phys. Chem. Chem. Phys.*, 2005, **7**, 3297–3305.

[10] D. Rappoport and F. Furche, *J. Chem. Phys.*, 2010, **133**, 134105.

[11] *TURBOMOLE V7.4 2019, a development of University of Karlsruhe and Forschungszentrum Karlsruhe GmbH, 1989-2007, TURBOMOLE GmbH, since 2007; available from http://www.turbomole.com.*

[12] A. Klamt, F. Eckert and M. Diedenhofen, *J. Phys. Chem. B*, 2009, **113**, 4508–4510.

[13] M. J. Vainio and M. S. Johnson, *J. Chem. Inf. Model.*, 2007, **47**, 2462–2474.

[14] T. A. Halgren, *J. Comput. Chem.*, 1996, **17**, 490–519.

[15] Perdew, Burke and Ernzerhof, *Phys. Rev. Lett.*, 1996, **77**, 3865–3868.

[16] J. P. Perdew, K. Burke and M. Ernzerhof, *Phys. Rev. Lett.*, 1997, **78**, 1396.

[17] A. Klamt and G. Schüürmann, *J. Chem. Soc., Perkin Trans. 2*, 1993, 799–805.

[18] C. F. Macrae, I. Sovago, S. J. Cottrell, P. T. A. Galek, P. McCabe, E. Pidcock, M. Platings, G. P. Shields, J. S. Stevens, M. Towler and P. A. Wood, *J. Appl. Crystallogr.*, 2020, **53**, 226–235.

[19] C. Adamo and V. Barone, *J. Chem. Phys.*, 1999, **110**, 6158–6170.

[20] R. Ditchfield, W. J. Hehre and J. A. Pople, *J. Chem. Phys.*, 1971, **54**, 724–728.

[21] M. M. Francl, W. J. Pietro, W. J. Hehre, J. S. Binkley, M. S. Gordon, D. J. DeFrees and J. A. Pople, *J. Chem. Phys.*, 1982, **77**, 3654–3665.

[22] M. S. Gordon, J. S. Binkley, J. A. Pople, W. J. Pietro and W. J. Hehre, *J. Am. Chem. Soc.*, 1982, **104**, 2797–2803.

[23] P. C. Hariharan and J. A. Pople, *Theoret. Chim. Acta*, 1973, **28**, 213–222.

[24] W. J. Hehre, R. Ditchfield and J. A. Pople, *J. Chem. Phys.*, 1972, **56**, 2257–2261.

[25] *Gaussian 09, Revision A.02, Frisch, M. J., Trucks, G. W., Schlegel, H. B., Scuseria, G. E., Robb, M. A., Cheeseman, J. R., Scalmani, G., Barone, V., Mennucci, B., Petersson, G. A., Nakatsuji, H., Caricato, M., Li, X., Hratchian, H. P., Izmaylov, A. F., Bloino, J., Zheng, G., Sonnenberg, J. L., Hada, M., Ehara, M., Toyota, K., Fukuda, R., Hasegawa, J., Ishida, M., Nakajima, T., Honda, Y.,*

Kitao, O., Nakai, H., Vreven, T., Montgomery, J. A., Jr., Peralta, J. E., Ogliaro, F., Bearpark, M., Heyd, J. J., Brothers, E., Kudin, K. N., Staroverov, V. N., Kobayashi, R., Normand, J., Raghavachari, K., Rendell, A., Burant, J. C., Iyengar, S. S., Tomasi, J., Cossi, M., Rega, N., Millam, J. M., Klene, M., Knox, J. E., Cross, J. B., Bakken, V., Adamo, C., Jaramillo, J., Gomperts, R., Stratmann, R. E., Yazyev, O., Austin, A. J., Cammi, R., Pomelli, C., Ochterski, J. W., Martin, R. L., Morokuma, K., Zakrzewski, V. G., Voth, G. A., Salvador, P., Dannenberg, J. J., Dapprich, S., Daniels, A. D., Farkas, Ö., Foresman, J. B., Ortiz, J. V., Cioslowski, J., Fox, D. J. Gaussian, Inc., Wallingford CT, 2009.

[26] T. H. Dunning, *J. Chem. Phys.*, 1989, **90**, 1007–1023.

[27] R. A. Kendall, T. H. Dunning and R. J. Harrison, *J. Chem. Phys.*, 1992, **96**, 6796–6806.

[28] D. E. Woon and T. H. Dunning, *J. Chem. Phys.*, 1993, **98**, 1358–1371.

[29] C. M. Breneman and K. B. Wiberg, *J. Comput. Chem.*, 1990, **11**, 361–373.

[30] P. G. Karamertzanis and C. C. Pantelides, *J. Comput. Chem.*, 2005, **26**, 304–324.

[31] P. G. Karamertzanis and C. C. Pantelides, *Mol. Phys.*, 2007, **105**, 273–291.

[32] M. Habgood, I. J. Sugden, A. V. Kazantsev, C. S. Adjiman and C. C. Pantelides, *J. Chem. Theory Comput.*, 2015, **11**, 1957–1969.

[33] W. D. S. Motherwell, H. L. Ammon, J. D. Dunitz, A. Dzyabchenko, P. Erk, A. Gavezzotti, D. W. M. Hofmann, F. J. J. Leusen, J. P. M. Lommerse, W. T. M. Mooij, S. L. Price, H. Scheraga, B. Schweizer, M. U. Schmidt, B. P. van Eijck, P. Verwer and D. E. Williams, *Acta Crystallogr. Sect. B Struct. Sci.*, 2002, **58**, 647–661.

[34] D. S. Coombes, S. L. Price, D. J. Willock and M. Leslie, *J. Phys. Chem.*, 1996, **100**, 7352–7360.

[35] S. L. Price, M. Leslie, G. W. A. Welch, M. Habgood, L. S. Price, P. G. Karamertzanis and G. M. Day, *Phys. Chem. Chem. Phys.*, 2010, **12**, 8478–8490.

[36] A. J. Stone, *GDMA: A Program for Performing Distributed Multipole Analysis of Wave Functions Calculated using the Gaussian Program System, 2.2*, University of Cambridge: Cambridge, United Kingdom, 2010.

[37] A. V. Kazantsev, P. G. Karamertzanis, C. S. Adjiman and C. C. Pantelides, *J. Chem. Theory Comput.*, 2011, **7**, 1998–2016.

[38] J. A. Chisholm and S. Motherwell, *J. Appl. Crystallogr.*, 2005, **38**, 228–231.

[39] J. P. Perdew, K. Burke and M. Ernzerhof, *Phys. Rev. Lett.*, 1996, **77**, 3865–3868.

[40] A. Tkatchenko, R. A. DiStasio, R. Car and M. Scheffler, *Phys. Rev. Lett.*, 2012, **108**, 236402.

[41] A. Ambrosetti, A. M. Reilly, R. A. DiStasio and A. Tkatchenko, *J. Chem. Phys.*, 2014, **140**, 18A508.

[42] V. Blum, R. Gehrke, F. Hanke, P. Havu, V. Havu, X. Ren, K. Reuter and M. Scheffler, *Comput. Phys. Commun.*, 2009, **180**, 2175–2196.

[43] F. Knuth, C. Carbogno, V. Atalla, V. Blum and M. Scheffler, *Comput. Phys. Commun.*, 2015, **190**, 33–50.

[44] V. W. zhe Yu, F. Corsetti, A. García, W. P. Huhn, M. Jacquelin, W. Jia, B. Lange, L. Lin, J. Lu, W. Mi, A. Seifitokaldani, Á. Vázquez-Mayagoitia, C. Yang, H. Yang and V. Blum, *Comput. Phys. Commun.*, 2018, **222**, 267–285.

[45] V. Havu, V. Blum, P. Havu and M. Scheffler, *J. Comput. Phys.*, 2009, **228**, 8367–8379.

[46] A. Hjorth Larsen, J. Jørgen Mortensen, J. Blomqvist, I. E. Castelli, R. Christensen, M. Dułak, J. Friis, M. N. Groves, B. Hammer, C. Hargus, E. D. Hermes, P. C. Jennings, P. Bjerre Jensen, J. Kermode, J. R. Kitchin, E. Leonhard Kolsbjerg, J. Kubal, K. Kaasbjerg, S. Lysgaard, J. Bergmann Maronsson, T. Maxson, T. Olsen, L. Pastewka, A. Peterson, C. Rostgaard, J. Schiøtz, O. Schütt, M. Strange, K. S. Thygesen, T. Vegge, L. Vilhelmsen, M. Walter, Z. Zeng and K. W. Jacobsen, *J. Phys. Condens. Matter*, 2017, **29**, 273002.

[47] S. P. Ong, W. D. Richards, A. Jain, G. Hautier, M. Kocher, S. Cholia, D. Gunter, V. L. Chevrier, K. A. Persson and G. Ceder, *Comput. Mater. Sci.*, 2013, **68**, 314–319.

[48] A. Tkatchenko and M. Scheffler, *Phys. Rev. Lett.*, 2009, **102**, 073005.

[49] B. Hourahine, B. Aradi, V. Blum, F. Bonafé, A. Buccheri, C. Camacho, C. Cevallos, M. Y. Deshaye, T. Dumitrică, A. Dominguez, S. Ehlert, M. Elstner, T. van der Heide, J. Hermann, S. Irle, J. J. Kranz, C. Köhler, T. Kowalczyk, T. Kubař, I. S. Lee, V. Lutsker, R. J. Maurer, S. K. Min, I. Mitchell, C. Negre, T. A. Niehaus, A. M. N. Niklasson, A. J. Page, A. Pecchia, G. Penazzi, M. P. Persson, J. Řezáč, C. G. Sánchez, M. Sternberg, M. Stöhr, F. Stuckenberg, A. Tkatchenko, V. W.-Z. Yu and T. Frauenheim, *J. Chem. Phys.*, 2020, **152**, 124101.

[50] J. Hoja, H.-Y. Ko, M. A. Neumann, R. Car, R. A. DiStasio and A. Tkatchenko, *Sci. Adv.*, 2019, **5**, eaau3338.

[51] *GetData Graph Digitizer*, `http://getdata-graph-digitizer.com/`, Accessed: 2022-12-19.

[52] R. de Gelder, R. Wehrens and J. A. Hageman, *J. Comput. Chem.*, 2001, **22**, 273–289.

3. Group 5

Blind test methods

Graeme M. Day, Joseph E. Arnold, James Bramley, Patrick W. V. Butler, Ramon Cuadrado, Joseph Glover, Christopher R. Taylor

School of Chemistry, University of Southampton, Southampton, SO17 1BJ, United Kingdom

XXVII

Submission 1

Conformational sampling. Conformers were generated using low-mode sampling [1] with the Macromodel code and the OPLS-3 force field. These were re-optimised using Gaussian09 [2] with the PBE0 functional, GD3BJ dispersion correction and using 6-31G** basis for all atoms except iodine (I), which used the LANL2DZ basis with a core pseudopotential. Duplicate conformers were removed by comparison of all atom positions, with an RMSD limit of 0.3 Å.

Crystal structure generation: Crystal structures were generated and lattice energy minimised in the following space groups: 61, 14, 19, 2, 4, 15, 33, 9, 29, 5, 1, 60, 7, 18, 96, 76, 145, 43, 56, 13, 169, 88, 20, 86 and 148, all with Z`=1. Searches were performed for selected low energy conformers with Z`=2 in space groups : 1, 2, 33, 4, 14, 15, 19, 61 and 29.

Structure generation was performed using a quasi-random search, using the GLEE software [3].

Crystal structure optimisation: Two stages of optimisation were performed for each generated structure: rigid-molecule optimisation using the W99 force field [8] and atomic partial charges (fitted to the electrostatic potential generated from the full set of atomic multipoles); rigid-molecule optimisation using the W99 force field and atomic multipoles generated from a distributed multipole analysis of the DFT charge density. The iodine repulsion was in the force field was treated as anisotropic, based on parameters derived in the third blind test of crystal structure prediction.

Duplicate crystal structures were removed, first by clustering using comparison of simulated powder X-ray diffraction patterns, followed by clustering using the CSD API (with a 30-molecule cluster).

Structures for submission 1 were submitted based on their energy ranking after the second force field optimisation.

Submission 2

The set of structure provided for re-ranking were optimised using solid state DFT in the VASP [11] code. Calculations were performed with the PBE functional and a planewave basis set with pseudopotentials and GD3BJ dispersion correction. Optimisations were performed in three stages: fixed unit cell optimisation, followed by relaxed cell optimisation, both with a 500 eV planewave basis set cutoff. A final stage of optimisation was then performed with a 600 eV basis set cutoff and tighter convergence thresholds (PREC = Accurate, EDIFFG = -0.02). All calculations used a maximum electronic k-point spacing of 0.05 Å^{-1}.

XXVIII Calculations were stopped when it was revealed that the crystal structure has been reported.

XXIX

Conformational sampling. Conformers were generated using CREST [9] at the semi-empirical GFN2-xtb level of theory. These were re-optimised using Gaussian09 [2] with the PBE0 functional, GD3BJ dispersion correction and using 6-311G** basis set. Conformers were clustered manually, finding only two conformers.

Crystal structure generation: Crystal structures were generated and lattice energy minimised in the following space groups: 61, 14, 19, 2, 4, 15, 33, 9, 29, 5, 1, 60, 7, 18, 96, 76, 145, 43, 56, 13, 169, 88, 20, 86 and 148, all with Z`=1 using both conformers. Z`=2 searches were performed with all threecombinations of conformers in the following space groups: 1, 2, 33, 4, 14, 15, 19, 61 and 29. Further searchers were performed using distorted conformations generated by rotating torsion angles fromt eh gas phase minimised values.

Structure generation was performed using a quasi-random search, using the GLEE software [3]. All conformers were included in searching.

Crystal structure optimisation: Three stages of optimisation were performed for each generated structure: rigid-molecule optimisation using the FIT force field [5] and atomic partial charges (fitted to the electrostatic potential generated from the full set of atomic multipoles); rigid-molecule optimisation using the FIT force field and atomic multipoles generated from a distributed multipole analysis of the DFT charge density; fully-flexible tight binding DFT [7], using the DFTB 3ob parameter set ad D3 dispersion correction.

Duplicate crystal structures were removed, first by clustering using comparison of simulated powder X-ray diffraction patterns, followed by clustering using the CSD API (with a 30-molecule cluster). Clustering was performed prior to and after DFTB re-optimisation of structures.

Structures for submission 1 were submitted based on their energy ranking after DFTB. Best matches to the provided powder X-ray diffraction pattern were judged manually.

XXX

Conformational sampling . Conformers were generated using low-mode sampling [1] with the Macromodel code and the OPLS-3 force field. Separate searches were also run with CREST [9]. These lists were combined and unique conformers were re-optimised using Gaussian09 [2] with the PBE0 functional, GD3BJ dispersion correction and 6-311G(d,p) basis set. Duplicate conformers were removed by comparison of all atom positions, with an RMSD limit of 0.1 Å.

Crystal structure generation: Quasi-random structure generation [3] was performed with a series of rigid conformations in 2:1, 1:1 and 1:2 stoichiometries. For the larger component, 20 of the generated conformers were used, chosen as the 6 lowest energy conformers + 14 higher energy conformers with extended alkyl chains. To account for hydroxyl group flexibility, 8 OH orientations were sampled per conformer, so that a total of 160 starting molecular geometries were used. CSP in al stoichiometries was performed in space groups 1, 2, 19, 4, 61, 14 and 15.

Crystal structure optimisation: Four stages of optimisation were performed for each generated structure: rigid-molecule optimisation using the FIT force field [5] and atomic partial charges (fitted

to the electrostatic potential generated from the full set of atomic multipoles); rigid-molecule optimisation using the FIT force field and atomic multipoles generated from a distributed multipole analysis of the DFT charge density; fully-flexible tight binding DFT [7], using the DFTB 3ob parameter set ad D3 dispersion correction. Final energies were evaluated by reoptimizing all unique structures after DFTB with the FIT force field + multipoles, and taking the intramolecular energy from a DFT (PBE0/6-311G(d,p)) single-point energy.

Structures were ranked by their energy with respect to the stoichiometric sum of calculated energies for the pure component crystal structures. The pure component structures were obtained from the CSD and optimised with the same procedure as CSP structures.

XXXI

Submission 1

Conformational sampling. Conformers were generated using low-mode sampling [1] with the Macromodel code and the OPLS-3 force field. These were re-optimised using Gaussian09 [2] with the PBE0 functional, GD3BJ dispersion correction and 6-311G(d,p) basis set. Duplicate conformers were removed by comparison of all atom positions, with an RMSD limit of 0.1 Å. This resulted in 10 distinct conformers in the gas phase.

Crystal structure generation: Crystal structures were generated and lattice energy minimised in the following space groups: 61, 14, 19, 2, 4, 15, 33, 9, 29, 5, 1, 60, 7, 18, 96, 76, 145, 43, 56, 13, 169, 88, 20, 86 and 148, all with Z`=1. Z`=2 searches were performed with all combinations of conformers in the following space groups: 1, 2, 33, 4, 14, 15, 19, 61 and 29.

Structure generation was performed using a quasi-random search, using the GLEE software [3]. All conformers were included in searching. In addition, CSP was performed (Z`=1 only) using a set of conformations generated by distorting the gas phase conformers. 125 distorted conformations were generated by distorting the three flexible torsions by +/- 72 degrees and +/- 144 degrees from their phase geometries.

Crystal structure optimisation: Three stages of optimisation were performed for each generated structure: rigid-molecule optimisation using the FIT force field [5] and atomic partial charges (fitted to the electrostatic potential generated from the full set of atomic multipoles); rigid-molecule optimisation using the FIT force field and atomic multipoles generated from a distributed multipole analysis of the DFT charge density; fully-flexible tight binding DFT [7], using the DFTB 3ob parameter set ad D3 dispersion correction.

Duplicate crystal structures were removed, first by clustering using comparison of simulated powder X-ray diffraction patterns, followed by clustering using the CSD API (with a 30-molecule cluster). Clustering was performed prior to and after DFTB re-optimisation of structures.

Structures for submission 1 were submitted based on their energy ranking after DFTB.

Submission 2

The set of structure provided for re-ranking were optimised using solid state DFT in the VASP [11] code. Calculations were performed with the PBE functional and a planewave basis set with pseudopotentials and GD3BJ dispersion correction. Optimisations were performed in

three stages: fixed unit cell optimisation, followed by relaxed cell optimisation, both with a 500 eV planewave basis set cutoff. A final stage of optimisation was then performed with a 600 eV basis set cutoff and tighter convergence thresholds (PREC = Accurate, EDIFFG = -0.02). All calculations used a maximum electronic k-point spacing of 0.05 Å^{-1}.

XXXII

Submission 1

Conformational sampling. Conformers were generated using low-mode sampling [1] with the Macromodel code and the OPLS-3 force field. These were re-optimised using Gaussian09 [2] with the PBE0 functional, GD3BJ dispersion correction and 6-31G(d,p) basis set. Duplicate conformers were removed by comparison of all atom positions, with an RMSD limit of 0.1 Å. This resulted in 4089 distinct conformers in the gas phase.

Crystal structure generation: Crystal structures were generated and lattice energy minimised in the following space groups: 61, 14, 19, 2, 4, 15, 33, 9, 29, 5, 1, 60, 7, 18, 96, 76, 145, 43, 56, 13, 169, 88, 20, 86 and 148, all with Z`=1. Structure generation was performed using a quasi-random search, using the GLEE software [3]. All conformers up to 32.5 kJ/mol above the lowest energy gas phase conformer were included in searching. In addition higher energy conformers were also included, where the molecular energy + a molecular surface area bias term were within 22.5 kJ/mol of the global energy minimum conformer. The bias term was calculated as lowering the energy by 0.49 kJ/mol per Å^2 of solvent accessible surface area. This was based on ref [6]. A total of 528 conformers were included in CSP.

Crystal structure optimisation: Three stages of optimisation were performed for each generated structure: rigid-molecule optimisation using the FIT force field and atomic partial charges (fitted to the electrostatic potential generated from the full set of atomic multipoles); rigid-molecule optimisation using the FIT force field and atomic multipoles generated from a distributed multipole analysis of the DFT charge density; fully-flexible tight binding DFT, using the DFTB 3ob parameter set ad D3 dispersion correction.

Duplicate crystal structures were removed, first by clustering using comparison of simulated powder X-ray diffraction patterns, followed by clustering using the CSD API (with a 30-molecule cluster). Clustering was performed prior to and after DFTB re-optimisation of structures.

Structures for submission 1 were submitted based on their energy ranking after DFTB.

Submission 2

The set of structure provided for re-ranking were optimised using solid state DFT in the VASP [11] code. Calculations were performed with the PBE functional and a planewave basis set with pseudopotentials and GD3BJ dispersion correction. Optimisations were performed in three stages: fixed unit cell optimisation, followed by relaxed cell optimisation, both with a 500 eV planewave basis set cutoff. A final stage of optimisation was then performed with a 600 eV basis set cutoff and tighter convergence thresholds (PREC = Accurate, EDIFFG = -0.02). All calculations used a maximum electronic k-point spacing of 0.05 Å^{-1}.

XXXIII

Submission 1

Conformational sampling. Conformers were generated using low-mode sampling [1] with the Macromodel code and the OPLS-3 force field. These were re-optimised using Gaussian09 [2] with the PBE0 functional, GD3BJ dispersion correction and 6-311G++(d,p) basis set. Duplicate conformers were removed by comparison of all atom positions, with an RMSD limit of 0.1 Å. This resulted in 10 distinct conformers of the anion in the gas phase and 3 distinct conformers of the cation.

Crystal structure generation: Crystal structures were generated and lattice energy minimised in the following space groups: 2, 14, 4, 19, 1, 29, 33, 15 and 61, all with Z`=1. Structure generation was performed using a basin hopping search, using the GLEE software [10]. Searches were performed using all combinations of cation and anion conformers. Additional searchers were performed with the starting anion conformation distorted from the gas phase optimised conformers in 72 degree increments around the three flexible bonds, and performing CSP with all distorted anion conformations within 25 kJ/mol of the lowest energy conformer.

Crystal structure optimisation: Three stages of optimisation were performed for each generated structure: rigid-molecule optimisation using the FIT force field and atomic partial charges (fitted to the electrostatic potential generated from the full set of atomic multipoles); rigid-molecule optimisation using the FIT force field and atomic multipoles generated from a distributed multipole analysis of the DFT charge density; fully-flexible tight binding DFT, using the DFTB 3ob parameter set ad D3 dispersion correction.

Duplicate crystal structures were removed, first by clustering using comparison of simulated powder X-ray diffraction patterns, followed by clustering using the CSD API (with a 30-molecule cluster). Clustering was performed prior to and after DFTB re-optimisation of structures.

DFTB was found to transfer the proton in some crystal structures, resulting in a co-crystal, rather than a salt. Therefore, structures for submission 1 were submitted based on a combination of DFTB structures and structures from the force field stage (FIT + multipoles). Structures from both stages were ranked by relative energy, relative to the global minimum at that stage of calculations. We then took the 1500 structures with lowest relative energies from both sets combined, removing any structures that were duplicated between the two sets. Due to the combination of methods, the relative energies within this set of structures are not reliable.

Submission 2

The set of structure provided for re-ranking were optimised using solid state DFT in the VASP [11] code. Calculations were performed with the PBE functional and a planewave basis set with pseudopotentials and GD3BJ dispersion correction. Optimisations were performed in three stages: fixed unit cell optimisation, followed by relaxed cell optimisation, both with a 500 eV planewave basis set cutoff. A final stage of optimisation was then performed with a 600 eV basis set cutoff and tighter convergence thresholds (PREC = Accurate, EDIFFG = -0.02). All calculations used a maximum electronic k-point spacing of 0.05 $Å^{-1}$.

[1] I. Kolossváry and W. C. Guida , *Biopolymers*, 1996, 7863 , 5011 —5019

[2] Gaussian 09, Revision D.01, M. J. Frisch, G. W. Trucks, H. B. Schlegel, G. E. Scuseria, M. A. Robb, J. R. Cheeseman, G. Scalmani, V. Barone, B. Mennucci, G. A. Petersson, H. Nakatsuji, M. Caricato, X. Li, H. P. Hratchian, A. F. Izmaylov, J. Bloino, G. Zheng, J. L. Sonnenberg, M. Hada, M. Ehara, K. Toyota, R. Fukuda, J. Hasegawa, M. Ishida, T. Nakajima, Y. Honda, O. Kitao, H. Nakai, T. Vreven, J. A. Montgomery, Jr., J. E. Peralta, F. Ogliaro, M. Bearpark, J. J. Heyd, E. Brothers, K. N. Kudin, V. N. Staroverov, T. Keith, R. Kobayashi, J. Normand, K. Raghavachari, A. Rendell, J. C. Burant, S. S. Iyengar, J. Tomasi, M. Cossi, N. Rega, J. M. Millam, M. Klene, J. E. Knox, J. B. Cross, V. Bakken, C. Adamo, J. Jaramillo, R. Gomperts, R. E. Stratmann, O. Yazyev, A. J. Austin, R. Cammi, C. Pomelli, J. W. Ochterski, R. L. Martin, K. Morokuma, V. G. Zakrzewski, G. A. Voth, P. Salvador, J. J. Dannenberg, S. Dapprich, A. D. Daniels, O. Farkas, J. B. Foresman, J. V. Ortiz, J. Cioslowski, and D. J. Fox, Gaussian, Inc., Wallingford CT, 2013.

[3] David H. Case, Josh E. Campbell, Peter J. Bygrave, and Graeme M. Day, Journal of Chemical Theory and Computation 2016 12 (2), 910-924

[4] Sarah L. Price, Maurice Leslie, Gareth W. A. Welch, Matthew Habgood,Louise S. Price,Panagiotis G. Karamertzanis and Graeme M. Day, Phys. Chem. Chem. Phys., 2010,12, 8478-8490

[5] D. S. Coombes , S. L. Price , D. J. Willock and M. Leslie , *J. Phys. Chem.*, 1996, **100** , 7352 —7360

[6] H. P. G. Thompson and G. M. Day, *Chem. Sci.*, 2014,5, 3173-3182

[7] B. Hourahine, B. Aradi, V. Blum, F. Bonafé, A. Buccheri, C. Camacho, C. Cevallos, M. Y. Deshaye, T. Dumitrică, A. Dominguez, S. Ehlert, M. Elstner, T. van der Heide, J. Hermann, S. Irle, J. J. Kranz, C. Köhler, T. Kowalczyk, T. Kubař, I. S. Lee, V. Lutsker, R. J. Maurer, S. K. Min, I. Mitchell, C. Negre, T. A. Niehaus, A. M. N. Niklasson, A. J. Page, A. Pecchia, G. Penazzi, M. P. Persson, J. Řezáč, C. G. Sánchez, M. Sternberg, M. Stöhr, F. Stuckenberg, A. Tkatchenko, V. W.-z. Yu, and T. Frauenheim, "DFTB+, a software package for efficient approximate density functional theory based atomistic simulations", J. Chem. Phys. 152, 124101 (2020) https://doi.org/10.1063/1.5143190

[8] D. E. Williams *J. Comput. Chem.*, 2001, **22** , 1154 —1166

[9] P. Pracht, F. Bohle and S. Grimme, *Phys. Chem. Chem. Phys.*, 2020,22, 7169-7192

[10] S. Yang and G. M. Day, *J. Chem. Theory Comput*. 2021, 17, 3, 1988–1999

[11] G. Kresse and J. Hafner, Phys. Rev. B, 1993, 47, 558; G. Kresse and J. Hafner, Phys. Rev. B, 1994, 49, 14251; G. Kresse and J. Furthmuller, Comput. Mat. Sci., 1996, 6, 15; G. Kresse and J. Furthmuller, Phys. Rev. B, 1996, 54, 11169; G. Kresse and D. Joubert, Phys. Rev., 1999, 59, 1758

4. Group 6

Blind test 2021-2022, van Eijck (UPACK)

1 Introduction

This contribution describes the results of using the UPACK program package [1]. It was designed to generate a list of possible crystal structures by a random search method, using an empirical force field. Soon it became obvious that the ranking in such a list is generally inadequate. Therefore, the crystal structure generation should be followed (if possible) by more sophisticated calculations. An anisotropic intermolecular potential was implemented by Mooij [2] into the TINKER package, using ideas developed by Price and Stone. For flexible molecules, ab-initio intramolecular energies [3] were sometimes used. Later, blind tests have suggested that an entirely different approach, based on DFT-D calculations [4], performs much better.

It thus appears that for general use the UPACK program, as available to the scientific community [5], is now obsolete with regard to sufficiently accurate energy calculations. The follow-up using the programs XTINKER or GAMESS is not well documented and unavailable to other researchers for copyright reasons. Still, a sufficiently reliable list of potential structures can always be used as starting point for more sophisticated calculations, as preferred by the user.

In the series of blind tests the target structures tended to become larger and more flexible. The generation of possible structures and their subsequent ranking involve a correspondingly larger amount of computer time. The organizers of the present test have separated more clearly both stages of the prediction. The first part involves the construction of many hypothetical structures, without emphasis on ranking ("landscape generation"). After that, the second challenge is to rank a set of structures according to the probability of observing them.

In earlier work [1] we have noted that searches started to fail when D, the number of variables, is around 20. Of course, computer power has increased enormously since the year 2000, but the present molecules are not only larger but also more complex. Table 1 illustrates the situation.

Table 1. Degrees of freedom (D) of the search problems.

	XXVII	XXVIII	XXIX	XXX(*)	XXXI	XXXII	XXXIII
Cell	4	4	4	4	4	4	4
Position and orientation	6	6	6	12/18/18	6	6	12
Relevant dihedral angles	8	8	2	5/5/10	3	10	3
Total dimensionality D	18	18	12	21/27/32	13	20	19

The crystallographic parameters are given for $P2_1/c$, but the situation may be different for other space groups. For instance, $P1$ has 6 cell parameters but 3 position parameters can be chosen freely.

(*) For the three stoichiometric possibilities, see below.

2 Force fields

For each compound an individual force field was constructed. The intramolecular parameters for bond distances and bond angles were taken from comparable structures in de CSD database [6]. The potentials for the dihedral angles were modeled by Fourier terms. Torsional angles involving sp^2 atoms were restrained to planarity with aid of a large twofold term. Methyl groups had a threefold barrier of 0.8 kJ/mol for each combination of end atoms. The other dihedral angles can be essential to determine the conformation, and their choice is often critical for successful modeling. Their numbers were shown in Table 1; their barrier was taken as zero unless discussed otherwise in Section 4 for each target compound separately.

Point charges on the atoms were taken from ESP fittings, using MOLDEN [7] on 6-31G** wave functions calculated by the program GAMESS-UK [8].

In the first stage of the structure generation the interatomic van der Waals interaction was described by a Lennard-Jones ("LJ") potential. This form was chosen to ensure that short distances always remain repulsive, which is not the case for an exponential function. Essentially the parameters were taken from Jorgensen's OPLS force field [9-11], with some additions for halogens [12].

However, after this initial stage the Price-Williams ("PW") force field, with Buckingham repulsion [13-15], was preferred. Further energy minimization in this force field produced a preliminary set of structures. If computationally feasible, a final minimization was done where the intramolecular energy was calculated ab-initio (the "PW-AI" method). Here GAMESS-UK [8] was used again, with charges recalculated for each individual structure. A detailed discussion of this procedure and its merits has been given in the Supplementary Material of the sixth blind test [16]. The details of the PW potential are given in Table 2. Values for S, Si and Cu were deduced from the Dreiding force field [17].

Table 2. Parameters of the Price-Williams potential: $U_{Buck} = A\exp(-Br) - C/r^6$

	A (kcal mol^{-1})	B (Å^{-1})	C (kcal mol^{-1} Å^6)	Reference
H (on N)	1202	4.66	5.14	14
H (on O)	541	4.66	5.14	15
H (on C)	2861	3.74	32.6	13
C	88371	3.60	583.1	13
N	60834	3.78	329.4	13
O	54987	3.96	268.5	13
F	86932	4.16	201.7	13
S	55988	2.98	2947.3	17
Cl	227200	3.25	3128	12
Br	287800	3.25	3962	12
I	287800	3.14	4878	12
Si	50454	2.81	3758	17
Cu	8137	3.46	175.2	17

3 Procedure

The random search technique implemented in the UPACK program package [1,5] was used. Parameters were the crystal cell data and the molecular positions and orientations. All work was done with fully flexible molecules. A set of dihedral angles essential for the conformation (Table 1) was defined, and these angles were also treated as random parameters in the search. So there was no set of distinct starting conformations.

For each compound many structures with one independent molecule (or set of molecules) in the asymmetric unit were created in the thirteen space groups $P2_1/c$, $P\overline{1}$, $P2_12_12_1$, $P2_1$, $Pbca$, $C2/c$, $Pna2_1$, Cc, $Pca2_1$, $C2$, $P1$, $Pbcn$, and Pc. As explained above, the best structures were further optimized with the PW potential. After each calculation equivalent structures were removed by clustering. Here one should inspect the clustering list to be reasonably sure that the result is complete: all low-energy structures should have been found several times. Except for XXIX this was not the case, and many more cycles of structure generation were needed. This work was rather tedious, especially for the space groups with eight equivalent positions in the unit cell. Finally, structures with low PW energy were further optimized by the PW-AI method.

In the previous blind test is was possible to gain time by abandoning the preferred approach, where all torsional degrees of freedom are set to random values in each starting structure. Starting dihedrals could be set to the most probable values (and then allowed to optimize, as always). In the present study it was difficult to select such most probable conformations. Neither was there any indication for preferred space groups. It was tried to optimize the standard settings for structure generation, and some gain in efficiency could be obtained for the large molecules studied here. Details of computer use are given in Table 3.

For compound XXX the symbols C and T indicate the components cannabinol and tetramethylpyrazine, respectively. Here the stoichiometry challenge is a difficult problem. It can be reduced to study of an equilibrium like

$$\mathrm{CT} + \mathrm{T} \leftrightarrow \mathrm{CT_2}$$

CT and CT_2 are crystals whose energy is to be estimated in this work, but what about T? It is probably in solution, and its (free) energy depends on the interaction with the solvent. Indeed, it would be hardly surprising if the choice of solvent turned out to be important – not only for the kinetics but also for the thermodynamics. For instance, one would expect that strong attraction between the solvent and T would not favour the CT_2 form.

Lacking information about the solvent, it seems best to assume that T is also in the crystalline state. Thus the two components of XXX must also be studied to find their energy. A limited structure generation will suffice, as long as the differences between possible structures within one compound are smaller than the energetics of stoichiometry.

Table 3. Computational details.

	N(LJ)	CPU	N(PW)	CPU	N (PW-AI)	CPU	Total CPU
XXVII	458000	5900	1780	1500	148	720	8120
XXVIII	641000	700	3519	650	–	–	1350
XXIX	260000	160	5371	50	2027	220	430
XXIX ($Z'' = 2$)	630000	360	14140	80	1191	440	880
XXX-CT	1600000	4000	4305	600	1015	1030	5630
XXX-CT_2	382000	2100	584	150	304	350	2600
XXX-C_2T	138000	1100	385	20	220	450	1570
XXX-T	5000	20	999	10	619	50	80
XXX-C	10000	120	901	120	35	30	270
XXXI	1175000	650	2734	200	1566	620	1470
XXXII	307000	2350	1788	150	108	400	2900
XXXIII	1339000	3900	1628	400	1598	680	4980
Total CPU		21360		3930		4990	30280

N(LJ) is the number of structures generated (LJ force field), N(PW) is the number of structures retained after further energy minimization (PW force field), N(PW-AI) is the number of structures considered in the final ranking (PW-AI method). Various computers were used; CPU is the computing time (hours) standardized for one 2.66 GHz Intel Quad 9400 processor at Utrecht University.

4 Submission 1: Landscape Generation

The relations between the compound properties and the overall results are given in Table 4 for the submitted structures.

Table 4. Overview of key results for submitted structures.

	D	N(dih)	N(at)	N(clus)	RMS	Submitted		Range
XXVII	18	8	100	2	3.1	PW	1500	20
XXVIII	18	8	53	9	–	PW	1500	26
XXIX	12	2	20	110	3.2	PW-AI	1500	19
XXX-CT	21	5	71	1	3.2	PW-AI	976	24
XXX-CT_2	27	5	93	1	4.0	PW-AI	304	35
XXX-C_2T	32	10	120	1	5.8	PW-AI	220	68
XXX-T	10	0	22	458	1.8	–		
XXX-C	15	5	49	2	3.8	–		
XXXI	13	3	32	53	6.6	PW-AI	1500	46
XXXII	20	10	76	1	30.7	PW	1500	39
XXXIII	19	3	43	10	16.4	PW-AI	1500	91

The dimensionality D and the number of dihedrals N(dih) are taken from Table 1; N(at) is the number of atoms. N(clus) is the average number of times the best 10 structures were found. RMS is the root mean square deviation (kJ/mol) between the PW and PW-AI energies. Finally the numbers of submitted structures and their range in kJ/mol are given.

When N(clus) is small (compounds XXVII, XXX and XXXII) there is little hope that the experimental structure can be found among the submissions. For XXIX and XXXI the results should be essentially complete, with XXVIII and XXXIII as doubtful intermediate cases. A low value of RMS gives hope for a reliable ranking.

Compound XXVII. The Si–C≡C–C linkages were assumed to be linear with free rotation. Even after considerable investment in computer time the number of times the best structures were found (N(clus) in Table 4) was way too low to have a complete structure generation. Evidently the large number of atoms (100) is at least as important as the dimensionality (18), which in itself should be acceptable.

To avoid excessive computer effort the two iodine atoms were replaced by chlorine atoms in the AI calculations. Previous experience (compound XIII) has shown that the charges for the halogens are usually not so different. The structures treated in this way gave a good correlation with the PW energies (RMS in Table 4). So although finding the experimental structure would be a piece of luck, the PW ranking may be reliable.

Compound XXVIII. This is a curious structure, with NH groups linked to a $CuCl_2$ group. Considering more or less comparable structures in the CSD, the C–C=N···Cu group was kept planar. Free rotation was allowed about Cu···N and also for the four phenyl groups. The angles N···Cu···N and Cl–Cu–Cl were set at 155°with the bending force constant reduced by a factor of 10. The stretching force constants for atoms linked to Cu were likewise reduced.

The odd number of electrons around copper prevented ab-initio calculations. Standard force field values were taken for the atomic charges. Considering the complexity of the molecule, the structure generation seems reasonably complete. In the last cycle only a few structures of low ranking had to be added to the list.

No effort was made to consult the experimental data that were published during the blind test.

Compound XXIX. There are two essential dihedrals, *viz.* ω_1 = C(NH_2)–C–C(O)–O and ω_2 = C–C(O)–O–C(H_3). To reproduce similar fragments from the CSD, the corresponding torsional constants were set to enforce a distribution with peaks around 0°and 180°. For the NH_2 group only a weak preference for planarity was assumed.

After the structure generation the discrepancy between the PW and PW-AI energies was large (RMS value 31 kJ/mol). This was traced to the set with $\omega_2 \approx 0°$, which had high energies in the PW-AI force field. Trusting that better than PW, these structures were eliminated from the list.

For this compound simulated experimental data were provided. An inspection of the most favourable PW-AI powder diagrams did not give a satisfactory correspondence with that pattern. Therefore the structure generation was augmented with $Z'' = 2$ structures, which was quite feasible for this small compound. That exercise should also provide additional $Z'' = 1$ structures from space groups not considered in the standard method [18]. By studying only $P2_1/c$, $P\overline{1}$, $P2_12_12_1$, $P2_1$ and $P1$ [18] over 14000 structures were found, with poor clustering properties. It turned out that 669 structures could be reduced to $Z'' = 1$, of which 47 had not been found before. Only 5 of these new ones occurred in space groups studied in the $Z'' = 1$ structure generation, and were thus really missed previously.

For the first submission only the best 150 $Z'' = 2$ structures were included, corresponding to the statistical occurrence (around 10%) of such structures. Here the information from the powder diagram was not used, although an automated comparison with all 19511 structures could have eliminated a large majority. Such a program was not available. For the second submission the given diagram was manually compared with corresponding ones for hypothetical structures within a range of 13 kJ/mol. The 10 best-looking structures were selected, but no really satisfactory correspondence was present.

Compound XXX. The hydroxyl group in cannabinol was restrained to planarity. In the simulations it was found pointing away from the nearest phenyl ring. In the alkane tail the standard threefold C–C potential appeared to be adequate as it gave

dihedral angle distributions similar to CSD findings.

The challenge is to investigate forms with less than 4 components. Thus there are three possibilities, denoted in Tables 3 and 4 by CT, CT_2 and C_2T. The structure generation for the CT compound was already very time-consuming (Table 3). Even more disappointing, all low-energy structures were found only once (Table 4), suggesting that finding the experimental structure would be a very lucky event. For that reason not so much time was invested in the even more complex study of the CT_2 and C_2T compounds.

The problem of determining the most likely stoichiometric form has been discussed above. We compare three processes with the same left-hand side:

$$2\,C + 2\,T \rightarrow 2\,CT \quad (1)$$
$$2\,C + 2\,T \rightarrow CT_2 + C \quad (2)$$
$$2\,C + 2\,T \rightarrow C_2T + T \quad (3)$$

and calculate the energy differences, assuming – probably incorrectly – that all substances are in the crystalline state. The structure generation for the pure components was a comparatively simple task (Table 4). The results are given in Table 5. It is seen that all transitions seem energetically unfavourable; furthermore, CT is the most probable, but only with a small margin with respect to CT_2. The third compound, C_2T, would not be very likely to occur.

Table 5 gives also the quantity ΔE_4. This is interesting because it can be found without worrying about the influence of the pure components. It is equal to $\Delta E_2 + \Delta E_3 - 1.5\Delta E_1$, showing that either CT_2 or C_2T (or both) must be very unfavourable with respect to CT. Its calculation by other participants would provide a useful comparison.

Table 5. Energy differences between the crystal forms of compound XXX.

	PW	PW-AI
$\Delta E_1 = 2\,E(CT)$ - $2\,E(C)$ - $2\,E(T)$	5.9	7.3
$\Delta E_2 = E(CT_2)$ - $E(C)$ - $2\,E(T)$	12.0	13.7
$\Delta E_3 = E(C_2T)$ - $2\,E(C)$ - $E(T)$	46.4	40.7
$\Delta E_4 = E(CT_2) + E(C_2T)$ - $3E(CT)$	49.5	43.5

For the blind test submission the relative PW-AI energies were augmented with the differences from Table 5, uncertain as they are. Ranking the best 100 structures the C_2T data were discarded and, rather arbitrarily, 90 CT structures and 10 CT_2 structures were submitted.

Now that the pure components were also studied, it was interesting to see whether an experimental structure was encountered. For the T component such a structure is known (MPYRAZ02). The molecule lies on an inversion center in *Pbca*. It was easily found in $P2_12_12_1$ and $Pca2_1$ with rank 30 at ΔE = 2.6 kJ/mol in the PW-AI force field.

Compound XXXI. No potential was set for the three conformation-determining dihedrals or for the five-membered ring. The latter was found to deviate from planarity. The resulting distribution of dihedral angles was similar for PW and PW-AI, yet the correspondence in energies was not satisfactory (rms deviation 10 kJ/mol). For this relatively simple compound the structure generation appears to be fairly complete.

Compound XXXII. A large molecule with at least 10 internal degrees of freedom. Some dihedrals appear to prefer planarity, but the CSD information is so scanty that it is hardly useful to enumerate the choice of all torsional potentials that were set. The structure generation did not produce significant clustering and the correspondence between PW and PW-AI is bad. Only the 1500 best PW structures are submitted, without serious hope of hitting the experimental one.

Compound XXXIII. The anion is identical to the one in COKROJ, so details of the geometry could be taken over. No potential was set for the three conformation-determining dihedrals. The structure generation was fairly tedious, but in the end the clustering was acceptable. As for XXXI, the dihedral distribution was comparable for PW and PW-AI, but the energy comparison is bad. The ionic structure of the components was a complication: no preferred isolated dimer geometry could be found, so in the calculations charges +1 and -1 were used which excluded the possibility of charge transfer.

5 Submission 2: Structural Ranking

The hypothetical structures received from the CCDC were recalculated with the PW force field, in order to find out quickly whether problems arose. This was indeed the case for about 50 structures, since UPACK can only treat triclinic, monoclinic and orthorhombic structures. The only quick solution was to expand these structures, eliminating symmetry operations not available in the program. In this way two structures in compound XXXIII could not be treated as the number of artificially independent molecules became too large to handle. A few other structures gave diverging energy optimization for unknown reasons. The final numbers of structures studied ($N1$) are given in Table 6.

The CCDC results are guaranteed to contain the experimental structure(s), so a comparison with the UPACK-generated structures is interesting. The numbers of CCDC structures in the low-energy range of 15 kJ/mol ($N2$) are given in Table 6. The next line shows the number of these ($N3$) that occur in the present structure generation submission (CCDC structures obviously taken from that submission excluded). For XXVIII the overlap is excellent, except that just the CCDC structure with lowest energy was not found. At the other extreme, there was no overlap for XXXII where the structure generation was already judged to be hopelessly incomplete.

For the final ranking in submission 2 the energies were recalculated with the PW-AI method (except XVIII). As explained above, for compound XXVII iodine was replaced

by chlorine in the AI calculations. See Table 6 for computing times.

Table 6. Results for the ranking challenge.

	XXVII	XXVIII	XXXI	XXXII	XXXIII
$N1$ (finally ranked)	99	495	100	499	497
$N2$ (low CCDC structures)	32	29	45	15	36
$N3$ (common structures]	11	25	12	0	23
CPU time (hours)	455	10	55	1200	195

$N1$ is the number of structures included in this challenge.
$N2$ is the number of structures in a PW energy range of 15 kJ/mol.
$N3$ is the number of these that were present in submission 1.
The CPU time is defined as in Table 3.

6 Post-analysis

Up to this section nothing in the text has been changed after the experimental structures were disclosed.

All experimental structures were energy-minimized in the same force field as used in the predictions. Thus the comparison of energies and structure details was practically exact, in contrast to the CCDC approach where calculated structures are compared directly with the observed ones. Tables 7 and 8 give the results.

Table 7. First submission: observed and predicted structures.

	Form	ΔE (PW)	ΔE (PW-AI)	Submitted	Remarks, see text
XXVII	A	4.6	–	0038	Core atoms only
XXVIII		5.1	–	0015, 0019	Structures greatly deformed
XXIX		6.8	-0.3	–	$Z'' = 3$
XXX-CT		17.3	12.3	–	
XXX-C_2T		-22.0	-23.1	–	Disorder
XXXI	A	15.0	13.8	–	Disorder
	B	13.6	15.2	0198 (0389)	Success
	C	22.4	21.2	–	Space group $R\overline{3}$
XXXII	A	64.5	–	–	Disorder
	B-LT	51.4	–	–	$Z'' = 2$
	B-RT	54.6	–	–	From powder pattern
XXXIII	A	31.4	34.0	–	Disappearing polymorph
	B	17.7	1.8	–	Found and lost

ΔE (kJ/mol) denotes the difference between experimental and lowest predicted energy.
"Submitted" refers to the number in the files data_vaneyck.... for structures that bear some resemblance to the experimental structure. So this number is also the rank in the list of energies.

Table 8. Second submission: ranking of provided structures.

	Form	CCDC number	ΔE	Rank	Remarks
XXVII		28	1.1	2	
XXVIII		144	2.7	4	PW force field
XXXI	A(Maj)	98	11.3	28	Disorder
	A(Min)	1	7.6	14	
	B	25	12.4	33	
	C	89	17.9	61	
XXXII	A	317	167.4	473	Disorder
	B-LT	232	40.4	86	
	B-RT	30	69.6	243	From powder pattern
XXXIII	A	233	35.6	205	Disappearing polymorph
	B	452	4.0	3	

ΔE (kJ/mol) denotes the energy difference between CCDC-generated "experimental representative" structures and those with lowest energy. Except for XVIII, all structures were energy-optimized in the PW-AI force field.

Inspection of Tables 7 and 8 suggests that the ranking for compounds XXVII, XXVIII, XXIX and XXIII is adequate, especially in the PW-AI calculations. A more detailed discussion for the individual compounds follows.

Compound XXVII. The structure (form A at 90 K) was not found precisely. However, the CCDC performed a separate analysis where the six isopropyl groups were disregarded. Then one match was found at $\Delta E = 7.0$ kJ/mol.

Compound XXVIII. Upon energy minimization the experimental structure is seriously deformed. The cell parameters and the torsional angles change so much that it is no longer recognized as the correct structure. Nevertheless, with the force field used it is the only result that can be expected from the structure generation. It was indeed found in the submitted structures. As there is only one independent molecule on an inversion center in the unit cell, it could be generated in P1. However, it was also found a few times in P-1 with doubled cell.

Compound XXIX. The experimental structure was not found because it has three independent molecules in the unit cell. Its PW-AI energy is a little lower than for all submitted hypothetical structures.

As I found no satisfactory correspondence with the given powder diagram, the search had been extended to $Z'' = 2$. Getting no improvement, the natural next step would have been to investigate more space groups rather than larger Z''. With hindsight $Z'' = 3$ was tried in $P2_1/c$, but the correct structure was not found even after 500 hours CPU time – not surprising for $D = 28$ degrees of freedom...

Compound XXX. Contrary to my expectations, the experimental structures are CT and C_2T, the latter being disordered. So the structure generation was incomplete, as expected. This is especially obvious for C_2T, where all submitted structures are also much too high in energy. So the energies used to estimate the relative stabilities (Table 5) cannot be trusted at all.

Compound XXXI. Form A is disordered and was not found. Two fluorine positions are possible, in Table 7 their average energies are used. For form B two possible candidates were submitted; the CCDC analysis indicated #0389 whereas I prefer #0198. Form C was not found because space group $R\overline{3}$ was not investigated.

Compound XXXII. No structure was found, as expected. Form A is disordered, again the average energy for two possible forms is used in Table 7. Obviously the force field is quite unsuitable, see the large ΔE values in Table 8.

Compound XXXIII. No correct structure was submitted, although there was satisfactory clustering (Table 4). Upon reconsideration it was seen that form B was indeed among the generated ones, but discarded too early by a silly mistake: a preliminary version of the force field was used. This form is calculated to have a much lower energy than the vanishing polymorph A.

7 Discussion

The results are disappointing. As remarked after Table 4, for compounds XXVII, XXX and XXXII the finding of experimental structure(s) was *a priori* considered improbable. Other participants did find them, suggesting that insufficient effort was spent to construct adequate force fields. Besides, computer facilities were limited and the UPACK program appears to have passed its "best before" date. For one thing, the $R\overline{3}$ form C of compound XXXI could not be found because the program can only handle triclinic, monoclinic and orthorhombic structures.

For compound XXVII the structure prediction was only successful if the configuration of the six isopropyl groups was disregarded. For compounds XXVIII and XXXIII the torsional terms in the force fields were too carelessly constructed. As a result the correct structures were found, but extremely distorted (XXVIII) or too soon rejected in an early stage (XXXIII).

In the end only form B of compound XXXI was predicted satisfactorily, a rather poor result compared to many other participants. The final conclusion is that UPACK must be considered as being out of date, not surprising for a program developed around 1995.

8 References

[1] B. P. van Eijck and J. Kroon, Acta Cryst. **B56** 535-542 (2000).
[2] W. T. M. Mooij, B. P. van Eijck and J. Kroon, J. Phys. Chem. **A103**, 9883-9890 (1999).
[3] B. P. van Eijck, W. T. M. Mooij and J. Kroon, J. Comput. Chem. **22** 805-815 (2001).
[4] M. A. Neumann, F. J. J. Leusen and J. Kendrick, Angew. Chemie Int. Ed. **47**, 2427-2430 (2008).
[5] B.P. van Eijck, http://www.crystal.chem.uu.nl/∼vaneyck/upack.html
[6] F. H. Allen, Acta Cryst. **B58**, 380-388 (2002).
[7] G. Schaftenaar and J. H. Noordik, J. Comput.-Aided Mol. Design, **14**, 123-134 (2000).
[8] M. F. Guest et al., Mol. Phys **103**, 719-747 (2005).
[9] W. L. Jorgensen and D. L. Severance, J. Am. Chem. Soc. **112**, 4768-4774 (1990).
[10] W. L. Jorgensen, D. S. Maxwell, and J. Tirado-Rives, J. Am. Chem. Soc. **118**, 11225-11236 (1996).
[11] E. K. Watkins and W. L. Jorgensen, J. Phys. Chem. **A105**, 4118-4125 (2001).
[12] B. P. van Eijck, Phys. Chem. Chem. Phys. **4**, 4789-4794 (2002).
[13] D. E. Williams and D. J. Houpt, Acta Cryst. **B42**, 286-295 (1986)
[14] D. S. Coombes, S. L. Price, D. J. Willock, and M. Leslie, J. Phys. Chem. **100**, 7352-7360 (1996).
[15] T. Beyer and S. L. Price, J. Phys. Chem. **B104**, 2647-2655 (2000).
[16] A. M. Reilly *et al.*, Acta Cryst. **B72**, 439-459 (2016)
[17] S. L. Mayo, B. D. Olafson, and W. A. Goddard III, J. Phys. Chem. **94**, 8897-8909 (1990).
[18] B. P. van Eijck, J. Comput. Chem. **23**, 456-462 (2002)

9 Acknowledgements and correspondence

Toine Schreurs and Martin Lutz provided computer facilities and assistance.
The author is retired from Utrecht University, The Netherlands.
Correspondence address: vaneyck@chem.uu.nl or vaneijck@xs4all.nl

5. Group 8

The 7th blind test. Crystal structure prediction with *FlexCryst*

(Group 8)

Detlef Walter Maria Hofmann,[a]* Liudmila Nikolaevna Kuleshova[b] and Luca Pilia[c]

[a]CRS4, Loc. Piscina Mana 1, 09010 Pula (CA), Italy, [b]FlexCryst, Schleifweg 23, 91080 Uttenreuth, Germany, and [c] Department of Mechanical, Chemical and Materials Engineering, University Cagliari, Via Marengo 2, 09123. Cagliari, Italy. E-mail: dwmhofmann@aol.com

Abstract

The results of crystal structure prediction (phase one) were submitted for four of seven test molecules XXVII, XXIII, XXXI and XXXIII. The other test molecules were not attempted for different technical reasons: molecule XXIX, because module "POWDER" of *FlexCryst* was under major revision, the molecule XXX, for the lack of accessible CPU time, the molecule XXXII, for too high conformational degrees of freedom.

Flowchart of prediction included: molecular conformations generation (*Mercury*), crystal structure generation (*FlexCryst*), energy calculation of the conformations (DFT *Gaussian*), Gibbs energy calculation of the lattice (*FlexCryst*), clustering according to the powder diagrams (*FlexCryst*) and final sorting of crystal structures.

Method

In the contrast to the most presented approaches, we calculate the inter-molecular interaction energies with a general force field, recently developed by applying data mining to the experimental structural information stored in CSD (Kuleshova, 2021; Hofmann & Kuleshova, 2022).

The main feature of the used here general force field (Hofmann & Kuleshova, 2023) was the parameters, which were derived for **all available** in CSD types of atoms at the same time and therefore self-consistent. Since only crystal structures, defined in room temperature experiments were used to derive the effective potentials, obviously that the obtained scoring function allows for calculation of the intermolecular Gibb's energy at room temperature.

For three of submitted structures we used our standard procedure (the conformations search followed with DFT probe energy of obtained molecular conformations), while for the structure XXVIII the experimental molecular conformation was taken. (Evidently, a knowledge-based crystal structure prediction discovers, if the crystal structure is contained in the training set and to subtract out the effect was not furthermore reasonable.)

Generation of the conformations. The conformations of tested molecules (for exclusion of XXVIII) have been generated with the CSD Conformer Generator. With this step the correct molecular geometries were found nearly for all tested molecules. Generation failed only for disordered and low temperature structures. For the case of disordered structures it remains to us unclear how to compare conformations

correctly. For the low temperature structures the knowledge-based conformer generation failed because into design only room temperature structures were taken into account.

Calculation of the conformational energy. The single-point calculations of the energy of conformers generated by CSD were performed at Density Functional Theory (DFT) (Parr et al., 1989)[1] level employing the *GAUSSIAN 16* (Gaussian 16, Frisch et al., 2016)[2] software package. The functional used throughout this study was B3LYP (Becke et al., 1993; Lee et al., 1988)[3]. The basis set employed for all atoms was the valence double-zeta 6-31+G(d)[4] (Francl et al., 1982; Rassolov et al., 2001; Frisch et al 1984) except for the case of XXVIII, where, for the iodine atoms, the pseudopotential LANL2DZ was used (Hay et al., 1985).[5] All structures were input using ArgusLab 4.0 (Thompson, ArgusLab 4.0.1).[6]

As energy we obtained the enthalpy; the energy of a given conformer ΔG_i^{intra} we expressed as difference in energy with the lowest conformation, which is assumed to be approximately the difference in the Gibb's energy.

$$\Delta G_i^{intra} = G_i^{intra} - G_{min}^{intra} \approx H_i^{intra} - H_{min}^{intra} \quad (1)$$

Generation of crystal structures . For every tested molecule the first ten molecular conformations with the best values of ΔG_i^{intra} were used consequently for crystal structures generation. During the generation, conformations were kept rigid. The crystal structures were generated randomly with *FlexCryst*.

Unfortunately, during all period of the blind test in our institution (CRS4), only a few CPUs were available (max 5000 CPUs hours per run) for calculations. For these reasons the generation of structures was restricted by the space groups 1, 2, 14 and 19, with only one molecule in the asymmetric cell. These restrictions hindered a possibility of successful prediction for structures XXXI C and XXXIII.

Calculation of the lattice energy. The generated structures were minimized with the data mining force field. The Gibb's energy is hereby the sum of pairwise interaction of the atoms in the crystal.

$$G_i^{inter} = \sum g(n_k m_l) \quad (2)$$

Before to perform crystal structure predictions for every tested molecule we checked the suitability of force field for similar structures containing the same atom types and calculated the energy of these structures. In this way we have discovered that the CSD contained already the test molecule XXVIII and informed the organizers of the blind test. During the minimization the conformation was kept rigid. For this reason, we used experimental geometry for crystal structure prediction and step of generation and probe of molecular conformation was omitted.

Clustering according to the powder diagram. The obtained crystal structures after minimization were clustered according to their powder diagrams. As similarity index, the area between the anti-derivatives of the two powder diagrams was used (Hofmann et al., 2009). The clustering was used to control the progress of the CSP and stop the CSP, if the desired coverage of the landscape was achieved, and to eliminate in the final list of submitted structures multi fold structures.

Sorting of the crystal structures. The total energy of the crystal structure has been calculated as the sum of the intra- and the inter-molecular energy.

$$G_i^{crystal} = \Delta G_i^{intra} + G_i^{inter} \quad (3)$$

The crystal structures have been sorted according to this energy.

References

- Becke, D. (1993). *J. Chem. Phys.* **98**, 5648-5652.
- Francl, M. M.; Pietro, W. J.; Hehre, W. J.; Binkley, J. S.; DeFrees, D. J.; Pople, J. A. & Gordon, M. S. (1892). *J. Chem. Phys.* **77**, 3654-65.
- Frisch, M. J.; Pople, J. A. & Binkley, J. S. (1984). *J. Chem. Phys.* **80**, 3265-69.
- Gaussian 16, Revision C.01, Frisch, M. J.; Trucks, G. W.; Schlegel, H. B.; Scuseria, G. E.; Robb, M. A.; Cheeseman, J. R.; Scalmani, G.; Barone, V.; Petersson, G. A.; Nakatsuji, H.; Li, X.; Caricato, M.; Marenich, A. V.; Bloino, J.; Janesko, B. G.; Gomperts, R.; Mennucci, B.; Hratchian, H. P.; Ortiz, J. V.; Izmaylov, A. F.; Sonnenberg, J. L.; Williams-Young, D.; Ding, F.; Lipparini, F.; Egidi, F.; Goings, J.; Peng, B.; Petrone, A.; Henderson, T.; Ranasinghe, D.; Zakrzewski, V. G.; Gao, J.; Rega, N.; Zheng, G.; Liang, W.; Hada, M.; Ehara, M.; Toyota, K.; Fukuda, R.; Hasegawa, J.; Ishida, M.; Nakajima, T.; Honda, Y.; Kitao, O.; Nakai, H.; Vreven, T.; Throssell, K.; Montgomery, J. A., Jr.; Peralta, J. E.; Ogliaro, F.; Bearpark, M. J.; Heyd, J. J.; Brothers, E. N.; Kudin, K. N.; Staroverov, V. N.; Keith, T. A.; Kobayashi, R.; Normand, J.; Raghavachari, K.; Rendell, A. P.; Burant, J. C.; Iyengar, S. S.; Tomasi, J.; Cossi, M.; Millam, J. M.; Klene, M.; Adamo, C.; Cammi, R.; Ochterski, J. W.; Martin, R. L.; Morokuma, K.; Farkas, O.; Foresman, J. B.; Fox, D. J. Gaussian, Inc., Wallingford CT (2016).
- Hay P. J. & Wadt, W. R. (1985). *J. Chem. Phys*. **82**, 270-83.
- Hofmann, D. W. M. & Kuleshova, L. N. (2022). International Tables for Crystallography Volume C Mathematical, physical and chemical tables, chap. Data mining. I. Machine learning in crystallography. International Union of Crystallography.
- Hofmann, D. W. M. & Kuleshova, L. N. (2023). Acta Crystallographica A79 132-144 .
- Hofmann, D. W. M., Kuleshova, L. N., Hofmann, F. & D'Aguanno, B. (2009). J. Chem. Phys. Letters, 475, 149.
- Kuleshova, L. N. (2021). Acta Crystallographica A, 77, C711{C711.
- Lee, C.; Yang, W.; Parr, R. G. (1988). *Phys. Rev. B*, **37**, 785-789.
- Parr, R. G.; Yang, W. Density Functional Theory of Atoms and Molecules; Oxford University Press: Oxford, 1989.
- Rassolov, V. A.; Ratner, M. A.; Pople, J. A.; Redfern, P. C. & Curtiss, L. A. (2001). *J. Comp. Chem.* **22**, 976-84.
- Thompson, M. A. ArgusLab 4.0.1; Planaria Software LLC: Seattle, WA, http://www.arguslab.com/arguslab.com/ArgusLab.html/.

6. Group 10

Supplementary information for the 7th Blind Test phase 1 submission of Group 10

Yingdi Jin*, Zhuocen Yang, Lu Tan, Chao Chang, Guangxu Sun, Xuekun Shi, Congcong Liu, Xin Yue, Wenbo Fu, Xiaolu Lin, Yunfei Zhou, Zenghui Liu, Qun Zeng, He Li, Baimei Shi, Tian Zhou, Chandler Greenwell, Michael A. Bellucci, Sivakumar Sekharan.

XtalPi, Shenzhen Jingtai Technology Co., Ltd., International Biomedical Innovation Park II 3F, 2 Hongliu Rd, Futian District, Shenzhen, China

Methodology

The polymorph landscape for each compound is predicted with XtalCSP. XtalCSP is an intelligent crystal structure prediction platform for real industrial production practice based on cloud-computing [1]. This platform consists of two main parts as shown in figure 1, one is the automated crystal structure prediction process (the lower part of figure 1), and the other is a decision tree (the upper part of figure 1) for controlling the CSP process. The automated CSP process integrates many components, including conformation analyses, force-field parameterization, crystal structure generation, clustering, high-precision ranking, stability evaluation at limited temperatures and crystallizability analysis, etc. Each component contains a series of algorithms to adapt to various systems. And the decision tree (the upper part of the process) assigns appropriate algorithms for each component to adapt to the current system. The decision-making basis of the decision tree in each component of the CSP process is different. This document will cover the part in the orange box in figure 1 and focus on the structure generation stage.

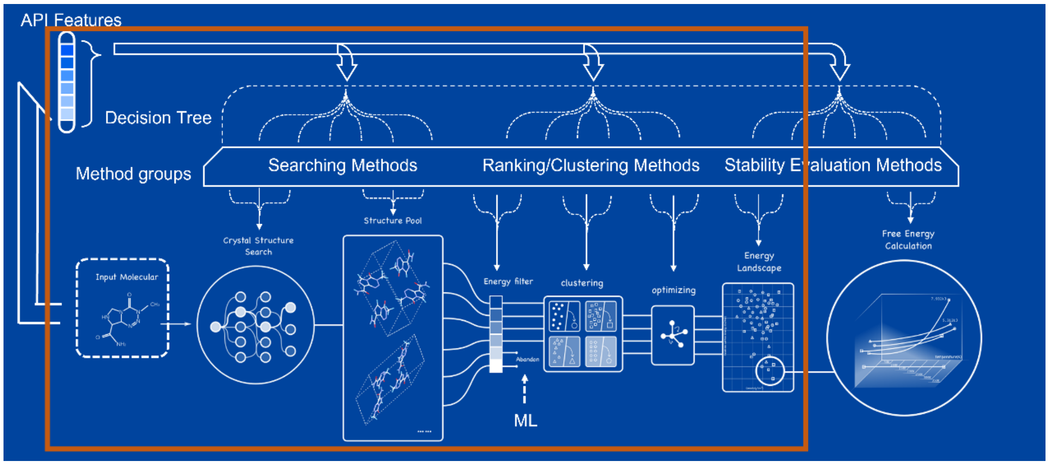

Figure 1

In the structure generation component, new structures mainly come from two sources, one is to generate totally new structures through generation algorithms, and the other is through continuous evolution algorithms. We mainly use completely random and Generative Adversarial Network (GAN) to generate totally new structures, while using global optimization algorithms to obtain new structures from continuous changes of existing structures [2,3,4].

When generating a totally new structure, the complete random algorithm can ensure the diversity of the structures. But it can't ensure that the generated structure is reasonable. It needs to combine an evaluator after each generation of the structure to determine whether the structure is chemically reasonable. So, we developed a series of scoring models based on machine learning algorithms such as Random Forest, Cubist, Gboost, etc. to judge the chemical rationality of a newly generated structure. However, this method is very inefficient for extremely complex systems. Sometimes it is difficult to generate a single chemical reasonable structure even if more than tens of thousands of generations and judgments are implemented. Therefore, we add GAN to deal with extremely complex systems. GAN includes two models. One is a generative model and the other is a discriminative model. The task of generative models is to generate low-energy crystal structures. The task of the discriminant model is to judge whether the crystal structure is real or fake. The real structure comes from the searched low-energy structure, and the fake structure comes from the generated model. GANs are characterized by molecular flexibility angles and crystal parameters in crystals.

For the continuous evolution of structures, we are not restricted to one specific algorithm because all kinds of global optimization algorithms have their advantages and disadvantages. At present, the most commonly used global optimization methods in this field are mainly divided into three categories, stochastic methods represented by direct Monte Carlo and Parallel Tempering Monte Carlo (PTMC) method [5], heuristic algorithms represented by Particle Swarm Optimization (PSO) [6, 7] and based on response surface approaches represented by Bayesian optimization (BO) [8]. The stochastic method has the best global property, and theoretically, as long as it runs long enough, it can always reach the global minimum of the potential energy surface. However, the efficiency of this kind of method is very low, which makes it necessary to consume a large amount of computation to ensure convergence even if the system is not too complex. To this end, we combined the Monte Carlo method with a series of traditional deterministic optimization methods represented by simplex optimization and developed our Self Adaptive Monte Carlo method. Even so, it is still impractical to only use the stochastic methods to achieve the global minimum when the degree of freedom is greater than a certain value. Because the calculation time required to reach the global minimum increases exponentially with the increase of the degree of freedom. Other kinds of approaches including the heuristic method and response surface approaches usually search the space in a more intelligent way. This makes them generally have higher efficiency and faster convergence. For this reason, we also added heuristic algorithms such as Particle Swarm Optimization (PSO), Cuttlefish Algorithm (CFA) etc. and response surface approach Bayesian optimization (BO) to our global optimization algorithm group. However, heuristic algorithms cannot always reach the global optimum, and they are prone to premature when encountering complicated systems with a too high degree of freedom. That is, the algorithm has shown convergence before it encounters a real low energy region. The Bayesian optimization, on the other hand, usually reaches a relatively optimal region rather than a global minimum. Another important problem is that, In addition to reaching the global minimum, CSP also needs to cover all local minima in the global low-energy region as much as possible. Therefore, we usually need to combine these global optimization algorithms with some deterministic local optimization to get the final landscape. Various global optimization methods and various structure generators mentioned in the previous paragraph can combine dozens of structure generation strategies.

The decision tree is responsible for selecting one or more executions from the dozens of strategies combined by these global optimization algorithms and new structure generators. In the structure generation component, the main decision-making basis includes the system components, Z' values, degrees of freedom, API molecular features, co-former features, potential hydrogen bond donors and receptors, characteristic functional groups, ring characteristics, heavy atom types, etc. With the accumulation of practical experience, the decision tree is becoming more and more accurate as well as algorithms are increasing.

Furthermore, in the structure generation stage, we adopted the method of multi round execution, and update the force field between rounds. Since we use our tailor-made force field, we can constantly update the force field parameters during a CSP process to make it gradually approach the quantum chemistry energy surface. The decision tree is responsible for determining how many rounds the structure generation process needs, how long each time it is executed and when the force field parameters need to be updated. Then the decision tree estimates the search completeness through the built-in statistic module and finishes the structure generation process when appropriate. After a hierarchical process of energy ranking and structure screening, the final landscape is generated. Then the lattice energies of the final landscape are evaluated using the optPBE-vdW [9] exchange-correlation functional corrected for dispersion effects as implemented in the Vienna ab Initio Simulation Package (VASP) [10,11,12].

Table 1. Searched space groups of each 7th CSP blind test targets

Target	Search Space group
XXVII	1 2 4 5 7 9 13 14 15 18 19 20 29 33 56 60 61 76 78 92 96 144 145 169 170
XXVIII	1 2 4 5 7 9 13 14 15 18 19 20 29 33 56 60 61 76 78 92 96 144 145 169 170
XXIX	1 2 4 5 7 9 13 14 15 18 19 20 29 33 56 60 61 76 78 92 96 144 145 169 170
XXX	1 2 4 5 7 9 14 15 19 29 33 60 61 62
XXXI	1 2 4 5 7 9 13 14 15 18 19 20 29 33 56 60 61 76 78 92 96 144 145 169 170
XXXII	1 2 4 5 7 9 13 14 15 18 19 20 29 33 56 60 61 76 78 92 96 144 145 169 170
XXXIII	1 2 4 5 7 9 14 15 19 29 33 60 61 62

Alignment of Experimental and Predicted Structures

Table 2 shows the RMSD20 calculation results and the structure overlays between first round submitted crystal structures and the published experimental solved crystal structures of each 7th blind test target. The angle tolerance is set as 40 degrees and the distance tolerance is 40 percent. If a disordered experimental form is classified to have a dynamic disorder, both the major and minor components would be used to perform the RMSD20 calculation and the lowest RMSD value would be presented. Meanwhile, experimental forms with the static disorder would be considered

as separate experimental forms and each of the major and minor components would be used to perform the RMSD20 calculation.

Table 2. Alignment of experimental and predicted structures

Target	Matched submitted crystal structures	RMSD20 result	Structural overlay
XXVII Form A	data_11_Z1_st_YVAfc 7hblQA9yLo8.cif	0.514 Å	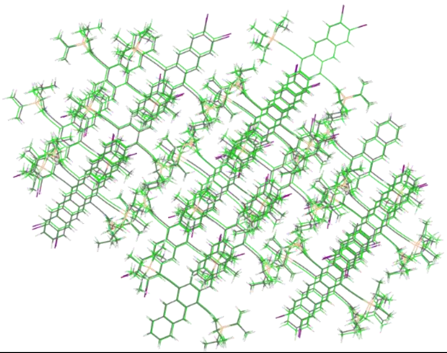
XXVIII Form A	data_2_T1_st_YK2w9 oAjAAAnSf6Z.cif	0.196 Å	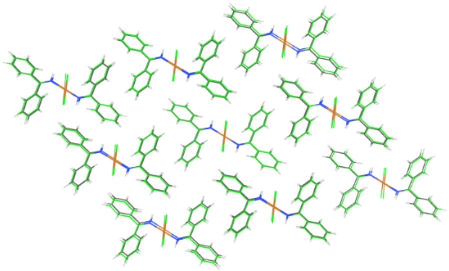
XXIX Form A	data_1_z3_stYSxRB1 Za6zN2UQXt.cif	0.183 Å	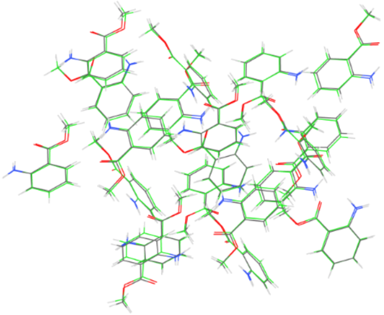
XXX Form A	data_1_A2B_st_YFhl-gwufAABru26.cif	0.232 Å	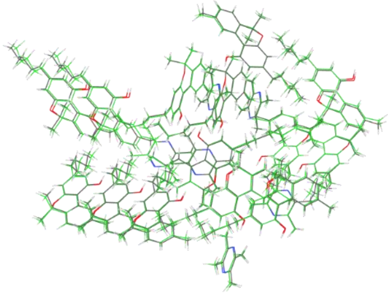

XXX<br>Form B	data_11_AB_st_X_fxy<br>C1FJgABaZL2.cif	0.191 Å	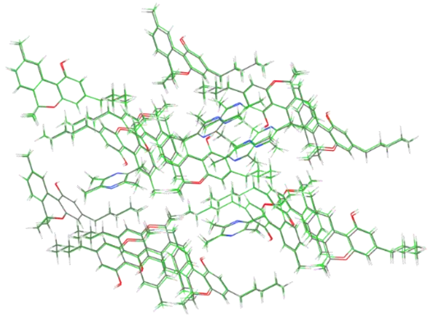
XXXI<br>Form A<br>Major	X3	0.114 Å	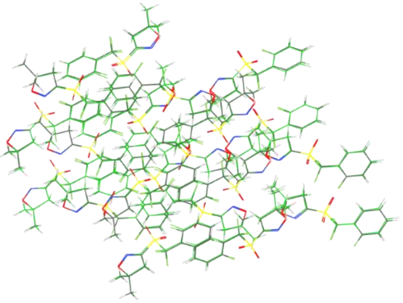
XXXI<br>Form A<br>Minor	X6	0.256 Å	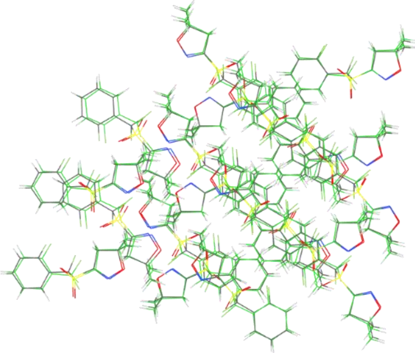
XXXI<br>Form B	X188	0.396 Å	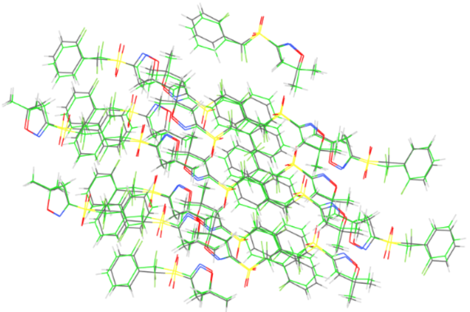

XXXII Form A	X815	0.219 Å	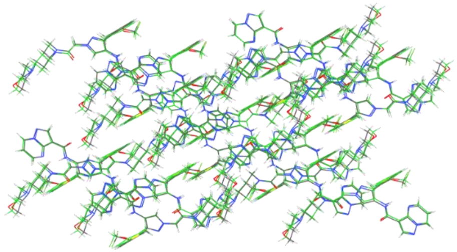
XXXII Form B	X10	0.34 Å	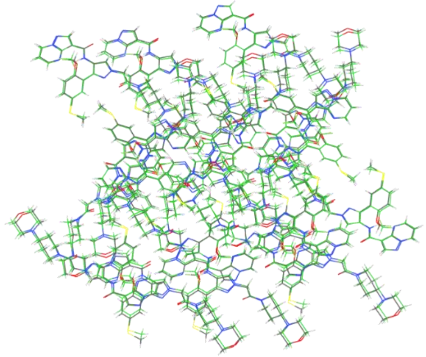
XXXIII Form A	X1	0.216 Å	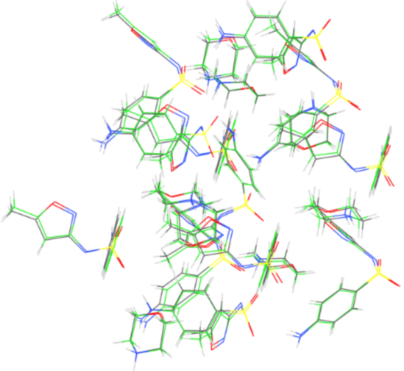
XXXIII Form B	X5	0.179 Å	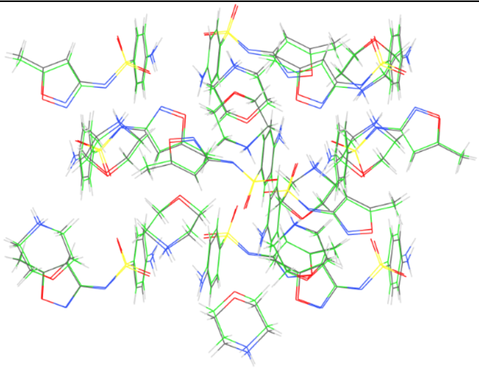

* Please notice that we did not perform CSP for Target XXXI with space group #148 (R-3). The experimental observed Form C, however, has the space group #148 (R-3). After checking, it is confirmed that the XtalCSP is able to find Form C using the standard self-adopted MC method with reasonable searching steps.

The Most Likely Observed Stoichiometries of Target XXX

For target XXX, two experimentally known forms with different stoichiometries where the number of components is < 4. This means that for the stoichiometry prediction challenge, the potential stoichiometry ratios of API: conformer to be considered are 1:1, 2:1 and 1:2. For convenience, we will express them as AB, A2B and AB2 respectively in the following. In order to compare the relative stability of crystal structures with different stoichiometric ratios, it is necessary to establish the conversion relationship between crystals with different stoichiometric ratios through thermodynamic cycles. We adopt the method described in this publication [13], using the gas state as the intermediate state to establish the free energy change between the crystal states with different chemical components. The crystal free energy is approximated by lattice energy with zero temperature approximation. Here, taking the A2B crystal state with the lowest energy as the reference state, and considering the relative free energy difference (approximate to be lattice energy change at zero temperature) between this form with other stoichiometries, we can get the virtual crystals that are most likely to be observed in the experiment(table3) with eq. (1) and eq. (2).

$$\Delta G_{shift}(AB) = E_{AB_{cryst}} + E_{A_{cryst}} \quad (1)$$

$$\Delta G_{shift}(AB_2) = E_{AB_{cryst}} + E_{A_{cryst}} - E_{B_{cryst}} \quad (2)$$

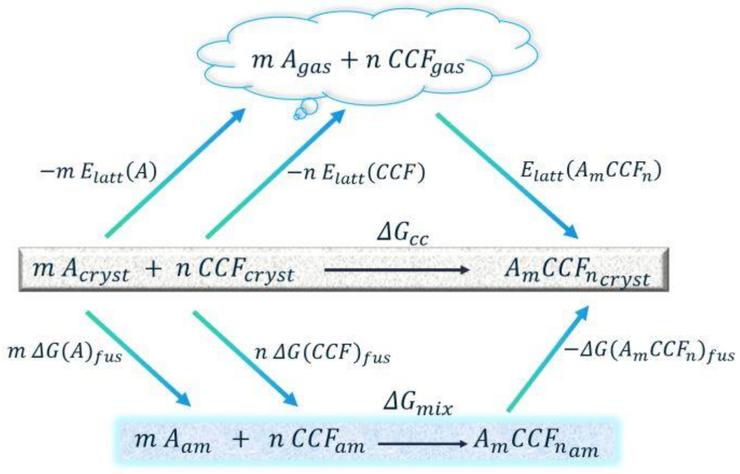

Figure 2. The thermodynamic cycle between different stoichiometries.

Table 3. relative free energy difference (approximate to be lattice energy change at zero temperature) between lowest A2B virtual form with other stoichiometries virtual forms.

Name	Type	Delta lattice energy (kJ/mol)
Rank1_st_YFhl-gwufAABru26	A2B	0.0000
Rank2_st_YJKrcxsYUhK3VkTx	A2B	2.2855
Rank3_st_X_athRjU-QABKcwU	AB	5.8048
Rank4_st_X_ae8BjU-QmPN	A2B	6.0262
Rank5_st_YFhl-gwufAABruPp	A2B	6.0544
Rank6_st_YJKrcxsYUhK3VkYY	A2B	7.9738

Rank7_st_X_ae8BjU-QmPJ	A2B	8.8262
Rank8_st_YFhl-gwufAABru4-	A2B	9.9090
Rank9_st_YFhl-gwufAABrvNa	A2B	10.3326
Rank10_st_YFhl-gwufAABruwj	A2B	10.5901
Rank11_st_X_fxyC1FJgABaZL2	AB	10.7642
Rank12_st_YFhl-gwufAABru3J	A2B	11.4367
Rank14_st_X_fxyC1FJgABaZLK	AB	11.5930
Rank15_st_X_at0xjU-QABKc1O	AB2	11.7378
Rank16_st_X_athRjU-QABKcyb	AB	11.8877
Rank17_st_X_athRjU-QABKcwT	AB	11.9078
Rank18_st_X_athRjU-QABKcwV	AB	12.0242
Rank19_st_X_athRjU-QABKcyd	AB	12.2063
Rank23_st_X_athRjU-QABKczz	AB	12.6044
Rank25_st_X_athRjU-QABKc0G	AB	13.0363
Rank29_st_X_fxyC1FJgABaZL7	AB	13.5387
Rank38_st_X_at0xjU-QABKc5H	AB2	14.4990
Rank143_st_X_at0xjU-QABKc1P	AB2	19.1695
Rank183_st_X_at0xjU-QABKc2G	AB2	19.9416
Rank240_st_X_at0xjU-QABKc1Y	AB2	20.7689
Rank379_st_X_at0xjU-QABKc5-	AB2	22.3316
Rank407_st_X_at0xjU-QABKc2m	AB2	22.5544
Rank408_st_X_at0xjU-QABKc5w	AB2	22.5560
Rank426_st_X_at0xjU-QABKc1q	AB2	22.6513
Rank463_st_X_at0xjU-QABKc5F	AB2	23.0327

Force Field Parameterization of Target XXVIII

The initial force field parameters of ligands came from the general Amber force field (GAFF2) [14]. In order to obtain the configuration of the specific structure (trans- or cis-) in the CSP process, we added topological connections between Cu2+ and ligands, such as bonds, angles and torsion angles. To obtain the corresponding force field parameters, we conducted ab initio calculations for the compounds. The equilibrium bond lengths and angles are obtained from the optimized QM structures, bond stretching and bond angle bending force constants were produced using a modified Seminario method [15]. To preserve the freedom of ligands in spatial position, the force constants of torsional angle x-Cu-N-x were set to 0.0, and the other force constants of torsional angles were refitted by QM torsion scan data. The electrostatic potential (ESP) was used to derive point charges with the RESP program. The method m062x/6-31+G* (PCM) optimization is followed by ESP calculation at the HF/6-31G* level. The charge of Cu ion is not limited to +2 but depends on the fitting result. We scanned the Cu-N and Cu-Cl bonds of the simplified model molecules to obtain continuous scanning points, and then calculated the QM single point energies of these structures. By adjusting the van der Waals parameters of Cu2+, the potential energy surface of QM/MM is consistent, and we finally obtain the van der Waals parameters of Cu2+, as well as the other atoms adopted parameters from GAFF2.

ILUM Tackle Highly Symmetric Molecules (Target XXVII)

'Ilum' is Xtalpi's in-house software for comparing crystal structures to identify similarities in molecular packing environments. We adopt the algorithm that James Alexander Chisholm etc. mentioned in reference [16] and carry out some engineering acceleration. This algorithm has two

characteristics. One is that with the increase of the number of atoms in the system, the calculation cost increases rapidly. The other is that the computational cost of systems with highly symmetric molecules could be really high and sometimes fail. According to a recent benchmark test on multiple high symmetry molecules, the implementation of Ilum is able to tackle some highly symmetric crystals. Target XXVII, as a molecule with high symmetry, it has hundreds of traversal paths [16] (the following is represented by N_{path}), the comparison speed of Ilum is about 30ms/pair. Generally, for cases with $N_{path} < 6$, Ilum generates a speed of 3-16 ms/pair; for the other cases with $N_{path} \geq 6$, which are deemed as "highly symmetric", Ilum keeps a speed of 3-80 ms/pair, still fast enough for CSP research. All these tests were performed on 110 cores of Intel Xeon Gold 6348 CPU @ 2.60GHz.

Hierarchical Clustering of Target XXVII

Due to the extremely high symmetry of Target XXVII molecule (>4000 identical pathways in molecular RMSD calculation), the regular RMSD-based crystal structure comparison is considerably time-consuming. This makes the clustering method based on the whole pairwise RMSD calculation unaffordable. Moreover, the rotation of the six terminal isopropyl groups does not significantly contribute to the RMSD result, which may lead to the misidentification of identical crystal structures. Therefore, to achieve both efficiency and accuracy, we adopted a 3-step hierarchical strategy to make the clustering (Figure 3).

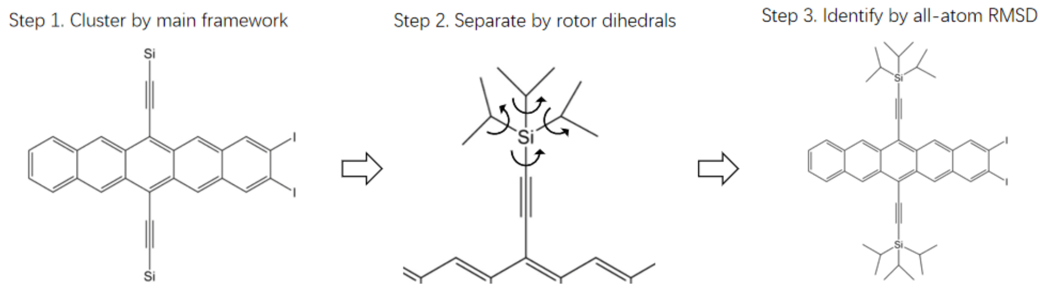

Figure 3. Hierarchical clustering strategy

The first step of the process is the regular RMSD-based clustering only takes the main framework of the molecule into account. This separates the crystal structures into different packing-pattern groups. By removing the six terminal isopropyl groups, the number of identical pathways used to calculate RMSD reduces to 2, which tremendously accelerates the structure comparison process.

In the second step, crystal structures within each packing-pattern group are further separated based on the values of molecular rotor dihedral angles. The crystal structures with similar dihedral-angle sequences (identical pathways considered) are grouped as a potential-match group.

The third step is the pairwise all-heave-atom RMSD calculation within each potential-match group. XtalPi’s in-house structure similarity comparison tool, ILUM, is available for efficiently tackling highly symmetric molecule systems. The crystal structures with small RMSD value are then identified as similar ones.

This hierarchical clustering strategy significantly reduces the total amount of the time-consuming all-heave-atom RMSD calculation through the first and second clustering steps, making the entire calculation much more efficient while maintaining or even increasing the accuracy of the clustering result.

PXRD and Structural Analysis of Target XXIX

The PXRD pattern of the experimental form of target XXIX was provided as a plot in the entry document of the 7th CSP Blind Test. However, as shown in figure 4, the provided PXRD plot had poor quality and might have had bad crystallinity, represented by non-negligible background noise, bumpy baseline, uncertain peak positions of diffraction peaks at low 2θ region, and wide FWHM of each diffraction peak.

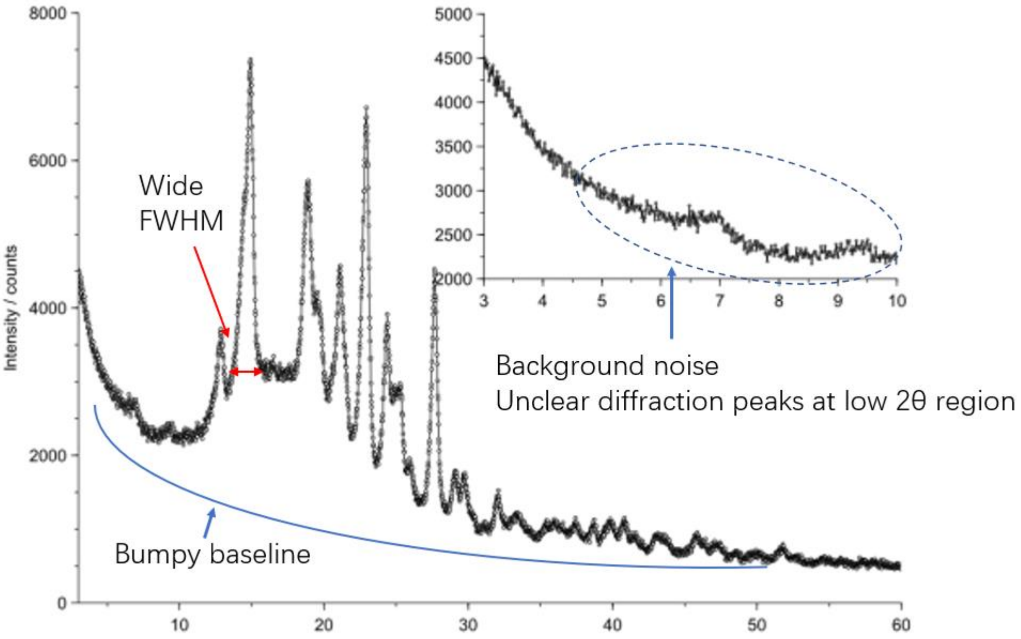

Figure 4. Raw PXRD plot of the experimental form of XXIX

To prepare the PXRD plot for indexing and further analysis, we first transformed the PXRD plot into a 2D array (x: 2θ, y: intensity) and smoothed/normalized the PXRD data curve. The processed PXRD curve is shown in Figure 5.

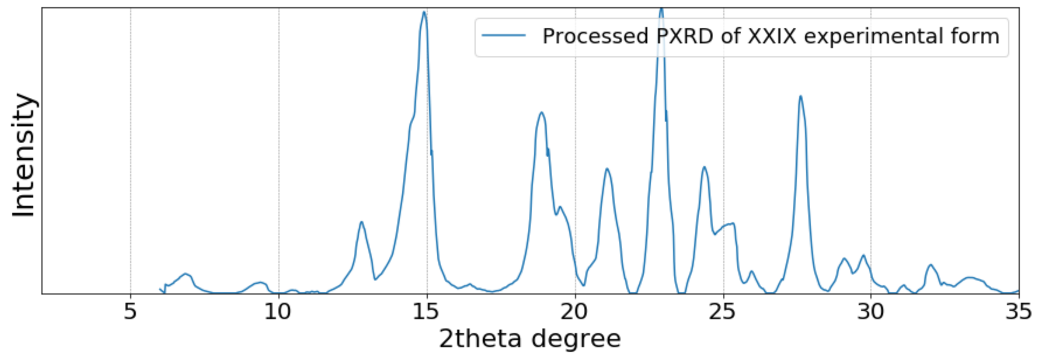

Figure 5. Processed PXRD curve of the experimental form of XXIX

We performed PXRD indexing using an in-house developed indexing algorithm. Due to the poor quality of the provided PXRD pattern, particularly the background noise and unclear diffraction peaks at the low 2θ region, obtaining a highly reliable PXRD indexing result was challenging. However, some of the PXRD indexing results hinted that the experimental form could have Z'>2, specifically, Z'=3, Z'=4, or Z'=6 forms. Our previous observations indicate that small APIs (namely molecule weight within 200 g/mol) could crystallize as a high Z' prime form [17]. The PXRD indexing result supported us to move forward with the Z'=3, 4, and 6 CSP study. Z'=5 was not selected due to the limited observation for small organic molecule crystals. Some representative high Z' prime indexing results are shown in Figure 6.

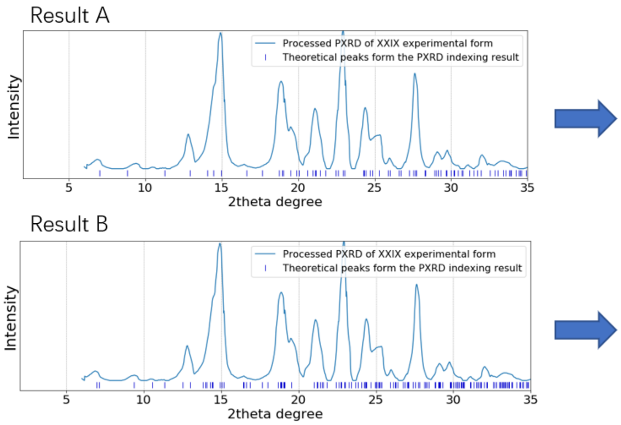

	Result A	Result B
a	12.614 Å	7.881 Å
b	10.042 Å	12.488 Å
c	9.367 Å	13.853 Å
α	90°	96.237°
β	93.12°	96.833°
γ	90°	93.119°
**Z prime**	**3**	**6**
Space group	P c (#7)	P 1 (#1)

Figure 6. Representative high Z prime indexing result of XXIX

We implemented PXRD comparison between the experimentally obtained PXRD and the simulated PXRDs of CSP-predicted crystal structures with comparably lower lattice energies. Since the theoretical PXRD is simulated at 0K, refinement can correct the changes of lattice cell and molecular structure caused by the temperature effect, allowing us to better compare theoretical and experimental PXRD patterns [18]. We used a powder pattern comparison algorithm published by de Gelder and coworkers in 2001 [19], which has been widely used in this area. After the Rietveld refinement, the theoretical PXRD of the most stable polymorph predicted by CSP with Z'=3 (namely data_1_z3_st_YSxRB1Za6zN2UQXt.cif in the submission data) has a good PXRD match compared to the experimentally observed PXRD. This is a strong indicator that we have identified

the experimental form as the most stable one in the current landscape. The PXRD overlay plot shown in Figure 7 presents the PXRD similarity between the experimentally obtained pattern and the theoretical PXRD of refined CSP-predicted form.

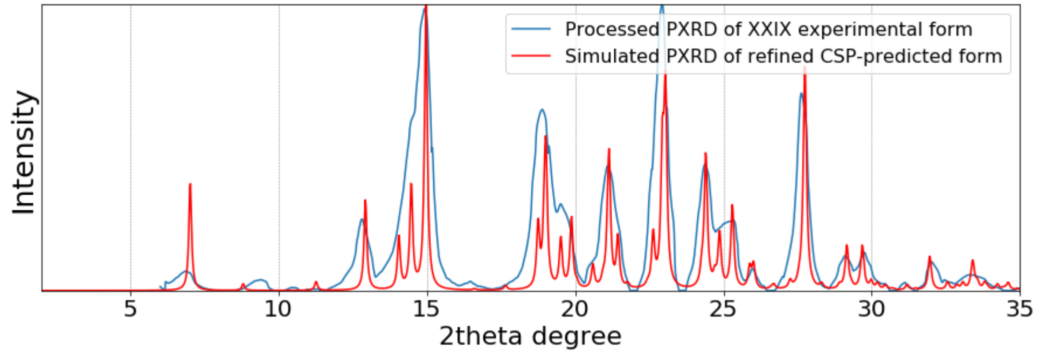

Figure 7. PXRD comparison between the experimental pattern and the theoretical PXRD of refined CSP-predicted form.

It has been noticed that the most plausible experimental form match, which is the rank 1 predicted form, data_1_z3_st_YSxRB1Za6zN2UQXt.cif, would have a layer of mismatch compared to the known experimental form if the matching criteria shrinking from RMSD30 35%/35° to RMSD90 25%/25°. We have performed RMSD30, RMSD60, and RMSD90 calculation using in-house RMSD comparison tool ILUM with 25%/25° distance and angle tolerance. As shown in the table below, a layer of mismatched molecules can be seen in the RMSD90 result.

Table 4. Alignment of experimental and predicted structures of target XXIX with increasing molecule shell size.

RMSD criteria	Molecules in common	RMSD value	Structural overlay
RMSD30	30 out of 30	0.216 Å	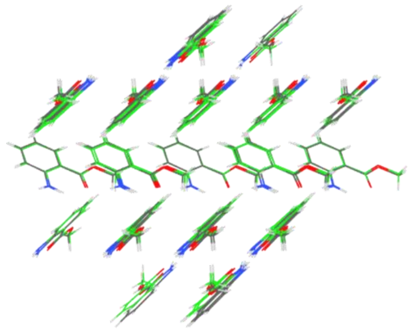

RMSD60	60 out of 60	0.239 Å	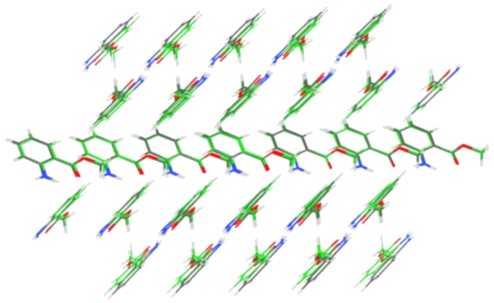
RMSD90	78 out of 90	0.243 Å	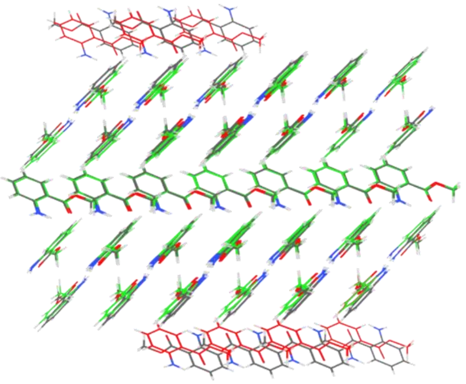

Upon examination, several structures resembling the experimental form were identified at earlier search and ranking stages, including data_1_z3_st_YSxRB1Za6zN2UQXt.cif and another form with a P 21/c space group (referred to as candidate 2). These stages encompassed force field ranking, semi-empirical ranking, and an intermediate DFT-D ranking stage with less stringent criteria. The crystal structure data_1_z3_st_YSxRB1Za6zN2UQXt.cif exhibited the lowest lattice energy among all predicted structures at the intermediate DFT-D ranking stage, while the candidate 2 structure demonstrated a relative lattice energy of approximately 3.2 kJ/mol compared to data_1_z3_st_YSxRB1Za6zN2UQXt.cif. Subsequent clustering calculations employing the RMSD15 40%/40° criteria revealed that these two polymorphs, along with several others, belonged to a single cluster group. Owing to their lattice energy disparities, data_1_z3_st_YSxRB1Za6zN2UQXt.cif was chosen as the representative polymorph for this cluster group and proceeded to the later DFT-D calculation with more stringent criteria. The candidate 2 structure was compared to the experimental form using the RMSD90 25%/25° criteria, revealing a commonality of 90 out of 90 molecules between the two structures. We state that this polymorph was successfully predicted; however, due to the lattice energy difference and clustering criteria, candidate 2 was not selected as the representative polymorph for this group and was not ultimately submitted as one of our results.

Clustering constitutes an essential stage in the crystal structure prediction (CSP) process. The standards and criteria for clustering calculations must be meticulously chosen, as they significantly influence the accuracy of the results and the efficiency of the calculations. In previous studies [5, 20], RMSD20 or RMSD30 have been commonly regarded as reasonable

criteria that strike a balance between accuracy and efficiency requirements. It is evident that adopting looser criteria would enhance the calculation efficiency, enabling the inclusion of more potentially stable polymorphs at earlier stages. However, this approach carries the risk of producing outcomes similar to those observed in the current system. Conversely, employing stricter criteria, such as RMSD90, may substantially increase computational costs and calculation wall time, rendering it unsuitable for all situations. As emphasized, it is crucial to systematically discuss and consider the clustering criteria at different stages, which warrants further exploration in subsequent studies.

Static and Dynamic Disorder Discussion

The disorder phenomenon has been widely observed in the experimental forms of the 7th Blind Test targets. More specifically, based on experimental CIF files provided by the 7th Blind Test team, Target XXVII Form A, Target XXX Form A, Target XXXI Form A, and Target XXXII Form A have disordered atoms/functional groups in the cell. Disorders in the experimental obtained single crystals can be classified as two different types: the static disorder, known as a crystal formed by APIs with different conformations; and the dynamic disorder, shown as terminal groups to be comparably flexible at higher temperatures. It is worth evaluating the disorder type of these disordered experimental forms to better validate the CSP result with experimental data. Molecular dynamic (MD) simulation would be a useful tool to analyze the disorder type since the dynamic disorder is expected to be captured by the simulation at higher temperatures. If the disordered group of a disordered experimental form is not comparably flexible during the MD simulation at higher temperatures, the corresponding disorder is considered to be a static disorder since the disordered atoms tend to remain unchanged at higher temperatures, supporting that the experimental form is formed by APIs with slightly different conformations. On the contrary, if the disordered terminal groups become more and more flexible as temperature increase, the experimental form is suspected to have a dynamic disorder because the temperature effect contributes to the flexibility of those terminal groups and result in atoms/terminal groups with different occupancy.

We have performed MD simulation for experimental forms with disorders, which are Target XXVII Form A, Target XXX Form A, Target XXXI Form A, and Target XXXII Form A. Here, we use the torsion angle distribution to demonstrate the disorder behavior at different temperatures. The change of selected torsion angles is directly correlated to the disorder behavior of each disordered group. Disordered groups and corresponding analyzed torsion angles are shown in table 5.

A significant disorder has been observed in the 290K experimental obtained crystal structure of XXVII Form A. The dihedral distribution of terminal carbon groups suggests that these carbon groups are comparably flexible during 300K MD simulation. This broad torsion angle distribution indicates that target XXVII Form A may have a dynamic disorder rather than a static disorder because the disorder can be captured using MD simulation at higher temperatures. Similar broad dihedral distribution was also observed in the XXX Form A and XXXII Form A. Interestingly, the MD simulation of XXX Form A indicates that the minor component could be the more preferable and observable component at higher temperatures, shown by the torsion angle distribution of XXX Form A major/minor at 300K: the terminal carbon tails in both major and minor component tend to have similar conformation with the minor component in the 300K MD simulation.

On the contrary, 300K MD simulation suggests the fluorobenzene ring is not able to flip to the other

side at higher temperatures, stating that the disorder in the experimentally solved crystal structure of target XXXI Form A could be a static disorder since the ring flipping energy barrier is still significant at higher temperatures. Please notice that due to the symmetry operation of the P 21/c space group, half of the APIs in the cells would have torsion angles with negative values compared to the other half of the APIs.

Table 5. Disorder analysis

Target	Disorder Group	Torsion Angle Distribution
XXVII Form A	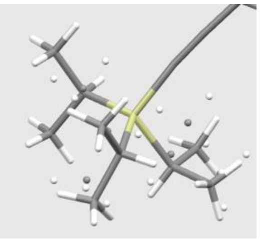	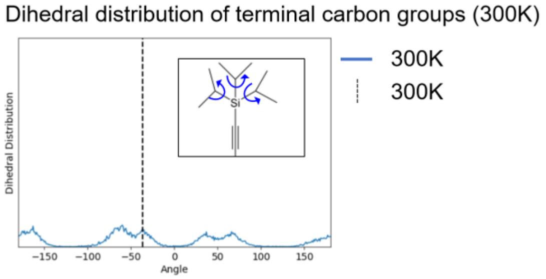
XXX Form A	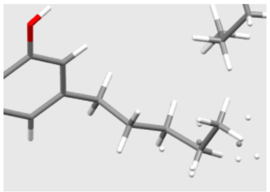	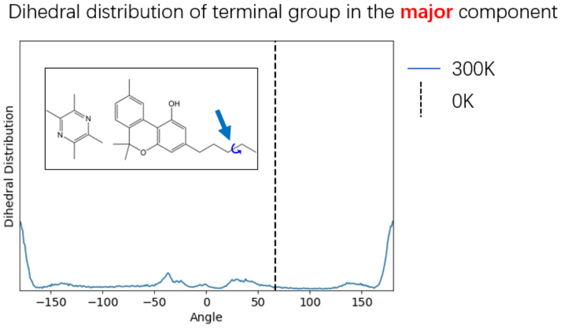 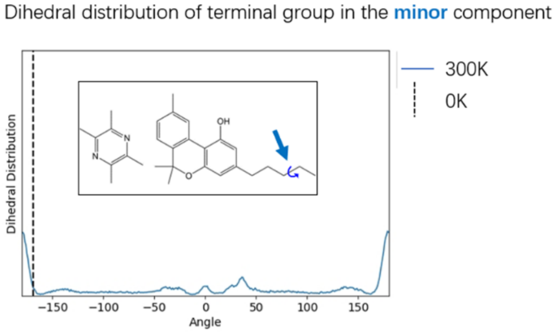

XXXI Form A	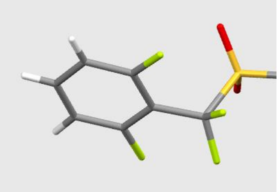	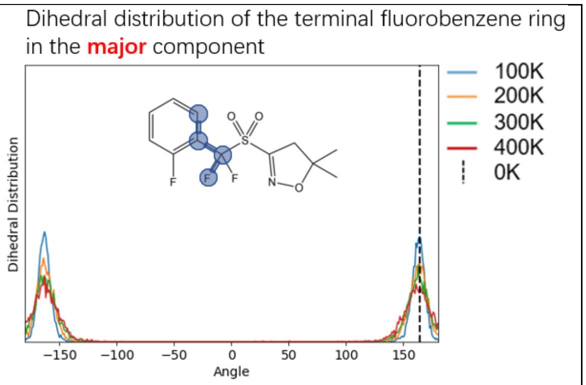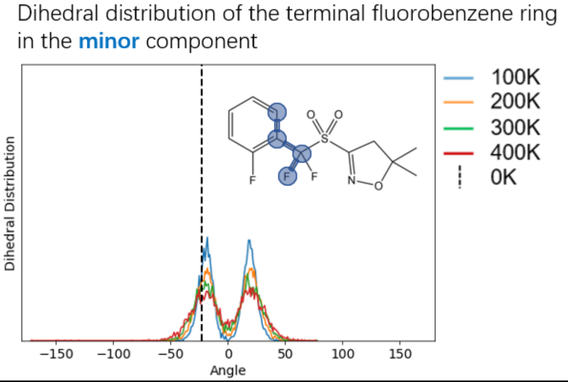
XXXII Form A	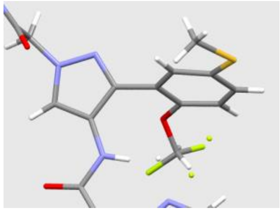	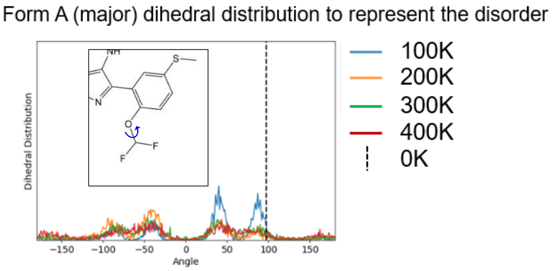

Reference:

1. Zhang, P., Wood, G. P. F., Ma, J., Yang, M., Liu, Y., Sun, G., Jiang, Y. A., Hancock, B. C., Wen, S. Cry. Grow. & Des. (2018), 18(11), 6891-6900
2. Teng, L., Fortunato, N. M., Opahle, I., Zhang, Y., Samathrakis, I., Shen, C., Gutfleisch, O., Zhang, H. npj Comp. Mat. (2021) 7, 66
3. Kim, B., Lee, S., Kim, J. Sci. Adv. (2020), 6, 1
4. Kim, S., Noh, J., Gu, G. H., Aspuru-Guzik, A., Jung, Y. ACS Cent. Sci. (2020), 6, 8, 1412–1420
5. Reilly, A. M., Cooper, R. I., Adjiman, C. S., et al. Acta Cryst. (2016). B72, 439–459
6. Wang, Y., Lv, J., Zhu, L., Ma, Y. Comput. Phys. Commun (2012), 183, 2063
7. Wang, Y., Lv, J., Zhu, L., Ma, Y. Phys. Rev. B (2010), 82, 094116
8. Cheng, G., Gong, X-G., Yin, W-J. arXiv (2011.10968)
9. Klimeš, J., Bowler, D. R., Michaelides, A. Phys. Rev. B (2011), 83, 19, 195131
10. Kresse, G., Hafner, J. Phys. Rev. B (1993), 47, 558
11. Kresse, G., Hafner, J. Phys. Rev. B (1994), 49, 14251

12. Kresse, G., Furthmueller, J. Comput. Mat. Sci. (1996), 6, 15
13. Sun, G., Jin, Y., Li, S., Yang, Z., Shi, B., Chang, C., Abramov, Y. A. J. Phys. Chem. Lett. (2020), 11, 20, 8832–8838
14. Wang, J., Wolf, R. M., Caldwell, J. W., Kollman, P. A., Case, D. A J Comput. Chem. (2004), 25, 1157–1174
15. Allen, A. E. A., Payne, M. C., Cole, D. J. J. Chem. Theory Comput. (2018), 9, 14(1), 274-281
16. Chisholm, J. A., Motherwell, S. J. Appl. Cryst. (2005), 38, 228–231
17. Li, X., Ou, X., Wang, B., Rong, H., Wang, B., Chang, C., Shi, B., Yu, L., Lu, M. Commun. Chem. (2020), 3(1), 152
18. Rietveld, H. M. J. Appl. Cryst. (1969), 2, 65-71
19. de Gelder, R., Wehrens, R., Hageman, J. A. J. Appl. Cryst. (2001), 34(3), 337-341
20. Sacchi, P., Lusi, M., Cruz-Cabeza, A. J., Nauha, E., Bernstein, J. Cryst. Eng. Comm (2020), 22, 717

7. Group 11

Description of Methods used in Phase 1 of the BT7 Submission

Sarah M. Clarke,[†] Adrian F. Rumson,[†] R. Alex Mayo,[†] Alberto Otero-de-la-Roza,[*,‡] and Erin R. Johnson[*,†]

Department of Chemistry, Dalhousie University, 6274 Coburg Rd, Halifax, Nova Scotia, B3H 4R2, Canada, and Departamento de Química Física y Analítica, Facultad de Química, Universidad de Oviedo, 33006 Oviedo, Spain

E-mail: aoterodelaroza@gmail.com; erin.johnson@dal.ca

Two conformers of compound XXIX were optimized using the B3LYP-XDM[1–3] functional and the 6-31+G* basis set with Gaussian 09 E.01.[4] Initial structure generation was performed using USPEX, version 10.4.[5–8] Six runs were performed, taking $Z = 4$ or $Z = 8$ with both conformers, and a 1:1 ratio of the two. A minimum of 10,000 structures were generated for each of the $Z = 4$ runs and a minimum of 6,000 were generated for each of the $Z = 8$ runs, using 100 structures per generation (30% from heredity, 50% random, and 10% each from soft mutation and rotation). The random structures were generated for the P1, P$\bar{1}$, P2_1, Pc, C2, Cc, C2/c, P2_1/c, P222, P222_1, P2_12_12, P$2_12_12_1$, and Pca2_1 space groups, which are common in molecular crystals. Antiseeds were used starting from generation 2, with "antiseedsMax" set to 0.01 and "antiseedsSigma" to 0.005. Volume estimates were 800 Å^3 for

*To whom correspondence should be addressed
†Dalhousie University
‡Universidad de Oviedo

$Z = 4$ and 2000 Å^3 for $Z = 8$. The "tiny" force field in Tinker[9] version 8.9.1 was used for rigid-molecule relaxation of the initial structures.

Subsequent rigid-molecule relaxation was then performed on all generated structures with DMACRYS[10] version 2.3.0 using the FIT potential. The B3LYP-XDM/6-31+G* conformational energy difference was added to the DMACRYS lattice energies to allow energetic comparison of all structures. All unique structures with DMACRYS energies within 3.5 kcal/mol of the minimum were carried forward to single-point energy evaluation with periodic-boundary DFT using Quantum ESPRESSO[11] versions 6.5 and 6.8. Structures were deemed to be duplicates if their volumes were identical to within 0.1 Å^3, their energies identical to within 0.01 eV, and their PXRD difference (POWDIFF) was less than 0.07. The POWDIFF values were determined from critic2[12] using the de Gelder's cross-correlation algorithm,[13] for 2θ between 5-50° and ideal Cu Kα radiation. The Gaussian broadening parameter was set to 0.05° and the triangle weighting used $\ell = 1°$.

All structures within 2.0 kcal/mol of the DFT minimum were carried forward to full DFT relaxations. The DFT calculations used the projector augmented-wave (PAW) approach,[14] the B86bPBE functional,[15,16] and the XDM dispersion correction.[17,18] Plane-wave cutoffs were set to 80 and 800 Ry for the wavefunction and density, respectively. Regular k-point meshes were selected automatically for each crystal using an R_k length parameter of 50 Bohr. The number of points (N_i) along each direction was determined from the reciprocal lattice vectors ($\mathbf{b}_i$, for $i = 1, 2, 3$) via the formula $N_i = \text{int}[\max(1, R_k|\mathbf{b}_i| + 0.5)]$. For geometry relaxation the convergence thresholds on the forces and energy were set to 10^{-4} Ry/bohr and 10^{-5} Ry, respectively, as in our previous work.[19,20]

References

(1) Becke, A. D. Density-functional thermochemistry. III. The role of exact exchange. *J. Chem. Phys.* **1993**, *98*, 5648–5652.

(2) Lee, C.; Yang, W.; Parr, R. G. Development of the Colle-Salvetti correlation-energy formula into a functional of the electron density. *Phys. Rev. B* **1988**, *37*, 785.

(3) Otero-de-la-Roza, A.; Johnson, E. R. Non-Covalent Interactions and Thermochemistry using XDM-Corrected Hybrid and Range-Separated Hybrid Density Functionals. *J. Chem. Phys.* **2013**, *138*, 204109.

(4) Frisch, M. J. et al. Gaussian 09 Revision E.1. Gaussian Inc. Wallingford CT 2013.

(5) Oganov, A. R.; Glass, C. W. Crystal structure prediction using *ab initio* evolutionary techniques: Principles and applications. *J. Chem. Phys.* **2006**, *124*, 244704.

(6) Oganov, A. R.; Lyakhov, A. O.; Valle, M. How Evolutionary Crystal Structure Prediction Works – and Why. *Acc. Chem. Res.* **2011**, *44*, 227–237.

(7) Lyakhov, A. O.; Oganov, A. R.; Stokes, H. T.; Zhu, Q. New developments in evolutionary structure prediction algorithm USPEX. *Comput. Phys. Commun.* **2013**, *184*, 1172–1182.

(8) Zhu, Q.; Oganov, A. R.; Glass, C. W.; Stokes, H. T. Constrained evolutionary algorithm for structure prediction of molecular crystals: methodology and applications. *Acta Cryst. B* **2012**, *68*, 215–226.

(9) Rackers, J. A.; Wang, Z.; Lu, C.; Laury, M. L.; Lagardère, L.; Schnieders, M. J.; Piquemal, J.-P.; Ren, P.; Ponder, J. W. Tinker 8: Software Tools for Molecular Design. *J. Chem. Theory Comput.* **2018**, *14*, 5273–5289.

(10) Price, S. L.; Leslie, M.; Welch, G. W. A.; Habgood, M.; Price, L. S.; Karamertzanis, P. G.; Day, G. M. Modelling organic crystal structures using distributed multipole and polarizability-based model intermolecular potentials. *Phys. Chem. Chem. Phys.* **2010**, *12*, 8478–8490.

(11) Giannozzi, P.; Andreussi, O.; Brumme, T.; Bunau, O.; Nardelli, M. B.; Calandra, M.; Car, R.; Cavazzoni, C.; Ceresoli, D.; Cococcioni, M. Advanced capabilities for materials modelling with Quantum ESPRESSO. *Journal of Physics: Condensed Matter* **2017**, *29*, 465901.

(12) Otero-de-la-Roza, A.; Johnson, E. R.; Luaña, V. Critic2: A program for real-space analysis of quantum chemical interactions in solids. *Comput. Phys. Commun.* **2014**, *185*, 1007–1018.

(13) de Gelder, R.; Wehrens, R.; Hageman, J. A. A generalized expression for the similarity of spectra: application to powder diffraction pattern classification. *J. Comput. Chem.* **2001**, *22*, 273–289.

(14) Blöchl, P. E. Projector augmented-wave method. *Phys. Rev. B* **1994**, *50*, 17953.

(15) Becke, A. D. On the large-gradient behavior of the density functional exchange energy. *J. Chem. Phys.* **1986**, *85*, 7184.

(16) Perdew, J. P.; Burke, K.; Ernzerhof, M. Generalized gradient approximation made simple. *Phys. Rev. Lett.* **1996**, *77*, 3865.

(17) Otero-de-la-Roza, A.; Johnson, E. R. Van der Waals interactions in solids using the exchange-hole dipole moment. *J. Chem. Phys.* **2012**, *136*, 174109.

(18) Johnson, E. R. In *Non-covalent Interactions in Quantum Chemistry and Physics*; Otero-de-la-Roza, A., DiLabio, G. A., Eds.; Elsevier, 2017; Chapter 5, pp 169–194.

(19) Whittleton, S. R.; Otero-de-la-Roza, A.; Johnson, E. R. The exchange-hole dipole dispersion model for accurate energy ranking in molecular crystal structure prediction. *J. Chem. Theory Comput.* **2017**, *13*, 441–450.

(20) Whittleton, S. R.; Otero-de-la-Roza, A.; Johnson, E. R. The exchange-hole dipole dispersion model for accurate energy ranking in molecular crystal structure prediction II: Non-planar molecules. *J. Chem. Theory Comput.* **2017**, *13*, 5332–5342.

8. Group 12

Supplementary information for 7th blind test of structure XXIX and XXXI

K. V. Jovan Jose and Gunjan R. Ramteke

School of Chemistry, University of Hyderabad, Hyderabad, India

Email: jovanjose@uohyd.ac.in

The sampling of molecular crystal structures over the entire configurational space is done through the method discussed in this article. The algorithm assumes one or more than one molecule in the asymmetric unit. Depending on the tendency of organic crystals to form crystals, the sampling is carried out on the 11 most significant space groups (p-1, p21, c2, cc, p21/m, p21/c, p21212, p212121, pna2, pnma, pbcn). A detailed description of the method is shown in Figure 1. The algorithm has two important events: the construction of the machine learning (ML) potential and the optimization of unit cell parameters.

Target XXIX and XXXI geometries have several isomers resulting from the conformational flexibility of molecules, obtained from the gas-phase optimization at B3LPY functional and basis 6-31G(d,p) uing Gaussian09 suite of programs. Along with the molecular geometry (M), the algorithm requires unit cell density cut-off (ρ_c) and molecular electrostatic potential (MESP) as the input. The unit cell density cut-offs are specific to the system and its respective space groups. MESP is a molecular scaler property and, classically, is defined at a point **r** as the energy required to bring a unit test positive charge from infinity to the point **r**, and mathematically is defined as

$$MESP(\boldsymbol{r}) = \sum_A \frac{Z_A}{|\boldsymbol{R}_A - \boldsymbol{r}|} - \int \frac{\rho(\boldsymbol{r}')d^3r'}{|\boldsymbol{r}' - \boldsymbol{r}|} \quad (1)$$

The MESP calculations were carried out at B3LYP functional and 6-31G(d) basis set using the G09 suite of packages. The crystal structure is described by multiple degrees of freedom arising from crystallographic unit cell parameters and the position of the asymmetric unit.

The initial orientation of the assymetric unit is considered from a set of random numbers. Space group symmetry-constrained sampling resulted in a large number of samples that must be screened before the energy analysis. One of the screening parameters is crystal density, which substantially influences qualities like morphology and energetics. The crystal structures within the specified density cut-off (ρ_c) are only accepted and passed to the next phase. Density cut-offs for the target XXIX are 8.65(p-1, p21, c2, cc), 17.31(p21/m, p21/c, p21212, p212121, pna21) and 34.63(pnma, pbcn). Similarly for XXXI it is 3.61(p-1, p21, c2, cc), 7.23(p21/m,

p21/c, p21212, p212121, pna21) and 14.46(pnma, pbcn). The other screening parameter is MESP growth potential, which measures the distribution of MESP on the opposite faces of the unit cell. It is observed in the experimentally obtained crystal structures that MESP distribution at the opposite faces of the unit cell is opposite, giving rise to the attractive interaction potential of the unit cell with the constituents of the neighbouring unit cell. Structures with a reasonable amount of growth potential are examined for energy evaluation. The pictorial represents growth potential in the crystal structure of XXIX crystallized in space group $P_{21,}$ as depicted in Figure 2. In contrast, the blue contour lines imply the positive potential, and red implies the negative.

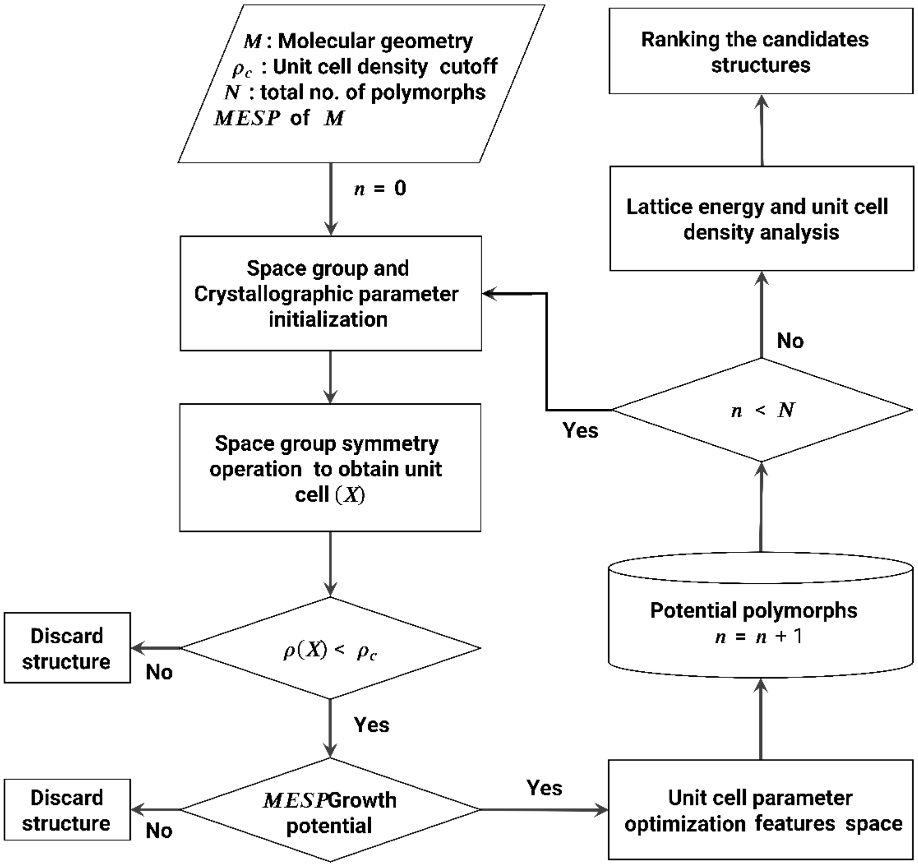

Figure 1 Flowchart of the algorithm employed for the polymorph search.

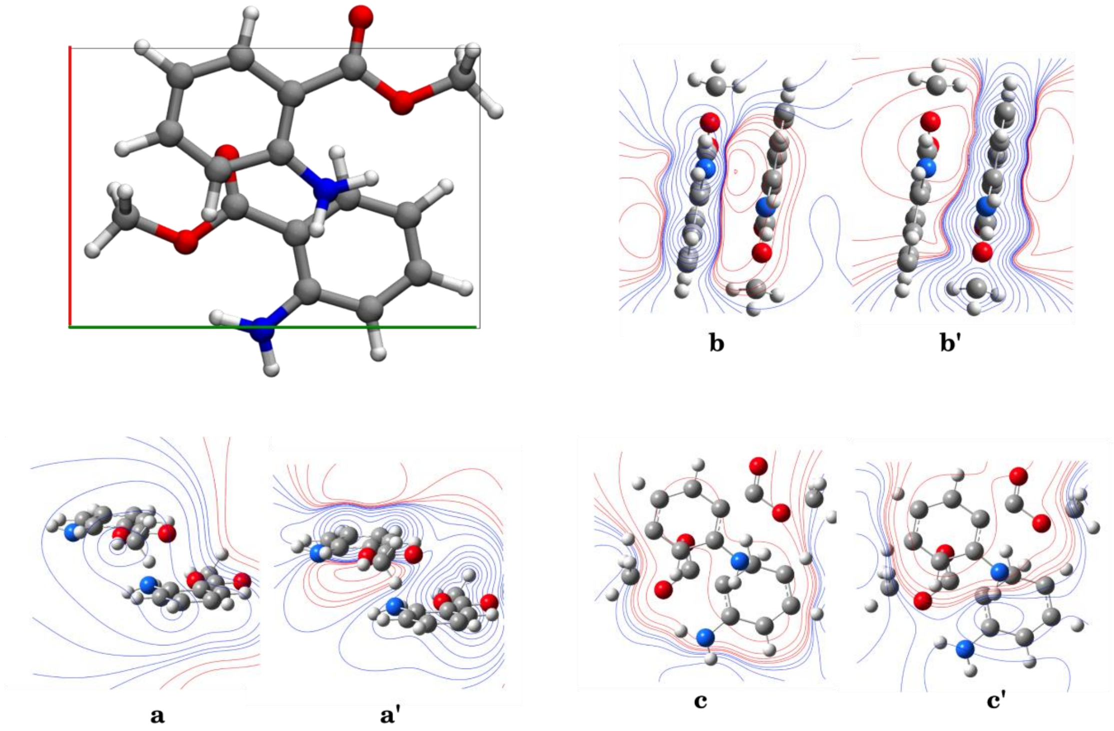

Figure 2 MESP distribution on opposite crystallographic faces. The blue contours display positive ESP potential, whereas the red contours display negative ESP potential.

The distribution of MESP through contours at the opposite faces enables one to forecast its stability, and based on which unfavourable geometries are discarded from the search.

Here, we attempted a distinct approach to assist the sampling procedure where specific features describe molecular information. Molecular features are the points located at the van der Waals region, specifically encrypting the MESP topographical information. These particles carrying MESP values are classified as positive or negative depending on the nature of the potential associated with them. The assessment of this topography points to the molecules transforming the unit cell to the feature space from cartesian space. Figure 3 illustrates the topography points assigned to the target XXIX and XXXI. In target XXIX, the molecules have Cs point group symmetry giving rise to a symmetrical arrangement of topography points. The interaction of each topographical feature point is measured by the feature vector that is constructed using equations (2 & 3); here, equation (3) defines the cut-off function.

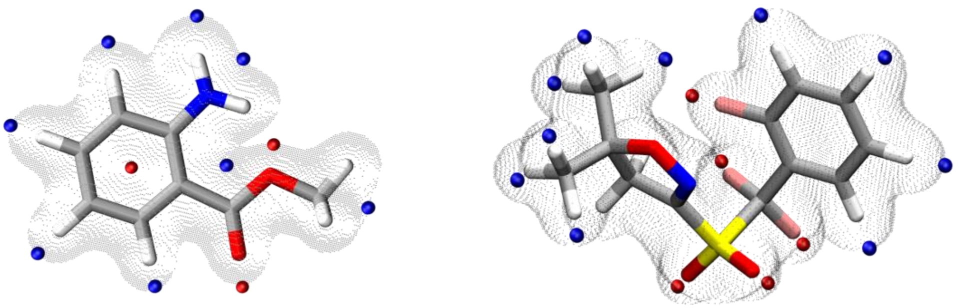

Figure 3 MESP topographical distribution of XXIX and XXXI targets. Blue indicates the positive MESP topography points, and red indicates the negative MESP topography points.

$$f_{v_i} = [\rho_i^p,\ \rho_i^n,\ Z_i] \tag{2}$$

$$\rho_i^Z = \sum_{j \neq i,\ z_j = z} e^{-r/\lambda}\ g_c(r) \tag{3}$$

$$g_c(r) = \begin{cases} \frac{1}{2} cos\left(\pi \frac{r}{r_c}\right) + \frac{1}{2}, & r \leq r_c \\ 0, & r > r_c \end{cases} \tag{4}$$

f_{v_i} is the feature vector; p and n are positive and negative topology points; Z is the type of topography point. The feature vector of interacting topographies is measured to receive guidance for the perform parameter optimization in cartesian space. The parameter optimization is pictorially represented in Figure 4. with crystal structure generated for XXIX in space group P_{21}. The three configurations show variations in the length of side 'a'. The features of majorly interacting topographies displayed in the consequent plots seem denser during optimization. For each optimization step, the energy is evaluated, and an energetically favourable configuration is accepted. Gaussian process regression model (GPR) fitted potential is employed to predict the reduced computational cost. The energies predicted by GPR are fair enough to provide an accurate relative description. GPR potential construction for the database exploits the sampling method where the energies are computed using density functional tight binding methods implemented in the DFTB+ package. The topographical evolution gives insight into the crystal packing and energetical stability. The predicted polymorphs are ranked according to the density of the unit cell.

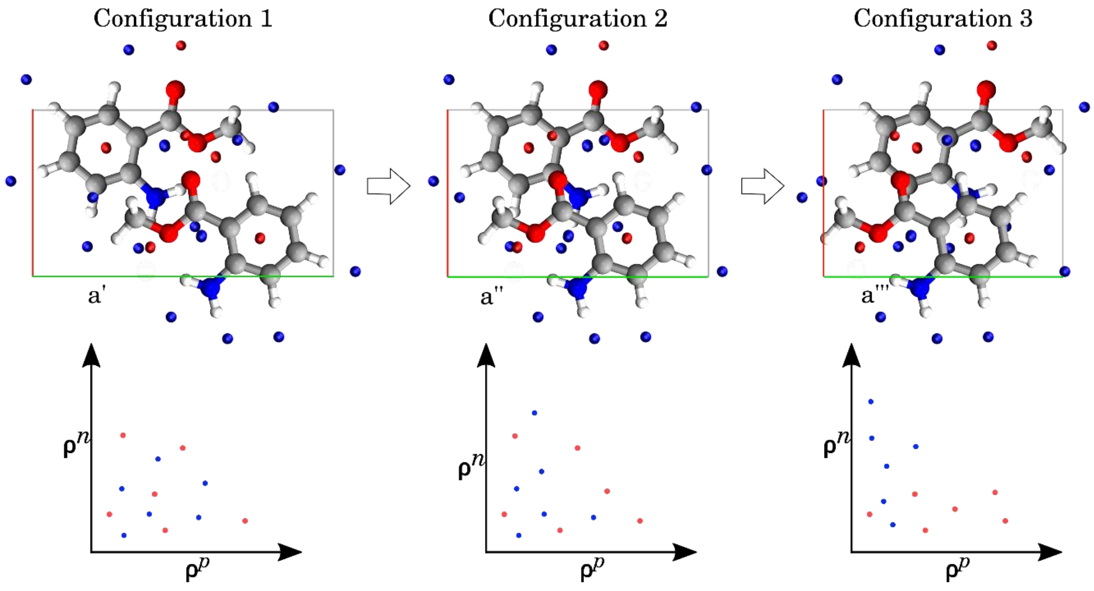

Figure 4 Parameter optimization guided by topographical evolution. The optimization of length 'a' in all the configurations is depicted with the feature getting denser.

.

Reference:

Gaussian 09, M. J. Frisch, G. W. Trucks, H. B. Schlegel, G. E. Scuseria, M. A. Robb, J. R. Cheeseman, G. Scalmani, V. Barone, B. Mennucci, G. A. Petersson, H. Nakatsuji, M. Caricato, X. Li, H. P. Hratchian, A. F. Izmaylov, J. Bloino, G. Zheng, J. L. Sonnenberg, M. Hada, M. Ehara, K. Toyota, R. Fukuda, J. Hasegawa, M. Ishida, T. Nakajima, Y. Honda, O. Kitao, H. Nakai, T. Vreven, J. A. Montgomery, Jr., J. E. Peralta, F. Ogliaro, M. Bearpark, J. J. Heyd, E. Brothers, K. N. Kudin, V. N. Staroverov, R. Kobayashi, J. Normand, K. Raghavachari, A. Rendell, J. C. Burant, S. S. Iyengar, J. Tomasi, M. Cossi, N. Rega, J. M. Millam, M. Klene, J. E. Knox, J. B. Cross, V. Bakken, C. Adamo, J. Jaramillo, R. Gomperts, R. E. Stratmann, O. Yazyev, A. J. Austin, R. Cammi, C. Pomelli, J. W. Ochterski, R. L. Martin, K. Morokuma, V. G. Zakrzewski, G. A. Voth, P. Salvador, J. J. Dannenberg, S. Dapprich, A. D. Daniels, Ö. Farkas, J. B. Foresman, J. V. Ortiz, J. Cioslowski, and D. J. Fox, Gaussian, Inc., Wallingford CT, 2009.

Hourahine, B., Aradi, B., Blum, V., Bonafé, F., Buccheri, A., Camacho, C., Cevallos, C., Deshaye, M. Y., Dumitric, T., Dominguez, A., Ehlert, S., Elstner, M., Van Der Heide, T., Hermann, J., Irle, S., Kranz, J. J., Köhler, C., Kowalczyk, T., Kubař, T., Lee, I. S.,

Lutsker, V., Maurer, R. J., Min, S. K., Mitchell, I., Negre, C., Niehaus, T. A., Niklasson, A. M. N., Page, A. J., Pecchia, A., Penazzi, G., Persson, M. P., Řezáč, J., Sánchez, C. G., Sternberg, M., Stöhr, M., Stuckenberg, F., Tkatchenko, A., Yu, V. W. Z. & Frauenheim, T. (2020). *J. Chem. Phys.* **152**, 124101.

Politzer, P. & Murray, J. S. (2002). *Theor. Chem. Acc.* **108**, 134–142.

Sørensen, K. H., Jørgensen, M. S., Bruix, A. & Hammer, B. (2018). *J. Chem. Phys.* **148**, 241734.

Steed, K. M. & Steed, J. W. (2015). *Chem. Rev.* **115**, 2895–2933.

9. Group 13

Supporting Information Document

Section 1. List of members

Authors: Dmitry Khakimov, Tatyana Pivina

Affiliation: N.D. Zelinsky Institute of Organic Chemistry, 47 Leninsky pros., 119991, Moscow, Russia

Section 2. Methodology

The methodology includes three stages: (1) calculations of the three-dimensional molecular structure, (2) construction of the MEP and determination of the optimal effective charges on atoms in molecules, (3) modeling of crystal packing. All quantum chemical calculations of molecular structures (first step) were performed by Gaussian 09 software package [1]. The DFT method with B3LYP functional and the aug-cc-PVDZ basis set with a Grimme's D2 dispersion correction was used. The electrostatic point charges (second step) were obtained by approximation of quantum mechanical electrostatic potential of the molecule with analytical potential of point charges on program FitMEP [2]. The optimal charges were formed both by the magnitude and by the atomic positions determined by the least squares procedure when their displacements in three dimensions were considered, which led to a significant improvement in the quality of the calculations. Subsequent scanning of the PES was carried out using the current version of the PMC software package [3], modified according to the original methodology [4-7]. The technique was tested on the results of previous Blind Test and showed that the model crystal packings coincide with the experimental ones without any preconditions.

The crystal structures (XXIX, XXX, and XXXIII) were generated using the PMC program [3] to globally minimize the energy of the considered organic molecular crystal. Semi-empirical intermolecular potential functions included non-valence interactions of 6-12 Lennard-Jones atom-atom potentials for van der Waals energy and electrostatic interactions of point charges placed on atoms or their auxiliary sites defined in the MEP. The best model of extra-atomically centered charges found for all test target molecules was, at least, several times better in RMSD compared to models with fixed charges on atoms. For a salt with sulfur atoms, the 6-exp-potential model was used with the parameters given in Table 4.

The PMC program makes it possible to take into account the harmonic potential functions for valence-bonded atoms and auxiliary force centers of rigid fragments in a conformationally flexible molecule. Further, when modeling the crystal structure, the molecules were assumed to be solids without taking into account the mobility of fragments. As it turned out later, the

mobility correction is critical for finding the correct packing, given in this case the incorrect definition of the conformer.

It should be noted that both intra- and intermolecular contributions to the total energy of both van der Waals and electrostatic terms were calculated with the same set of parameters of the atom-atom potential and electrostatic charges.

The space groups $P2_1/c$, $P2_12_12_1$, P-1, $P2_1$, Pbca, C2/c, $Pna2_1$, Pnma, $Pca2_1$, Cc, C2, P1, $P2_1/m$, Pbcn, Pc, $P4_12_12$, $P4_1$, Pccn, Fdd2, $Cmc2_1$, $P3_1$, R-3, $P2_12_12$, $I4_1/a$, $P6_1$, P42/n, Pbcm, C2/m, $Pmn2_1$, Iba2, $P42_1c$, R3, $P3_121$, P2/c, $C222_1$ with one independent molecules in the cell were tried for XXIX. Space groups $P2_1/c$, $P2_12_12_1$, P-1, $P2_1$, Pbca, C2/c, $Pna2_1$, $Pca2_1$, Cc, C2, P1 with two independent molecules in the cell were tried for XXIX, XXX (co-crystal), XXXIII (salt).

For cocrystal XXX in a ratio of 1:2 and 2:1, the following groups were used: $P2_1/c$, $P2_12_12_1$, P-1, $P2_1$, Pbca, C2/c, $Pna2_1$, $Pca2_1$, Cc, C2, P1, $P3_1$, R-3, Pbcn, Pc.

The starting molecular orientations were selected from the list of 1080 matrices providing a kind of regular 'grid' of the full rotation space in the three dimensions (with the separation angle ω =27.78° between any two nearest-neighbor orientations). On account of the crystal and approximate molecular symmetry the number of starting orientations for global minimization is effectively reduced [8].

All initial models were energy-optimized when the molecules were represented as rigid bodies, taking into account six more degrees of freedom for each independent molecule in the u.c., describing the positional and orientational coordinates of the molecules. Energy-minimized structures were sorted by energy in each space group and processed by the CRYCOM crystal structure comparison program [9] to create a list of unique structures and select the desired one from it.

Section 3. The structure of the compounds in the blind test

For compound XXIX, various conformers were considered, of which it is worth highlighting the two most energetically favorable structures with planar molecules, but with different orientations of the COOMe group. For calculations, a model was chosen with a conformation in which the COOMe group is the most distant from the NH_2 group (the smallest distance between H2B and O4), which turned out to be energetically more favorable by 3.02 kcal/mol than the structure with the opposite orientation of this group (the smallest distance between H2B and O3).

In cocrystal XXX, the point group for the cannabinoid structure is C1, and for tetramethylpyrazine D2h. The alkyl “tail” in the cannabinoid is mobile (groups C18 - C21). For

calculations, a conformation with the configuration of a normal (zigzag form) alkyl chain along the C3-C17-C18-C19-C20-C21 direction was chosen.

For salt XXXIII, the structure of the morpholinium cation is planar with a Cs point group. A search for an anion with a sulfonyl group also revealed a planar Cs group (which turned out to be different from the experimental one).

Section 4. Post-analysis of test results

Analysis of the structures after the presentation of the results revealed the following errors, which prevented the correct prediction of crystal packings.

For structure XXIX, the geometry was correctly determined, however, we used calculations with only one and two independent molecules in the unit cell, since there were no three molecules in the tests before (like in experiment).

A blind test with Z'=3 showed that the model packing is close in character to the experimental crystalline packing, but there is a difference in the alternation of layers of independent molecules, although the cell parameters, as well as the space group, are close to each other (Table 1 and Figure 1).

Table 1. Unit cell of the experimental and post-analysis model of target XXIX.

XXIX	Experimental	New model
cell_length_a	25.1585(5)	23.623
cell_length_b	9.9830(2)	9.810
cell_length_c	9.3583(2)	9.428
cell_angle_alpha	90	90
cell_angle_beta	93.258(2)	93.50
cell_angle_gamma	90	90

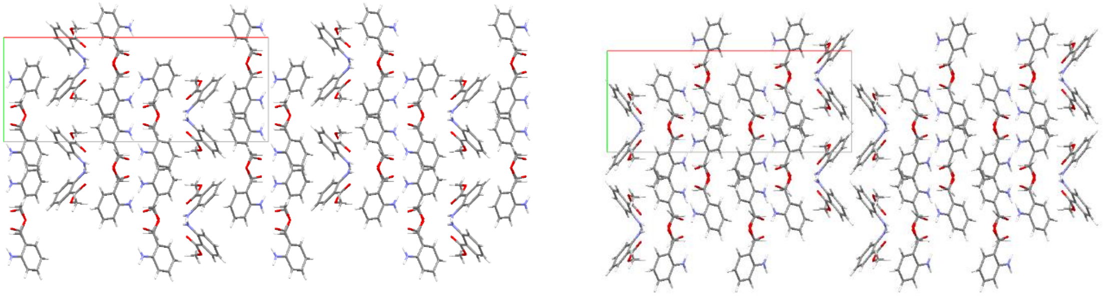

Experimental XXIX New model XXIX

Figure 1. Crystal packings of the experimental and post-analysis model of target XXIX.

For structure XXX, there is also a problem with geometry, where the group in the cannabinol fragment has a different torsion angle.

In structure XXX_B, the alkyl chain has a break at the C18 atom. This conformation is 0.97 kcal/mol less favorable than the one we considered initially. However, the lattice energy for it exceeds the energy difference of molecules in the gas phase. Recalculation with the new geometry of the molecule gave good agreement with the XXX_B structure (Table 2).

The XXX_A structure contains three independent molecules: tetramethylpyrazine with the D2h point group and two molecules with the cannabinoid structure, but with different directions of the alkyl chain (opposite direction along the C20-C21 atoms).

We modeled 1:1 cocrystal, which completely coincided with the result of X-ray diffraction analysis (Table 2).

Table 2. Unit cell of the experimental and post-analysis model of target XXX_B.

XXX_A	Experimental	New model
cell_length_a	14.8774(5)	15.296
cell_length_b	10.1190(3)	10.434
cell_length_c	17.5619(6)	17.713
cell_angle_alpha	90	90
cell_angle_beta	104.4280(10)	103.22
cell_angle_gamma	90	90

Post analysis of other ratios in the cocrystal was not carried out by us and will be the task of the following studies.

The best developed and tested methods for blind modeling of organic compounds include atom-atom potentials for C, H, N, O atoms. We tried to make a model for sulfur atoms, taking into account the new anion geometry in salt XXXIII, which was given as experimental.

The new structure in the gas phase from the experiment is less favorable by 17 kcal/mol, but the gain in lattice energy for it is 5 kcal/mol. This means that the total energy is still less for a structure with the Cs group than with C1 and leads us to the mandatory use of the torsion term in the energy equation in the future to find the correct result.

It should be noted that the blind testing approach for the new geometry correctly identifies the experimental packing as the first polymorph (Table 3).

Table 3. Unit cell of the experimental and post-analysis model of target XXXIII.

XXXIII	Experimental	New model
cell_length_a	11.7407(2)	12.398
cell_length_b	12.4207(2)	12.489
cell_length_c	11.3775(2)	10.816
cell_angle_alpha	90	90
cell_angle_beta	90	90
cell_angle_gamma	90	90

Thus, in the case of non-rigid molecules, taking into account the torsion term in the energy formula begins to play a decisive role. In the case of simple molecules that do not have degrees of freedom for rotation, the predictions give an exact result.

Section 5. CPU resources and hardware used

Most calculations have been performed on the computer 'mvs100k' of the Joint Supercomputer Center of the Russian Academy of Sciences (JSCC RAS) [10]. The numbers of parallel processors, *N*p, of Intel Xeon 5450 type (operating on GHz) used in these computations varied from 16 to 32. The CPU times (as rescaled to *N*p = 96) have amounted approximately 250 hours for three target structures XXIX, XXX and XXXIII.

Acknowledgement

The authors are deeply grateful to Dr. Alexandr V. Dzyabchenko for the provided programs for crystal structures simulation. The supercomputer resources were provided by the HPC centers of N. D. Zelinsky IOC RAS and 'MVS100K' of Russian Academy of Science.

References

[1] M. J. Frisch, G. W. Trucks, H. B. Schlegel, G. E. Scuseria, M. A. Robb, J. R. Cheeseman, G. Scalmani, V. Barone, G. A. Petersson, H. Nakatsuji, X. Li, M. Caricato, A. Marenich, J. Bloino, B. G. Janesko, R. Gomperts, B. Mennucci, H. P. Hratchian, J. V. Ortiz, A. F. Izmaylov, J. L. Sonnenberg, D. Williams-Young, F. Ding, F. Lipparini, F. Egidi, J. Goings, B. Peng, A. Petrone, T. Henderson, D. Ranasinghe, V. G. Zakrzewski, J. Gao, N. Rega, G. Zheng, W. Liang, M. Hada, M. Ehara, K. Toyota, R. Fukuda, J. Hasegawa, M. Ishida, T. Nakajima, Y. Honda, O. Kitao, H. Nakai, T. Vreven, K. Throssell, J. A. Montgomery, Jr., J. E. Peralta, F. Ogliaro, M. Bearpark, J. J. Heyd, E. Brothers, K. N. Kudin, V. N. Staroverov, T. Keith, R. Kobayashi, J. Normand, K. Raghavachari, A. Rendell, J. C. Burant, S. S. Iyengar, J. Tomasi, M. Cossi, J. M. Millam, M. Klene, C. Adamo, R. Cammi, J. W. Ochterski, R. L. Martin, K. Morokuma, O. Farkas, J. B. Foresman, D. J. Fox, Gaussian 09, Revision D.01, Gaussian, Inc., Wallingford CT, **2016**.
[2] A. V. Dzyabchenko, A multipole approximation of the electrostatic potential of molecules, *Russ. J. Phys. Chem. A.*, **2008**, 82(5), 758–766.
[3] A. V. Dzyabchenko, From molecule to solid: the prediction of organic crystal structures, *Russ. J. Phys. Chem. A*, **2008**, 82(10), 1663–1671.
[4] D. V. Khakimov, V. P. Zelenov, N. M. Baraboshkin, T.S. Pivina, The unusual combination of beauty and power of furoxano-1,2,3,4-tetrazine 1,3-dioxides: a theoretical study of crystal structures, *J. Mol. Model.*, **2019**, 25, 107-115.
[5] D. V. Khakimov, A. V. Dzyabchenko, T. S. Pivina, Crystal structure prediction of bifurazano[3,4-b:3',4'-f]furoxano[3'',4''-d]oxacycloheptatriene (BFFO) in the experimentally known monohydrated and proposed anhydrous forms, *Propellants, Explosives, Pyrotechnics*, **2019**, 44, 1528-1534.

[6] D. V. Khakimov, A. V. Dzyabchenko, T. S. Pivina, Computer simulation of the crystal structure of tetrazino-tetrazine tetraoxide (TTTO) isomers with one and two independent molecules in the unit cell, *Russian Chemical Bulletin*, **2020**, 69, 212-217.
[7] V. P. Zelenov, N. M. Baraboshkin, D. V. Khakimov, N. V. Muravyev, D. B. Meerov, I. A. Troyan, T. S. Pivina, A. V. Dzyabchenko, and Ivan V. Fedyanin, Time for quartet: the stable 3:1 cocrystal formulation of FTDO and BTF – a high-energy-density material, *CrystEngComm.*, **2020**, 22, 4823 – 4832.
[8] A. V. Dzyabchenko, Symmetry of the lattice-energy functional of a molecular crystal, *Acta Cryst. A*, **1983**, 39(6), 941-946.
[9] A. V. Dzyabchenko, Method of crystal-structure similarity searching, *Acta Cryst. B*, **1994**, 50(4), 414-425.
[10] http://www.jscc.ru/resources/hpc/

Appendix

Here we provide more details on the intermolecular potentials that we used in the generation of structures and the ranking of the XXXIII target.

Table 4. Parameters of the 6-exp atom-atom potentials used with XXXIII.

Atoms, *A*, *B*, α

S	S	4.09175	-0.16385	3.30000
S	H	3.67364	-0.04632	3.52000
S	C	3.98823	-0.12589	3.45000
S	N	3.85053	-0.11675	3.54000
S	O	3.70266	-0.13097	3.63000
S	N'	3.85053	-0.11675	3.54000
S	H*	2.68000	-0.06140	0.00000
H	H	3.31075	-0.01276	3.74000
H	C	3.60620	-0.03424	3.67000
H	N	3.48945	-0.03137	3.76000
H	O	3.36118	-0.03477	3.85000
H	N'	3.48945	-0.03137	3.76000
H	H*	2.80500	-0.04670	0.00000
C	C	3.89828	-0.09497	3.60000
C	N	3.77255	-0.08711	3.69000
C	O	3.63677	-0.09639	3.78000
C	N'	3.77255	-0.08711	3.69000
C	H*	3.19000	-0.05970	0.00000
N	N	3.65446	-0.07935	3.78000
N	O	3.52604	-0.08731	3.87000
N	N'	3.65446	-0.07935	3.78000
N	H*	3.33500	-0.04450	0.00000
O	O	3.40459	-0.09569	3.96000
O	N'	3.52604	-0.08731	3.87000
O	H*	1.90000	-1.11000	0.00000
N'	N'	3.65446	-0.07935	3.78000
N'	H*	1.96000	-0.90000	0.00000
H*	H*	2.68000	-0.06140	0.00000

10. Group 16

Supplementary Information

Dylan M. Anstine[1], Dana O'Connor[2], Roman Zubatyuk[1], Rithwik Tom[3], Imanuel Bier[2], Kamal S. Nayal[1], Yi Yang[2], Wenda Deng[2], Kehan Tang[2], Olexandr Isayev[1], and Noa Marom[1,2,3]

[1] Department of Chemistry, Carnegie Mellon University, Pittsburgh, Pennsylvania 15213, USA
[2] Department of Materials Science and Engineering, Carnegie Mellon University, Pittsburgh, Pennsylvania 15213, USA
[3] Department of Physics, Carnegie Mellon University, Pittsburgh, Pennsylvania 15213, USA

Overview of Methodology

Our general approach to predict crystal structures for the targets XXVII, XXIX and XXXI was to perform geometry optimization for a large number of randomly generated trial structures and select the structures with low static energy. To speed up the calculations we trained target-specific AIMNet neural network potential models, which approximate the DFT potential energy surface. This way, we were able to explore millions of trial structures for each of the targets. For the targets XXIX and XXXI we carried out DFT optimizations for the best structures found with the AIMNet potential. For the target XXVII, due to the size of the molecule, the submitted ranking was solely based on AIMNet results. The matching with diffraction data was performed for the best structures found with AIMNet and DFT optimizations.

Conformer Generation

For each of the targets, a conformer pool was generated before being passed to our random crystal structure generator, Genarris[1]. Conformer generation was target dependent, as detailed in the bulleted list below:

- Target XXVII: A dense conformational ensemble of molecules was prepared in two stages. First, a set of 50 conformations was generated using the RDKit ETKDGv2 method, which produces random rotations around triple bonds. Then for each conformer a systematic search for all possible rotations of i-Pr groups was performed with the Confab algorithm as implemented in Openbabel.[2] The final conformation pool contained a total of 1.8×10^4 conformations.
- Target XXIX: Conformations were obtained with the Bayesian Optimization using Knowledge of correlated tensions (BOKEI)[3] method and optimized with B97-3c.[4] Generation of the asymmetric unit for Z'=2 search was performed by sampling all possible molecular dimer geometries in a grid search method for each low-energy conformation. The dimer geometries were then optimized and ranked in energy by a system specific AIMNet potential (see Section 3). All dimers with relative energy of less than 5.5 kcal/mol and all hydrogen-bonded dimers with relative energy less than 8.5 kcal/mol were used for Z'=2 crystal structure generation. This resulted in 660 dimer geometries.
- Target XXXI: A dense conformational ensemble for this target was generated using Openeye OMEGA software by driving rotatable torsion angles.[5] A total of 658 conformers was generated with an energy cutoff of 15.0 kcal/mol and 0.3 Å RMSD similarity threshold.

Genarris

Genarris begins generation with an estimation of the unit cell volume, for which we have developed a machine learned model.[6] Next, Genarris automatically determines all compatible space groups, including occupation of special positions.[1] While Genarris can be used to generate in specified space groups, we generated in all compatible space groups. Genarris then selects a compatible space group and generates a structure according to space group symmetry within a volume distribution around the predicted unit cell volume. The first molecule is randomly placed in the unit cell. The remaining molecules are placed using space group symmetry operations. For this blind test we used a development version of Genarris that

generates structures using pools of different conformers by randomly selecting a conformer from the pool for each generation attempt. The number of crystal structures generated was system dependent. For target XXVII, a total of 4 x 10^6 and 1 x 10^6 structures were generated for Z=2 and Z=4, respectively. During the crystal structure generation process for target XIX, we used a single XXIX molecule as well as systematically constructed dimers as the structural units. To ensure reasonable structures, dimers were preoptimized with the AIMNet potential and filtered by energy. A total of 6.4 x 10^6 structures were then generated for Z' = 1, 2 and for Z = 1, 2, 3, 4, 6, 8, 12, 16 and 18. Crystal structure energies of these systems were further evaluated with the AIMNet potential for structures generated from 2 planar and 3 non-planar conformations. Only one planar conformer with the N-H...O=C intramolecular hydrogen bond resulted in low energy crystal structures. For target XXXI, 1.7 x 10^6 structures were generated for Z=2, 4, and 8. The crystal structures produced for all targets were checked to ensure that they were physically reasonable. Briefly, Genarris performs this analysis by evaluating the comparison

$$d_{A,B} > s_r(r_A + r_B) \qquad (1)$$

where $d_{A,B}$ is the distance between molecules A and B, s_r is the specific radius ratio (chosen as 0.7), and r_A and r_B are the van der Waals radii of molecules A and B, respectively.[1]

AIMNet2

AIMNet (atoms-in-molecules) is a chemically inspired, modular deep neural network (DNN) molecular potential that utilizes multimodal and multitask learning to obtain an information-rich representation of an atom in a molecule[7]. The design of AIMNet is partially motivated by Bader's theory of atoms-in-molecules (AIM)[8] in which a molecule can be partitioned into interacting atoms via an observable electron density distribution function. In the AIMNet model, instead of electron density, atoms are characterized by learnable feature vectors and complex interatomic interactions are learned using a DNN. These feature vectors are updated iteratively during the forward pass of the DNN, which is a process that is inspired by the neighbor-driven changes of electron density distribution within atomic basins. The model transforms atomic coordinates (R) and atomic numbers (Z) into atomic environment vectors (AEVs) which are then embedded as features and allowed to interact through iterative updates (referred to as message passing). The model can then predict molecular and/or atomic properties using these learned representations.

In this work, our most recent developmental version of the AIMNet2 model was trained to learn diverse atomic or molecular properties: namely, the total molecular interaction energy, components of the atomic forces, and atom-centered partial charges calculated using Hirshfeld partitioning. The models were trained to reproduce DFT-calculated properties and include explicit long-range charge-charge Coulomb dispersion interactions.

Target specific AIMNet models were trained for XXVII, XXIX, and XXXI using N-mer sampling. Briefly, N-mers were extracted from the generated crystal structures (N=1, 2, 3, 4, and 8) and subjected to gas phase DFT calculations at the B97M-D4/def2-TZVPP level of theory with Orca v5.0.3[9] to form a training dataset. In the interest of computational efficiency, we first trained low-level accuracy models using energies, atomic forces, and partial charges from GFN2-xTB[10] molecular dynamics trajectories on the extracted N-mers. Our preliminary testing showed that initializing AIMNet's weights using low accuracy tight binding data prior to training to more accurate DFT calculations (B97M-D4/def2-TZVPP) assisted in stabilizing training and accelerating convergence. It is worthwhile to highlight that the final AIMNet models consisted of training using only DFT level data and all tight binding data was removed from the training dataset. The final models were trained on monomers, dimers, trimers, tetramers, and

octamers of each target molecule, and they were used to optimize the randomly generated trial crystal structures (refer to Table 1).

Table 1 Summary of target specific AIMNet training iterations and the final number of crystal structures each model was able to optimize.

Target	Active Learning Iterations	Final Composition of Training Set (10^3)	Crystal Structures Optimized (10^6)
XXVII	8	13 monomers<br>75 dimers<br>7 trimers	5
XXIX	3	0.184 monomers<br>55 dimers<br>64 trimers<br>44 tetramers<br>1.5 octamers	6.4
XXXI	4	6.3 monomers<br>125 dimers<br>76 trimers<br>14 tetramers	3.5

Post-Processing

For Target XXVII, optimized structures were symmetrized using Spglib with the tolerance (symprec*)* of 0.2 Å and the duplicate structures were removed using Pymatgen[11] StructureMatcher with a site tolerance of 0.2 Å and default lattice and angle tolerances (*stol=0.2, ltol=0.2, angle_tol=5*). The corresponding submission included a list of the best 1500 structures ranked by the lattice energy. For the other two targets, the best structures were selected for DFT calculation and re-optimized with the PBE-MBD method using the FHI-aims[12] version 200112 with the tier-1 basis set, 3x3x3 k-grid and light numerical settings. The MAE and RMSE errors of the AIMNet model with reference to DFT on the relative lattice energies came out to be 2.0 kJ/mol and 3.2 kJ/mol (Target XIX) and 2.5 kJ/mol and 3.8 kJ/mol (Target XXXI) respectively. The AIMNet model systematically overestimated the crystal density by 4.1 - 4.2% on average compared to DFT but once that was accounted for, the RMS error of AIMNet predicted crystal density reduced to 1.4 - 1.5% with respect to DFT. DFT calculations were performed for a subset of the best crystal structures predicted by the AIMNet model for the Targets XIX and XXXI. The final submission included a mixture of both AIMNet and DFT optimized structures, identified by the prefix 'aimnet' or 'dft' correspondingly.

Results and Analysis

Genarris proved very successful for the attempted targets XXVII, XXIX, and XXXI. Table 2 shows the number of molecules and Z' searched for each target, the total number of structures generated, and if the experimental form(s) were identified. As can be seen, Genarris generated all but one experimental form, the C form for Target XXXI, which was a solvent stabilized structure.

Table 2 Summary of crystal structure search using Genarris. All targets were identified other than the XXXI C, which was discolsed as being solvent stabilized.

Target	Z searched	Total Number of Structures (10^6)	Exp. Identified (Y/N)
XXVII	z = 2,4	5.0	Y
XXIX	z' = 1,2<br>z=1,2,3,4,6,8,12,16,18	6.4	N*
XXXI	z=2,4,8	1.7	Y (A major, A minor, B)<br>N (C)

* For target XXIX we did not explore crystal structures with Z'=3 and therefore, the experimental structure was not identified. However, our closest matching structure has a partially common motif with the experimental one and CSD structure. Also, our AIMNet model predicts ~5 kJ/mol lower energy for experimental Z'=3 structure compared to the best submitted structure.

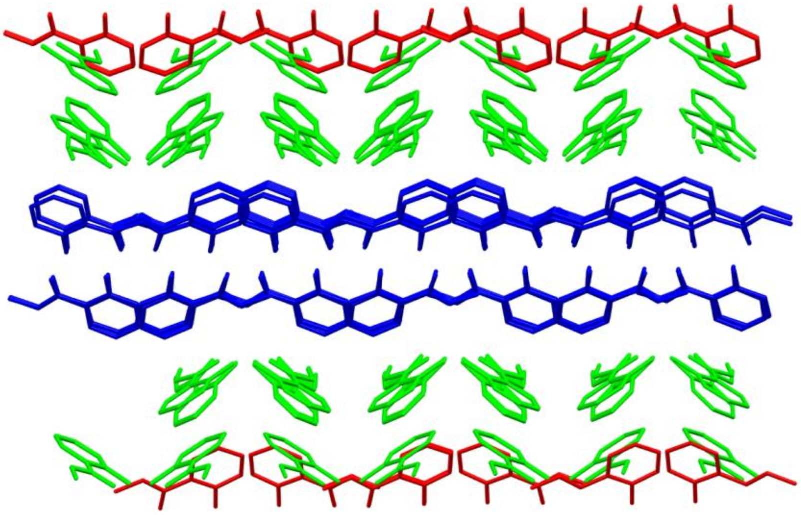

Figure 1: Overlap of the lowest energy generated structure via our CSP methods and the experimental structure for the target XXIX. Molecules are colored be symmetry equivalence.

We did not perform any trials to generate structures with Z'=3 for target XXIX in our CSP workflow, and therefore, we did not find the experimentally identified structure. However, our best structure match, which has Z'=2, exhibits significant similarity with the experimental structure. Figure 1 shows the overlap between the top ranked structure in our submission list ant the experimental structure, where the molecules are colored by symmetry equivalence. Visually, it is clear that our generated structure and the experimental one has common domain containing molecules A and B (blue and green) of the experimental structure, however the molecule C (red) has different orientation. To quantify the agreement of the matching domain, the RMSD of the structures was determined using the COMPAC matching algorithm implemented in CCDC Mercury[13], where selecting 15 molecule clusters yields 0.354 Å (all 15 molecules matched). Simulated powder X-ray diffractograms for the experimental structure and our best matching structure are compared to the provided experimental diffractogram in Figure 2.

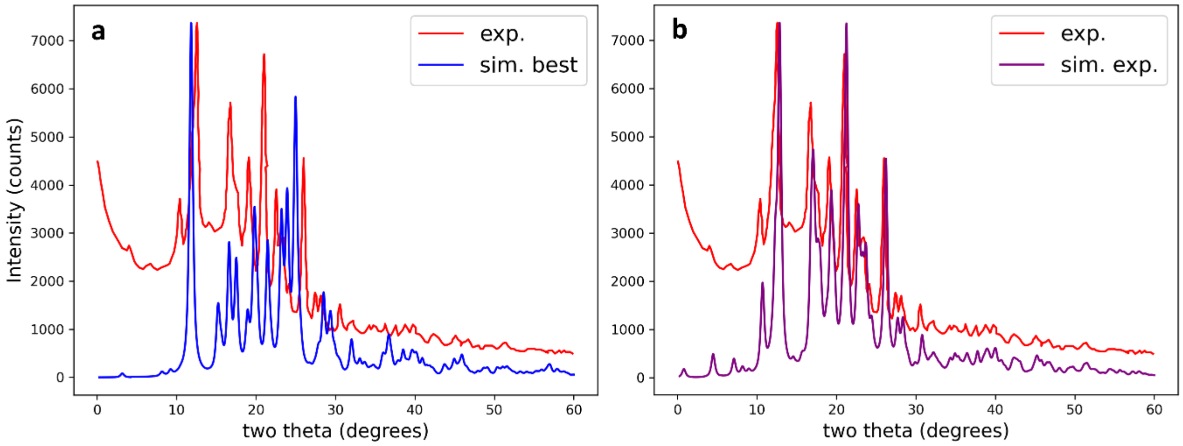

Figure 2: Simulated powder X-ray diffraction (PXRD) spectra for Target XXIX. a) the experimentally obtained PXRD (red) compared to the simulated PXRD of our best matching structure (blue). b) the experimentally obtained PXRD (red) compared to the simulated PXRD of the experimental structure (purple).

For Target XXVII, energy is not necessarily correlated with density. Figure 3 shows the relative lattice energy of the generated structures for Target XXVII as a function of density, with the generated experimental structure by Genarris shown in blue. As can be seen, the experimental form is not ranked as a low energy structure, lying between 12-15 kJ/mol above the minimum energy structure.

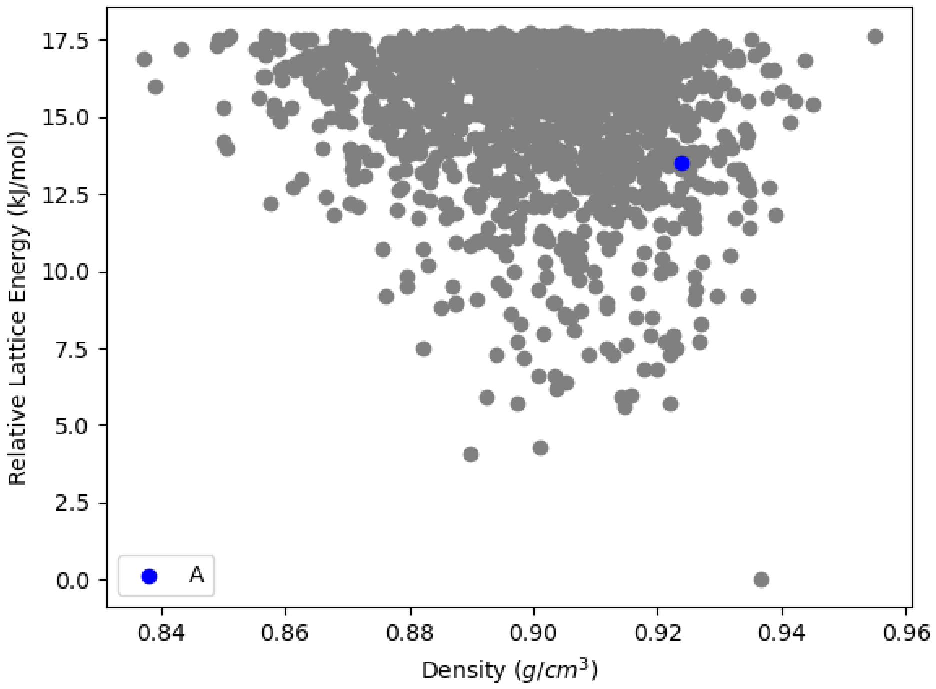

Figure 3: Relative lattice energy calculated using AIMNet as a function of density for the 1500 structures of Target XXVII. The experimental form is colored in blue.

For Target XXXI, energy is not necessarily correlated with density. As can be seen in Figure 4, the experimental forms for Target XXXI are in the relatively low-energy region of the distribution. The experimental forms were ranked as followed: A major > A minor > B. There are only two structures lower in energy than the A major form, though by only about ~1-2 kJ/mol. Additionally, about ~1 kJ/mol separates the A minor from the A major and then a ~1-2 kJ/mol gap between the A minor and B forms.

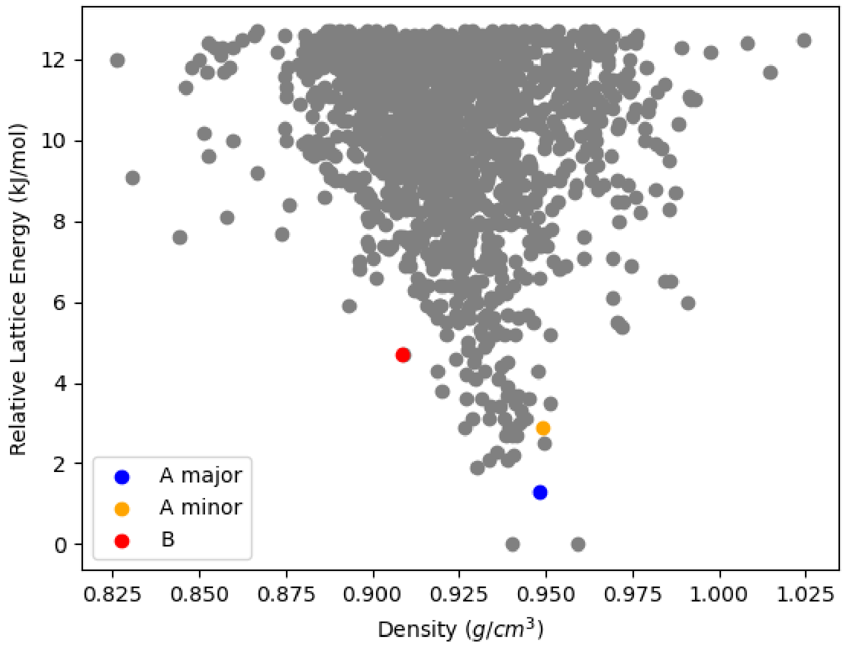

Figure 4: Relative lattice energy obtained using AIMNet as a function of density for the 1500 structures of Target XXXI. Experimental forms are colored.

Summary of Computational Resources

The following computational resources were utilized:

- PSC Bridges2 supercomputer (AMD EPYC 7742 CPUs): Generation of crystal structures and Crystal structure optimizations
- Nvidia RTX 3090 GPUs: AIMNet model training
- SDSC Expanse (AMD EPYC 7742 CPUs), TACC Frontera (Intel Xeon Platinum 8280 CPUs), and Open Science Grid (various CPUs, Nvidia GTX 1080, GTX 1080ti and Tesla V100S GPUs): Crystal structure optimizations

The total computational time spent came from the following steps:

- Generation of 5M crystal structures with Genarris: 6.6 x 10^5 CPU core-hours
- B97M-D4/def2-TZPP DFT energy and force calculations for N-mers using ORCA 5.0 software: 5.5 x 10^4 CPU core-hours
- AIMNet model training: 600 GPU hours
- Optimization of 5 x 10^6 crystal structures with AIMNet: 2.3 x 10^6 CPU core-hours
- DFT crystal structure optimizations: 1.45 x 10^6 CPU core-hours
- PXRD Calculation and Refinement for Target XXIX: 5 CPU Hours

Acknowledgements

O.I acknowledges support from NSF CHE-1802789 and CHE-2041108. We also acknowledge the Extreme Science and Engineering Discovery Environment (XSEDE) award CHE200122, which is supported by NSF grant number ACI-1053575. This research is part of the Frontera computing project at the Texas Advanced Computing Center. Frontera is made possible by the National Science Foundation award OAC-1818253. This research in part was done using resources provided by the Open Science Grid which is supported by the award 1148698, and the U.S. DOE Office of Science.

N.M. acknowledges support from National Science Foundation (NSF) through grant DMR-2131944. This research used resources of Argonne Leadership Computing Facility (ALCF), which is a DOE Office of Science User Facility supported under Contract DE-AC02-06CH11357. We also acknowledge the Extreme Science and Engineering Discovery Environment (XSEDE) award MAT210006 which supported 3M CPU core-hours.

References

(1) Tom, R.; Rose, T.; Bier, I.; O'Brien, H.; Vázquez-Mayagoitia, Á.; Marom, N. Genarris 2.0: A Random Structure Generator for Molecular Crystals. *Comput Phys Commun* **2020**, *250*, 107170. https://doi.org/https://doi.org/10.1016/j.cpc.2020.107170.

(2) O'Boyle, N. M.; Banck, M.; James, C. A.; Morley, C.; Vandermeersch, T.; Hutchison, G. R. Open Babel: An Open Chemical Toolbox. *J Cheminform* **2011**, *3* (1), 33. https://doi.org/10.1186/1758-2946-3-33.

(3) Chan, L.; Hutchison, G. R.; Morris, G. M. BOKEI: Bayesian Optimization Using Knowledge of Correlated Torsions and Expected Improvement for Conformer Generation. *Physical Chemistry Chemical Physics* **2020**, *22* (9), 5211–5219. https://doi.org/10.1039/C9CP06688H.

(4) Brandenburg, J. G.; Bannwarth, C.; Hansen, A.; Grimme, S. B97-3c: A Revised Low-Cost Variant of the B97-D Density Functional Method. *J Chem Phys* **2018**, *148* (6), 064104. https://doi.org/10.1063/1.5012601.

(5) Hawkins, P. C. D.; Skillman, A. G.; Warren, G. L.; Ellingson, B. A.; Stahl, M. T. Conformer Generation with OMEGA: Algorithm and Validation Using High Quality Structures from the Protein Databank and Cambridge Structural Database. *J Chem Inf Model* **2010**, *50* (4), 572–584. https://doi.org/10.1021/ci100031x.

(6) Bier, I.; Marom, N. Machine Learned Model for Solid Form Volume Estimation Based on Packing-Accessible Surface and Molecular Topological Fragments. *J Phys Chem A* **2020**, *124* (49), 10330–10345. https://doi.org/10.1021/acs.jpca.0c06791.

(7) Zubatyuk, R.; Smith, J. S.; Leszczynski, J.; Isayev, O. Accurate and Transferable Multitask Prediction of Chemical Properties with an Atoms-in-Molecules Neural Network. *Sci Adv* **2022**, *5* (8), eaav6490. https://doi.org/10.1126/sciadv.aav6490 .

(8) Bader, R. F. W. Atoms in Molecules. *Acc Chem Res* **1985**, *18* (1), 9–15. https://doi.org/10.1021/ar00109a003.

(9) Neese, F. Software Update: The ORCA Program System—Version 5.0. *WIREs Computational Molecular Science* **2022**, *12* (5), e1606. https://doi.org/10.1002/wcms.1606.

(10) Bannwarth, C.; Ehlert, S.; Grimme, S. GFN2-XTB—An Accurate and Broadly Parametrized Self-Consistent Tight-Binding Quantum Chemical Method with Multipole Electrostatics and Density-Dependent Dispersion Contributions. *J Chem Theory Comput* **2019**, *15* (3), 1652–1671. https://doi.org/10.1021/acs.jctc.8b01176.

(11) Ong, S. P.; Richards, W. D.; Jain, A.; Hautier, G.; Kocher, M.; Cholia, S.; Gunter, D.; Chevrier, V. L.; Persson, K. A.; Ceder, G. Python Materials Genomics (Pymatgen): A Robust, Open-Source Python Library for Materials Analysis. *Comput Mater Sci* **2013**, *68*, 314–319. https://doi.org/10.1016/j.commatsci.2012.10.028.

(12) Blum, V.; Gehrke, R.; Hanke, F.; Havu, P.; Havu, V.; Ren, X.; Reuter, K.; Scheffler, M. Ab Initio Molecular Simulations with Numeric Atom-Centered Orbitals. *Comput Phys Commun* **2009**, *180* (11), 2175–2196. https://doi.org/10.1016/j.cpc.2009.06.022.

(13) Macrae, C. F.; Sovago, I.; Cottrell, S. J.; Galek, P. T. A.; McCabe, P.; Pidcock, E.; Platings, M.; Shields, G. P.; Stevens, J. S.; Towler, M.; Wood, P. A. Mercury 4.0: From Visualization to Analysis, Design and Prediction. *J Appl Crystallogr* **2020**, *53* (1), 226–235. https://doi.org/10.1107/S1600576719014092.

11. Group 17

Supplementary Information for The 7th CSP Blind Test, Phase 1

Hiroyuki Matsui and Kotaro Shinohara

Research Center for Organic Electronics (ROEL), Yamagata University, Jonan 4-3-16, Yonezawa, Yamagata, 992-8510 Japan

h-matsui@yz.yamagata-u.ac.jp

An initial conformation (XXVII-AB) was prepared by substituting two iodine atoms to the known conformation of 6,13-bis(triisopropylsilylethynyl)pentacene (TIPS-pentacene)[1]. Another conformation (XXVII-AA) was prepared by rotating the TIPS group by 180°. These two conformations were used as initial conditions. 10000 initial crystal structures were generated by randomly placing two molecules of the same conformation in a randomly generated cell. No space group was assumed. The 10000 crystal structures were optimised using GULP software[2] with the Dreiding force field. The charge of each atom was calculated by electrostatic potential (ESP) fitting with the HLYGAt method[3] at the B3LYP/6-311G** level using Gaussian 16[4]. No vibrational effect was taken into account at this stage. Clustering was performed based on cell energy, cell volume, and powder pattern similarity. Finally, the 1500 structures with the lowest energy were submitted in the first submission. All had the XXVII-AB conformation.

[1] The Cambridge Crystallographic Database (CSD), refcode: VOQBIM

[2] General Utility Lattice Program (GULP), Version 5.2, https://gulp.curtin.edu.au/

[3] H. Hu, Z. Lu and W. Yang, "Fitting Molecular Electrostatic Potentials from Quantum Mechanical Calculations," *J. Chem. Theory and Comput.* 3 (2007) 1004-13.

[4] Gaussian 16, Revision A.03, M. J. Frisch *et al.*, Gaussian, Inc., Wallingford CT, 2016.

12. Group 18

Authors & Affiliations

Zeinab M. Saeed,[1] Bhausaheb Dhokale,[1][2] Tamador Alkhidir,[1] Mubarak Almehairbi,[1] and Sharmarke Mohamed[1][3]

[1] Green Chemistry & Materials Modelling Laboratory, Khalifa University of Science and Technology, P.O. Box 127788, Abu Dhabi (UAE)

[2] Department of Chemistry, University of Wyoming, Laramie, Wyoming 82071 (USA)

[3] Center for Catalysis and Separations, Khalifa University of Science and Technology, P.O. Box 127788, Abu Dhabi (UAE)

1. Detailed Methodologies for CSP (Submission 1)

1.1 System XXIX

A search of the putative conformations of XXIX were initiated in Spartan (*Wavefunction Inc.* , 2019) by performing a molecular mechanics conformer distribution search with the aid of the MMFF force field. Only the most stable conformers with energies equal to or less than 40 kJ mol^{-1} relative to the global minimum conformer were kept for further analysis. A restriction of keeping only the most stable 500 conformers within this energy range was applied. During the search only two conformers were returned. These conformers were used as input for a gas phase optimization at the B97D/6-31G(d,p) level of theory using GAUSSIAN09 (Frisch *et al.*, 2009). The gas-phase optimized geometry for each of the two conformational minima generated by GAUSSIAN09 were used as input for separate rigid-body crystal structure prediction searches using the CrystalPredictor II code (Habgood *et al.*, 2015). For each search, a total of 500,000 energy minimizations were requested. In each case, a Z′=1 search was requested. During the crystal structure prediction search, the electrostatic contributions towards the intermolecular forces were modelled using atomic charges derived from the *ab initio* wavefunction. The dispersion-repulsion contributions towards the lattice energy were modelled using a Buckingham exp-6 function using Williams' FIT potential parameters. For more details on the FIT potential parameters used, the reader is referred to our recent work using this parameter set (Shruti *et al.*, 2022). Final lattice energies were estimated using a distributed-multipole model using DMACRYS (Price *et al.*, 2010). The dispersion-repulsion contributions were estimated using the same FIT potential parameter set used in the initial CSP search but the electrostatic contributions were estimated using a more accurate distributed multipole model (up to rank 4) derived from the B97D/6-31G(d,p) wave function. The DMACRYS lattice energy optimization was performed using a rigid-body approximation. For specific details on the clustering methods used at each stage, the reader is referred to our previous work (Shunnar *et al.*, 2020).

1.2 System XXX

The molecular structures of cannabinol (CBN) and tetramethylpyrazine (TMP) were extracted from the crystal structures with CSD refcodes CANNOL and BUNFUN respectively. The gas phase conformational minima for CBN and TMP were calculated at the B97D/6-31G(d,p) level of theory using GAUSSIAN09. The gas phase conformational minima were then used as input for a rigid-body CSP search using CrystalPredictor II. A total of 6 searches were performed. Initially, 2 searches were performed, for the most stable crystal structures of TMP and CBN respectively ($Z'=1$). Due to challenges in securing sufficient computing resources, only 10,000 minimizations were requested for each search for the crystal structures of TMB and CBN. The intention behind these limited searches was to find suitable low-energy packings for TMP and CBN in order to understand packing preferences. Then, 4 separate rigid-body searches for cocrystals of varying stoichiometries were initiated using a total of 500,000 minimizations for each search. The following stoichiometries of CBN:TMP were searched for putative crystal structures: 1:1, 1:2 and 2:1. The methodology clustering at each stage, estimate of the lattice energies for the predicted crystal structures as well as the methodology for the final lattice energy estimates using a distributed-multipole model were identical to that used for XXIX.

1.3 System XXXI

The molecular structure for XXXI was sketched in Spartan and a search for putative conformations was initiated by performing a molecular mechanics conformer distribution search with the aid of the MMFF force field. Only the most stable conformers with energies equal to or less than 40 kJ mol^{-1} relative to the global minimum conformer were kept for further analysis. A total of 10 conformers were returned within a relative energy of 12.55 kJ mol^{-1} relative to the global minimum conformer. The two most stable conformers were passed to GAUSSIAN09 for geometry optimization at B97D/6-31G(d,p) level of theory. However, only one conformer converged during the optimization and this single conformer was progressed to the next stage for a rigid-body crystal structure prediction search using the CrystalPredictor II code. A total of 500,000 minimizations were requested in the CrystalPredictor search. The methodology for clustering at each stage, estimate of the lattice energies for the predicted crystal structures as well as the methodology for the final lattice energy estimates using a distributed-multipole model were identical to that used for XXIX.

1.4 System XXXII

The molecular structure for XXXII was sketched in Spartan and a search for putative conformations was initiated using a molecular mechanics conformer distribution search with the aid of the MMFF force field. Only the most stable conformers with energies equal to or less than 40 kJ mol^{-1} relative to the global minimum conformer were kept for further analysis. The most stable conformer was used as input for a gas phase optimization at the B97D/6-31G(d,p) level of theory using GAUSSIAN09. The gas-phase optimized geometry for XXXII was used was used as input to a rigid-body crystal structure prediction search using the CrystalPredictor II code. A total of 500,000 minimizations were requested in the CrystalPredictor search. The methodology for clustering at each stage, estimate of the lattice energies for the predicted crystal structures as well as the methodology for the final lattice energy estimates using a distributed-multipole model were identical to that used for XXIX.

References

Frisch, M. J., Trucks, G. W., Schlegel, H. B., Scuseria, G. E., Robb, M. A., Cheeseman, J. R., Scalmani, G., Barone, V., Mennucci, B., Petersson, G. A., Nakatsuji, H., Caricato, M., Li, X., Hratchian, H. P., Izmaylov, A. F., Bloino, J., Zheng, G., Sonnenberg, J. L., Hada, M., Ehara, M., Toyota, K., Fukuda, R., Hasegawa, J., Ishida, M., Nakajima, T., Honda, Y., Kitao, O., Nakai, H., Vreven, T., Montgomery Jr., J. A., Peralta, J. E., Ogliaro, F., Bearpark, M. J., Heyd, J., Brothers, E. N., Kudin, K. N., Staroverov, V. N., Kobayashi, R., Normand, J., Raghavachari, K., Rendell, A. P., Burant, J. C., Iyengar, S. S., Tomasi, J., Cossi, M., Rega, N., Millam, N. J., Klene, M., Knox, J. E., Cross, J. B., Bakken, V., Adamo, C., Jaramillo, J., Gomperts, R., Stratmann, R. E., Yazyev, O., Austin, A. J., Cammi, R., Pomelli, C., Ochterski, J. W., Martin, R. L., Morokuma, K., Zakrzewski, V. G., Voth, G. A., Salvador, P., Dannenberg, J. J., Dapprich, S., Daniels, A. D., Farkas, Ö., Foresman, J. B., Ortiz, J. V., Cioslowski, J. & Fox, D. J. (2009). Version Revision D.01.

Habgood, M., Sugden, I. J., Kazantsev, A. V., Adjiman, C. S. & Pantelides, C. C. (2015). *J. Chem. Theory Comput.* **11**, 1957-1969.

Price, S. L., Leslie, M., Welch, G. W. A., Habgood, M., Price, L. S., Karamertzanis, P. G. & Day, G. M. (2010). *PCCP* **12**, 8478-8490.

Shruti, I., Almehairbi, M., Saeed, Z. M., Alkhidir, T., Ali, W. A., Vishwakarma, R., Mohamed, S. & Chopra, D. (2022). *Cryst. Growth Des.* **22**, 5861-5871.

Shunnar, A. F., Dhokale, B., Karothu, D. P., Bowskill, D. H., Sugden, I. J., Hernandez, H. H., Naumov, P. & Mohamed, S. (2020). *Chemistry – A European Journal* **26**, 4752-4765.

Wavefunction Inc., Spartan’18, Version 1.3.0, 18401 Von Karman Ave., Suite 370, Irvine CA 92612, 2019.

13. Group 19

Supporting information statement for 7th Crystal Structure Prediction Challenge

Group: OpenEye Scientific Software
Contributors: Hari Muddana, Varsha Jain, Tom Darden, Geoff Skillman
Corresponding author: hmuddana@eyesopen.com

1. Methodology: Round1

Our approach for predicting and ranking crystal structures of a molecule has four stages:

1. *Conformer generation:* We generate low-energy 3D conformations of the molecule
2. *Packing generation:* The low-energy conformers are packed in top space groups and optimized using a multipole force-field, Intermolecular Energy Force-Field (IEFF) [1].
3. *QM optimization:* The low-energy IEFF packings are then optimized and rescored using quantum-mechanical energy models, to give 0K predictions.

The summary of different stages for various challenges are given in Table 1, and each of the above stages is described in more detail below.

**Challenge**	**Conformer generation**			**Packing generation**	**QM Optimization**
	# confs	*Geom. Opt.*	*Single point energy*	*#space groups (top)*	
XXVII	15	HF-3c	B3LYP-D3MBJ/6-31G*	20	No
XXIX	10	HF-3c	B3LYP-D3MBJ/6-31G*	20	HF-3c
XXX	934 (dimers) 2338 (trimers)	HF-3c	HF-3c	10	HF-3c
XXXI	748	HF-3c	B3LYP-D3MBJ/6-31G*	20	HF-3c
XXXII	982	HF-3c	B3LYP-D3MBJ/6-31G*	20	No
XXXIII	544	HF-3c	B3LYP-D3MBJ/6-31G*	20	No

Table 1. Summary of conformer generation, packing generation, and QM optimization for various challenges.

Conformer generation: 3D conformers of the molecule were generated using OE's Omega toolkit using custom torsion rules [2-3]. The torsion rules were generated based on the torsional energy surfaces at B3LYP-D3MBJ/6-31G* level of theory. Low-energy conformers are filtered for packing generation.

For challenge molecule XXVII, we substituted Si with Carbon, and Iodine with Chlorine, due to lack of force-field parameters for Si and Iodine. This analogue of the original molecule was used for crystal structure prediction.

For challenge XXX, dimer and trimer geometries were generated using IEFF force-field [1]. First, we packed the monomers separately into various crystal structures, and those conformers that yielded low-energy crystal packings were chosen for dimer generation. Low-energy dimers were generated by scanning all relative translations and rotations.

Those dimers that yielded low-energy crystal packings were chosen for trimer generation, following a similar protocol as that of dimers.

For challenge XXXIII, low-energy salt configurations were generated using IEFF force-field [1]. First, we generated low-energy 3D conformations of the molecule and scanned the relative placement of the ion using IEFF. Low-energy dimer conformations were selected for packing generation.

Packing generation: Crystal structures for each conformer in the most frequently observed space groups were generated by randomly varying the conformer orientation and unit cell parameters within certain bounds [1]. First, the conformer is assigned a random orientation and placed in the unit cell. The conformer was then projected along each axis (a,b,c) of the unit cell. The length of this projection was used to randomly pick the length of each axis. Next, we randomly assigned unit cell angles based on volumetric restrictions. Packings with packing fraction between 0.55 and 0.85, and no atom-atom collisions are selected for further optimization using a multipole force-field, IEFF.

For challenge XXIX, packings that have a powder spectrum that is most similar to the provided powder spectrum were submitted. Similarity of powder spectrum is calculated as the cross-correlation metric between the powder spectrums.

QM optimization of crystal structures: We optimized the crystal structures using an approach referred to here as “dimer expansion”. In this approach, the total energy and gradients of the system are calculated as a sum of all dimers within the crystal. The fractional coordinates of the molecule and unit cell parameters are optimized separately in three iterations. First, we optimize the geometry of the molecule while keeping the unit cell parameters fixed, and then perform unit cell optimization while keeping the geometry of the molecule fixed. Finally, we perform another iteration of optimizing the geometry of the molecule. Conformer geometries were optimized using GeomeTRIC [4], and the unit cell parameters were optimized using NLOpt [5]. The gradients for both conformer geometry and unit cell parameters are computed using HF-3c method[6]. Specifically, we build a cluster of the crystal out to 12A and decompose that into dimers, accounting for symmetrically related pairs. We compute the energy and gradients of each dimer and sum them up to get the crystal energy and gradients, respectively. Optimization steps are continued until the large component of the gradient is less than 0.0001 Hartree/Angstrom, or a maximum number of steps has reached. Psi4 1.3.2 was used for all dimer energy and gradient calculations [7].

References

[1] Elking, Dennis M., Laszlo Fusti-Molnar, and Anthony Nichols. "Crystal structure prediction of rigid molecules." *Acta Crystallographica Section B: Structural Science, Crystal Engineering and Materials* 72.4 (2016): 488-501.

[2] Hawkins, Paul CD, et al. "Conformer generation with OMEGA: algorithm and validation using high quality structures from the Protein Databank and Cambridge Structural Database." *Journal of chemical information and modeling* 50.4 (2010): 572-584.
[3] Hawkins, Paul CD, and Anthony Nicholls. "Conformer generation with OMEGA: learning from the data set and the analysis of failures." *Journal of chemical information and modeling* 52.11 (2012): 2919-2936.
[4] Wang, Lee-Ping, and Chenchen Song. "Geometry optimization made simple with translation and rotation coordinates." *The Journal of chemical physics* 144.21 (2016): 214108.
[5] Johnson, Steven G. "The NLopt nonlinear-optimization package." (2014).
[6] Sure, Rebecca, and Stefan Grimme. "Corrected small basis set Hartree-Fock method for large systems." *Journal of computational chemistry* 34.19 (2013): 1672-1685.
[7] Parrish, Robert M., et al. "Psi4 1.1: An open-source electronic structure program emphasizing automation, advanced libraries, and interoperability." *Journal of chemical theory and computation* 13.7 (2017): 3185-3197.

14. Group 20

Supporting information Group 20

Summary
We were able to confirm the first successful prediction of configurational disorder and its thermodynamic implications for target XXX form A. After detailed analysis of the experimental data made available after the deadline, we were able to identify additional matches for XXX "Group E" as our submitted rank 1 and for the room-temperature form of XXXII form B as our submitted structure 159. All 17 experimentally observed local minima were trivially generated, and 15 of these were both within the limits on the number of structures set by the organisers and the standard energy cut-offs of our software, the two exceptions being one minor disorder component for XXXII Form A and XXXI Form C with 80 Å^3 void space per *Z*. In an industrial context, where it is important to implement a cost-effective compromise to locate all thermodynamically *relevant* local minima, our software performed as intended by correctly generating—blind—all thermodynamically relevant experimental local minima.

Method development was ongoing in parallel with the actual blind test calculations, *e.g.*, to cope with the Cu-containing compound and to address the problems caused by the high molecular symmetry of the Si compound, and as a result different compounds may have been run with different features enabled at different stages.

The basis for the structure generations is parallel-tempering Monte Carlo with a tailor-made force field (TMFF) parameterised against quantum mechanical (QM) reference data as described by Neumann (2008). For some compounds, the TMFF was extended to use a multipole description for the atomic charges as opposed to a monopole description as used in earlier versions. Tens of thousands of structures are generally generated within the energy window relevant for the structure generation with the TMFF, but only 500 or 1,500 structures could be submitted. The reduction of the number of structures is accomplished by our *reranking* step, which uses a cascade of increasingly more accurate QM calculations combined with similarity measures to estimate which of the tens of thousands of structures should be considered for final optimisation with QM. For some blind test compounds, this reranking step was augmented with Machine Learning (ML) to predict which of the TMFF structures after energy minimisation with QM would end up near the bottom of the energy window (or rather, the logic is reversed: the ML is used to weed out structures that do not need to be considered for energy optimisation because they are predicted by the ML model to become high-energy structures with QM despite their favourable TMFF energy).

The calculations were performed with our in-house developed software *GRACE*, which calls *VASP* (Kresse & Furthmüller, 1996, Kresse & Hafner, 1993, Kresse & Joubert, 1999), *Turbomole* (Ahlrichs *et al*., 1989, Furche *et al*., 2014) and *FHI-aims* (Blum *et al*., 2009) for QM calculations.

Figure 1 shows the overlays of all experimental structures and their blind[1] predictions. For XXXII Form B RT, we corrected the experimental structure that was made available by the organisers after the submission deadline, an overlay of the corrected experimental structure as determined by us from Rietveld refinement and our blind-submitted structure_259.cif is shown in Figure 9. For XXX 1:1 "Group E", no experimental structure was made available by

[1] For the "experimentally-assisted challenge" (XXIX), the simulated powder diffraction pattern made available by the blind test organisers showed that no matches could be found with one or two molecules in the asymmetric unit, triggering a structure generation with three molecules in the asymmetric unit that is not part of our standard protocol.

the organisers, but an overlay of the experimental structure as determined by us from Rietveld refinement overlaid with our submitted rank 1 is shown in Figure 7. XXXI Form C was outside the energy window for submission and we include it in a separate Figure (Figure 2) based on our post analysis, likewise for XXXII Form A minor occupation. For XXIX, a simulated powder diffraction pattern was made available, allowing us to perform a Rietveld refinement and thereby adjusting our calculated 0 K unit-cell parameters to the correct room-temperature parameters; Figure 1 shows the experimental single-crystal structure *versus* rank 1 from our prediction, Figure 5 shows the experimental single-crystal structure *versus* rank 1 after our automated powder diffraction matching and Rietveld refinement procedure.

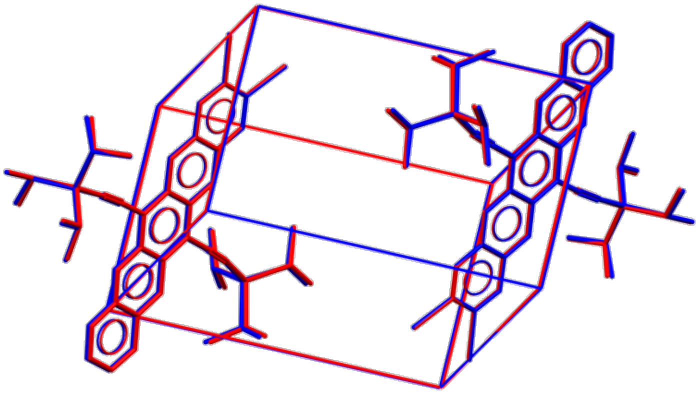

XXVII

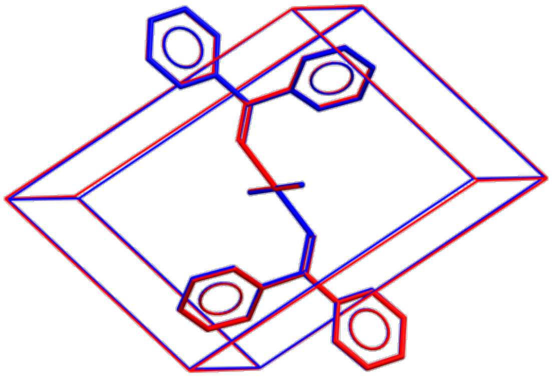

XXVIII

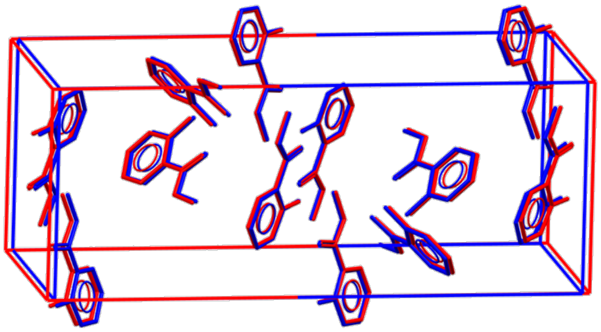

XXIX

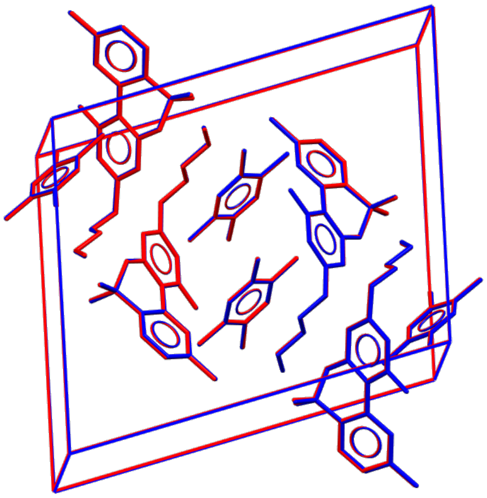

XXX 1:1

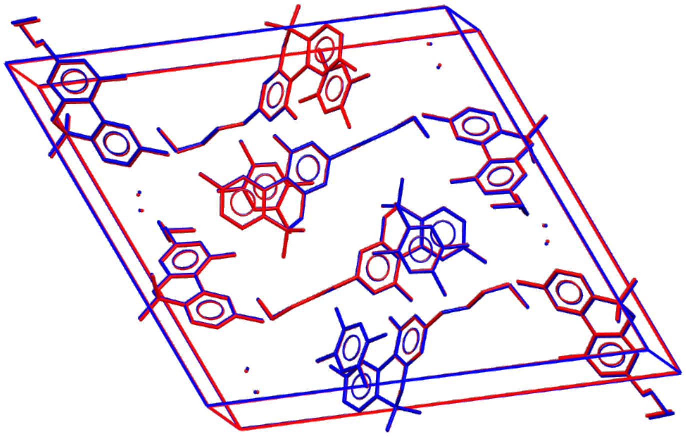

XXX 2:1 (disordered)

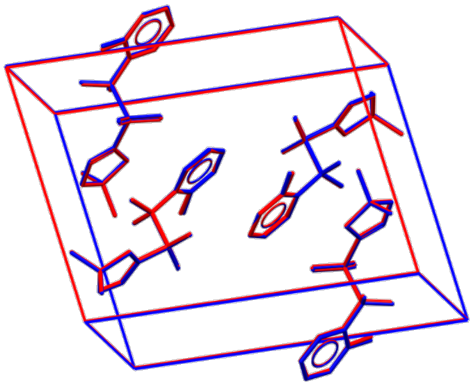

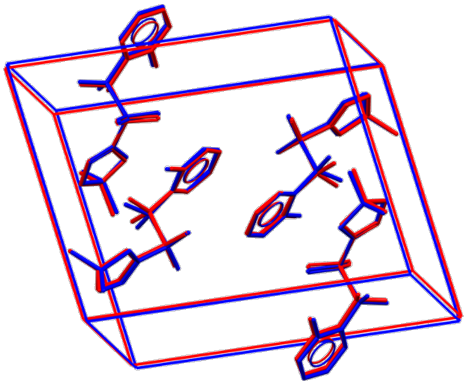

XXXI Form A (major occupancy / minor occupancy)

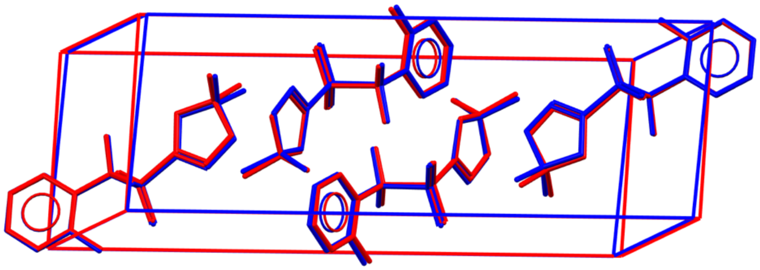

XXXI Form B

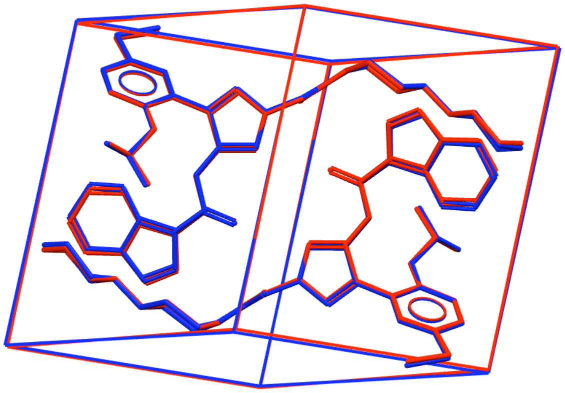

XXXII Form A major occupancy

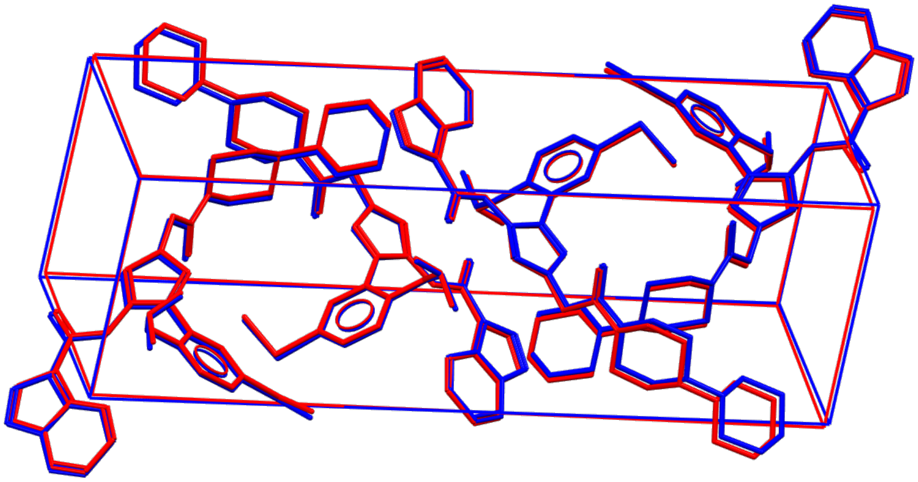

XXXII Form B low temperature

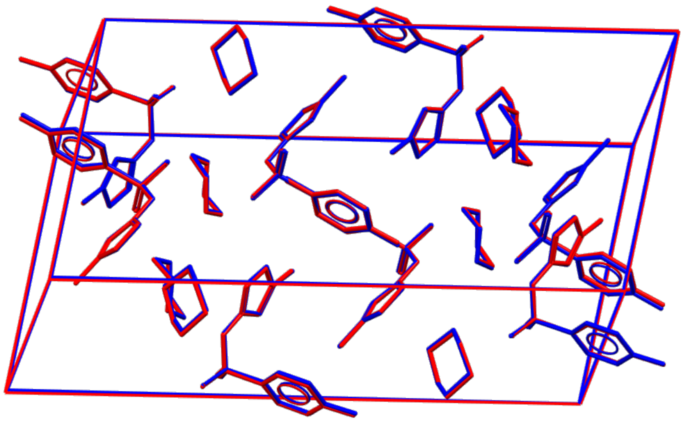

XXXIII Form A

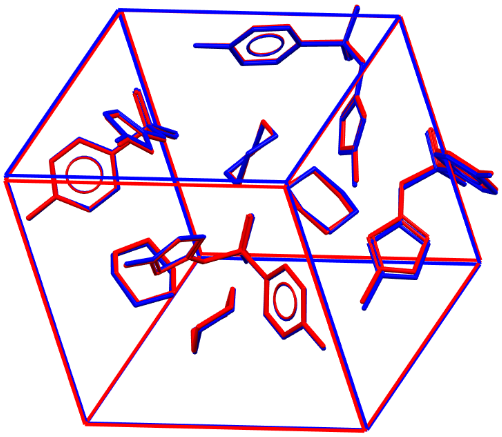

XXXIII Form B

Figure 1. Overlays of the experimental structures (red) and their blind predictions (blue); see Figure 7 and Figure 9 for overlays of blind predictions of additional experimental phases identified by our group but not a formal part of this blind test. Note that the disorder in XXX 2:1 was correctly predicted and was part of our submitted structure. Hydrogen atoms omitted for clarity.

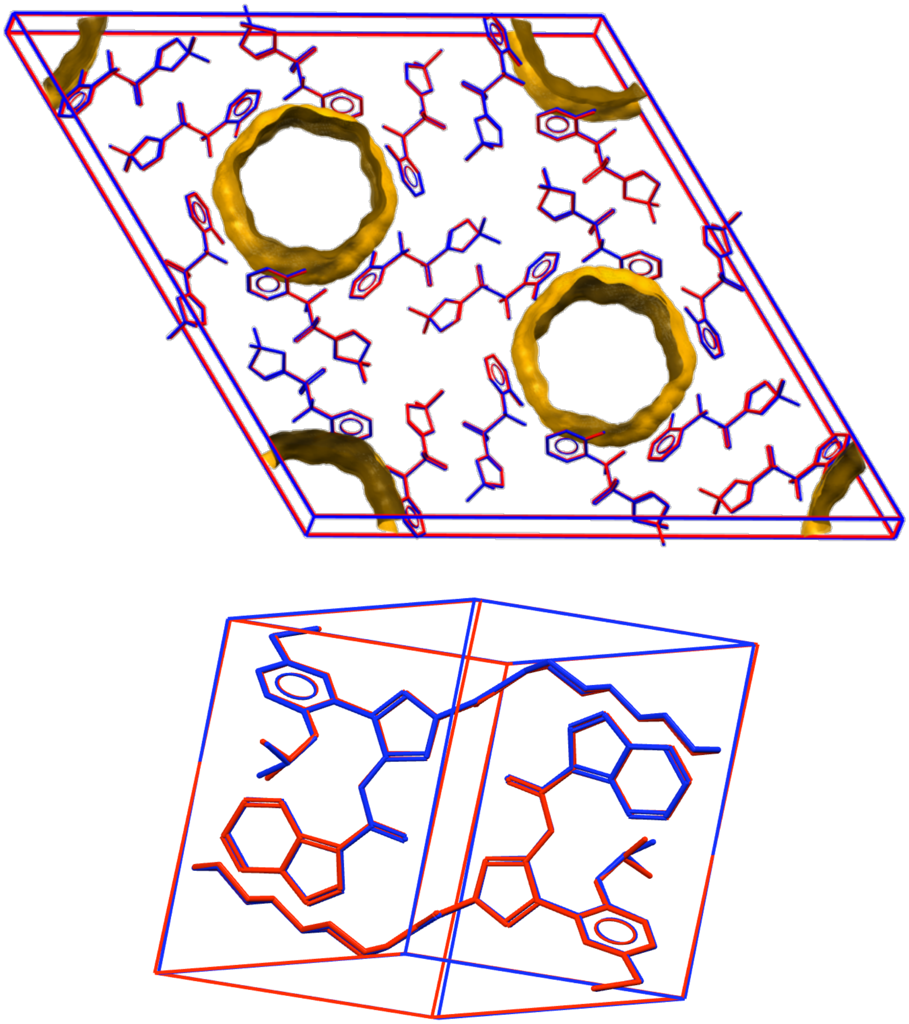

Figure 2. Overlays of the two experimental structures of XXXI Form C (with large voids) and XXXII Form A minor occupancy (red) and their structures as located in our post analysis (blue). Hydrogen atoms omitted for clarity.

XXVII. During the structure generation, completeness is assessed by, among other criteria, how many structures have thus far been generated only once; the more such structures, the more phase space there is left to explore. For the Si compound, the high molecular symmetry caused the structure matching algorithm to produce many false negatives, *i.e.*, to label many structures as unique whereas in fact they corresponded to structures that had already been found, poisoning the single-hit statistics and leading the structure generation to erroneously believe that the phase space had not been adequately explored yet and preventing the convergence criteria from being met. Improvements to the matching algorithm were made. Where required, missing dispersion-correction parameters for silicon were derived.

XXVIII. For the Cu compound, force field terms had to be added to describe a square-planar coordination. The *trans* configuration was used as provided. The quantum-mechanical calculations for the reference data had to be made spin-aware. Because support for square-planar Cu had to be added to our software from scratch, after each step in the prediction procedure, the computational results were compared against the published experimental

structure to ensure that it was still present among the top 1,500 structures. This was the case throughout the entire procedure and the Cu compound can be predicted with the latest version of our software as-is. Part of the calculations were run on the Azure cloud.

XXIX. For the experimentally-assisted challenge, the powder pattern was digitised to allow automated comparison against the predicted structures with our software module “Structure Solution Factory”. We started out with our standard crystal structure prediction protocol, which generates structures with one and two molecules in the asymmetric unit in a selected set of space groups covering about 98% of all molecular crystal structures in the CSD. In the information supplied by the blind test organisers, an inset was shown with two very small peaks at around 7.0° and 9.5° 2θ. It was furthermore stated that the powder diffraction pattern was simulated, so it was clear that these two small peaks were not impurities but had to be explained by the submitted structure. After establishing that none of the generated structures explained the small peaks at very low 2θ, it was decided to expand the search space to include all 230 space groups and to include Z'=3. The Z'=3 structure generation produced a satisfactory match in $P2_1/c$.

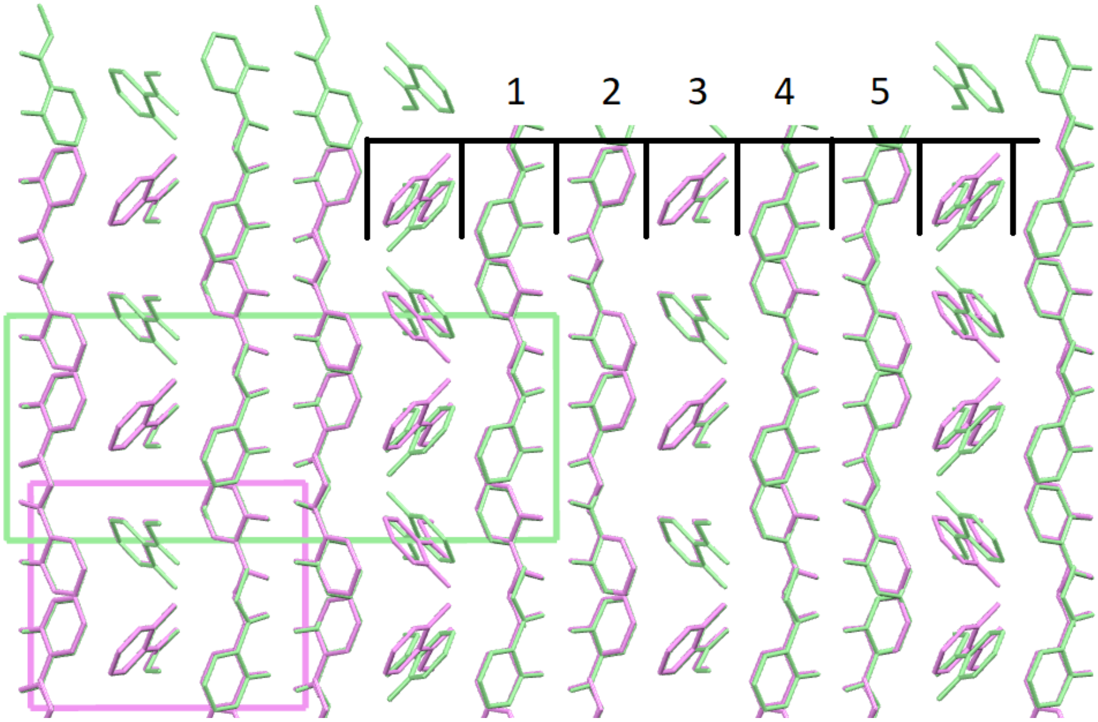

Figure 3. Overlay of our submission rank 1 (light green, $P2_1/c$, Z'=3) and rank 2 (violet, *Pc*, Z'=3), showing the five layers they have in common. Hydrogen atoms omitted for clarity.

The Z'=3 structure generation also produced a match in *Pc* that has five layers in common with the $P2_1/c$ structure and that has a calculated lattice energy only 0.02 kcal/mol higher than the $P2_1/c$ structure, strongly suggesting the possibility of polytypism (Figure 3), but only the $P2_1/c$ structure explained the two peaks at 7.0° and 9.5° 2θ (Figure 4) and it was therefore this structure that we submitted as our solution. Figure 5 shows the overlay of the experimental single-crystal structure as made available by the organisers after the submission deadline and the structure resulting from the automated Rietveld refinement with our Structure Solution Factory module.

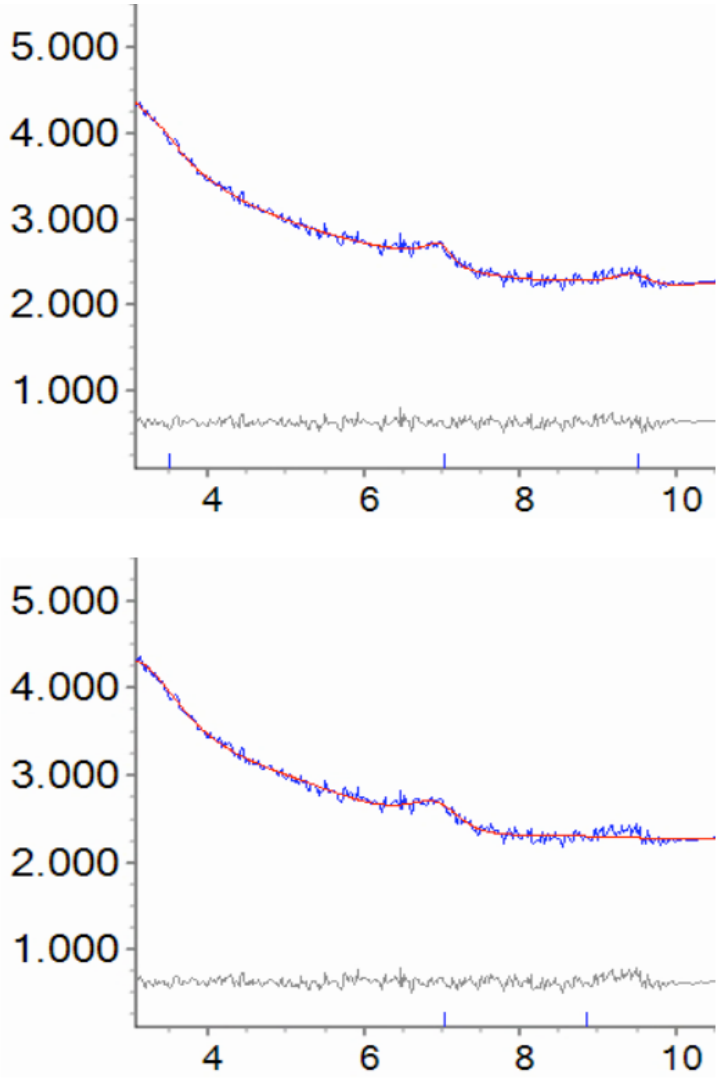

Figure 4. Rietveld refinement fit to the peaks at 7.0° and 9.5° 2θ for the $P2_1/c$, Z'=3 structure (top) and the Pc, Z'=3 structure (bottom). Experimental data in blue, calculated pattern in red, difference curve in black. Tickmarks are shown in blue.

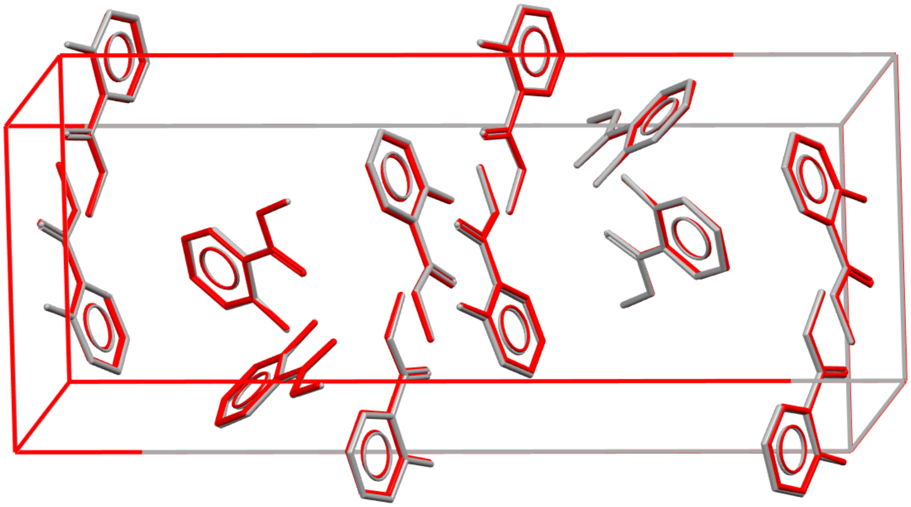

Figure 5. Single-crystal structure of target XXIX (red) overlaid with the structure from our automated Rietveld refinement ("Structure Solution Factory") (grey). Hydrogen atoms omitted for clarity.

XXX. For the stoichiometry challenge, the crystal structures of pure tetramethylpyrazine and pure cannabinol were taken from CSD entries MPYRAZ02 and CANNOL, respectively. Structure generation with multiple molecules, even if chemically different compounds, and molecular flexibility have been implemented in our software *GRACE* from its inception 20 years ago so the prediction of the 1:1, 1:2, and 2:1 co-crystals could be run with the standard version of our software.

Target XXX was special in that not only the *generated* but also the *ranked* (by free energy) candidates had to be submitted at this stage of the blind test, prompting us to calculate the temperature-dependent free energies of the most promising candidates using our TRHu(ST) method (Firaha *et al.*, 2023). A routine scan for similarity among these thermodynamically relevant candidates suggested that several of these could be considered different configurations of the same disordered crystal structure. These sets of structures were merged by transforming the crystal structures so that most of the atoms could be overlaid, and, taking the lowest-energy structure as basis, the alternative configurations were manually added to the .cif file of the basis structure. The proper calculation of the disorder contribution to the free energy, including all statistical thermodynamics formulae, are described in detail elsewhere (Woollam *et al.*, 2018). This procedure resulted in five crystal structures being submitted as disordered, each with two disorder configurations. Upon further energy minimizations, two disorder configurations converged to the same structure, which was overlooked during the analysis and the duplicate configuration led to a spurious free energy contribution of $-RT\ln 2$ = -0.41 kcal/mol, which in turn meant that the affected 1:1 structure ended up as rank 1. When this error is corrected, ranks 1 and 2 (1:1 stoichiometry only) swap places.

Among the experimental data released for target XXX was a powder diffraction pattern for a phase referred to as "Group E", which had not been indexed and for which no crystal structure had been determined. Again, the fact that for this target we had ranked the predicted structures by free energy played an important role, because instead of trying to match the Group E pattern against all 1,500 generated structures, which would require the use of a computer program, the free-energy ranking acts as an initial screen and the

experimental data ideally only has to be matched against one of the top ten or so structures, a matching procedure that can be done manually. Our submitted rank 1 structure, a 1:1 stoichiometry structure, immediately provided an excellent visual match. The blind test organisers were contacted with a request for the pattern in electronic form, the Rietveld refinement is shown in Figure 6.

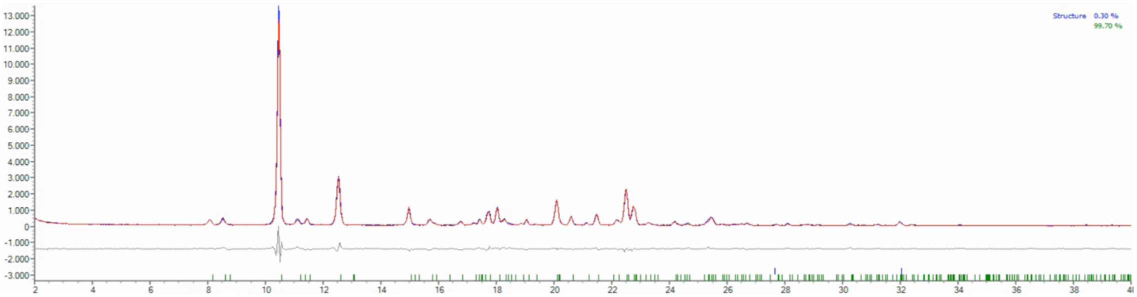

Figure 6. Rietveld refinement of our rank 1 for target XXX against the powder diffraction pattern for "Group E". Experimental data in blue, calculated pattern in red, difference curve in black.

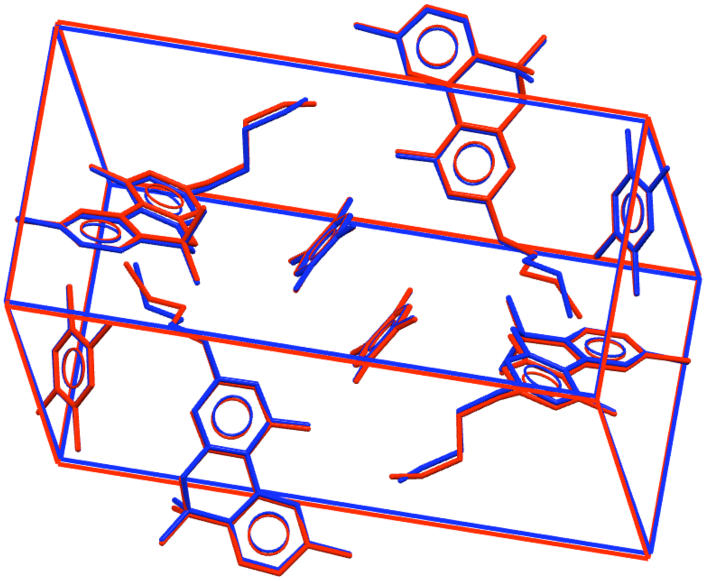

Figure 7. Overlay of the structure of XXX "group E" from Rietveld refinement (red) and our submitted rank 1 (blue). Hydrogen atoms omitted for clarity.

Looking at the final results per stoichiometry, our final prediction is then that 1. No co-crystal with 1:2 stoichiometry will be observed, 2. A co-crystal with stoichiometry 1:1 will be observed corresponding to rank 5 in our combined list, 3. A co-crystal with stoichiometry 2:1 will be observed corresponding to rank 2 in the combined list. All these predictions turned out to be correct, and our predicted rank 2 for the 1:1 stoichiometry also turned out to have been observed (Table 1).

Table 1. Summary of results for the stoichiometry challenge.

Rank as submitted combined	Rank as corrected combined	Stoichiometry	Predicted rank as corrected per stoichiometry	Experimental rank per stoichiometry
1	5	1:1	2, *i.e.* NO or NS	NS
2	1	2:1	1	1
3	2	2:1	2, *i.e.* NO or NS	NO
4	3	2:1	3, *i.e.* NO or NS	NO
5	4	1:1	1	1

NO = Not Observed, NS = Not Stable.

XXXI. The agrochemical compound was run on the AWS cloud through *Rescale*.

In experiment, a Form C had been observed with a very low density, which is due to an exceptionally large void space of 80 Å^3/Z. The structure was generated in our structure generation as rank 7,153 among all Z'=1 structures, but because of the large void space the lattice energy is calculated to be unfavourable and this structure did not make it into our final list of 1,500 structures.

XXXII. Large, flexible molecules have been implemented in our software *GRACE* from its inception 20 years ago, but after it was noted that the structure generation with two molecules in the asymmetric unit appeared to be exceptionally slow, more than a month was spent on experimenting to speed up the structure generation. In the end, the mechanism to update the temperatures for the parallel tempering was identified as being suboptimal and subsequently overhauled. The reported CPU time includes all the time spent on the compound, including the experimenting and validation, and with the latest version of our software the structure generation would be about two orders of magnitude faster for Z'=2.

Form A turned out to be disordered. The major occupancy was present in our list of submitted structures, but the minor occupancy was not. A structure generation in $P1$, Z'=2 with a more lenient energy window quickly located the structure, showing that it was approximately 1 kcal/mol above our standard energy cut-off.

For XXXII Form B, a low-temperature (LT) and a room-temperature (RT) phase were provided. The LT phase had been determined from single-crystal data, the RT phase from powder diffraction data. The structure of the RT phase showed several short non-bonded C····C contacts and distorted upon energy minimisation with DFT. An overlay of the two experimental structures showed that the morpholine ring in the RT structure should be rotated over 180°. An excellent Rietveld refinement (Figure 8) and an excellent reproduction with DFT (Figure 9) of the corrected structure confirmed this diagnosis. The LT phase matches our submission structure_71.cif, the corrected RT phase matches our submission structure_259.cif.

Given the ease with which we are able to match experimental powder diffraction data with our predicted structures, even for challenging cases with subtle differences, more matches could have been expected if additional powder diffraction patterns had been made available.

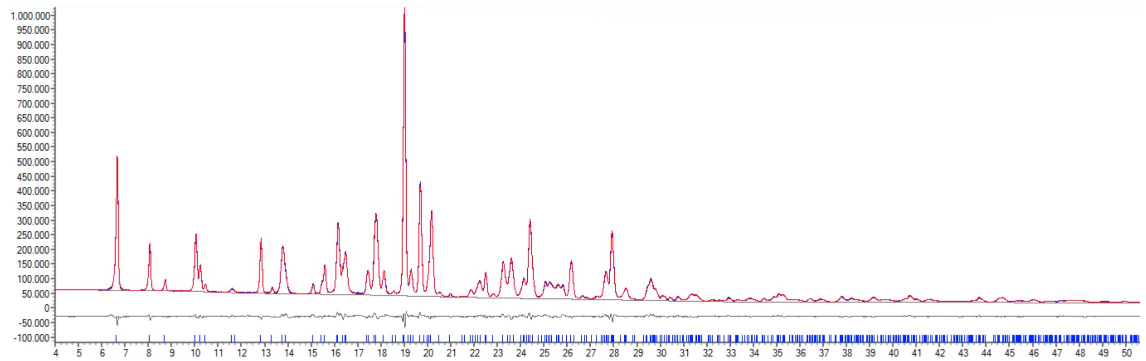

Figure 8. Rietveld refinement of the corrected XXXII form B RT structure. Experimental data in blue, calculated pattern in red, difference curve in black.

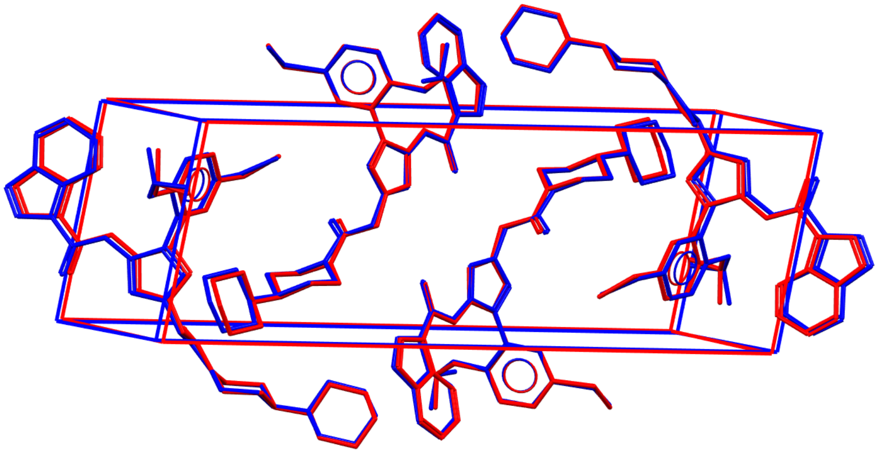

Figure 9. Overlay of the corrected XXXII form B RT structure from Rietveld refinement (red) and the same structure after energy minimisation with DFT (blue). Hydrogen atoms omitted for clarity.

XXXIII. Molecular salts were implemented in our software *GRACE* more than a decade ago and the prediction could be run with the standard version of our software.

In summary, all 17 experimentally observed local minima were trivially generated, and 15 of these were both within the limits on the number of structures set by the organisers and the standard energy cut-offs of our software, the two exceptions being one minor disorder component for XXXII Form A and XXXI Form C with 80 Å^3 void space per *Z*. In an industrial context, where it is important to implement a cost-effective compromise to locate all thermodynamically *relevant* local minima, our software performed as intended by correctly generating—blind—all thermodynamically relevant experimental local minima.

Ahlrichs, R., Bär, M., Häser, M., Horn, H. & Kölmel, C. (1989) *Chem. Phys. Lett*. **162**, 165−253.

Blum, V., Gehrke, R., Hanke, F., Havu, P., Havu, V., Ren, X., Reuter, K. & Scheffler, M. (2009). *Comput. Phys. Commun*. **180**, 2175−2196.

Firaha, D., Liu, Y. M., van de Streek, J., Sasikumar, K., Dietrich, H., Helfferich, J., Aerts, L., Braun, D. E., Broo, A., DiPasquale, A. G., Lee, A. Y., Le Meur, S., Nilsson Lill, S. O., Lunsmann, W. J., Mattei, A., Muglia, P., Putra, O. D., Raoui, M., Reutzel-Edens, S., Rome, S., Sheikh, A. Y., Tkatchenko, A., Woollam, G. R. & Neumann, M. A. (2023). *Nature* **623**, 324-328.

Furche, F., Ahlrichs, R., Hättig, C., Klopper, W., Sierka, M. & Weigend, F. (2014) *WIREs Comput. Mol. Sci*. **4**, 91−100.
Kresse, G. & Furthmüller, J. (1996). *Phys. Rev. B* **54**, 11169−11186.
Kresse, G. & Hafner, J. (1993). *Phys. Rev. B* **47**, 558−561.
Kresse, G. & Joubert, D. (1999). *Phys. Rev. B* **59**, 1758−1775.
Neumann, M. A. (2008). *J. Phys. Chem. B* **112**, 9810−9829.
Woollam, G. R., Neumann, M. A., Wagner, T. & Davey, R. (2018). *Faraday Discuss*. **211**, 209-234.

15. Group 21

Supporting Information for Phase 1

Authors of submission for XXVII, XXIX, and XXXIII:

Shigeaki Obata[1,2], Yasuhiro Ikabata[2], and Hitoshi Goto[1,2]

Authors of submission for XXX:

Shigeaki Obata[1,2] and Hitoshi Goto[1,2]

Authors of submission for XXXI:

Shigeaki Obata[1,2], Yohei Utsumi[3], Yasuhiro Ikabata[2], Koji Okuwaki[3,4], Naofumi Nakayama[1], Kaori Fukuzawa[3,4], Etsuo Yonemochi[3], and Hitoshi Goto[1,2]

[1] CONFLEX Corporation, Shinagawa Center Bldg. 6F, 3-23-17 Takanawa, Minato-ku, Tokyo 108-0074, Japan.

[2] Information and Media Center, Toyohashi University of Technology, 1-1 Hibarigaoka, Tempaku-cho, Toyohashi, Aichi 441-8580, Japan.

[3] School of Pharmacy and Pharmaceutical Sciences, Hoshi University, 2-4-41 Ebara, Shinagawa-ku, Tokyo 142-8501, Japan.

[4] Graduate School of Pharmaceutical Sciences, Osaka University 1-6 Yamadaoka, Suita, Osaka 565-0871, Japan.

E-mail: obata@conflex.co.jp, gotoh@tut.jp, fukuzawa-k@phs.osaka-u.ac.jp

Overview of prediction methods

Structure generation:

The method employs a typical grid-systematic search algorithm and considers four variables required for generating different molecular packings, that is, molecular conformation, orientation, and spatial position, and symmetry [1]. The process in the structure generation consists of (a) determination of initial molecular geometry and conformations, (b) systematic generation of initial crystal structures, (c) optimization of the initial crystal structures, and (d) elimination of identical crystal structures.

(a) Determination of initial molecular geometry and conformations

We create initial geometry from a chemical diagram by ChemDraw 20.0 and the initial one is subjected to geometry optimization and conformation search [2, 3] in gas phase. Some conformers are selected for step (b) according to their energies and conformations. The calculations are performed using MMFF94s potential [4].

(b) Systematic generation of initial crystal structures

Each selected conformer is rotated around the x, y, and z axes. Various initial crystal structures with different molecular packing arrangements are generated using each oriented molecule as asymmetric unit, different spatial positions of the molecule, and common space group symmetries [1].

(c) Optimization of the initial crystal structures

The initial structures are subjected to crystal structure optimization with a fully flexible molecular model under restriction of space group symmetry [1, 5]. In the optimization, we minimize total energy of the crystal per asymmetric unit defined by

$$E_{\mathrm{crystal}} = E_{\mathrm{intra}} + E_{\mathrm{inter}} \qquad (1)$$

where the E_{intra} is the sum of intramolecular energies of molecule(s) in the asymmetric unit and the E_{inter} is the sum of intermolecular interaction energies per asymmetric unit in the crystal. The van der Waals and electrostatic interactions are calculated by the cutoff and Ewald summation [6] techniques, respectively, with a real space cutoff of 20 Å. Both intra- and inter-molecular interactions are estimated by means of MMFF94s potential [4]. The minimizations are carried out using the full diagonal Newton-Raphson method. The convergence thresholds on the root-mean-square deviation of gradients over all parameters and the root-mean-square deviation of displacements of the parameters are set to 1.0×10^{-6} kcal/mol/Å and 1.0×10^{-6} Å, respectively. The optimized crystal structures are confirmed whether they have no imaginary frequencies by performing a normal mode analysis.

(d) Elimination of identical crystal structures

Adequate space groups and lattice constant parameters of the optimized crystal structures are determined using the software PLATON [7] or Materials Studio 2018 [8]. Identical structures among all optimized crystal structures are eliminated using similarity between powder X-ray diffraction (PXRD) patterns [1, 9] and Crystal Packing Similarity Tool in the CSD Python API [10].

Landscape generation:

Table 1 summarizes generation methods of landscapes for each target. We created energy landscapes of XXVII, XXX, XXXI, and XXXIII and assessment value landscape of XXIX. The MMFF94s structures obtained by the structure generations were used in the landscapes. The energies of MMFF94s structures for XXVII and XXXIII were re-determined by QUANTUM ESPRESSO (QE) V.6.7 [11, 12] using GGA-PBE exchange-correlation functional [13], D3 dispersion correction [14], and scalar relativistic ultrasoft pseudopotential [15] (PBE-D3) under periodic boundary condition. CIF2Cell software was used for making input files of QE [16]. The energies of MMFF94s structures for XXXI were re-determined by fragment molecular orbital (FMO) method [17] at the MP2 level of theory (FMO-MP2) [18] with 6-31G† basis set [19-22]. The energy landscape of XXX was based on MMFF94s potential. The assessment value for XXIX was calculated by MMFF94s energy and similarity between calculated and observed PXRD patterns [1].

Table 1. Summary of generation methods of landscapes for each target.

Target	Structure	Landscape
XXVII	MMFF94s	PBE-D3 energy
XXIX	MMFF94s	MMFF94s energy and PXRD pattern similarity
XXX	MMFF94s	MMFF94s energy
XXXI	MMFF94s	FMO-MP2/6-31G† energy
XXXIII	MMFF94s	PBE-D3 energy

Detail of the structure and landscape generations with respect to each target are described in each target’s section.

Target XXVII

Structure generation:

The conformation search provided 2,331 conformers. In the conformational analysis, we found that the lower energy conformers can be classified into three characteristic conformations according to torsion angles of C2, C1, Si1, and C25 and C11, C12, Si2, and C42 (Figure 1). Therefore, we selected three conformers which have the lowest energy among conformers with each characteristic conformation (Figure 1). Each conformer shows the lowest, 3rd lowest, and 7th lowest energies among all conformers, respectively, on MMFF94s potential. After the selection, we calculated torsion potentials of τ_1, τ_2, τ_3, τ_4, τ_5, and τ_6 of each conformer that determine conformation of isopropyl groups (Figures 1 and 2). In Figure 2, the torsion potentials of τ_2, τ_3, τ_5, and τ_6 have two local minima within 0.1 kcal/mol, respectively. Each conformer shows the same potential curves. We further calculated torsion potential of τ_3 with respect to the conformer at around 230 degrees on the torsion potential of τ_2 (Figure 2) and torsion potential of τ_2 with respect to the conformer at around 150 degrees on the torsion potential of τ_3 (Figure 2) using the lowest energy conformer (Figure 3). In Figure 3, there is only one local minimum under low energy area. Therefore, we employed two conformations on the τ_2, τ_3, τ_5, and τ_6, respectively, and the τ_2 and τ_3 or τ_5 and τ_6 were not changed at the same time. Thus, we made 27 (= 3 conformers x 9 conformations) initial

conformers and optimized them in gas phase. Finally, the 21 unique conformers were obtained by the conformational analysis and were used for the generation of initial crystal structures.

In the determination of initial molecular conformation, we also employed the conformation in the crystal structure of 2,3-Dibromo-6,13-bis(triisopropylsilylethynyl)pentacene [23]. Thus, we changed Br to I atoms in the molecule and optimized the modified molecule. The optimized conformer was also used for the generation of initial crystal structures.

In the generation of initial crystal structures, we used 22 unique conforms mentioned above, 10 common space groups (*P*1, *P*1-, $P2_1$, *Cc*, $P2_1/c$, *C*2/*c*, $P2_12_12_1$, $Pna2_1$, *Pbca*, and *Pnma*), and molecular rotational step of 20 degrees. The number of molecules in the asymmetric unit was set to one.

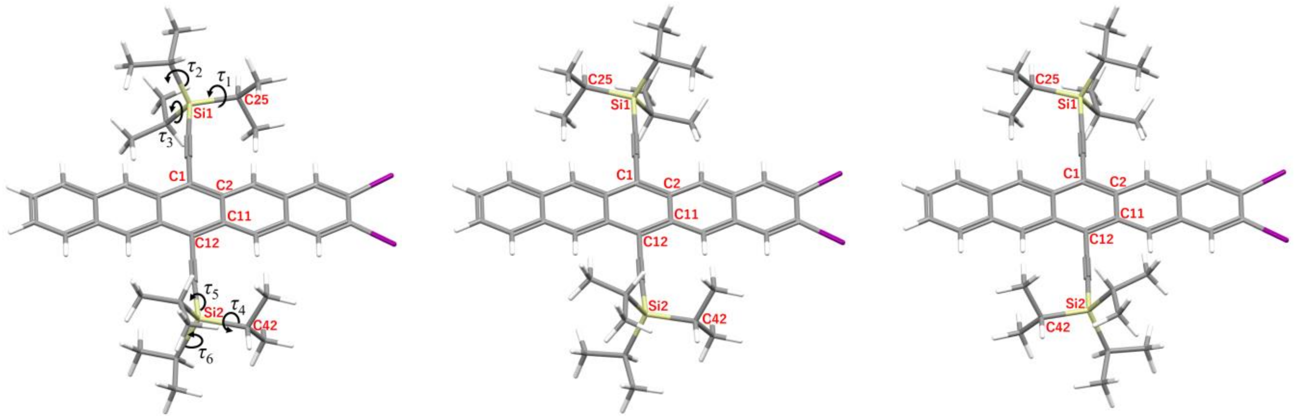

(a) (b) (c)

Figure 1. The (a) lowest, (b) 3rd lowest, and (c) 7th lowest energy conformers of XXVII based on MMFF94s potential. The conformers have about 0 or 180 degrees in the torsion angles of C2, C1, Si1, and C25 and C11, C12, Si2, and C42.

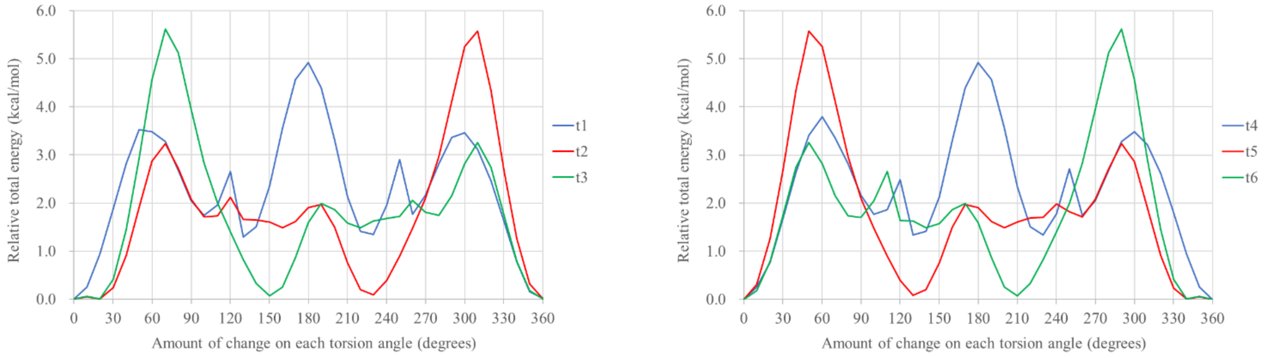

(a) (b)

Figure 2. Torsion potentials of τ_1, τ_2, τ_3, τ_4, τ_5, and τ_6 of the lowest energy conformer. The initial angles of τ_1, τ_2, τ_3, τ_4, τ_5, and τ_6 are -178.97, 49.32, -67.88, 178.97, -49.32, and 67.88 degrees, respectively.

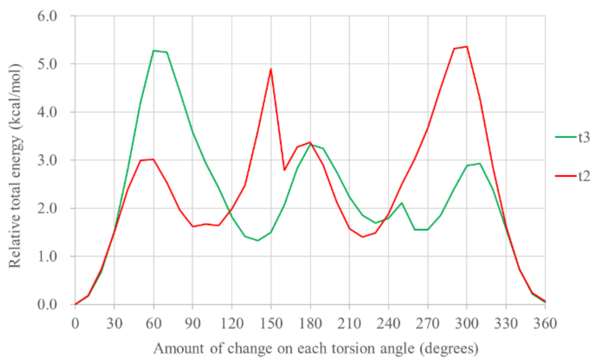

Figure 3. Torsion potentials of τ_3 and τ_2 with respect to the conformers at around 230 and 150 degrees on the torsion potentials of τ_2 and τ_3 of the lowest energy conformer, respectively.

Landscape generation:

The unique crystal structures obtained by the structure generation were ranked according to their crystal energies based on MMFF94s potential, and we selected top 2000 structures. The 2000 structures were subjected to energy calculation by PBE-D3 scheme under periodic boundary condition with kinetic energy cutoffs of 80 Ry and 800 Ry for the wavefunction and charge density, respectively, and the *k*-point mesh spacing of about 0.20 $Å^{-1}$, and we determined their final energies. The 2000 structures were re-ranked according to their final energies, and top 1500 structures were submitted.

Target XXIX

Structure generation:

Two conformers were obtained by the conformation search (Figure 4). In the generation of initial crystal structures, all conformers were used, and the number of molecules in the asymmetric unit was set to one (*Z*'=1) and two (*Z*'=2). The molecules in the asymmetric unit with *Z*'=2 initially have the same conformation. In the case of *Z*'=1, we used 19 space groups (*P*1, *P*1-, $P2_1$, *C*2, *Pc*, *Cc*, $P2_1/m$, *C*2/*m*, *P*2/*c*, $P2_1/c$, *C*2/*c*, $P2_12_12$, $P2_12_12_1$, $Pca2_1$, $Pna2_1$, *Pccn*, *Pbcn*, *Pbca*, and *Pnma*) and molecular rotational step of 20 degrees. In the case of *Z*'=2, we used 2 space groups (*P*1- and $P2_1/c$) and applied rotational step of 45 degrees to each molecule in the asymmetric unit.

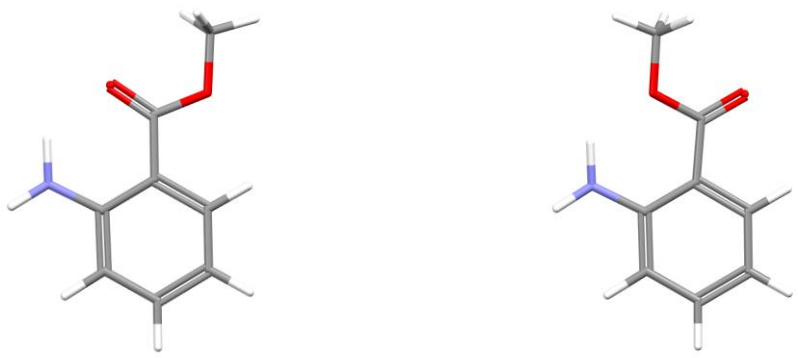

(a) (b)

Figure 4. The (a) lowest and (b) 2nd lowest energy conformers of XXIX based on MMFF94s potential.

Landscape generation:

The unique crystal structures obtained by the structure generation within about 4 kcal/mol from the global minimum in the crystal energy were ranked based on their assessment values that were calculated by the crystal energy and similarity between calculated and reference PXRD patterns [1]. The crystal energy is based on MMFF94s potential. To make the reference PXRD data, the figure of simulated PXRD pattern of an experimentally observed structure provided by CCDC blind test team was converted into numerical data by RODEM program [24] and background in the pattern was subtracted by DASH program [25]. Top 1500 structures based on the ranking were submitted.

A list of 10 structures:

Top 50 structures based on the ranking by the assessment value were re-optimized by PBE-D3 scheme. In the optimization, all degrees of freedom for representing the crystal structure, that is, atomic positions and unit cell dimensions were relaxed with restriction of space group symmetry under periodic boundary condition. The kinetic energy cutoffs were set to 50 Ry and 500 Ry for the wavefunction and charge density, respectively, and the *k*-point mesh spacing was set to about 0.30 $Å^{-1}$. The convergence thresholds on total energy and forces for ionic minimization and on pressure for variable cell relaxation were set to 1.0×10^{-5} a.u, 1.0×10^{-4} a.u, and 5.0×10^{-2} kbar, respectively. The re-optimized 50 structures were ranked based on new assessment values that were calculated using PBE-D3 energy and PXRD pattern similarity, and top 10 structures were submitted.

Post analysis:

Z'=3 case also had to be considered to get the actual structure.

Figure 5 shows crystal energy and assessment value landscapes of XXIX. These figures were made using the crystal structures obtained by the structure generation and the experimental crystal structure optimized by our method. The energy based on MMFF94s potential is used in the figures. In the assessment value landscape, the structures are arranged depending on PXRD pattern similarity unlike the crystal energy landscape (Figure 5), and the experimental crystal structure optimized shows 2nd, 100th, and 269th places in rankings by the assessment value, crystal energy, and PXRD similarity, respectively. Therefore, using the assessment value, the actual crystal structure can be identified with a high accuracy from computationally predicted structures, and our approach works in the experimentally assisted challenge.

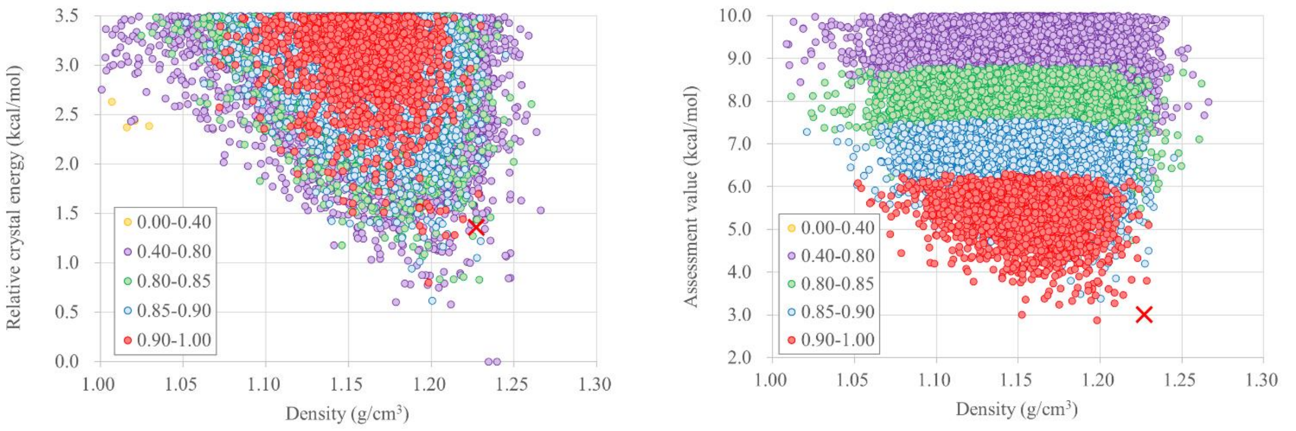

(a) (b)

Figure 5. (a) Crystal energy and (b) assessment value landscapes of XXIX. Each symbol represents one crystal structure. The color coding of symbols is defined according to PXRD pattern similarity value. The experimental crystal structure optimized by our method is indicated by the cross mark.

Target XXX

Method for stoichiometry prediction:

For the stoichiometry prediction, we use a cohesive energy of molecules (i.e., lattice energy) defined by

$$E_{\mathrm{coh}} = \Delta E_{\mathrm{intra}} + E_{\mathrm{inter}} \qquad (2)$$

$$\Delta E_{\mathrm{intra}} = E_{\mathrm{intra,solid}} - E_{\mathrm{intra,gas}} \qquad (3)$$

where the $E_{\mathrm{intra,solid}}$ is the sum of intramolecular energies of molecules in asymmetric unit and the $E_{\mathrm{intra,gas}}$ is the sum of each total energy of the molecules at isolated sate in gas phase. The $\Delta E_{\mathrm{intra}}$ means an energy difference on conformational change from gas to solid phases. The E_{inter} is the sum of intermolecular interaction energies per asymmetric unit in the crystal. Therefore, the E_{coh} equals to a value obtained by subtracting the $E_{\mathrm{intra,gas}}$ from the E_{crystal}. The energy is estimated by MMFF94s potential.

In the stoichiometry prediction of XXX, we estimated tetramethylpyrazine (TMP) and cannabinol (CBN) in 1:1, 2:1, and 1:2 stoichiometries, respectively. The co-crystals with different stoichiometries were estimated by

$$\Delta E_{\mathrm{coh}}(N{:}M, \mathrm{TMP{:}CBN}) = E_{\mathrm{coh}}(N{:}M, \mathrm{TMP{:}CBN}) - NE_{\mathrm{coh}}(\mathrm{TMP}) - ME_{\mathrm{coh}}(\mathrm{CBN}) \qquad (4)$$

where the $E_{\mathrm{coh}}(N{:}M, \mathrm{TMP{:}CBN})$ is cohesive energy of crystal structure of TMP and CBN in *N*:*M* stoichiometry, and the $E_{\mathrm{coh}}(\mathrm{TMP})$ and $E_{\mathrm{coh}}(\mathrm{CBN})$ are minimum cohesive energy of TMP and CBN crystals with *Z*'=1, respectively. Therefore, in case of a negative value of the $\Delta E_{\mathrm{coh}}(N{:}M, \mathrm{TMP{:}CBN})$, the co-crystallization of TMP and CBN yields greater cohesive energy gain than each crystallization of TMP and CBN, that is, the co-crystallization is expected to occur, and vice versa for a positive value [26-31].

Structure generation:

Five conformers of CBN were obtained by the conformation search with an assumption that the conformation of alkyl chain is all trans (Figure 6). TMP has only one conformation. The five conformers of CBN and one conformer of TMP were used for the structure generation process.

In the structure generation for TMP and CBN co-crystals in 1:1 and 2:1 stoichiometries, we used two common space groups of *P*1- and $P2_1$/c, and in case of TMP and CBN co-crystal in 1:2 stoichiometry, three subgroups of the two common space groups, that is, *P*1, $P2_1$, and *Pc* were employed considering inversion symmetry of TMP. In the structure generation for TMP and CBN co-crystals in 1:1 and 1:2 stoichiometries, we performed extra crystal structure searches using $P2_1$, $C2/c$, $P2_12_12_1$, and *Pbca* and *C*2, *Cc*, $P2_12_12_1$, and $Pca2_1$, respectively, since the stoichiometries show lower cohesive energy in the first crystal structure search. In the extended search, we employed a random search algorithm in the generation of initial crystal structures, and the molecular orientation and spatial position were determined randomly. The rotational step of 45 degrees was applied to each molecule in the asymmetric unit.

In the structure generation for CBN crystal, we used 10 common space groups of *P*1, *P*1-, $P2_1$, *Cc*, $P2_1/c$, $C2/c$, $P2_12_12_1$, $Pna2_1$, *Pbca*, and *Pnma*. In TMP case, three space groups of *C*2, *Pc*, and $Pca2_1$ were added to the 10 space groups because of inversion symmetry of TMP. The rotational step of 20 degrees was employed and the number of molecules in the asymmetric unit was set to one (*Z*'=1).

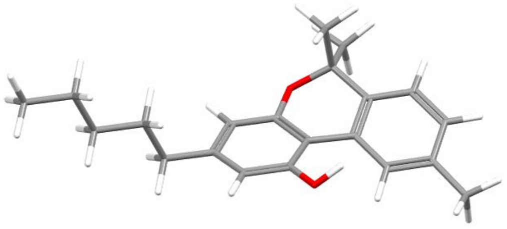

(a)

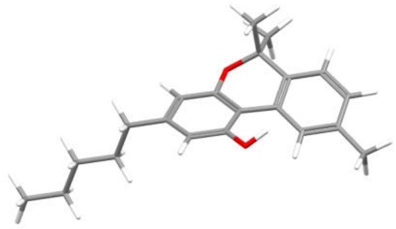

(b)

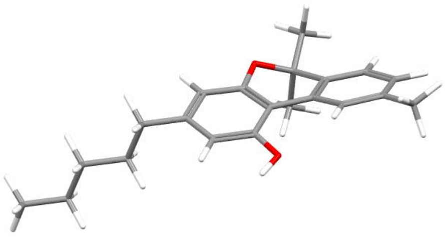

(c)

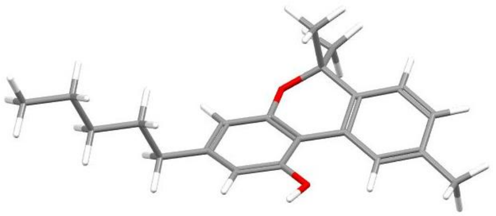

(d)

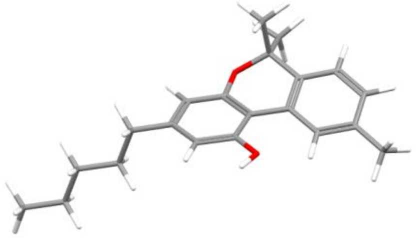

(e)

Figure 6. Conformers of cannabinol based on MMFF94s potential. The conformers are ordered based on their conformational energies.

Landscape generation:

Each energy landscape of crystal structures with TMP and CBN in 1:1, 2:1, and 1:2 stoichiometries was made according to their cohesive energies. The 629 and 667 structures with TMP and CBN in 1:1 and 2:1 stoichiometries within 4 kcal/mol from the global minima, respectively, and the 204 structures with TMP and CBN in 1:2 stoichiometry within 7 kcal/mol from the global minimum were selected for submission of 1500 structures.

For submission of 100 structures, the 1500 structures with the different stoichiometries were ranked according to the ΔE_{coh} and top 100 structures were submitted.

Stoichiometry prediction:

In our estimation, the likely observed stoichiometry of TMP and CBN co-crystal is 1:2 and 1:1 since the stoichiometries show negative value in the ΔE_{coh}. The co-crystal of TMP and CBN in 2:1 stoichiometry that shows positive value in the ΔE_{coh} is not likely to be observed.

Post analysis:

We extended the structure generation for CBN crystal up to *Z*'=2 since the observed crystal structure of CBN shows *Z*'=2 [32], although the experimental information wasn't used in the blind test of XXX. In the extended structure generation, all conformers of CBN and two comm space groups of $P1$- and $P2_1/c$ were used, and the rotational step of 45 degrees was applied to each molecule in the asymmetric unit. The molecules in the asymmetric unit initially have the same conformation. Figure 7 (a) shows crystal energy landscape of CBN. We confirmed that the structure corresponding to the observed one is included in the structures obtained by the structure generation (Figure 7 (a) and Figure 8 (a)). The structure shows 2nd place in ranking by MMFF94s potential. Furthermore, we found that the minimum energy of CBN crystal with *Z*'=2, that is, it has the actual *Z*' value, is slightly lower than that of CBN crystal with *Z*'=1, with the difference of about 0.6 kcal/mol (Figure 7 (a)). In addition, it was confirmed that the result of stoichiometry prediction is the same whether the minimum energy of crystal with *Z*'=1 or crystal with *Z*'=2 is used in the equation 4.

The crystal energy landscape of TMP is also shown in Figure 7 (b). We confirmed that the structure corresponding to the observed one of TMP [33] is included in the structures obtained by the structure generation (Figure 7 (b) and Figure 8 (b)). The structure shows 11th place in ranking by MMFF94s potential.

In the blind test, we could not obtain the predicted structures corresponding to the observed ones of XXX due to the assumption that the conformation of alkyl chain of CBN is all trans and the consideration of inversion symmetry of TMP in the generation of initial crystal structures.

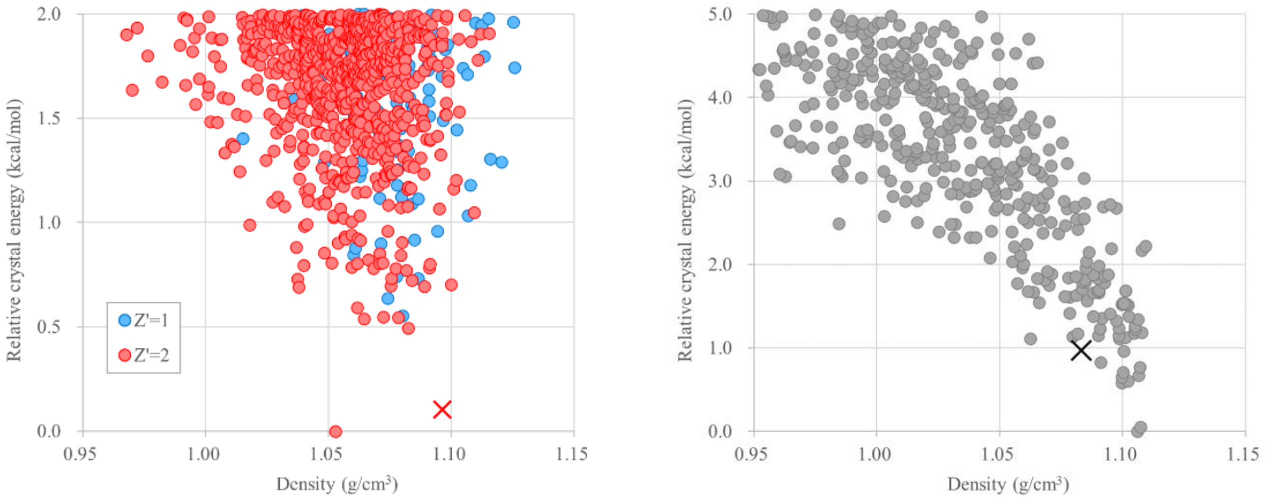

(a) (b)

Figure 7. Crystal energy landscapes of (a) cannabinol and (b) tetramethylpyrazine. Each symbol represents one crystal structure. The structure corresponding to the observed one is indicated by the cross mark.

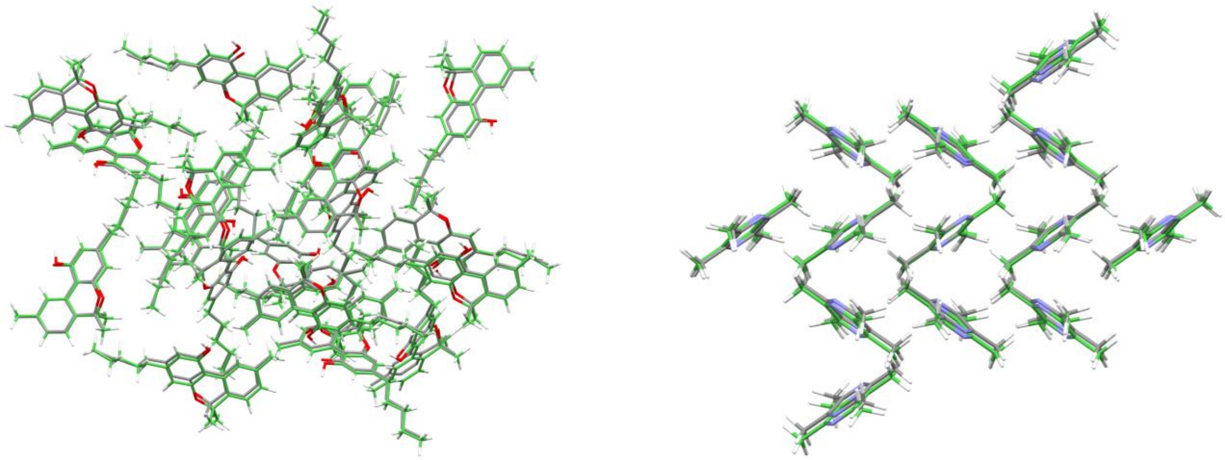

(a) (b)

Figure 8. Superpositions of predicted and observed structures of (a) cannabinol and (b) tetramethylpyrazine. RMSD$_{20}$ values of (a) and (b) are 0.279 and 0.239 Å at the calculations with distance and angle tolerances of 25 % and 25 deg., respectively.

Target XXXI

Structure generation:

Eleven conformers were obtained by the conformation search (Figure 9). In the generation of initial crystal structures, we employed all conformers, 19 common space groups (*P*1, *P*1-, $P2_1$, *C*2, *Pc*, *Cc*, $P2_1/m$, *C*2/*m*, *P*2/*c*, $P2_1/c$, *C*2/*c*, $P2_12_12$, $P2_12_12_1$, $Pca2_1$, $Pna2_1$, *Pccn*, *Pbcn*, *Pbca*, and *Pnma*), and molecular rotational step of 20 degrees. The number of molecules in the asymmetric unit was set to one.

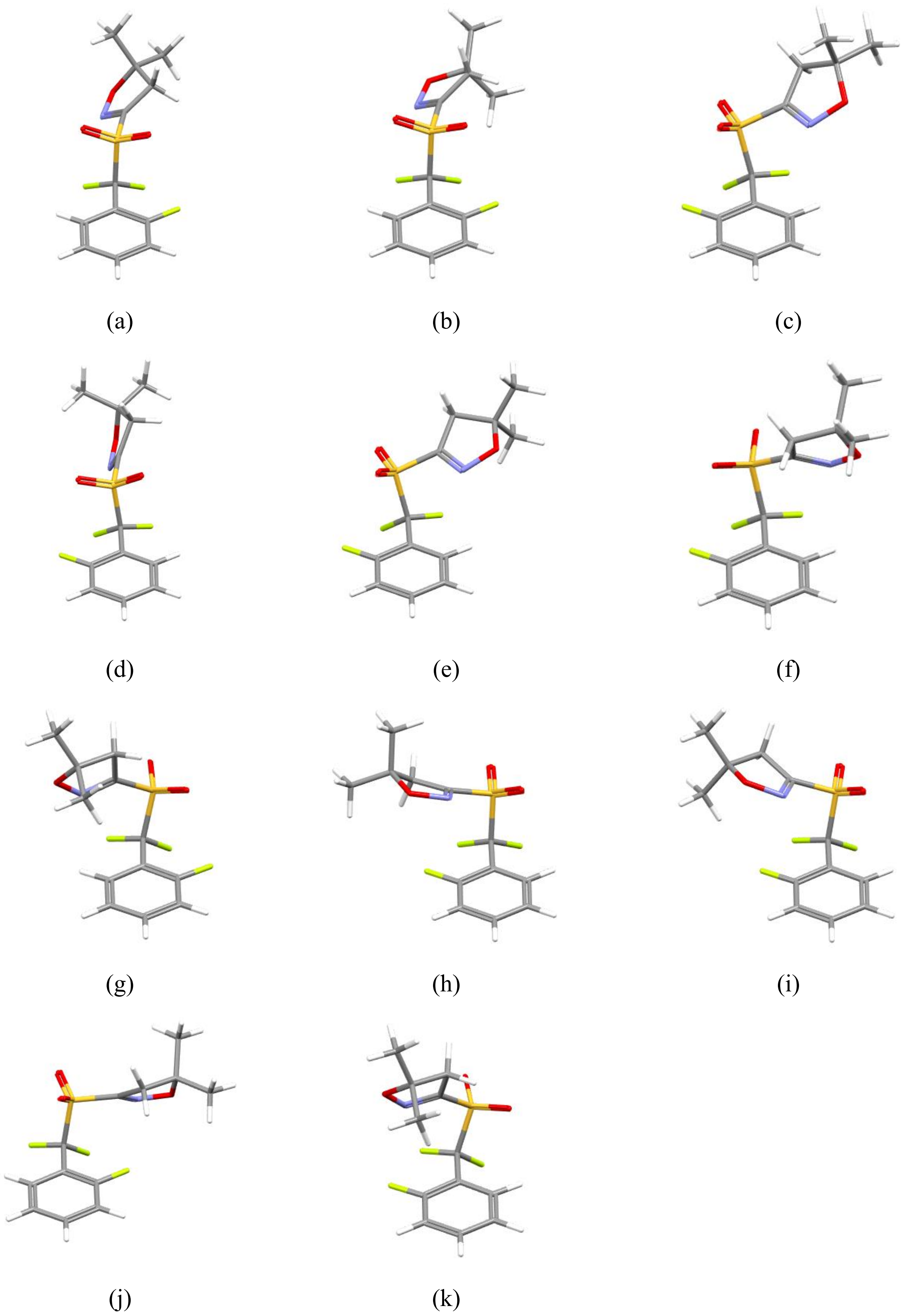

Figure 9. Conformers of XXXI based on MMFF94s potential. The conformers are ordered based on their conformational energies.

Landscape generation:

The unique structures obtained by the structure generation were ranked according to their crystal energies based on MMFF94s potential, and we selected top 2000 structures. The 2000 structures were subjected to energy calculation by PBE-D3 scheme under periodic boundary condition with kinetic energy cutoffs of 80 Ry and 800 Ry for the wavefunction and charge density, respectively, and the *k*-point mesh spacing of about 0.20 Å^{-1}. 1500 structures selected based on PBE-D3 energy estimation were further estimated by FMO-MP2 with 6-31G† basis set, and we determined their final energies. In FMO calculation, molecular clusters with crystal radius of 12 Å were used to estimate energy of the crystal structures, and one molecule was treated as one fragment. A rank of the 1500 structures was determined according to their final energies.

The absolute lattice energy per molecule, with respect to isolated gas-phase molecule in the lowest energy conformation, was calculated by subtracting energy of the isolated gas-phase molecule from the final energy of crystal structure. To get the energy of the isolated gas-phase molecule, the 11 conformers were subjected to energy calculation by FMO-MP2 with 6-31G† basis. As the results, the 8th lowest energy conformer based on MMFF94s potential shows the lowest energy on FMO-MP2 calculation, therefore, it was used as the energy of the isolated gas-phase molecule.

Post analysis:

We confirmed that the crystal structure corresponding to polymorph A with minor fluorine atom position (A_{Min}) is included in the structures obtained by the structure generation (Figure 10). However, it shows 4.09 kcal/mol in the relative crystal energy from the global minimum and 3,341st place in the ranking based on MMFF94s potential. Therefore, the predicted structure was unfortunately excluded from the candidates for the submission. Furthermore, the post analysis clarified that MMFF94s potential can't represent conformations in the observed crystal structures of polymorphs A with major fluorine atom position (A_{Maj}), B, and C (Figures 9 and 11) although MP2/6-31G(d) calculation can provide them (Figure 12). Modification of force field parameters, especially torsion potential parameters, in MMFF94s potential is needed.

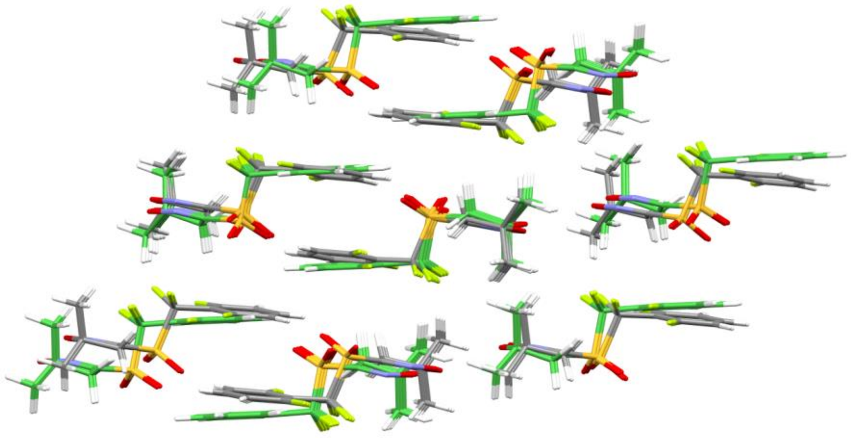

Figure 10. Superposition of predicted and observed structures of XXXI polymorph A_{Min}. $RMSD_{20}$ is 0.888 Å at the calculation with distance and angle tolerances of 30 % and 30 deg., respectively.

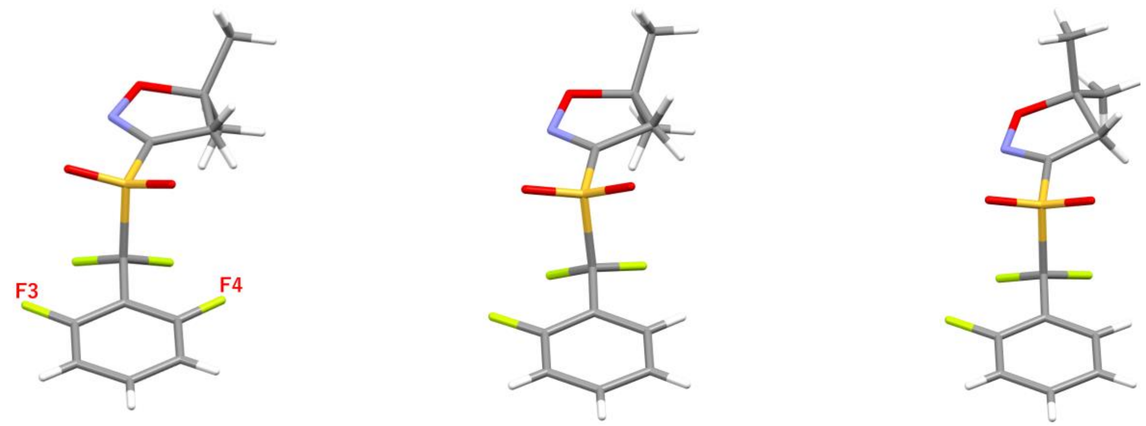

(a) (b) (c)

Figure 11. Conformations in the observed polymorphs (a) A, (b) B, and (c) C. The fluorine atoms labeled as F3 and F4 in the figure (a) are major and minor atomic positions, respectively.

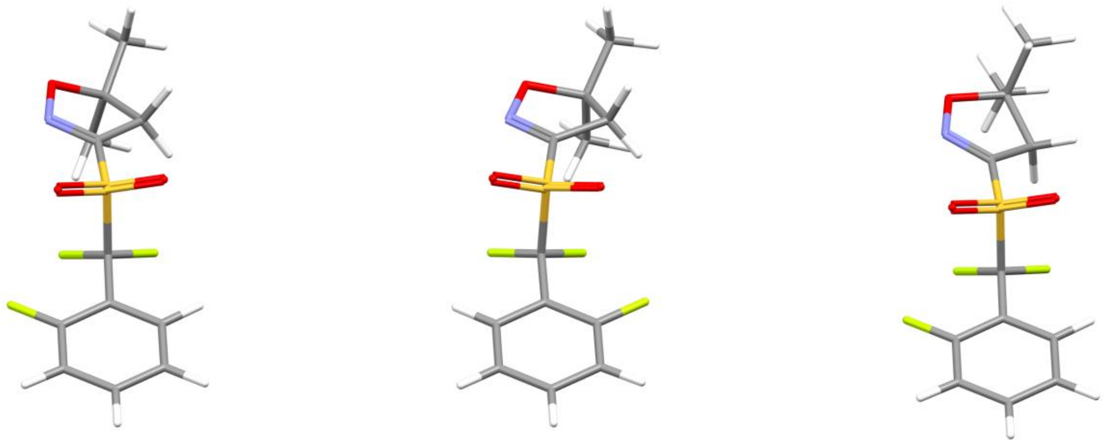

(a) (b) (c)

Figure 12. Conformers in the polymorphs (a) A_{Maj}, (b) A_{Min}, and (c) C optimized by MP2/6-31G(d) calculations at isolated system. The conformer in the polymorph B converged to the same structure as in figure (a) in the MP2 calculation.

Target XXXIII

Structure generation:

Six and four conformers of sulfamethoxazole anion and morpholinium were obtained by the conformation searches, respectively (Figures 13 and 14). Four ionic pairs were generated using four conformers of sulfamethoxazole anion (Figures 13 (a), (b), (d), and (e)) and the lowest energy conformer of morpholinium (Figure 14 (a)). In the generation of initial crystal structures, each ionic pair was used as asymmetric unit, the 10 common space groups ($P1$, $P1$-, $P2_1$, Cc, $P2_1$/c, $C2/c$, $P2_12_12_1$, $Pna2_1$, $Pbca$, and $Pnma$) were used, and the rotational step of 45 degrees was applied to each molecule in the asymmetric unit.

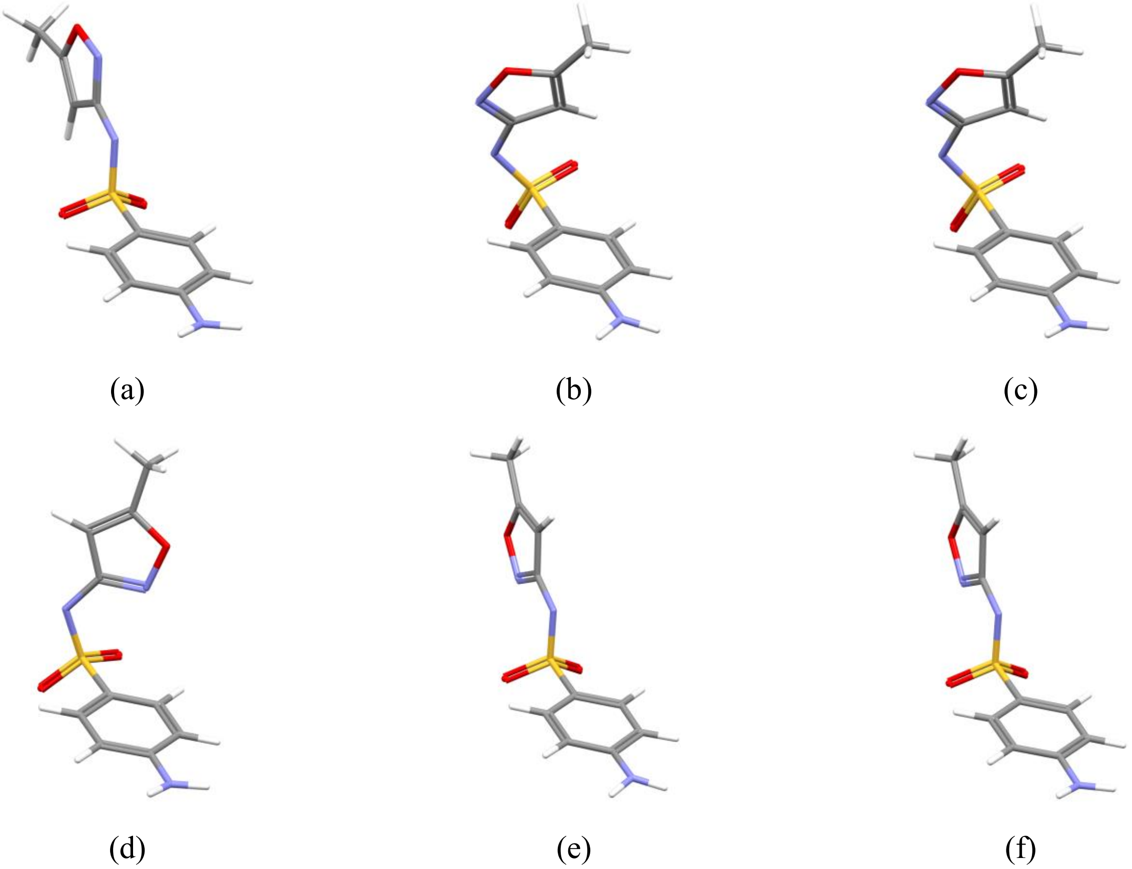

Figure 13. Conformers of sulfamethoxazole anion based on MMFF94s potential. The conformers are ordered based on their conformational energies.

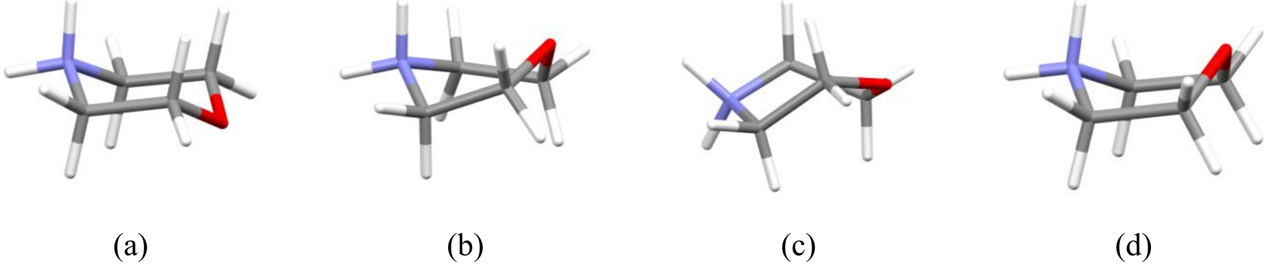

Figure 14. Conformers of morpholinium based on MMFF94s potential. The conformers are ordered based on their conformational energies.

Landscape generation:

The unique crystal structures obtained by the structure generation were ranked according to their crystal energies based on MMFF94s potential, and we selected top 2000 structures. The 2000 structures were subjected to energy calculation by PBE-D3 scheme under periodic boundary condition with kinetic energy cutoffs of 80 Ry and 800 Ry for the wavefunction and charge density, respectively, and the *k*-point mesh spacing of about 0.20 $Å^{-1}$, and we determined their final energies. The 2000 structures were re-ranked according to their final energies, and top 1500 structures were submitted.

Post analysis:

We confirmed that the crystal structure corresponding to polymorph B is included in the structures obtained by the structure generation (Figure 15). However, it shows 6.79 kcal/mol in the relative crystal energy from the global minimum and 2,960th in the ranking based on MMFF94s potential. Therefore, the predicted structure was unfortunately excluded from the candidates for the submission. When the crystal energies of 2,960 structures were estimated by PBE-D3 scheme, the crystal structure corresponding to polymorph B showed the lowest energy.

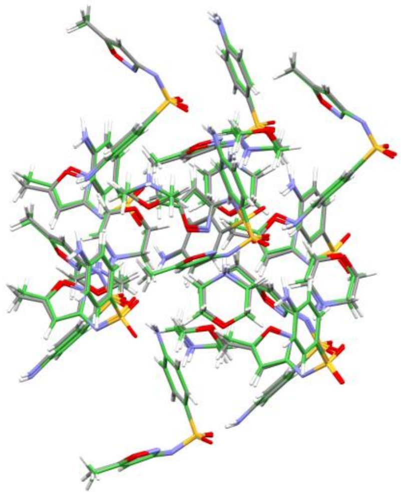

Figure 15. Superposition of predicted and observed structures of XXXIII polymorph B. $RMSD_{20}$ is 0.207 Å at the calculation with distance and angle tolerances of 25 % and 25 deg., respectively.

Computational costs

Table 2. Summary of computational costs

Target	CPU time (h)[a] Structure generation	PBE-D3 calc. / FMO calc.	Resource[b]
XXVII	231,208	102,378	Intel Xeon Gold 6154 CPU 3.00 GHz

XXIX	17,488	75,402	Intel Xeon Gold 6132 CPU 2.60 GHz

XXX	580,436	---	Intel Xeon Gold 6154 CPU 3.00 GHz

XXXI	29,578	30,974	Intel Xeon Gold 6132 CPU 2.60 GHz
		1,829,097	FUJITSU A64FX CPU 2.2 GHz
XXXIII	440,852	36,358	Intel Xeon Gold 6154 CPU 3.00 GHz
		---	

[a] The CPU time was normalized into that when using the CPU shown in the Resource column. [b] The upper resource was used in the structure generation and PBE-D3 calculation and the lower one was used in FMO calculation.

Author contributions

S.O. and H.G. conceived and designed the works of XXVII, XXIX, XXX, and XXXIII. S.O. and H.G. conceived and designed the work of XXXI with K.F. and E.Y. S.O. performed the structure generations and assessment value estimations. Y.I. and S.O. performed PBE-D3 calculations. Y.U. and K.O. performed FMO calculations. N.N. helped with FMO calculation. Authors of submissions for each target analyzed the results for the corresponding target. S.O. wrote the SI and all authors approved the final version.

Acknowledgements

S.O. and H.G. thank Prof. Dr. S. L. Price for her valuable advice on our prediction of XXX. In this work, we used the computer resources by Research Institute for Information Technology, Kyushu University, ACCMS, Kyoto University, and Information and Media Center, Toyohashi University of Technology. Part of this work used computational resources of Fugaku supercomputer through the HPCI System Research Project (Project ID: hp220143). The FMO calculations were performed in the activities

of the FMO drug design consortium (FMODD). This work was supported by JSPS KAKENHI Grant Numbers 17H06373 (H.G.), 21K05002 (Y.I.), and 21K05105 (N.N.).

References

[1] Ishii, H., Obata, S., Niitsu, N., Watanabe, S., Goto, H., Hirose, K., Kobayashi, N., Okamoto, T. & Takeya, J. (2020). *Sci. Rep.* **10**, 2524.

[2] Goto, H. & Osawa, E. (1989). *J. Am. Chem. Soc.* **111**, 8950–8951.

[3] Goto, H. & Osawa, E. (1993). *J. Chem. Soc., Perkin Trans.* **2**, 187–198.

[4] Halgren, T. A. (1996). *J. Comput. Chem.* **17**, 490–519.

[5] Obata, S. & Goto, H. (2015). *AIP Conf. Proc.* **1649**, 130–134.

[6] Ewald, P. P. (1921). *Ann. Phys.* **369**, 253.

[7] Spek, A. L. (2009). *Acta Cryst.* **D65**, 148–155.

[8] BIOVIA, Dassault Systèmes, BIOVIA Materials Studio 2018, San Diego: Dassault Systèmes (2017).

[9] De Gelder, R., Wehrens, R. & Hageman, J. A. (2001). *J. Comput. Chem.* **22**, 273–289.

[10] Groom, C. R., Bruno, I. J., Lightfoot, M. P. & Ward, S. C. (2016). *Acta Cryst.* **B72**, 171–179.

[11] Giannozzi, P., Baroni, S., Bonini, N., Calandra, M., Car, R., Cavazzoni, C., Ceresoli, D., Chiarotti, G. L., Cococcioni, M., Dabo, I., Dal Corso, A., de Gironcoli, S., Fabris, S., Fratesi, G., Gebauer, R., Gerstmann, U., Gougoussis, C., Kokalj, A., Lazzeri, M., Martin-Samos, L., Marzari, N., Mauri, F., Mazzarello, R., Paolini, S., Pasquarello, A., Paulatto, L., Sbraccia, C., Scandolo, S., Sclauzero, G., Seitsonen, A. P., Smogunov, A., Umari, P. & Wentzcovitch, R. M. (2009). *J. Phys. Condens. Matter* **21**, 395502.

[12] Giannozzi, P., Andreussi, O., Brumme, T., Bunau, O., Buongiorno Nardelli, M., Calandra, M., Car, R., Cavazzoni, C., Ceresoli, D., Cococcioni, M., Colonna, N., Carnimeo, I., Dal Corso, A., de Gironcoli, S., Delugas, P., DiStasio, R A., Ferretti, A., Floris, A., Fratesi, G., Fugallo, G., Gebauer, R., Gerstmann, U., Giustino, F., Gorni, T., Jia, J., Kawamura, M., Ko, H.-Y., Kokalj, A., Kucukbenli, E., Lazzeri, M., Marsili, M., Marzari, N., Mauri, F., Nguyen, N. L., Nguyen, H.-V., Otero-de-la-Roza, A., Paulatto, L., Ponce, S., Rocca, D., Sabatini, R., Santra, B., Schlipf, M., Seitsonen, A. P., Smogunov, A., Timrov, I., Thonhauser, T., Umari, P., Vast, N., Wu, X. & Baroni, S. (2017). *J. Phys. Condens. Matter* **29**, 465901.

[13] Perdew, J. P., Burke, K. & Ernzerhof, M. (1996). *Phys. Rev. Lett.* **77**, 3865–3868.

[14] Grimme, S., Antony, J., Ehrlich, S. & Krieg, H. (2010). *J. Chem. Phys.* **132**, 154104.

[15] Dal Corso, A. (2014). *Comput. Mater. Sci.* **95**, 337–350.

[16] Björkman, T. (2011). *Comput. Phys. Commun.* **182**, 1183–1186.

[17] Kitaura, K., Ikeo. E., Asada, T., Nakano, T. & Uebayasi, M. (1999). *Chem. Phys. Lett.* **313**,701–706.

[18] Mochizuki, Y., Nakano, T., Koikegami, S., Tanimori, S., Abe, Y., Nagashima, U. & Kitaura, K. (2004). *Theor. Chem. Acc.* **112**, 442–452.

[19] Petersson, G. A., Bennett, A., Tensfeldt, T. G., Al-Laham, M. A., Shirley, W. A. & Mantzaris, J. (1988). *J. Chem. Phys.* **89**, 2193–2218.

[20] Petersson, G. A. & Al-Laham, M. A. (1991). *J. Chem. Phys.* **94**, 6081–6090.

[21] Utsumi, Y., Umeda, D., Okuwaki, K., Obata, S., Nakayama, N., Goto, H., Furuishi, T., Fukuzawa, K. & Yonemochi, E. (2021). *J. Comput. Chem. Jpn.* **20**, 92–93.

[22] Utsumi, Y., Okuwaki, K., Umeda, D., Obata, S., Nakayama, N., Goto, H., Furuishi, T., Fukuzawa, K. & Yonemochi, E. to be submitted.

[23] Swartz, C. R., Parkin, S. R., Bullock, J. E., Anthony, J. E., Mayer, A. C. & Malliaras, G. G. (2005). *Org. Lett.* **7**, 3163–3166.

[24] RODEM v2.0.4, MoDeCH, Tokyo, Japan (2021)

[25] David, W. I. F., Shankland, K., van de Streek, J., Pidcock, E., Motherwell, W. D. S. & Cole, J. C. (2006). *J. Appl. Cryst.* **39**, 910–915.

[26] Karamertzanis, P. G., Kazantsev, A. V., Issa, N., Welch, G. W. A., Adjiman, C. S., Pantelides, C. C. & Price, S. L. (2009). *J. Chem. Theory Comput.* **5**, 1432−1448.

[27] Issa, N., Barnett, S. A., Mohamed, S., Braun, D. E., Copley, R. C. B., Tocher, D. A. & Price, S. L. (2012). *CrystEngComm* **14**, 2454−2464.

[28] Chan, H. C. S., Kendrick, J., Neumann, M. A. & Leusen, F. J. J. (2013). *CrystEngComm* **15**, 3799−3807.

[29] Taylor, C. R. & Day, G. M. (2018). *Cryst. Growth Des.* **18**, 892–904.

[30] Shunnar, A. F., Dhokale, B., Karothu, D. P., Bowskill, D. H., Sugden, I. J., Hernandez, H. H., Naumov, P. & Mohamed, S. (2020). *Chem. Eur. J.* **26**, 4752−4765.

[31] Sun, G., Jin, Y., Li, S., Yang, Z., Shi, B., Chang, C. & Abramov, Y. A., (2020). *J. Phys. Chem. Lett.* **11**, 8832–8838.

[32] Ottersen, T., Rosenqvist, E., Turner, C. E. & El-Feraly, F. S. (1977). *Acta Chem. Scand.* **31**, 781–787.

[33] Braam, A. W. M. & Eshuis, A., Vos, A. (1981). *Acta Crystal.* **B37**, 730–732.

16. Group 22

Group 22 Supplementary Information

1. Structure generation.

For all targets except XXX (stoichiometry prediction) we presented results of two approaches in the 85/15 ratio. The 85% were obtained using an experimental version of USPEX code with symmetry-preserving variation operators for molecular crystals, and structures are given together with their energies relative to the ground state. The remaining 15% are the lowest-energy structures produced with the public version of the USPEX code – these structures were given as a separate block; since these calculations used a different force field, to avoid confusion these structures were listed without energies. Results for target XXX (stoichiometry prediction) were produced entirely using the latter methodology (public version of USPEX code and relaxations done using GULP and DREIDING force field with charges from Qeq procedure). To generate structures, we considered the five fixed compositions (1:1, 1:2, 2:1, 2:3, 3:2). The generated structures were ranked according to the energy of each structure/composition relative to the convex hull, which is the thermodynamically rigorous way of judging stability of compounds.

Let us describe the two approaches in more detail. The main features of the experimental version of USPEX, compared to an older version, are the space-group-aware heredity and mutation operators, which were previously not available. These operators, by design, preserve the space group of the structure(s) they act upon, which incurs the cost of being able to span only the region of phase space corresponding to the parent structure(s) space group – as a consequence, we had to perform a separate global optimization run for each space group that we wanted to consider. The initial random generation of structures was produced using the PyXtal library [10.1016/j.cpc.2020.107810]. For each target we chose 10-15 most common space groups according to CCDC statistics (for harder cases like target XXVII we used even fewer space groups). We did not explicitly consider point group symmetry of the molecules, therefore all molecules in all structures occupy the general position of the corresponding space group. Z' value was assumed to equal 1 everywhere, except for targets XXIX, XXXI, XXXII, where we also considered Z'=2.
For the global optimization run, we made a decision to use a realistic yet simple model, which led us to use the DREIDING force field together with Mulliken charges obtained from a single-point vacuum calculation for each considered molecule. Then, using this model, each structure in the global optimization run was carefully relaxed using the GULP code [10.1080/0892702031000104887]. Global optimization stopped when the best found structure did not change for 10 generations.

When doing structure prediction using the public version of USPEX, we again considered only the most common space groups – for each of them, we performed a random sampling run with all molecules assumed to occupy the general position of each structure's space group. For this part, a different force field was used, namely AMOEBA.

2. **Structure ranking.**

Here, we used a two-step strategy. First, the synthon approach was used to investigate the extracted CSD-structures of similar systems. Having established the most likely synthons, we analyzed different methods of energy ranking. In the second step, we used the PBE-D3, PBE-

MBD, and PBE0-MBD methods to re-relax and rank structures found in crystal structure prediction runs. Comparing the energy ranking with synthon ranking, we saw that PBE-D3 gives the best consistency and chose this method for energy ranking. Our final results show PBE-D3 energy ranking.

17. Group 23

Supplementary information for predicting the crystal structure of the XXIX molecule

Bingqing Cheng,[1] Jan Gerit Brandenburg,[2] and Chris J. Pickard[3,4,*]

[1]*The Institute of Science and Technology Austria, Am Campus 1, 3400 Klosterneuburg, Austria*

[2]*Merck Data Office, Merck KGaA, Frankfurter Str. 250, 64293 Darmstadt, Germany*

[3]*Department of Materials Science & Metallurgy, University of Cambridge, 27 Charles Babbage Road, Cambridge, CB3 0FS, United Kingdom*

[4]*Advanced Institute for Materials Research, Tohoku University, Sendai, Japan*

* cjp20@cam.ac.uk

SUMMARY

We first trained a *Δ*-learning potential fitted to the difference between PBE and semi-empirical tight-binding DFTB(3ob). We then used the combination of this *Δ*-learning potential, DFTB, and the D3 dispersion as the potential energy surface, and searched for polymorphs by generating initial structures using AIRSS, and then running short molecular dynamics trajectories at 300 K. Finally, all the searched structures were screened using ASAP, and then matched to the provided X-ray pattern.

One-line summary: Polymorph search using a *Δ*-learning potential at a finite temperature

METHODS

Initial structure generation

To generate a set of diverse starting structures for the XXIX molecule an ab initio random structure search (AIRSS) was performed [1]. Initial unrelaxed crystal structures were generated using the *buildcell* code with Z'=1 and Z=2 and 4, choosing random space groups with 2 and 4 symmetry operators in the primitive cell, respectively. Unoptimised conformers of the XXIX molecule were generated from its SMILES using the *openbabel* package [2]. Unit cells were generated with a random shape, an initial volume within 5% of 200 $\mathring{A}^3$/mol and minimum intermolecular distance randomly selected from between 1 and 3 $\mathring{A}$.

AIRSS search

These randomly-generated starting structures were geometry optimised using the CASTEP plane wave DFT code [3], using the PBE density functional with TS correction for dispersion forces. The high throughput QC5 set of on-the-fly pseudopotentials were used, with a high planewave cutoff of 500 eV. The structures were relaxed with the l-BFGS algorithm, using the universal preconditioner [4]. A coarse k-point sampling grid of $0.1 \times 2\pi$ Å^{-1} typically resulted in a single non-Gamma point being used. We thus generated 1,500 structures from the AIRSS search.

Fitting of the Δ-learning potential

We fit Δ-learning potentials to the difference between PBE and semi-empirical tight-binding DFTB(3rd order, 3ob Slater-Koster parametrization) for the XXIX molecule. [5–7] The Δ-learning approach has advantages, because the difference (the Δ part) is typically small as well as smooth and thus can be learned from just a small set of configurations. As such, the computational cost associated with building the training set can be much reduced. In practice, we trained two generations of the Δ-potential to ensure the accuracy.

For the first generation of the potential, we used the following procedures:

- We generated 100 initial structures for the XXIX molecule build initial structures with Z'=1 and Z=2 using *buildcell*.
- For these initial structures, we ran NVT MD simulations at 400 K-600 K and a range of densities. These MD simulations aim to generate thermalized configurations rather than ergodic sampling of the system, so the simulation time was short (5 ps). We used the i-pi code [8] coupled with DFTB+ [9] with DFTB(3ob) parameterization for the MD simulations.
- From the MD trajectories, we selected 2,000 diverse structures using the farthest point sampling technique. The ASAP code [10] allows the fast selection using a simple command line.
- We recomputed the PBE DFT energies for the selected structures using CASTEP [3].
- We fitted Behler-Parrinello [11] neural network potentials to the difference between the DFTB and the PBE energies and forces for these structures. In order to provide uncertainty estimates of the MLPs, we made 4 fits of the potential using different random splits of the training and the test sets.

To further improve the accuracy and the generalizability of the MLP, we fitted the second and the final generation using the following procesures:

- We generated another 3,900 initial structures for the XXIX molecule build initial structures with Z=1, 2, 3, and 4 using *buildcell*.
- We ran NST (constant temperature and stress) MD simulations using a combination of the first-generation Δ-learning potential and DFTB (DFTB(3ob) and the D3 dispersion

parameterized for PBE). The pressure was kept at 0. The addition of the two potential energy surfaces was enabled by the flexibility of the i-pi code [8]. The potential energy and force evaluation of the MLP part was performed using LAMMPS with a Behler-Parrinello [11] neural network implementation [12].

- We selected 2,500 structures from the NST MD trajectories, based on the criterion that the four fits of the MLPs have the largest uncertainties about the energies of the structures (from the standard deviations of the energies predicted by the 4 fits).
- We recomputed the PBE DFT energies for the 2,500 structures using CASTEP [3].
- We used all the 4,500 structures to fit the second and the final version of the Δ-learning potential.

We illustrate the accuracy of the Δ-learning approach in Fig. 1. It can be seen that the combination of the baseline DFTB potential energy surface and the correction terms (green symbols) are in good agreement with the PBE lattice energies, with significant improvement from just using the DFTB PES alone (purple symbols), particularly for the low energy polymorphs.

Structure search using the Δ-learning potential

Using the combination of the DFTB(3ob) (with the D3 dispersion parameterized for PBE) and the Δ-learning potential, we performed a comprehensive search of crystal structures. The initial structures were again built with *buildcell*. In addition, we included the 1,500 structures from AIRSS searches. Most searches are for Z'=1,2,3, 4,6 and symmetry operation=1,2,4. The search uses short MD simulations (1000 steps) at the NST ensemble at zero stress and 300 K. We recorded the average potential energy (PE) during the last 500 steps of the MD run. In order to remove the thermal noise and facilitate the subsequent analysis, we also performed a geometry optimization for each structure obtained from MD, and saved the optimized geometry. We generated a total of 24,790 structures this way.

To showcase the diversity and the energy lanscape of the found structures, in Fig. 2 we illustrate these structures on the molar volume-PE map using chemiscope [13]. Most of the low energy structures have a molar volume of about 210 $\mathring{A}^3$ per molecule. Four structures with the lowest PE all have 3 molecules in the unit cell.

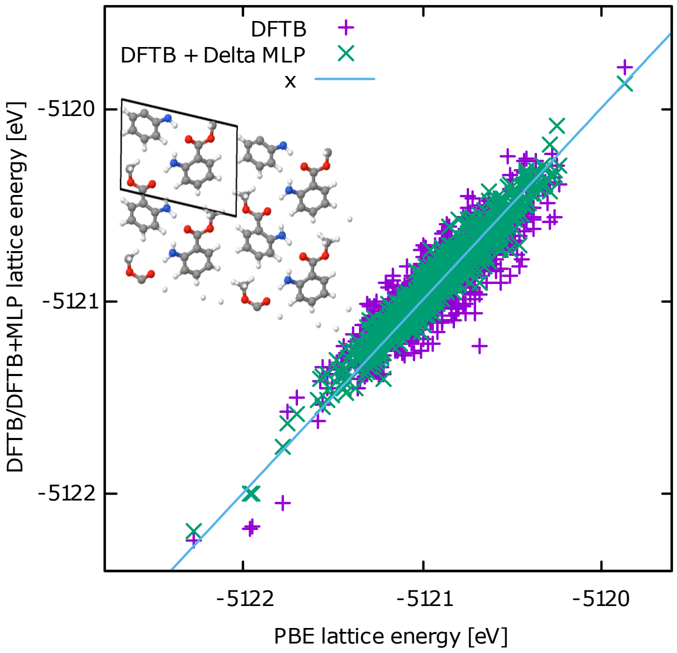

FIG. 1. The comparison between PBE DFT, the baseline semi-empirical tight-binding DFTB(3ob), and the Δ-learning corrected (DFTB+$\Delta U_{\text{PBE-DFTB}}$) methods for the lattice energies of XXIX crytals with Z'=2. One polymorph is illustrated on the inset.

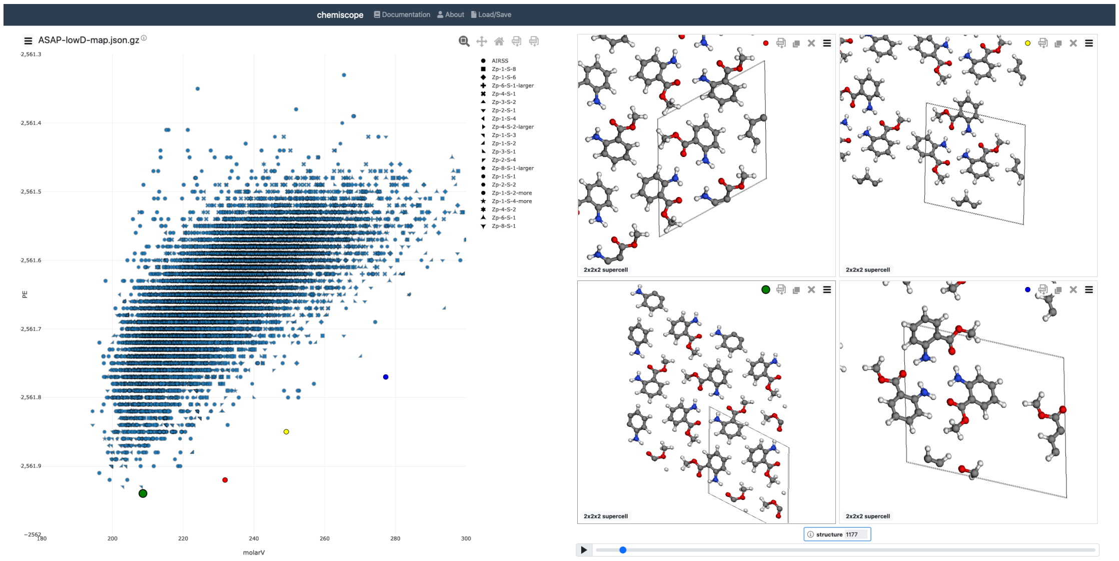

FIG. 2. Chemiscope visualization of all the 24,790 structures found in the structure search using the Δ-learning potential. 4 structures with low PE are illustrated, and all have Z=3.

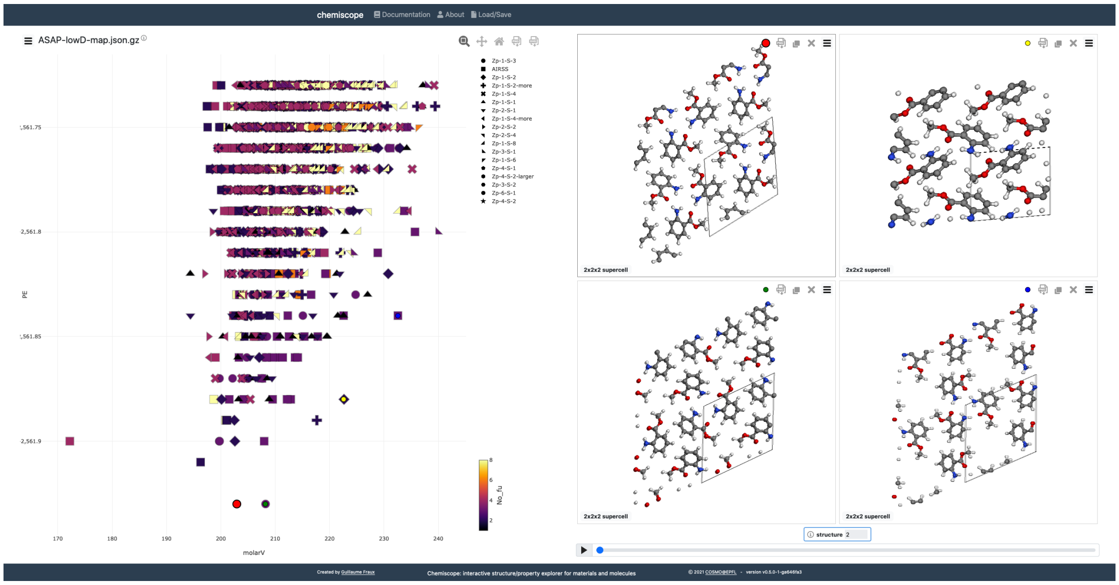

FIG. 3. Chemiscope visualization of the 1500 structures selected. 4 structures with low PE are illustrated.

Analysis of the set of structures found

We then screen the structures found in the search following two strategies: The first is to remove redundant structures using ASAP [10]: generate SOAP descriptors for all the structures, and then remove the structures that have the almost duplicated SOAP descriptors. The second is to remove high energy or high molar volume structures. In the end we kept 1,500 structures, as illustrated in Fig.3.

Matching to the X-ray pattern

The provided pixel graphic of the measured X-ray powder pattern has been digitized with the WebPlotDigitizer version 4.6.[14] The digital powder pattern has been loaded to Match version 2 [15], the background radiation identified and removed, and a light Gaussian smoothening of overall spectrum has been applied, all with default settings. The pre-processed spectrum has been used to match with the computer generated structures. We used the PXRD similarity measure from the CCDC software [16] that gives a similarity measure from 0 (no match) to 1 (perfect match) for all generated crystal structures. The most-likely structures were determined by combining the pareto front of the energy-density landscape with the powder matching quality and the relative lattice energies.

Acknowledgements BC acknowledges resources provided by the Cambridge Tier-2 system operated by the University of Cambridge Research Computing Service funded by EPSRC Tier-2 capital grant EP/P020259/1.

---

[1] C. J. Pickard and R. J. Needs, "Ab initio random structure searching," Journal of Physics: Condensed Matter **23**, 053201 (2011).

[2] O. B. development team, "Open babel," (2016).

[3] S. J. Clark, M. D. Segall, C. J. Pickard, P. J. Hasnip, M. I. J. Probert, K. Refson, and M. C. Payne, "First principles methods using CASTEP," Zeitschrift für Kristallographie - Crystalline Materials **220**, 567 (2005).

[4] D. Packwood, J. Kermode, L. Mones, N. Bernstein, J. Woolley, N. Gould, C. Ortner, and G. Csányi, "A universal preconditioner for simulating condensed phase materials," The Journal of Chemical Physics **144**, 164109 (2016).

[5] M. Elstner, D. Porezag, G. Jungnickel, J. Elsner, M. Haugk, T. Frauenheim, S. Suhai, and G. Seifert, "Self-consistent-charge density-functional tight-binding method for simulations of complex materials properties," Phys. Rev. B **58**, 7260 (1998).

[6] M. Gaus, A. Goez, and M. Elstner, "Parametrization and benchmark of dftb3 for organic molecules," J. Chem. Theory Comput. **9**, 338 (2013).

[7] J. G. Brandenburg and S. Grimme, "Accurate Modeling of Organic Molecular Crystals by Dispersion-Corrected Density Functional Tight Binding (DFTB)," J. Phys. Chem. Lett. **5**, 1785 (2014).

[8] V. Kapil, M. Rossi, O. Marsalek, R. Petraglia, Y. Litman, T. Spura, B. Cheng, A. Cuzzocrea, R. H. Meißner, D. M. Wilkins, *et al.*, "i-pi 2.0: A universal force engine for advanced molecular simulations," Computer Physics Communications **236**, 214 (2019).

[9] B. Hourahine, B. Aradi, V. Blum, F. Bonafé, A. Buccheri, C. Camacho, C. Cevallos, M. Deshaye, T. Dumitrică, A. Dominguez, *et al.*, "Dftb+, a software package for efficient approximate density functional theory based atomistic simulations," The Journal of chemical physics **152**, 124101 (2020).

[10] B. Cheng, R.-R. Griffiths, S. Wengert, C. Kunkel, T. Stenczel, B. Zhu, V. L. Deringer, N. Bernstein, J. T. Margraf, K. Reuter, *et al.*, "Mapping materials and molecules," Accounts of Chemical Research **53**, 1981 (2020).

[11] J. Behler and M. Parrinello, "Generalized neural-network representation of high-dimensional potential-

energy surfaces," Phys. Rev. Lett. **98**, 146401 (2007).

[12] A. Singraber, J. Behler, and C. Dellago, "Library-based LAMMPS implementation of high-dimensional neural network potentials," Journal of Chemical Theory and Computation **15**, 1827 (2019).

[13] G. Fraux, R. K. Cersonsky, and M. Ceriotti, "Chemiscope: Interactive structure-property explorer for materials and molecules," Journal of Open Source Software **5**, 2117 (2020).

[14] A. Rohatgi, "WebPlotDigitizer, Version 4.6 https://github.com/ankitrohatgi/WebPlotDigitizer," Pacifica, CA, USA (2022).

[15] H. Putz and K. Brandenburg, "Match! - Phase Analysis using Powder Diffraction, https://www.crystalimpact.de/match," Crystal Impact, Bonn, Germany (2022).

[16] C. F. Macrae, I. Sovago, S. J. Cottrell, P. T. A. Galek, P. McCabe, E. Pidcock, M. Platings, G. P. Shields, J. S. Stevens, M. Towler, and P. A. Wood, "*Mercury 4.0*: from visualization to analysis, design and prediction," Journal of Applied Crystallography **53**, 226 (2020).

18. Group 24

Detailed methodology for 24-SLPrice-CSPBT7 Submission 1

1 Overview

The Price group have generally used the Ψ_{mol} approach, based on the electronic structure of the molecule, including:

- Assessing the conformational range the molecule may take in its crystal structures
- Generating structures with CrystalPredictor[1] (or MOLPAK[2] in one case)
- Refining those structures with DMACRYS[3] to include a distributed multipole model of the charge distribution for the electrostatic contribution, modelling changes in molecular conformation within the crystal from the packing forces using CrystalOptimizer[4]

In all cases, the molecular conformation and intramolecular energy penalty were calculated using GAUSSIAN09,[5] the conformation specific charge density was calculated in GAUSSIAN09[5] and represented by atomic distributed multipoles up to hexadecapole (for use in DMACRYS[3]) calculated using GDMA2.[6] Unless otherwise stated, exp-6 repulsion-dispersion parameters were taken from the FIT potential[7] including polar hydrogen parameters from Coombes's work on polar organic molecules,[8] chlorine parameters from Williams's work on perchlorohydrocarbons[9] and fluorine parameters from Williams's work on perfluorohydrocarbons.[10] Parameters for other potential types are described for each system. The form of the repulsion-dispersion potential between atom *i* in molecule *M* of type ι and atom *k* in molecule N of type κ separated by distance R_{ik} is given in Equation 1.

$$U = \sum_{i\in M,k\in N} \sqrt{A_{\iota\iota}A_{\kappa\kappa}} \exp\left(-\left(\frac{B_{\iota\iota}+B_{\kappa\kappa}}{2}\right)R_{ik}\right) - \frac{\sqrt{C_{\iota\iota}C_{\kappa\kappa}}}{R_{ik}^{6}}$$

Equation 1.

The total lattice energy was calculated according to Equation 2, where U_{inter} is the intermolecular energy of the crystal relative to the molecules being infinitely separated, calculated by lattice summation of the repulsion-dispersion terms in Equation 1 and the electrostatic contribution to the lattice energy for all terms in the multipole expansion up to R^{-5}, and ΔE_{intra} is the conformational energy penalty with respect to the gas phase optimized conformation.

Equation 2. $$E_{latt} = U_{inter} + \Delta E_{intra}$$

All searches for single component targets were only run with one whole molecule in the asymmetric unit, i.e. Z'=1. XXXIII was only run with one anion and one cation in the asymmetric unit. CrystalPredictor v2.4.3 was used for systems treated as either rigid (XXVIII, XXIX, XXXII) or with simple flexibility (XXXI), while v2.4.3.2 was used for systems where torsion groups were used (XXX, XXXIII). CrystalOptimizer v2.4.7 was usually used (XXIX, XXX, XXXI), while v2.4.8 was used for systems which included PCM correction of the *ab initio* calculations (XXVIII, XXXIII). DMACRYS v2.3.1.1 was used unless otherwise stated.

The atomic numbering is that used in the main paper, but anyone using the .cif files generated by the Price group are advised to check whether the original numbering has been replaced.

As with previous Blind Tests, each molecule comes with its own unique challenges and the specific methodology is different for each. The 7th test introduced some systems where the assumption of the Ψ_{mol} approach, that the charge distribution of a molecule within the crystal is very similar to that of the isolated molecule, is more of an approximation. However, we tackled all of the molecules in the spirit of seeing how far adaptations to our usual methodology could take us. Thus, we have separated the report into sections detailing the conformational space covered, the search methodology, the structure refinement, our degree of confidence in our submission, and a post-result analysis.

2 XXVII – optoelectronic compound

2.1 Conformational details

The CSD was searched for structures of similar compounds, and trends investigated. Most molecular conformations had exactly one isopropyl group on each TIPS group with a H_C_Si_C angle of 180°. The other two groups had this angle at ±60°. Most molecular conformations appeared visually symmetrical across the pentacene group.

Twelve conformations of the molecule were constructed for one TIPS group, with each possible conformation of H_C_Si_C angles so that exactly one was 180°. The groups are labelled by H_C_Si_C angle, starting from the one nearest the iodine atoms and moving anticlockwise ("1" denotes 180°, "m" denotes -60°; "p" denotes +60°). The variables within the other TIPS group were related to this either by inversion ("i") or mirror symmetry about the plane perpendicular to the main axis of the pentacene group ("m"), giving a total of 24 conformational starting points. Each conformation of the molecule was fully optimized with the PBE0 method and the 3-21G basis set, using an iodine radius of 1.98 Å.

2.2 Search methodology

We have previously found that the point charge model used in CrystalPredictor is not suitable for highly conjugated systems such as coronene, so we decided to use MOLPAK as the rigid-molecule structure generation method, which uses a pseudo-hard-sphere optimization of the structures, before passing structures directly to DMACRYS.

Searches were carried out in MOLPAK[2] with each of the 24 optimized conformations. All available packing types were used, covering space groups P1, P-1, P2, P21, C2, Pm, Pc, Cc, P2/m, $P2_1/m$, P2/c, $P2_1/c$, C2/c, $P222_1$, $P2_12_12$, $P2_12_12_1$, Pma2, $Pca2_1$, Pnc2, $Pmn2_1$, Pba2, $Pna2_1$, Pnn2, Pbcn, Pbca. 10° steps were used for the molecule orientation within each cartesian direction, and the 500 densest structures within each packing type were passed to DMACRYS (this value was set so high that nothing was discarded).

2.3 Structure refinement

It was not possible to use CrystalOptimizer for this molecule, since we did not wish to adapt the code to use the iodine extensions in the GAUSSIAN steps.

2.3.1 DMACRYS

For each conformation, the charge density was calculated with the PBE0 method and the 6-311G(d,p) basis set (basis functions for iodine were downloaded from https://www.basissetexchange.org/ and read in), with an iodine radius of 1.98 Å.

A repulsion-dispersion potential was constructed for the molecule as follows:

- Parameters were taken from the FIT potential[7] for the aliphatic carbon atoms (including the sp-hybridized carbon atoms) and the silicon atom also used the carbon parameters (since it is very well shielded). (In the structure generation phase, the aliphatic hydrogens were erroneously assigned the anisotropic hydrogen parameters.)
- Parameters were taken from Graeme Day's anisotropic potential[11] for the pentacene and attached hydrogen and iodine atoms. (In the structure generation phase, only the isotropic part of the potential was used.)

Structures were then optimized with DMACRYS v2.0.8.

Duplicate structures were removed within each conformation's search. Structures were considered duplicates if the energy difference was less than 0.2 kJ mol^{-1}, the difference in unit cell volume was less than 0.5 $Å^3$, the powder pattern similarity was greater than 0.97, and an $RMSD_{15}$ overlay better than 0.5 Å was achieved. Structures were not compared between searches.

2.3.2 Energy landscape reduction

Dr Nicholas Francia attempted to reduce this energy landscape by ambient Molecular Dynamics (MD) simulations of near cubic supercells of side ~ 50 Å, which produced clusters of up to 32 structures. 142 had free rotation of TIPS

groups. Due to timing and the difficulty of comparing the many disordered structures, added to the difficulties of using Crystal Packing Similarity to determine the unique CSP_0 structures, these calculations were not used to reduce the energy landscape in Submission 1, (it could have been a 24% reduction). However, the MD had alerted us to the high probability of dynamic disorder in the TIPS groups. We noted these difficulties in our Submission, where we stated that no structures melted, only a few transformed, and there was good evidence for dynamic disorder of the TIPS groups. Dr Francia has been continuing this work as part of the CCDC team investigating dynamic disorder in the experimental structures.

2.4 Degree of confidence

As CrystalPredictor has to use an atomic charge model and the synthesis of XXVII as an optoelectronic material implied that π-stacking of the conjugated aromatic ring system was expected, the decision to use MOLPAK prioritized the generation of structures with π-stacking through using atomic mutipoles. We feel that we covered the gross conformational space for this molecule well, although flexible refinement of the crystal structures allowing small adjustments of the TIPS groups was not possible. The intention to allow this adjustment through the MD simulations could not be done because of time and difficulty in comparing structures with what seemed the likely physical dynamic disorder. The computational method used for the conformational analysis and charge density calculation was the best we could afford for a molecule of this size. The mixing of the repulsion-dispersion potential from the different sources is not ideal, but was intended to include the anisotropy of the I and π system in the aromatic interactions.

2.5 Post-result analysis

The search generated the observed structure as BXXVII_m1mp_ca21 with a $RMSD_{30}$ = 0.661 Å. The lack of flexibility that could be implemented in the search makes this a good result. When only the core of the molecule is compared, this matches with $RMSD_{30}$ = 0.554 Å.

3 XXVIII – metalorganic compound

Although this test was not "blind," we did not access the experimental .cif file before submission.

3.1 Conformational details

Model molecular conformations were constructed for this molecule, within the range for which the Ψ_{mol} approach was valid, i.e. with Cu and its ligands forming a molecule. Two square-planar-trans conformations were constructed, with the N-H bonds on the same or opposite sides. Similarly, two tetrahedral conformations were constructed, with the N-H bonds on the same or opposite sides. A square-planar-cis conformation was constructed, but only one configuration of the N-H bonds was used.

For each of these geometries of the copper centre, all possible combinations of the phenyl angles were generated. Since they are correlated on adjacent rings, this was a maximum of four (and many were duplicates) for each gross conformation.

In order to preserve the square-planar symmetry, two torsion angles were constrained to 0° or 180° in all square-planar conformations, and the Cl_Cu_Cl angle was constrained to 179.0° in the square-planar-trans or one N_Cu_Cl angle was constrained to 179.0° in the square-planar-cis conformations. All conformations were optimized at the UB3LYP level of theory with the 6-31+G(d,p) basis set for C, H, N, O and Cl and the LanL2DZ basis set for Cu. Conformations were then refined with the PBE method, 6-31G basis set, PCM with dielectric of 11 and "loose" convergence criteria. This 2-step method was used since exploratory work with the PBE/6-31G method were prone to GAUSSIAN errors, and the environment within the crystal would correspond to a high dielectric.

Table 1. The gross descriptions of the geometries used for XXVIII, with the constraints applied to the ab initio optimization prior to the searches and the full list of independent degrees of freedom used in the CrystalOptimizer refinement (torsion angles are given in red and bond angles are given in blue). The numbering used for this molecule in our work does not take advantage of the intramolecular symmetry, since DMACRYS only works with whole molecules. Hence the atomic numberings differ from the numbering scheme used in the main paper.

<table>
<tr><td>Gross conformation type</td><td>Constraints in geometry optimizations</td><td>CrystalOptimizer degrees of freedom</td></tr>
<tr><td colspan="3">Tetrahedral conformations</td></tr>
<tr><td>A
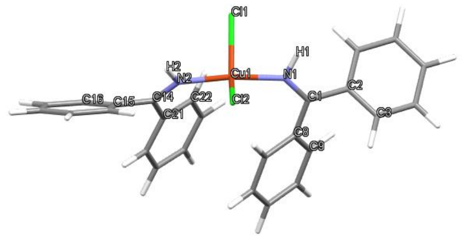
</td><td>None</td><td rowspan="2">Central geometry:
N1_Cu1_Cl1_Cl2, N2_Cu1_Cl1_Cl2, Cl2_Cu1_Cl1, N1_Cu1_Cl1, N2_Cu1_Cl1
Pyramidality at N atoms:
C1_N1_Cu1_H1, C14_N2_Cu1_H2, H1_N1_Cu1, H2_N2_Cu1, C1_N1_Cu1, C14_N2_Cu1
Major torsion angles:
H1_N1_Cu1_Cl2, C2_C1_N1_Cu1, C3_C2_C1_N1, C9_C8_C1_N1, H2_N2_Cu1_Cl1, C15_C14_N2_Cu1, C16_C15_C14_N2, C22_C21_C14_N2
Major bond angles
C2_C1_N1, C8_C1_N1, C15_C14_N2, C21_C14_N2</td></tr>
<tr><td>B
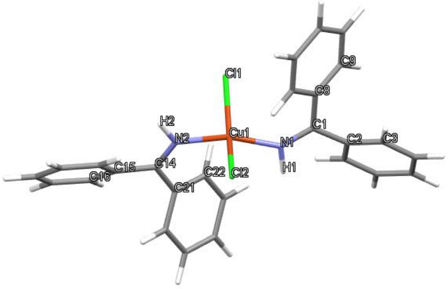
</td><td>None</td></tr>
<tr><td colspan="3">Square-planar-trans conformations</td></tr>
<tr><td>C
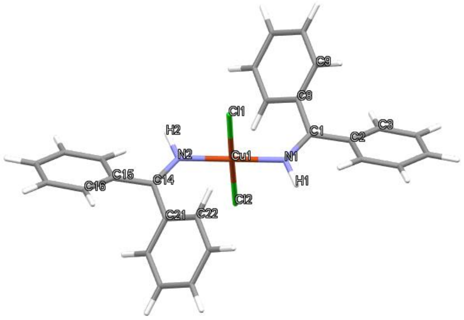
</td><td>Cl2_Cu1_Cl1=179.0
N1_Cu1_Cl1_Cl2=0.0
N2_Cu1_Cl1_Cl2=180.0</td><td rowspan="2">Central geometry:
Cl2_Cu1_N1_Cl1, N2_Cu1_Cl1_N1, N1_Cu1_Cl1, Cl2_Cu1_N1, N2_Cu1_Cl1
Pyramidality at N atoms:
C1_N1_Cu1_H1, C14_N2_Cu1_H2, H1_N1_Cu1, H2_N2_Cu1, C1_N1_Cu1, C14_N2_Cu1
Major torsion angles:
H1_N1_Cu1_Cl2, C2_C1_N1_Cu1, C3_C2_C1_N1, C9_C8_C1_N1, H2_N2_Cu1_Cl1, C15_C14_N2_Cu1, C16_C15_C14_N2, C22_C21_C14_N2
Major bond angles:
C2_C1_N1, C8_C1_N1, C15_C14_N2, C21_C14_N2</td></tr>
<tr><td>D
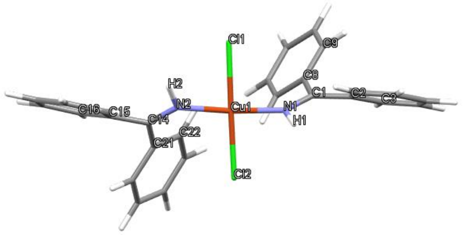
</td><td>Cl2_Cu1_Cl1=179.0
N1_Cu1_Cl1_Cl2=0.0
N2_Cu1_Cl1_Cl2=180.0</td></tr>
<tr><td colspan="3">Square-planar-cis conformations</td></tr>
<tr><td>F
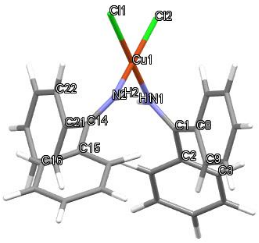
</td><td>N1_Cu1_Cl1=179.0
N1_Cu1_Cl1_Cl2=0.0
N2_Cu1_Cl1_Cl2=180.0</td><td>Central geometry:
N1_Cu1_Cl2_Cl1, N2_Cu1_Cl1_Cl2, Cl2_Cu1_Cl1, N1_Cu1_Cl2, N2_Cu1_Cl1
Pyramidality at N atoms:
C1_N1_Cu1_H1, C14_N2_Cu1_H2, H1_N1_Cu1, H2_N2_Cu1, C1_N1_Cu1, C14_N2_Cu1
Major torsion angles:
H1_N1_Cu1_Cl2, C2_C1_N1_Cu1, C3_C2_C1_N1, C9_C8_C1_N1, H2_N2_Cu1_Cl1, C15_C14_N2_Cu1, C16_C15_C14_N2, C22_C21_C14_N2
Major bond angles
C2_C1_N1, C8_C1_N1, C15_C14_N2, C21_C14_N2</td></tr>
</table>

3.2 Search methodology

For each of the conformations optimized in Table 1, point charges were calculated at the same PBE/6-31G(PCM ε=11) level of theory, with the HLYGAT charge fitting method (as Cu is not included in CHELPG, which we normally use).

Rigid molecule searches were carried out with CrystalPredictor, using these point charges and repulsion-dispersion parameters taken from the FIT potential for C, H, N, O and Cl and from the UFF potential for Cu^+. The selected space groups were P1, P-1, $P2_1$, C2, Pc, Cm, Cc, $P2_1/m$, C2/m, P2/c, $P2_1/c$, C2/c, $P222_1$, $P2_12_12$, $P2_12_12_1$, $C222_1$, $Pca2_1$, $Pmn2_1$, Pba2, $Pna2_1$, $Cmc2_1$, Aba2, Fdd2, Iba2, Pnna, Pccn, Pbcm, Pnnm, Pmmn, Pbcn, Pbca, Pnma, Cmcm, Cmca, Fddd, Ibam, $P4_1$, $P4_3$, $P4_12_12$, P4/n, $P4_2/n$, I4/m, $I4_1/a$, $P4_32_12$, $P-42_{1c}$, I-42d, $P3_1$, $P3_2$, R3, P-3, R-3, $P3_121$, $P3_221$, R3c, R-3c, $P6_1$, $P6_3$, $P6_3/m$, $P2_13$, Pa-3. Cell lengths in the range 10-80 Å and cell angles in the range 50-130° were generated as the starting points, and a minimum density of 300 kg m^{-3} was required before structures were passed to CrystalPredictor's internal minimizer (resulting structures can have any cell shape or size and any density). 20,000 structures were generated for each conformation.

3.3 Structure refinement

3.3.1 DMACRYS

DMACRYS was used to minimize the lattice energy of the crystal structures, using the repulsion-dispersion potential derived in Section 3.2, and the PBE/6-31G(PCM ε=11) derived multipoles.

3.3.2 CrystalOptimizer

The independent degrees of freedom optimized in CrystalOptimizer are given in Table 1. There needed to be some slight differences in the definition of the independent degrees of freedom between the tetrahedral and the square-planar conformations to prevent the latter relaxing to tetrahedral in the first step.

The intramolecular conformation and energy were evaluated at the PBE/6-31G(PCM ε=11) level of theory; the charge density was calculated at the same level of theory; the repulsion-dispersion potential was as described in Section 3.2. Optimizations were run for at least five outer minimizations, but full convergence was not sought (it was achieved in some cases).

3.4 Degree of confidence

We have never worked with metallorganics before, and the methodology would certainly not have worked if the structure had included any Cu···Cu bonding. However, the methods would be applicable if the Cu atom was buried and so the crystal structure was essentially packing the organic ligands. However, the conformational flexibility at the Cu centre was a concern. DMACRYS refinement requires an exp-6 repulsion-dispersion potential for each atomic type, and the FIT parameter set did not extend to Cu. However, since the copper atom is not involved in repulsive intermolecular interactions (if the Ψ_{mol} method is applicable), the lattice energies may not be particularly sensitive to the choice of parameters for copper centres. We found the Universal Force Field[12] contained parameters for Cu^+ with a tetrahedral geometry, and our model performed adequately for DMACRYS lattice energy minimization of 12 out of 14 related crystal structures. If the target crystal structure had any intermolecular close copper-copper contacts, or the molecule formed dimers with intermolecular copper-chlorine contacts, this target would have been outside the range of applicability of the Ψ_{mol} method.

The multipoles, which are a key part of the DMACRYS refinement, are probably of much greater importance than the choice of repulsion-dispersion potential. Initial work with related compounds showed that the most critical factor was the use of a polarizable continuum for the charge density calculations. A pseudopotential for copper (LanL2DZ) performed better than not using one (although the improvement was not worth the problems of implementing this in CrystalOptimizer), and the improvement with the PCM was even greater. Comparing different functionals (PBE, PBE0, M06, B3LYP, CAM-B3LYP, wB97xD) showed little difference, and PBE was selected as being as good as PBE0 while being significantly less costly.

We chose to run a series of rigid molecule searches (rather than a smaller number of flexible searches) as the conformational ranges available seemed to be quite narrow and the phenyl groups on the outside of the molecule

have no directional interactions. It turned out that the tetrahedral and square-planar-trans searches covered some of the same space, as structures were identified as duplicates between the regions.

3.5 Post-result analysis

The methodology was successful as the experimental structure was in our Submission 1 as XXVIII_dfCB11_CB11, with an $RMSD_{30}$ overlay of 0.459 Å. The test was treated as blind throughout, with no reference to the published experimental work.

4 XXIX – powder diffraction challenge

4.1 Conformational details

The molecule was deemed rigid, and was optimized at the PBE0/6-31G(d,p) level of theory.

4.2 Search methodology

The point charges were calculated for the optimized molecule with the PBE0/6-31G(d,p) level of theory. Repulsion-dispersion parameters were taken from the FIT potential. The selected space groups were P1, P-1, $P2_1$, C2, Pc, Cm, Cc, $P2_1/m$, C2/m, P2/c, $P2_1/c$, C2/c, $P222_1$, $P2_12_12$, $P2_12_12_1$, $C222_1$, $Pca2_1$, $Pmn2_1$, Pba2, $Pna2_1$, $Cmc2_1$, Aba2, Fdd2, Iba2, Pnna, Pccn, Pbcm, Pnnm, Pmmn, Pbcn, Pbca, Pnma, Cmcm, Cmca, Fddd, Ibam, $P4_1$, $P4_3$, $P4_12_12$, P4/n, $P4_2/n$, I4/m, $I4_1/a$, $P4_32_12$, $P\text{-}42_{1c}$, I-42d, $P3_1$, $P3_2$, R3, P-3, R-3, $P3_121$, $P3_221$, R3c, R-3c, $P6_1$, $P6_3$, $P6_3/m$, $P2_13$, Pa-3. Cell lengths in the range 3-40 Å and cell angles in the range 50-130° were generated as the starting points, and a minimum density of 300 kg m^{-3} was required before structures were passed to CrystalPredictor's internal minimizer (resulting structures can have any cell shape or size and any density). 1,000,000 structures were generated.

4.3 Structure refinement

4.3.1 CrystalOptimizer

Structures were refined with CrystalOptimizer, optimizing the methyl rotation, the carbonyl:phenyl single bond, the carbonyl:methoxy single bond, the two amine torsion angles (including calculating the gradients of the multipoles), and the C-O-C bond angle at the ester oxygen atom.

The PBE0/6-31G(d,p) level of theory was used to evaluate the intramolecular conformation and energy contribution. The charge density was also calculated at this level of theory, and distributed multipoles up to hexadecapole extracted. Repulsion-dispersion parameters were taken from the FIT potential.

4.3.2 Landscape reduction

The consideration of the dynamic motion of the molecules was considered essential as the PXRD pattern was nominally at 0 °C but the crystal melts at 24 °C. MD simulations were performed on 2093 Z'=1 rigid CrystalPredictor structures with the GAFF force field at 20 °C and 100 kPa in the nearest approximation to cubic supercells of side 40 Å that contained an integral number of unit cells. In the MD, 460 structures melted (118 in 300 ps NVT and 342 in 300 ps NPT) and we were left with clusters of up to 7 structures that were unique. It was notable that only 711 MD supercell averages could be represented by a smaller cell, even allowing disorder. Many structures had a larger Z' than the CSP_0 input structures which were all Z'=1. From each cluster, we selected the one with the lowest energy CSP_0 to give 1255 distinct Z'=1 structures for the Submission 1 landscape.

4.3.3 Powder pattern extraction and comparison

The provided powder pattern was digitized by Dr Rui Guo, and the background removed by Dr Kreso Bucar. This PXRD was compared with all search structures using AutoFIDEL (kindly provided by Jonas Nyman), and nothing was seen to be a good match, but some of the MD structures with higher Z' provided the best matches.

IDs	Score	Z'	Space Group
BXXIX_842	0.7328	1	$P2_12_12_1$
BXXIX_2330	0.7315	1	$P2_1/c$
BXXIX_1253	0.7268	1	$P2_1/c$
BXXIX_1575	0.7076	16	$P\bar{1}$
BXXIX_2461	0.7057	1	$I4_1/a$
BXXIX_11	0.6760	2	$P2_1/c$
BXXIX_1534	0.5893	2	$P\bar{1}$
BXXIX_14	0.5755	1	$P2_1/c$
BXXIX_9	0.5732	2	$P\bar{1}$
BXXIX_659	0.5383	1	$P2_1/c$

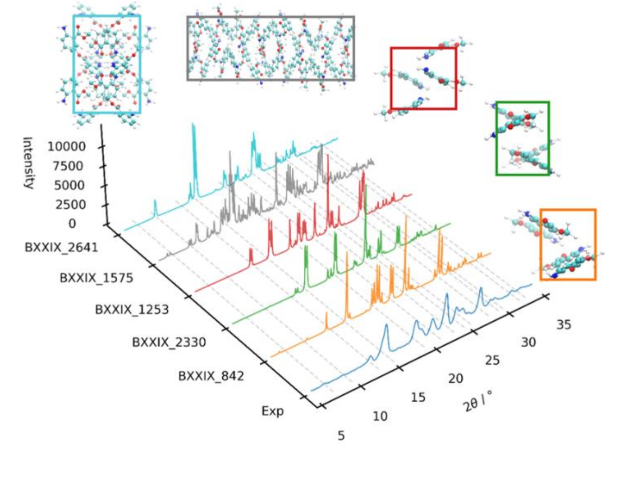

Figure 1. The structures submitted for giving the best match to the PXRD, with the score from Nyman's AutoFIDEL given and those with a score of >0.7 illustrated.

4.4 Degree of confidence

Although we knew that we had no matches to the powder pattern within the Z'=1 search, we didn't feel it was worthwhile running costly searches with higher Z', as the effect of temperature on the stable structures was as large as might be expected for a crystal so close to the melting point. The errors in either neglecting the effect of temperature or using the GAFF force field did not warrant the work involved in extending the calculations to Z'=2, let alone higher.

4.5 Post-result analysis

The experimental structure was a genuine Z'=3 structure, i.e. it could not be approximated by any Z'=1 structure even with MD. However, the experimental structure was only 0.07 kJ mol^{-1} more stable than our global minimum, and had some similarity to our submitted structures. For example, our BXXIX_3 (3.55 kJ mol^{-1} above the global minimum structures) matched 11 out of 15 molecules with an $RMSD_{11}$ of 0.168 Å with the experimental structure, showing that our lack of success was predominately due to not considering Z'=3.

5 XXX – stoichiometry challenge

5.1 Conformational details

The tetramethylpyrazine molecule was considered as rigid, as a CSP on just tetramethylpyrazine with flexible methyl groups was very similar to a rigid search.

A CSD survey was carried out for the cannabinol molecule, which showed that the hydroxyl group could take any angle and that the pentane tail was also very flexible. However, while the angle between the pentane and the aromatic ring was very flexible, all the other C_C_C_C torsions were close to 180° with slightly more than 10% around ±60° (Figure 2). Because of the cost from the high number of search variables in the 2:1 cannabinol:tetramethylpyrazine co-crystal search, it was decided to assume that the hydrocarbon tail was extended, ignoring the bent (±60°) conformations. In light of the experimental structures (Figure 2), this was a mistake.

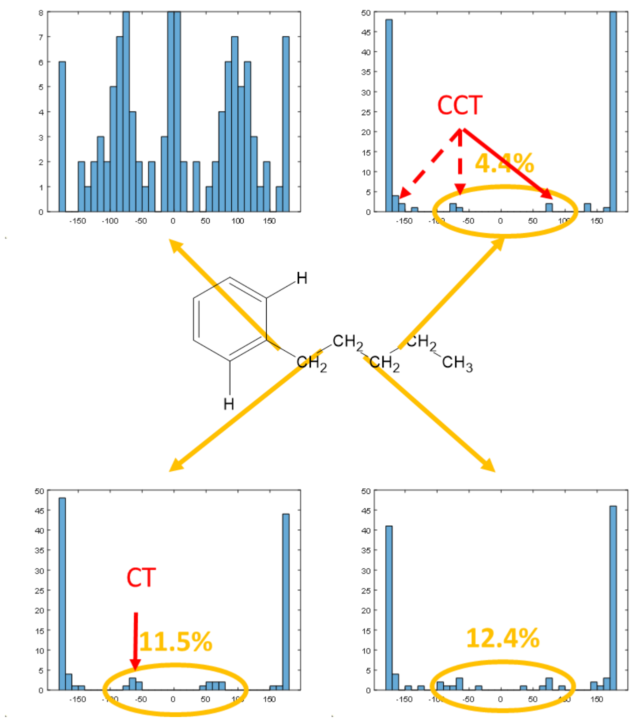

Figure 2. The CSD distributions of the conformation of the hydrocarbon chain in the central fragment, showing that the experimental structures of the 1:1 cannabinol:tetramehtylpyrazine (CT) and 2:1 cannabinol:tetramethylpyrazine (CCT) cocrystals adopted relatively rare conformations.

A database of Local Approximate Model (LAM) points was set up, defining the torsion angles, intramolecular energy, and point charge model for a series of conformations. The independent degrees of freedom were split into two torsion groups. For the first the tail was analyzed, and for the second the hydroxyl group was analyzed. For the tail, the angle between the pentacene and the aromatic ring was allowed to take any angle, but the next three single bonds were only allowed to take values between 120 and 240° (the methyl group was not considered at this stage, in line with the treatment of tetramethylpyrazine). The hydroxyl angle took almost any value, apart from where there was a steric clash with the other aromatic ring. See Table 2 for full details. At each conformation, the constrained optimization was carried out in GAUSSIAN with the PBE0 method and 6-31G(d,p) basis set.

Table 2. The range of values used for the construction of LAM points for cannabinol, and the final range of the angles within the CrystalPredictor search.

Angle	LAM start	LAM step	LAM end	Search range
Torsion group 1				
Between pentane and aromatic ring	200°	40°	520°	180-540°
Other pentane single bonds	140°	40°	220°	120-240°
Torsion group 2				
Hydroxyl angle	160°	30°	430°	145-445°

Point charges at each step were also calculated with the same computational method, and stored in the same databases.

5.2 Search methodology

Three separate searches with the desired tetramethylpyrazine to cannabinol stoichiometries were carried out in CrystalPredictor with the conformational description detailed in Section 5.1. Repulsion-dispersion parameters were taken from the FIT potential. The selected space groups were P1, P-1, $P2_1$, C2, Pc, Cm, Cc, $P2_1/m$, C2/m, P2/c, $P2_1/c$, C2/c, $P222_1$, $P2_12_12$, $P2_12_12_1$, $C222_1$, $Pca2_1$, $Pmn2_1$, Pba2, $Pna2_1$, $Cmc2_1$, Aba2, Fdd2, Iba2, Pnna, Pccn, Pbcm, Pnnm,

Pmmn, Pbcn, Pbca, Pnma, Cmcm, Cmca, Fddd, Ibam, $P4_1$, $P4_3$, $P4_12_12$, P4/n, $P4_2/n$, I4/m, $I4_1/a$, $P4_32_12$, $P\text{-}42_{1c}$, I-42d, $P3_1$, $P3_2$, R3, P-3, R-3, $P3_121$, $P3_221$, R3c, R-3c, $P6_1$, $P6_3$, $P6_3/m$, $P2_13$, Pa-3. Cell lengths in the range 3-40 Å (3-50 Å for the 1:2 and 2:1 stoichiometries) and cell angles in the range 50-130° were generated as the starting points, and a minimum density of 300 kg m^{-3} was required before structures were passed to CrystalPredictor's internal minimizer (resulting structures can have any cell shape or size and any density). 1,000,000 structures were generated in each stoichiometry.

5.3 Structure refinement

5.3.1 CrystalOptimizer

CrystalOptimizer refinement was carried out on the crystal structures from all three searches. The tetramethylpyrazine was kept rigid, and the five torsion angles treated as flexible at the search stage were optimized. The flexibility of the oxygen-containing ring was also optimized. The PBE0/6-31G(d,p) level of theory was used to evaluate the intramolecular conformation and energy contribution, as well as for the atomic multipoles. Repulsion-dispersion parameters were taken from the FIT potential.

5.4 Assessment of stoichiometry

Equivalent searches for the single components were also undertaken. The lowest energy structure in the tetramethylpyrazine search had the same cell parameters as the entry on the CSD without full atomic coordinates, and the second lowest energy crystal structure matched the fully characterized experimental crystal structure. In the case of cannabinol, the experimental structure (CANNOL) was not found in the search. The experimental crystal structure was optimized with the same computational model, and found to be significantly lower in energy than anything found in the search.

The cocrystallization energy of each of the cocrystal search structures was calculated according to Equation 3. This enabled comparison between the searches with different stoichiometry C_mT_n, with $E_{latt}(T)$ taken as the global minimum in the single component search for tetramethylpyrazine and $E_{latt}(C)$ taken as the lattice energy of CANNOL calculated with the same computational model.

Equation 3.

$$\Delta E_{cocrystal} = \frac{E_{latt}(C_mT_n) - nE_{latt}(T)}{m} - E_{latt}(C)$$

5.5 Degree of confidence

Running the single component search for cannabinol highlighted that the CrystalPredictor lattice energy minimization step with the point charge model for the electrostatics was not suitable for the cannabinol molecule. Indeed, running just this lattice energy minimization on the experimental crystal structure of CANNOL led to very poor reproduction. The hydroxyl angle is at the lowest energy when it is in the plane of the ring, but this configuration prohibits the formation of one of the hydrogen bonds in the Z'=2 structure of CANNOL. While CrystalPredictor generated starting points with the hydroxyl hydrogen atom out of the plane of the ring, virtually all minimized search structures had this hydrogen atom in the plane of the ring, and very few hydrogen bonded structures were seen. It seemed possible that this would cause problems for the cocrystal searches as well.

5.6 Post-result analysis

The experimental structures both had a bent aliphatic tail of cannabinol (Figure 2), and so could not have been generated in our search.

6 XXXI – agrochemical

6.1 Conformational details

All molecular geometry optimizations and charge density calculations (for both point charge calculations and distributed multipole analysis) were carried out with the PBE0/6-31G(d,p) method. The range of angles used for the database of LAM points is given in Table 3.

Table 3. The range of values used for the construction of LAM points for XXXI, and the final range of the angles within the CrystalPredictor search.

Angle	LAM start	LAM step	LAM end	Search range
C1_S1_C8_C10	70°	40°	310°	50 to 330°
C2_C1_S1_C8	40°	40°	320°	20 to 340°
C7_C2_C1_S1	75°	40°	115°	55 to 135°

6.2 Search methodology

A CrystalPredictor search was carried out with the torsion angle ranges as described in Table 3. Repulsion-dispersion parameters were taken from the FIT potential for C, H, N, O, with parameters for the sulfoxide group taken from Scheraga's work.[13] The selected space groups were P1, P-1, $P2_1$, C2, Pc, Cm, Cc, $P2_1/m$, C2/m, P2/c, $P2_1/c$, C2/c, $P222_1$, $P2_12_12$, $P2_12_12_1$, $C222_1$, $Pca2_1$, $Pmn2_1$, Pba2, $Pna2_1$, $Cmc2_1$, Aba2, Fdd2, Iba2, Pnna, Pccn, Pbcm, Pnnm, Pmmn, Pbcn, Pbca, Pnma, Cmcm, Cmca, Fddd, Ibam, $P4_1$, $P4_3$, $P4_12_12$, P4/n, $P4_2/n$, I4/m, $I4_1/a$, $P4_32_12$, $P-42_{1c}$, I-42d, $P3_1$, $P3_2$, R3, P-3, R-3, $P3_121$, $P3_221$, R3c, R-3c, $P6_1$, $P6_3$, $P6_3/m$, $P2_13$, Pa-3. Cell lengths in the range 3-40 Å and cell angles in the range 50-130° were generated as the starting points, and a minimum density of 300 kg m^{-3} was required before structures were passed to CrystalPredictor's internal minimizer (resulting structures can have any cell shape or size and any density). 1,000,000 structures were requested.

6.3 Structure refinement

Lattice energy minimization of all structures was carried out in CrystalOptimizer. The degrees of freedom optimized were the three torsion angles of the backbone, the torsion angles defining the sulfoxide oxygen atoms, one torsion angle describing each methyl rotation, the two bond angles defining the sulfoxide oxygen atoms and the two bond angles defining the fluorine atoms on the backbone.

Structures were compared to remove duplicates. Structures were considered duplicates if the energy difference was less than 10 kJ mol^{-1}, the difference in density was less than 0.1 g cm^{-3}, and an $RMSD_{15}$ overlay better than 0.5 Å was achieved.

6.4 Degree of confidence

This small organic molecule is the sort of size we are used to dealing with, and so should have been within our capabilities. The *ab initio* method chosen for the conformational scans and charge density was one that we have had success with in the past, and the repulsion-dispersion potential, combining the parameters for sulfoxide with the FIT potential, has also been successful, although we have noted difficulties with the F parameters in previous studies. The sensitivity to the choice of repulsion-dispersion potential and the use of a polarizable continuum model to model intermolecular polarization was such that we had only moderate confidence in the structures.

6.5 Post-result analysis

A_{min} was successfully included as BTXXXI_df1_1 with an $RMSD_{30}$ overlap of 0.315 Å, A_{maj} was successfully included as BTXXXI_df2_2 with an $RMSD_{30}$ overlap of 0.856 Å, and B was successfully included as BTXXXI_df239_239 with an $RMSD_{30}$ overlap of 0.865 Å.

7 XXXII – big pharmaceutical

The high degree of flexibility of this molecule meant that a flexible CrystalPredictor search was not possible. Hence, the CSP method developed by Luca Iuzzolino[14, 15] during his CCDC-sponsored PhD was used as the basis for our CSP.

7.1 Conformational details

The decision of which degrees of freedom to treat as flexible was guided by CSD analysis using the Conformer Generator, using the decision tree in Figure 3a (adapted from ref 14, Figure 3). The decisions reached for each torsion angle within the molecule are summarized in Table 4.

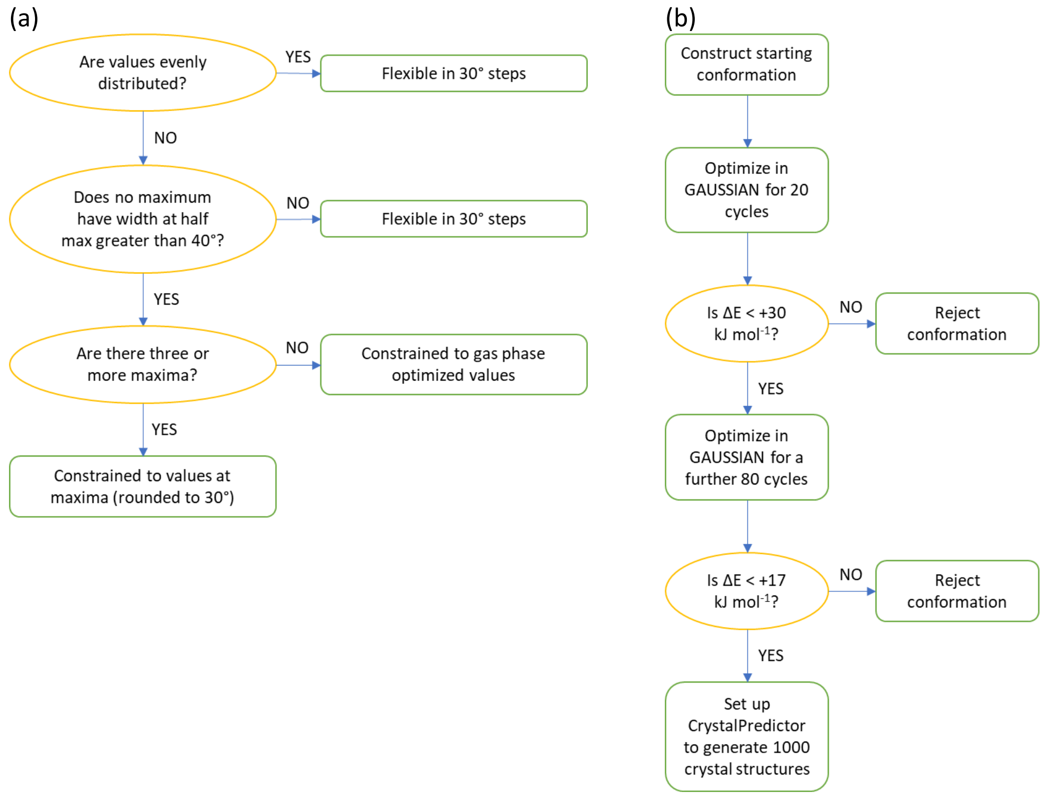

Figure 3. Decisions trees for (a) guiding the choice of flexible degrees of freedom and (b) generating conformations for CrystalPredictor searches.

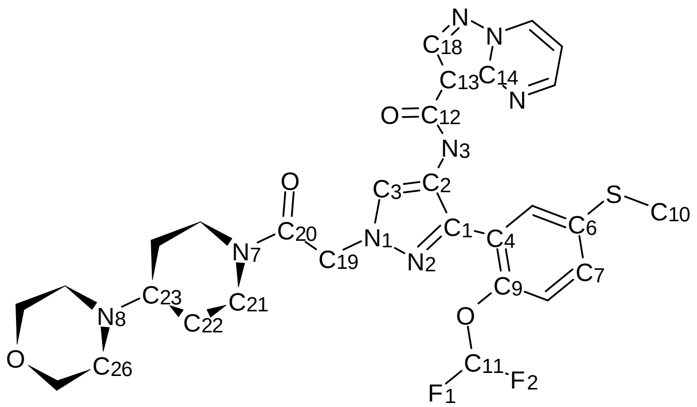

Figure 4. Numbering scheme for XXXII.

All conformational optimizations were carried out with the PBE0 method and the 6-31G(d,p) basis set.

Four distinct conformations with the gross conformation variables defined in Table 4 were constructed, labelled B, D, G and J. (A, C, F, and H had C29_C24_C23_N5 ~0°, but this was later found to not be relevant.) These four conformations were fully optimized. The angles listed in Table 4 under “Constraints” or “Gross conformations” were fixed at the values in these gas phase optimizations. All combinations of the angles listed under “Variables” in Table 4 were generated, and these variables were also fixed. A GAUSSIAN geometry optimization was run for 20 iterations. If there were steric clashes or the energy was above a threshold, the conformation was discarded. For non-converged conformations with energy below this threshold, optimization was continued for a further 80 steps. This is summarized in the decision tree in Figure 3b.

Table 4. The independent degrees of freedom considered for CrystalPredictor work for molecule XXXII.

Torsion angle	Possible values	Comment
Constraints		
C12_N3_C2_C1	Constrained to gas phase optimized (~180°)	
C13_C12_N3_C2	Constrained to gas phase optimized (~180°)	
C18_C13_C12_N3	Constrained to gas phase optimized (~180°)	0° was considered, but this was higher in energy since the intramolecular H-bond wasn't present, yet the polar hydrogen was still sterically prohibited for forming inter-molecular H-bonds
F1_C11_O3_C9	Constrained to gas phase optimized (~180°)	
H_C10_S_C6	Constrained to gas phase optimized (~60°)	
Gross conformations		
C21_N7_C20_C19	Constrained to gas phase optimized (~0°, ~180°)	~0°=B,D; ~180°=G,J
C10_S_C6_C7	Constrained to gas phase optimized (~0°, ~180°)	~0°=B,G; ~180°=D,J
Variables		
C20_C19_N1_C3	0°, 30°, 60°, 90°, 120°, 150°, 180°, 210°, 240°, 270°, 300°, 330°	
N7_C20_C19_N1	-60°, 60°, 180°	
C26_N8_C23_C22	-60°, 60°, 180°	
C9_C4_C1_C2	0°, 30°, 60°, 90°, 120°, 150°, 180°, 210°, 240°, 270°, 300°, 330°	
C11_O_C9_C4	-150°, -90°, 90°, 150°	

7.2 Search methodology

For all conformations within 17 kJ mol^{-1} of the lowest energy conformation, CrystalPredictor searches were carried out. The point charges were calculated for the original four fully-optimized conformations at the PBE0/6-31G(d,p) level of theory. Repulsion-dispersion parameters were taken from the FIT potential for C, H, N, O, F, with parameters for sulfur taken from the Halgren potential.[16] The selected space groups were P1, P-1, $P2_1$, C2, Pc, Cm, Cc, $P2_1/m$, C2/m, P2/c, $P2_1/c$, C2/c, $P222_1$, $P2_12_12$, $P2_12_12_1$, $C222_1$, $Pca2_1$, $Pmn2_1$, Pba2, $Pna2_1$, $Cmc2_1$, Aba2, Fdd2, Iba2, Pnna, Pccn, Pbcm, Pnnm, Pmmn, Pbcn, Pbca, Pnma, Cmcm, Cmca, Fddd, Ibam, $P4_1$, $P4_3$, $P4_12_12$, P4/n, $P4_2/n$, I4/m, $I4_1/a$, $P4_32_12$, $P-42_{1c}$, I-42d, $P3_1$, $P3_2$, R3, P-3, R-3, $P3_121$, $P3_221$, R3c, R-3c, $P6_1$, $P6_3$, $P6_3/m$, $P2_13$, Pa-3. Cell lengths in the range 5-60 Å and cell angles in the range 50-130° were generated as the starting points, and a minimum density of 300 kg m^{-3} was required before structures were passed to CrystalPredictor's internal minimizer (resulting structures can have any cell shape or size and any density). At least 1,000 structures were generated for each conformation.

The point charges were only calculated once in each conformational region, and used for all conformations in that region. This is an approximation, but a test on one conformation suggested that this would give a similar energy landscape if the search for each conformation could have been run to completion.

7.3 Structure refinement

7.3.1 DMACRYS

For each conformation, the multipoles were extracted from the PBE0/6-31G(d,p) charge density, and the structures were minimized with DMACRYS, keeping the molecular conformation rigid, and using the repulsion-dispersion parameters described in Section 7.2.

7.3.2 DFTB3-D3

DFTB3-D3 was used to optimize the conformation of the molecule within the packing environment.[15]

7.3.3 DMACRYS rerun

DMACRYS was then run again, with the same method as described in Section 7.3.1 using the multipoles for the exact conformation of the molecule following the DFTB3-D3 refinement. The intramolecular energy ascertained in the charge density calculation was used to determine the energy penalty of deforming the molecule from the gas phase

optimum conformation (ΔE_{intra}). (Hence, unlike CrystalOptimizer refinement, any errors in the conformation introduced by the DFTB3-D3 refinement of the crystal structure would contribute to ΔE_{intra}).

7.4 Degree of confidence

This molecule was highly flexible. While we tried to cover all the applicable conformational space, it was not possible to run searches for every conformation. The energy-based decision on which conformations to run searches on was very risky, and we were fully aware that the searches themselves were not complete as we only requested a handful of structures.

Our decision to use DFTB3-D3 to optimize the crystal structures, allowing some conformational adjustment, has been shown to be very effective for large flexible molecules.[15] The lack of reliability of the DFTB3-D3 energies meant that a single GAUSSIAN job was run for each structure to obtain the charge density for the multipoles and the intramolecular energy, with the intermolecular energy evaluated with DMACRYS. This final energy was shown to be a better approximation than the DFTB3-D3 energy[15] but still is not as accurate as CrystalOptimizer would have been, as CrystalOptimizer refines the conformation by constrained optimizations of the molecular structure.

7.5 Post-result analysis

Neither of the experimentally observed crystal structures were found within our searches. In the case of Form A, all conformational combinations which could have matched this molecule were fully optimized, but slightly too high in energy to be considered for searches. In the case of Form B, the two symmetry independent molecules differ in the angles at the thiomethoxy group, and so could not have come from a Z'=1 search. Furthermore, all conformational combinations which could have matched these conformations were also a few kJ mol^{-1} too high in energy to be used in searches.

8 XXXIII – organic salt

As salts are charged, there can be very strong electrostatic fields within the crystal structure which can polarize the charge distribution in a very localized manner that is poorly modelled by the Ψ_{mol} approach, even using a polarizable continuum model (PCM) with a dielectric constant of 11.

8.1 Conformational details

All molecular geometry optimizations and charge density calculations were carried out with the PBE0/6-31+G(d,p)(PCM ε=11) method.

The morpholinium cation was kept rigid throughout at the gas phase optimized conformation.

The independent degrees of freedom of the sulfamethoxazole anion were split into two torsion groups: the first one included the three torsion angles in the middle of the molecule, and the second one included one single torsion linking the amine group to the phenyl ring. For the amine group, the improper dihedral of the second hydrogen atom (H_N_C_H) was fixed at 135.845°, which was the value in the gas phase optimized conformation.

Table 5. The range of values used for the construction of LAM points for the sulfamethoxazole anion, and the final range of the angles within the CrystalPredictor search.

Angle	LAM start	LAM step	LAM end	Search range
Torsion group 1 "A"				
C_C_S_N	0°	40°	120°	-20 to 140°
C_S_N_C	40°	30°	100°	25 to 115°
S_N_C_C	-65°	40°	215°	-85 to 235°
Torsion group 1 "B"				
C_C_S_N	0°	40°	120°	-20 to 140°
C_S_N_C	145°	30°	325°	130 to 340°
S_N_C_C	-60°	40°	60°	-80 to 80°
Torsion group 1 "C"				
C_C_S_N	0°	40°	120°	-20 to 140°
C_S_N_C	135°	40°	215°	115 to 235°

S_N_C_C	160°	40°	200°	140 to 220°
Torsion group 2 “a”				
Amine hydrogen	-10°	40°	70°	-30 to 90°
Torsion group 2 “b”				
Amine hydrogen	170°	40°	250°	150 to 270°

Point charges were calculated for the molecule with the same *ab initio* method.

8.2 Search methodology

Six separate searches with one morpholinium cation and each of the combinations of the two torsion groups (see Table 5) of the sulfamethoxazole anion were carried out in CrystalPredictor. Repulsion-dispersion parameters were taken from the FIT potential, with parameters for the sulfoxide group taken from Scheraga’s work.[13] The selected space groups were P1, P-1, $P2_1$, C2, Pc, Cm, Cc, $P2_1/m$, C2/m, P2/c, $P2_1/c$, C2/c, $P222_1$, $P2_12_12$, $P2_12_12_1$, $C222_1$, $Pca2_1$, $Pmn2_1$, Pba2, $Pna2_1$, $Cmc2_1$, Aba2, Fdd2, Iba2, Pnna, Pccn, Pbcm, Pnnm, Pmmn, Pbcn, Pbca, Pnma, Cmcm, Cmca, Fddd, Ibam, $P4_1$, $P4_3$, $P4_12_12$, P4/n, $P4_2/n$, I4/m, $I4_1/a$, $P4_32_12$, $P\text{-}42_{1c}$, I-42d, $P3_1$, $P3_2$, R3, P-3, R-3, $P3_121$, $P3_221$, R3c, R-3c, $P6_1$, $P6_3$, $P6_3/m$, $P2_13$, Pa-3. Cell lengths in the range 3-40 Å and cell angles in the range 50-130° were generated as the starting points, and a minimum density of 300 kg m^{-3} was required before structures were passed to CrystalPredictor’s internal minimizer (resulting structures can have any cell shape or size and any density). 500,000 structures were requested in each of Aa, Ab, Ba and Bb, and 250,000 structures were requested in each of Ca and Cb.

CrystalPredictor’s internal clustering algorithm and an in-house Clustering algorithm were both run to remove duplicate structures.

8.3 Structure refinement

The 2,000 unique lowest energy crystal structures for each search were taken forward to the next stage. Lattice energy minimization of all structures was carried out in CrystalOptimizer. The morpholinium cation was kept rigid, and the degrees of freedom optimized for the sulfamethoxazole anion were the three angles of Torsion group 1 (see Table 5), the torsion angles defining the sulfoxide oxygen atoms, the torsion angles defining the amine hydrogen atoms, one torsion angle describing the methyl rotation, the two bond angles within the linking group, the two bond angles defining the sulfoxide oxygen atoms and the two bond angles defining the amine hydrogen atoms. With the exception of the torsion angle to define the methyl rotation, gradients of the multipoles were also calculated for all degrees of freedom; this increases computational cost at each step but stops the optimization taking the wrong trajectory. Past experience led us to include torsion angles within fairly rigid (e.g. π-conjugated) rings at points adjacent to large functional groups. Four such degrees of freedom were included, although amine and methyl groups are not usually considered large.

Only two iterations of CrystalOptimizer were run, so only a handful of structures were fully optimized. However, comparing the energy ranking after two CrystalOptimizer steps and that after full convergence (carried out for a few structures) found that most of the change in energy was from applying the different computational model rather than actual optimization of the structures, and this was deemed sufficient to get the best energy ranking in the available time.

Structures were compared to remove duplicates. Structures were considered duplicates if an $RMSD_{30}$ overlay of < 0.3 Å was achieved.

8.4 Degree of confidence

Charged species in CSP give rise to a different set of challenges from neutral molecules. The *ab initio* calculations on them are not representative, since the *in vacuo* calculations have to contain just one ion. To deal with this, we used a basis set with diffuse functions and a polarizable continuum for all our *ab initio* work.

The conformational space covered and the extent of the search both appear complete, so we have high confidence in them. We also feel that the energy model for the structure refinement was a good one (with the caveats from the previous paragraph), even though we didn’t have time to fully refine all structures.

8.5 Post-result analysis

This search was successful, in that both experimental crystal structures were generated, low in energy. Form A matched XXXIII_dfBa282_Ba282 with $RMSD_{30}$ = 0.359 Å and Form B matched XXXIII_dfAa686_Aa686 with $RMSD_{30}$ = 0.269 Å.

9 References

(1) Sugden, I. J.; Adjiman, C. S.; Pantelides, C. C. Accurate and efficient representation of intramolecular energy in ab initio generation of crystal structures. II. Smoothed intramolecular potentials. *Acta Crystallographica Section B-Structural Science Crystal Engineering and Materials* **2019**, *75*, 423-433, Article. DOI: 10.1107/S2052520619005778.

(2) Holden, J. R.; Du, Z. Y.; Ammon, H. L. Prediction of Possible Crystal-Structures For C-, H-, N-, O- and F-Containing Organic Compounds. *Journal of Computational Chemistry* **1993**, *14* (4), 422-437.

(3) Price, S. L.; Leslie, M.; Welch, G. W. A.; Habgood, M.; Price, L. S.; Karamertzanis, P. G.; Day, G. M. Modelling Organic Crystal Structures using Distributed Multipole and Polarizability-Based Model Intermolecular Potentials. *Physical Chemistry Chemical Physics* **2010**, *12* (30), 8478-8490.

(4) Kazantsev, A. V.; Karamertzanis, P. G.; Adjiman, C. S.; Pantelides, C. C. CrystalOptimizer. An efficient Algorithm for Lattice Energy Minimisation of Organic Crystal using Isolated-Molecule Quantum Mechanical Calculations. In *Molecular System Engineering*, Adjiman, C. S., Galindo, A. Eds.; Process Systems Engineering, Vol. 6; WILEY-VCH Verlag GmbH & Co., 2010; pp 1-42.

(5) *Gaussian 09, Revision D.01*; 2009.

(6) Stone, A. J. GDMA: A Program for Performing Distributed Multipole Analysis of Wave Functions Calculated Using the Gaussian Program System. *GDMA2.2* **2010**.

(7) Williams, D. E.; Cox, S. R. Nonbonded Potentials For Azahydrocarbons: the Importance of the Coulombic Interaction. *Acta Crystallographica Section B - Structural Science* **1984**, *40* (8), 404-417.

(8) Coombes, D. S.; Price, S. L.; Willock, D. J.; Leslie, M. Role of Electrostatic Interactions in Determining the Crystal Structures of Polar Organic Molecules. A Distributed Multipole Study. *Journal of Physical Chemistry* **1996**, *100* (18), 7352-7360.

(9) Hsu, L. Y.; Williams, D. E. Intermolecular Potential-Function Models for Crystalline Perchlorohydrocarbons. *Acta Crystallographica Section A - Crystal Physics, Diffraction, Theoretical and General Crystallography* **1980**, *36* (MAR), 277-281.

(10) Williams, D. E.; Houpt, D. J. Fluorine Nonbonded Potential Parameters Derived From Crystalline Perfluorocarbons. *Acta Crystallographica Section B - Structural Science* **1986**, *42* (JUN), 286-295.

(11) Day, G. M.; Motherwell, W. D. S.; Ammon, H. L.; Boerrigter, S. X. M.; Della Valle, R. G.; Venuti, E.; Dzyabchenko, A.; Dunitz, J. D.; Schweizer, B.; van Eijck, B. P.; et al. A third blind test of crystal structure prediction. *Acta Crystallographica Section B - Structural Science* **2005**, *61* (5), 511-527.

(12) Rappe, A. K.; Casewit, C. J.; Colwell, K. S.; Goddard, W. A.; Skiff, W. M. UFF, a Full Periodic-Table Force-Field For Molecular Mechanics and Molecular-Dynamics Simulations. *Journal of the American Chemical Society* **1992**, *114* (25), 10024-10035.

(13) Motherwell, W. D. S.; Ammon, H. L.; Dunitz, J. D.; Dzyabchenko, A.; Erk, P.; Gavezzotti, A.; Hofmann, D. W. M.; Leusen, F. J. J.; Lommerse, J. P. M.; Mooij, W. T. M.; et al. Crystal structure prediction of small organic molecules: a second blind test. *Acta Crystallographica Section B - Structural Science* **2002**, *58*, 647-661.

(14) Iuzzolino, L.; Reilly, A. M.; McCabe, P.; Price, S. L. Use of Crystal Structure Informatics for Defining the Conformational Space Needed for Predicting Crystal Structures of Pharmaceutical Molecules. *Journal of Chemical Theory and Computation* **2017**, *13* (10), 5163-5171. DOI: 10.1021/acs.jctc.7b00623.

(15) Iuzzolino, L.; McCabe, P.; Price, S. L.; Brandenburg, J. G. Crystal structure prediction of flexible pharmaceutical-like molecules: density functional tight-binding as an intermediate optimisation method and for free energy estimation. *Faraday Discussions* **2018**, *211*, 275-296, 10.1039/C8FD00010G. DOI: 10.1039/C8FD00010G.

(16) Halgren, T. A. Representation of Vanderwaals (Vdw) Interactions in Molecular Mechanics Force-Fields - Potential Form, Combination Rules, and Vdw Parameters. *Journal of the American Chemical Society* **1992**, *114* (20), 7827-7843.

19. Group 25

Supporting Information: The Seventh CCDC Blind Test of Crystal Structure Prediction Methods

Cheng Shang, Zhi-Pan Liu

Collaborative Innovation Center of Chemistry for Energy Material, Shanghai Key Laboratory of Molecular Catalysis and Innovative Materials, Key Laboratory of Computational Physical Science (Ministry of Education), Department of Chemistry, Fudan University, Shanghai 200433, China

Methodology:

For each molecular crystal target, the structure candidates are generated by the rigid-SSW method as implemented in LASP software[1, 2], where the potential energy surface (PES) of each target is represented by the generalized amber force field (GAFF) [3]. The rigid-SSW method is an extension of the original stochastic surface walking (SSW) method by applying rigidbody constrains to atomic groups of molecules to reduce the degrees of freedom during configurational change. The original SSW method[4, 5] is a general-purpose PES exploration method that was firstly developed in 2013. The method has an automated PES climbing mechanism to manipulate an image on the PES from a minimum to a high energy configuration by adding a serial of bias potential along a random mode direction. The high energy image as a local minimum on the bias-potential-modified PES is then optimized to a new minimum on the unbiased PES. A structure selection module based on Metropolis Monte Carlo algorithm is applied to decide whether to accept the new minimum. By repeating the procedure, one can explore the whole PES and identify new structures unbiasedly. In the original SSW method for the crystal structure prediction, e.g. n atoms in a unit cell, the $3n$-dimensional atomic mode is combined with a 9-dimensional lattice mode perturbing the lattice parameters, where the translation and rotation of the entire structure is removed. By applying rigid constraints, the bond and angles within a molecule are fixed. Then only the translational and rotational movements of the entire molecule, and internal rotational degrees of freedom within molecules are considered during structure exploration with no further symmetry constrains. For each target, we performed 50 parallel rigid-SSW runs with different randomly generated starting configurations. A maximum number of 10^5 minima are visited for each run. The number of molecules that considered in the unit cell is 4 for targets XXVII to XXXII and 8 (1:1) for XXXIII.

All the minima of non-P1 space group are collected after rigid-SSW exploration. A Steinhardt-type order parameter is used for clustering the candidates. The distinct candidates are then optimized by an active-learning neural network potential model[6-8] as implemented in LASP software to select the best 1500 minima, where the best 300 minima are finally refined by using DFT computation with the vdW-DF2 functional[9]

as implemented in Vienna ab initio simulation package (VASP) software[10, 11]. A plane wave basis set cutoff energy of 600 eV was used, and Brillouin zone integrations were performed on a Monkhorst-Pack grid with a k-point spacing of approximately 0.05 $Å^{-1}$. All the optimization is performed by Quasi-Newton BFGS method.

1 Pei-Lin Kang, Cheng Shang and Zhipan Liu, *Chin. J. Chem. Phys.*, **2021**, *34*, 583

2 Si-Da Huang, Cheng Shang, Pei-Lin Kang, Xiao-Jie Zhang and Zhi-Pan Liu, *WIREs Comput Mol Sci*, **2019**, *9*, e1415

3 J. Wang, R. M. Wolf, J. W. Caldwell, P. A. Kollman and D. A. Case, *J. Comput. Chem.*, **2004**, *25*, 1157-1174

4 C. Shang and Z. P. Liu, *Journal of Chemical Theory and Computation*, **2013**, *9*, 1838-1845

5 X. J. Zhang, C. Shang and Z. P. Liu, *J Chem Theory Comput*, **2013**, *9*, 3252-60

6 Cheng Shang and Zhi-Pan Liu, Chapter 14 - Constructing machine learning potentials with active learning, Quantum Chemistry in the Age of Machine Learning, 2023, Elsevier,

7 Si-Da Huang, Cheng Shang, Pei-Lin Kang and Zhi-Pan Liu, *Chemical Science*, **2018**, *9*, 8644-8655

8 Si-Da Huang, Cheng Shang, Xiao-Jie Zhang and Zhi-Pan Liu, *Chemical Science*, **2017**, *8*, 6327-6337

9 Kyuho Lee, Éamonn D. Murray, Lingzhu Kong, Bengt I. Lundqvist and David C. Langreth, *Phys. Rev. B*, **2010**, *82*, 081101

10 G. Kresse and J. Furthmüller, *comp. mater. sci.*, **1996**, *6*, 15-50

11 G. Kresse and J. Furthmüller, *Phys. Rev. B*, **1996**, *54*, 11169-11186

20. Groups 26 and 27

Supplementary Information
Groups 26 & 27: Stage 1

Rajni M. Bhardwaj, Eric J. Chan, Richard Hong, Ommair Ishaque, Aling Jing, John W. Melkumov, Rahul Nikhar, Rafał Podeszwa, Atta Rehman, Jutta Rogal, Hongxing Song, Krzysztof Szalewicz, Mark E. Tuckerman, and Leslie Vogt-Maranto

(Dated: May 23, 2024)

I. METHODOLOGY

All the monomers in the 7th blind test (7BT) of crystal structure predictions (CSPs) included flexible monomers, i.e., monomers with soft degrees of freedom. CSPs for such systems require in general the knowledge of both the intermonomer and intramonomer force fields (FFs) [also called potential energy surfaces (PESs)]. Efficient strategies, available in the autoPES code, have been developed by the University of Delaware group for generation of rigid-monomer intermonomer FFs fully from first principles [1–3]. Such aiFFs, where 'ai' stands for *ab initio*, can be generated for dimers of monomers of the size appearing in 7BT. Also, an efficient and accurate CSP method, based on such aiFFs and called aiFF@CSPs [4], was under advanced development during 7BT. The most recent version of the autoPES codes does allow the development of full-dimensional FFs (including both intermonomer and intramonomer components). However, this method would be too expensive to apply to the 7BT molecules mainly due to the dimensionality curse: all rigid-monomer PESs are at the most 6-dimensional (6D), whereas flexible-monomer intramonomer FFs are $3N-6$-dimensional for dimers with N atoms. Thus, to perform 7BT CSPs, we used rigid-monomer aiFFs and to account for monomer flexibility, we used two strategies. For monomers with limited flexibility, we assumed that the geometry of a monomer in a crystal corresponds to one of the local minima on the electronic potential energy surface of the gas-phase monomer. Thus, such monomer is one of the conformational isomers, or conformers, of this molecule. For more flexible monomers, we used empirical intramonomer FFs. Although we did realize that reliability of these FFs for monomers of the type included in 7BT is limited, there were no other options within the time limits of the stage 1 of 7BT. The work on individual 7BT targets was performed by subsets of the authors, see Table I.

TABLE I: Summary of authors (represented by their initials) who worked on each system: a check-mark signifies that an author contributed in the submission for that system.

System	RMB	EJC	RH	OI	AJ	JWM	RN	RP	AR	JR	HS	KS	MET	LVM
XXVII					✓		✓	✓				✓		
XXVIII							✓	✓	✓			✓		
XXIX		✓					✓	✓		✓	✓	✓	✓	✓
XXX		✓					✓	✓		✓	✓	✓	✓	✓
XXXI				✓			✓	✓		✓		✓		✓
XXXII		✓				✓	✓	✓	✓	✓		✓	✓	✓
XXXIII	✓		✓				✓	✓				✓	✓	✓

The general protocol of our CSPs in 7BT included searches for approximate geometries of

conformers followed by optimizations of conformers' geometries, generation of intermolecular aiFFs, and using the conformer(s), aiFFs, and possibly intramolecular empirical FFs (for flexible-monomer searches) in CSPs. A summary of methods used for individual targets is presented in Table II.

The conformer searches started with building 3D structures from the 2D structural formulas provided by the Cambridge Crystallographic Data Centre (CCDC). The 3D structure was optimized using an empirical FF to achieve the initial approximation to bond lengths and angles. Next, a number of plausible monomer conformations were generated by varying the soft degrees of freedom; specific details for each system are available in their respective sections. In this step, programs such as Avogadro [5], Baloon [6], conformer generator [7] (a part of Mercury [8]), Conformator [9], and PLUMED [10, 11] were used. The structures obtained in this way were then optimized using *ab initio* methods (except for one case) and ranked energetically. In most cases, we used various density-functional theory (DFT) variants supplemented by dispersion corrections (DFT+D). Depending on the system, either the equilibrium monomer, i.e., the configuration of the global minimum, and/or higher-energy conformers, corresponding to local minima within a few kcal/mol of the global minimum, was/were selected to generate aiFFs. These conformers were also utilized in CSP searches performed with rigid monomers (for targets XXVII, XXVIII, XXIX, and XXXI), using in some cases several conformers and mixtures of conformers. The remaining targets (XXX, XXXII, and XXXIII) have monomers that are too flexible for the conformer-based approach to work (although for target XXX we applied rigid-monomer CSPs as well). For these targets, we performed flexible-monomer CSPs using empirical intramolecular FFs such as OPLS [12] or GAFF [13]. Once a set of polymorphs was generated with a flexible-monomer FF, the monomer energies were computed with an *ab initio* method and used to determine monomer-deformation penalties in lattice energies, leading to our final rankings. In flexible-monomer simulations, we used intermolecular aiFFs developed using rigid monomers (possibly several conformers simultaneously), which is possible due to the functional form of our aiFFs, as discussed below.

Intermolecular aiFFs were developed using the autoPES program [1, 2] which generates sets of dimer configurations (grid points), submits all required *ab initio* calculations, extracts the results from the outputs, and fits these results with analytic functions. For the *ab initio* calculations, either perturbation theory or the supermolecular method was applied. For four systems, we used symmetry-adapted perturbation theory (SAPT) [14] based on a DFT description of the monomers, SAPT(DFT) [15], implemented in the SAPT2020 [3, 16] package, using ORCA-4.2.1 [17, 18] for monomer DFT calculations. The intermolecular energies included also the so-called $\delta E_{\text{int}}^{\text{HF}}$ correction which is the difference between the supermolecular Hartree-Fock (HF) interaction energy and the sum of SAPT terms that are included in the former energy (in this case, SAPT based on HF description of the monomers, SAPT(HF), was used). The aug-cc-pVDZ basis sets [19, 20] were used in these calculations unless noted otherwise. They were applied in the so-called monomer-centered plus basis set form (MC^+BS) [21] where a given monomer basis set includes all functions cen-

TABLE II: Summary of methodologies used. See text for acronyms. All CSPs used the UPACK package. All intermonomer FFs included SAPT(DFT) asymptotics.

System	monomer geometry	intermonomer FF	intramonomer FF	CSP type
XXVII	Avogadro/DFT+D	DFT+D	none	rigid monomer, equilibrium conformer
XXVIII	Baloon/DFT+D	DFT+D	none	rigid monomer, equilibrium conformer
XXIX	Mercury/DFT	SAPT(DFT)/CCfC	none	rigid monomer, 2 conformers
XXX	Mercury/DFT+D	SAPT(DFT)/CCfC	OPLS	rigid/flexible monomer
XXXI	Conformator DFT+D	SAPT(DFT)	none	rigid monomer, 10 conformers
XXXII	Avogadro/PLUMED	DFT+D	GAFF	flexible monomer
XXXIII	Mercury/DFT+D	SAPT(DFT)	GAFF	flexible monomer

tered on this monomer plus the isotropic part of the basis set of the interacting partner (farbond), following the default approach in the autoPES software [1, 2] for developing potential energy surfaces. In some cases, MC$^+$BSs included also a set of midbond functions located half-way between monomers: in all cases it was the 3s3p2d2f set that is the default of autoPES.

The supermolecular approach was used for three systems. The counterpoise-corrected intermolecular energies were calculated using the PBE0 [22, 23] DFT functional plus the D3 dispersion correction [24]. The use of the counterpoise method implies that monomer energies were computed in the dimer-center basis set (DCBS). Midbond functions were not used in supermolecular calculations. Unless noted otherwise, the aug-cc-pVDZ basis set [19, 20] was used.

Using the autoPES program [1, 2], the intermolecular interaction energies were fitted to the following functional form:

$$V = V_{\text{elst}} + V_{\text{exp}} + V^{(2)}_{\text{asymp}} = \sum_{a\in A, b\in B} u_{ab}(r_{ab}) = \sum_{a\in A, b\in B} \left[u_{\text{elst},ab}(r_{ab}) + u_{\text{exp},ab}(r_{ab}) + u^{(2)}_{\text{asymp},ab}(r_{ab}) \right] \quad (1)$$

where a (b) goes over the set of atoms in monomer A (B) and r_{ab} is the distance between atoms a

and b. The atom-atom functions are of the form:

$$\begin{aligned}
u_{\mathrm{elst},ab}(r_{ab}) =& f_1(\delta_1^{ab}, r_{ab})\frac{q_a q_b}{r_{ab}}, \\
u_{\mathrm{exp},ab}(r_{ab}) =& \left[1 + \sum_{i=1}^{k} a_i^{ab}(r_{ab})^i\right] e^{\alpha^{ab}-\beta^{ab}r_{ab}} + \frac{A_{12}^{ab}}{(r_{ab})^{12}}, \\
u^{(2)}_{\mathrm{asymp},ab}(r_{ab}) =& -\sum_{n=6,8} f_n(\delta_n^{ab}, r_{ab})\frac{C_n^{ab}}{(r_{ab})^n},
\end{aligned} \tag{2}$$

where q_a, q_b, a_i^{ab}, α^{ab}, β^{ab}, A_{12}^{ab}, C_n^{ab}, and δ_n^{ab} are adjustable parameters and f_n are the Tang-Toennies damping functions [25]

$$f_n(\delta, r) = 1 - e^{-\delta r}\sum_{m=0}^{n}\frac{(\delta r)^m}{m!}. \tag{3}$$

Unless stated otherwise, the fit functional form used the second order polynomial and included only the lowest induction+dispersion term, C_6^{ab}/r_{ab}^6. This term and the Coulomb term were damped in all fits. We first fitted the charges q_a, q_b, and the C_n^{ab} coefficients to a set of interaction energies calculated using asymptotic theory consistent with SAPT(DFT), as implemented in SAPT2020 [3, 16]. These parameters were kept fixed during the subsequent stages of the fitting process. The *ab intio* distributed asymptotic (aiDA) expansion of Refs. 26 and 27 was employed since it is currently computationally more efficient than the center-of-mass based expansion [1]. In the next stage, parameters a_i^{ab}, α^{ab}, β^{ab}, A_{12}^{ab}, and δ_n^{ab} were fitted to dimer *ab initio* interaction energies, E_{int}, computed on a set of grid points encompasing intermediate- and short-range intermolecular separations (collectively called close-range grid points). If the interaction energies were computed using SAPT(DFT), the damping parameters δ_1^{ab} were fitted separately to the electrostatic energies and the δ_6^{ab} parameters to the sum of induction and dispersion energies plus their exchange counterparts. For all supermolecular calculations, these parameters were fitted together with the other close-range parameters. The process of generating grid points is iterative and guided by the values of interaction energies in different regions of configuration space. In the first aiFF development step, the OPLS-AA [12] FF was used to get the first approximation of the PES needed for this guidance. Subsequently, intermediate versions of the aiFF were used. In the fitting process, the grid-points with negative interaction energies, E_{int}, had larger weights than points with positive E_{int} (see Refs. 1 and 2 for details of the weighting function). For the convergence test, the total set of grid points was divided into a test (30%) and a training set (70%). The fit was considered converged when the root mean square error (RMSE) of the test data was less than 1.2×RMSE of the training data, and the fit did not contain "holes", i.e., nonphysical low-energy regions at small intermonomer separations. If holes were detected, an iterative procedure was performed consisting in adding grid points in the regions of holes and refitting the PES.

The aiFFs were developed using only rigid monomers in the training set. In some cases, several conformers were included. The fit form was always the one defined by Eqs. (1)–(3), which does not include any information about the monomer geometry. Consequently, grid points computed for monomers with several geometries can be used in the fitting process. More importantly, this allows us to compute interaction energies from the fit for arbitrary monomer geometries, resulting in a "flexibilized" aiFF. Such flexibilized aiFFs were used in CSPs with monomer conformations different from the global gas-phase minimum, and, in particular, in CSPs with flexible monomers.

One should mention here that autoPES [2] allows the development of flexible-monomer PESs including only selected monomer's degrees of freedom. For the 7BT targets containing up to 3 soft degrees of freedom per monomer, the corresponding surfaces would have contained up to 12 degrees of freedom in total. These PESs would allow flexible-monomer CSPs entirely from first principles. However, the required computational resources were too large to try such an approach within the time constraints of the 7BT.

For certain targets, the first generation aiFFs were improved by incorporating some nearest neighbor dimer configurations extracted from 5×5×5 supercells of the top ranking polymorphs obtained in CSPs with the initial version of this aiFF into the training set, followed by a refit. This so-called clusters cut from crystals (CCfC) approach was developed in Ref. 28. Although the raw RMSE improvements of the refitted aiFFs were generally small, the addition of dimer configurations from the top-ranking polymorphs improves the aiFFs in the regions relevant to crystals.

For CSPs, we used a locally modified version of UPACK [29]. This modification [4] extends the set of functional forms for the site-site functions available in UPACK [the Buckingham plus charges (exp-6+q) or Lennard-Jones plus charges (LJ+q or 12-6-1) potentials] to the more involved forms represented by Eq. (2). In the rigid-monomer UPACK CSP protocol, 20 polymorphs per space group are initially generated in an unconstrained way. The largest density polymorph encountered is used to constrain the coordinate space in the subsequent step (so-called packing step) that generates a large number of crystal structures applying a quasi-random approach: 5000 structures per space group are generated by default. That is, a monomer in the fixed geometry of one of its conformers is placed in random positions and orientations within the space group constraints. We always searched in at least the following 13 space groups, which are the default in UPACK (unless noted otherwise): $P1$, $P\bar{1}$, $P2_1$, $C2$, Pc, Cc, $P2_1/c$, $C2/c$, $P2_12_12_1$, $Pca2_1$, $Pna2_1$, $Pbcn$, and $Pbca$ with the number of molecules in the asymmetric unit $Z' = 1$ in most cases ('pack12' program of UPACK). To increase sampling in space group 14, searches were sometimes performed in the non-standard settings $P2_1/n$ and $P2_1/a$ using the same random seed as for other space groups rather than starting searches with multiple seeds. The polymorphs generated were first roughly optimized using an empirical FF with generic LJ parameters (taken from OPLS-AA [12] in most cases) and SAPT charges or the restrained electrostatic potential (RESP) charges [30] for

the Coulomb part of the 12-6-1 potential. Duplicates (identical crystals to within some threshold) generated in this process were removed by subjecting the resulting list to clustering [31] ('dist' program of UPACK). Subsequently, the polymorphs from the reduced set were subjected to tight optimizations using aiFFs ('pack3' program of UPACK), which was followed by clustering to arrive at the final CSP lists.

In addition to UPACK structure generation codes, we used the Extended Variables Coupled to Crystal Polymorph Modified Replica Exchange (EVCCPMRE) method [32]. Unlike a conventional replica exchange, the EVCCPMRE approach performs exchanges between replicas of EV's, including molecular centers, orientations, and torsional angles, that are harmonically coupled to a crystal polymorph generator. For the latter, the EVCCPMRE codes used a modified version of UPACK. Each EV reference system propagates via both EV exchanges between temperature baths and canonical sampling moves using Metropolis-type Monte Carlo (MC) updates. The reference system framework was also implemented with a history-dependent biasing to further enhance sampling. A more detailed description of the method including a case study of the ensemble properties will be provided in a forthcoming publication [32].

All the targets in 7BT include monomers with soft degrees of freedom (highly-flexible monomers) and should be investigated using flexible-monomer FFs. As already stated, we used two strategies to deal with monomer flexibility. For targets with the largest number of soft degrees of freedom, we did perform flexible-monomer CSPs using flexibilized intermonomer aiFFs and generic empirical intramonomer FFs. We used UPACK in these calculations in a similar way as in the rigid-monomer case, except, of course, that the intramolecular degrees of freedom are probed both in the packing and optimization steps. For less flexible monomers, we decided that the computationally more efficient strategy based on rigid-monomer CSPs for seclected conformers should work, i.e., we selected k consecutive lowest-energy conformers and performed k independent rigid-monomer CSPs using flexibilized intermonomer aiFFs. Results of all such CSPs were used to form a list of structures ordered by lattice energies which included monomer-deformation penalties.

The successful predictions of the experimental crystal were gauged by the root-mean-square deviation (RMSD) between the positions of atoms in a predicted polymorph and those in the experimental one, always neglecting hydrogen atoms. RMSD involving n monomers, in most cases $n = 30$, is indicated by RMSD$_n$. The RMSDs are calculated after overlapping the two crystals to minimize the RMSD using the Mercury software [8]. We assumed tolerances on differences of positions and angles of individual atoms in the two polymorphs up to 35% and 35°, respectively, unless stated otherwise.

For all targets, we have performed an analysis of our procedures, described in the "post-submission" sections. In all cases, we used the same FFs that had been used to generate the submitted structures in stage 1.

II. TARGET XXVII

Target XXVII is a large molecule containing 100 atoms. It consists of a rigid polyaromatic pentacene core with two triisopropylsilane (TIPS) groups connected to it via a nearly linear −C≡C− link. The participants were requested to submit 1500 polymorphs without any ranking.

A. Conformer search and optimization

We obtained an initial 3D monomer geometry with the Avogadro software [5] by building a very approximate 3D model from the CCDC skeletal 2D formula and then optimizing this 3D structure using the conjugate gradients algorithm with the MMFF94 [33, 34] empirical FF. A genetic algorithm (GA) [35] with the default Avogadro parameters was then applied to generate a number of conformers from the optimized 3D monomer. Rotatable bonds were assigned by Avogadro automatically. Ten structures with the lowest MMFF94 energies were subsequently optimized using the conjugated gradients method with the PBE0 functional [23] supplemented by the D3(BJ) dispersion function [36] [PBE0+D3(BJ)]. Orca-4.2.1 electronic structure code [18] was used for this optimization with the def2-SVP [37] and def2-ECP [38] (for iodine only) basis sets.

B. Details of potential energy surface development

We created a 6D rigid-monomer PES using the global minimum monomer's geometry and the autoPES package [1, 2]. Equations (1)–(3) show the functional form of the fit. The general procedure is explained in Sec. I. The aiFF parameters for approximately symmetry-equivalent atoms, shown in Fig. 1, have the same values. The symmetry-equivalent atoms were selected following the OPLS-AA parametrization determined by LigParGen [39], making equivalent those atoms whose partial charges and van der Waals parameters vary by less than 0.1. In the aiFF development, we first fitted the charges, C_6, and C_8 parameters in Eq. (2) to 12,000 long-range grid points generated randomly. The long-range interaction energies for these dimers were calculated by asymptotic perturbation theory consistent with SAPT(DFT), implemented in the SAPT2020 codes [3, 16] using de Oliviera's *et al.* ADZP all-electron basis set [40] for iodines and the def2-TZVP basis set [37] for other elements. These parameters were then frozen in the fitting of the total PES to a set of close-range grid points (configurations with the center of mass distances between monomers, R, distributed around radial van der Waals minima). We calculated the interaction energies at these grid points with PBE0+D3(BJ) in the def2-TZVPP [37] basis sets using the Orca package. The counterpoise correction was applied to remove the basis set superposition error. Since the interaction energies of close-range and long-range dimers were calculated with different methods, we tested the differences between these approaches (i.e., the difference between

the supermolecular interaction energy from PBE0+D3(BJ)/def2-TZVPP and that given by the sum of asymptotic components of aiFF, both interaction energies computed for exactly the same geometry of the dimer) at a long-range separation between centers of mass of monomers equal to 16 Å (about three times larger than the van der Waals equilibrium distance of 5.4 Å), keeping the same orientation of monomers as in the minimum. The discrepancy relative to the supermolecular interaction energy turned out to be about 6%. Since this difference results both from inadequacies of PBE0+D3(BJ) in the asymptotic region and from inaccuracies of the fit, its magnitude appears reasonable. The finite-range set consisted of 12,266 grid points. The RMSE for points with $E_{\rm int} < 0$ is 0.69 kcal/mol. It took around 8 hours of wall time to calculate the interaction energy for one dimer on 16 cores of the AMD EPYC 7502 2500 MHz processor, while the other steps of our protocol are timewise negligible in comparison.

C. Crystal Structure Predictions

Only the monomer in the global minimum geometry was used in rigid-monomer CSPs. The UPACK CSP protocol described in Sec. I was applied with default parameters, except that we included also the groups $P2_1/a$ and $P2_1/n$. Given the size of the monomer, the cell dimensions were limited in the search to lay between 10 Å and 70 Å. The OPLS-AA FF [12] was used in the loose optimizations and aiFF was used in the tight optimizations. After one more round of clustering, a set of 1500 polymorphs ordered by lattice energy was generated and submitted to CCDC.

D. Results

After the submission deadline, it was found by CCDC that in addition to the previously known form A, there exists also a form B polymorph. However, the crystal structure of form B could not be determined, see the main text. It was also found that rotations of the TIPS groups around the links lead to disorder in crystals of form A. Due to the disorder issues, the evaluations of the submissions were performed using the standard approach (all non-hydrogen atoms) with RMSD_{20} and also using only the pentacene "core" (i.e., excluding the disordered isopropyl groups) match with RMSD_{30}. In the list of 1500 crystals submitted by us, none satisfied the criteria set by CCDC. In the standard comparison, none of our polymorphs had 20 molecules matching within the 25%/25° tolerances, one of the criteria used by CCDC for target XXVII. The best match with these tolerances had 6 matching molecules and $\text{RMSD}_6 = 3.7$ Å. Our list did include the structure ranked #749 by lattice energy with 30 molecules matching the molecules in the 90 K experimental crystal using the core-only comparison (with 30%/30° tolerances). However, this structure has RMSD_{30} of 2.05 Å, beyond the CCDC criteria of 1 Å.

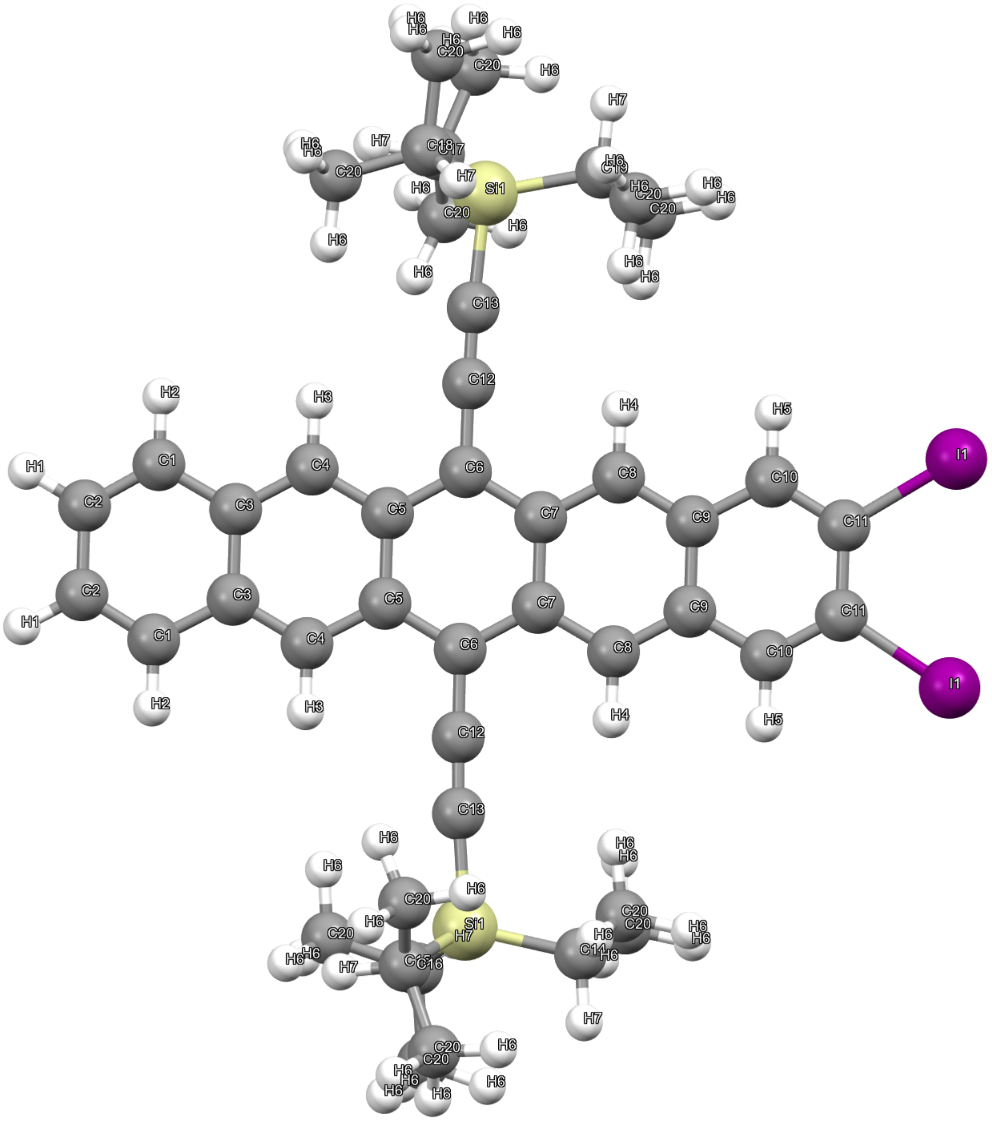

FIG. 1: Global minimum conformer of target XXVII. Atoms that have identical PES parameters are labeled with the same numeral. All methyl group hydrogens have atom type H6 in the top TIPS and H8 in the bottom TIPS, while the non-methyl hydrogens in the isopropyl groups are classified as type H7.

E. Post-submission analysis

The main reason our predictions were not better was that we performed rigid-monomer CSPs only for the global minimum conformer. This conformer has RMSD of 0.71 Å relative to the monomer from the 90 K experimental crystal. Thus, a major part of the allowed discrepancy is taken already by the discrepancy between monomers. In the post-submission work, we performed rigid-monomer CSPs using the conformer of rank 7, which has the smallest RMSD relative to the experimental monomer, amounting to 0.60 Å. This conformer's energy is 2 kcal/mol above the global minimum monomer energy. Despite this still fairly large RMSD, CSPs with this conformer resulted in a match with $RMSD_{30} = 0.98$ Å (tolerances 30%/30°, including only core non-hydrogen atoms), which would have been a success in the test. The rank of this polymorph is 45, acceptable for a system that large. Thus, the method consisting in performing a set of rigid-monomer CSPs for each low-energy conformer works reasonably well for target XXVII, we just did not have enough time to consider conformers beyond the minimum-energy one.

The reason for the fairly large RMSDs between the conformers and the experimental monomer is that the global minimum conformer shown in Fig. 1 has the C6-C12-C13-Si bonds nearly linear, with the C12-C13-Si bond angle of 177° (with the same value for the other TIPS group). Thus, the Si atom lies almost in the plane of the rings and the isopropyl groups are approximately symmetric with respect to the rings. As it can be seen from the figure of the experimental crystal structure in the main paper, such maximally extended TIPS groups cause steric hindrance. This hindrance can be reduced if one of the TIPS groups bends off-plane. Indeed, in the experimental monomer the C12-C13-Si bond angle is 170° (176° for the other TIPS group) and the C6-C12-C13-Si dihedral angles is 45° (22°).

III. TARGET XXVIII

Target's XXVIII monomer includes 53 atoms. It contains a central Cu atom bound to two Cl atoms and two N-H groups. The latter groups are connected via double bonds to carbon atoms which are then connected to two phenyl groups each. Rotations about Cu-N bonds constitute very soft coordinates, and the total number of flexible degrees of freedom is 8. Due to an unpaired electron on Cu, XXVIII is an open-shell system. No information other than the 2D structure was provided and the participants were asked to submit a list of 1500 unranked plymorphs. While the crystal structure of XXVIII was coincidentally published during 7BT by an external group, this information was not used in our process.

A. Conformer search and optimization

The 2D structure supplied by CCDC was the input to the Balloon package [6]. The initial 3D geometries of monomers were generated using the distance geometry method [41]. This method utilizes a collection of atom-atom distance ranges, commonly referred to as "bounds". If a set of all atom-atom distances in a molecule is selected within these bounds, a unique monomer geometry is defined. All possible geometries generated in this way on a reasonably dense grid should include monomer geometries fairly close to the conformer geometries (i.e., geometries of minima on the monomer's energy surface). The set of monomer geometries from the distance geometry method was subject to geometric modifications using GA [35]. These modifications include, in general, variations of the angles of rotation around flexible bonds, the stereochemistry of double bonds and tetrahedral chiral centers, as well as the conformations of nonaromatic rings. Finally, geometry of each of the monomers produced by GA was optimized by minimizing its energy computed using the MMFF94 force field [33, 34]. This step also removes duplicates since they converge to the same structure. Also, the structures whose energies were greater than a preset value from the set's lowest energy were eliminated. In this way, eight unique conformers were generated. All these conformers were further optimized using the unrestricted (due to the open-shell character of XXVIII) PBE+D3(BJ) method [22, 24] implemented in the ORCA-4.2.1 package [17, 18]. The use of dispersion-corrected functionals is important for monomers as large as that of target XXVIII. The aug-cc-pVTZ [19, 20] basis set was used in this geometry optimization. The global-minimum conformer is shown in Fig. 2.

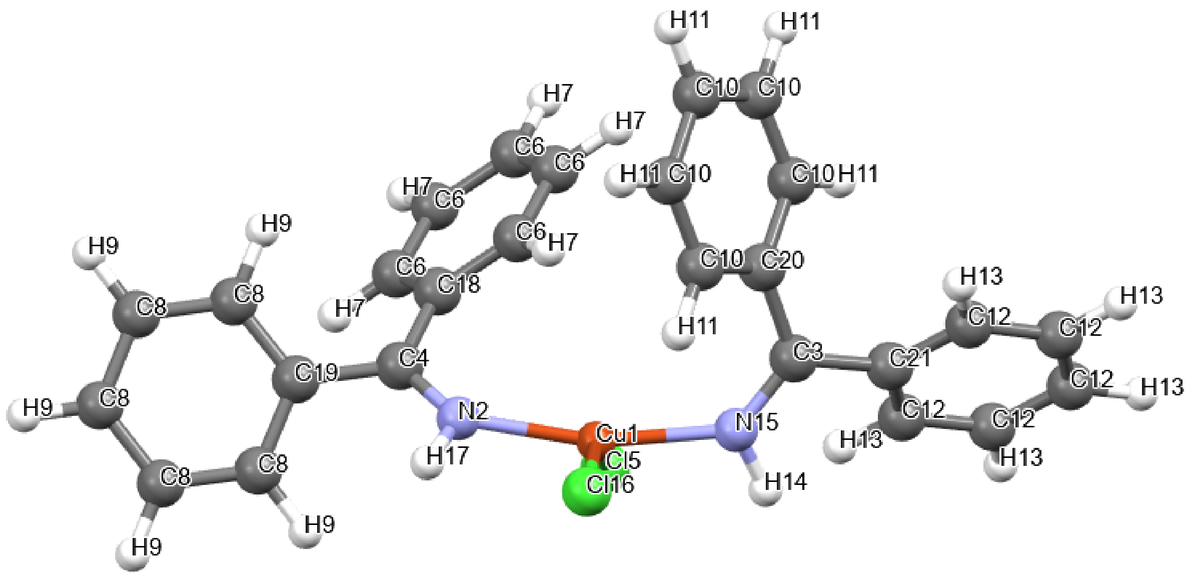

FIG. 2: Global-minimum conformer of target XXVIII.

B. Details of potential energy surface development

For the generation of the aiFF with autoPES [1, 2], we used the global-minimum conformer. A general description of the fitting process is given in Sec. I. The interaction energies were calculated in the supermolecular way with the counterpoise correction using PBE0+D3(BJ) [22–24] with the cc-pVTZ basis set. As in the optimization of the monomer geometry, we used the unrestricted Kohn-Sham method to take into account the open-shell character of monomer XXVIII. However, the asymptotic programs that are used in autoPES for long-range fitting work only for closed-shell molecules. The only practical way around this problem was to replace in the asymptotic calculations the copper atom with the zinc atom, while keeping the same geometry as in the Cu-containing molecule. To find how well this approximation works, the interaction energy curves for various *ab initio* methods were compared and it was found that this replacement of Cu with Zn led to errors of about 6-7% in the long-range region, i.e., at the distances between monomers' centers of mass equal to about 2.5–3 times the van der Waals minimum distance, justifying our procedure. The training set of interaction energies was fitted by the functional form of the potential given in Eq. (1). The polynomial term in the site-site function of Eq. (2) was expanded to second order and the $1/r_{ab}^{12}$ term was included in order to achieve the correct behavior of the potential at very close range. In the asymptotic part of Eq. (2), only the Coulomb and the C_6^{ab}/r_{ab}^6 terms were included. The atom-atom parameters in Eq. (1) for (approximately) symmetry-equivalent atoms were set to be equal, see Fig. 2, which reduced the number of atom types from 53 to 21. The RMSE of the fit for $E_{\rm int} < 0$ was 0.42 kcal/mol. Such a value is typical of molecules of this size and the relative error with respect to the interaction energy of the global minimum was only 1.9%. The number of grid points ($N_{\rm grid}$) used was 6328 and the total number of free parameters ($N_{\rm FP}$) was 504. Thus, the ratio $N_{\rm grid}/N_{\rm FP}$ was 12.6.

C. Crystal Structure Predictions

The CSP protocol for system XXVIII followed the description in Sec. I with all parameters used at their default values, except that we included also the groups $P2_1/a$ and $P2_1/n$. These CSPs assumed rigid monomers at the minimum-energy conformation.

D. Results

In our submitted list of 1500 polymorphs, no structure with RMSD_{30} below 1.0 Å relative to the experimental crystal was found. The closest prediction matched only 4 molecules with $\text{RMSD}_4 = 2.1$ Å. Such a performance was due to our use of only the global-minimum conformer in the PES generation and in the CSPs. This conformer has an RMSD of 2.1 Å relative to the

experimental monomer, and therefore it was impossible to meet the criterion of 1.0 Å set by CCDC already at the monomer level.

E. Post-submission analysis

If not the time constraints, we would have performed CSPs with all 8 low-energy conformers that we found. However, even if we had done this, it would be difficult to find the experimental structure to within the 7BT accuracy thresholds. The reason is that our conformers' lowest RMSD relative to the experimental monomer is 0.8 Å (for conformer 2 with the second-lowest energy), very close to the criterion of 1.0 Å for the crystal. CSPs performed with this conformer found only 8 matching molecules within the standard tolerances, and $RMSD_8$ was equal to 1.19 Å. At this point, it looked like this system, which has 8 soft degrees of freedom, may be too flexible for the conformer-based procedure we used, i.e., the monomers undergo too significant deformations relative to their gas-phase conformer geometries due to the forces exerted by the other crystal molecules.

To investigate this issue further, we performed CSPs using the rationalized experimental monomer geometry. First, the X–H bonds of the experimental structure, where X is any atom bound to hydrogen, were optimized using the PBE+D3(BJ) method with the aug-cc-pVTZ basis set. This step was necessary since the positions of hydrogen atoms in experimental structures have very large uncertainties. Next, we performed the CSP procedure with such monomer. The experimental polymorph was found at rank 1 with an $RMSD_{30}$ of 0.184 Å, indicating that the quality of the intermolecular aiFF is sufficient for CSPs (this is also confirmed by the performance of our group in stage 2, where we identified the experimental polymorph as rank 1 on the list of structures provided by CCDC).

Thus, the main question became why none of the conformers is closer to the experimental monomer. To check if we have not missed a conformer, we performed full optimization of the experimental conformer and it ended in conformer 2. In fact, the two geometries are quite similar despite the large RMSD. The experimental monomer has inversion symmetry, while the geometry of conformer 2 is distorted due to Jahn-Teller effect. We have then built a monomer from conformer 2 in such a way that we took half of this monomer and obtained the other half by the inversion symmetry operation. The RMSD of this monomer with respect to the experimental one is only 0.137 Å, and it is only 1.4 kcal/mol above conformer 2. Thus, the monomers in the crystal tunnel between conformer 2 and its symmetric equivalent, so that the experiment may measure the average geometric position. Note that while we found this by comparing the *ab initio* optimized and experimental monomers, one could predict *a priori* that the experimental monomer will have this kind of structure. Another possible explanation is that the electromagnetic field on the monomer due to other monomers in the crystal results in breaking the degeneracy of monomer's wave function

without the Jahn-Teller deformation. We have performed CSPs with the centrosymmetric *ab initio* monomer and they resulted in the experimental-like crystal at rank 1 with $\mathrm{RMSD}_{30} = 0.292$. Thus, target XXVIII actually does belong to the group of crystals that can be investigated by the conformer-based protocol, provided that symmetric structures close to conformers are considered.

IV. TARGET XXIX

The target XXIX monomer has 20 atoms and 4 soft degrees of freedom. However, only 2 of them have to be considered since the rotation of the methyl group has a minor impact on CSPs and the amino group forms an internal hydrogen bond in all low-energy conformers. A low-resolution powder X-ray diffraction (PXRD) pattern graph of the experimental crystal structure was provided by the CCDC. In addition to submitting 1500 unranked structures, participants were also asked to submit a ranked list of 10 structures matched to the PXRD pattern. Target XXIX was not a part of the stage 2 of 7BT.

A. Conformer search and optimization

The Avogadro package [5] was used for building a 3D structure from the 2D one provided by the organizers. A preliminary minimization of this structure was performed with the universal force field (UFF) [42]. This 3D structure was used as the starting point in the conformer generator program [7], which is a part of Mercury [8], to generate additional conformers. We selected two of these conformations (the remaining ones where much higher in energy or differed only by small rotations of the methyl group) and optimized them using the PBE0 functional and the aug-cc-pVTZ basis set [19] in ORCA-4.2.1. A local minimum conformer was found within about 2.8 kcal/mol above the global minimum one (shown in Fig. 3). Hence, both conformations were assumed to be relevant for CSPs. The global minimum conformer has an intramolecular hydrogen bond between the amine hydrogen and carboxylic oxygen, whereas the local minimum one has an intramolecular hydrogen bond between the amine hydrogen and the ester oxygen atom.

B. Details of potential energy surfaces

The aiFFs for the target XXIX dimer were developed using SAPT(DFT) and the aug-cc-VDZ basis set in the $\mathrm{MC^{+}BS}$ form with midbond functions. Both conformers described above (hereafter, the global and local minima conformers will be called A and B, respectively) and their combinations were used, i.e., we developed 3 PESs AA, BB, and AB. A set of 6212, 6705, and 14,293 dimer configurations, respectively, was used for training these PESs. The PESs have RMSEs of 0.14, 0.15,

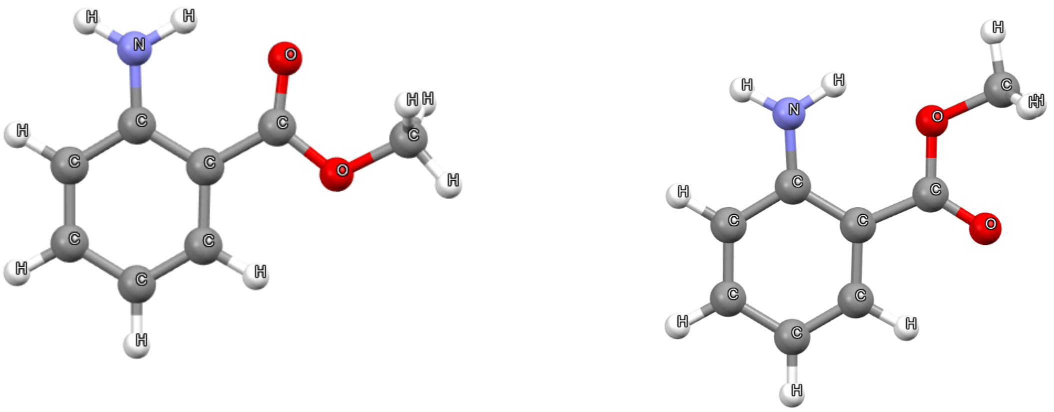

FIG. 3: Global-minimum and lowest local-minimum conformers of target XXIX. For this monomer, each atom had a distinct set of parameters.

and 0.15 kcal/mol on the 5039, 4808, and 10,153 dimer configurations in the negative interaction energy region, respectively.

After CSPs were performed using the first generation of the potentials AA and BB, the top 20 generated polymorphs ranked by lattice energies were selected for extracting dimers and improving the PESs using the CCfC procedure of Ref. 28 (see Sec. I). The potentials were then refitted using 253 and 252 additional dimers extracted from AA and BB CSPs, respectively. These new potentials are labeled $\overline{\text{AA}}$ and $\overline{\text{BB}}$. Due to time constraints, the CCfC procedure was not complete: CCfC iterations have to be performed until the CSPs rankings do not change between two consecutive iterations [28], while we performed only one iteration. The RMSEs of these PESs were the same as of the corresponding initial fits. Also due to time constraints, we were not able to perform CCfC for the AB aiFF. $\overline{\text{AA}}$, $\overline{\text{BB}}$, and AB FFs were used in the final CSPs. All PESs should be hole-free for all practical purposes (i.e., the remaining holes should be behind high energy barriers). However, since the CCfC were incomplete or not performed, the relative energies of some local minima may be less accurate than the RMSEs indicate.

C. Crystal Structure Predictions

Using the optimized geometries of the two conformers (A and B) and the aiFFs ($\overline{\text{AA}}$, $\overline{\text{BB}}$, and AB), rigid-monomer UPACK CSPs were performed in 20 space groups: the 13 default ones plus $P222$, $P222_1$, $P2_12_12$, $C222_1$, $Pba2$, $Iba2$, and $Pccn$ (also the variants of $P2_1/c$ were used), with $Z' = 1$, 2, 3, and 4. In addition to searching pure crystals of each conformer, a subcategory of

searches included mixing of A and B conformers in ratios 1:1, 2:1, 1:2, 2:2, 3:1, and 1:3. All searches used only rigid monomers. To rank the structures, the intermolecular energy contributions from the SAPT(DFT) potential were combined with a conformer energy penalty of 3.1 kcal/mol from the isolated molecule DFT calculations for each instance of the higher energy conformer in the structure (this number is slightly different from that reported in Sec. IV A since it was inadvertently computed in a different basis set). The 1500 lowest energy structures were submitted.

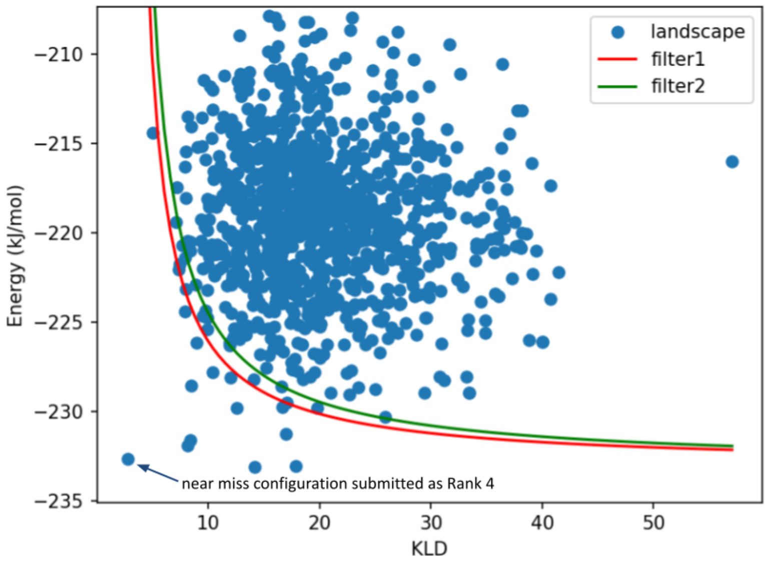

FIG. 4: Energy vs. KLD Pareto-chart for the mixed conformer 2:1 ($Z' = 3$) search in $P2_1/n$. Structures below the threshold (filter 2) are selected for further unit cell parameter optimization.

The set of all polymorph candidates obtained from UPACK's CSPs was then processed using a semi-automated 'PXRD-based' screening procedure which allowed consideration of both the energetic ranking as well as the quality of the fit between simulated and provided PXRD. This screening procedure involved several stages detailed herein. Individual batches of structures were organized based on Z', conformer type, conformer ratio, and space group used in UPACK's CSPs. A batch-wise energy cutoff was used so that the PXRDs were calculated for a manageable set of <3000 structures per batch. Because the observed PXRD information was provided as a raster image, a separate treatment with image processing tools [43] was necessary to reconstruct the peaks using a Gaussian kernel and performing a background subtraction so that the observed PXRD intensities were represented as functions of 2θ angle. The PXRD similarity measures, S12 [44] and Kullback-Leibler's divergence (KLD) [45], were calculated for each structure using the observed intensities. Screening based on these similarity measures selected a few hundred candidate structures per batch. Then, an 'Energy vs. KLD Pareto-chart' [46] protocol was used to reduce the number of possible candidates to 30 per batch (see Fig. 4). These structures were then

subjected to a cell parameter optimization procedure to minimize a cost function based on S12 and KLD. This optimization is a generalized least-squares fitting in which FF interaction energies are initially used only to hold monomers in place while the cost function is the KLD. After each iteration, however, monomers' positions within the unit cell are lattice-energy minimized keeping the unit cell fixed. The post-fitting value of the cost function is used to identify a possible match to the observed PXRD. From all random searches, the best candidate was a mixed conformer, with an A:B ratio of 2:1 ($Z' = 3$) in $P2_1/n$ (see Fig. 5), ultimately submitted as the rank #4 candidate on the 10 ranked structures list.

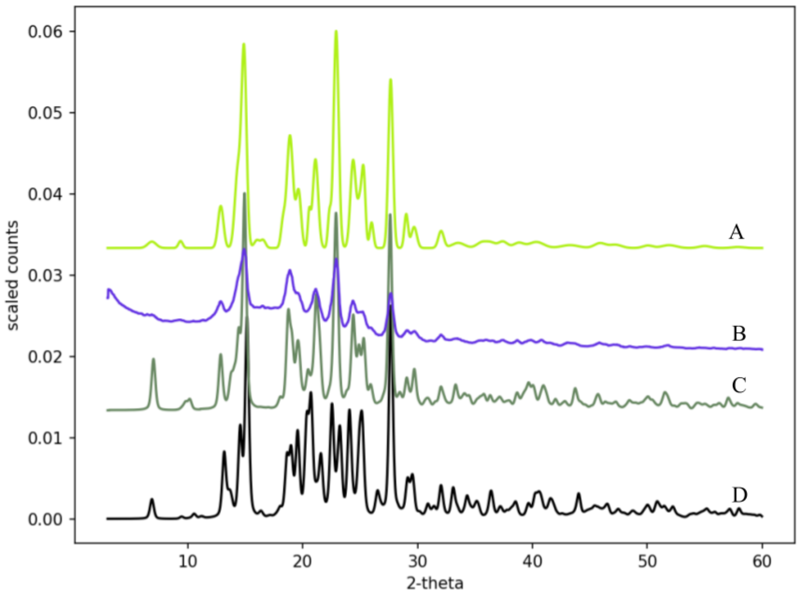

FIG. 5: Visual comparison of PXRD related to the rank #4 structure submission. Patterns are as follows: A: background-corrected reconstruction of raw observed data; B: reconstruction of the raw observed data; C: calculated from the submitted structure (after unit cell parameter optimization); D: calculated from structure as generated (before the unit cell parameter optimization).

This rank #4 structure was used as the starting point for additional work to identify matching candidates. Extended variable (EV) coordinates taken from this structure were used as seeding coordinates for structure searches performed with the EVCCPMRE algorithm [32]. This gave another candidate in space group $P2_1$ which was submitted as rank #2. The structures for the rank #1, #3, #5, #6, and #7 were constructed manually, optimized with the aiFF potential, and finally subjected to unit cell optimization using the PXRD cost function described above. Structures for rank #8, #9, and #10 were other candidates from the optimization of the cell

parameters to maximize the PXRD overlap that had low cost-function values. Although the 10 ranked submitted polymorphs were using both the cost functions and the lattice energy, all of them have lattice energies near that of the minimum-energy structure.

D. Results

The analysis of our CSPs performed by the CCDC on the 1500 submitted structures has been reported as resulting in no matches. This is partly due to changes in the criteria of what constitutes a match. In fact, polymorph #937 on the submitted list is a pure conformer A crystal (as is the experimental crystal built of monomers nearly identical to conformer A) with $Z' = 3$, 27 matching molecules, and $\text{RMSD}_{27} = 0.197$ Å relative to the experimental crystal (with the distance and angle tolerances of 35% and 35°, respectively). These results show that our aiFFs and the CSP protocol provide reliable predictions. However, the RMSD_{20} criterion used in previous blind tests was changed by CCDC to RMSD_{30} at the stage of evaluating the submissions and therefore this polymorph has not been considered a success despite being a pretty good match of the experimental one, in particular due to its very small RMSD. We should state, however, that despite the good match, the space groups of the two polymorphs were different: *Pbcn* for #937 and $P2_1/c$ for the experimental crystal.

While structure #937 was the closest to the experimental one by the RMSD_{20} criterion, our analysis indicates that polymorphs close to the experimental crystal were also found at ranks 66, 75, 303, 367, and 459 in $Z' = 2$ with $\text{RMSD}_{20} = 0.285$, 0.268, 0.243, 0.243, and 0.281 Å, respectively. None of these polymorphs matches with 30 molecules, but the highest numbers of matched molecules are close to 30. The RMSD values (number of matching molecules) are as follows: 0.328 (27), 0.298 (27), 0.295 (28), 0.291 (28), 0.336 Å (28), respectively.

Obviously, there were no matches in our submission of the 10 ranked structures. Among the structures submitted on this list, the structures with ranks #1–7, all with $Z' = 3$, are a family of structurally similar polymorphs that reasonably closely resemble the experimental structure, as they have RMSD values (number of matching molecules) of 0.284 (17), 0.283 (17), 0.266 (19), 0.382 (18), 0.595 (19), 0.595 (19), 0.711 Å (19), respectively (with 35% distance and 35° angle tolerances). Although the number of matching molecules is smaller than in the case of UPACK-only CSP results presented above, the PXRD/EVCCPMRE predctions are significant due to the fact that these polymorphs are ranked high.

E. Post-submission analysis

The overlap of conformer A with the experimental monomer is perfect, with RMSD below 0.001 Å (for conformer B, the RMSD is 1.1 Å). Thus, in hindsight, monomer flexibility was not an

issue for target XXIX, i.e., rigid-monomer CSPs using only monomers in the conformer A geometry and $Z' = 1 - 4$ should give as good predictions as flexible-monomer CSPs.

While the match of the structure #937 with experiment was reasonably good, its energy ranking (not considered by CCDC in the evaluation of the 1500 structures in submission 1) was not. Using a similar methodology, the rankings of experimental-like crystals in Ref. [4] obtained using only aiFFs were 16 or better, most at rank 1. To check our procedure, we repeated UPACK CSPs considering only homogeneous crystals, both for conformer A and B, in $Z' = 1$, 2, and 3. The results of these CSPs were combined and ranked by lattice energies which in the case of crystals made of conformer B included the monomer deformation penalty. This combined list included a pure conformer-A polymorph which is a good match to the experimental polymorph at rank 10 in $Z' = 2$ with 28 matching molecules and $\text{RMSD}_{28} = 0.352$ Å. Both polymorphs belong to the $P2_1/c$ space group. Although the predicted Z' value is different from that of the experimental crystal and the number of matching molecules is still below 30, this reasonably high lattice-energy ranking might suggest that some kind of mistake could have been made when sorting the structures for the submission. The other possibility is that the AB PES included a number of local minima with energies too large in magnitude, but only by a couple of kcal/mol. Such regions are mostly not detected by the hole removing procedure of autoPES. If such a potential is used in CSPs, polymorphs probing these local minima may have low lattice energies despite not resembling the experimental crystal. This can happen even if a given aiFF is very accurate in the regions of all minima relevant for predicting correct crystal structures, so that it can achieve a good match with the experimental structure, but this match can be far on the lattice-energy list. The CCfC protocol is actually aimed at removing this problem, but due to time restrictions we were not able to perform it for the AB PES and we made only one iteration for AA and BB. In the submission, the list included both pure and mixed-conformer crystals and it is possible that some of the latter had too low lattice energies due to the issue described here. In the post-submission CSPs, we included only pure crystals.

Another question that we considered in the post-submission analysis is why the PXRD/EVCCP-MRE refinement of the structures predicted by UPACK resulted in a reduced number of matching molecules. While we do not have an answer to this question, we acknowledge that challenges arise for any actionable re-purposing of information from near-miss structures to obtain a correct experimental structure. Future efforts involving a combination of EVCCPMC [32] and crystal adiabatic free energy dynamics CAFED [47] may answer these challenges.

As a part of the post-analysis, we have investigated further recycling of structural information from near-miss structures within the EVCCPMC framework. By "near-miss" (which could also be called near-hit), we label structures that are false positive, i.e., are not clear negatives, but are not exactly the correct structures that needed to be identified. In particular, when we have taken the EV directly from the experimental structure and used to seed the EVCCPMC algorithm, the

experimental structure was generated with a single EVCCPMC step. Remarkably, if a particular near-miss EV is used as a seed, then only a small number of EVCCPMC steps are required to generate structures with similarity scores as high as 29/30 molecules ($\text{RMSD}_{29} = 0.167$ Å). These results suggest that a screening procedure that incorporates PXRD similarity measurements is useful.

V. TARGET XXX

Target XXX was a cocrystal consisting of a small, essentially rigid monomer T (tetrametylpyrazine) with 18 atoms and a large monomer C (cannabinol) with 49 atoms. Monomer C has an essentially rigid main body and a very flexible pentane appendage. In addition to the 2D structures of the monomers, CCDC provided information that possible stoichiometries C:T are 1:1, 2:1, 1:2, of which two have been observed. The participants were asked to submit 1500 unranked structures, 100 ranked structures, and a prediction of actual stoichiometries. Target XXX was not included in stage 2 of 7BT.

A. Conformer search and optimization

The conformer search protocol for system XXX follows the same steps as that of system XXIX. The top 20 conformations of C generated by the conformer generator and a single conformation of T were optimized using PBE0 plus D3 and the aug-cc-pVTZ basis set in ORCA-4.2.1. The four lowest-energy conformations of C were selected as possible candidates for CSPs. These conformations are within 1 kcal/mol of the global minimum, but geometrically they are quite different due to the orientation of the pentane appendage with respect to the rest of the molecule. Ultimately, due to time limitations, the lowest-energy local minimum, which is only 0.25 kcal/mol above the global minimum, was used for CSPs. The global minimum was deemed unlikely to create optimal crystal packing since the pentane tail is not in the same plane as the aromatic rings. The conformers used in the aiFF development are shown in Fig. 6.

B. Details of potential energy surfaces

Four aiFFs (TT, $\overline{\text{TT}}$, CC, and TC) were developed using rigid monomers and applying SAPT(DFT) with the aug-cc-pVDZ basis set in the MC^{+}BS form with midbond functions. A set of 672, 5471, and 2463 dimer configurations was used for training the potentials of TT, CC, and TC, respectively. The converged aiFFs of TT and TC have an RMSE of 0.16 and 0.39 kcal/mol on the 553 and 1701 dimer configurations in the negative interaction energy region, respectively.

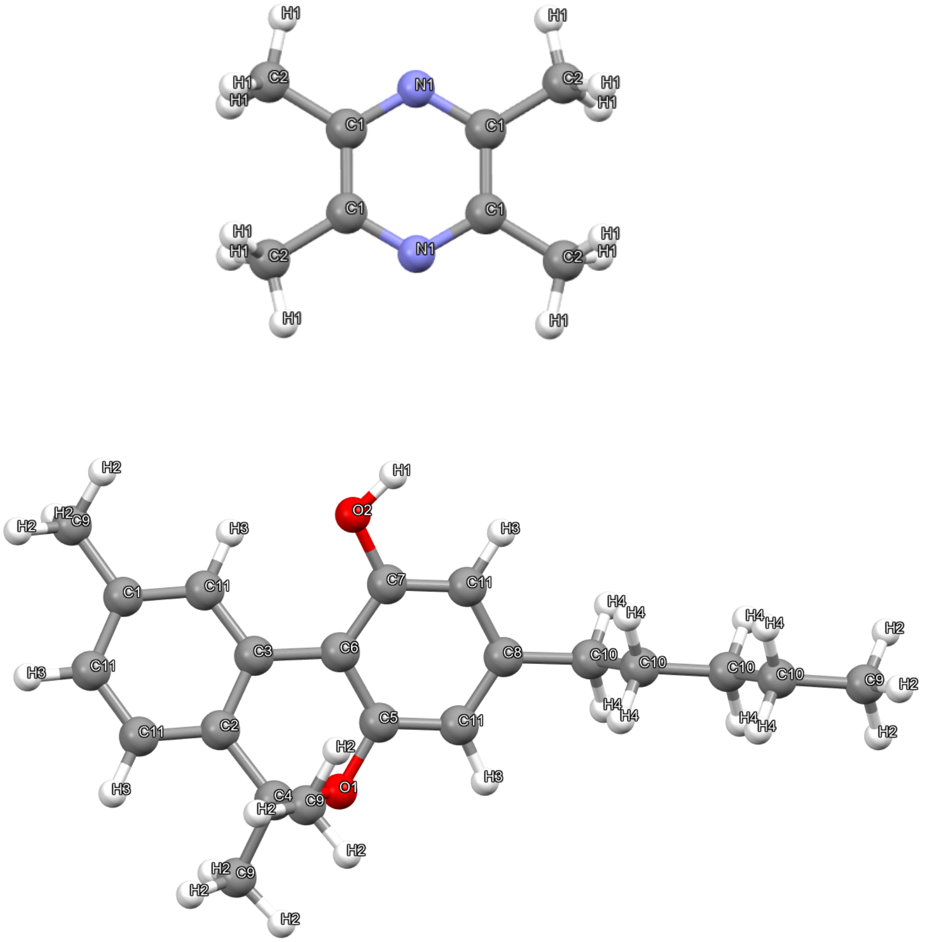

FIG. 6: Conformers of target XXX used in CSPs.

Both fits were converged and hole-free. Next, we improved the TT aiFF by adding 224 dimers extracted from the top 20 crystals of the homogeneous T CSP using the CCfC procedure of Ref. 28, obtaining the $\overline{\mathrm{TT}}$ fit with RMSE of 0.17 kcal/mol on the 769 dimer configurations with negative interaction energies. The accuracy of the two fits could be validated since the experimental crystal structure of monomer T is known. The CCfC procedure led to a significant improvement in the ranking of experimental-like crystal structures of T, i.e., from rank 34 (RMSD_{20} = 0.161 Å) for the TT FF to rank 4 (RMSD_{20} = 0.216 Å) for the $\overline{\mathrm{TT}}$ FF. The latter aiFF was used in CSPs. Due to time limitations, we were not able to perform CCfC refinements for the fits TC and CC. In fact, we were not able to fully converge the CC aiFF, which, as a consquence, contained some holes. This fit has an RMSE of 0.47 kcal/mol on a set of 2885 dimer configurations in the negative interaction energy region.

C. Crystal Structure Predictions

For each stoichiometry (1:1, 1:2, and 2:1), the UPACK program suite [4, 29] was used to generate 3000-8000 random structures for both flexible and rigid monomers in each of the 13 common space groups (UPACK's default). In the latter case, the conformers from Sec. V A were used. After the structures were generated, we used a somewhat different protocol for structure optimization than in other UPACK searches. This protocol consisted of three consecutive steps: (i) using the OPLS-AA force field [12, 48] with aiFF partial charges for both the intermolecular and intramolecular interactions, we performed both flexible-monomer and rigid-monomer crude optimizations that started from the corresponding randomly-generated structures; (ii) using the output from the previous step (reduced by removal of duplicates), a second flexible-monomer minimization with a larger cutoff (18 Å) was performed with the same OPLS-AA force field (this means that monomers treated as rigid so far were allowed to flex; also, in this step only, we used UPACK's option of imposing pressure with $P = 1$ bar); (iii) on the reduced output from step (ii), final rigid-molecule minimizations with the aiFFS were performed. While the last minimization step assumed rigid monomers, in general each of the monomers had a different geometry. The polymorphs generated in each of the three types of searches were ordered by their lattice energies including monomer deformation penalties relative to the minimum-energy monomer in the intramonomer force field used. Also, in rigid-monomer searches, monomer energies have to be taken into account since we consider cocrystals with different stoichiometries. The lattice-energy orderings were used to limit the number of polymorphs passed from one step to another.

Low-energy structures identified from all these random searches were subsequently used as seed structures for performing both rigid-monomer and flexible-monomer searches with the EVCCPMRE [32] method. EVCCPMRE searches were performed using OPLS-AA with charge assignments from the aiFF. A minimum of 3 cycles of EVCCPMRE were performed for each set of stoichiometries generating 400 structures per space group per cycle. As new global minimum energy structures were identified across all space groups, the structural information was recycled as seed EV's for each successive search cycle. This approach was successful in identifying global minima that are deeper than and have different geometries from those generated by UPACK, as well as many low-energy and high-density structures that were not produced by UPACK. The EVCCPMRE-generated structures were reoptimized keeping monomers rigid in their geometries from the EVCCPMRE generation and using the aiFF for intermonomer interactions. After adding proper monomer deformation penalties, a single list of 1500 structures was formed, with the structures ordered by lattice energies. This list, as well as its subset of 100 top-ranked polymorphs, were submitted. Our search included three possible stoichiometries, but our list of submitted structures included only 1:1 and 2:1 C:T stoichiometries, as this was our stoichiometry prediction.

D. Results

Two polymorphs of the target XXX cocrystal are known. Form A has stoichiometry C:T = 2:1 and exhibits crystallographic disorder in the appendage, leading to forms A_{maj} and A_{min}. Form B has stoichiometry 1:1. Thus, our stoichiometry prediction was correct. However, none of the polymorphs on our list was close enough to any of the experimental polymorphs to constitute a successful prediction according to the CCDC criteria.

E. Post-submission analysis

We first analyzed the similarity of the conformers used to develop the aiFFs to the monomers in the experimental polymorphs. The lowest-energy conformer of tetrametylpyrazine matched very well with monomers in all forms of the XXX cocrystal: the RMSD was 0.017/0.051 Å for the A/B forms. The *ab initio* conformer of cannabinol used in the aiFF developments (conformer 2, the lowest local-minimum monomer) is significantly different from the cannabinol monomer in the experimental polymorph B, as depicted in Fig. 7a, with an RMSD of 1.219 Å. However, most of the differences come from the pentane appendage (this is better visible better if the rings are aligned). The same conformer matches fairly closely with the monomer in the experimental polymorph A_{maj}, see Fig. 7b: the monomer overlap RMSD is 0.421 Å. Again, the majority of the deviation is coming

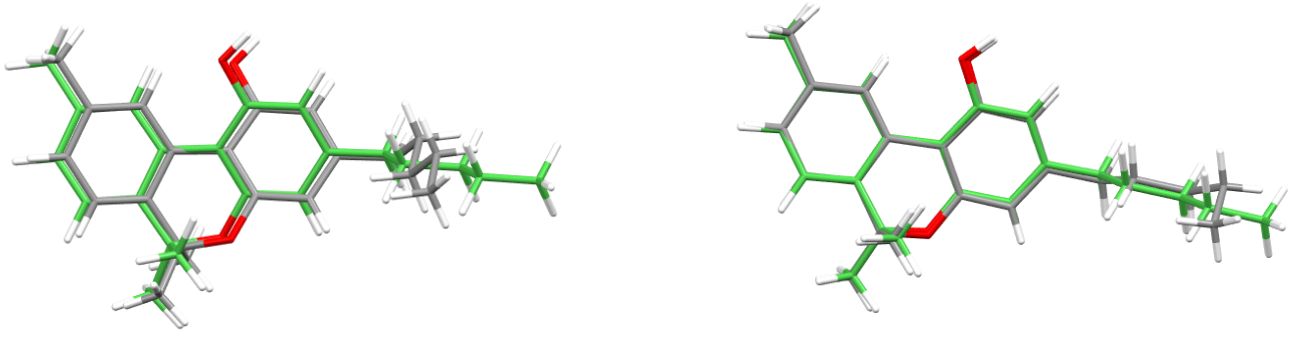

(a) Polymorph B, 1:1 stoichiometry

(b) Polymorph A_{maj}, C:T = 2:1 stoichiometry

FIG. 7: Overlap of the *ab initio* conformer of C (the first local-minimum monomer) (green) with the experimental monomers from polymorphs (a) B and (b) A_{maj} for target XXX.

from the two terminal carbons of the pentane appendage, which are situated 0.806 and 1.591 Å away from the equivalent carbon atoms in the experimental monomer. Thus, the conformers used in the development of aiFFs were reasonably close to experimental monomers.

However, in our set of conformers of cannabinol, there are closer matches than those for conformer 2, with RMSDs of 0.845 and 0.372 Å, respectively. Thus, one may hope that conformer-

based rigid-monomer CSPs could be successful for target XXX. To check this, we carried out such CSPs with conformer 2 as a part of the post-submission analysis. The best prediction was for the A_{maj} form with rank 23 and $RMSD_6 = 2.7$ Å. However, the most likely reasons for this poor match are the shortcomings of the CC aiFF discussed above, so at this point we cannot answer the question concerning suitability of conformer-based CSPs for target XXX. Similarly, we are unable to assess the adequacy the OPLS-AA representation of intramonomer interactions in target XXX.

VI. TARGET XXXI

Target XXXI monomer contains 32 atoms. It includes two rings connected by an -SO_2-CF_2-bridge. The monomer has three relevant soft degrees of freedom (the two methyl groups are also rotatable, but these rotations have only a minor effect on crystal structures). The participants were informed that two polymorphs of the crystal are known and were requested to submit 1,500 unranked structures.

A. Conformer search and optimization

We used Avogadro [5] to create a 3D structure of the monomer from the 2D chemical diagram provided by CCDC and then applied UFF [42] to carry out a preliminary minimization of the monomer. These two steps were conducted to supply a reasonable initial monomer geometry to the Conformator program [9]. Conformator constructs ensembles of monomers with different geometries utilizing a collection of typical torsion angle data from the Cambridge Structural Database [49], and then ranks conformations using the empirical FFs from Refs. 50 and 51. With the maximum number of conformers set to 20, the 'Best' option was utilized. The 10 lowest energy conformers were selected and optimized using the ORCA-4.2.1 [17, 18] package with the PBE [22] functional plus the D3(BJ) dispersion correction [24] approach and the aug-cc-pVTZ [19] basis set. The set included two pairs of optical isomers and all conformers were within an energy range of 3.5 kcal/mol above the global minimum. For the development of the PES, we only used the global-minimum conformer. Other conformers were used in the crystal structure predictions and analyzed in the post-submission part.

B. Details of potential energy surface

The PES for the dimer of monomer XXXI was generated with autoPES [1, 2] utilizing SAPT(DFT) [15] with monomers described by the PBE [22] functional and the aug-cc-pVDZ [19] basis set in the MC^+BS form with midbond functions. The atom-atom functional form of the

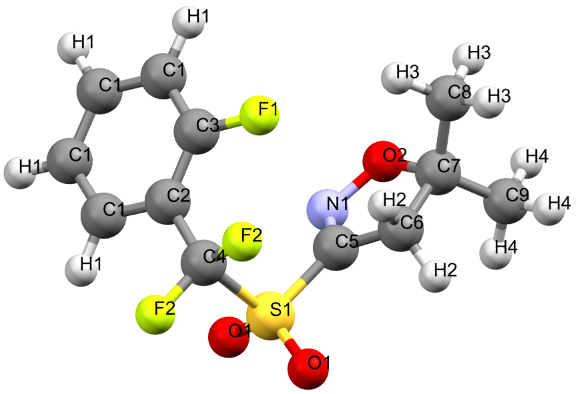

FIG. 8: Global-minimum conformer of target XXXI.

PES is given by Eq. (1). For details of the fitting process, see Sec. I. As stated above, only the global-minimum conformer was used in the development of the rigid-monomer PES. Atoms with approximately symmetric environments in the monomer were regarded as equivalent, reducing the number of atom types from 32 to 18 (see Fig. 8) and, respectively, the required number of free parameters in the fitting of the PES. The polynomial term in the atom-atom function of Eq. (2) was truncated at the second order and the $1/r_{ab}^{12}$ term was included. In the asymptotic part, only the Coulomb term and the $n = 6$ induction-dispersion term were included. A set of 10,672 dimer configurations in the close range and 12,000 in the long range was used for fitting the PES. The converged aiFF has an RMSE of 0.16 kcal/mol on the 6605 dimer configurations in the $E_{\text{int}} < 0$ region. The total number of parameters (N_{FP}) was 378 and the ratio of the total number of the grid points to the number of free parameters, $N_{\text{grid}}/N_{\text{FP}}$, was 28. The number of detected minima in the PES was $N_{\text{min}} = 92$.

C. Crystal Structure Predictions

All CSPs for target XXXI were performed with rigid-monomer searches using the UPACK program suite [4, 29]. For each of the 10 lowest-energy conformers (including optical isomers), 5000 structures were generated with a random search for $Z' = 1$ in each of the 13 most common space groups (the default of UPACK). The generalized AMBER force field (GAFF) [13] was used in the first optimization step. The parameters of GAFF were determined with the Antechamber program package provided by AmberTools20 [52] except for the partial charges which were obtained for each conformer separately using the RESP approach [30] and the electron density computed

using Gaussian16 [53] with the aug-cc-pVTZ basis set and the PBE+D3(BJ) functional. Crystal structure searches were performed for each of the rigid-molecule conformers separately. The initial minimization was followed by a refinement step employing the aiFF. As stated above, this fit was developed with a training database containing only the global minimum conformer, but was used here in its flexibilized form for other conformers. For the final ranking, a conformer energy penalty, corresponding to the *ab initio* energy difference between the different conformers relative to the global energy minimum as determined in section VI A, was added to the intermolecular energy of each structure to obtain the lattice energy.

D. Results

While the 7BT information provided by CCDC specified that there are two known polymorphs of the crystal of monomer XXXI, it turned out that one of these polymorphs included crystallographic disorder involving the fluorine atoms, leading to polymorphs A_{maj} and A_{min}. The other polymorph is denoted by B (there exists also polymorph C, but it has a structure with voids and was not expected to be found in CSPs). Within our 1,500 submitted structures, the structure with rank 1192 was identified by CCDC as a match to the experimental structure A_{min} with an $RMSD_{30}$ of 0.857 Å.

E. Post-submission analysis

We first analyzed the conformers generated by us by comparisons with the experimental monomers. The global minimum conformer is far from any experimental monomer, with the smallest RMSD of 1.81 Å (relative to the monomer from the A_{min} polymorph). This is due to its fairly 'closed' structure, see Fig. 8. Conformers 2 and 4, with 'open' structures, are much closer to experimental monomers. Conformer 4 matches the monomers from experimental structures B, A_{maj}, and C with RMSD of 0.16, 0.31, and 0.37 Å, respectively. Conformer 2 matches the monomer from A_{min} with RMSD of 0.277 Å. This relation of conformers 2 and 4 to the monomers from A_{min} and A_{maj}, respectively, is expected since the two conformers and the disordered forms differ by the rotation around the S1-C5 axis (Fig. 8). Since conformers 2 and 4 have small RMSDs compared to the CCDC acceptance criteria for the crystals, our method of rigid-monomer searches with low-energy conformers should work reasonably well for target XXXI.

To investigate why our method did not result in more matches, and also why the only match was so high in rank (ranking was not required by CCDC, but our list was ranked), we repeated the CSPs replacing GAFF by OPLS-AA with aiFF charges in the crude optimization stage and using the same aiFF for the final optimizations as in the submission CSPs. First, we performed CSPs using the rationalized monomer from the A_{min} polymorph. The rationalization consisted in

optimizing the X-H bonds using the PBE+D3(BJ) approach and the aug-cc-pVTZ basis set (this step is needed due to the approximate locations of hydrogen atoms in experimental structures). These CSPs resulted in the experimental structure ranked as number 2 with an $RMSD_{30}$ of 0.204 Å. We then repeated the procedures with conformer 2 (the closest match to the monomer from A_{min}) and got a match to the A_{min} crystal with $RMSD_{30}$ of 0.963 Å at rank 5, a satisfactory prediction. The same procedures were repeated with the rationalized form of the monomer from structure B, leading to the prediction of the form B crystal at rank 4 with $RMSD_{30}$ of 0.241 Å. Also the crystal A_{maj} was predicted at rank 2 with $RMSD_{30}$ of 0.666 Å. Analogous CSPs with conformer 4 gave A_{maj} at rank 3 with $RMSD_{30}$ of 0.630 Å and B at rank 24 with $RMSD_{30}$ of 0.361 Å. It remains unclear why these structures were not found in our original CSP search.

VII. TARGET XXXII

The target XXXII monomer contained 76 atoms and 11 soft torsional degrees of freedom (not counting the methyl group). CCDC provided information that eight polymorphs of this crystal are known. Participants were asked to submit 1,500 unranked structures.

A. Conformer search and optimization

The Avogadro package [5] was used to create an initial 3D structure of target XXXII from the 2D chemical diagram provided by CCDC. This structure was first optimized using GAFF [13] to obtain realistic bond lengths and angles and then used as the starting point in the genetic algorithm implemented in Avogadro to generate a set of plausible conformers. Two types of searches were performed, one energy-based (using GAFF) and the other using the structural RMSD diversity to score conformers. The energies of the monomers from the latter search were then computed using GAFF. No optimizations were performed at an *ab initio* level since it was clear that due to the highly flexible character of monomer XXXII, the monomer geometries generated in flexible-monomer CSPs will be significantly different from that of the *ab initio* equilibrium conformer.

To make sure that monomers with reasonable geometries are used as starting structures in CSPs, a separate procedure was performed to screen for low-energy conformers. To this end, gas phase unified free-energy dynamics (UFED) [54–56] simulations were performed using the LAMMPS package [57] combined with the PLUMED package [10, 11]. GAFF with AM1-BCC partial charges [58] was selected as the force field. The UFED scheme was used in order to enhance molecular dynamics (MD) sampling of specified torsional motions. Initial equilibration was performed using a simulated annealing scheme which gave the 'closed' conformer (indexed as the 0 ps simulation time marker, see Fig. 9). Driven dynamics with different combinations of the eleven monomer XXXII torsion angles was explored using UFED and any significant conformational mobility was

tracked by comparing the distributions of the torsion angles. The results of these trials revealed that only three out of the eleven torsions required enhanced sampling in order to capture the conformational changes deemed relevant. Most significant was a transition involving a closed and 'open' (flat) configurations of the molecule: the open configuration is the transition state between two more stable closed states (see Fig. 9). This opening and closing was not observed in UFED simulations where all eleven torsion angles were driven, with simulated annealing or with standard MD.

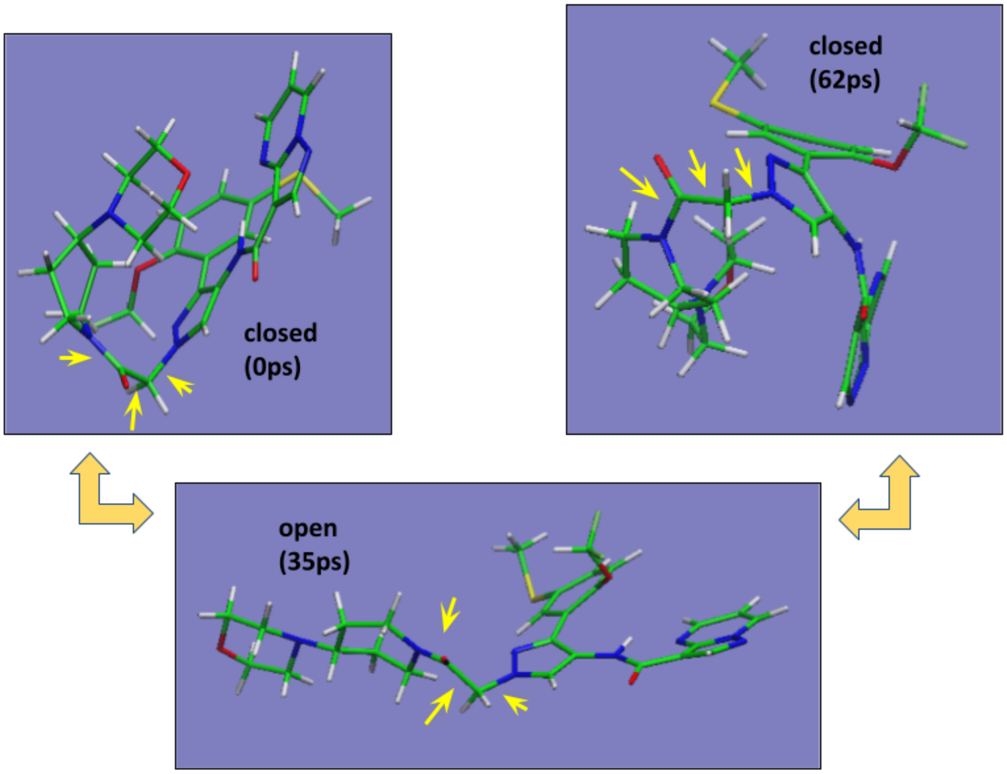

FIG. 9: Pathway of target molecule XXXII opening and closing transition observed in gas phase UFED simulations. The top images represent closed states. An open state at 35 ps (bottom) represents the transition state configuration between the two closed states (0 ps and 62 ps). Yellow arrows are used to indicate important angles that were driven.

B. Details of potential energy surface

The lowest-energy conformer found in the Avogadro search was used in the autoPES package [1, 2] to develop a 6-dimensional (rigid-monomer) PES. Due to the size of the system, we decided to use the supermolecular PBE0+D3(BJ) approach [23, 24] with the def2-TZVP [37] basis set and the counterpoise correction. SAPT(DFT) calculations would have been possible but would limit the number of grid points. The *ab initio* interaction energies were fitted by the atom-atom analytic function of Eq. (1). The polynomial term was truncated at the second order in the expression of Eq. (2) and the strongly repulsive term ($1/r_{ab}^{12}$) was used. Only the C_6^{ab}/r_{ab}^6 terms were included in Eq. (2). We limited the number of independent atom types by treating chemically-similar atoms as equivalent, i.e., having the same parameters (see Fig. 10 for the equivalences). This procedure reduced the number of sites from 76 to 20 and, hence, the number of free parameters in the fit. The long-range component of the potential was obtained with asymptotic perturbation theory consistent with SAPT(DFT) using SAPT2020 codes [3, 16] with the same functional and basis sets for the monomers as in the close-range calculations, assuring a smooth transition. After fitting, the RMSE for $E_{\text{int}} < 0$ was 0.48 kcal/mol. Such RMSE is reasonable for molecules of this size, and the ratio of RMSE to the magnitude of the global minimum energy is only 1.6%. The total number of grid points N_{grid} used was 13,852, and the total number of free parameters N_{FP} was 460, yielding $N_{\text{grid}}/N_{\text{FP}} = 30.1$.

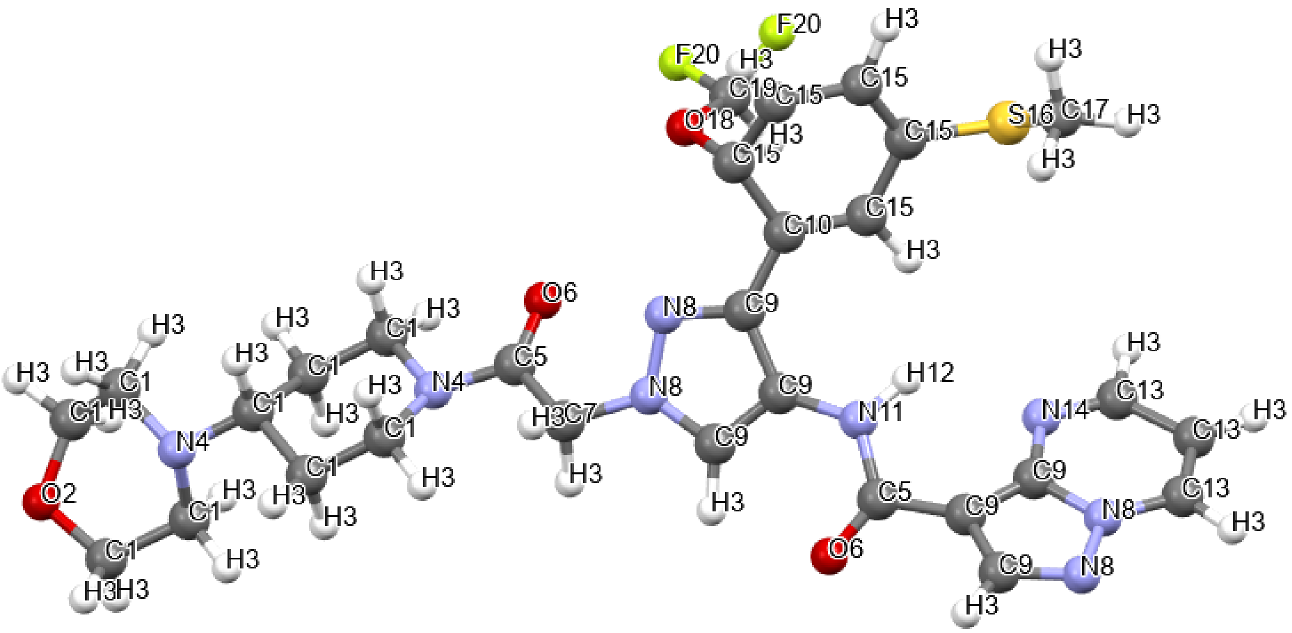

FIG. 10: Equivalences of atomic sites for monomer XXXII.

C. Crystal Structure Predictions

All CSPs for target XXXII used flexible monomers (unless noted otherwise). GAFF was applied as the all-dimensional FF (inter- and intramonomer) in the initial stages of the UPACK search. In the structure-refinement step, aiFF was applied for intermolecular and GAFF for intramolecular interactions, respectively. An initial set of 1000 structures per space group ($Z' = 1$) was generated using the quasi-random search scheme of UPACK [29] within 15 space groups: the 13 UPACK default ones plus $P2_1/a$ and $P2_1/n$. While the structures resulting from these random searches were retained, the majority of structures were generated using the EVCCPMRE algorithm [32]. Configurations identified from UFED monomer studies, as well as low-energy structures from the quasi-random UPACK searches were all useful as EVs seed for the EVCCPMRE algorithm. Because of the large number of torsional degrees of freedom, the XXXII target molecule was ideal to test flexible-molecule EVCCPMRE structure generation. EVCCPMRE was attempted for approximately 100 sets of 1000 structures per space group over 15 space groups which yielded approximately 60,000 raw structures. Feedback of EV structural information from new identified global minima into the EVCCPMRE algorithm was also performed. The results of separate collections of the EVCCPMRE generated structures over the selected space groups truncated the pool to 15,000 raw structures (1000 per space group). An analysis of these results shows that EVCCPMRE yielded a more stable global minimum, as well as better overall coverage of low energy structures in comparison to the equivalent collection of structures generated using the quasi-random method. The EVCCPMRE generated structures were appended to the candidates predicted from quasi-random UPACK searches and screened for duplicates. Unreasonably high energy structures were eliminated, yielding an intermediate data pool of over 44,000 structures which were then re-optimized (assuming rigid monomers) using the aiFF described above. For the ranking, intermolecular lattice energy contributions from the aiFF were combined with the corresponding GAFF intramolecular component.

D. Results

When experimental crystals were revealed, it turned out that out of the 8 known polymorphs, only 2 have known crystal structures. Form A exhibits disorder (forms A_{maj} and A_{min}) and there are some issues with form B, see the main text. Also, form B is $Z' = 2$, which makes searches difficult for such a large and flexible monomer. None of these three polymorphs were found, within the acceptance criteria, on our list of structures submitted to CCDC. The best matches that we could find were including only 6 monomers rather than the required 30, with $RMSD_6 = 1.62$, 2.85, and 3.28 Å for forms B (low temperature), A_{maj}, and A_{min}, respectively, with the corresponding rankings of 543, 418, and 418.

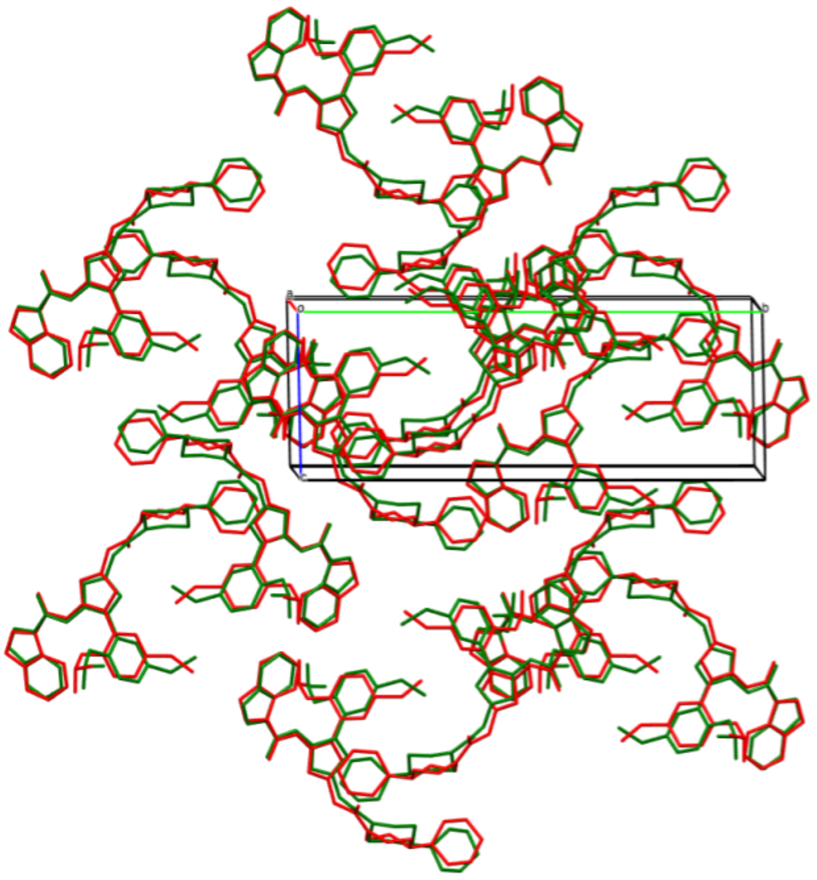

FIG. 11: Overlay of the form B room temperature experimental structure (green) of target XXXII with the GAFF/AM1-BCC optimized structure (red).

E. Post-submission analysis

The configuration of the molecule in the room temperature experimental form B was 'flat' which was identified as a high energy transition state from the vapor phase UFED simulations. When the initial ranking was performed using GAFF, the monomer in the rank #1 structure was also flat. We have compared the monomers on our submitted lists with those from the experimental structures and found the lowest RMSDs between the monomers equal to 0.65 (for #87), 0.87 (for #528), and 0.81 (for #225) Å for forms B, A_{maj}, and A_{min}, respectively. These RMSD are actually reasonably small considering that only the empirical GAFF was used to describe intramonomer interactions. This is not surprising for the monomer of target XXXII since this molecule is fairly similar to molecules of the type used in parametrizations of GAFF.

In order to evaluate the quality of our FFs, we performed a lattice-energy optimization with UP-

ACK starting from the experimental form B (space group $P2_1/c$, room temperature). First we used GAFF for both the inter- and intramonomer interactions. This CSP resulted in a packed structure and unit cell that was similar to the experimental one, however, the quantitative molecular geometry was fairly different: similarity between the experimental and GAFF optimized structures was only 13/30 molecules and $\mathrm{RMSD}_{13} = 0.581$ Å. (GAFF lattice energy is -547.170 kJ/mol, which gives a relative ranking #163 within the intermediate structure pool of EVCCPMRE/GAFF generated structures). The overlap of these structures is shown in Fig. 11. While this match is still below CCDC criteria, it is significantly better than any match on the submitted list. Thus, possible inadequacies of GAFF cannot explain the latter results. One explanation could be that our search protocol was unable to find a crystal structure reasonably similar to form B. Another explanation could be that the final optimizations with aiFF made the structures worse. To check the latter, we performed a rigid-monomer reoptimization of the GAFF-optimized form B using the aiFF. This changed the structure insignificantly: $\mathrm{RMSD}_{30} = 0.087$ Å with respect to the GAFF-optimized structure. (Interestingly, the rank was better: the lattice energy was -289.855 kJ/mol from aiFF combined with -220.883 kJ/mol GAFF-based intramolecular component to give -510.738 kJ/mol, which is a relative rank of #69 within the submitted structures. Note that the two ranks discussed above cannot be compared). Thus, the aiFF quality is not the reason for the poor predictions.

VIII. TARGET XXXIII

Target XXXIII was a cocrystal consisting of a smaller rigid positive ion of morpholine (16 atoms) that will be denoted by M and a larger negative ion of sulfamethoxazole (27 atoms) that will be denoted by S. The latter has three relevant flexible degrees of freedom. The approximate locations of charges were provided: on N in M and on the bridge N in S. The participants were told that two polymorphs of this system are known and were asked to provide a list of 1500 unranked structures.

A. Conformer search and optimization

The conformer search protocol for system XXXIII followed the same steps as that for system XXIX. The top 20 conformations of S and a single conformation of M generated by the conformer generator were optimized using PBE0 plus the D3 dispersion correction and the aug-cc-pVTZ basis set with ORCA-4.2.1. The three lowest-energy conformations are within 2 kcal/mol of the global minimum. The global-minimum conformers are shown in Fig. 12.

Since the developments of aiFFs and the initial CSPs were executed in parallel, another search for conformers was performed in the CSP thread in order to construct starting structures and to assign partial charges. The search initially took the conformations from the existing crystal struc-

tures of the sulfamethoxazole salts within the CSD structural database [49], refcode: COKROJ and DARNUI. Subsequently, each conformation was optimized using the HF method in the 3-21G* basis set [59–62] followed by a Møller-Plesset second-order (MP2) perturbation theory optimization in the 6-31G* basis set [59–62]. The Gaussian16 package [53] was used. For the morpholine cation, the chair and boat conformers were constructed using the Avogadro software [5] and then optimized in the same way. At the MP2/6-31G* level, the chair conformation is more stable than the boat conformation by over 4 kcal/mol.

B. Details of intermolecular potential energy surface

We developed three rigid-monomer intermonomer PESs: MM, SS, and MS. In all cases, only the global minimum conformers were used. Sets of 4500, 10,000, and 13,879 dimer configurations, respectively, were used for training the potentials. The interaction energies were computed with SAPT(DFT)/aug-cc-pVDZ in the MC$^+$BS form with midbond functions. The converged MS aiFF has the RMSE of 1.52 kcal/mol on the 8715 dimer configurations in the region of $E_{\mathrm{int}} < 0$. The global minimum of MS has an interaction energy of -108.2 kcal/mol and the magnitude of interaction energies at the local minima are of the order of 100 kcal/mol, therefore the ratio of RMSE to the magnitude of the *ab initio* energy is less than 1.5% at the global minimum and less than 2% for other relevant dimer configurations in the $E_{\mathrm{int}} < 0$ region. For MM and SS, the potential energy is repulsive everywhere (since they are identically charged monomers). The RMSEs on all points for MM and SS aiFFs are 2.27 kcal/mol and hundreds of kcal/mol, respectively. The reason for the very large RMSEs in the latter case will be discussed below.

C. Crystal Structure Predictions

During the first stages of flexible CSP searches, the GAFF force field [13] was used for both intermolecular and intramolecular interactions. The GAFF parameters were assigned using the Antechamber software as implemented in AmberTools20 [52]. Partial atomic point charges for this search were determined for both the anion and cation using the RESP charge assignment scheme with HF/3-21G*//MP2/6-31G* [60–62] in Gaussian16 [53]. For charge assignment for the sulfamethoxazole anion, both MP2-optimized conformations discussed above were used with Boltzmann weights at 298 K. The chair conformation was used to assign charges for the morpholine cation.

Using the GAFF intramolecular and intermolecular parameters, initial flexible-monomer CSPs were performed using the UPACK program [29] for both $Z' = 1$ and $Z' = 2$ ($Z' = 1$ here denotes an asymmetric unit containing one cations and one anion), where 30,000 random structures were generated in each of the 13 UPACK-default space groups. In contrast to other flexible-

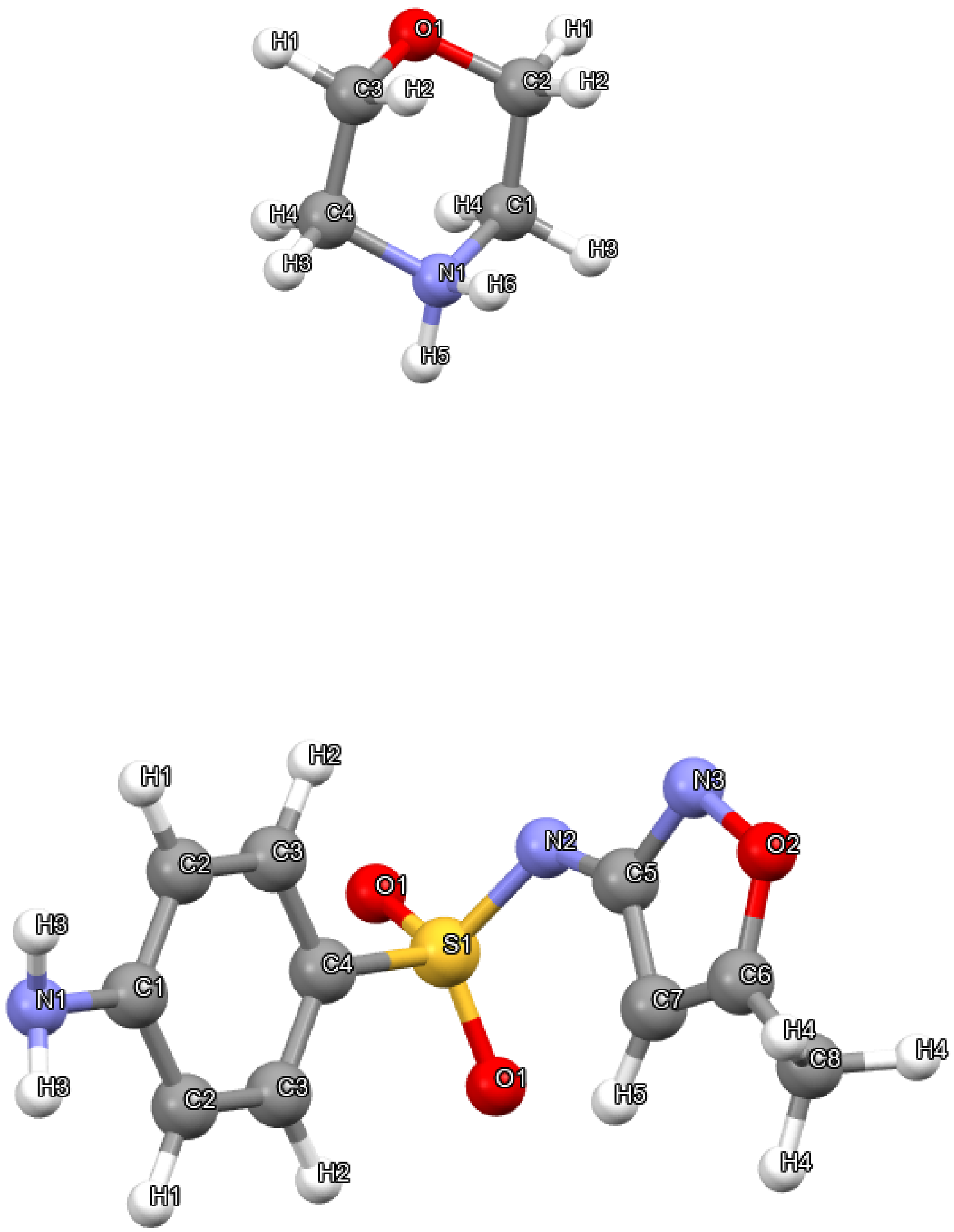

FIG. 12: Global-minimum conformers of target XXXIII.

monomer searches, we used here the capability of UPACK to perform searches using only selected intramonomer degrees of freedom: for the sulfamethoxazole anion, only the three flexible torsions in the bridge were randomly sampled during the search, while the morpholine cation was rigid in its chair conformation. The generated structures were then clustered using the 'rundist' package, as implemented in UPACK [31]. This package utilizes the radial distribution function of each atom in the asymmetric unit cell as the clustering criterion. A cutoff tolerance of 0.25 Å was used for the first round of clustering during the initial structure search, and 0.05 Å was used for the second round of clustering.

The structures resulting from the GAFF-based flexible-monomer search were then subjected to tight optimizations with rigid monomers using aiFFs. In general, in each of these structures, the geometries of monomers were different. Thus, aiFF was used in its flexibilized form.

Since the monomers in the rigid-monomer optimizations were all different, energy from intramolecular contributions has to be added for proper ranking. To address this, single-point energies of the isolated crystal monomers for the top 3000 structures were computed using PBE0/6-31G** with D3 dispersion corrections using Gaussian16, and the relative conformational energies were added to the aiFF intermolecular energies to get the total crystal lattice energy. The list of 1,500 polymorphs from this ranking was submitted to CCDC.

D. Results

There were no matches between polymorphs on our list and the two experimental polymorphs, forms A and B (one may note that form A, which was initially crystallized before the announcement of 7BT, could not be crystallized in subsequent experiments, a case of a disappearing polymorph, see the main text). This poor performance is due to the quality of the SS PES, see below.

E. Post submission analysis

The comparison of the *ab initio* optimized conformers with the experimental monomers shows that the cations are essentially identical with RMSDs of 0.016 (0.015) Å for polymorphs A (B). For the four consecutive energy-ordered anion conformers, the RMSDs are 0.092 (1.026), 1.355 (0.854), 1.079 (0.273), and 0.875 (1.052) Å. Thus, target XXXIII is an example of a crystal with flexible monomers for which reliable predictions are possible with a conformer-based CSP method. Unfortunately, our predictions failed due to the poor quality of the SS PES resulting from a flaw in autoPES: our post submission analysis found that the SS aiFF had 'holes'. This was due to the fact that the case of two identically charged monomers was overlooked in the design of the grid generation and hole-search algorithms of autoPES. In the former algorithm, the density of grid points decreases as the interaction energy increases, which for purely repulsive same-charge-

monomer surfaces resulted in insufficient density of grid points at separations important for crystal structures. The latter algorithm assumes that there are radial minima on the scans of a PES for fixed mutual orientations of monomers. Since there are no such minima on same-charge-monomer surfaces, the algorithm was not working properly and in particular was not reporting the presence of holes. This presence was actually indicated by the very large RMSE for all grid points, but this sometimes is the case for accurate PESs if a significant number of grid points is generated at very short intermonomer separations (with closest-contact atoms separated by distances well below the sum of their van der Waals radii). For the stage 2 of 7BT, the problem was rectified by refitting the SS aiFF, properly taking its same-charges characteristics into account.

[1] M. P. Metz, K. Piszczatowski, and K. Szalewicz, Automatic generation of intermolecular potential energy surfaces, J. Chem. Theory Comput. **12**, 5895 (2016).

[2] M. P. Metz and K. Szalewicz, Automatic generation of flexible-monomer intermolecular potential energy surfaces, J. Chem. Theory Comput. **16**, 2317 (2020).

[3] J. Garcia, R. Podeszwa, and K. Szalewicz, SAPT codes for calculations of intermolecular interaction energies, J. Chem. Phys. **152**, 184109 (2020).

[4] R. Nikhar and K. Szalewicz, Reliable crystal structure predictions from first principles, Nature Comm. **13**, 3095 (2022).

[5] M. D. Hanwell, D. E. Curtis, D. C. Lonie, T. Vandermeersch, E. Zurek, and G. R. Hutchison, Avogadro: an advanced semantic chemical editor, visualization, and analysis platform, J. Cheminf. **4**, 1 (2012).

[6] M. J. Vainio and M. S. Johnson, Generating conformer ensembles using a multiobjective genetic algorithm, J. Chem. Inf. Model. **47**, 2462 (2007).

[7] J. C. Cole, O. Korb, P. McCabe, M. G. Read, and R. Taylor, Knowledge-based conformer generation using the Cambridge Structural Database, J. Chem. Inf. Model. **58**, 615 (2018).

[8] C. F. Macrae, P. R. Edgington, P. McCabe, E. Pidcock, G. P. Shields, R. Taylor, M. Towler, and J. van de Streek, *Mercury*: visualization and analysis of crystal structures, J. Appl. Crystallogr. **39**, 453 (2006).

[9] N.-O. Friedrich, F. Flachsenberg, A. Meyder, K. Sommer, J. Kirchmair, and M. Rarey, Conformator: A novel method for the generation of conformer ensembles, J. Chem. Inf. Model. **59**, 731 (2019).

[10] S. Plimpton, Fast parallel algorithms for short-range molecular dynamics, J. Comp. Phys. **117**, 1 (1995).

[11] M. Bonomi, D. Branduardi, G. Bussi, C. Camilloni, D. Provasi, P. Raiteri, D. Donadio, F. Marinelli, F. Pietrucci, R. A. Broglia, and M. Parrinello, PLUMED: A portable plugin for free-energy calculations with molecular dynamics, Comput. Phys. Commun. **180**, 1961 (2009).

[12] W. L. Jorgensen and J. Tirado-Rives, The OPLS [optimized potentials for liquid simulations] potential functions for proteins, energy minimizations for crystals of cyclic peptides and crambin, J. Am. Chem. Soc. **110**, 1657 (1988).

[13] J. Wang, R. M. Wolf, J. W. Caldwell, P. A. Kollman, and D. A. Case, Development and testing of a general Amber force field, J. Comp. Chem. **25**, 1157 (2004).

[14] B. Jeziorski, R. Moszyński, and K. Szalewicz, Perturbation theory approach to intermolecular potential energy surfaces of van der Waals complexes, Chem. Rev. **94**, 1887 (1994).

[15] A. J. Misquitta, R. Podeszwa, B. Jeziorski, and K. Szalewicz, Intermolecular potentials based on symmetry-adapted perturbation theory including dispersion energies from time-dependent density

functional calculations, J. Chem. Phys. **123**, 214103 (2005).

[16] R. Bukowski, W. Cencek, P. Jankowski, M. Jeziorska, B. Jeziorski, J. Garcia, S. A. Kucharski, V. F. Lotrich, M. P. Metz, A. J. Misquitta, R. Moszyński, K. Patkowski, R. Podeszwa, F. Rob, S. Rybak, K. Szalewicz, H. L. Williams, R. J. Wheatley, P. E. S. Wormer, and P. S. Żuchowski, SAPT2020: An *ab initio* program for many-body symmetry-adapted perturbation theory calculations of intermolecular interaction energies, University of Delaware and University of Warsaw (2020), `http://www.physics.udel.edu/~szalewic/SAPT`.

[17] F. Neese, The ORCA program system, WIREs Comp. Mol. Sci. **2**, 73 (2012).

[18] F. Neese, Software update: the ORCA program system, version 4.0, WIREs Comput. Mol. Sci. **8**, e1327 (2018).

[19] R. A. Kendall, T. H. Dunning, Jr., and R. J. Harrison, Electron affinities of the first-row atoms revisited. Systematic basis sets and wave functions, J. Chem. Phys. **96**, 6796 (1992).

[20] D. E. Woon and T. H. Dunning, Jr., Gaussian basis sets for use in correlated molecular calculations. III. The atoms aluminum through argon, J. Chem. Phys. **98**, 1358 (1993).

[21] H. L. Williams, E. M. Mas, K. Szalewicz, and B. Jeziorski, On the effectiveness of monomer-, dimer-, and bond-centered basis functions in calculations of intermolecular interaction energies, J. Chem. Phys. **103**, 7374 (1995).

[22] J. P. Perdew, K. Burke, and M. Ernzerhof, Generalized gradient approximation made simple, Phys. Rev. Lett. **77**, 3865 (1996).

[23] C. Adamo and V. Barone, Toward reliable density functional methods without adjustable parameters: The PBE0 model, J. Chem. Phys. **110**, 6158 (1999).

[24] S. Grimme, J. Antony, S. Ehrlich, and H. Krieg, A consistent and accurate ab initio parametrization of density functional dispersion correction (DFT-D) for the 94 elements H-Pu, J. Chem. Phys. **132**, 154104 (2010).

[25] K. T. Tang and J. P. Toennies, An improved simple model for the van der Waals potential based on universal damping functions for the dispersion coefficients, J. Chem. Phys. **80**, 3726 (1984).

[26] F. Rob and K. Szalewicz, Distributed molecular polarisabilities and asymptotic intermolecular interaction energies, Mol. Phys. **111**, 1430 (2013).

[27] M. P. Metz, M. Shahbaz, H. Song, L. Vogt-Maranto, M. E. Tuckerman, and K. Szalewicz, Crystal structure predictions for 4-amino-2,3,6-trinitrophenol using a tailor-made first-principles-based force field, Cryst. Growth & Des. **22**, 1182 (2022).

[28] R. Nikhar and K. Szalewicz, Improving the crystal structure predictions using clusters cut from crystals, (2024), manuscript in preparation.

[29] B. P. van Eijck and J. Kroon, UPACK program package for crystal structure prediction: Force fields and crystal structure generation for small carbohydrate molecules, J. Comp. Chem. **20**, 799 (1999).

[30] C. I. Bayly, P. Cieplak, W. Cornell, and P. A. Kollman, A well-behaved electrostatic potential based method using charge restraints for deriving atomic charges: the RESP model, J. Chem. Phys. **97**, 10269 (1993).

[31] B. P. van Eijck and J. Kroon, Fast clustering of equivalent structures in crystal structure prediction, J. Comp. Chem. **18**, 1036 (1997).

[32] E. J. Chan and M. E. Tuckerman, Polymorph sampling with coupling to extended variables. Enhanced sampling of polymorph energy landscapes and free energy perturbation of polymorph ensembles, (2024) Acta Cryst. B, submitted and under revision as part of the special issue on CSPs.

[33] T. A. Halgren, Merck molecular force field. I. Basis, form, scope, parameterization, and performance of MMFF94, J. Comput. Chem. **17**, 490 (1996).

[34] T. A. Halgren, MMFF VII. Characterization of MMFF94, MMFF94s, and other widely available force fields for conformational energies and for intermolecular-interaction energies and geometries, J. Comp. Chem. **20**, 730 (1999).

[35] J. H. Holland, *Adaptation in Natural and Artificial Systems* (University of Michigan Press, Ann Arbor, MI, USA, 1975).

[36] S. Grimme, Density functional theory with London dispersion corrections, WIREs Comput. Mol. Sci. **1**, 211 (2011).

[37] F. Weigend and R. Ahlrichs, Balanced basis sets of split valence, triple zeta valence and quadruple zeta valence quality for H to Rn: Design and assessment of accuracy, Phys. Chem. Chem. Phys. **7**, 3297 (2005).

[38] K. A. Peterson, D. Figgen, E. Goll, H. Stoll, and M. Dolg, Systematically convergent basis sets with relativistic pseudopotentials. II. Small-core pseudopotentials and correlation consistent basis sets for the post-d group 16–18 elements, J. Chem. Phys. **119**, 11113 (2003).

[39] L. S. Dodda, I. Cabeza de Vaca, J. Tirado-Rives, and W. L. Jorgensen, LigParGen web server: an automatic OPLS-AA parameter generator for organic ligands, Nucleic Acids Res. **45**, W331 (2017).

[40] P. J. P. de Oliveira, C. L. Barros, F. E. Jorge, A. Canal Neto, and M. Campos, Augmented Gaussian basis set of double zeta valence quality for the atoms Rb and Y–Xe: Application in DFT calculations of molecular electric properties, J. Mol. Struct. (Theochem) **948**, 43 (2010).

[41] D. C. Spellmeyer, A. K. Wong, M. J. Bower, and J. M. Blaney, Conformational analysis using distance geometry methods, J. Mol. Graph. Model. **15**, 18 (1997).

[42] A. K. Rappé, C. J. Casewit, K. S. Colwell, W. A. Goddard III, and W. M. Skiff, UFF, a full periodic table force field for molecular mechanics and molecular dynamics simulations, J. Am. Chem. Soc. **114**, 10024 (1992).

[43] Peakfit v4 - scientific software for chromatographic peak analysis. (2007), `https://systatsoftware.com/peakfit/`.

[44] R. de Gelder, R. Wehrens, and J. A. Hageman, A generalized expression for the similarity of spectra: application to powder diffraction pattern classification, J. Comput. Chem. **22**, 273 (2001).

[45] S. Kullback and R. A. Leibler, On information and sufficiency, Ann. Math. Stat. **22**, 79 (1951).

[46] E. J. Chan, A. G. Shtukenberg, M. E. Tuckerman, and B. Kahr, Crystal structure prediction as a tool for identifying components of disordered structures from powder diffraction: A case study of benzamide II, Crystal Growth & Design **21**, 5544 (2021).

[47] T.-Q. Yu and M. E. Tuckerman, Temperature-accelerated method for exploring polymorphism in molecular crystals based on free energy, Phys. Rev. Lett. **107**, 015701 (2011).

[48] W. L. Jorgensen, D. S. Maxwell, and J. Tirado-Rives, Development and testing of the OPLS all-atom force field on conformational energetics and properties of organic liquids, J. Am. Chem. Soc. **118**, 11225 (1996).

[49] C. R. Groom, I. J. Bruno, M. P. Lightfoot, and S. C. Ward, The Cambridge Structural Database, Acta Cryst. B **72**, 171 (2016).

[50] M. Clark, R. D. Cramer III, and N. Van Opdenbosch, Validation of the general purpose tripos 5.2 force field, J. Comp. Chem. **10**, 982 (1989).

[51] L. Heinzerling, R. Klein, and M. Rarey, Fast force field-based optimization of protein–ligand complexes with graphics processor, J. Comp. Chem. **33**, 2554 (2012).

[52] D. A. Case, K. Belfon, I. Y. Ben-Shalom, S. R. Brozell, D. S. Cerutti, T. E. Cheatham, III, V. W. D. Cruzeiro, T. A. Darden, R. E. Duke, G. Giambasu, M. K. Gilson, H. Gohlke, A. W. Goetz, R. Harris, S. Izadi, S. A. Izmailov, K. Kasavajhala, A. Kovalenko, R. Krasny, T. Kurtzman, T. S. Lee, S. LeGrand, P. Li, C. Lin, J. Liu, T. Luchko, R. Luo, V. Man, K. M. Merz, Y. Miao, O. Mikhailovskii, G. Monard, H. Nguyen, A. Onufriev, F. Pan, S. Pantano, R. Qi, D. R. Roe, A. Roitberg, C. Sagui, S. Schott-Verdugo, J. Shen, C. L. Simmerling, N. R. Skrynnikov, J. Smith, J. Swails, R. C. Walker, J. Wang, L. Wilson, R. M. Wolf, X. Wu, Y. Xiong, Y. Xue, D. M. York, and P. A. Kollman, AMBER 2020, University of California, San Francisco.

[53] M. J. Frisch, G. W. Trucks, H. B. Schlegel, G. E. Scuseria, M. A. Robb, J. R. Cheeseman, G. Scalmani, V. Barone, G. A. Petersson, H. Nakatsuji, X. Li, M. Caricato, A. V. Marenich, J. Bloino, B. G. Janesko, R. Gomperts, B. Mennucci, H. P. Hratchian, J. V. Ortiz, A. F. Izmaylov, J. L. Sonnenberg, D. Williams-Young, F. Ding, F. Lipparini, F. Egidi, J. Goings, B. Peng, A. Petrone, T. Henderson, D. Ranasinghe, V. G. Zakrzewski, J. Gao, N. Rega, G. Zheng, W. Liang, M. Hada, M. Ehara, K. Toyota, R. Fukuda, J. Hasegawa, M. Ishida, T. Nakajima, Y. Honda, O. Kitao, H. Nakai, T. Vreven, K. Throssell, J. A. Montgomery, Jr., J. E. Peralta, F. Ogliaro, M. J. Bearpark, J. J. Heyd, E. N. Brothers, K. N. Kudin, V. N. Staroverov, T. A. Keith, R. Kobayashi, J. Normand, K. Raghavachari, A. P. Rendell, J. C. Burant, S. S. Iyengar, J. Tomasi, M. Cossi, J. M. Millam, M. Klene, C. Adamo, R. Cammi, J. W. Ochterski, R. L. Martin, K. Morokuma, O. Farkas, J. B. Foresman, and D. J. Fox, Gaussian 16 Revision C.01 (2016), Gaussian Inc. Wallingford CT.

[54] M. Chen, M. A. Cuendet, and M. E. Tuckerman, Heating and flooding: A unified approach for rapid generation of free energy surfaces, J. Chem. Phys. **137**, 024102 (2012).

[55] A. Barducci, G. Bussi, and M. Parrinello, Well-tempered metadynamics: A smoothly converging and tunable free-energy method, Phys. Rev. Lett. **100**, 020603 (2008).

[56] J. B. Abrams and M. E. Tuckerman, Efficient and direct generation of multidimensional free energy surfaces via adiabatic dynamics without coordinate transformations, J. Phys. Chem. B **112**, 15742 (2008).

[57] A. P. Thompson, H. M. Aktulga, R. Berger, D. S. Bolintineanu, W. M. Brown, P. S. Crozier, P. J. in 't Veld, A. Kohlmeyer, S. G. Moore, T. D. Nguyen, R. Shan, M. J. Stevens, J. Tranchida, C. Trott, and S. J. Plimpton, LAMMPS — a flexible simulation tool for particle-based materials modeling at the atomic, meso, and continuum scales, Comp. Phys. Comm. **271**, 108171 (2022).

[58] A. Jakalian, D. B. Jack, and C. I. Bayly, Fast, efficient generation of high-quality atomic charges. AM1-BCC model: II. Parameterization and validation, J. Comput. Chem. **23**, 1623 (2002).

[59] R. Ditchfield, W. J. Hehre, and J. A. Pople, Self-Consistent Molecular-Orbital Methods. IX. An Extended Gaussian-Type Basis for Molecular-Orbital Studies of Organic Molecules, J. Chem. Phys. **54**, 724 (1971).

[60] J. S. Binkley, J. A. Pople, and W. J. Hehre, Self-consistent molecular orbital methods. 21. Small split-valence basis sets for first-row elements, J. Am. Chem. Soc. **102**, 939 (1980).

[61] M. J. Frisch, M. Head-Gordon, and J. A. Pople, A direct MP2 gradient method, Chem. Phys. Lett. **166**, 275 (1990).

[62] G. A. Petersson and M. A. Al-Laham, A complete basis set model chemistry. II. Open-shell systems and the total energies of the first-row atoms, J. Chem. Phys. **94**, 6081 (1991).

21. Group 28

Supplementary Information

Group 28

1, Structure Generation

We generated 100000 structures by randomly picking space groups from the given list. The structures were either randomly generated from the scratch or from the mutation of the parent structures (through PyXtal v0.3.5).

2, Optimization and Ranking

For each structure, we first optimize its geometry with the GAFF force field through the CHARMM software, and then refine the energy ranking based on DFTB+ through the ASE software. No DFT energy correction was considered due to high computational cost.

3. Selection

At the final stage of structure selection, we use the PyMatgen StructureMatcher utility to detect the duplicate structures after the removal of hydrogen. The tolerance values are 0.3 Angstrom for site, 0.2 for fractional coordinates, 5 degree for cell angles.

For target XXVII submission 1, we considered 1500 structures based on energy ranking

For target XXIX submission 1, we considered 199 structures based on energy ranking

For target XXIX submission 2, we considered 10 structures based on PXRD match from the list of submission 1

For target XXXI submission 1, we considered 1500 structures based on energy ranking

For target XXXII submission 1, we considered 1495 structures based on energy ranking

For target XXXIIII submission 1, we considered 1453 structures based on energy ranking

Computational Hardware

In-house workstation with 64 cores (Intel(R) Xeon(R) CPU E5-2697A v4 @ 2.60GHz)
