# Supplementary material for: The seventh blind test of crystal structure prediction: structure generation methods
Source: Acta Crystallogr B Struct Sci Cryst Eng Mater. 2024 Dec 1;80(Pt 6):517–47. doi: 10.1107/S2052520624007492 (PMC11789161; doi:10.1107/S2052520624007492)
Supplement: Supplementary file 3 [file b-80-00517-sup3.pdf]

Supplementary Information C (Experimental reports). The Seventh Blind Test of Crystal Structure Prediction: Structure Generation Methods

Lily M. Hunnisett, *et al.* *

The Cambridge Crystallographic Data Centre, 12 Union Road, Cambridge CB2 1EZ, UK. E-mail: lhunnisett@ccdc.cam.ac.uk

1. *

1 Contents* 2

2 Executive Summary - XXVII 3

3 Executive Summary - XXVIII 12

4 Executive Summary - XXIX 20

5 Executive Summary - XXX 24

6 Executive Summary - XXXI 37

7 Executive Summary - XXXII 41

8 Executive Summary - XXXIII 123

2. Executive Summary - XXVII

The solid form screening of XXVII, commissioned during the test, yielded at least 5 different crystalline forms, named Groups A–E (of which Group A corresponded to the known Form A from the main paper). The XXVII materials, were produced by conventional solution crystallisation from a total of *ca* 150 experiments. The identity of A–C and E was confirmed by XRPD. Groups A–C are neat polymorphs, C being produced by desolvating a 1,4-dioxane solvate (Group E). Group D material remains an unconfirmed form, having been isolated once concomitantly with A and B.

The relative stability of Group A, B and D materials was established qualitatively through slurry bridging in two different solvents. Group A material was shown to be the most stable at 5°C and enantiotropically related to Group B, the predominant product of the solid form screen. Group B was shown to be most stable between 20 and 40°C.

Attempts by CCDC scientists and blind test participant groups at determining the structure of Group B, through indexing the PXRD pattern and using PXRD comparison methods against CSP structures, were unsuccessful.

The potential for structural (static or dynamic) disorder in the different crystal forms of XXVII, and the impact of disorder on the physical stability of the forms, was not addressed as part of the characterisation supporting the experimental solid form screen.

Target XXVII (2,3-diiodo-6,13-bis(triisopropylsilylethynyl) pentacene) - Crystal structure report

Sean Parkin and John Anthony
Department of Chemistry, University of Kentucky

Silylethyne-functionalized pentacenes have seen extensive use in the fields of organic electronics and photonics over the last two decades, due to their improved stability, increased solubility, and the ability to tune crystal packing by varying the structure of the silyl group.[1] Along with their direct use in materials applications, these pentacenes are also used as building blocks to generate semiconducting polymers[2] and to form oligomeric species for studies of useful photophysical properties.[3] 2,3-diiodo-6,13-bis(triisopropylsilylethynyl) pentacene (Diiodo TIPS Pent) was prepared as just such a precursor – elaboration of the iodine functional groups to cyano groups converted the chromophore to an acceptor for use in blends with semiconducting polymers to yield thin-film organic photovoltaics.[4] The material is prepared from known diiodo diethyl phthalate, via reduction to the corresponding dimethanol, oxidation to the dialdehyde, and condensation with 1,4-dihydroxyanthracene. Addition of lithiated triisopropylsilylacetylene followed by reductive deoxygenation yields the crude pentacene. As with the majority of silylethyne functionalized pentacenes, purification is most easily accomplished by recrystallization, in this case twice from hexanes, to yield samples suitable for crystallographic analysis.

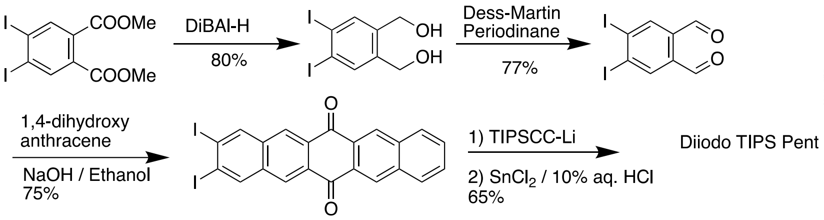

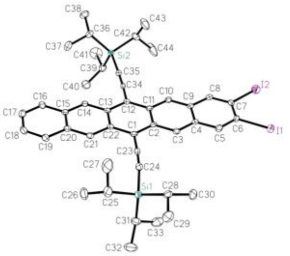

X-ray diffraction data for Diiodo TIPs Pent were collected at 90.0(2)K on a Nonius kappaCCD diffractometer using Mo*K*□ X-rays from a small blue plate-shaped crystal (0.15 x 0.10 x 0.03 mm) grown from solution in CH_2Cl_2. Raw data were integrated, scaled, merged and corrected for Lorentz-polarization effects using the HKL-SMN package.[5] Corrections for absorption were applied using XABS2.[6] The structure was solved by direct methods (SHELXS,[7]) and refinement was carried out against F^2 by weighted full-matrix least-squares (SHELXL,[7]). Hydrogen atoms were found in difference maps but subsequently placed at calculated positions and refined using a riding model. Non-hydrogen atoms were refined with anisotropic displacement parameters. Atomic scattering factors were taken from the International Tables for Crystallography (D).[8]

---

[1] (a) J.E. Anthony, D.L. Eaton, S.R. Parkin, "A road map to stable, soluble, easily crystallized pentacene derivatives," *Organic Letters* 4, 15-18, **2002**. 10.1021/ol0167356; (b) J. E. Anthony and A. G. Jones "Silyletnyne-substituted acenes and heteroacenes for organic electronics" in *Organic Electronics II – an Industrial Perspective* (H. Klauk, Ed.) Wiley-VCH, Berlin, April 2012.

[2] D. Lehnherr, R.R. Tykwinski, "Pentacene Oligomers and Polymers: Functionalization of Pentacene to Afford Mono-, Di-, Tri-, and Polymeric Materials," *Organic Letters* 9, 4583-4586, **2007**. 10.1021/ol702094d.

[3] C. Hetzer, D.M. Guldi, R.R. Tykwinski, "Pentacene Dimers as a Critical Tool for the Investigation of Intramolecular Singlet Fission," *Chemistry - A European Journal* 24, 8245-8257, **2018**. 10.1002/chem.201705355

[4] Y.-F. Lim, Y. Shu, S.R. Parkin, J.E. Anthony, G.G. Malliaras, "Soluble n-type pentacene derivatives as novel acceptors for organic solar cells," *Journal of Materials Chemistry* 19, **2009**. 10.1039/b818693f

[5] Z. Otwinowski & W. Minor (1997) "Processing of x-ray diffraction data collected in oscillation mode" Methods in Enzymology Volume 276: Macromolecular Crystallography part A, pp307-326. C.W. Carter, Jr. & R.M. Sweet, Eds., Academic Press.

[6] S. Parkin, B. Moezzi, H. Hope, "XABS2: an empirical absorption correction program," *Journal of Applied Crystallography* 28, 53-56, **1995**. 10.1107/s0021889894009428

[7]G.M. Sheldrick, "A short history ofSHELX," *Acta Crystallographica Section A Foundations of Crystallography* 64, 112-122, **2007**. 10.1107/s0108767307043930.

[8] International Tables for Crystallography, vol C: Mathematical, Physical and Chemical Tables. A.J.C. Wilson, Ed. (1992). Kluwer Academic Publishers, Holland.

Target XXVII (2,3-diiodo-6,13-bis(triisopropylsilylethynyl)pentacene) – Polymorph screen

Joanna Bis, Stephen Carino, Ricky Couch, Frank Tarczynski

Catalent Pharma Solutions, Morrisville, NC, USA

To support the findings of the blind test a crystal-form screen was conducted on 2,3-diiodo-6,13-bis(triisopropylsilylethynyl)pentacene. Herein the polymorph screen results are presented.

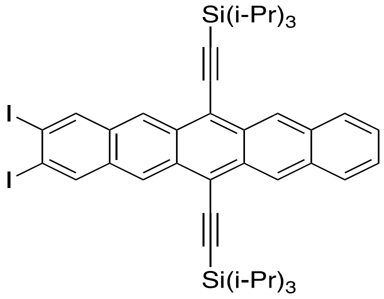

Chemical Formula: $C_{44}H_{52}I_2Si_2$
Exact Mass: 890.17
Molecular Weight: 890.88

Figure 1 - Molecular Structure, Weight and Formula of 2,3-diiodo-6,13-bis(triisopropylsilylethynyl)pentacene

1. PREPARATION OF 2,3-DIIODO-6,13-BIS(TRIISOPROPYLSILYLETHYNYL)PENTACENE

(Carried out by University of Kentucky)

Table 1 - Estimated Solubility of 2,3-diiodo-6,13-bis(triisopropylsilylethynyl)pentacene Group A

	Solvent (v/v)	Solubility at RT (mg/mL)	Solubility at 50°C (mg/mL)
1	Tetrahydrofuran	102-408	NA
2	Chloroform	94-376	NA
3	Toluene	55-110	NA
4	p-Xylene	51-102	NA
5	Dichloromethane	49-97	NA
6	Chlorobenzene	47-94	NA
7	Anisole	10-13	NA
8	Cyclohexanone	10-13	NA
9	4-Methylpentan-2-one	6-10	NA
10	Cyclohexane	6-10	NA
11	Ethyl acetate	1-6	NA
12	t-Butyl methyl ether	1-6	NA

13	2-Butanone	1-6	NA
14	Diisopropyl ether	1-6	NA
15	1,4-Dioxane	1-5	NA
16	Isopropyl acetate	1-5	NA
17	Heptane	>6	>6
18	Acetone	<1	>1
19	Water	<1	<1
20	Methanol	<1	<1
21	Acetonitrile	<1	<1
22	Dimethylsulfoxide	<1	<1
23	2-Propanol	<1	<1
24	2-Propanol:water (9:1)	<1	<1

NA: Not applicable

2. CRYSTAL-FORM SCREEN OF 2,3-DIIODO-6,13-BIS(TRIISOPROPYLSILYLETHYNYL)PENTACENE

The crystal-form screen was comprised of ~150 crystallization experiments and involved 48 solvents, three crystallization modes (temperature-cycling slurry ripening, rapid cooling and slow evaporation), and a temperature range of 5-40 °C. Group A was utilized as the input form.

2.1. Results of Crystal-Form Screen

The crystal-form screen of 2,3-diiodo-6,13-bis(triisopropylsilylethynyl)pentacene produced five crystal forms.

Group A is a nonsolvated crystal form consistent with the supplied material. DSC data shows a broad endotherm at 96 °C (ΔH=2 J/g), a broad endotherm at 203 °C (ΔH=2 J/g) and an exotherm at 256 °C (ΔH=3 J/g) followed immediately by an exotherm at 267 °C (ΔH=8 J/g). TGA analysis shows negligible weight loss up to 230 °C.

Group B is a new nonsolvated crystal form. DSC data shows a broad endotherm at 129 °C (ΔH=3 J/g) and a small endotherm at 277 °C (ΔH=1 J/g) followed immediately by an exotherm at 283 °C (ΔH=19 J/g). TGA analysis shows negligible weight loss up to 250 °C.

Group C is a new desolvated 1,4-dioxane solvate. DSC data shows a broad endotherm at 46 °C (ΔH=3 J/g), a broad exotherm at 253 °C (ΔH=1 J/g) and a broad exotherm at 276 °C (ΔH=9 J/g). TGA analysis of batch 103884-TC-017 shows negligible weight loss up to 250 °C.

Group D is a new crystal form of unknown nature and was observed from a single evaporation experiment (chlorobenzene/cyclohexane, 2:8 v:v) as a mixture with Groups A and B.

Group E is a new transient 1,4-dioxane solvate and desolvates to Group C within one day in the solid-state under ambient conditions. DSC data shows a broad endotherm at 53 °C (ΔH=56 J/g) and a small endotherm at 279 °C (ΔH=1 J/g) followed immediately by an exotherm at 284 °C (ΔH=21 J/g). TGA analysis shows 8.2% weight loss (0.9 eq) of 1,4-dioxane up to 200 °C.

PXRD patterns of Groups A-E are shown in Figure 2.

2.2. Preparation of Group B

Group B was the dominant form of the screening study, as evidenced by the 37 out of 48 slurry products. Group B can be easily produced by Stirring suspension of Group A while cycling the temperature between 40-5 °C for up to 72 hours in solvents such us acetone, methyl acetate, 2-propanol, etc.

2.3. Relative Stability Studies

Relative thermodynamic stability of nonsolvated Groups A and B and Group D was determined via competitive ripening studies in 4-methylpentan-2-one and anisole at 5, 20 and 40 °C.

The competitive ripening results are summarized in Table 2 and indicate that Group A is the more stable form at 5 °C, and Group B is the more stable form at 20 and 40 °C.

The results suggest that Groups A and B exhibit an enantiotropic relationship with a transition temperature between 5-20 °C.

Table 2 - Results of Relative Stability Study

#	Temperature (°C)	Solvent	Group to Saturate	Ripened Groups	Group at t=3 d
1	5	4-Methylpentan-2-one	A	A, B, D	A
2	5	Anisole	A	A, B, D	A
3	20	4-Methylpentan-2-one	A	A, B, D	B
4	20	Anisole	A	A, B, D	B
5	40	4-Methylpentan-2-one	A	A, B, D	B
6	40	Anisole	A	A, B, D	B

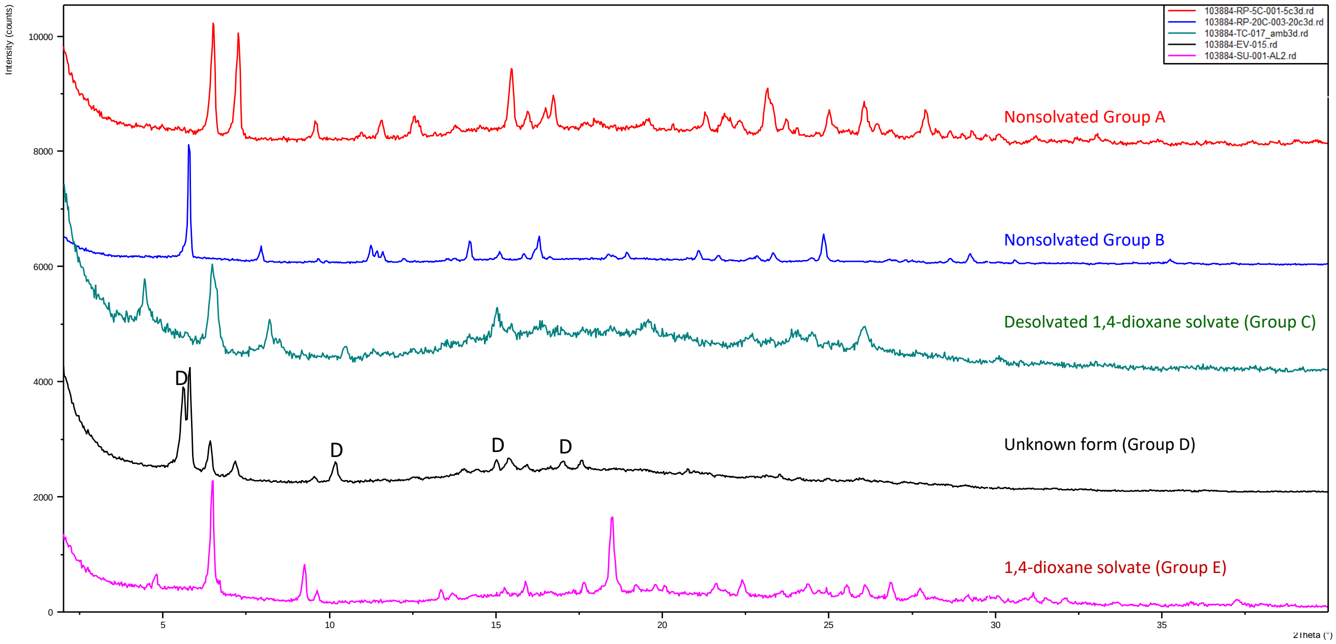

Figure 2 - PXRD Patterns of Groups A - E

Materials and Methods

2,3-diiodo-6,13-bis(triisopropylsilylethynyl)pentacene (lot IPT1) was provided by the University of Kentucky and used in this screening study without further purification or processing. The supplied material was reported as 99% pure. The supplied crystal form was designated as Group A.

Analysis of Crystal-Form Screening Products

FT-Raman spectroscopy was chosen as the primary method for analysis and grouping of samples. Representative samples from the groupings were analyzed by PXRD to verify their uniqueness. Where possible/practical, a representative sample of the unique group was further characterized by PLM, DSC, TGA and/or TGA-IR.

Solubility Assessment

Solubility of 2,3-diiodo-6,13-bis(triisopropylsilylethynyl)pentacene Group A (lot IPT1) was visually assessed in 24 diverse solvents at 20 °C and 50 °C to support the selection of solvents and corresponding dosing strategies for the subsequent screening experiments. The solubility was visually estimated at 20 °C by dosing small aliquots of solvent into a fixed amount of solid (~10 mg) until the dissolution point or a maximum volume (1.8 mL) was reached. Samples that contained undissolved solids at RT were heated to 50 °C for 1 h and the dissolution was assessed visually.

Relative Stability Studies

Saturated suspensions were prepared by stirring an excess of Group A in the solvents at 5, 20 and 40 °C for 18 h. The suspensions were filtered at the test temperature and the filtrates were combined with equal amounts of Groups A, B and D, as the sample quantity permitted. The suspensions were stirred at the test temperatures and aliquots were withdrawn periodically and analyzed by PXRD until a complete form conversion was achieved, or up to 3 days.

Instrumentation

Polarized-Light Microscopy (PLM). The photomicrographs were collected using Olympus BX60 polarized-light microscope equipped with Olympus DP70 camera or Olympus BX51 polarized-light microscope equipped with Olympus DP71 camera.

Powder X-Ray Diffraction (PXRD) Bruker. PXRD diffractograms were acquired on a Bruker D8 Advance system using Cu Ka (40 kV/40 mA) radiation and a step size of 0.03° 2θ and LynxEye detector. Configuration on the incident beam side: Goebel mirror, mirror exit slit (0.2 mm), 2.5 deg Soller slits, beam knife. Configuration on the diffracted beam side: anti-scatter slit (8 mm) and 2.5 deg. Soller slits. Samples were mounted flat on zero-background Si wafers.

Differential Scanning Calorimetry (DSC). DSC was conducted with a TA Instruments Q100 or Q2000 differential scanning calorimeter equipped with an autosampler and a refrigerated cooling system under 40 mL/min N_2 purge. DSC thermograms of samples were obtained at 10 °C/min or 15 °C/min in crimped Al pans, unless noted otherwise. The temperatures of exothermic and endothermic transitions recorded via DSC analysis are reported as onset values.

Thermogravimetric Analysis (TGA). TGA thermograms were obtained with a TA Instruments Q50 thermogravimetric analyzer under 40 mL/min N_2 purge for balance and 60 mL/min for sample in Al pans. TGA thermograms of samples were obtained at 10 °C/min or 15 °C/min, unless noted otherwise.

Thermogravimetric Analysis with IR Off-Gas Detection (TGA-IR). TGA-IR was conducted with a TA Instruments Q5000 thermogravimetric analyzer interfaced to a Nicolet 6700 FT-IR spectrometer (Thermo Electron) equipped with an external TGA-IR module with a gas flow cell and DTGS detector. TGA was conducted with 25 mL/min N_2 flow for sample purge, 10 mL/min N_2 flow for balance purge, and heating rate of 15 ^oC/min in Al pans. IR spectra were collected at 4 cm^{-1} resolution and 32 scans at each time point.

3. Executive Summary - XXVIII

A crystallisation screen of XXVIII was attempted using the Encapsulated Nanodroplet Crystallization (ENaCt) platform. Oxidative dimerization of the ligand was observed from all combinations of 20 different solvents and 4 oils. The limited scope (and success rate) of the crystallisation screening means that conclusions cannot be drawn as to whether the only known structure likely represents the thermodynamically stable form of XXVIII.

Target XXVIII (DichloroCopper(II)bis-diphenylmethanimine) – Crystal structure report

Mark R.J. Elsegood, Paul F. Kelly, and Luke A. Wilkinson.
Chemistry Department, Loughborough University, Loughborough, LE11 3TU
m.r.j.elsegood@lboro.ac.uk & p.f.kelly@lboro.ac.uk

The compound $C_{26}H_{22}Cl_2CuN_2$, (**1**), Mr = 496.90, crystallises in the triclinic crystal system with cell parameters *a* = 5.2612(9), *b* = 9.6022(16), *c* = 12.268(2) Å, α = 111.642(2), β = 91.090(2), γ = 99.961(2)°, *V* = 565.07(16) Å^3, D_{calc} = 1.460 g/cm^3. The data were collected at 150 K on a Bruker Apex 2 CCD diffractometer equipped with Mo-Kα radiation.[1] The structure was solved by direct methods[2] and refined on F^2 values to give $R1$ = 0.035 {for 3021 data with $I > 2\sigma(I)$} and $wR2$ = 0.101 {for all 3419 independent reflections}.[3] The structure was well-behaved and did not exhibit any noticeable disorder or twinning effects, and did not co-crystallise with any solvent molecules.

Half the molecule comprises the asymmetric unit since the Cu is on an inversion centre (Figure 1). H atoms on carbon atoms were constrained using a riding model, but that on the nitrogen was freely refined with U_{iso}. Molecules stack into columns parallel with the *a* axis, with a Cu···Cl separation of 4.087 Å meaning the Cu has a fundamentally square planar coordination environment but with an additional two *trans* contacts above and below that plane. It is a substantially distorted 4+2 environment with Cu–Cl···Cu' = 108°. The N*H* does not engage in any meaningful intramolecular H-bond, but instead very weakly H-bonds to the Cl in an adjacent molecule, though this may be a consequential contact from the extra Cu···Cl interactions described above (Figures 2 & 3).

The structure was published by the Redshaw group after the start of the structure prediction exercise.[4]

Synthesis: Benzophenone imide (0.270 g, 1.49 mmol) was added dropwise to anhydrous copper(II) chloride (0.100 g, 0.74 mmol) in degassed acetonitrile (60 mL) under a flow of N_2. The reaction was stirred at room temperature until the yellow/brown solution changed to pale blue and a purple solid had precipitated. The precipitate was isolated and dried *in vacuo* to give a lilac powder. Yield: 0.346 g

(94%). The compound was crystallised via vapour diffusion of diethyl ether into a dichloromethane solution of the compound, yielding purple/blue laths.

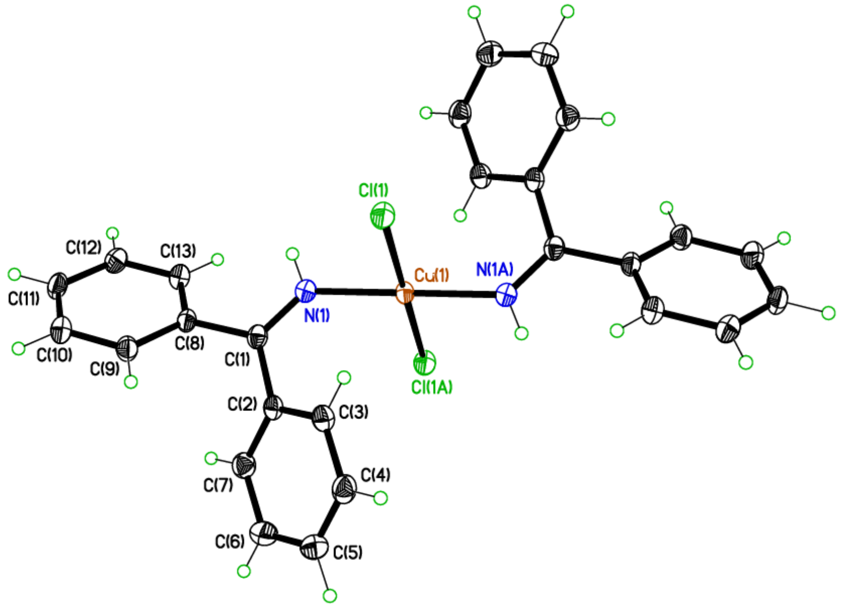

Figure 1. Displacement ellipsoid plot of **1** at the 50% probability level showing the square-planar Cu(II) geometry.

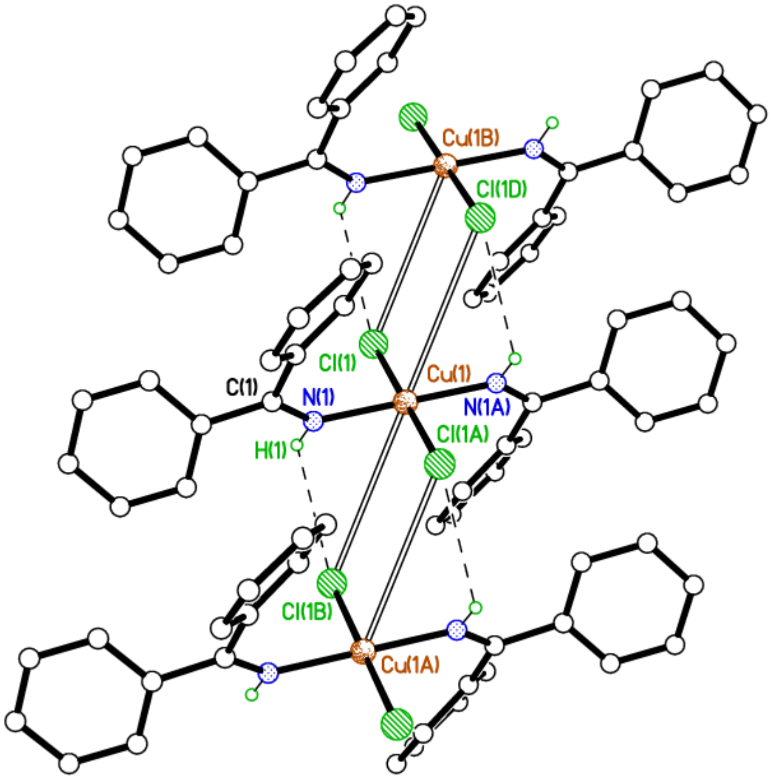

Figure 2. Stacked molecules of **1** showing the weak intermolecular Cu···Cl interactions at 4.087 Å and weak intermolecular H-bonds H(1)···Cl(1B) = 2.977(18) Å.

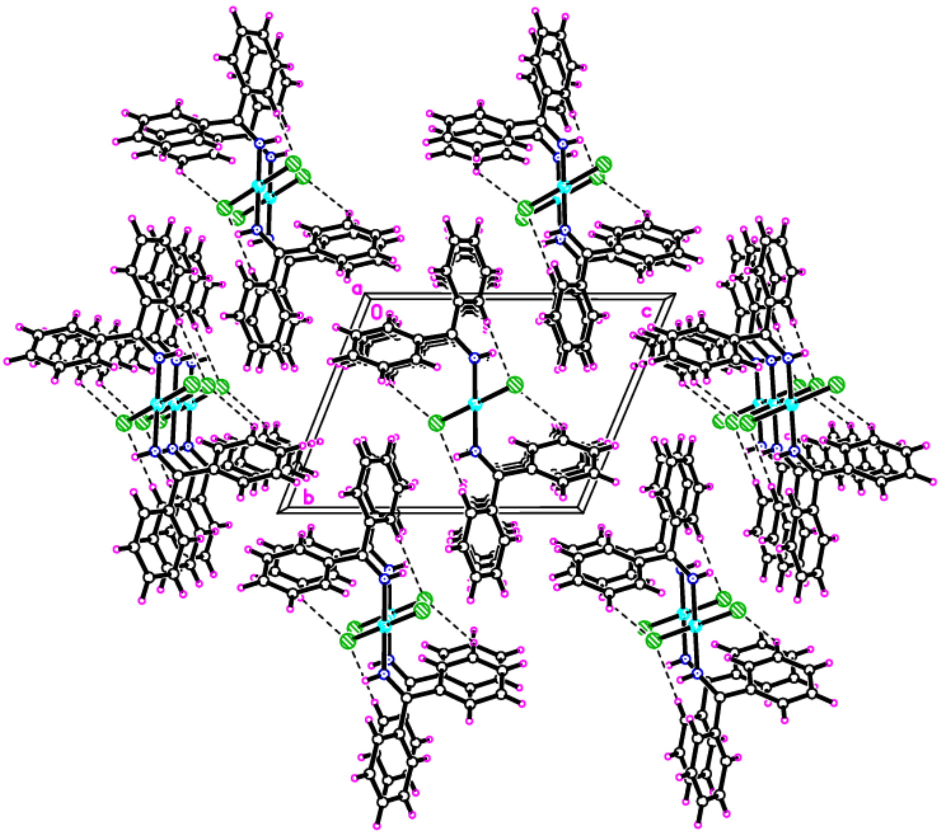

Figure 3. Packing plot of **1** viewed parallel to *a* showing molecules stacked in that direction.

References.

1. APEX 2 and SAINT (2008) software for CCD diffractometers. Bruker AXS Inc., Madison, USA.

2. G.M. Sheldrick, *Acta Cryst*. (2008), **A64**, 112-122.

3. G.M. Sheldrick, *Acta Cryst*. (2015), **C71,** 3-8.

4. A.F.A. Alshamrani, O. Santoro, S. Ounsworth, T.J. Prior, G.J. Stasiuk, and C. Redshaw, Polyhedron, (2021), **195**, 114977.

XXVIII – Polymorph screen report

Michael Probert, Jake Weatherston

School of Natural and Environmental Sciences, Newcastle University

ENaCt crystallisation screening for $CuCl_2(HNCPh_2)_2$:

ENaCt experiments were carried out using 20 different organic solvents in combination with 4 inert oils (plus no oil).

Solutions of $CuCl_2(HNCPh_2)_2$ were prepared by serial dilution. Using an SPT Labtech Mosquito, 50 nL droplets of $CuCl_2(HNCPh_2)_2$ were dispensed into 100 nL oil droplets inside 96-well crystallisation plates and sealed with a glass cover slip.

After two weeks crystallisation was assessed by cross-polarised optical microscopy, suitable crystals were then harvested for unit cell determination.

**Solvents used**		**Oils used**
DMSO	Dibutyl formamide	PDMSO
DMF	4 formyl morpholine	Fc-40
MeOH	1 formyl pyrrolidine	Mineral Oil
222 trifluroethanol	2 Nitroethanol	Fomblin YR-1800
Dichloroethane	1 methyl imidazole	
2 methyl THF	n-methyl formanilide	
1-4 Dioxane	Benzonitrile	
MeCN	Adiponitrile	
1-3 Dioxane		
Glycol sulfite		
Diethyl sulfoxide		
Dimethyl sulfite		

**P1**	**Solvent**	**Concentration mg/mL**
	DMSO	69.4
	DMF	41.7
	MeOH	34.7
	222 trifluroethanol	17.4

**P2**	**Solvent**	**Concentration mg/mL**
	DMSO	34.7
	DMF	20.8
	MeOH	17.4
	222 trifluroethanol	8.7

**P3**	**Solvent**	**Concentration mg/mL**
	DMSO	17.4

	DMF	10.4
	MeOH	8.7
	222 trifluroethanol	5.0

P4	Solvent	Concentration mg/mL
	Dichloroethane	26.0
	2 methyl THF	26.0
	1-4 Dioxane	26.0
	MeCN	26.0

P5	Solvent	Concentration mg/mL
	Dichloroethane	13.0
	2 methyl THF	13.0
	1-4 Dioxane	13.0
	MeCN	13.0

P6	Solvent	Concentration mg/mL
	1-3 Dioxane	24.2
	Glycol sulfite	24.2
	Diethyl sulfoxide	31.3
	Dimethyl sulfite	13.6

P7	Solvent	Concentration mg/mL
	1-3 Dioxane	8.1
	Glycol sulfite	8.1
	Diethyl sulfoxide	10.4
	Dimethyl sulfite	4.5

P8	Solvent	Concentration mg/mL
	Dibutyl formamide	7.5
	4 formyl morpholine	15.0
	1 formyl pyrrolidine	30.0
	2 Nitroethanol	20.0

P9	Solvent	Concentration mg/mL
	Dibutyl formamide	2.5
	4 formyl morpholine	5
	1 formyl pyrrolidine	10
	2 Nitroethanol	6.7

P10	Solvent	Concentration mg/mL
	1 methyl imidazole	31.3
	n-methyl formanilide	15.6

	Benzonitrile	10.4
	Adiponitrile	10.4

P11	Solvent	Concentration mg/mL
	1 methyl imidazole	15.6
	n-methyl formanilide	7.8
	Benzonitrile	5.2
	Adiponitrile	5.2

Results:

All crystallisation from ENaCt plates resulted in oxidative dimerization of the ligand with no observed crystallisation of the desired complex.

The complex was successfully crystallised as a large purple block from a 41.7 mg/mL DMF solution. Currently this is the only reported structure (REFCODE OJIGOG)

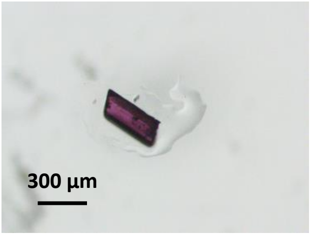

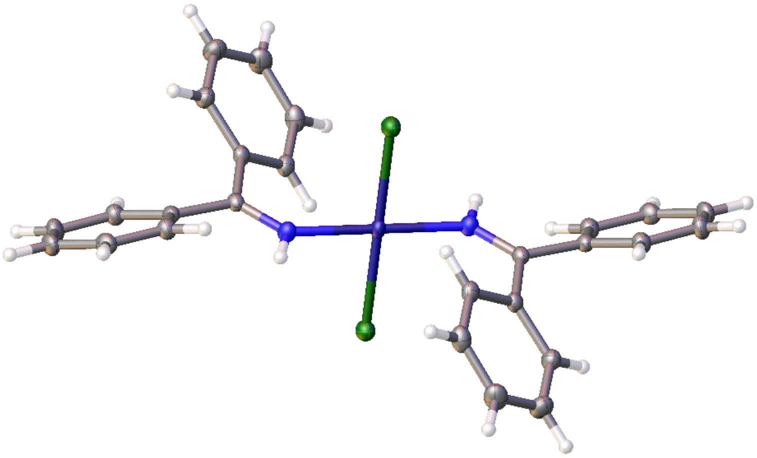

4. Executive Summary - XXIX

The crystallisation of liquid XXIX was attempted at subambient temperatures and also at high pressure in a diamond anvil cell. In each case, the same $Z^{prime} = 3$ crystal form that was used to simulate the low-quality PXRD pattern, was obtained. No further crystallisation screening was conducted.

Target XXIX (methyl anthranilate) – Crystal structure report

Ross, Marta J.; Kabova, Elena A. and Shankland, K

University of Reading, Reading UK

Materials and Methods

Single-crystal diffraction Methyl anthranilate (99%, product code A15681, lot 10224781) was purchased from Alfa Aesar. A small volume of liquid was poured into a Petri dish and placed in a cold room, where crystallisation occurred after lightly scratching the sample with a needle. A crystal of approximate dimensions 0.65 × 0.29 × 0.23 mm was isolated and mounted on a Rigaku Synergy single-crystal diffractometer equipped with a microfocus copper X-ray source, a Hypix 6000HE single-photon counting detector and an Oxford Cryosystems Cryostream cooling device, set to 274K. Data were collected over a period of 15 mins and the crystal structure solved using *ShelXT* [1] and refined using *ShelXL* [2] running as part of the *Olex2* system [3].

Powder diffraction Crystals of methyl anthranilate were ground for 1 minute at 30 Hz in a Retsch MM400 ball mill whose grinding vessel and balls had been previously cooled in liquid nitrogen. The resultant powder was loaded into a 1.0 mm borosilicate glass capillary and mounted on a Bruker D8 Advance Diffractometer operating in transmission capillary geometry, with a LynxEye detector, monochromatic incident radiation of wavelength 1.54056 Å, and an Oxford Cryosystems Cryostream Compact. Data were collected at 274K over the range 3–75° 2θ, with an 8 h data collection time (Figure X1, upper). The crystal structure was solved using *DASH* [4-6] and refined using *TOPAS* [7].

Simulated powder data for CSP XXIX Powder X-ray diffraction patterns for new physical forms are not always high quality and in order to simulate a scenario for the CSP challenge where this was the case, a low-quality PXRD pattern was carefully generated using *TOPAS*, based on the single-crystal structure. The simulated data were made accessible in the form of a PXRD plot (Figure X1, lower) and as an "xye" file if requested, together with relevant metadata i.e. capillary geometry, 274K, wavelength 1.54056 Å, step size 0.017°.

Results and Discussion

Methyl anthranilate crystallises in space group $P2_1/c$ with Z'=3. The structure as solved from experimental PXRD data is in excellent agreement (RMSD = 0.026 Å for a 15 molecule Crystal Packing Similarity in *Mercury* [8]) with the single-crystal structure, CSD REFCODE FASMEV.

1. G. Sheldrick, *Acta Crystallogr. Section A*, 2015, **71(1)**, 3-8.
2. G. Sheldrick, *Acta Crystallogr. Section C*, 2015, **71(1)**, 3-8.
3. O.V. Dolomanov, L. J. Bourhis, R. J. Gildea, J. A. K Howard and H. Puschmann 2009. *J. Appl. Crystallogr.*, 2009, **42(2)**, 339-341.
4. W. I. F. David, K. Shankland, J. van de Streek, E. Pidcock, W. D. S. Motherwell and J. C. Cole, *J. Appl. Crystallogr.*, 2006, **39**, 910-915.
5. T. A. N. Griffin, K. Shankland, J. V. van de Streek and J. Cole, *J. Appl. Crystallogr.*, 2009, **42**, 360-361.
6. E. A. Kabova, J. C. Cole, O. Korb, M. Lopez-Ibanez, A. C. Williams and K. Shankland, *J. Appl. Crystallogr.*, 2017, **50**, 1411-1420.
7. A. A. Coelho, *J. Appl. Crystallogr*. 2018, **51**, 210–218.
8. C. F. Macrae, I. Sovago, S. J. Cottrell, P. T. A. Galek, P. McCabe, E. Pidcock, M. Platings, G. P. Shields, J. S. Stevens, M. Towler and P. A. Wood, *J. Appl. Crystallogr.*, 2020, **53**, 226-235.

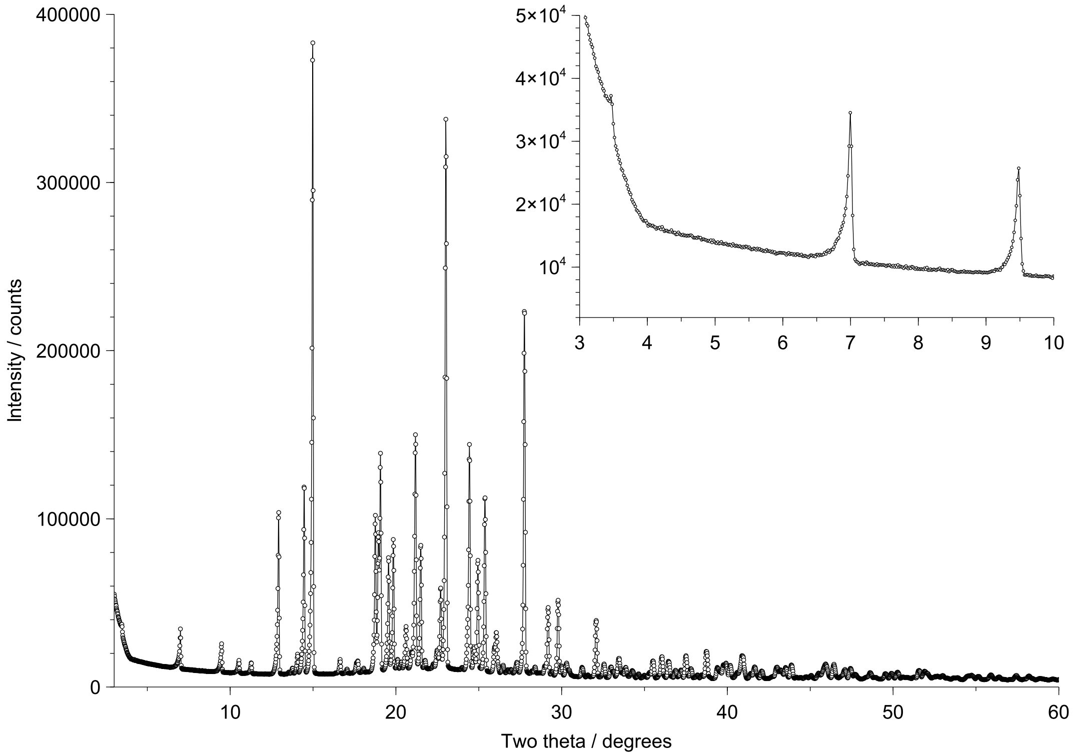

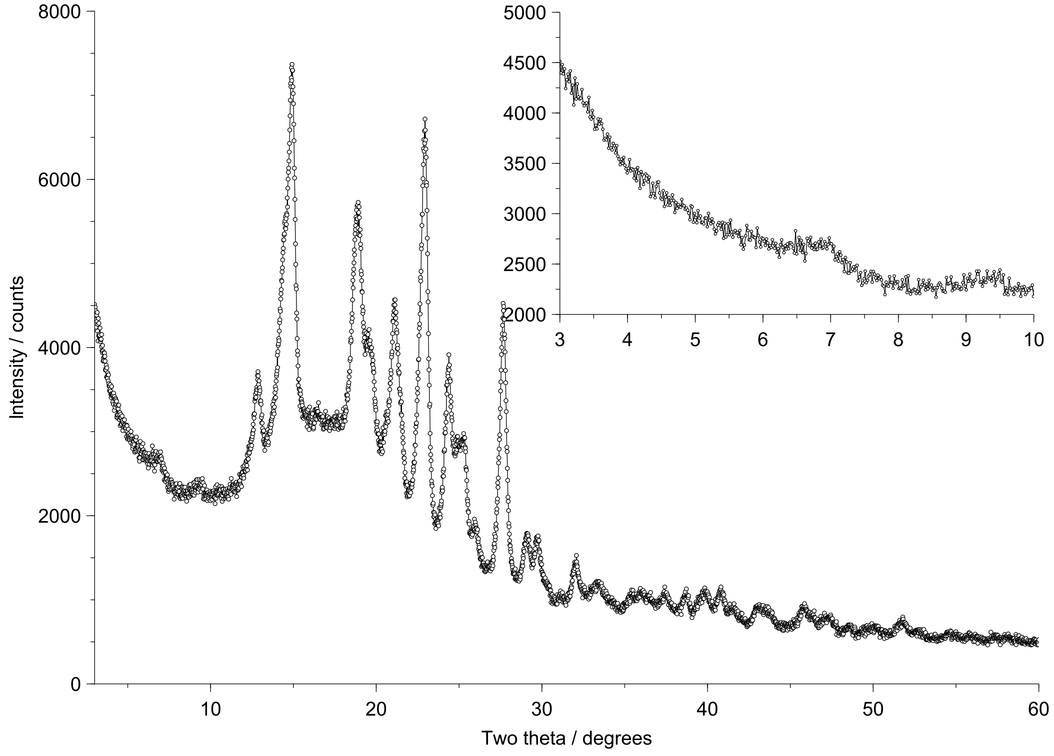

Figure X1. Experimental PXRD data for methyl anthranilate (upper plot) and simulated low quality PXRD data for the same structure (lower plot)

XXIX (methyl anthranilate) – Polymorph screen report

Michael Probert, Jake Weatherston

School of Natural and Environmental Sciences, Newcastle University

Methyl anthranilate low temperature crystallisation:

Methyl anthranilate was loaded into a Lindemann capillary tube, sealed and mounted to a magnetic goniometer base. The sample was then mounted to a diffractometer fitted with an open flow cooling system and crystallisation initiated.
The temperature was reduced in 1 K increments until a polycrystalline state was observed. Temperature cycling was then used to selectively melt and grow crystals until a single crystal large enough for analysis was observed in the tube. A single crystal of methyl anthranilate was obtained at 293 K.

Full single crystal X-ray diffraction data were then collected using a Bruker D8 Venture with Cu–Kα X-radiation at 293 K.

Methyl anthranilate high-pressure crystallisation:

Methyl anthranilate was loaded into a modified Merrill-Basset diamond anvil cell, using stainless steel gasket of 0.25 mm pre-indented to a thickness of 0.13 mm and the sample chamber drilled using an electric discharge machine with a 0.35 mm diameter.

The sample was loaded alongside a ruby sphere for pressure calibration using fluorescence spectroscopy.
An over-pressure was applied to the sample followed by a gradual reduction of pressure until multi-crystalline material was observed.
The pressure was then oscillated around the initial crystallisation pressure to selectively melt and grow crystals until an individual single crystal large enough for analysis was observed in the cell.
The multi-crystalline material was observed at a pressure of 2.66 kbar.
Diffraction data were collected on the sample by using a four-circle Huber diffractometer with Ag–Kα X-radiation.

Unit cell determination confirmed that the high-pressure form was the same as the low temperature form, due to quality of the accessible data the low-temperature model was used in the final refinements.

5. Executive Summary - XXX

Two cocrystal compositions of cannabinol (CBN) and 2,3,5,6-tetramethylpyrazine (TMP), 2:1 and 1:1, were screened for polymorphs by conventional solution crystallisation and slurrying. The original two cocrystal forms, along the pure component CBN and TMP forms, and an unstable, putative cocrystal form (Group E) were generated, frequently as mixtures, from *ca* 210 experiments encompassing 35 different solvent systems.

Target XXX – Crystal structure report

Joanna Bis, Stephen Carino, Ricky Couch, Lukasz Wojtas

Catalent Pharma Solutions, Morrisville, NC, USA

Single crystal X-ray diffraction analysis of two polymorphic forms reveals the presence of molecular dimers (SU016) and trimers (SD059) where 2,3,5,6-Tetramethylpyrazine molecules interact through NH…O hydrogen bond with one (SU016) or two (SD059) molecules of API (Figs. 1a and 1b, Tables 1 and 2). Those basic structural motifs further interact via weak, π…π, CH…π and CH…O, interactions to form structural ribbons (Figs. 2a & 3a) and complete structure (Figs. 2b and 3b). The full interaction pattern is relatively complex as it involves many weak interactions, however there are several features worth mentioning.

SU016: Apart from H-Bond the tetramethylpyrazine molecule is involved in π…π interactions with adjacent tetramethylpyrazine molecule as well as in CH_2/CH_3…π interactions (as donor and acceptor) with API molecules (Fig.2a). Adjacent API molecules "wrap" around tetramethylpyrazine molecules thanks to flexibility of aliphatic chain. Resulting structural ribbons are weakly interacting. (Fig.2b)

In case of SD059, the π…π / CH_3…π stacking is observed between API and pyrazine molecules leading, together with NH…O H-bonds, to formation of structural ribbon (Fig.3a. and (Fig.3b.)) Ribbons interact by CH…O hydrogen bonds. Presence of additional molecule of API eliminated stacking tendency between tetramethylpyrazine molecules observed in structure SU016.

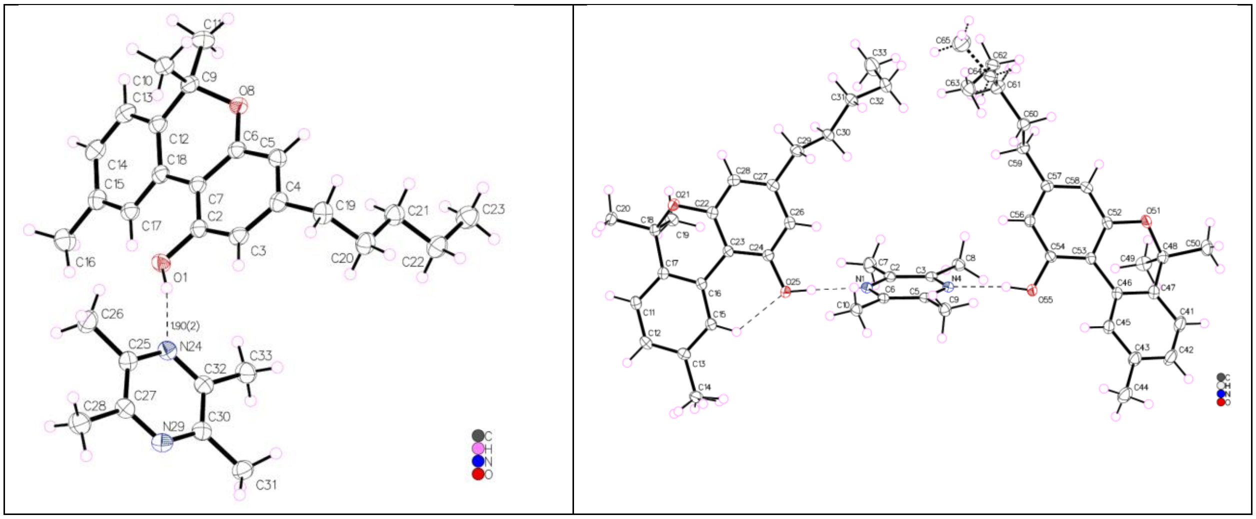

Fig.1a. Asymmetric unit and hydrogen bonding scheme within dimer of SU016	Fig.1b. Asymmetric unit and hydrogen bonding scheme within trimer of SD059

Table 1 Hydrogen Bonds for SU016.

D	H	A	d(D-H)/Å	d(H-A)/Å	d(D-A)/Å	D-H-A/°
O1	H1	N24	0.88(2)	1.90(2)	2.7630(13)	166.9(18)

Table 2 Hydrogen Bonds for SD059.

D	H	A	d(D-H)/Å	d(H-A)/Å	d(D-A)/Å	D-H-A/°
O25	H25	N1	0.897(18)	1.885(18)	2.7796(11)	174.8(16)
O55	H55	N4	0.881(17)	1.952(17)	2.8269(11)	171.9(16)

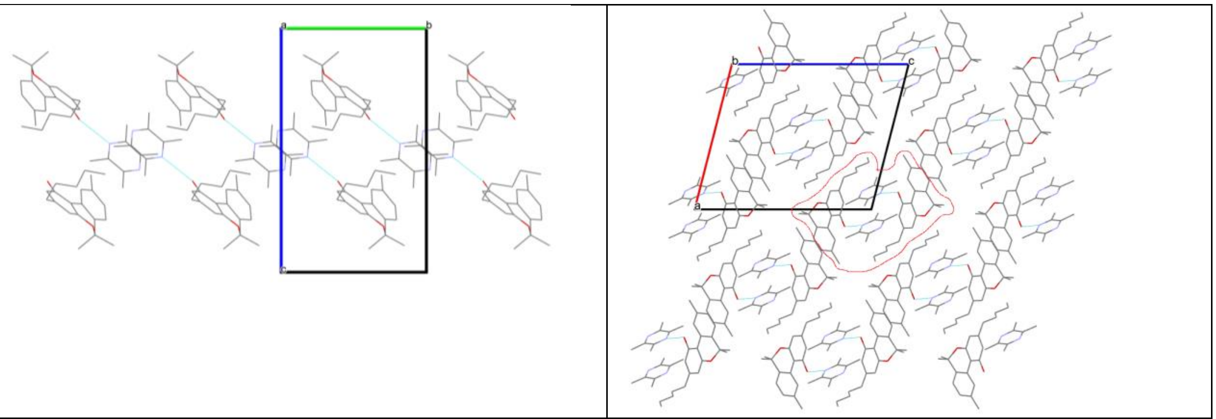

(a) (b)

Fig.2. Packing features of SU016. (a) Structural ribbon and (b) packing of ribbons in the structure. Ribbon is outlined in red.

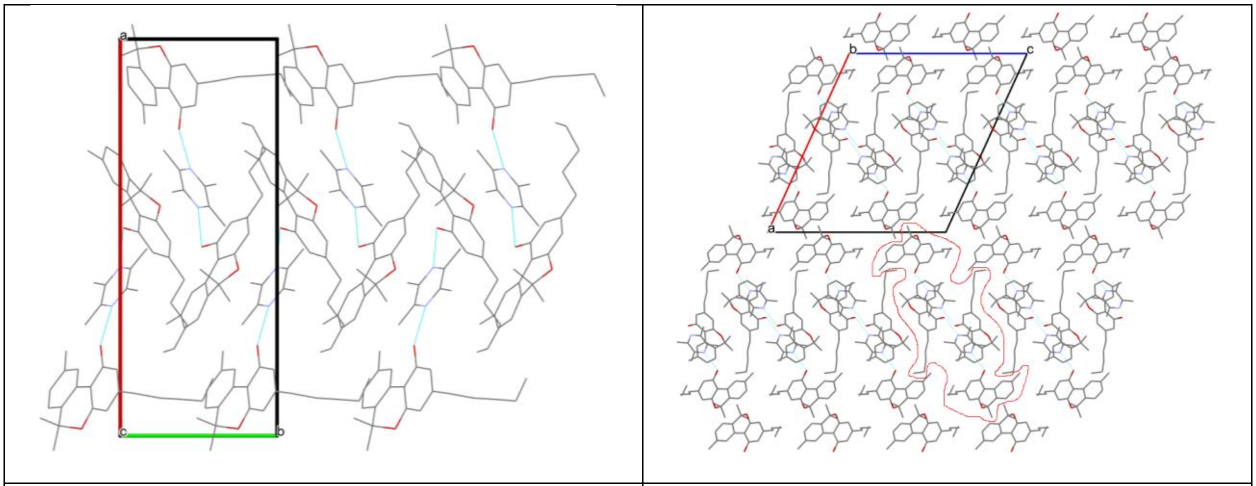

(a) (b)

Fig 3. Packing features of SD059. (a) Structural ribbon and (b) packing of ribbons in the structure. Ribbon is outlined in red.

Target XXX - Crystal-Form Screens of CBN/TMP Hemi- and Mono-Cocrystals

Joanna Bis, Stephen Carino, Ricky Couch

Catalent Pharma Solutions, Morrisville, NC, USA

Cannabinol (CBN, Figure 1) is a mildly psychoactive agent and a member of cannabinoid family, found in Cannabis plants. The related cannabidiol (CBD), has been reported in both domestic and international patent applications, to form cocrystals with various CCFs, including 2,3,5,6-Tetramethylpyrazine (TMP, Figure 1), with improved efficacy and bioavailability.[1] Subsequently, cocrystallization of CBN with TMP was attempted and resulted in a 1:0.5 (hemi-cocrystal) and 1:1 (mono-cocrystal) stoichiometric ratios.[2]

The single crystal X-ray structures of the CBN/TMP hemi- and mono-cocrystal structures were solved and utilized as targets for crystal structure prediction (CPS) exercise organized by CCDC, known as a “blind test”. To support the findings of the blind test and identify the most stable structures, polymorph screens were conducted on both cocrystals. Herein the single crystal structures of the two cocrystals as well as polymorph screen results are presented.

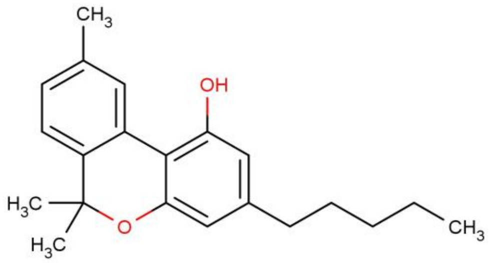

Molecular Formula = $C_{21}H_{26}O_2$
Molecular Weight = 310.43

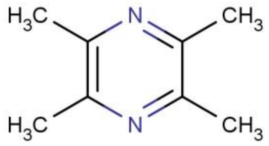

Formula: $C_8H_{12}N_2$
MW: 136.19 g/mol

Figure 1 - Molecular Structure, Weight and Formula of CBN (Left) and TMP (Right)

CBN (lot C15465) contains a weakly acidic phenol moiety with an estimated pKa of ~10. CBN is a white crystalline non-solvated powder with a sharp endotherm at 76 °C (ΔH=80 J/g). Solubility of CBN is high (>100 mg/mL) in majority organic solvents.

TMP (lot 183938) is a white crystalline non-solvated powder with a sharp endotherm at 85 °C (ΔH=156 J/g) and significant weight loss starting at approximately 50°C.

Isooctane was selected for preparation of the CBN and TMP cocrystals. The solubility of CBN and TMP in isooctane at room temperature are approximately 11-21 mg/mL and 52-104 mg/mL, respectively.

---

[1] International Patent Application Publication WO 2019/030158 A1, Feb. 14, 2019.

[2] WO 2021/138610

Preparation of CBN/TMP Hemi-Cocrystal

CBN (lot C15465, 2.91 g, 9.38 mmol) was combined with TMP (lot 183938; 639 mg, 0.5 eq) and isooctane (20.0 mL) and stirred at RT for 20 h. The product was isolated by vacuum filtration (using Whatman #1 filter paper, 2 layers), washed 4 times with isooctane (0.5 mL) and air dried for 6 h. The yield was 90% (3.20 g, 8.46 mmol) of hemi-cocrystal.

Preparation of CBN/TMP Mono-Cocrystal

CBN (batch C15465, 2.95 g, 9.49 mmol) was combined with TMP (lot 183938; 1.62 g, 1.25 eq) and isooctane (18 mL) and stirred for 20 h yielding a moderately thick suspension. The product was isolated by vacuum filtration (using Whatman #1 filter paper, 2 layers), washed three times with filtrate, and dried in a vacuum oven at 30 °C for 3 days. PXRD analysis of the vacuum dried product indicated the hemi-cocrystal and CBN. To drive the hemi-cocrystal to the mono-cocrystal, additional TMP (167 mg) and isooctane (10 mL) were and the suspension was stirred at RT overnight. The solids were isolated by vacuum filtration (Whatman #1 filter paper, 2 layers), washed twice with isooctane (0.5 mL) and air dried for 6 h. The yield was 88% (3.75 g, 8.39 mmol) of mono-cocrystal.

CBN/TMP Hemi- Cocrystal

The prepared CBN/TMP hemi-cocrystal is a non-solvated white crystalline powder with sharp endotherm at 73 °C (ΔH=86 J/g).

CBN/TMP Mono-Cocrystal

The prepared CBN/TMP mono-cocrystal is a white non-solvated crystalline powder with sharp endotherm at 76 °C (ΔH=92 J/g).

Solubility of both cocrystals was visually assessed in 18 diverse solvents at 20 °C and is shown in Table 1 – Estimated Solubility of CBN/TMP Hemi- and Mono- Cocrystals

#	Solvent (v/v)	Solubility Hemi at 20°C (mg/mL)	Solubility Mono at 20°C (mg/mL)
1	Methanol (MeOH)	>448	>400
2	2-Propanol (IPA)	>436	>388
3	Isopropyl ether (IPE)	>436	106-424
4	Dimethyl sulfoxide (DMSO)	>432	>408
5	Methyl t-butyl ether (MTBE)	>420	>404
6	2-Propanol:water (9:1)	>420	>408
7	Tetrahydrofuran (THF)	>404	>436
8	Toluene	>396	>436
9	Ethyl Acetate (EtOAc)	>384	>424
10	1-Octanol	>384	>368
11	Dichloromethane (DCM)	>364	>368
12	1-Hexanol	109-436	>436
13	Acetonitrile (MeCN)	108-432	91-364

14	Cyclohexane	104-416	97-388
15	Heptane	22-55	20-49
16	Petroleum ether	20-50	22-54
17	Isooctane	11-21	10-20
18	Water	<6	<5

n/a - not applicable

.

Table 1 – Estimated Solubility of CBN/TMP Hemi- and Mono- Cocrystals

#	Solvent (v/v)	Solubility Hemi at 20°C (mg/mL)	Solubility Mono at 20°C (mg/mL)
1	Methanol (MeOH)	>448	>400
2	2-Propanol (IPA)	>436	>388
3	Isopropyl ether (IPE)	>436	106-424
4	Dimethyl sulfoxide (DMSO)	>432	>408
5	Methyl t-butyl ether (MTBE)	>420	>404
6	2-Propanol:water (9:1)	>420	>408
7	Tetrahydrofuran (THF)	>404	>436
8	Toluene	>396	>436
9	Ethyl Acetate (EtOAc)	>384	>424
10	1-Octanol	>384	>368
11	Dichloromethane (DCM)	>364	>368
12	1-Hexanol	109-436	>436
13	Acetonitrile (MeCN)	108-432	91-364
14	Cyclohexane	104-416	97-388
15	Heptane	22-55	20-49
16	Petroleum ether	20-50	22-54
17	Isooctane	11-21	10-20
18	Water	<6	<5

n/a - not applicable

CRYSTAL-FORM SCREENS

The crystal-form screens of the hemi- and mono- cocrystals were each comprised of ~105 crystallization experiments and involved 35 solvent systems, three crystallization modes (slurry ripening, cooling, evaporation), and a temperature range of 5-25 °C.

The crystal-form screens of the CBN/TMP hemi- cocrystal produced the input forms of the hemi-cocrystal and mono-cocrystals, pure CBN and TMP, and a new unstable solid (designated Group E) with the hemi-cocrystal being the most dominant product.

The crystal-form screens of the CBN/TMP mono-cocrystal produced similar results as compared to the hemi- cocrystal screen, except that the most dominant product of the screen was a mixture of the hemi- and mono- cocrystals.

Group E was observed in both screens; however, it and was more frequently observed in the mono-screen. Group E was obtained only from evaporation experiments, usually as a mixture with the hemi- or mono-cocrystal.

Group E was obtained phase-pure only from evaporation of a solution of the mono-in tetrahydrofuran, as a very thick wet solid that could not be filtered. Several attempts to reproduce Group E by some other mode than evaporation (i.e. seeding a saturated solution, and solvent/antisolvent addition) were unsuccessful. Phase pure Group E proved to be unstable and converted to a mixture of the hemi- cocrystal and CBN at ambient or to a mixture of the mono- and hemi- cocrystals in a closed vial.

Overall, the observations suggest that Group E is a cocrystal of CBN and TMP; however due to the challenges on its production and inability to filter, and instability the stoichiometry could not be determined.

Group E appears to be a non-solvated cocrystal with a small endotherm at 67 °C (ΔH=9 J/g), followed by a large endotherm at 77 °C (ΔH=62 J/g).

PXRD patterns of the hemi- cocrystal, the mono- cocrystal, and the unstable putative cocrystal Group E are shown in Figure 2. PXRD patterns of the hemi-cocrystal, CBN, and TMP are shown in Figure 3. PXRD patterns of the mono- cocrystal, CBN, and TMP are shown in Figure 4.

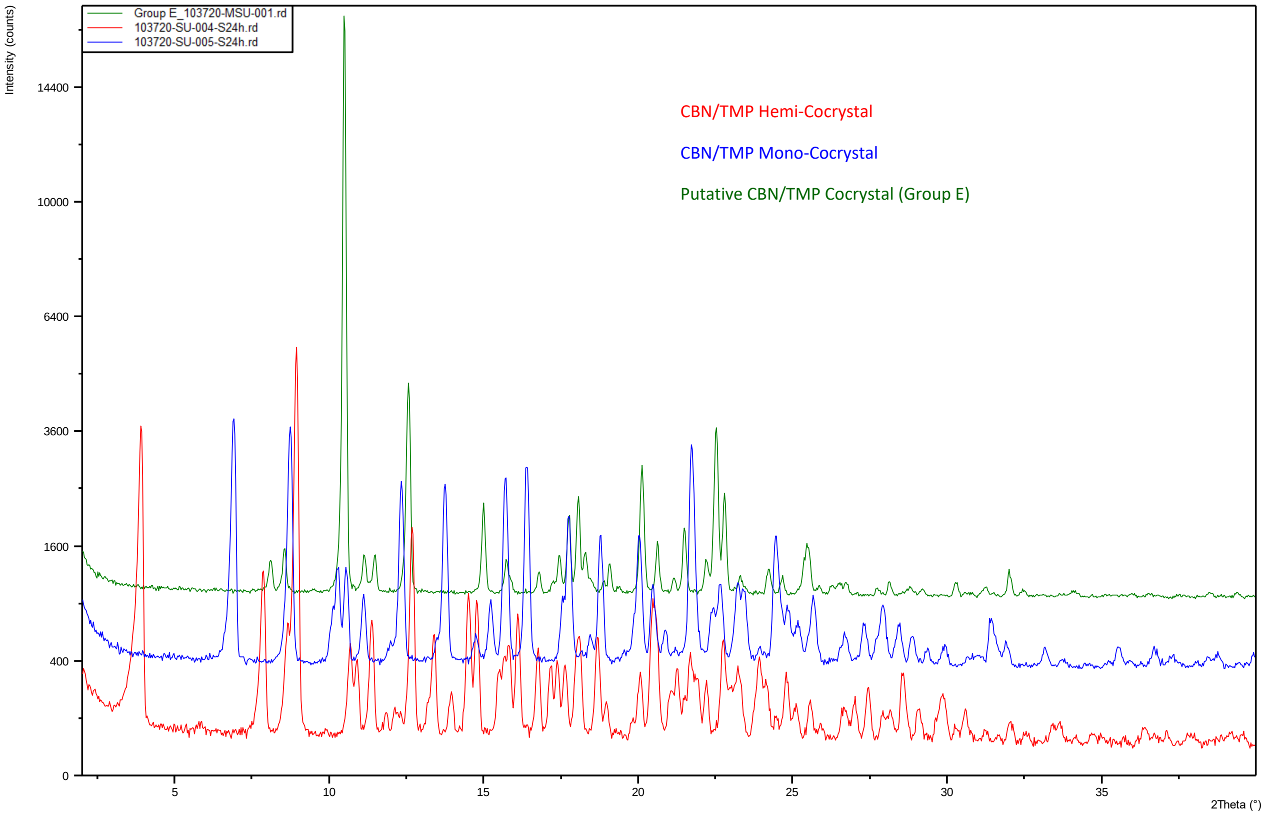

Figure 2 - PXRD Patterns of Hemi- and Mono- Cocrystals and Putative Cocrystal Group E

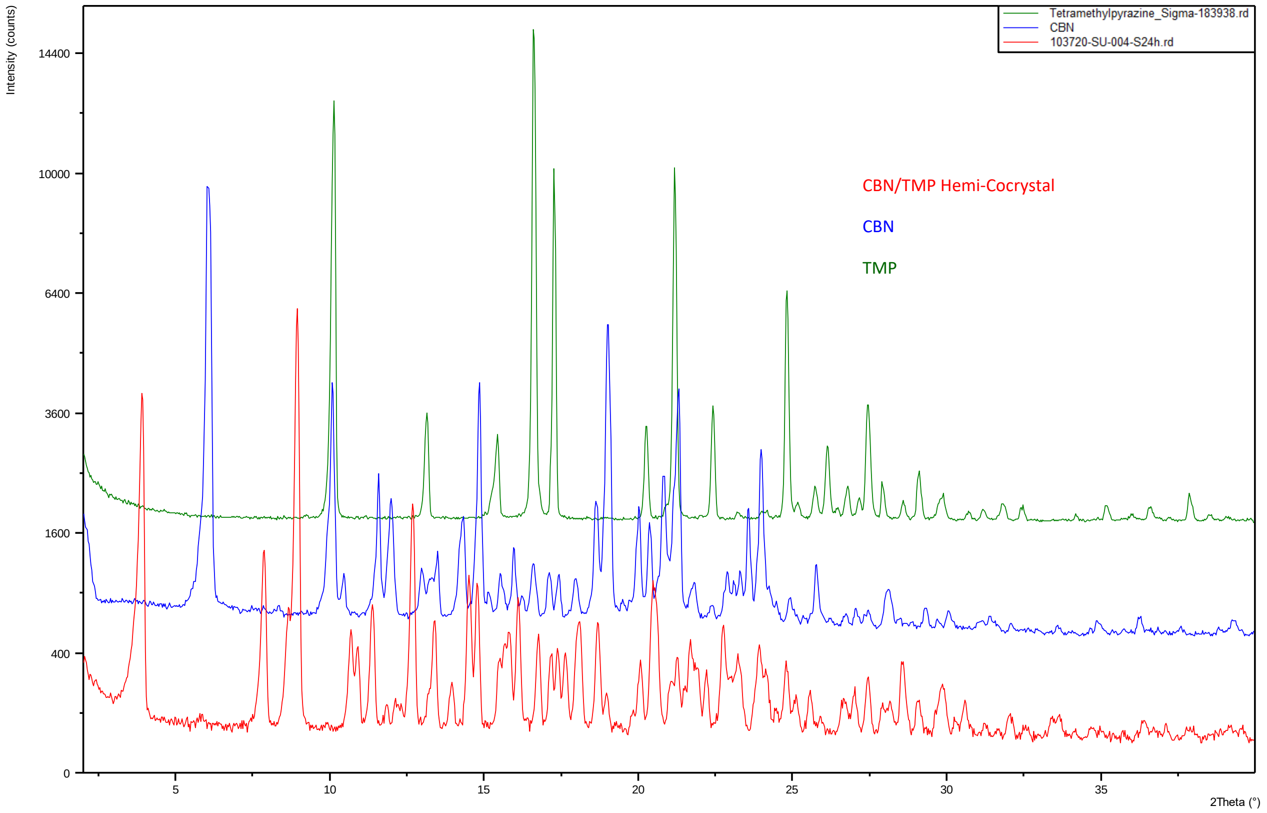

Figure 3 - PXRD Patterns of Hemi-Cocrystal, CBN, and TMP

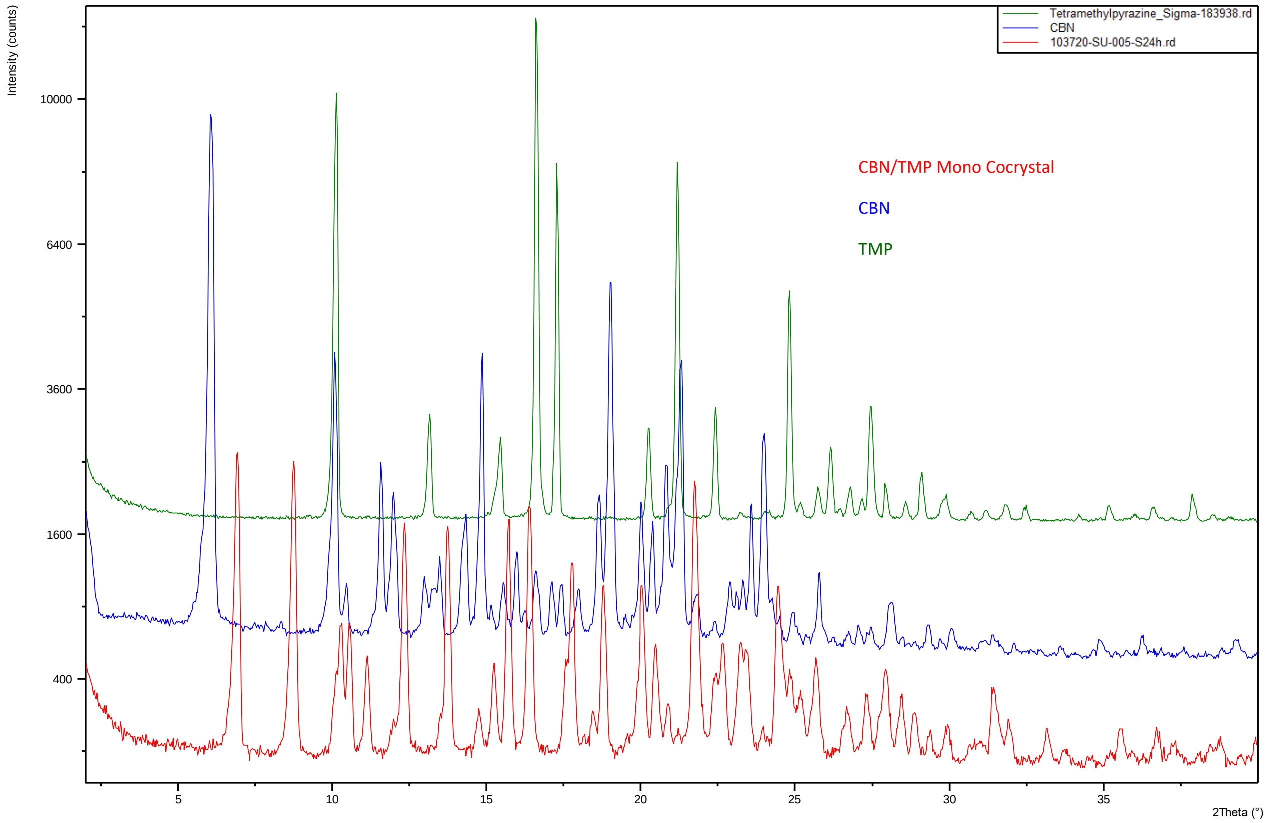

Figure 4 - PXRD Patterns of Mono-Cocrystal, CBN, and TMP

Materials and Methods

Cannabinol lot C15465 was provided by Purisys and used in this screening study without further purification or processing.

2,3,5,6-Tetramethylpyrazine (Sigma-Aldrich lots 183938 and 14003DE) were purchased from Sigma-Aldrich used in this screening study without further purification or processing.

Analysis of Crystal-Form Screening Products

FT-Raman spectroscopy and powder X-ray diffraction (PXRD) were chosen as the primary methods for analysis and grouping of samples. Representative samples from the groupings were analyzed by FT-Raman or PXRD to verify their uniqueness. Where possible/practical, a representative sample of the unique group was further characterized by PLM, DSC, and/or TGA-IR.

Solubility Assessment

Solubility of hemi- and mono- cocrystal was visually assessed in 18 diverse solvents at 20 °C and 40 °C to support the selection of solvents and corresponding dosing strategies for the subsequent screening experiments. The solubility was visually estimated at 20 °C by dosing small aliquots of solvent into a fixed amount of solid (~10 mg) until the dissolution point or a maximum volume (1.8 mL) was reached. Samples that contained undissolved solids at RT were heated to 40 °C for 1 h and the dissolution was assessed visually.

Instrumentation

Polarized-Light Microscopy (PLM). The photomicrographs were collected using Olympus BX60 polarized-light microscope equipped with Olympus DP70 camera or Olympus BX51 polarized-light microscope equipped with Olympus DP71 camera.

Powder X-Ray Diffraction (PXRD) Bruker. PXRD diffractograms were acquired on a Bruker D8 Advance system using Cu Ka (40 kV/40 mA) radiation and a step size of 0.03° 2θ and LynxEye detector. Configuration on the incident beam side: Goebel mirror, mirror exit slit (0.2 mm), 2.5 deg Soller slits, beam knife. Configuration on the diffracted beam side: anti-scatter slit (8 mm) and 2.5 deg. Soller slits. Samples were mounted flat on zero-background Si wafers.

Differential Scanning Calorimetry (DSC). DSC was conducted with a TA Instruments Q100 or Q2000 differential scanning calorimeter equipped with an autosampler and a refrigerated cooling system under 40 mL/min N_2 purge. DSC thermograms of samples were obtained at 10 °C/min or 15 °C/min in crimped Al pans, unless noted otherwise. The temperatures of exothermic and endothermic transitions recorded via DSC analysis are reported as onset values.

Thermogravimetric Analysis (TGA). TGA thermograms were obtained with a TA Instruments Q50 thermogravimetric analyzer under 40 mL/min N_2 purge for balance and 60 mL/min for sample in Al pans. TGA thermograms of samples were obtained at 10 °C/min or 15 °C/min, unless noted otherwise.

Thermogravimetric Analysis with IR Off-Gas Detection (TGA-IR). TGA-IR was conducted with a TA Instruments Q5000 thermogravimetric analyzer interfaced to a Nicolet 6700 FT-IR spectrometer (Thermo Electron) equipped with an external TGA-IR module with a gas flow cell and DTGS detector. TGA was conducted with 25 mL/min N_2 flow for sample purge, 10 mL/min N_2 flow for balance purge, and

heating rate of 15 ^{o}C/min in Al pans. IR spectra were collected at 4 cm^{-1} resolution and 32 scans at each time point.

6. Executive Summary - XXXI

Three crystal forms of XXXI produced from a form screen of 400+ conventional crystallisation conditions have been identified by XRPD and characterised by SCXRD. The forms, labelled F1, F2 and F3, are renamed A, B, and C, respectively, in the main paper. F3 (Form C) appears to be solvated (low temperature endotherm by DSC), although solvent was not identified in the porous crystal structure of this form. Details of the solid form screen beyond the qualitative stability assessment of F1 and F2 (Forms A and B) were not provided.

XXXI – Crystal form and polymorph screen report

John Hone, Adam Keates, Ian Jones

Syngenta Ltd, Berkshire, UK

A polymorph screen was conducted by performing a high-throughput evaporative, drown-out, cooling and temperature cycling crystallisations in 28 different solvent and solvent mixtures. After a total of over 400 crystallisations, this screen produced 3 polymorphs termed F1, F2 and F3 with the resulting single crystal structures being solved at 120, 200 and 120K respectively. Three crystals were picked for analysis: crystals grown from methanol by evaporation (F1), crystals grown by temperature cycling an aqueous suspension (F2) and crystals grown from a formulation containing benzylacetate with temperature cycling (F3). F3 is a solvate based on the voids, a PLATON SQUEEZE function was applied because no ordered solvent could be identified. The DSC behaviour of F3 is also typical for a solvate. Competitive slurry tests of F1 and F2 shows enantiotropic polymorphism with an approximate transition temperature around 55 °C. F2 being stable at lower temperatures and F1 stable at higher temperatures.

<u>Supplementary information for paper</u>

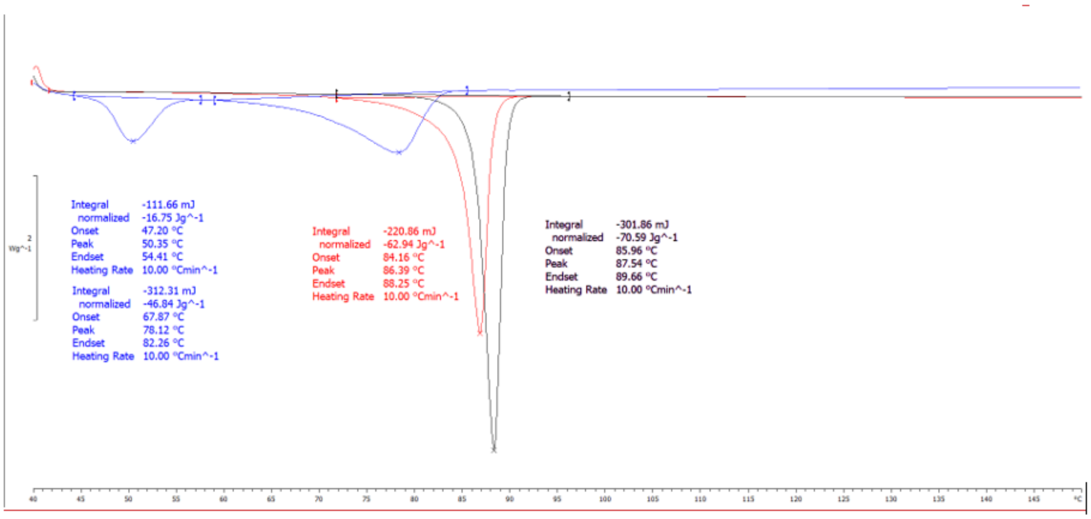

Figure X: DSC patterns of novel forms of F1 (black), F2 (red), F3 (blue)

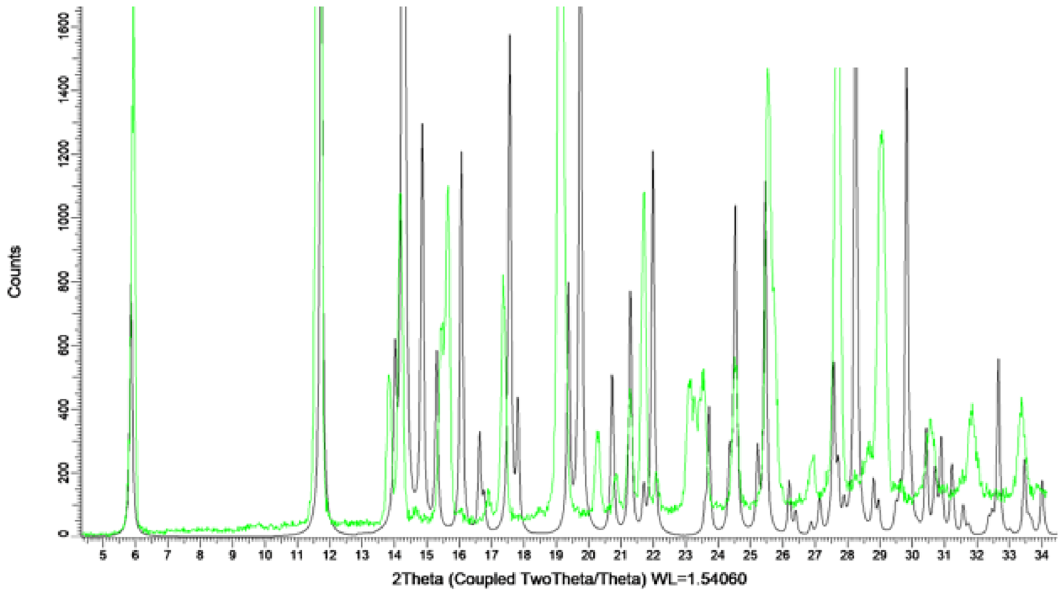
Counts
2Theta (Coupled TwoTheta/Theta) WL=1.54060
(a)

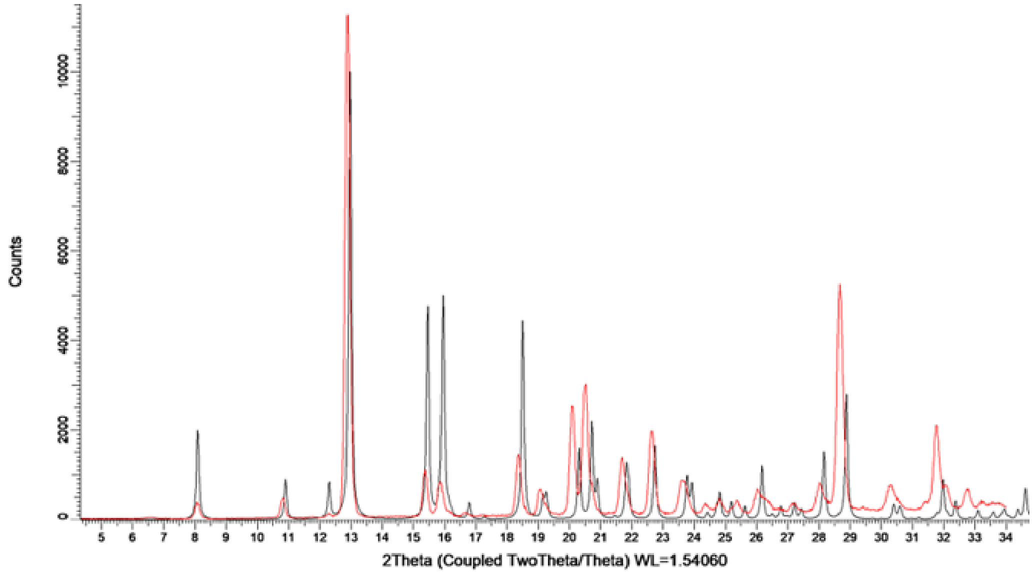
Counts
2Theta (Coupled TwoTheta/Theta) WL=1.54060
(b)

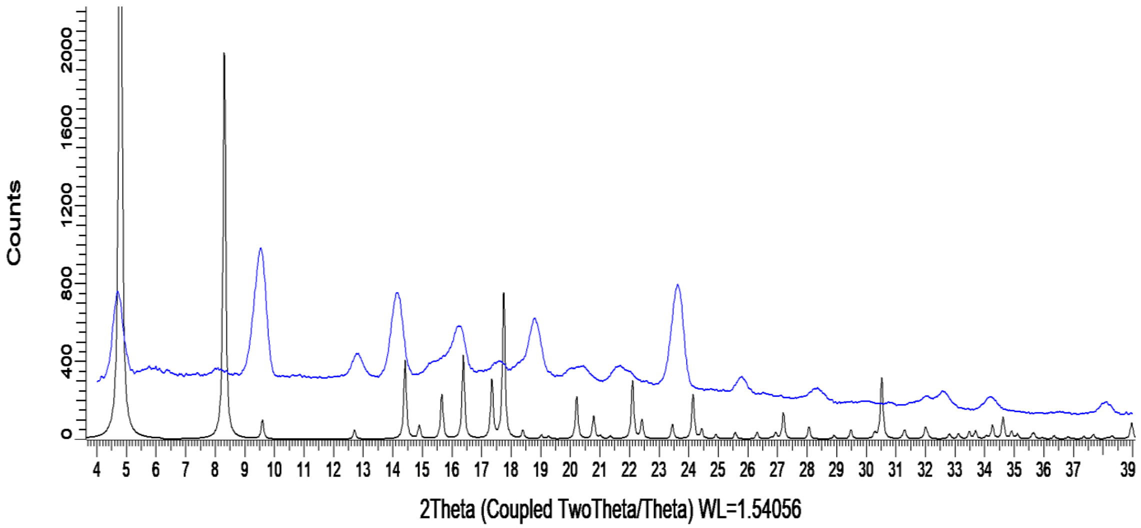

(c)

Figure X: Comparison to experimental (colour traces) and predicted patterns (black) from single crystals. (a) Form 1, (b) Form 2 & (c) Form 3

7. Executive Summary - XXXII

A solid form screen for XXXII, referred to as 'G02949338' in the following report, produced evidence of at least 25 crystal forms. Conventional crystallisation methods were used in the survey of 80 experimental conditions. The large number of different forms, many of which were observed just once, suggests that the search for polymorphs is possibly, if not likely, far from complete. XXXII shows a strong propensity to form solvates with diverse solvents. Most of the materials isolated, including those reported to be 'anhydrates', retain non-negligible amounts of solvent. Without full characterisation of the volatile content of the different forms or investigation into the possibility of low stoichiometry solvates or non-stoichiometric solvates, it is impossible to conclude from the reported screen that XXXII has been produced in 8 different non-solvated forms.

Two anhydrous polymorphs of XXXII were confirmed by SCXRD, with Form A (referred to as Type A in the screening report) being used to provide the input material for the solid form screen. Form B (referred to as Type M in the screening report) was not produced in the solution phase part of the solid form screen. Instead, this polymorph was produced from an iBuOAc slurry at 100°C, conditions which have been shown to favor non-solvated forms. Slurry bridging studies were subsequently used to qualitatively assess the relative stability of Forms A and B. Form B was shown to be thermodynamically more stable at and above ambient temperature than Form A. The DSC data reported for the 'putative' neat polymorphs suggests that Form B may not have the highest measured enthalpy, within the error of the measurements. As such, Form B should not be assumed the most stable lattice energy minimum.

During the blind test, an additional crystal structure of Form B was determined from

PXRD at room temperature (RT), a $Z' = 1$ structure in the $P2_1/c$ space group, which suggested a structural difference to the 90 K form (a $Z' = 1$ structure in the $P\bar{1}$ space group). A redetermination of the structure was later proposed by group 20. Solid-state nuclear magnetic resonance (NMR) shielding calculations carried out confirmed that the redetermined crystal structure from CSP provided a better fit to experimental data than that previously derived from PXRD and is reported here.

Table of Contents

1 Summary 1
2 Characterization of Anhydrates and Hydrates 3
2.1 Anhydrates 4
2.1.1 Type A 4
2.1.2 Type H 5
2.1.3 Type K and R 6
2.1.4 Type L 9
2.1.5 Type M 11
2.1.6 Type N 12
2.1.7 Type P 13
2.2 Hydrates 15
2.2.1 Type C 15
2.2.2 Type G 17
2.2.3 Type U 20
2.2.4 Type X 22
3 Evaluation of Anhydrates 25
3.1 Thermodynamic Stability Relationship 25
3.1.1 Type A, K and H 25
3.1.2 Type H, N and M 27
3.1.3 Type R, P and M 29
3.2 Kinetic Solubility 31
3.2.1 Type A and H 31
3.2.2 Type A, M and N 34
4 Conclusion 38
5 Appendix 39
5.1 Starting Materials 39
5.2 Abbreviation for Solvents Used 40
5.3 Instruments and Methods 40
5.3.1 XRPD 40

5.3.2 TGA and DSC 41
5.3.3 ^{1}H NMR 41
5.3.4 DVS 41
5.3.5 HPLC 42
5.4 Characterization of Solvates 43
5.4.1 Type I 43
5.4.2 Type B 46
5.4.3 Type E 48
5.4.4 Type J 51
5.4.5 Type Q 53
5.4.6 Type S 56
5.5 Characterization of Transient and Unidentified Forms 58
5.5.1 Type D 58
5.5.2 Type G0 59
5.5.3 Type F 60
5.5.4 Type O 61
5.5.5 Type T 61
5.5.6 Type V 62
5.5.7 Type W 63
5.6 Polymorph Screening 64
5.6.1 Solid Vapor Diffusion 65
5.6.2 Anti-solvent Addition 66
5.6.3 Slow Cooling 66
5.6.4 Slurry at RT 67
5.6.5 Slurry at 50 ºC 68
5.6.6 Solution Vapor Diffusion 68
5.6.7 Slow Evaporation 69
5.7 Notebook Reference 70

List of Tables

Table 2-1 Characterization summary of anhydrates and hydrates 3
Table 2-2 Characterization summary of solvates 3
Table 3-1 Summary of specific procedures of competitive slurries 26
Table 3-2 Summary of competitive slurries of Type A, H and K 26
Table 3-3 Summary of specific procedures of competitive slurries 28
Table 3-4 Summary of competitive slurries of anhydrates Type H, N and M..................... 28
Table 3-5 Summary of specific procedures of competitive slurries 30
Table 3-6 Summary of competitive slurries of anhydrates.. 30
Table 3-7 Summary of kinetic solubility of Type A and H at 37 ºC 32
Table 3-8 Summary of kinetic solubility of Type A, M and N at 37 ºC 35
Table 5-1 Sample information 39
Table 5-2 Solvent abbreviation list 40
Table 5-3 Parameters for XRPD test.. 40
Table 5-4 Parameters for TGA and DSC test... 41
Table 5-5 Parameters for DVS test 42
Table 5-6 Chromatographic conditions and parameters for solubility measurement......... 42
Table 5-7 Approximate solubility of Type A (805711-05-A) at RT 64
Table 5-8 Summary of polymorph screening experiments 65
Table 5-9 Summary of solid vapor diffusion experiments.. 65
Table 5-10 Summary of anti-solvent addition experiments... 66
Table 5-11 Summary of slow cooling experiments 67
Table 5-12 Summary of slurry conversion experiments at RT... 67
Table 5-13 Summary of slurry conversion experiments at 50 ºC....................................... 68
Table 5-14 Summary of solution vapor diffusion experiments... 69
Table 5-15 Summary of evaporation experiments.. 70

List of Figures

Figure 1-1 Inter-conversion diagram of G02949338 anhydrates 2
Figure 1-2 Inter-conversion diagram of G02949338 hydrates, solvates, transient or unidentified forms 2
Figure 2-1 XRPD pattern of Type A (805711-05-A) 4
Figure 2-2 TGA/DSC curves of Type A (805711-05-A) 5
Figure 2-3 XRPD patterns of Type H batches 6
Figure 2-4 TGA/DSC curves of Type H (805711-17-A1) 6
Figure 2-5 XRPD patterns of Type R (805711-41-A2) and Type K (805711-22-C) 7
Figure 2-6 TGA/DSC curves of Type K (805711-22-C) 8
Figure 2-7 TGA/DSC curves of Type R (805711-41-A2) 8
Figure 2-8 *In situ* VT-XRPD overlay of Type K (805711-22-C) at varying temperatures 9
Figure 2-9 XRPD pattern of Type L (805711-25-B) 10
Figure 2-10 TGA/DSC curves of Type L (805711-25-B) 10
Figure 2-11 XRPD overlay of Type L (805711-32-B) pre and post slurry in IBAc at RT 11
Figure 2-12 XRPD pattern of Type M (805711-37-A) 12
Figure 2-13 TGA/DSC curves of Type M (805711-37-A) 12
Figure 2-14 XRPD patterns of Type N batches 13
Figure 2-15 TGA/DSC curves of Type N (803718-05-A4) 13
Figure 2-16 XRPD pattern of Type P (805711-41-B) 14
Figure 2-17 TGA/DSC curves of Type P (805711-41-B) 14
Figure 2-18 XRPD patterns of Type C batches 15
Figure 2-19 TGA/DSC curves of Type C (805711-10-A1) 16
Figure 2-20 DVS plot of Type C (805711-14-A1) 16
Figure 2-21 XRPD overlay of Type C (805711-14-A1) before and after DVS test 17
Figure 2-22 XRPD overlay of heating experiments on Type C 17
Figure 2-23 XRPD patterns of different batches of Type G 18
Figure 2-24 TGA/DSC curves of Type G (805711-17-A2) 19
Figure 2-25 XRPD overlay of Type G (805711-17-A2) before and after heating 19
Figure 2-26 *In situ* VT-XRPD overlay of Type G (805711-22-A1) at variable temperatures

.... 20
Figure 2-27 XRPD pattern of Type U (805711-09-B) 21
Figure 2-28 TGA/DSC curves of Type U (805711-09-B) 21
Figure 2-29 ^{1}H NMR spectrum of Type U (805711-09-B) 22
Figure 2-30 XRPD overlay of Type U (805711-09-B) before and after heating 22
Figure 2-31 XRPD patterns of Type X batches 23
Figure 2-32 TGA/DSC curves of Type X (805711-08-B) 24
Figure 2-33 ^{1}H NMR spectrum of Type X (805711-08-B) 24
Figure 2-34 XRPD overlay of Type X (805711-13-A11) before and after heating 25
Figure 3-1 XRPD overlay for slurry competition of anhydrates Type A, H and K at RT and 50 ºC 26
Figure 3-2 XRPD overlay for slurry competition of anhydrates Type A, H and K at 70 and 100 ºC 27
Figure 3-3 XRPD overlay for slurry competition of anhydrates Type M and H 29
Figure 3-4 XRPD overlay for slurry competition of anhydrates Type M and N 29
Figure 3-5 XRPD overlay for slurry competition of anhydrates Type M and R 31
Figure 3-6 XRPD overlay for slurry competition of anhydrates Type M and S 31
Figure 3-7 Solubility profile of Type A and H in water and pH 6.5 phosphate buffer 32
Figure 3-8 XRPD overlay of solubility samples in water starting from Type A 33
Figure 3-9 XRPD overlay of solubility samples in pH 6.5 phosphate buffer starting from Type A 33
Figure 3-10 XRPD overlay of solubility samples in water starting from Type H 34
Figure 3-11 XRPD overlay of solubility samples in pH 6.5 phosphate buffer starting from Type H 34
Figure 3-12 Solubility profile of Type A, M and N in water and pH 6.5 phosphate buffer .. 35
Figure 3-13 XRPD overlay of solubility samples in water starting from Type A 36
Figure 3-14 XRPD overlay of solubility samples in pH 6.5 phosphate buffer starting from Type A 36
Figure 3-15 XRPD overlay of solubility samples in water starting from Type M 37
Figure 3-16 XRPD overlay of solubility samples in pH 6.5 phosphate buffer starting from Type M 37
Figure 3-17 XRPD overlay of solubility samples in water starting from Type N 38

Figure 3-18 XRPD overlay of solubility samples in pH 6.5 phosphate buffer starting from Type N 38
Figure 5-1 XRPD patterns of starting materials 39
Figure 5-2 XRPD patterns of Type I batches 44
Figure 5-3 TGA/DSC curves of Type I (805711-18-A1) 44
Figure 5-4 XRPD overlay of Type I (805711-18-A1) before and after heating 45
Figure 5-5 ^{1}H NMR spectrum of Type I (805711-18-A1) 45
Figure 5-6 TGA/DSC curves of Type I (803718-22-B) 46
Figure 5-7 XRPD patterns of Type B (805711-10-A7) and (805711-15-A3) 47
Figure 5-8 TGA/DSC curves of Type B (805711-15-A3) 47
Figure 5-9 ^{1}H NMR spectrum of Type B (805711-15-A3) 48
Figure 5-10 XRPD overlay of Type B (805711-15-A3) before and after heating 48
Figure 5-11 XRPD patterns of Type E (805711-10-A3) and (805711-15-A2) 49
Figure 5-12 TGA/DSC curves of Type E (805711-15-A2) 50
Figure 5-13 ^{1}H NMR spectrum of Type E (805711-15-A2) 50
Figure 5-14 XRPD overlay of Type E (805711-15-A2) before and after heating 51
Figure 5-15 XRPD patterns of Type J (805711-07-A12) and (805711-15-A4) 52
Figure 5-16 TGA/DSC curves of Type J (805711-15-A4) 52
Figure 5-17 ^{1}H NMR spectrum of Type J (805711-15-A4) 53
Figure 5-18 XRPD overlay of Type J (805711-15-A4) before and after heating 53
Figure 5-19 XRPD pattern of Type Q (803718-29-A3) 54
Figure 5-20 TGA/DSC curves of Type Q (803718-29-A3) 55
Figure 5-21 ^{1}H NMR spectrum of Type Q (803718-29-A3) 55
Figure 5-22 XRPD overlay of Type Q (803718-29-A3) pre and post heating 56
Figure 5-23 XRPD pattern of Type S (805711-12-A12) 57
Figure 5-24 TGA/DSC curves of Type S (805711-12-A12) 57
Figure 5-25 ^{1}H NMR spectrum of Type Q (803718-29-A3) 58
Figure 5-26 XRPD overlay of Type S (805711-12-A12) pre and post heating 58
Figure 5-27 XRPD overlay of Type D (805711-22-A1) pre and post air drying 59
Figure 5-28 XRPD pattern of Type G0 (805711-23-B) 60
Figure 5-29 XRPD overlay of Type F (803718-12-A3) pre and post air drying 60
Figure 5-30 XRPD overlay of Type O (803718-41-A1) pre and post air drying 61

Figure 5-31 XRPD overlay of Type T (805711-46-A5) pre and post air drying.................. 62
Figure 5-32 XRPD overlay of Type V (805711-12-A4) pre and post air drying.................. 63
Figure 5-33 XRPD overlay of Type V re-preparation trial (805711-46-A1)......................... 63
Figure 5-34 XRPD overlay of Type W (805711-08-A7) pre and post air drying................. 64

1 Summary

The purpose of this project was to perform a polymorph screening for compound G02949338 towards identifying all known crystal forms and evaluating stability relationships among these crystal forms.

Starting material of G02949338 (Batch B000814725, CP ID: 805711-05-A) as received was characterized by X-ray powder diffraction (XRPD), thermogravimetric analysis (TGA), and differential scanning calorimetry (DSC). Characterization results indicate starting material is an anhydrate, and this crystal form is assigned as Type A.

Using Type A as the starting material, a polymorph screening was conducted under 80 conditions, through methods of anti-solvent addition, evaporation, slow cooling, slurry conversion, and vapor diffusion. From screening and follow-up study, a total of 25 crystal forms were identified, including eight anhydrates, four hydrates, six solvates (one isomorphic form Type I), and seven transient or unidentified forms at ambient conditions.

Based on screening and characterization results, stability relationships of all the anhydrates were established via competitive slurries at varying temperatures from room temperature (RT, 25 ± 3 ºC) to 100 ºC. Inter-conversion diagram of all the anhydrates is illustrated in Figure 1-1. Combined with the difference of melting points, Type M, with the highest melting point, was confirmed to be the stable anhydrate above RT. In order to compare solubility difference of anhydrates Type M, N, H versus Type A in vitro, kinetic solubility was measured in water and pH 6.5 phosphate buffer at 37 ºC, sampled at the endpoint of 1, 2, 4 and 24 hours, with solids for XRPD characterization and filtrates for HPLC and pH tests. In both media, Type A converted into hydrate Type C within 1 hour, while Type M exhibited the slowest conversion rate and a smooth solubility change.

Based on screening and evaluation results, Type M is the thermodynamically stable anhydrate above RT, and recommended for further development.

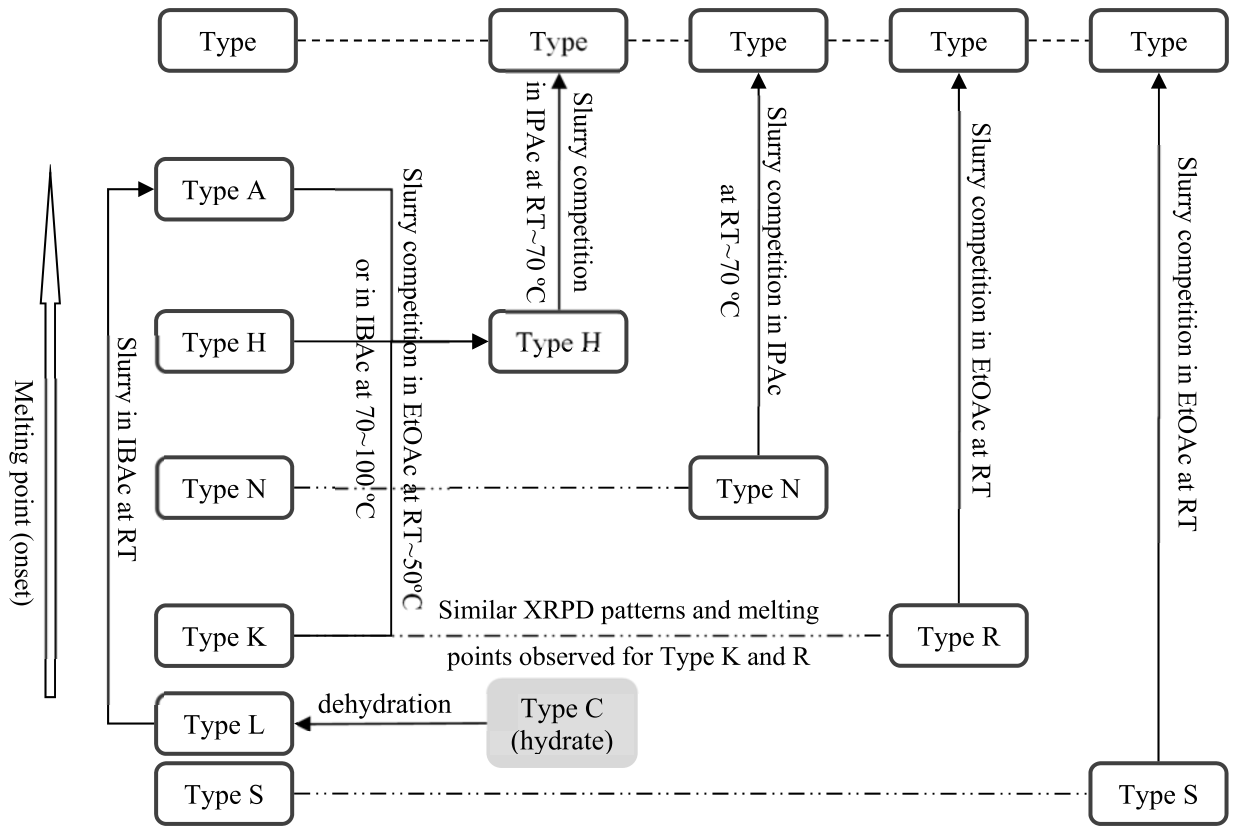

Figure 1-1 Inter-conversion diagram of G02949338 anhydrates

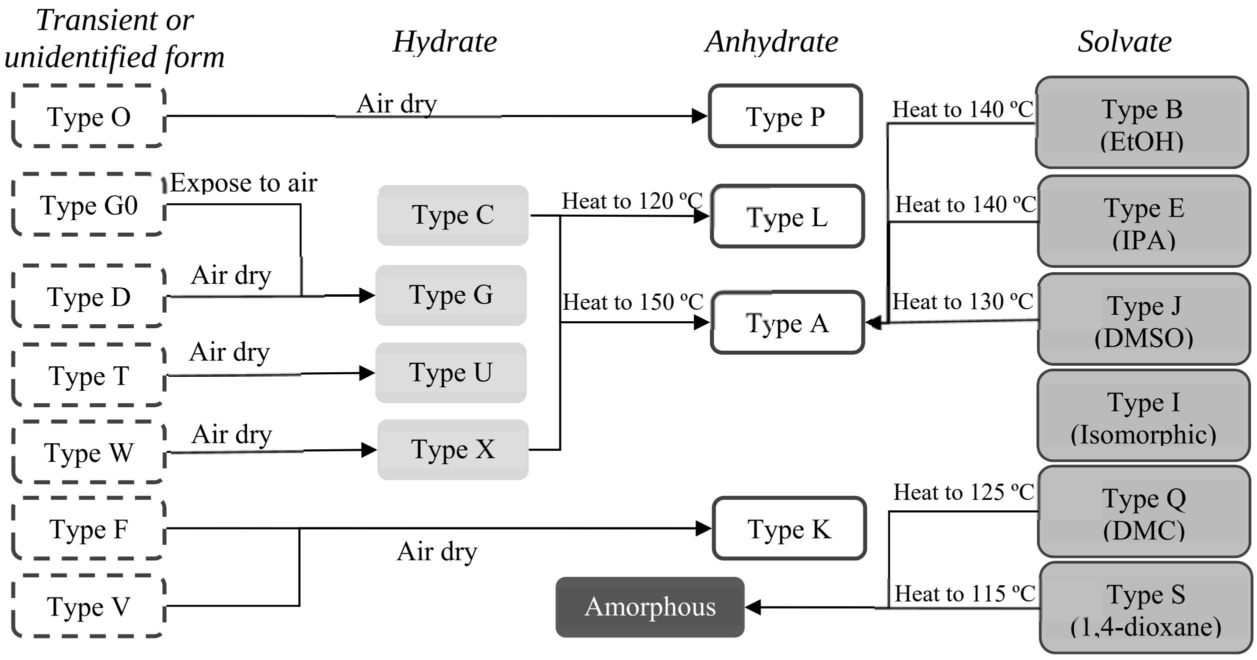

Figure 1-2 Inter-conversion diagram of G02949338 hydrates, solvates, transient or unidentified forms

2 Characterization of Anhydrates and Hydrates

From polymorph screening and follow-up investigation, a total of 25 crystal forms were obtained based on XRPD pattern comparison. These crystal forms were further identified and characterized, with characterization data summarized in Table 2-1 and Table 2-2. Study results of solvates and transient or unidentified forms can be referred to Section 5.4 and 5.5, respectively.

Table 2-1 Characterization summary of anhydrates and hydrates

Crystal Form	Batch ID	Wt Loss (%)	Endotherm (peak, ºC)	Speculated Form
Type A	805711-05-A	1.8	167.6	Anhydrate
Type H	805711-17-A1	1.7	159.1	
Type K	805711-22-C	4.1	148.1	
Type L	805711-25-B	1.8	131.8, 166.2	
Type M	805711-37-A	1.0	182.5	
Type N	803718-05-A4	1.3	160.5	
Type P	805711-41-B	1.5	120.0	
Type R	805711-41-A2	2.5	148.8, 183.0	
Type C	805711-10-A1	6.0	74.2, 99.1, 134.1, 167.4	Hydrate
Type G	805711-17-A2	3.8	77.2, 120.4	
Type U	805711-09-B	3.5	68.9, 110.4	
Type X	805711-08-B	3.0	66.3, 90.9, 132.1, 165.1	

Table 2-2 Characterization summary of solvates

Crystal Form	Batch ID	Wt Loss (%)	Endotherm (peak, ºC)	Solvent Content*	Speculated Form
Type B	805711-15-A3	7.9	112.9, 118.9, 168.2	6.3% EtOH	EtOH solvate
Type E	805711-15-A2	9.4	107.0, 168.5	6.8% IPA	IPA solvate
Type I	805711-18-A1	2.2	77.5, 120.2, 129.3	Limited acetone	Isomorphic form
	803718-22-B	6.0	68.6, 125.6	5.4% MEK	
Type J	805711-15-A4	12.4	111.1	12.2% DMSO	DMSO solvate
Type Q	803718-29-A3	7.0	124.1	6.1% DMC	DMC solvate
Type S	805711-12-A12	15.2	116.3	10.2% 1,4-dioxane	1,4-dioxane solvate

*: determined by proton nuclear magnetic resonance (^{1}H NMR) or gas chromatography (GC).

2.1 Anhydrates

2.1.1 Type A

The starting material (Batch No.: B000814725, CP ID: 805711-05-A) was characterized by XRPD, TGA and DSC. XRPD displayed in Figure 2-1 showed the starting material is crystalline, assigned as Type A. TGA and DSC data are displayed in Figure 2-2. A weight loss of 1.8 % up to 150 ºC in TGA and the only sharp melting endotherm at 166.3 ºC (onset temperature) in DSC suggested that Type A is an anhydrate.

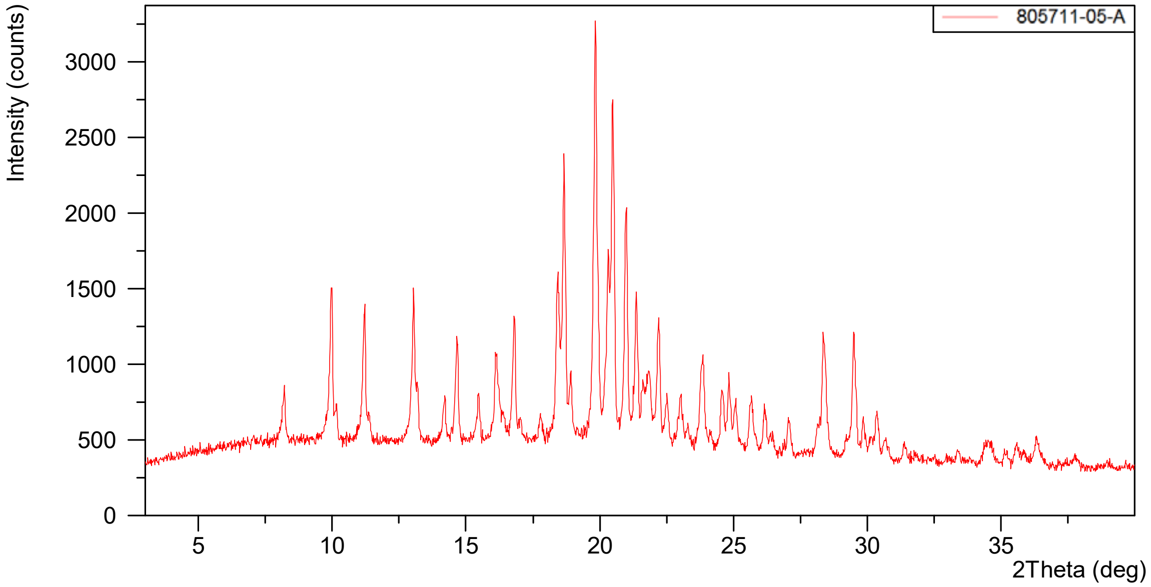

Figure 2-1 XRPD pattern of Type A (805711-05-A)

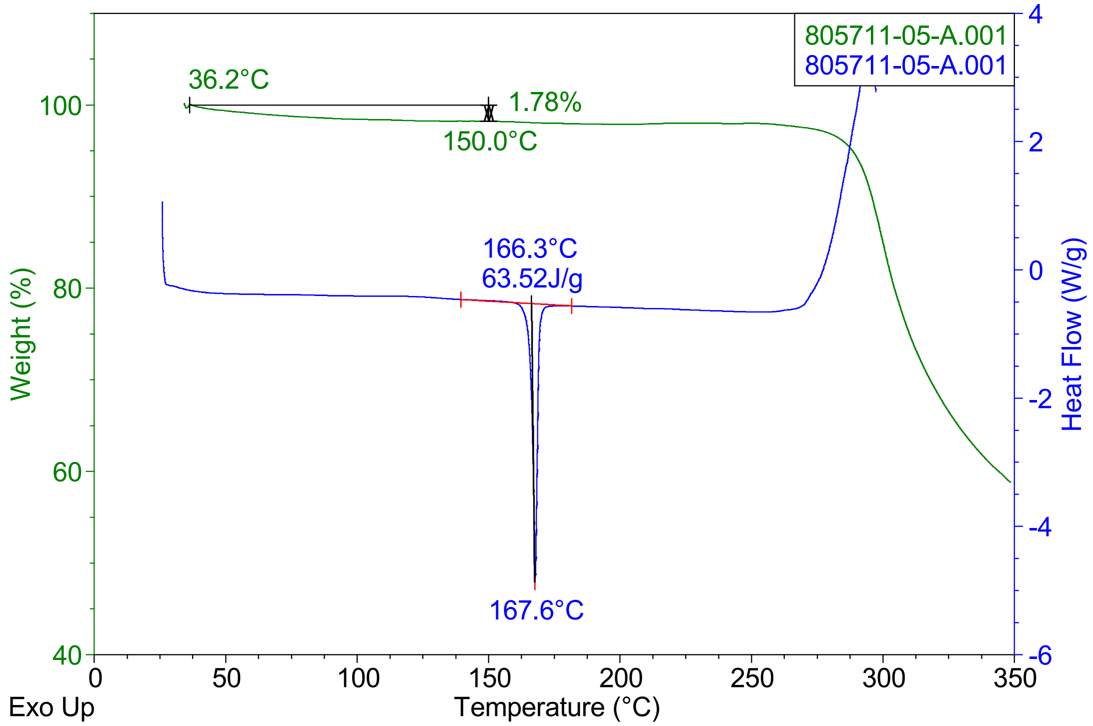

Figure 2-2 TGA/DSC curves of Type A (805711-05-A)

2.1.2 Type H

Type H can be prepared via solvent-mediated crystallization from multiple solvent systems. Type H sample (805711-17-A1) was obtained via slurry of Type A (805711-15-A) in EtOAc at 50 ºC, and its XRPD pattern is shown in Figure 2-3. As per TGA and DSC data shown in Figure 2-4, a weight loss of 1.7% up to 150 ºC was observed in TGA and DSC result showed a sharp melting endotherm at 154.6 ºC (onset temperature), revealing that Type H is an anhydrate.

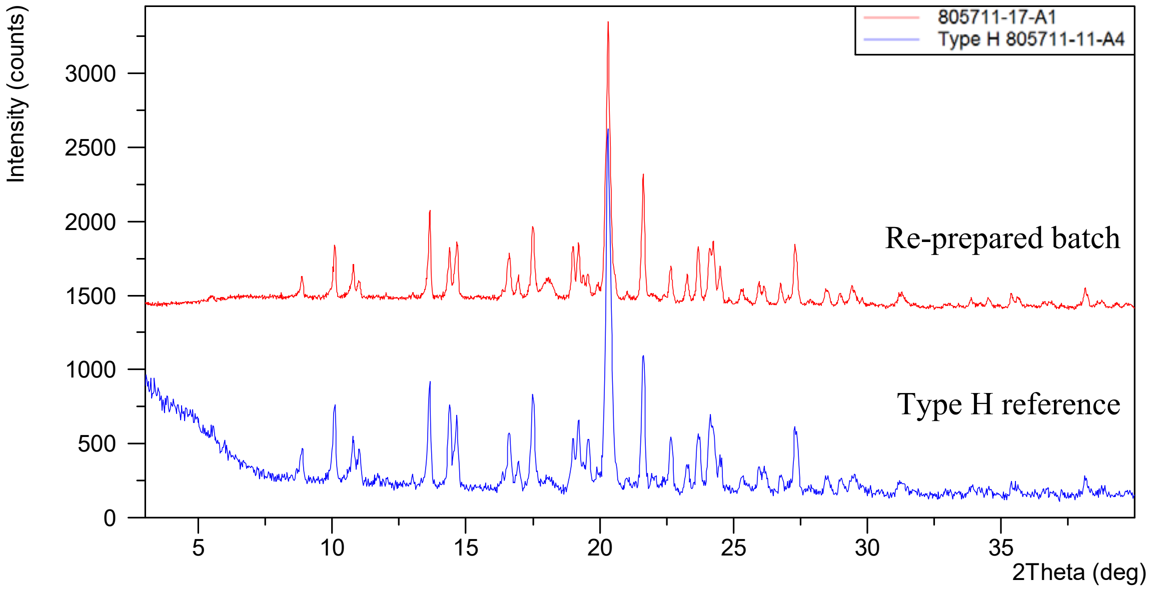

Figure 2-3 XRPD patterns of Type H batches

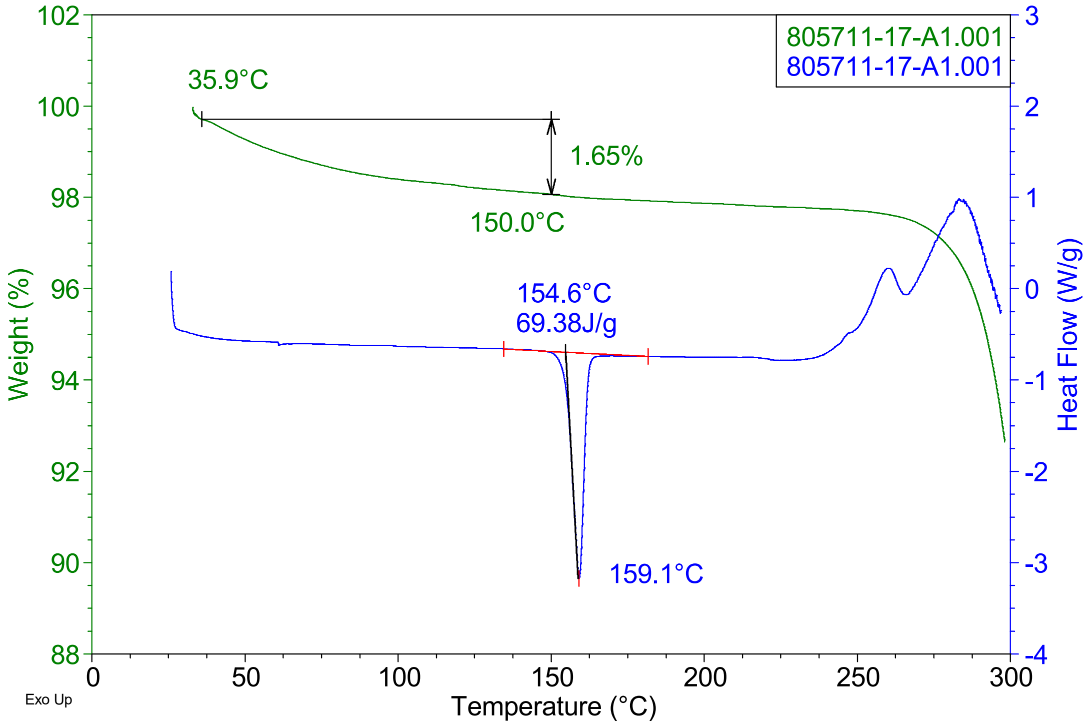

Figure 2-4 TGA/DSC curves of Type H (805711-17-A1)

2.1.3 Type K and R

Type K can be prepared from multiple solvent systems, while Type R is observed from DMSO/IBAc or DMSO/IPAc system. From the XRPD comparison in Figure 2-5, XRPD patterns of Type R and K

are similar with slight difference as marked.

Type K sample (805711-22-C) was obtained via slurry of Type A (805711-15-A) in DCM/n-heptane (v/v, 1:1) at RT. As shown by the TGA and DSC results (Figure 2-6), a weight loss of 4.1% up to 120 ºC and a sharp melting endotherm at 142.4 ºC (onset temperature) were observed for Type K (805711-22-C). To investigate the significant weight loss, *in situ* variable temperature-XRPD (VT-XRPD) was performed on Type K sample (805711-22-C). As displayed in Figure 2-8, no form change was observed, indicating that Type K is an anhydrate.

Type R sample (805711-41-A2) was suspended in DMSO/IBAc (v/v, 1:1) at -20 ºC, to improve its crystallinity. TGA and DSC data are displayed in Figure 2-7. A weight loss of 2.5% up to 140 ºC was observed in TGA and DSC showed a sharp melting endotherm at 145.0 ºC (onset temperature). The minor endotherm at 183.0 ºC (peak temperature) was attributed to melting of Type M.

Slight difference in XRPD patterns and melting points was observed for Type K and R, which may suggest the structure similarity and need further investigation.

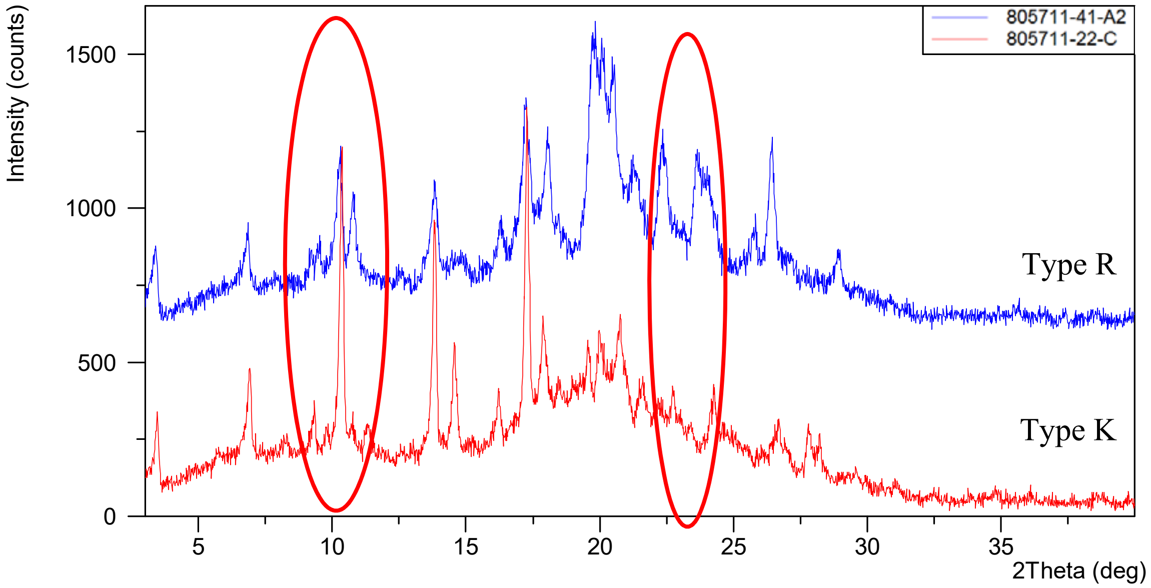

Figure 2-5 XRPD patterns of Type R (805711-41-A2) and Type K (805711-22-C)

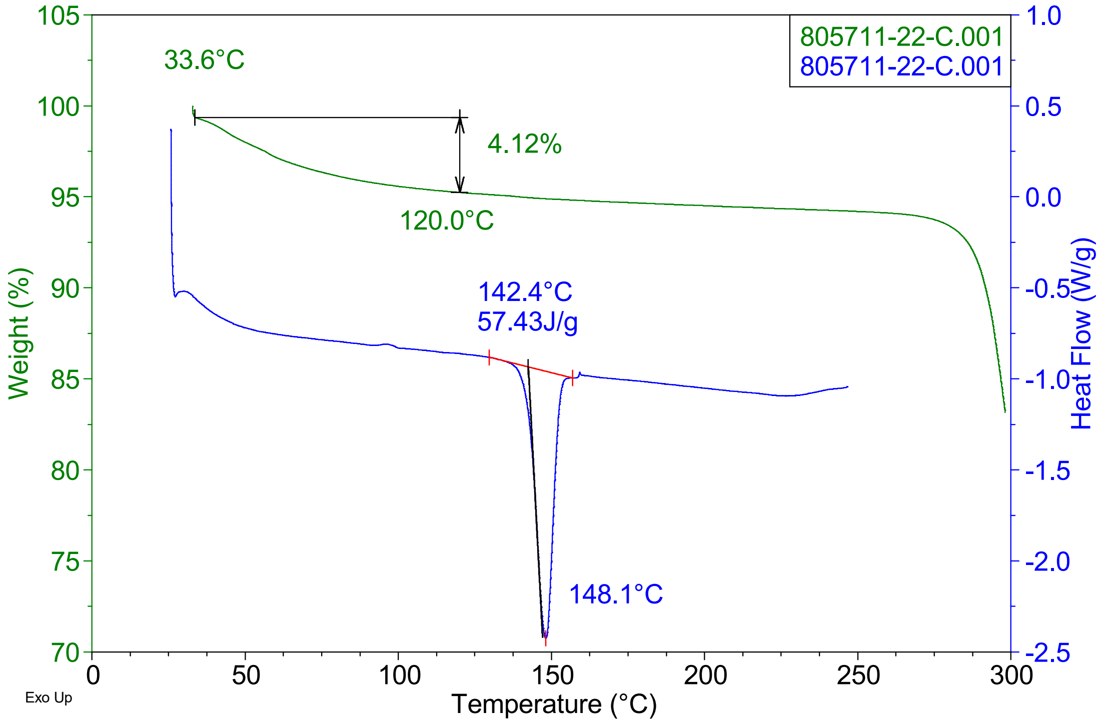

Figure 2-6 TGA/DSC curves of Type K (805711-22-C)

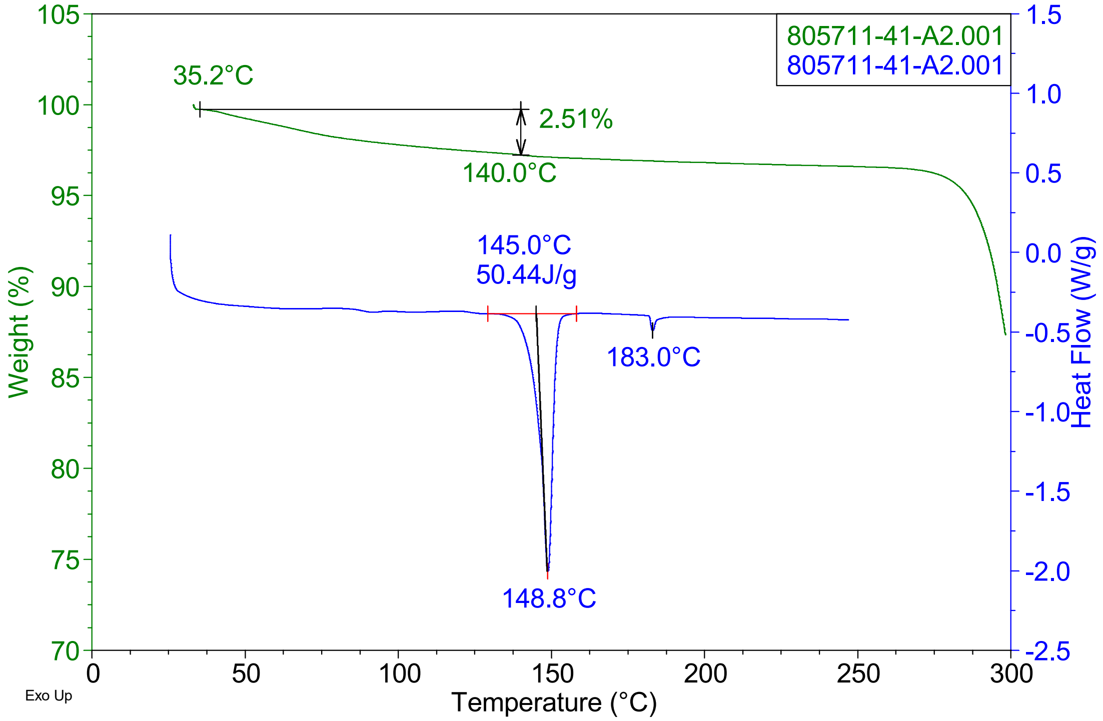

Figure 2-7 TGA/DSC curves of Type R (805711-41-A2)

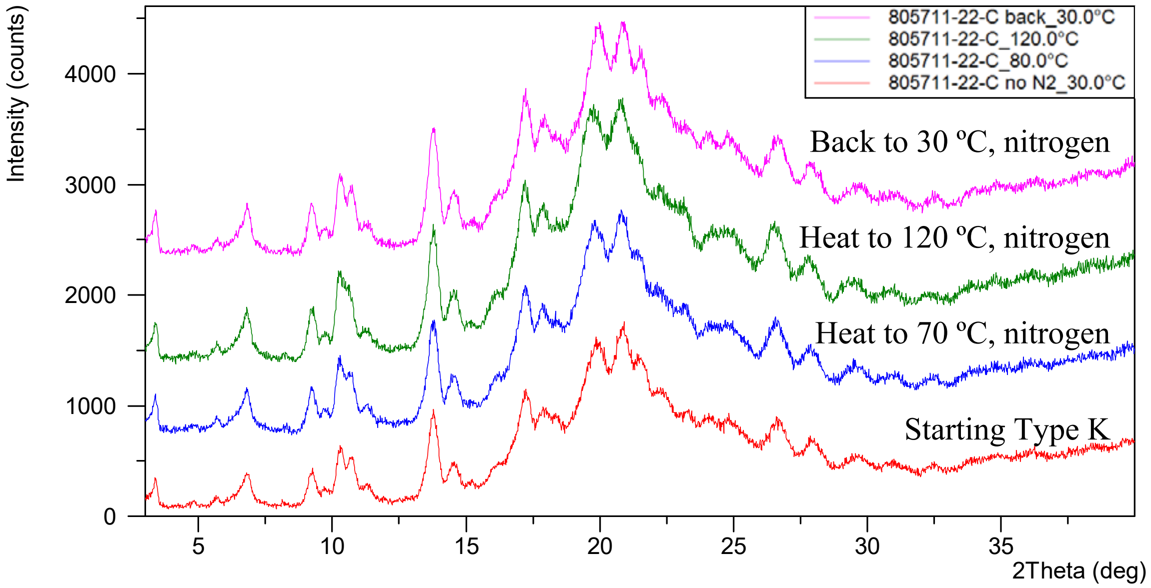

Figure 2-8 *In situ* VT-XRPD overlay of Type K (805711-22-C) at varying temperatures

2.1.4 Type L

Type L sample (805711-25-B) was obtained via heating hydrate Type C sample (805711-14-A1) to 120 ºC and cooling to RT under protection of nitrogen. Its XRPD pattern is shown in Figure 2-9. As per TGA and DSC data shown in Figure 2-10, a limited weight loss of 1.8% up to 120 ºC was observed in TGA and DSC result showed concomitant endotherm and exotherm before melting at 166.2 ºC (peak temperature), suggesting the anhydrate identity of Type L. The concomitant endotherm and exotherm was confirmed to be the recrystallization signal into Type A (see Section 2.2.1). As shown in Figure 2-11, Type L converted into Type A after slurry in IBAc at RT, so Type A is deemed to be thermodynamically more stable than Type L above RT.

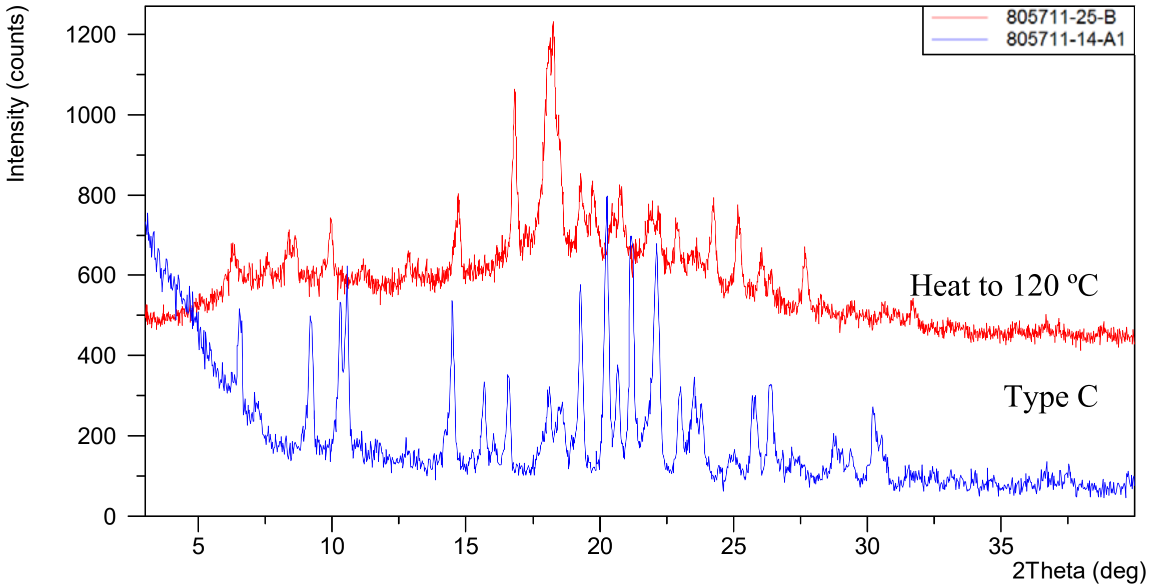

Figure 2-9 XRPD pattern of Type L (805711-25-B)

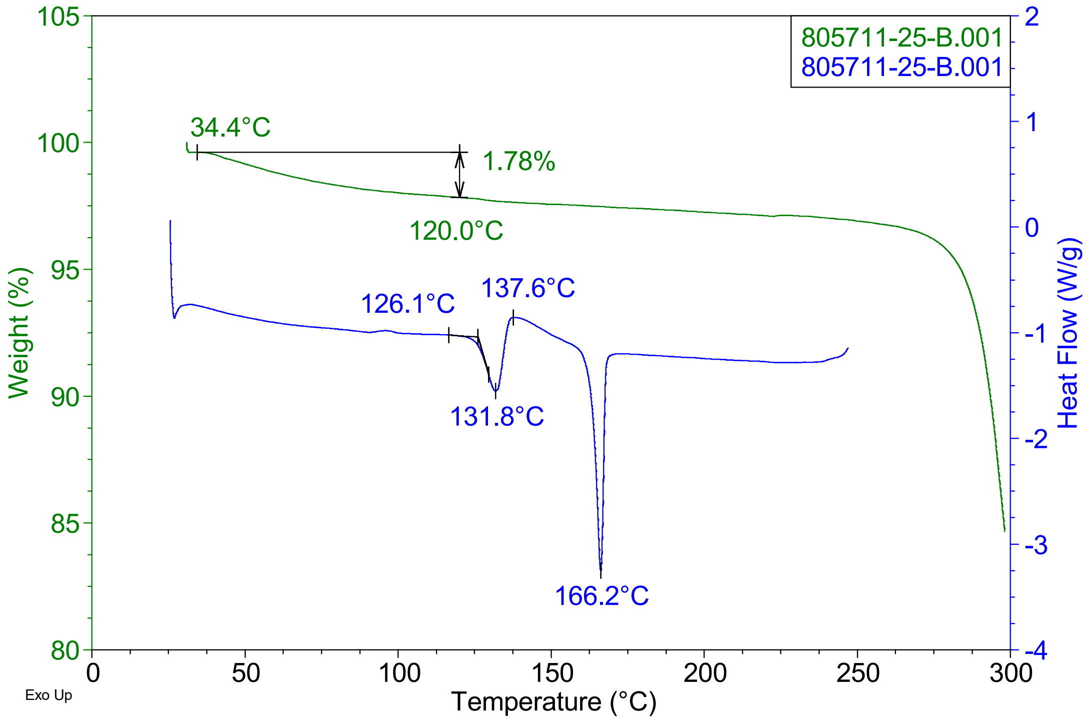

Figure 2-10 TGA/DSC curves of Type L (805711-25-B)

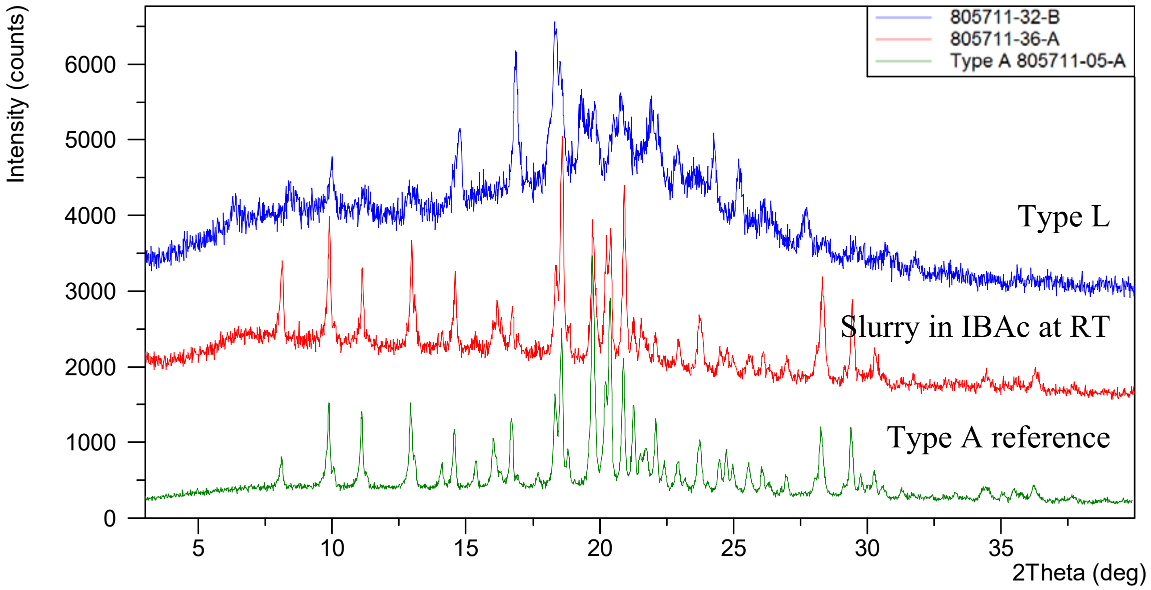

Figure 2-11 XRPD overlay of Type L (805711-32-B) pre and post slurry in IBAc at RT

2.1.5 Type M

Type M is first obtained via slurry at 100 ºC in IBAc. Type M sample (805711-37-A) was prepared by slurry of Type A at 100 ºC. XRPD pattern is shown in Figure 2-12. As per TGA and DSC data shown in Figure 2-13, a weight loss of 1.0% up to 150 ºC was observed in TGA and DSC result showed a sharp melting endotherm at 180.4 ºC (onset temperature), indicating that Type M is an anhydrate.

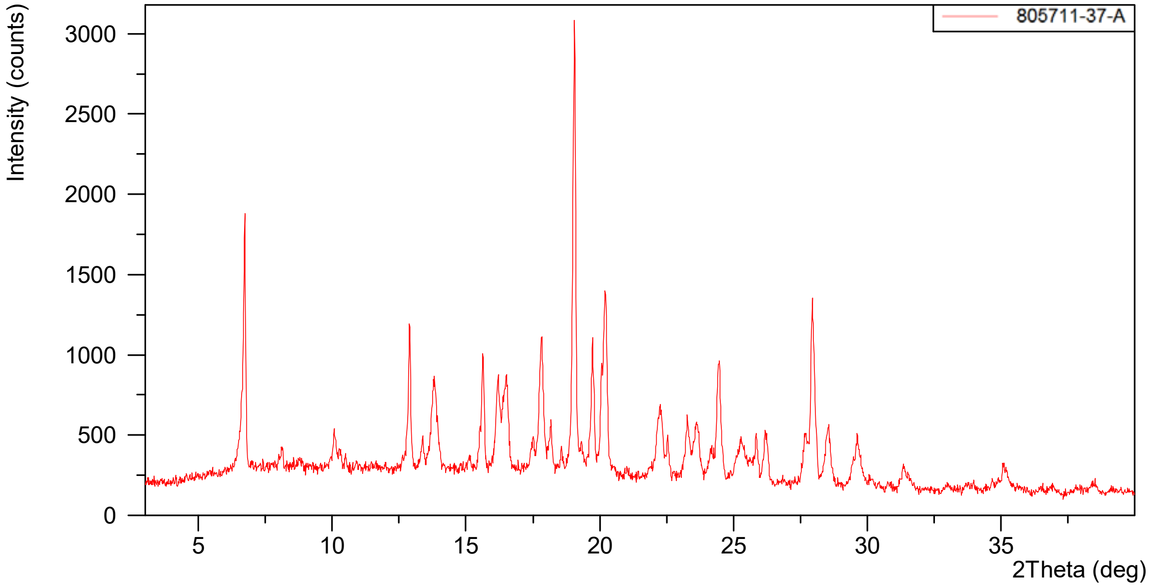

Figure 2-12 XRPD pattern of Type M (805711-37-A)

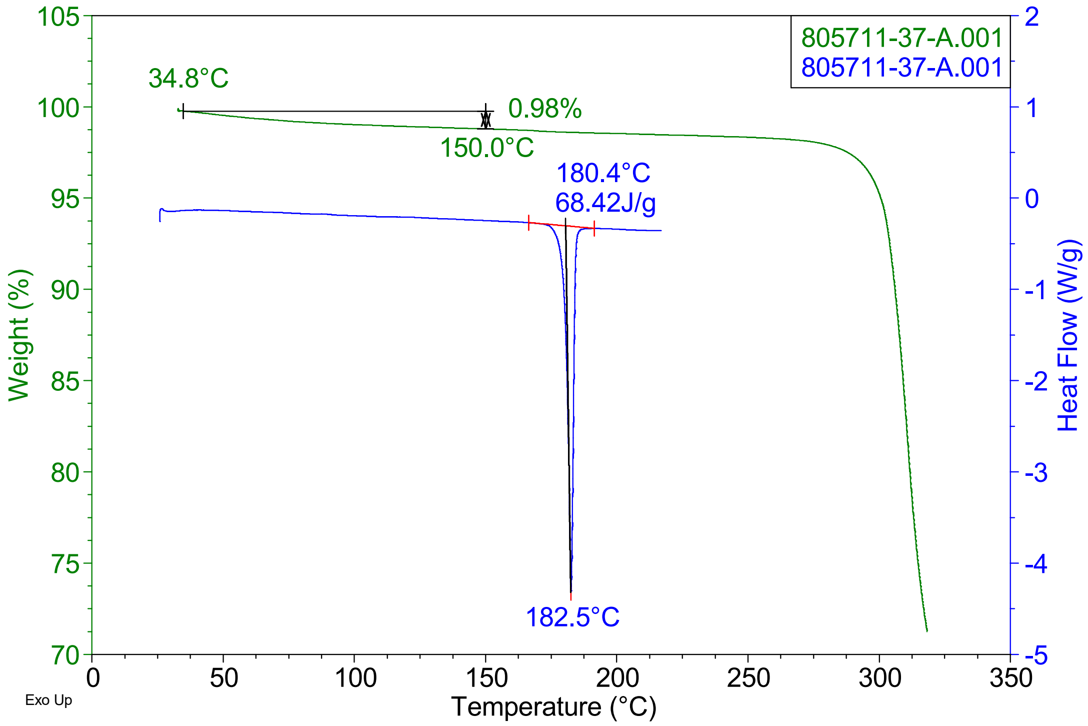

Figure 2-13 TGA/DSC curves of Type M (805711-37-A)

2.1.6 Type N

Type N can be obtained via interaction of Type A solids with 1,4-dioxane vapor. Type N sample (Bath XML2312P4, with a CP ID of 803718-05-A4) was received from Peptech on 21-Oct-2015, and its XRPD pattern is shown in Figure 2-14. As per TGA and DSC data shown in Figure 2-15, a weight loss of 1.3% up to 150 ºC was observed in TGA and DSC showed a sharp melting endotherm at 153.6 ºC (onset temperature). Based on the data collected, Type N is deemed to be an anhydrate.

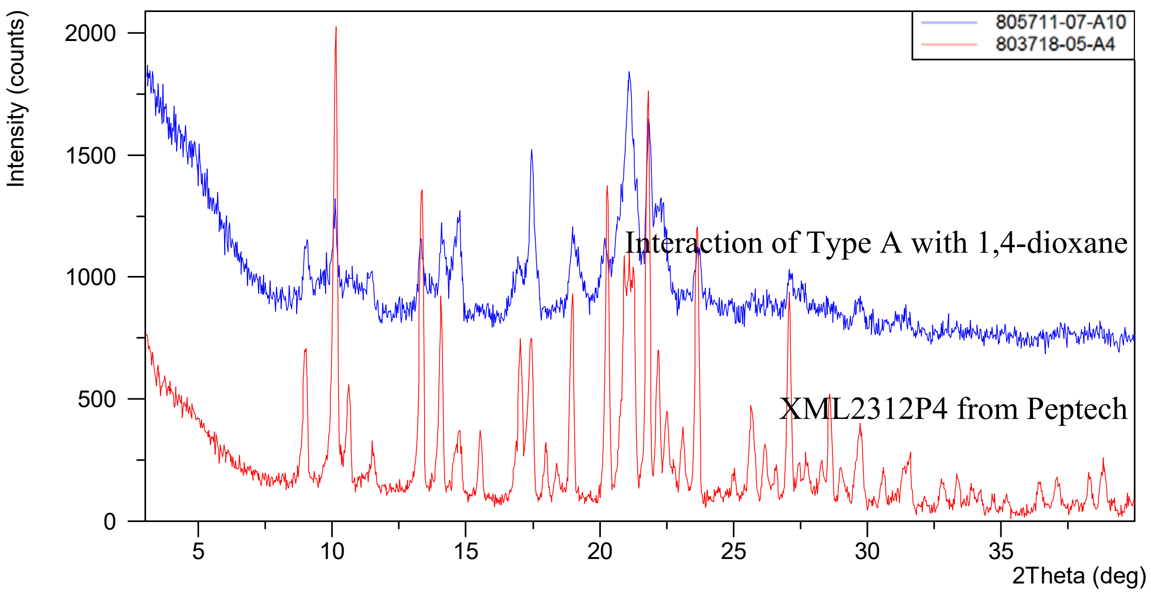

Figure 2-14 XRPD patterns of Type N batches

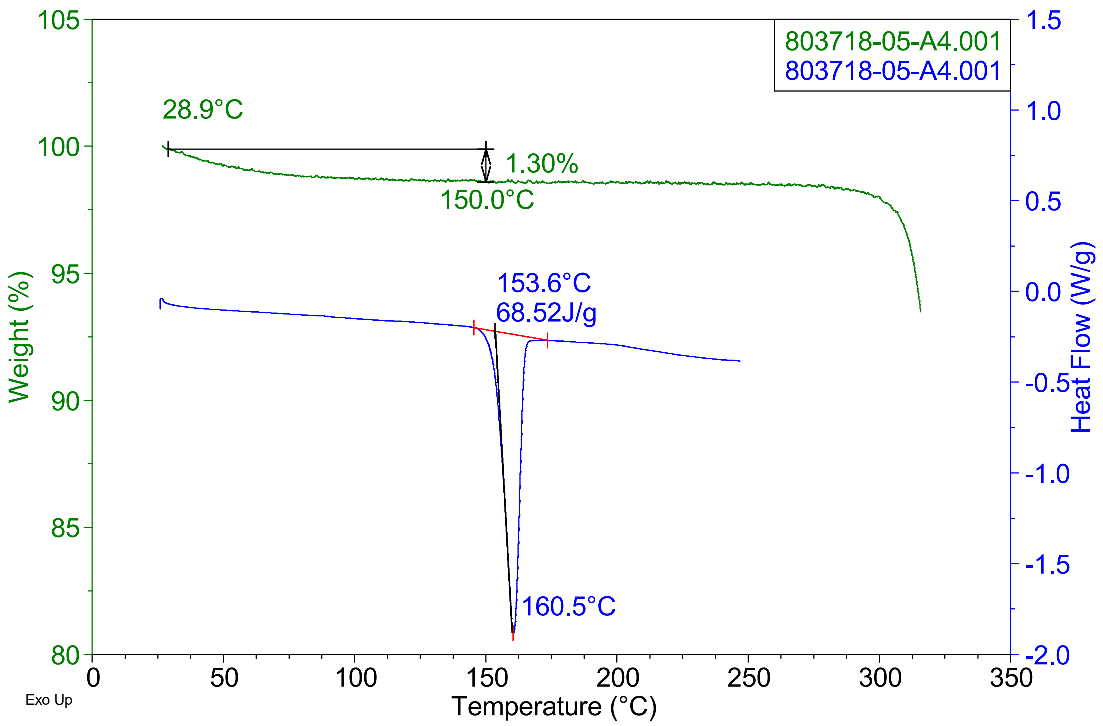

Figure 2-15 TGA/DSC curves of Type N (803718-05-A4)

2.1.7 Type P

Type P sample (805711-41-B) was obtained via air drying of wet cake Type O. Its XRPD pattern is shown in Figure 2-16. As per TGA and DSC data shown in Figure 2-17, a limited weight loss of 1.5%

up to 100 ºC was observed in TGA and DSC result showed an endotherm at 112.8 ºC (onset temperature), suggesting that Type P is an anhydrate. The minor thermal signals around 90 ºC in DSC were attributed to instrument noise.

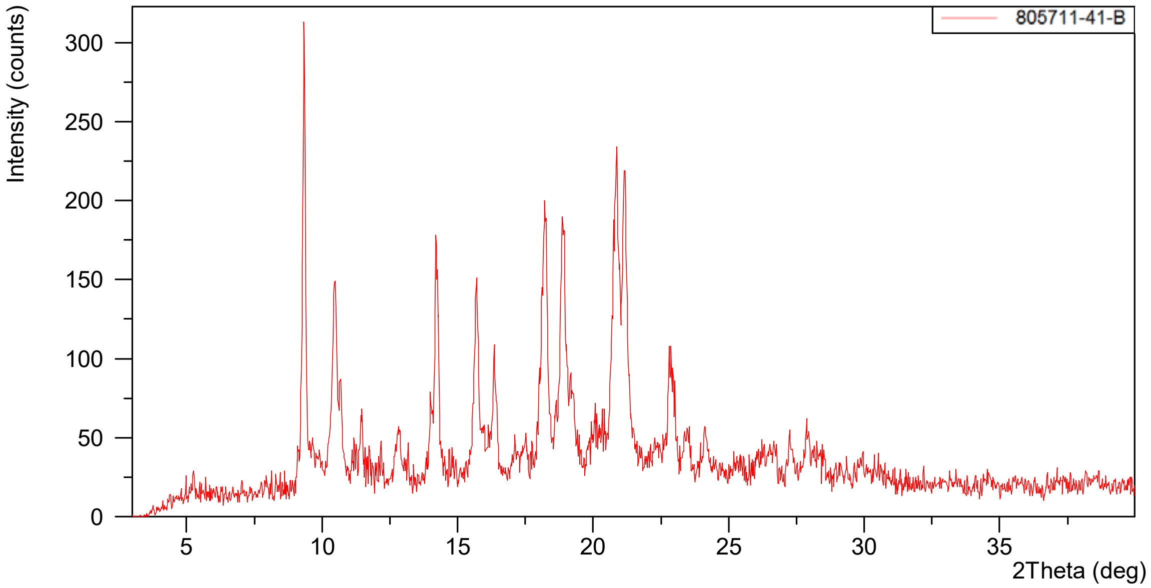

Figure 2-16 XRPD pattern of Type P (805711-41-B)

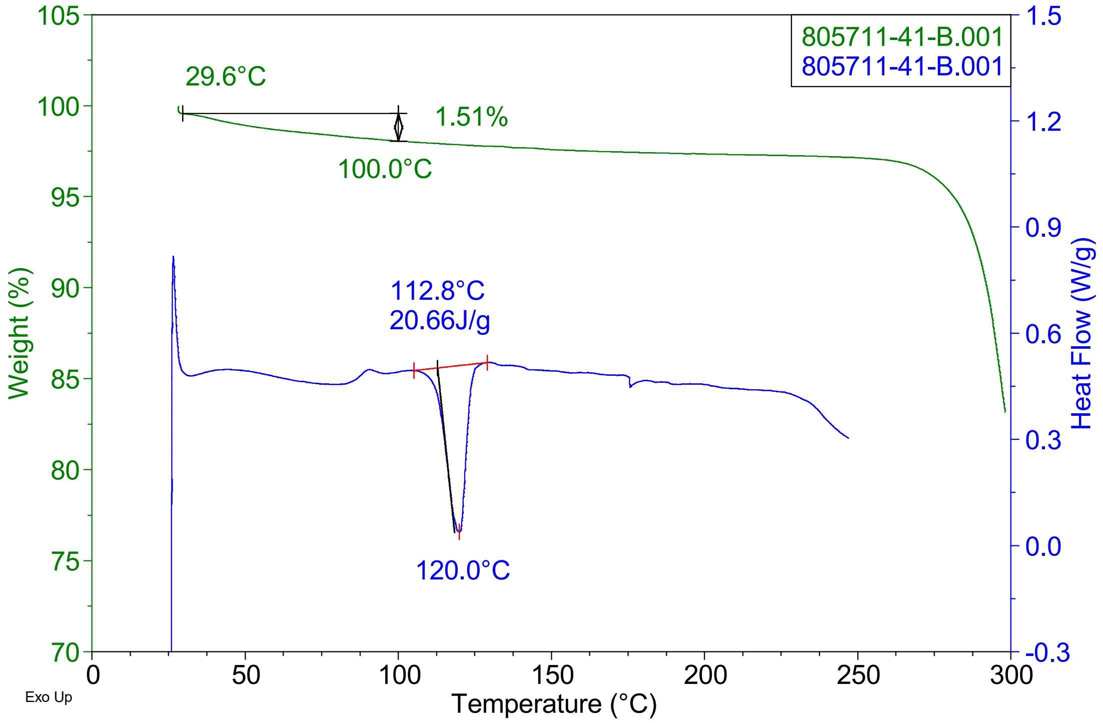

Figure 2-17 TGA/DSC curves of Type P (805711-41-B)

2.2 Hydrates

2.2.1 Type C

Type C can be isolated from aqueous systems. Two batches of Type C (805711-10-A1) and (805711-14-A1) were obtained via slurry of Type A (805711-05-A) at RT. Their XRPD patterns are shown in Figure 2-18. As per TGA and DSC data of Type C sample (805711-10-A1) shown in Figure 2-19, a weight loss of 6.0% up to 150 ºC was observed in TGA and DSC showed multiple endotherms and one possible endotherm. Therefore, Type C is speculated to be a hydrate.

Dynamic vapor sorption (DVS) was employed to evaluated its physical stability under varying humidity. Type C sample was pre-equilibrated at ambient humidity of 80%RH before test. DVS result in Figure 2-20 showed two significant platforms was observed when relative humidity decreased from 95%RH to 30%RH and dehydration occurred when relative humidity was less than 30%RH, indicating possible stability risk for Type C under varying humidity at 25 ºC. XRPD comparison showed no form change after DVS test, as shown in Figure 2-21. XRPD results of heating experiments (Figure 2-22) showed distinctive XRPD pattern was observed (assigned as Type L), after Type C sample (805711-14-A1) was heated to 120 ºC and cooled to RT under protection of nitrogen. After being heated to 150 ºC, Type C converted into Type A.

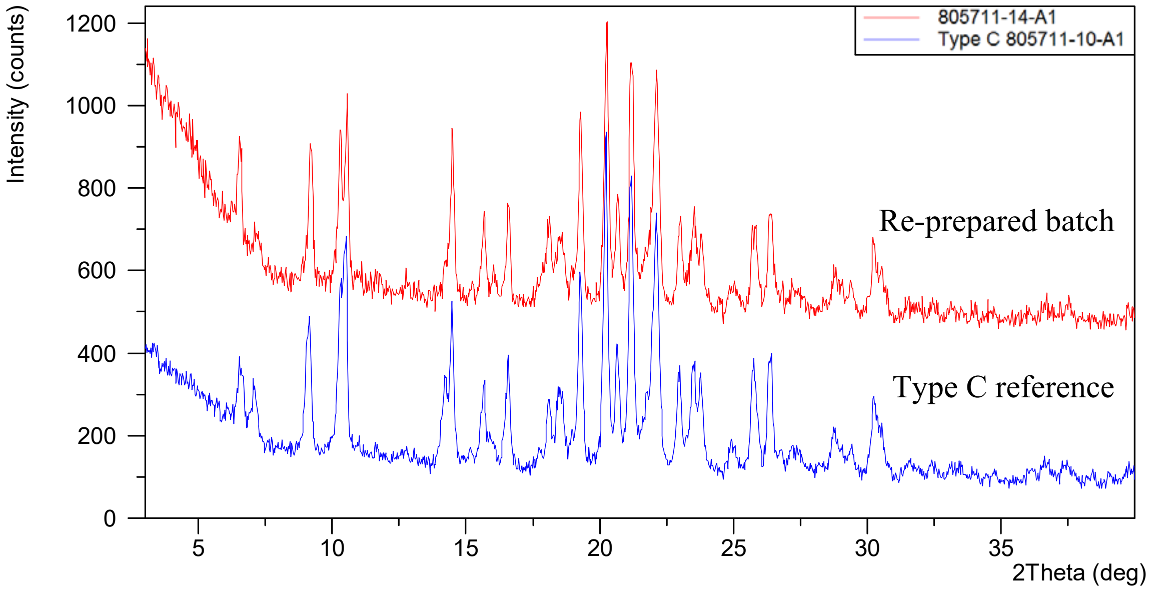

Figure 2-18 XRPD patterns of Type C batches

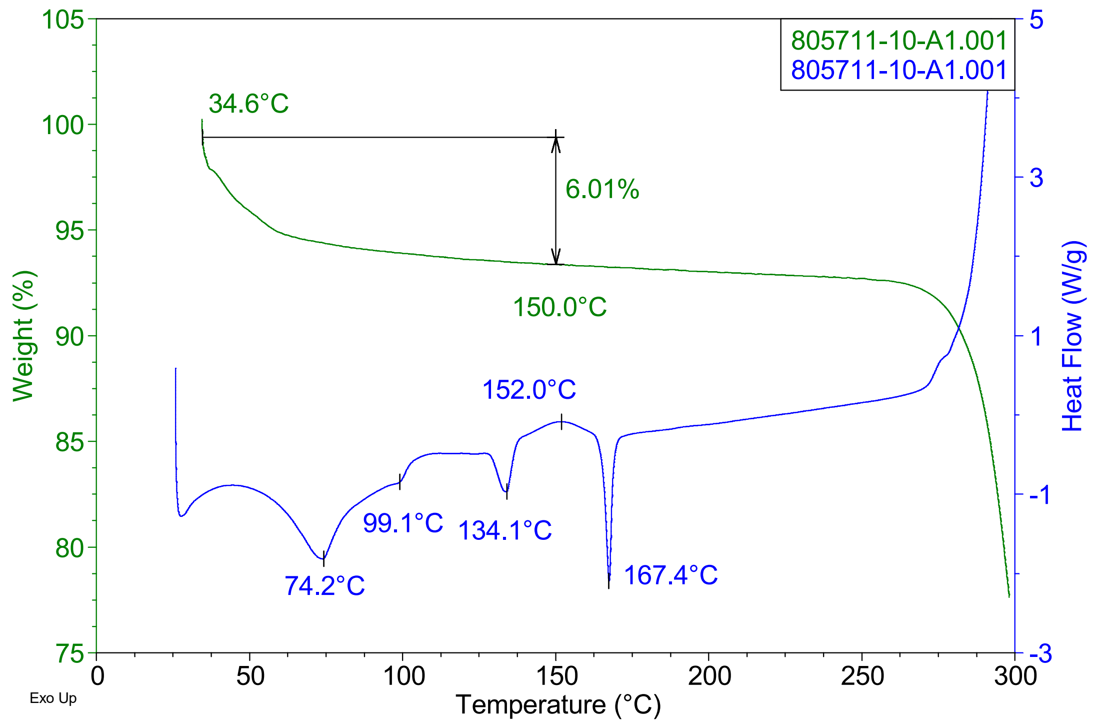

Figure 2-19 TGA/DSC curves of Type C (805711-10-A1)

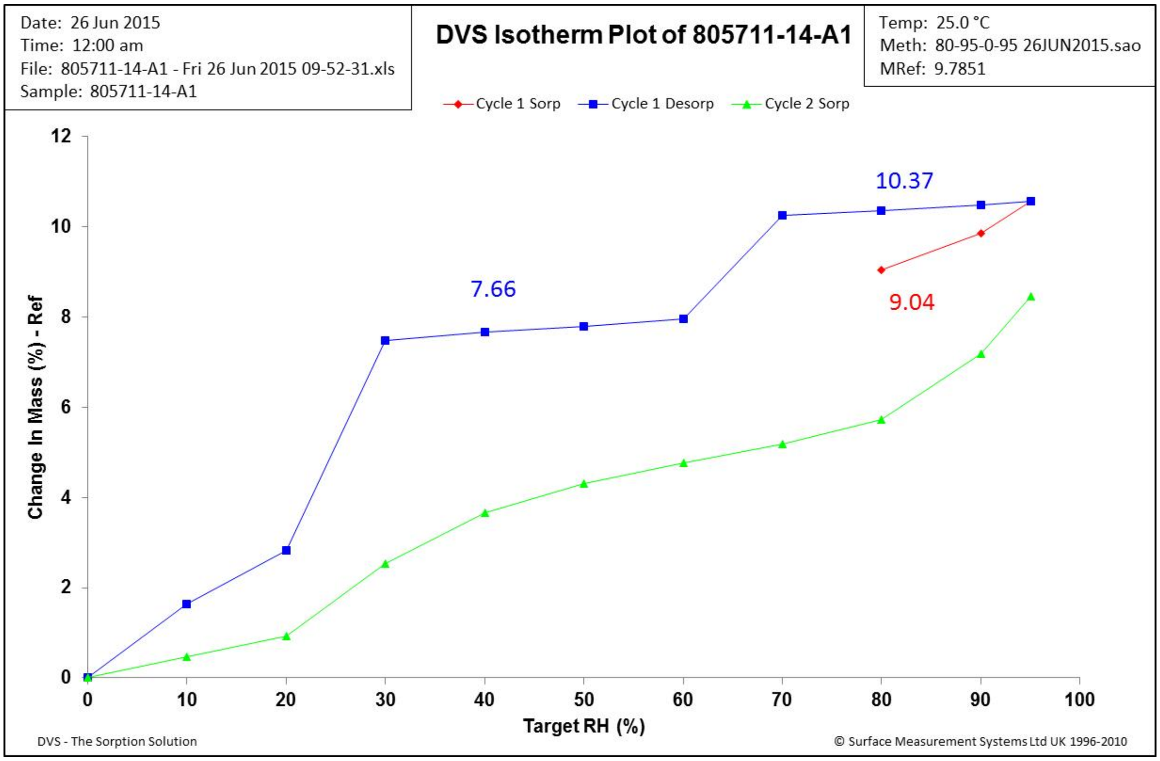

Figure 2-20 DVS plot of Type C (805711-14-A1)

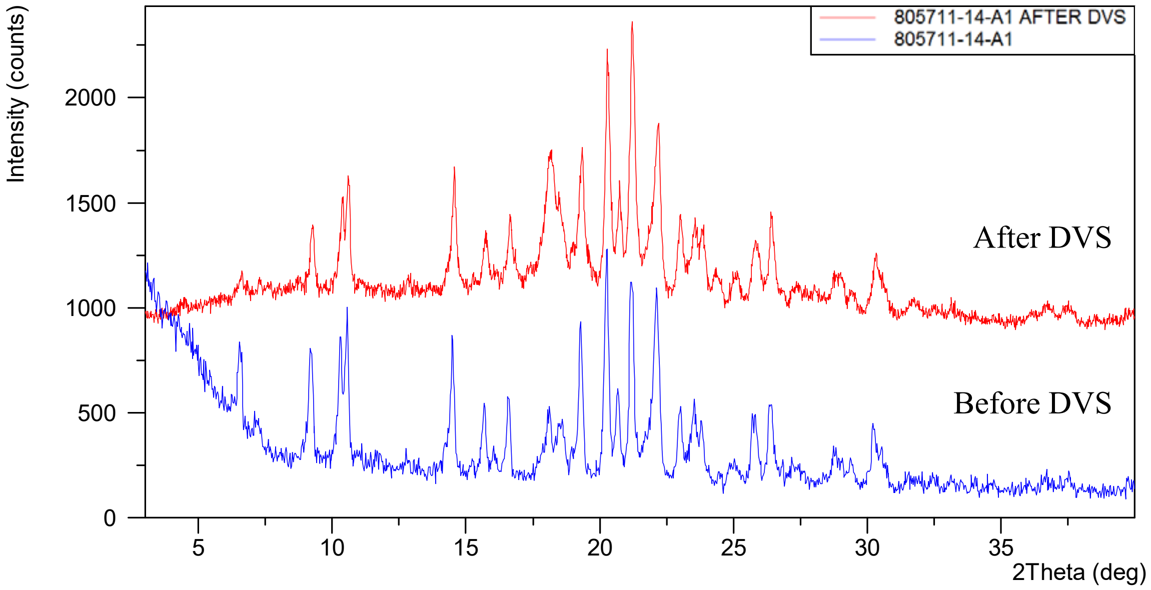

Figure 2-21 XRPD overlay of Type C (805711-14-A1) before and after DVS test

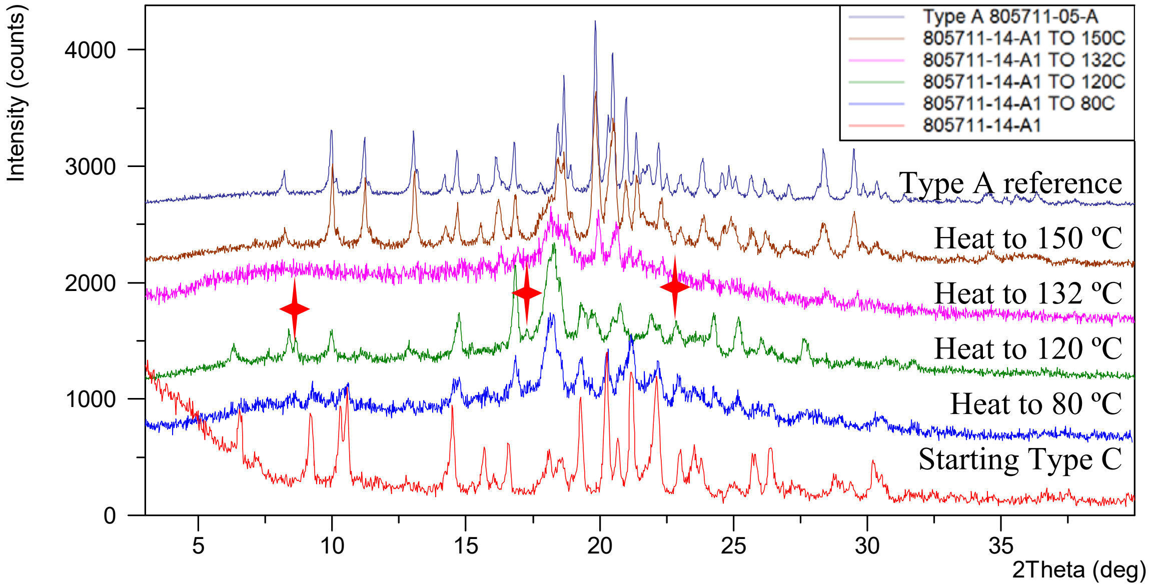

Figure 2-22 XRPD overlay of heating experiments on Type C

2.2.2 Type G

Type G can be prepared via slurry of Type A in MeOH/H_2O (v/v, 1:1) at 50 ºC or air drying of Type D. Type G sample (805711-17-A2) was produced via slurry of Type A (805711-15-A) in MeOH/H_2O (v/v, 1:1) at 50 ºC, while another batch (805711-22-B) was obtained via slurry of Type A (805711-15-A) in MeOH at RT, followed by drying the isolated cake at 50 ºC. As per TGA and DSC data of

Type G (805711-17-A2), a weight loss of 3.8% up to 100 ºC was observed in TGA and DSC result showed two endotherms at 77.2 and 120.4 ºC (peak temperature).

To investigate the significant weight loss and broad endotherm, heating experiment and *in situ* VT-XRPD were conducted on Type G sample. As shown in Figure 2-25, no form change was observed after Type G sample (805711-17-A2) was heated to 100 ºC, cooled to RT and re-exposed to air. *In situ* VT-XRPD is shown in Figure 2-26. After being heated to elevated temperatures under protection of nitrogen, distinctive diffraction peaks highlighted were observed. Therefore, Type G is speculated to be a hydrate and bound water can be absorbed back readily at ambient conditions. The dehydrated form of Type G is named as Type G0 (805711-23-B).

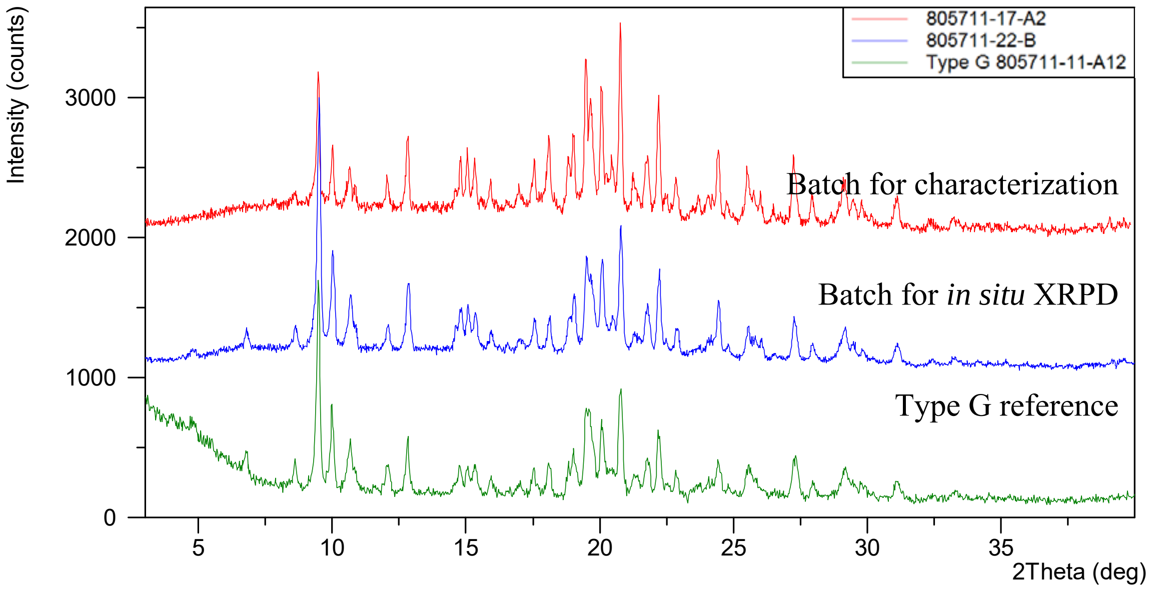

Figure 2-23 XRPD patterns of different batches of Type G

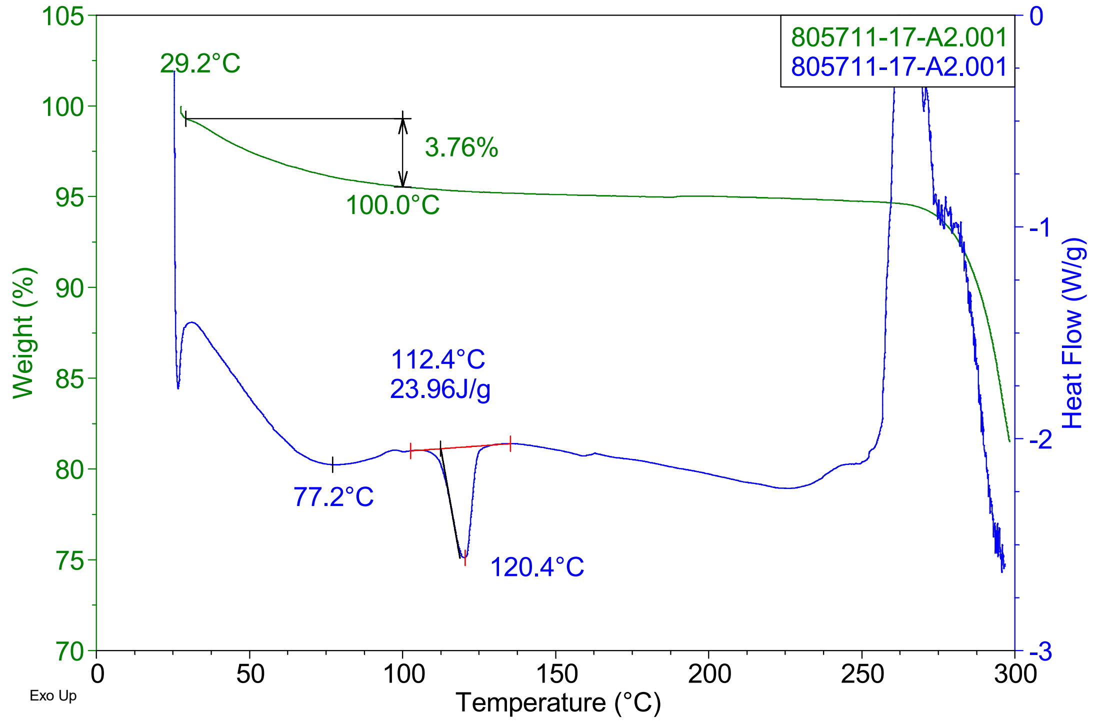

Figure 2-24 TGA/DSC curves of Type G (805711-17-A2)

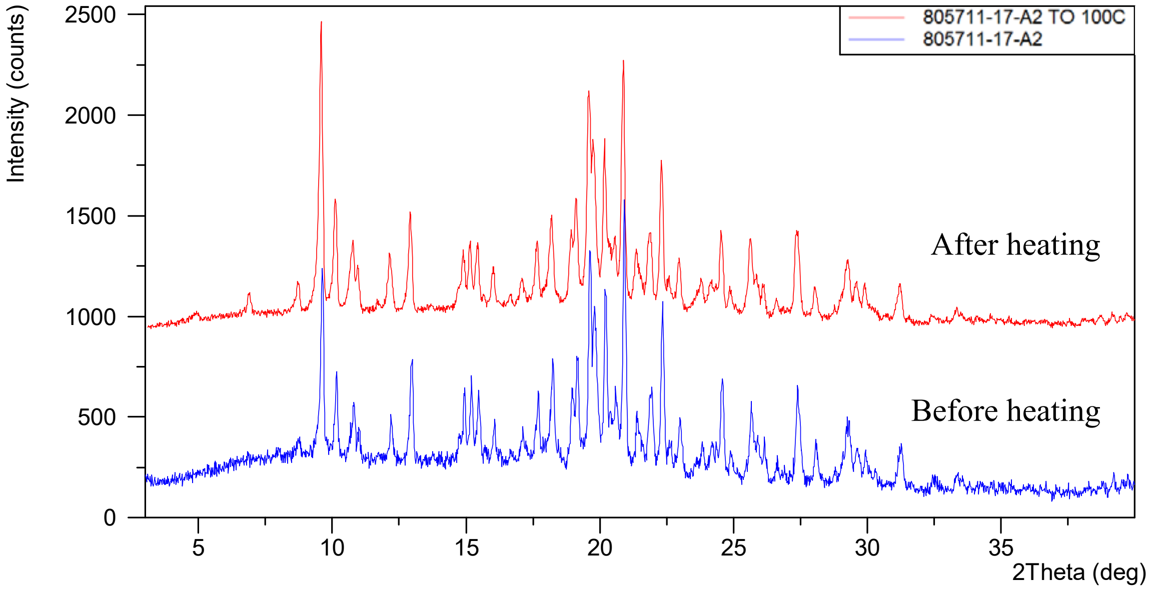

Figure 2-25 XRPD overlay of Type G (805711-17-A2) before and after heating

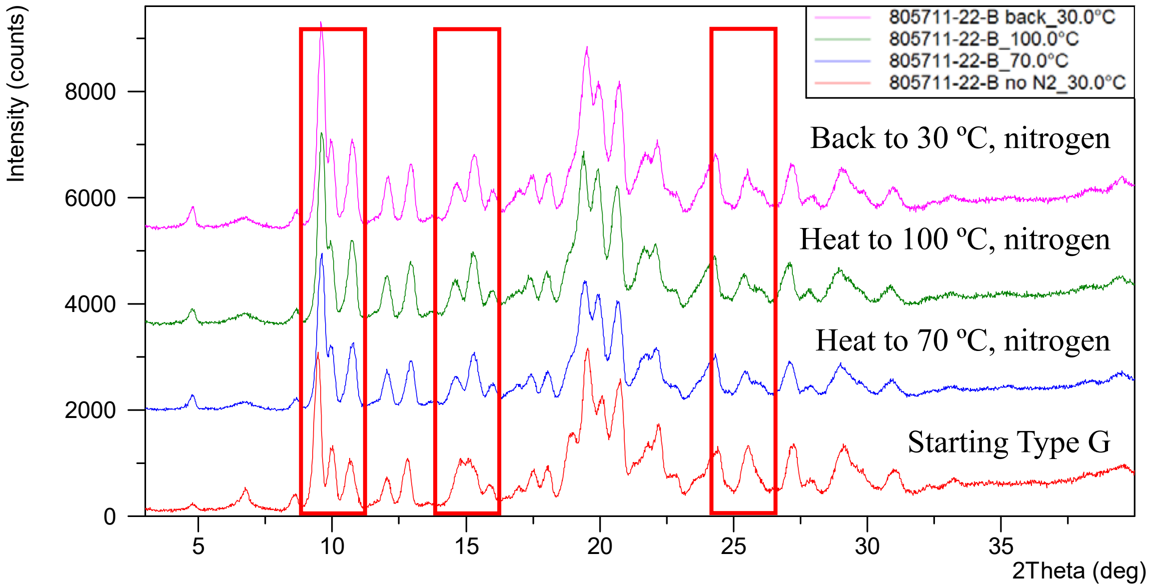

Figure 2-26 *In situ* VT-XRPD overlay of Type G (805711-22-A1) at variable temperatures

2.2.3 Type U

Type U sample (805711-09-B) was obtained via air drying of transient form Type T. XRPD pattern is shown in Figure 2-27. As per TGA and DSC data (Figure 2-28), a weight loss of 3.5% up to 100 ºC was observed in TGA and DSC showed two endotherms at 68.9 and 110.4 ºC (peak temperature). To investigate the significant weight loss in TGA, ^{1}H NMR and heating experiment were conducted on Type U sample. ^{1}H NMR result (Figure 2-29) showed no MeOH was detected, suggesting Type U is non-solvated. No form change was observed after Type G sample (805711-09-B) was heated to 90 ºC and cooled to RT under protection of nitrogen, as shown Figure 2-30. From the above data, Type U is speculated to be a hydrate and bound water can be absorbed back readily at ambient conditions.

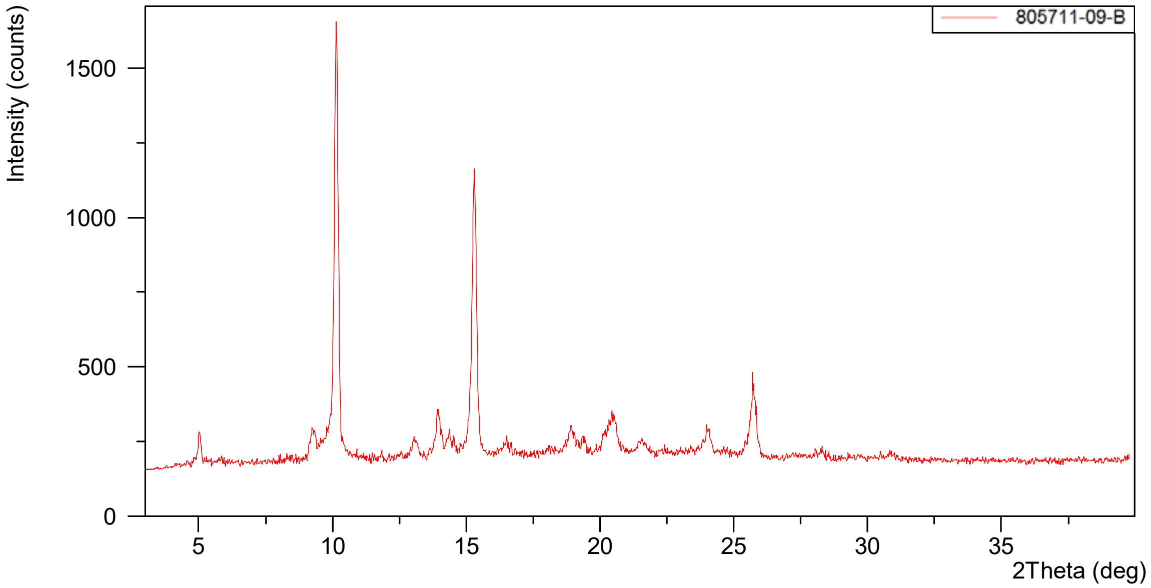

Figure 2-27 XRPD pattern of Type U (805711-09-B)

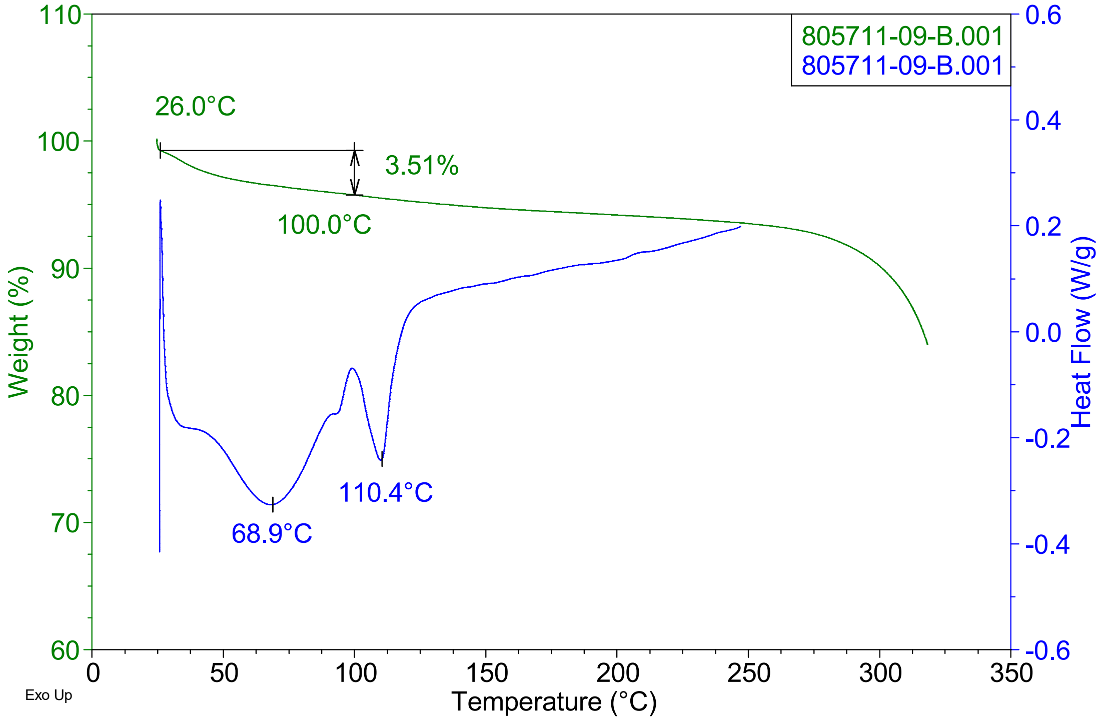

Figure 2-28 TGA/DSC curves of Type U (805711-09-B)

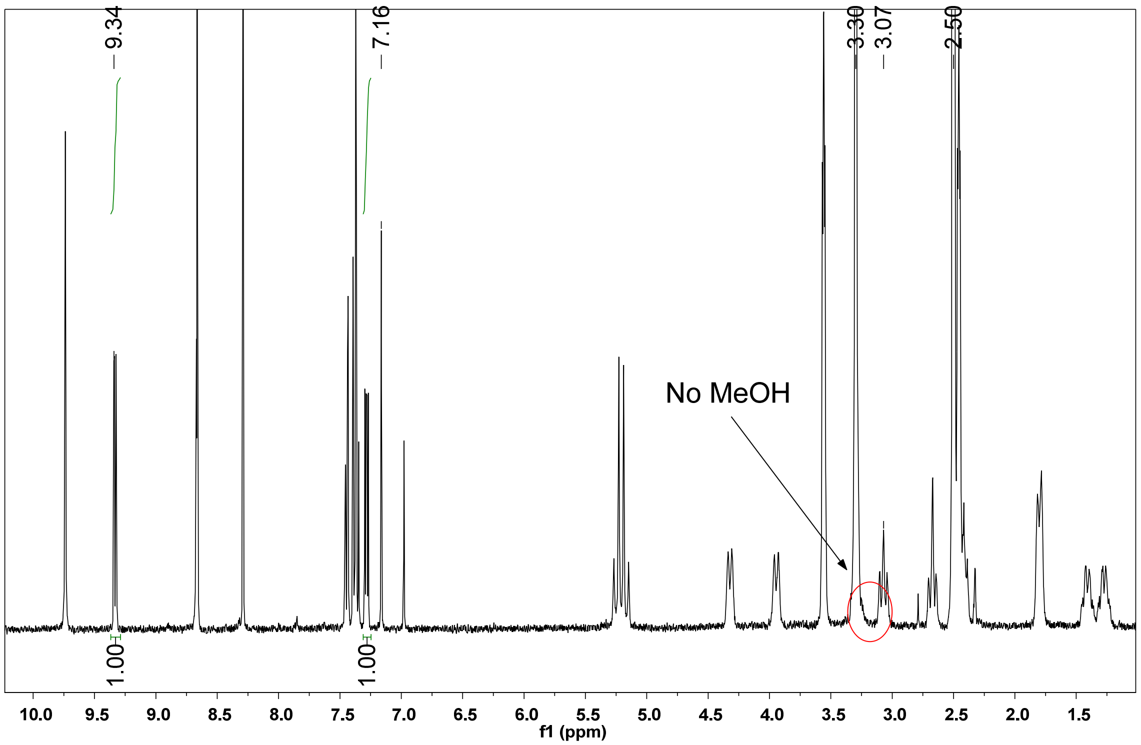

Figure 2-29 ^{1}H NMR spectrum of Type U (805711-09-B)

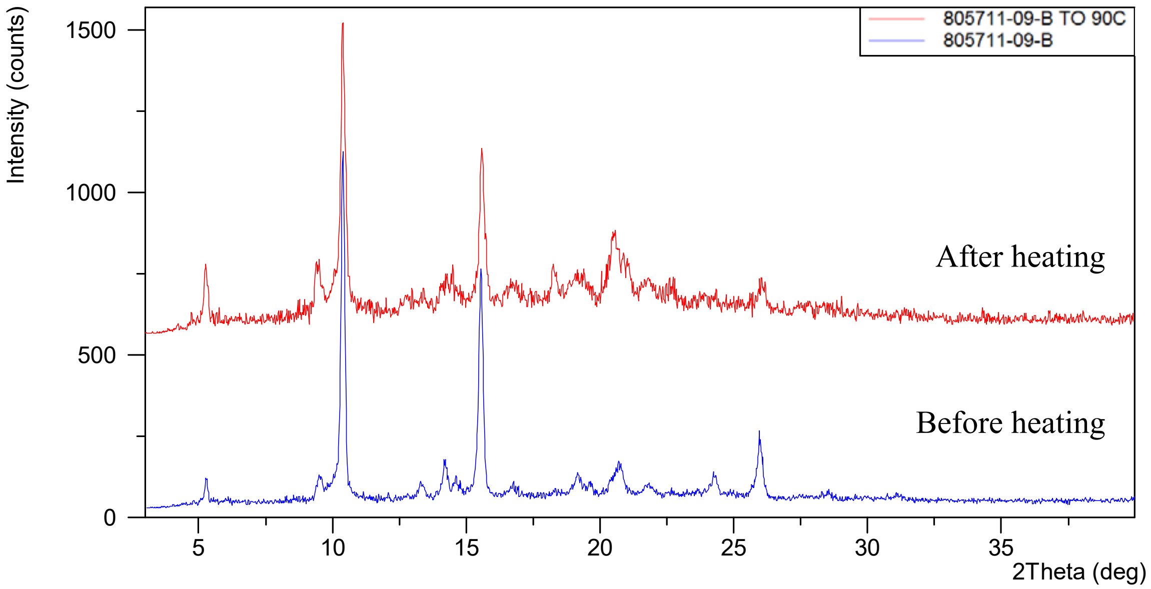

Figure 2-30 XRPD overlay of Type U (805711-09-B) before and after heating

2.2.4 Type X

Type X sample could be obtained via slow evaporation in DCM/toluene mixture at RT, or air drying of Type W that crystallized from ACN/H_2O. XRPD patterns are displayed in Figure 2-31. As per

TGA and DSC data of Type X (805711-08-B) displayed in Figure 2-32, a weight loss of 3.0% up to 150 ºC was observed in TGA and DSC showed four endotherms at 66.3, 90.9, 132.1 and 165.1 ºC (peak temperature).

^{1}H NMR result (Figure 2-33) showed that no solvent ACN was detected for Type X sample (805711-08-B), indicating the non-solvated structure. Heating experiments were conducted on Type X sample (805711-13-A11). As shown in Figure 2-34, Type X converted into a mixture of Type X and L after being heated to 100 ºC, and converted into Type A after being heated to 150 ºC. Based on the data collected, Type X is speculated to be a hydrate.

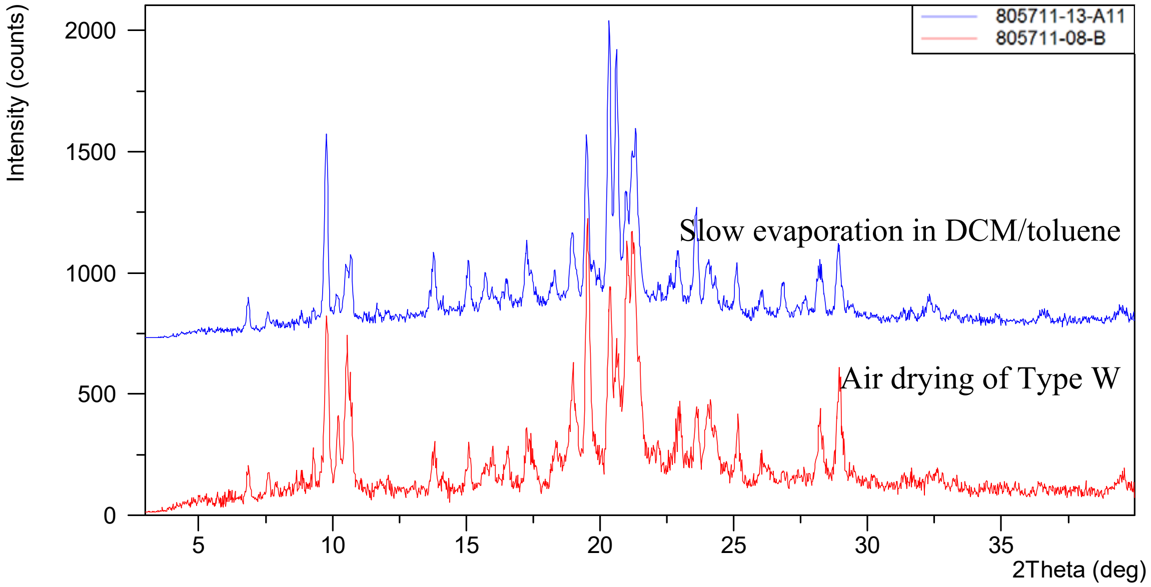

Figure 2-31 XRPD patterns of Type X batches

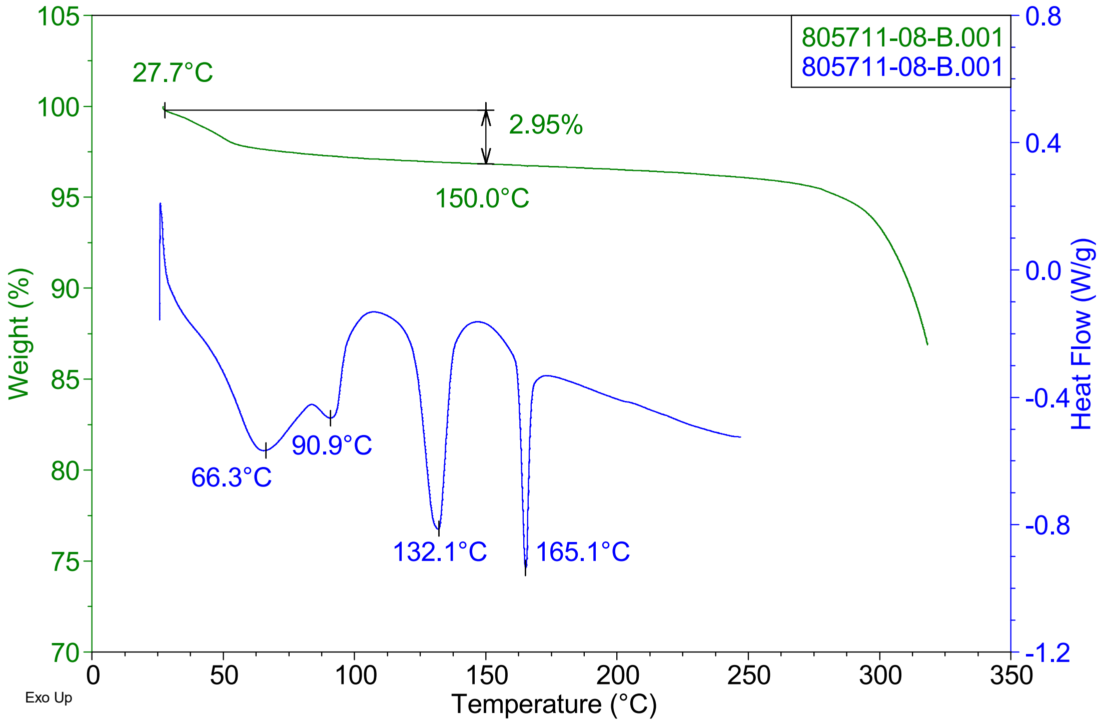

Figure 2-32 TGA/DSC curves of Type X (805711-08-B)

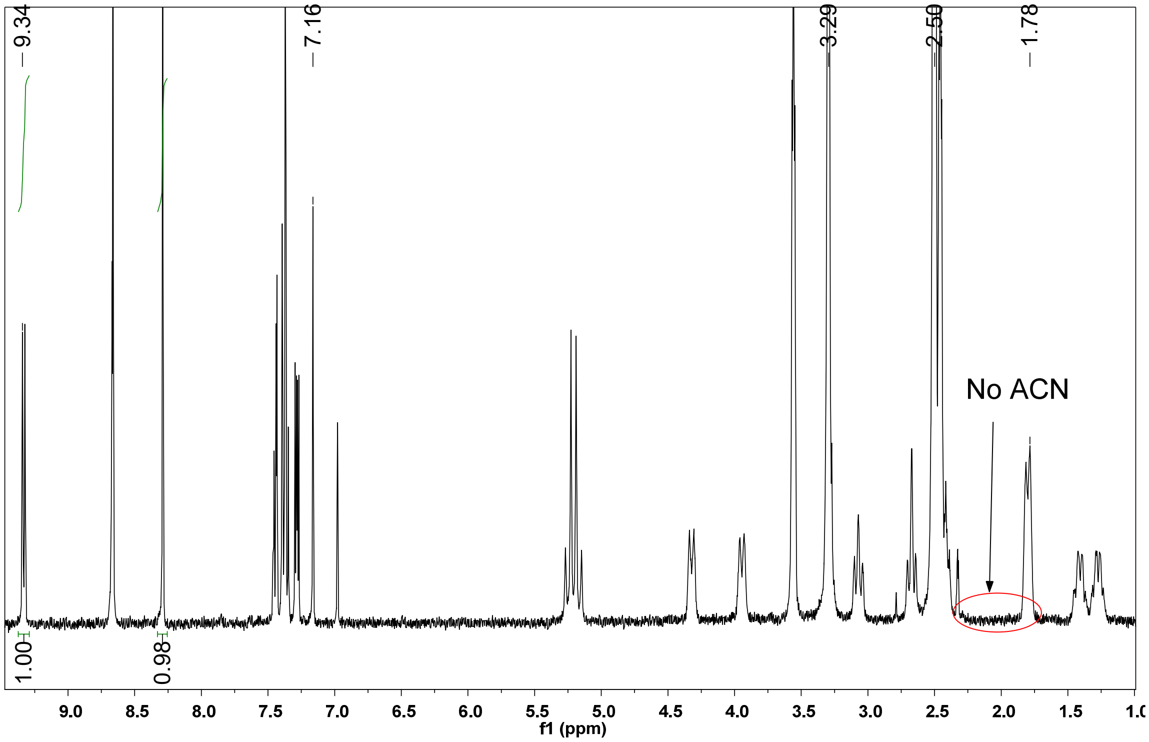

Figure 2-33 ^{1}H NMR spectrum of Type X (805711-08-B)

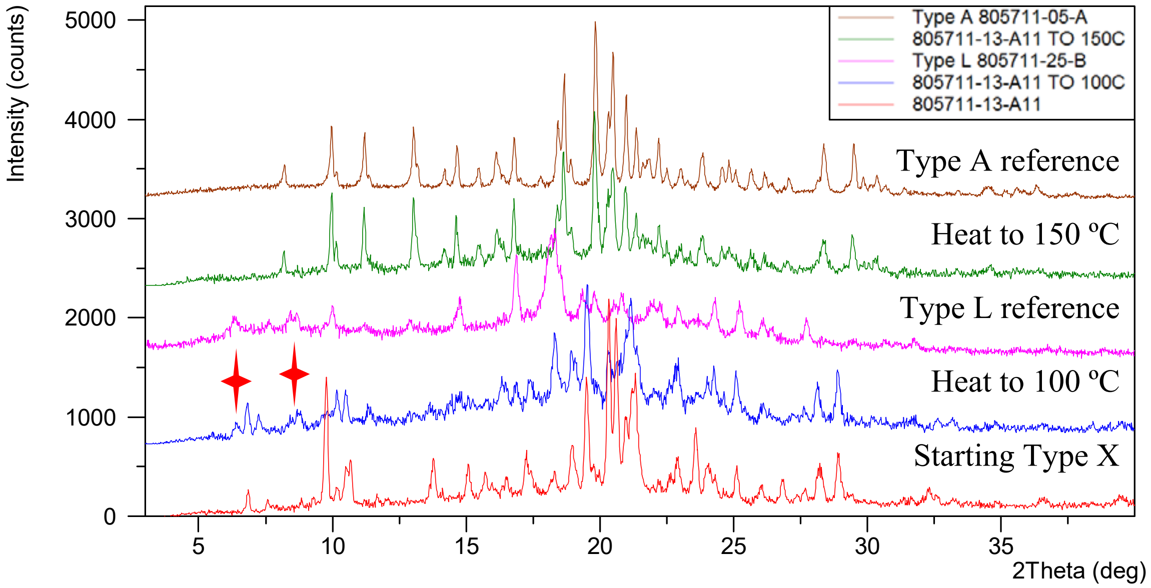

Figure 2-34 XRPD overlay of Type X (805711-13-A11) before and after heating

3 Evaluation of Anhydrates

Based on the solid-state characterization data and preliminary form identity, eight re-producible anhydrates were identified so far. Experiments were set up to establish their stability relationships and to evaluate the dissolution difference compared to starting material Type A.

3.1 Thermodynamic Stability Relationship

3.1.1 Type A, K and H

To determine the stability relationships of Type A, K and H, slurry competition experiments were performed at different temperatures of RT (~25 ºC), 50, 70 and 100 ºC. Detailed procedures are described in Table 3-1. Pure Type H was obtained at all tested temperatures, indicating higher thermodynamic stability of Type H from RT to 100 ºC, compared to Type A and K. XRPD patterns are shown in Figure 3-1 and Figure 3-2, with results summarized in Table 3-2.

Table 3-1 Summary of specific procedures of competitive slurries

Steps	Specific Procedures of Slurry Competition	
	RT and 50 ºC	70 ºC and 100 ºC
1	Weigh ~ 5 mg of Type A (805711-15-A), Type H (805711-17-A1) and Type K (805711-18-A2) into a HPLC vial.	Weigh ~ 5 mg of Type A (805711-28-A), Type H (805612-12-A) and a mixture of Type F and K (805711-19-A) into a HPLC vial.
2	Prepare saturated EtOAc solution at 50 ºC using Type A solids (805711-15-A) by magnetic stirring for 1 hr.	Prepare saturated IBAc solution at 100 ºC using Type A solids (805711-28-A) by magnetic stirring for 1 hr.
3	Distribute ~ 0.8 mL of EtOAc solution in Step 2 through 0.45 µm Nylon filter into the mixture in Step 1.	Distribute ~ 0.5 mL of IBAc solution in Step 2 through 0.45 µm Nylon filter into the mixture in Step 1.
4	Stir magnetically at RT and 50 ºC.	Stir magnetically at 70 and 100 ºC.
5	Centrifuge and sample for XRPD analysis.	Centrifuge and sample for XRPD analysis.

Table 3-2 Summary of competitive slurries of Type A, H and K

Starting Mixture	Temperature (ºC)	Solvent	Sampling Time (day)	Final Form
Type A, H and K	RT	EtOAc	13	Type H
	50		2	Type H
	70	IBAc	1	Type H
	100		1	Type H

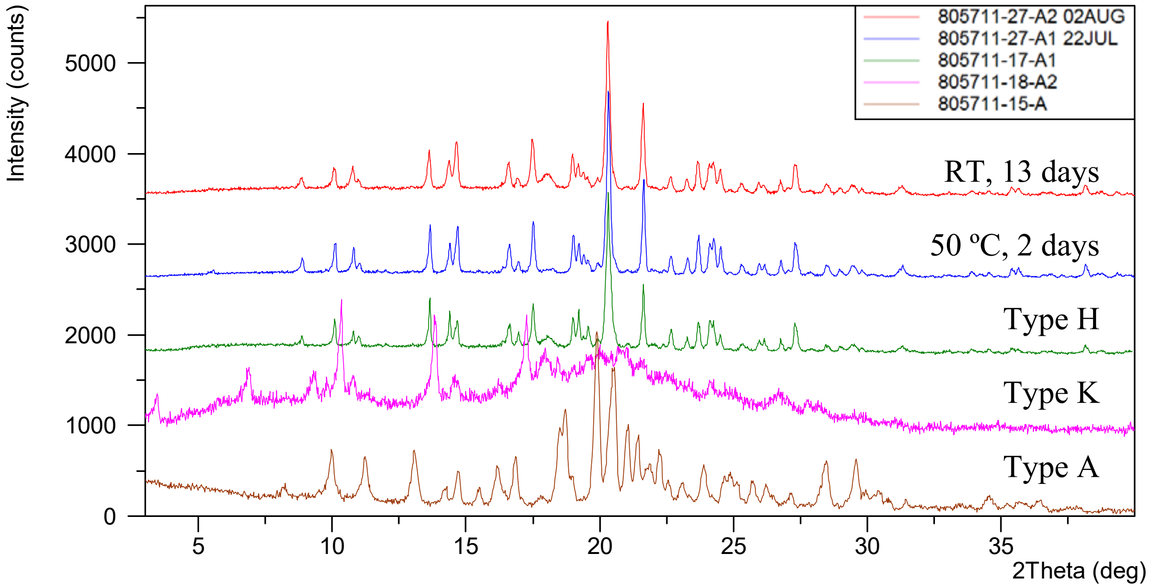

Figure 3-1 XRPD overlay for slurry competition of anhydrates Type A, H and K at RT and 50 ºC

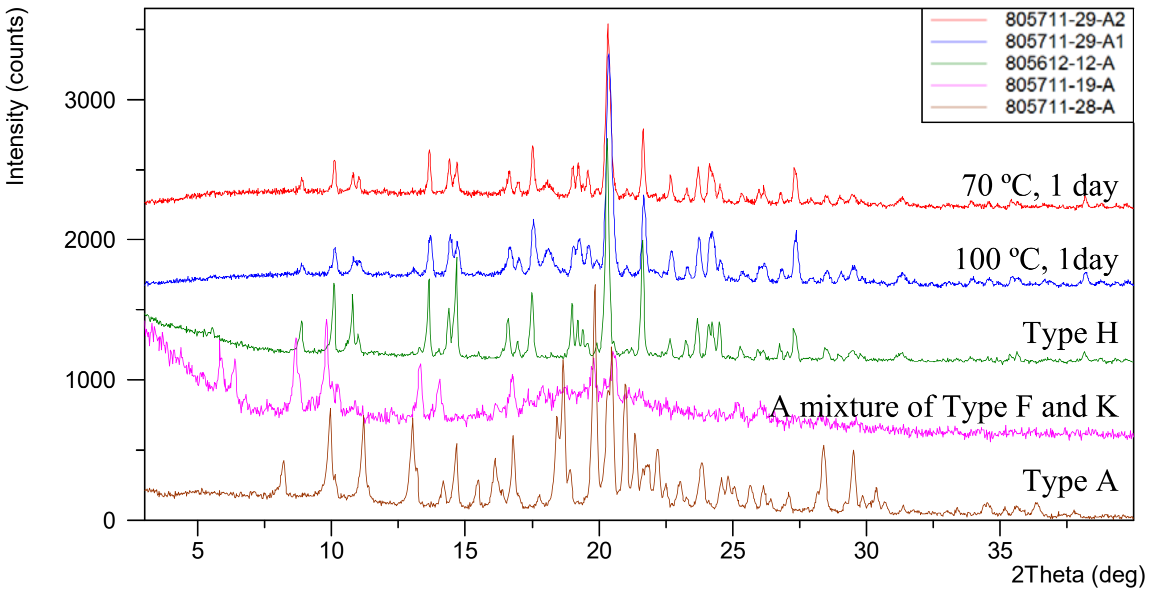

Figure 3-2 XRPD overlay for slurry competition of anhydrates Type A, H and K at 70 and 100 ºC

3.1.2 Type H, N and M

To determine the stability relationships of Type H, N and M, slurry competition experiments were performed at different temperatures of RT, 50 and 70 ºC. Detailed procedures are described in Table 3-3. Pure Type M was obtained at all tested temperatures, indicating higher thermodynamic stability of Type M from RT to 70 ºC, compared to Type H and N. XRPD patterns are shown in Figure 3-3 and Figure 3-4, with results summarized in Table 3-4.

Table 3-3 Summary of specific procedures of competitive slurries

Steps	Specific Procedures of Slurry Competition at RT, 50 and 70 ºC	
	Type M and H	Type M and N
1	Weigh ~ 5 mg of Type H (805711-35-B) and Type M (805711-37-A) into a HPLC vial.	Weigh ~ 5 mg of Type N (803718-05-A4) and Type M (803718-06-A) into a HPLC vial.
2	Prepare saturated IPAc solution at 70 ºC using Type A solids (805711-28-A) by magnetical stirring for 1 hr.	Prepare saturated IPAc solution at 70 ºC using Type K solids (803718-05-B) by magnetic stirring for 3 hr.
3	Distribute ~ 0.5 mL of EtOAc solution in Step 2 through 0.45 μm Nylon filter into the mixture in Step 1.	Distribute ~ 0.5 mL of IPAc solution in Step 2 through 0.45 μm Nylon filter into the mixture in Step 1.
4	Stir magnetically at RT, 50 and 70 ºC, respectively.	Stir magnetically at RT, 50 and 70 ºC, respectively.
5	Centrifuge and sample for XRPD analysis.	Centrifuge and sample for XRPD analysis.

Table 3-4 Summary of competitive slurries of anhydrates Type H, N and M

Starting Mixture	Temperature (ºC)	Solvent	Sampling Time (day)	Final Form
Type M and H	RT	IPAc	3	Type M
	50		3	Type M
	70		3	Type M
Type M and N	RT	IPAc	8	Type M
	50		1	Type M
	70		1	Type M

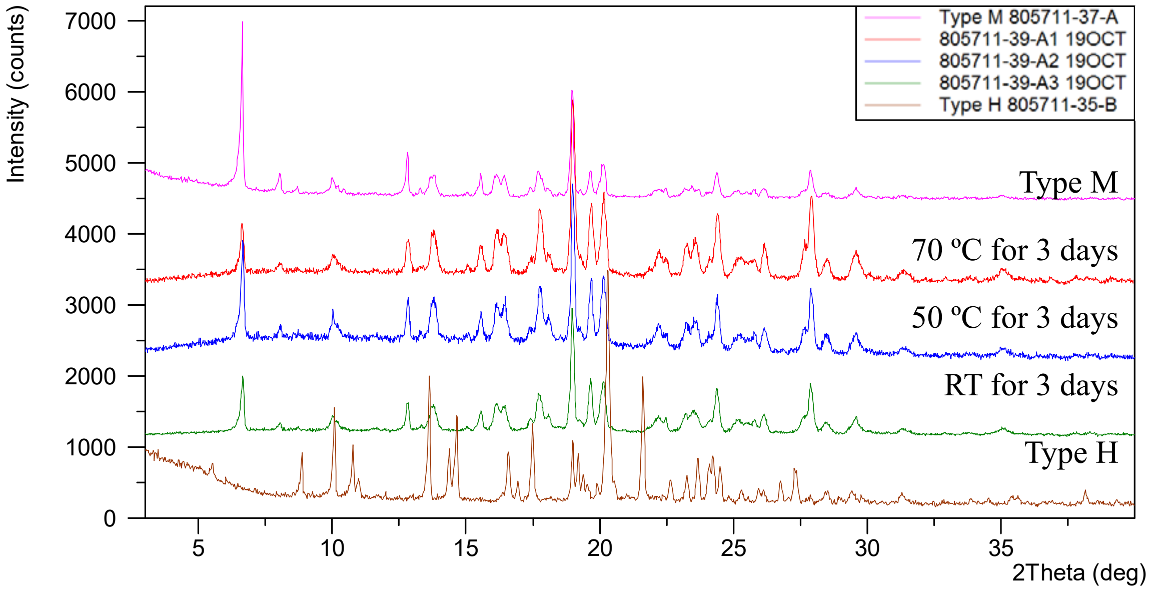

Figure 3-3 XRPD overlay for slurry competition of anhydrates Type M and H

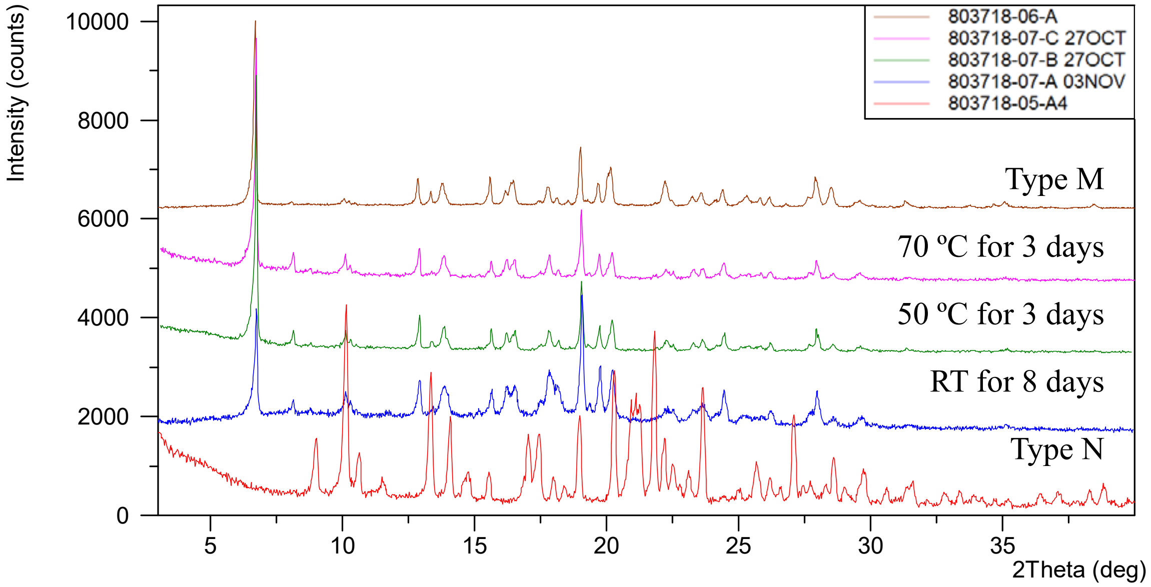

Figure 3-4 XRPD overlay for slurry competition of anhydrates Type M and N

3.1.3 Type R, P and M

To investigate the thermodynamic stability relationship of Type R, P and M, slurry competition experiments were performed at RT. Detailed procedures are described in Table 3-5. Pure Type M was obtained at both conditions, indicating higher thermodynamic stability of Type M at RT, compared to Type R and P. XRPD patterns are shown in Figure 3-5 and Figure 3-6, with results summarized in

Table 3-6.

Combined with that Type M possesses the highest melting point among all identified anhydrates, Type M is deemed to be the stable anhydrate above RT, and thus recommended for further development.

Table 3-5 Summary of specific procedures of competitive slurries

**Steps**	**Specific Procedures of Slurry Competition at RT**	
	Type M and R	Type M and P
1	Weigh ~ 5 mg of Type R (805711-41-A2) and Type M (803718-46-B) into a HPLC vial.	Weigh ~ 5 mg of Type P (805711-41-B) and Type M (803718-46-B) into a HPLC vial.
2	Prepare saturated EtOAc solution at RT using Type M solids (803718-46-B) by magnetic stirring for 1 hr.	Prepare saturated EtOAc solution at RT using Type M solids (803718-46-B) by magnetic stirring in for 1 hr.
3	Distribute ~ 0.5 mL of EtOAc solution in Step 2 thorugh 0.45 μm Nylon filter into the mixture in Step 1.	Distribute ~ 0.5 mL of IPAc solution in Step 2 through 0.45 μm Nylon filter into the mixture in Step 1.
4	Stir magnetically at RT.	Stir magnetically at RT.
5	Centrifuge and sample for XRPD analysis.	Centrifuge and sample for XRPD analysis.

Table 3-6 Summary of competitive slurries of anhydrates

**Starting Mixture**	**Temperature (°C)**	**Solvent**	**Sampling Time (day)**	**Final Form**
Type M and R	RT	EtOAc	5	Type M
Type M and P	RT	EtOAc	2	Type M

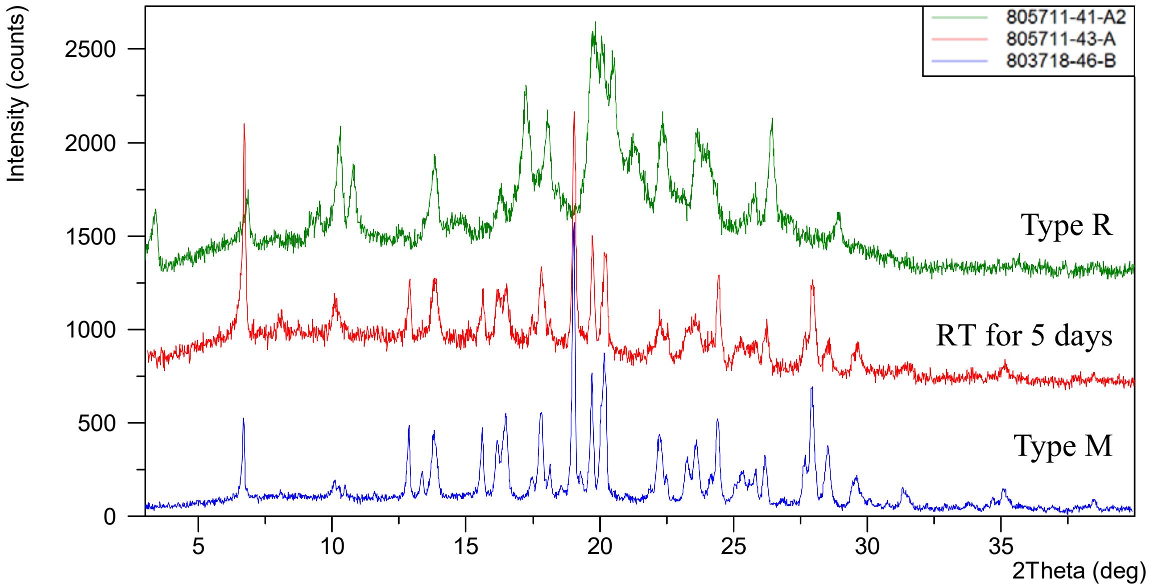

Figure 3-5 XRPD overlay for slurry competition of anhydrates Type M and R

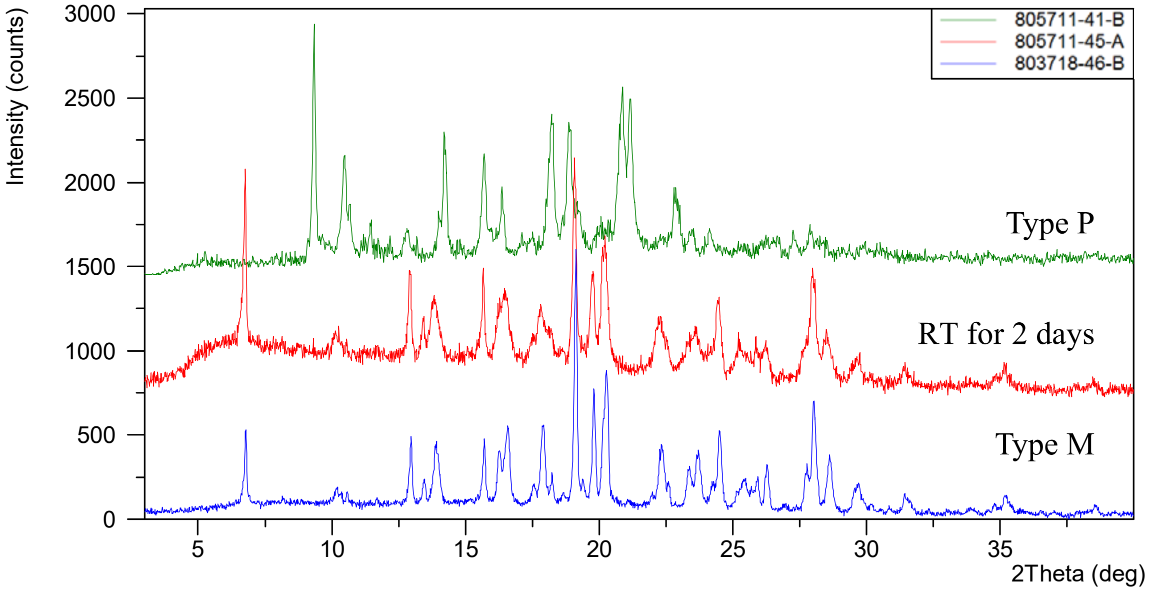

Figure 3-6 XRPD overlay for slurry competition of anhydrates Type M and S

3.2 Kinetic Solubility

3.2.1 Type A and H

Type H is thermodynamically more stable than Type A at RT~100 ºC. In order to determine solubility difference of Type A and H in vitro, kinetic solubility was measured in water and pH 6.5 phosphate buffer at 37 ºC. Suspensions with an initial solid loading of 3.5 mg/mL were kept rolling with a speed

of 25 rpm, with filtrates for HPLC and pH tests and solids for XRPD characterization at the endpoints of 1, 2, 4 and 24 hrs.

XRPD patterns of solubility samples are shown in Figure 3-8 to Figure 3-11. In both media, Type A converted into hydrate Type C within 1 hour, whereas no form change was observed for Type H before being suspended for 4 hours. Type H finally converted into hydrate Type C at the endpoint of 24 hrs. Kinetic solubility results are summarized in Table 3-7, and solubility profiles are illustrated in Figure 3-7. As a result of easier conversion to hydrate Type C, Type A sample showed a lower solubility than Type H at the time points of 1~4 hrs.

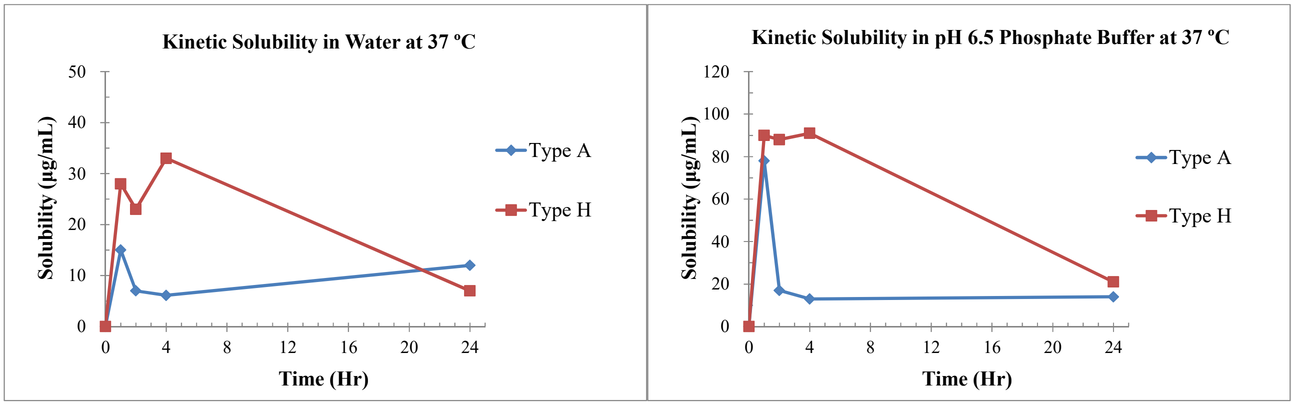

Figure 3-7 Solubility profile of Type A and H in water and pH 6.5 phosphate buffer

Table 3-7 Summary of kinetic solubility of Type A and H at 37 ºC

**Crystal Form (Batch No.)**	**Media**	**1 Hr**			**2 Hrs**			**4 Hrs**			**24 Hrs**		
		S	pH	FC	S	pH	FC	S	pH	FC	S	pH	FC
Type A (805711-28-A)	Water	15	4.4	Yes	7	4.2	Yes	6	4.4	Yes	12	6.1	Yes
Type H (805612-12-A)	Water	28	6.7	No	23	6.2	No	33	6.3	No	7	5.9	Yes
Type A (805711-28-A)	pH 6.5 phosphate buffer	78	6.5	Yes	17	6.4	Yes	13	6.5	Yes	14	6.5	Yes
Type H (805612-12-A)	pH 6.5 phosphate buffer	90	6.5	No	88	6.4	No	91	6.4	No	21	6.5	Yes

S: solubility in µg/mL, pH: final pH, FC: form change, Y: yes, N: no. LOQ equals 1.7 µg/mL.

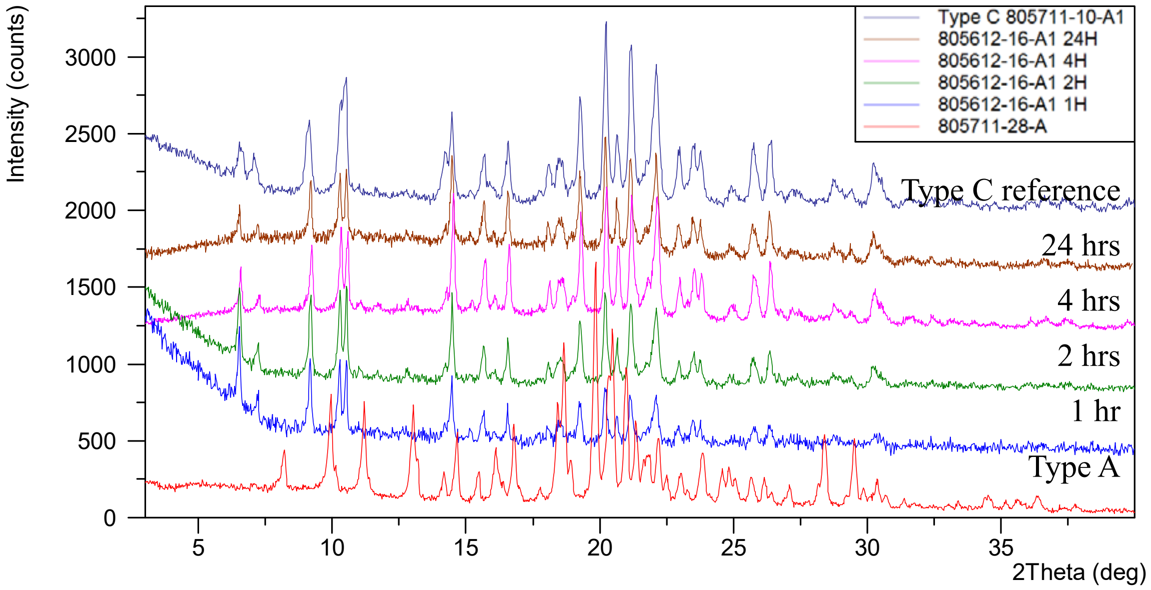

Figure 3-8 XRPD overlay of solubility samples in water starting from Type A

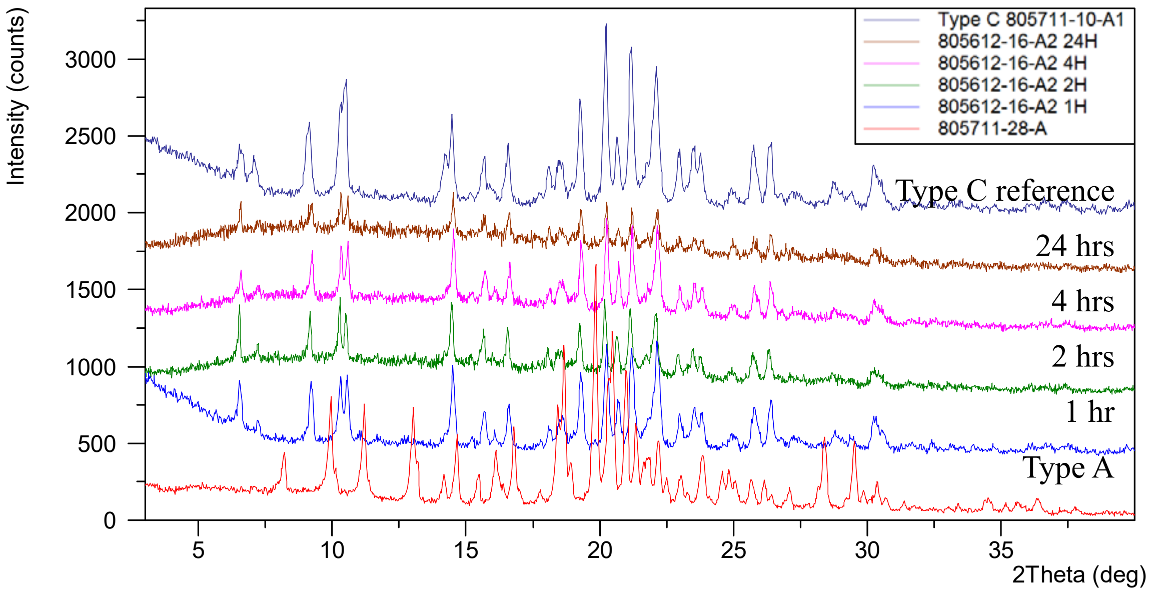

Figure 3-9 XRPD overlay of solubility samples in pH 6.5 phosphate buffer starting from Type A

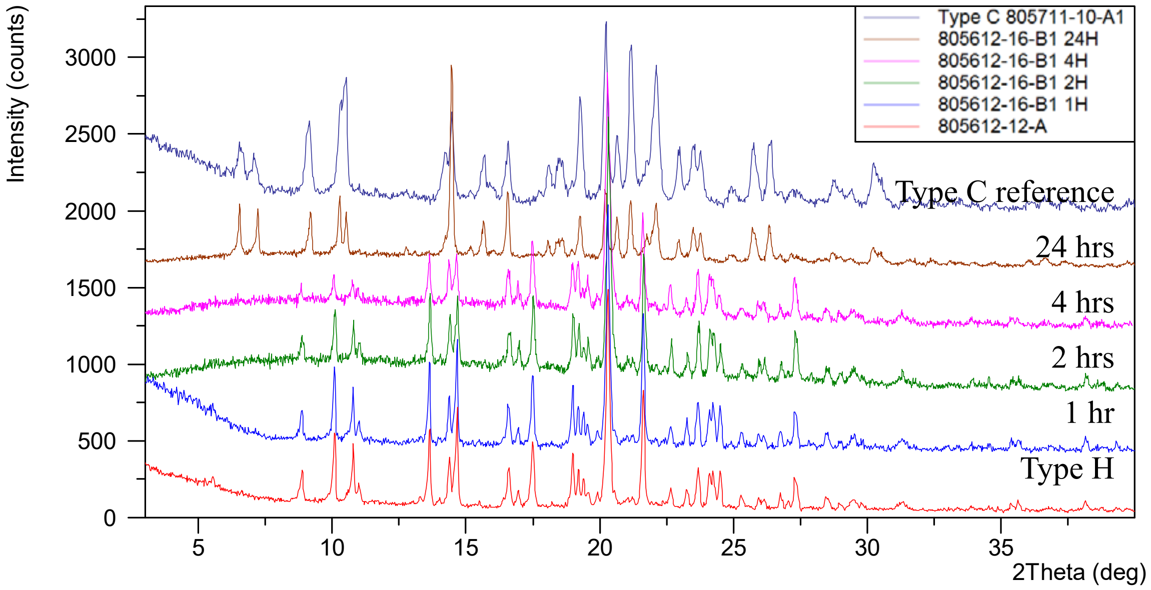

Figure 3-10 XRPD overlay of solubility samples in water starting from Type H

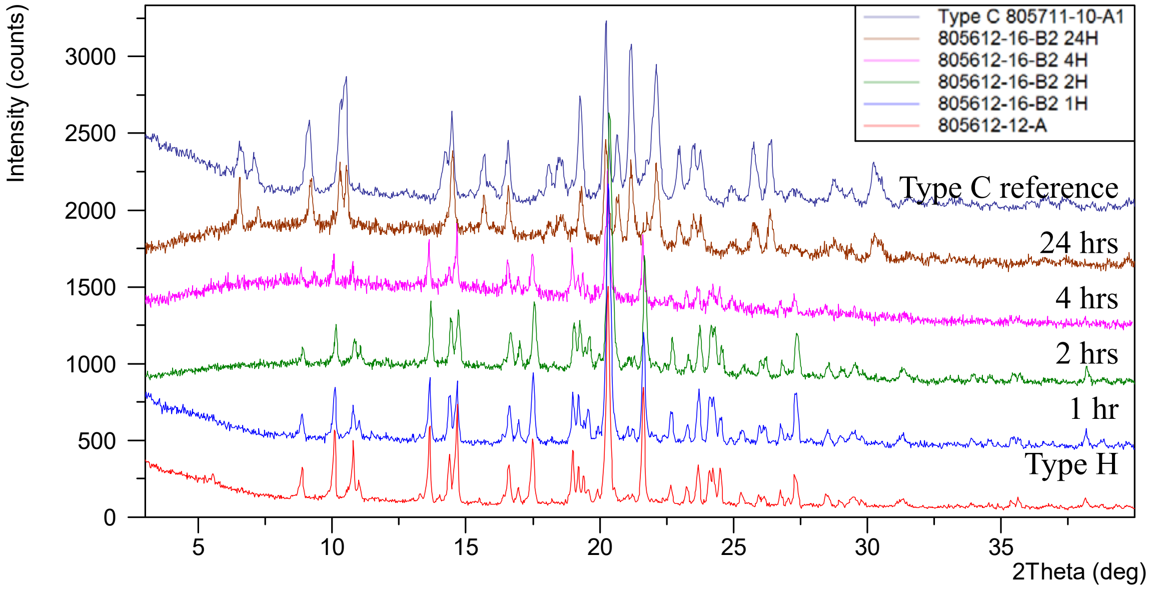

Figure 3-11 XRPD overlay of solubility samples in pH 6.5 phosphate buffer starting from Type H

3.2.2 Type A, M and N

In order to determine solubility difference of Type A, M and N in vitro, kinetic solubility was measured in water and pH 6.5 phosphate buffer at 37 ºC. Suspensions with an initial solid loading of 3.5 mg/mL were kept rolling with a speed of 25 rpm, with filtrates for HPLC and pH tests and solids for XRPD characterization at the endpoints of 1, 2, 4 and 24 hrs.

XRPD patterns of solubility samples are shown in Figure 3-13 to Figure 3-18. In both media, Type A converted into hydrate Type C within 1 hour, whereas no form change was observed for Type M and N until being suspended for 4 hours (characteristic diffraction peaks of Type C appeared for Type N in water for 4 hrs, displayed in Figure 3-17). At the endpoint of 24 hrs, Type N converted into pure hydrate Type C, whereas characteristic diffraction peaks of Type M were still observed in both media. Kinetic solubility results are summarized in Table 3-8, and solubility profiles are illustrated in Figure 3-12. As a result of easier conversion to hydrate Type C, Type A sample showed a lower solubility than Type M and N at the time points of 1~4 hrs. Meanwhile, Type M showed a smooth solubility change, due to a slow conversion rate.

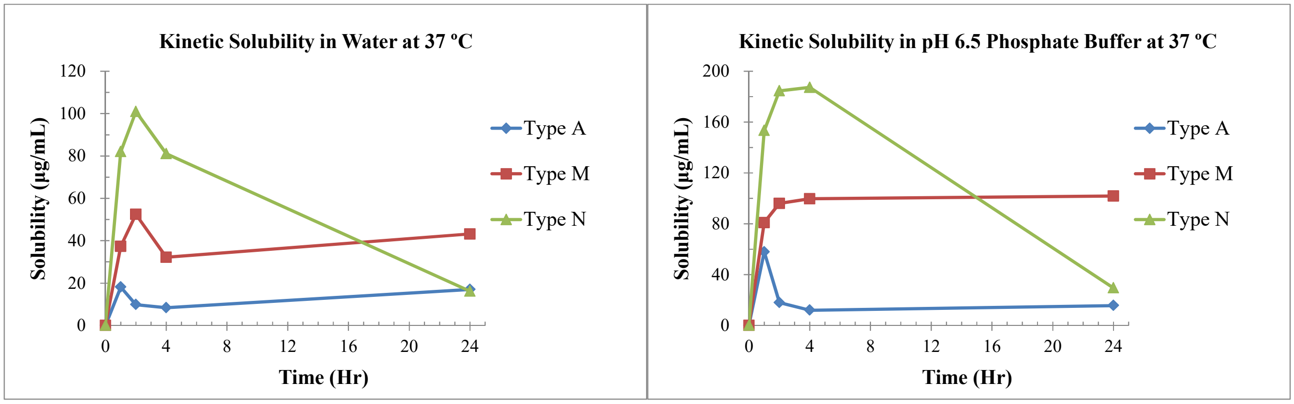

Figure 3-12 Solubility profile of Type A, M and N in water and pH 6.5 phosphate buffer

Table 3-8 Summary of kinetic solubility of Type A, M and N at 37 ºC

**Crystal Form (Batch No.)**	**Media**	**1 Hr**			**2 Hrs**			**4 Hrs**			**24 Hrs**		
		S	pH	FC	S	pH	FC	S	pH	FC	S	pH	FC
Type A (805711-28-A)	Water	18	7.2	Yes	98	7.1	Yes	83	7.2	Yes	17	7.0	Yes
Type M (803718-09-B)		37	7.3	No	52	7.1	No	32	7.3	No	43	7.0	Yes
Type N (803718-05-A4)		82	7.3	No	100	7.2	No	81	7.5	Yes	16	7.0	Yes
Type A (805711-28-A)	pH 6.5 phosphate buffer	58	6.5	Yes	18	6.5	Yes	12	6.5	Yes	16	6.5	Yes
Type M (803718-09-B)		81	6.5	No	96	6.5	No	100	6.5	No	100	6.5	Yes
Type N (803718-05-A4)		150	6.5	No	180	6.5	No	190	6.5	No	30	6.5	Yes

S: solubility in μg/mL, pH: final pH, FC: form change, Y: yes, N: no. LOQ equals 1.4 μg/mL.

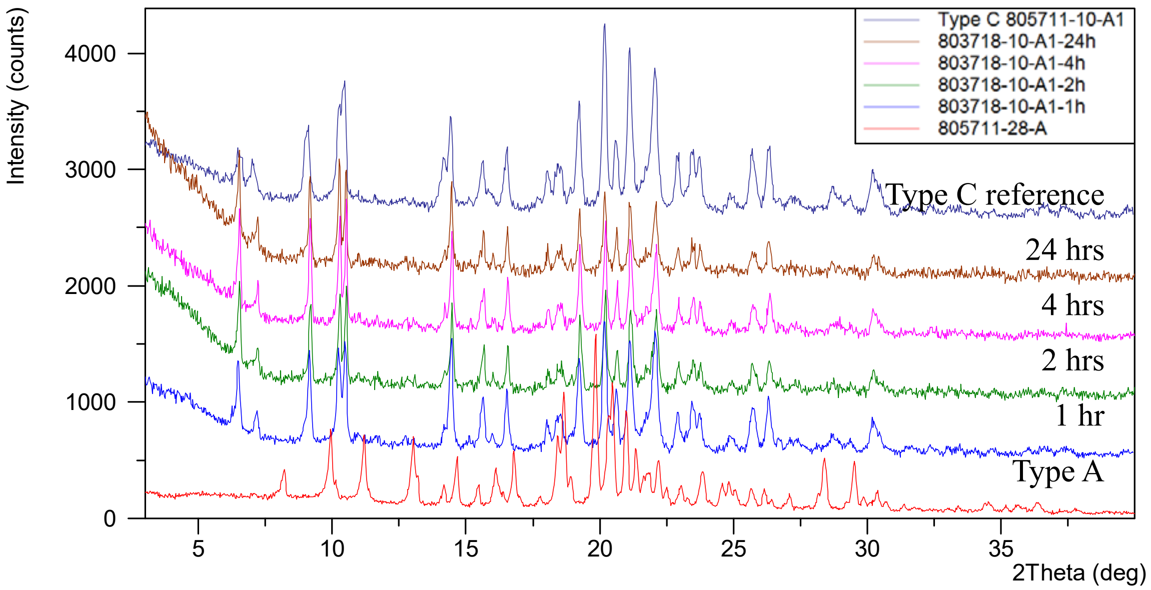

Figure 3-13 XRPD overlay of solubility samples in water starting from Type A

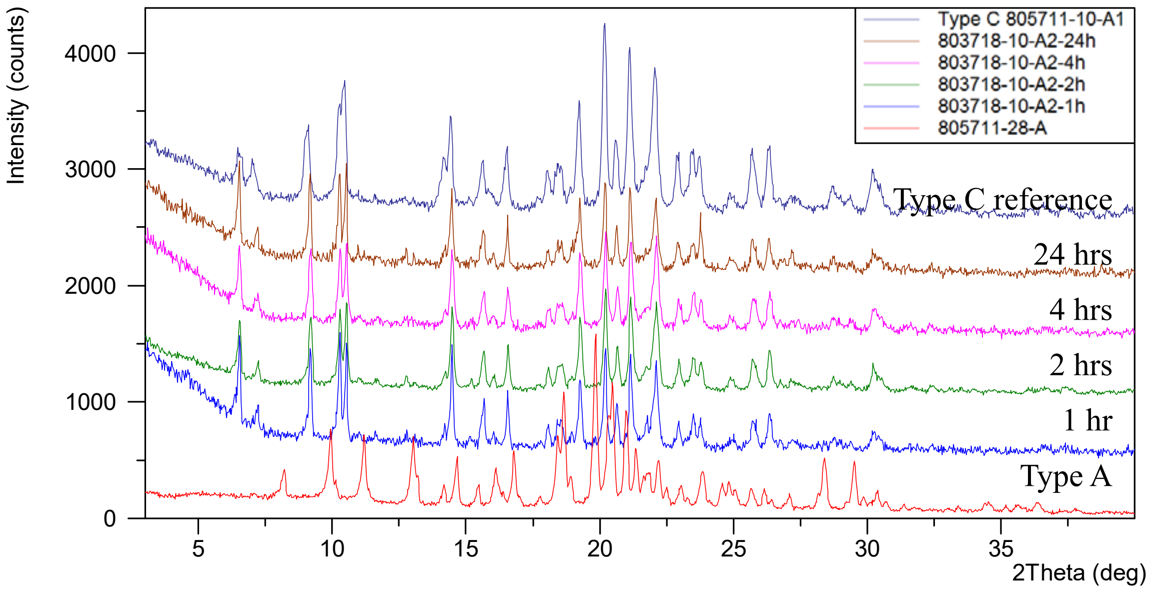

Figure 3-14 XRPD overlay of solubility samples in pH 6.5 phosphate buffer starting from Type A

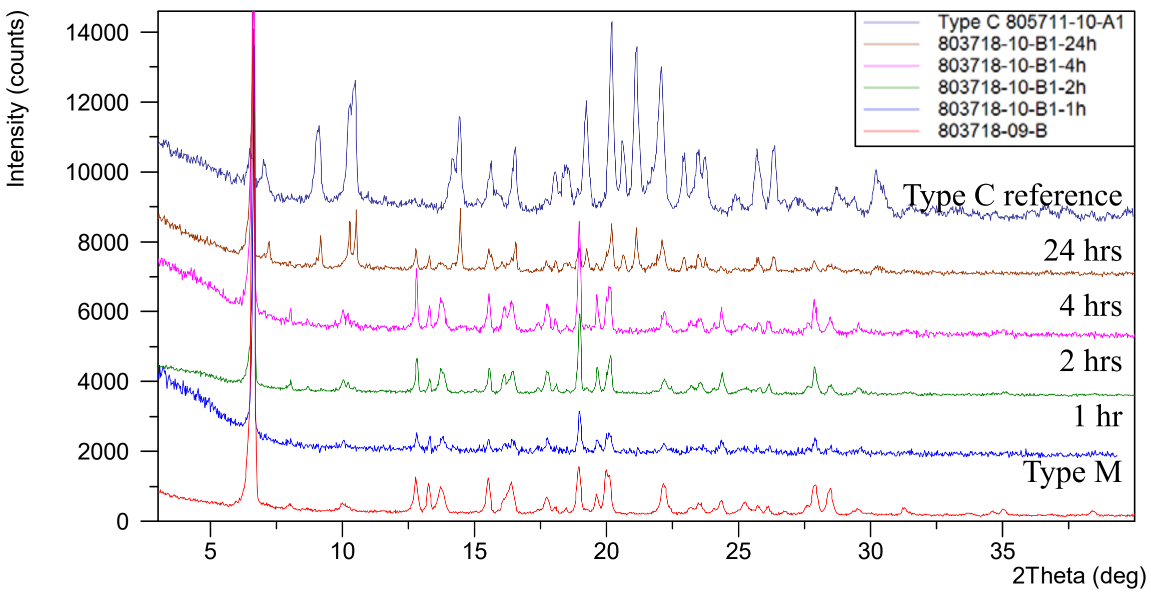

Figure 3-15 XRPD overlay of solubility samples in water starting from Type M

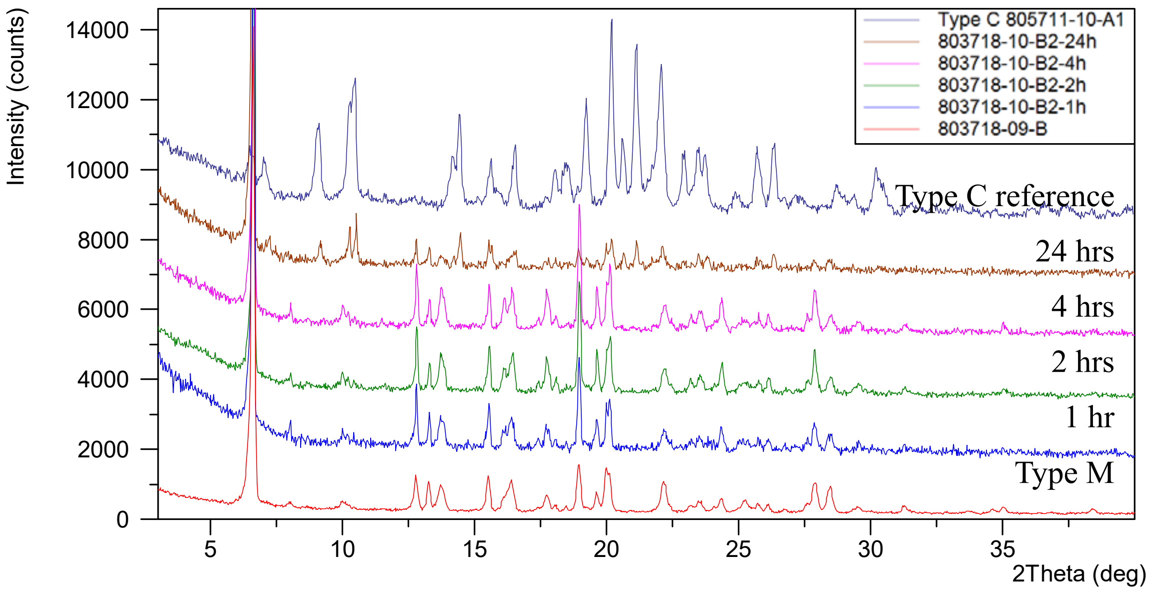

Figure 3-16 XRPD overlay of solubility samples in pH 6.5 phosphate buffer starting from Type M

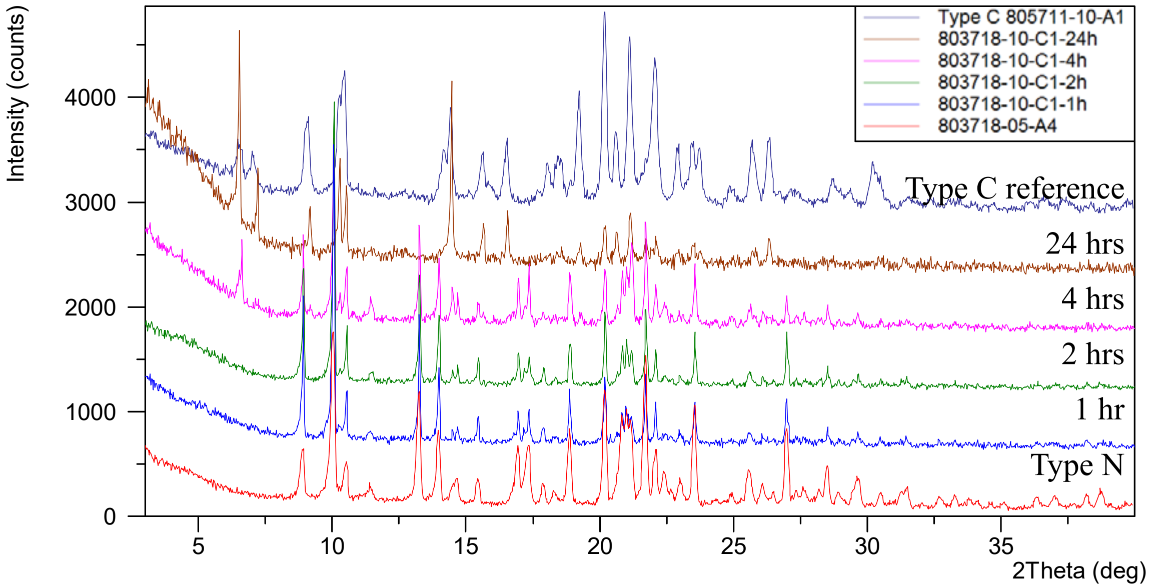

Figure 3-17 XRPD overlay of solubility samples in water starting from Type N

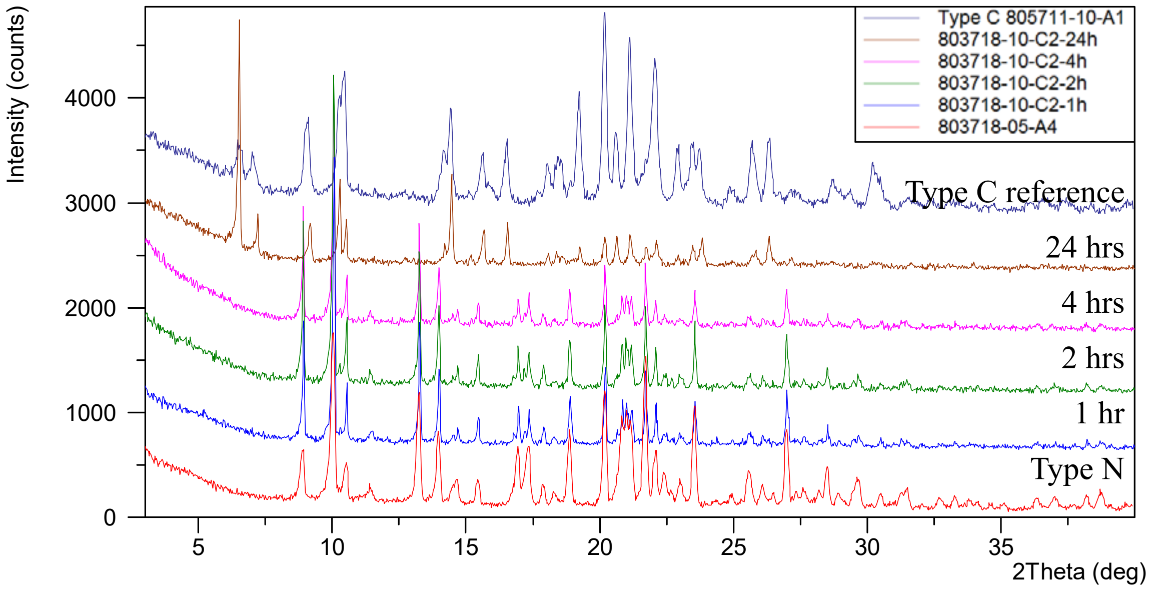

Figure 3-18 XRPD overlay of solubility samples in pH 6.5 phosphate buffer starting from Type N

4 Conclusion

Using anhydrate Type A as starting material, a polymorph screening for G02949338 was conducted through methods of solution crystallization and solid phase transition. From XRPD comparison on samples from screening and follow-up investigation, a total of 25 crystal forms, including eight reproducible anhydrates were identified so far. Thermodynamic stability relationships among the

anhydrates were investigated by competitive slurries at various temperatures, and Type M was confirmed to be the stable anhydrate above RT. Kinetic solubility of Type H, N and M was measured in water and pH 6.5 phosphate buffer at 37 ºC., using Type A as control. In both media, Type A converted into hydrate Type C within 1 hour, and showed a lower solubility. Meanwhile, Type M exhibited a smooth solubility change and the slowest conversion rate compared with other four anhydrate forms.

Based on solid-state properties and evaluation results, Type M is the most thermodynamically stable anhydrate above RT and recommended for further development.

5 Appendix

5.1 Starting Materials

Three batches of G02949338 Type A as received were used directly in the polymorph screening, with XRPD patterns shown in Figure 5-1. Detailed information is shown in Table 5-1.

Table 5-1 Sample information

Crystal Form	Batch No.	CP ID	Received Date
G02949338 Type A	B000814725	805711-05-A	07-May-2015
	G02949338.1-10	805711-15-A	15-Jun-2015
	G02949338.1-9	805711-28-A	16-Jul-2015

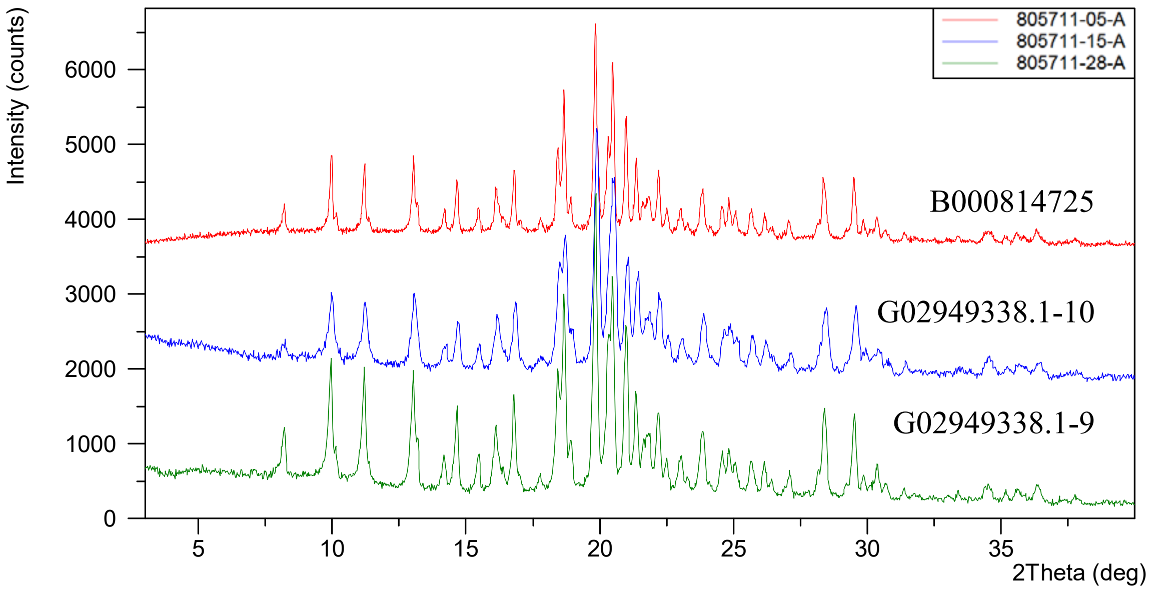

Figure 5-1 XRPD patterns of starting materials

5.2 Abbreviation for Solvents Used

The solvent abbreviations are listed in Table 5-2.

Table 5-2 Solvent abbreviation list

Abbreviation	Solvent	Abbreviation	Solvent
MeOH	Methanol	THF	Tetrahydrofuran
EtOH	Ethanol	2-MeTHF	2-Methyltetrahydrofuran
IPA	Isopropyl alcohol	DCM	Dichloromethane
MIBK	4-Methyl-2-pentanone	ACN	Acetonitrile
EtOAc	Ethyl acetate	DMSO	Dimethylsulfoxide
IPAc	Isopropyl acetate	IBAc	Isobutyl acetate
MTBE	Methyl tert-butyl ether	DMC	Dimethyl carbonate

5.3 Instruments and Methods

5.3.1 XRPD

For XRPD analysis, PANalytical and Bruker X-ray powder diffractometers were used. The XRPD parameters used are listed in Table 5-3.

Table 5-3 Parameters for XRPD test

Parameters	PANalytical (Reflection Mode)	PANalytical (Reflection Mode)	Bruker (Reflection Mode)
Model	Empyrean	X' Pert[3]	D2 PHASER
X-Ray wavelength	Cu, kα,<br>Kα1 (Å): 1.540598,<br>Kα2 (Å): 1.544426<br>Kα2/Kα1 intensity ratio: 0.50	Cu, kα,<br>Kα1 (Å): 1.540598,<br>Kα2 (Å): 1.544426<br>Kα2/Kα1 intensity ratio: 0.50	Cu, kα,<br>Kα1 (Å): 1.5406,<br>Kα2 (Å): 1.54439<br>Kα2/Kα1 intensity ratio: 0.50
X-Ray tube setting	45 kV, 40 mA	45 kV, 40 mA	30 kV, 10 mA
Divergence slit	Automatic	1/8°	0.6mm
Scan mode	Continuous	Continuous	Continuous
Scan range (°2TH)	3°-40°	3°-40°	3°-40°
Scan step time [s]	17.8	46.7	0.1
Step size (°2TH)	0.0167	0.0263	0.0201

Parameters	PANalytical (Reflection Mode)	PANalytical (Reflection Mode)	Bruker (Reflection Mode)
Test Time (s)	5 min 30 s	5 min 04 s	3 min 27 s

5.3.2 TGA and DSC

TGA data was collected using a TA Q500/Q5000 TGA from TA Instruments. DSC was performed using a TA Q200/Q2000 DSC from TA Instruments. Detailed parameters used are listed in Table 5-4.

Table 5-4 Parameters for TGA and DSC test

Parameters	TGA	DSC
Method	Ramp	Ramp
Sample pan	Platinum, open	Aluminum, crimped
Temperature	RT-300 ºC	RT-250 ºC
Heating rate	10 ºC/min	10 ºC/min
Purge gas	N_2	N_2

5.3.3 ^{1}H NMR

^{1}H NMR data was collected on Bruker 400M NMR Spectrometer using DMSO-d_6.

5.3.4 DVS

Dynamic Vapor Sorption (DVS) was measured via a SMS (Surface Measurement Systems) DVS Intrinsic. Parameters for DVS test were listed in Table 5-5.

Table 5-5 Parameters for DVS test

Parameters	Values
Temperature	25 ºC
Sample size	10-20 mg
Gas and flow rate	N_2, 200 mL/min
dm/dt	0.002%/min
Min. dm/dt stability duration	10 min
Max. equilibrium time	180 min
RH range	0%RH-95%RH-0%RH
RH step size	10% (0%RH-90%RH-0%RH)<br>5% (90%RH-95%RH and 95%RH-90%RH)

5.3.5 HPLC

Agilent 1100 HPLC was utilized and detailed chromatographic conditions for solubility measurement are listed in Table 5-6.

Table 5-6 Chromatographic conditions and parameters for solubility measurement

HPLC	Agilent 1100 with VWD detector	
Column	Waters Xbridge C18, 150 × 4.6 mm, 5 µm	
Mobile phase	A: 0.1% TFA in H_2O	
	B: 0.1% TFA in acetonitrile	
Gradient table	Time (min)	%B
	0.0	10
	6.0	90
	8.0	90
	8.1	10
	10.0	10
Run time	10 min	
Post time	0.0 min	
Flow rate	1.0 mL/min	
Injection volume	10 µL	
Detector wavelength	UV at 220 nm, reference 400 nm	
Column temperature	40 ºC	

HPLC	Agilent 1100 with VWD detector
Sampler temperature	RT
Diluent	MeOH

5.4 Characterization of Solvates

5.4.1 Type I

Type I can be generated from a variety of solvent systems (Figure 5-2) and slight difference is highlighted in XRPD. Type I sample (805711-18-A1) was obtained via adding n-heptane into acetone solution. As per TGA and DSC data shown in Figure 5-3, a weight loss of 2.2% up to 180 ºC was observed in TGA and DSC result showed multiple endotherms. ^{1}H NMR in Figure 5-5 showed no significant level of acetone, indicating the non-solvated structure. As compared in Figure 5-4, no form change was observed after Type I sample (805711-18-A1) was heated to 100 ºC, cooled to RT and re-exposed to air. Therefore, Type I sample (805711-18-A1) is deemed to be a hydrate and bound water can be absorbed back readily at ambient conditions.

Type I sample (803718-22-B) was obtained via cooling crystallization in MEK. As per TGA and DSC data shown in Figure 5-6, a weight loss of 6.0% up to 120 ºC was observed in TGA and DSC result showed a broad endotherm at 68.6 ºC before melting at 125.6 ºC (peak temperature). As analyzed by GC, 5.4% of MEK was detected and consistent with the TGA weight loss, suggesting that Type I sample (803718-22-B) is a MEK solvate.

From the above data, Type I is deemed to be an isomorphic form, which can interact with water or solvent in the crystal lattice.

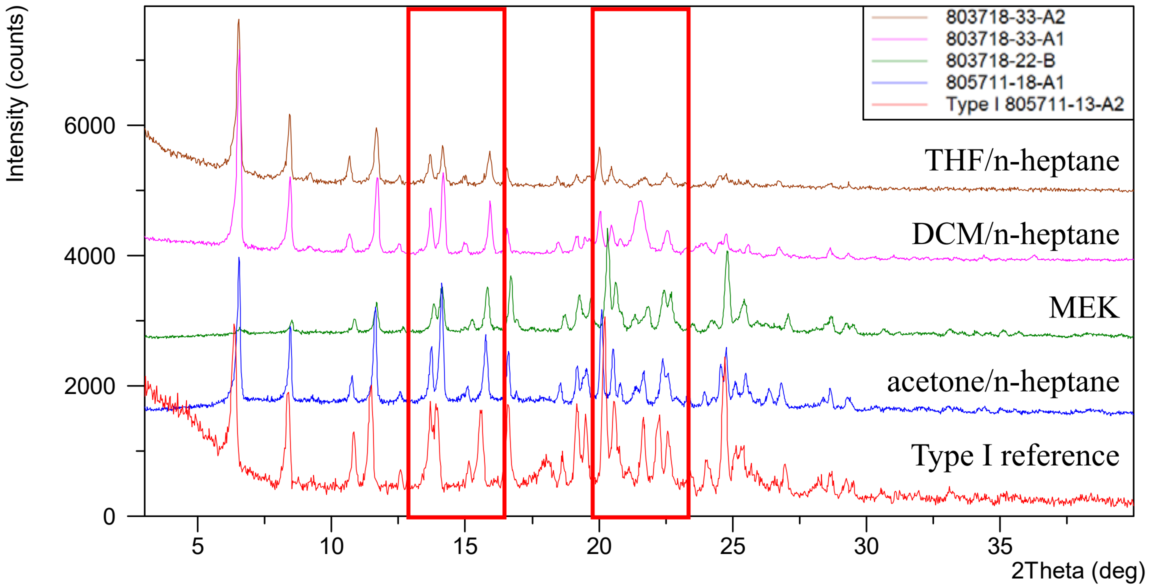

Figure 5-2 XRPD patterns of Type I batches

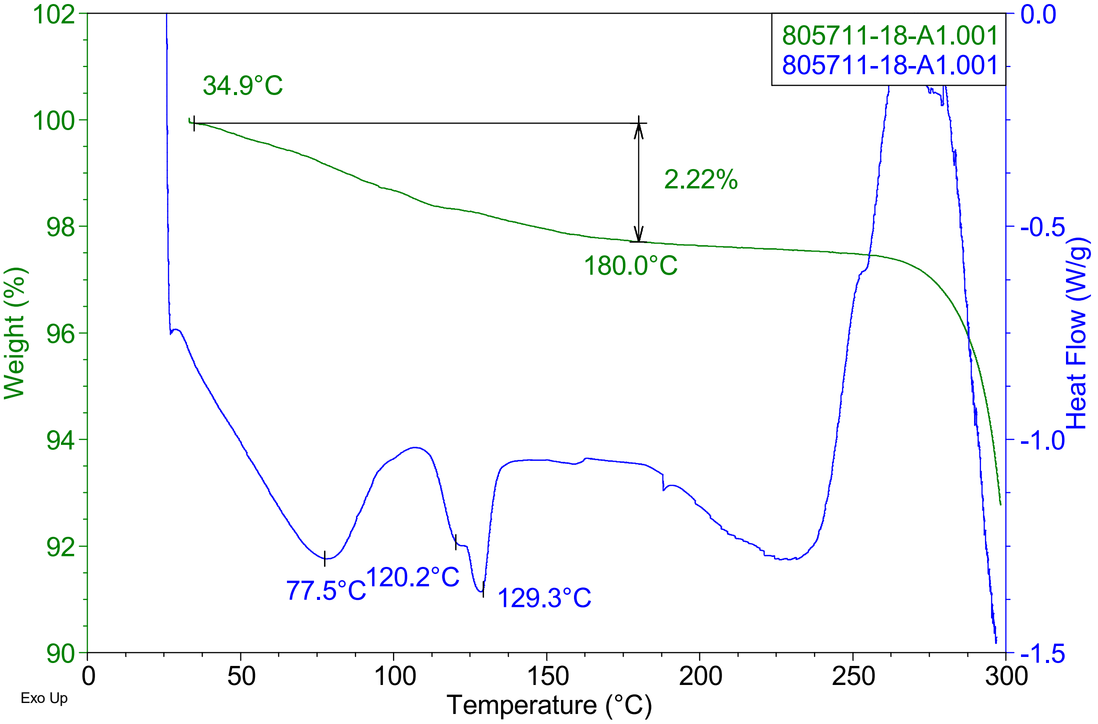

Figure 5-3 TGA/DSC curves of Type I (805711-18-A1)

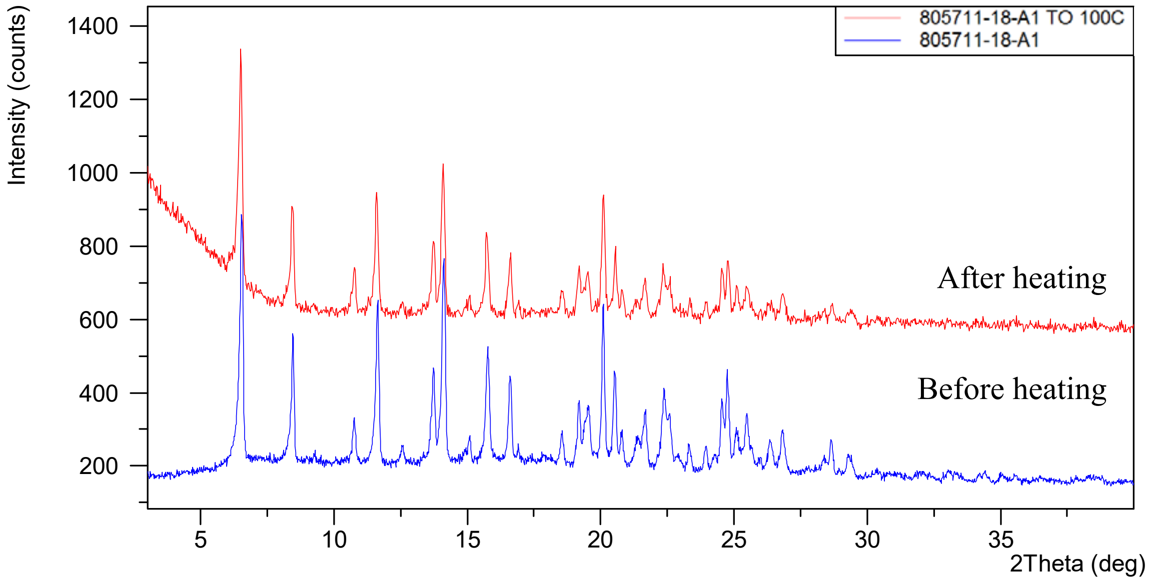

Figure 5-4 XRPD overlay of Type I (805711-18-A1) before and after heating

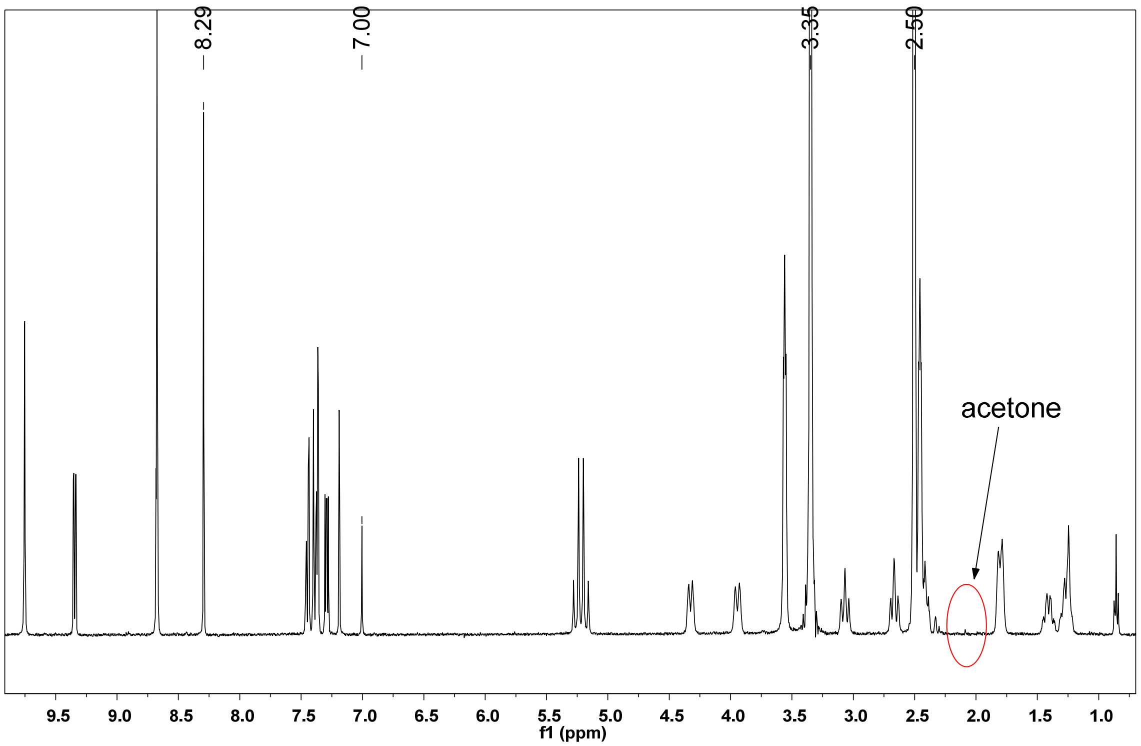

Figure 5-5 ^{1}H NMR spectrum of Type I (805711-18-A1)

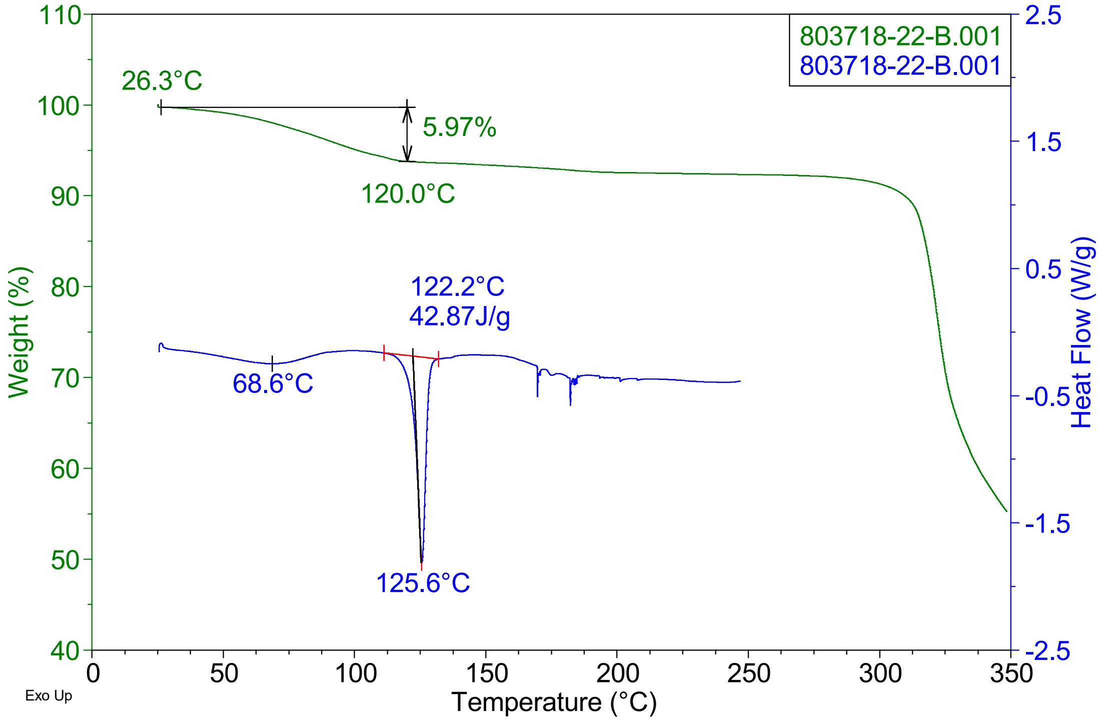

Figure 5-6 TGA/DSC curves of Type I (803718-22-B)

5.4.2 Type B

Type B can be obtained via EtOH-mediated crystallization. Type B sample (805711-15-A3) was obtained via interaction of Type A solids (805711-15-A) with EtOH vapor. Its XRPD pattern is shown in Figure 5-7. TGA and DSC data are shown Figure 5-8. A two-step weight loss of 7.9% was observed up to 160 ºC in TGA and DSC result showed overlapped endotherms before melting at 165.9 ºC (onset temperature).

As analyzed by ^{1}H NMR in Figure 5-9, a molar ratio of EtOH to freebase was determined to be 0.91 (6.3%). As shown in Figure 5-10 , Type B converted into Type A after Type B sample (805711-15-A3) was heated to 140 ºC and cooled to RT under protection of nitrogen. From the above data, Type B is deemed to be an EtOH solvate.

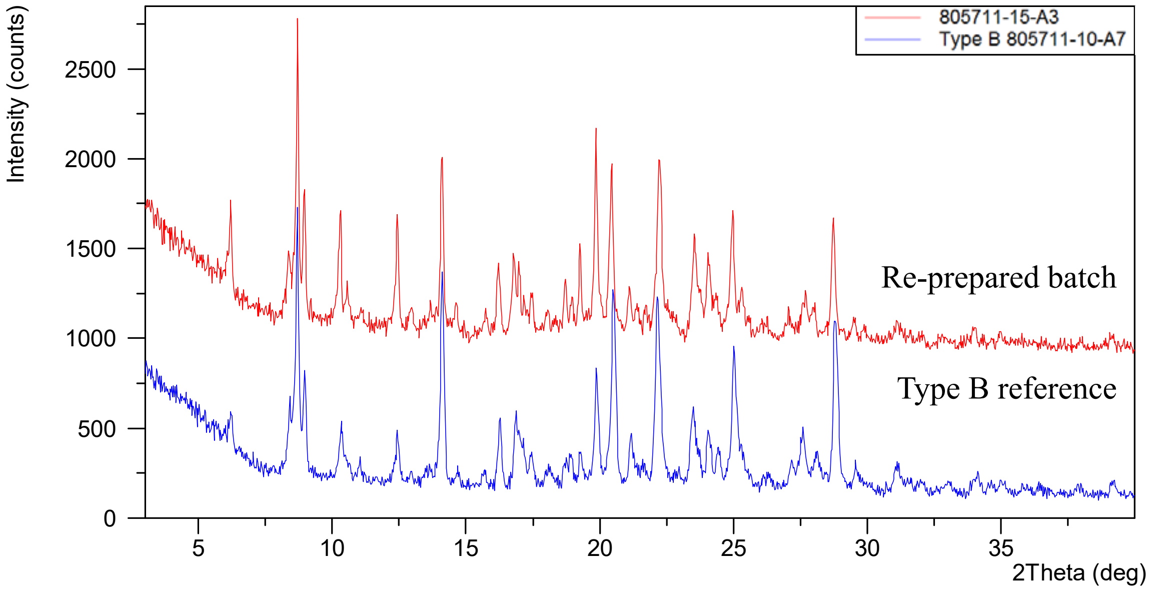

Figure 5-7 XRPD patterns of Type B (805711-10-A7) and (805711-15-A3)

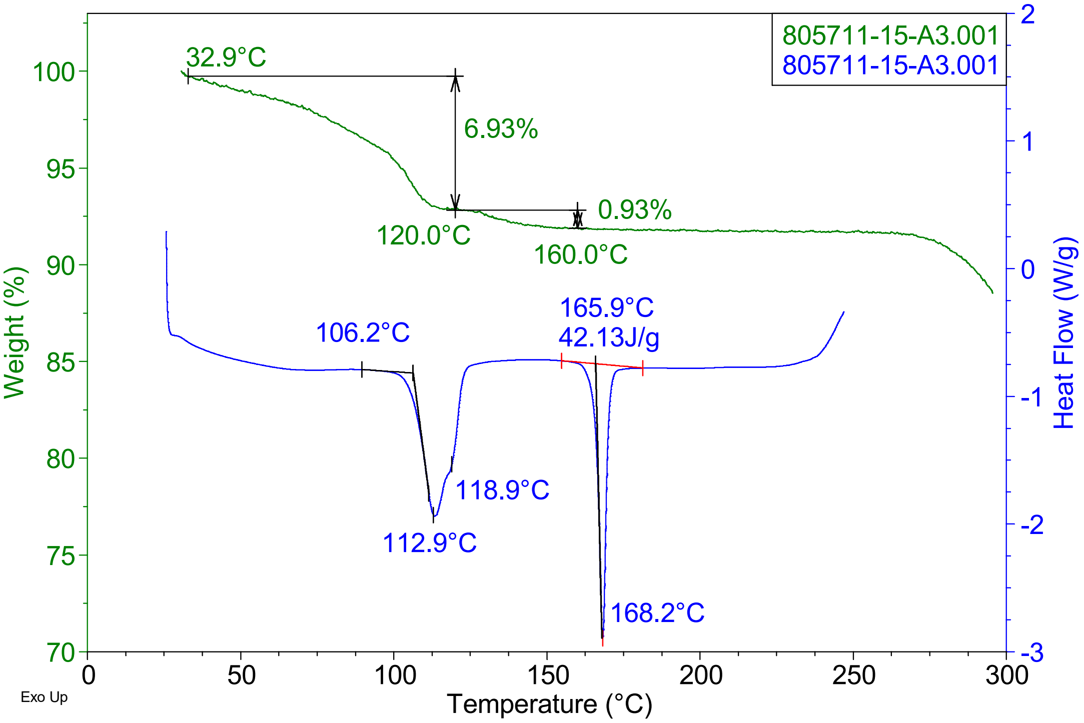

Figure 5-8 TGA/DSC curves of Type B (805711-15-A3)

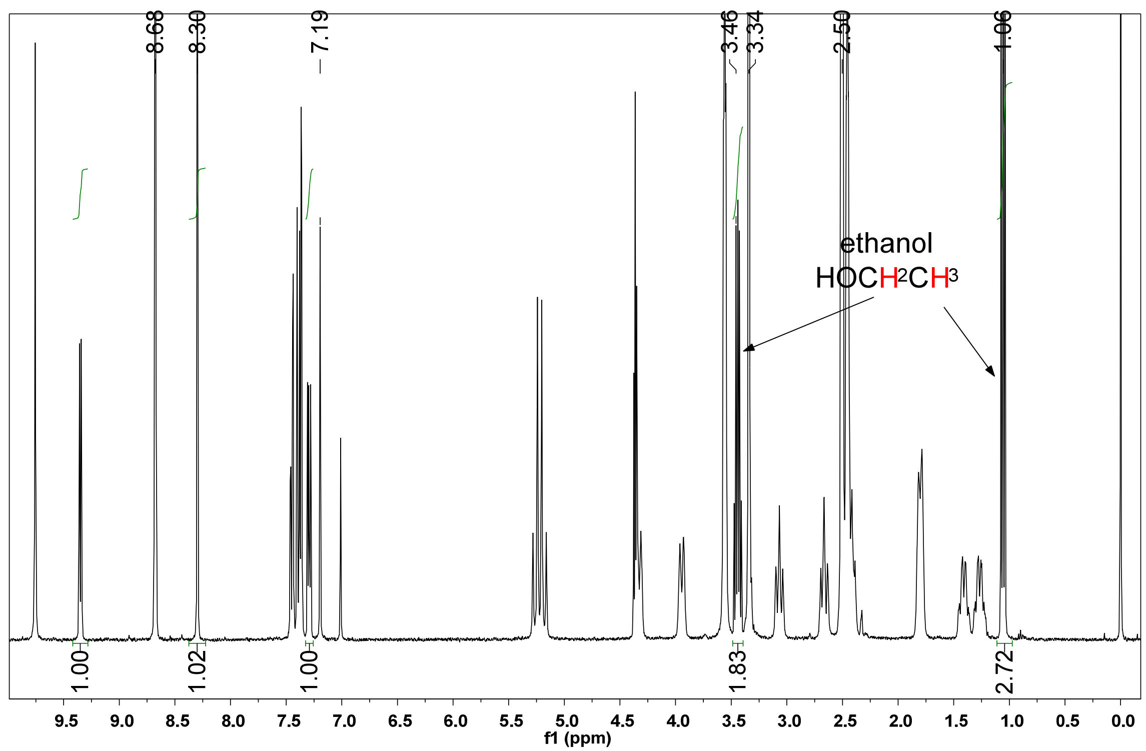

Figure 5-9 ^{1}H NMR spectrum of Type B (805711-15-A3)

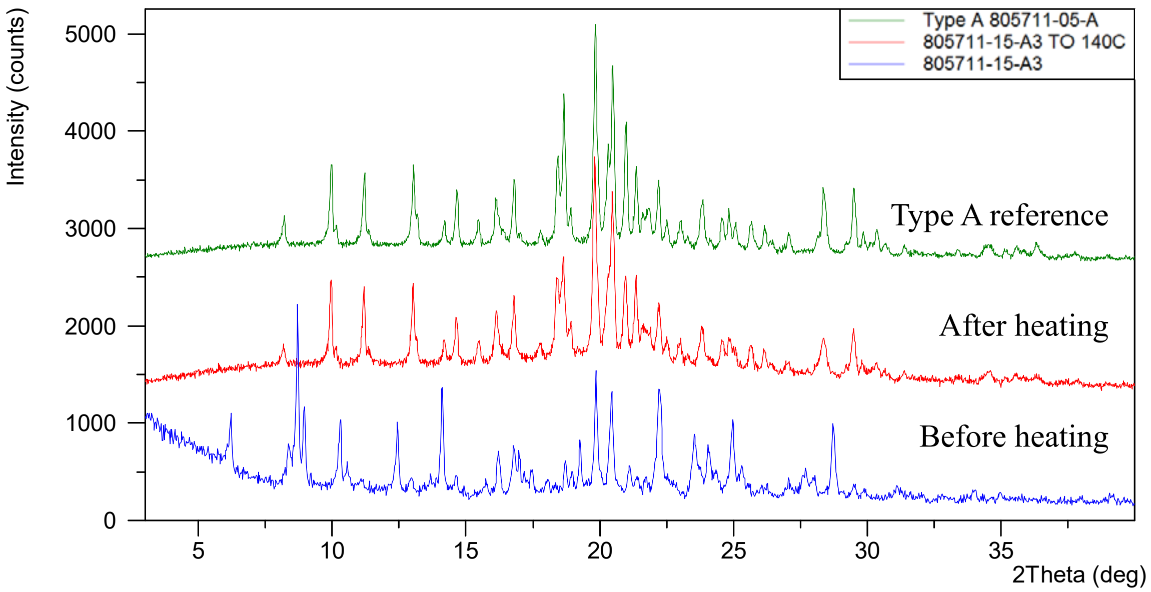

Figure 5-10 XRPD overlay of Type B (805711-15-A3) before and after heating

5.4.3 Type E

Type E can be obtained via IPA-mediated crystallization. Type E sample (805711-15-A2) was obtained via slurry of Type A solids (805711-15-A) in IPA at RT. Its XRPD pattern is shown in

Figure 5-11. TGA and DSC data are shown Figure 5-12. A two-step weight loss of 9.4% was observed up to 160 ºC in TGA and DSC result showed an endotherm at 103.1 ºC before melting at 166.2 ºC (onset temperature).

As analyzed by ^{1}H NMR result shown in Figure 5-13, a molar ratio of IPA to freebase was determined to be 0.82 (6.8%). As shown in Figure 5-14, Type E converted into Type A after Type E sample (805711-15-A2) was heated to 140 ºC and cooled to RT under protection of nitrogen. From the above data, Type E is deemed to be an IPA solvate.

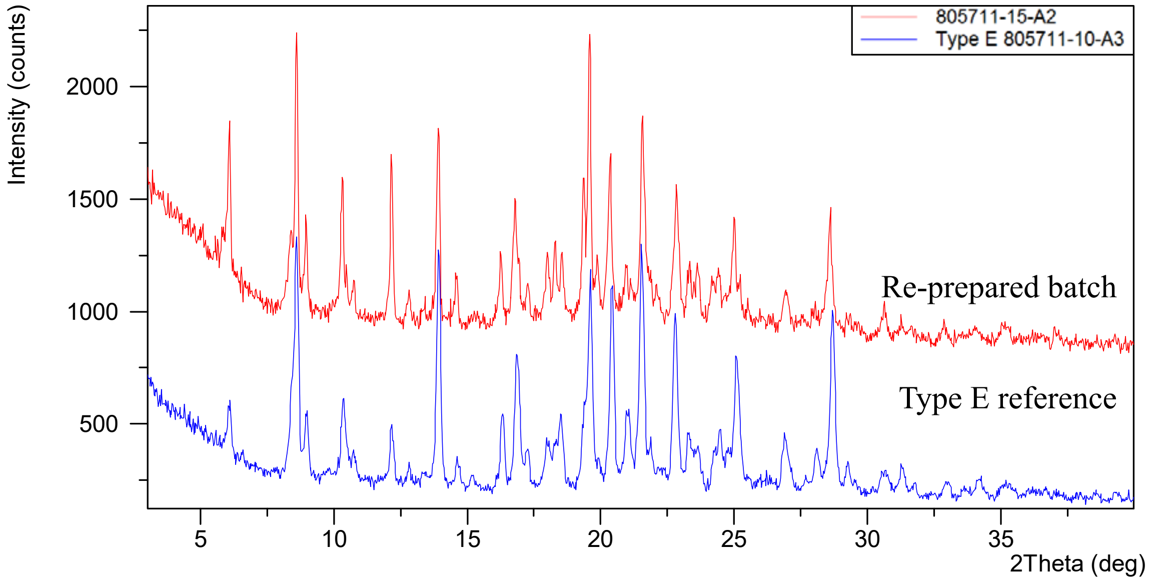

Figure 5-11 XRPD patterns of Type E (805711-10-A3) and (805711-15-A2)

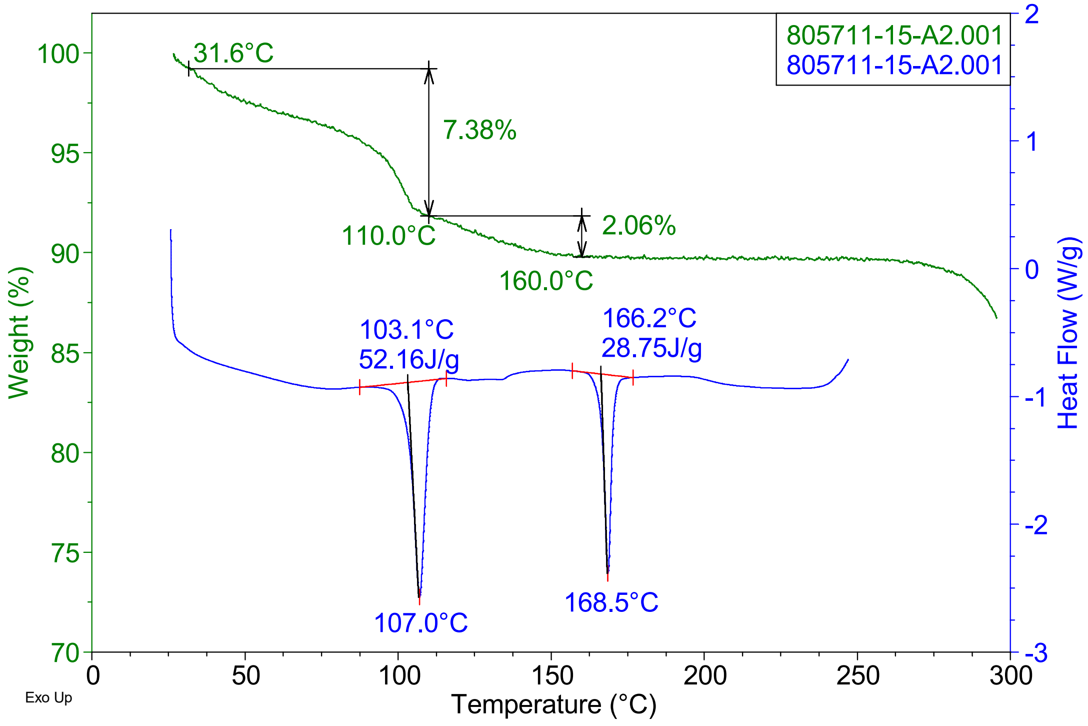

Figure 5-12 TGA/DSC curves of Type E (805711-15-A2)

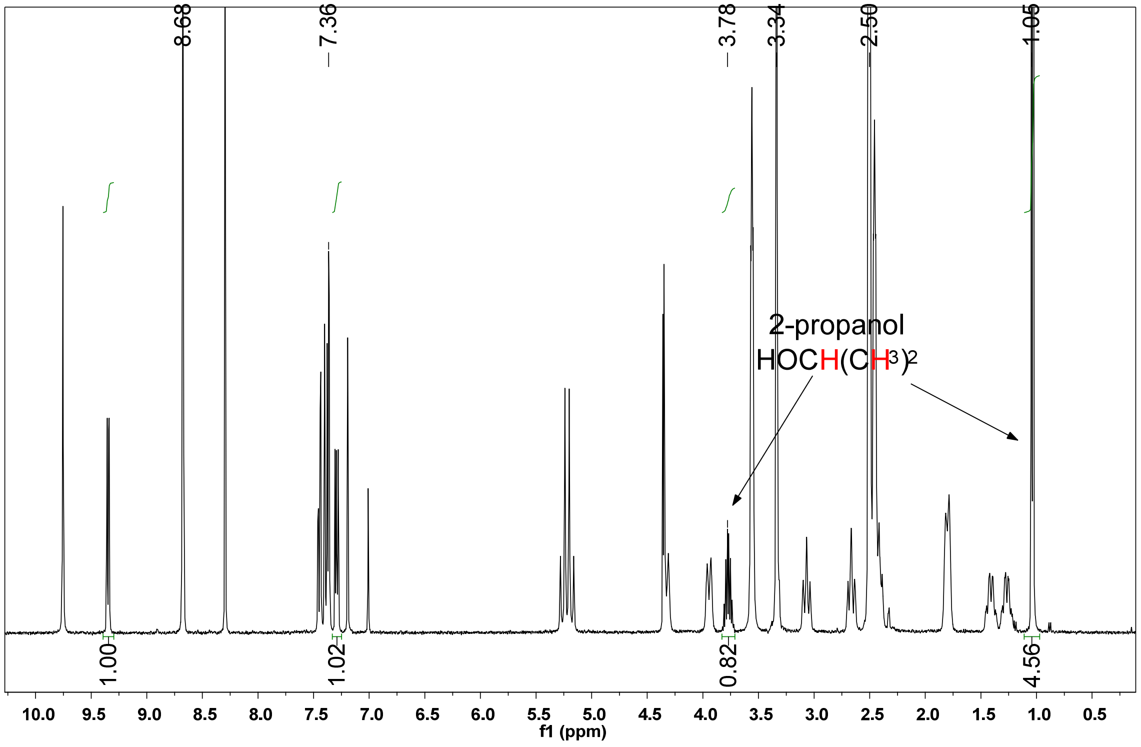

Figure 5-13 ^{1}H NMR spectrum of Type E (805711-15-A2)

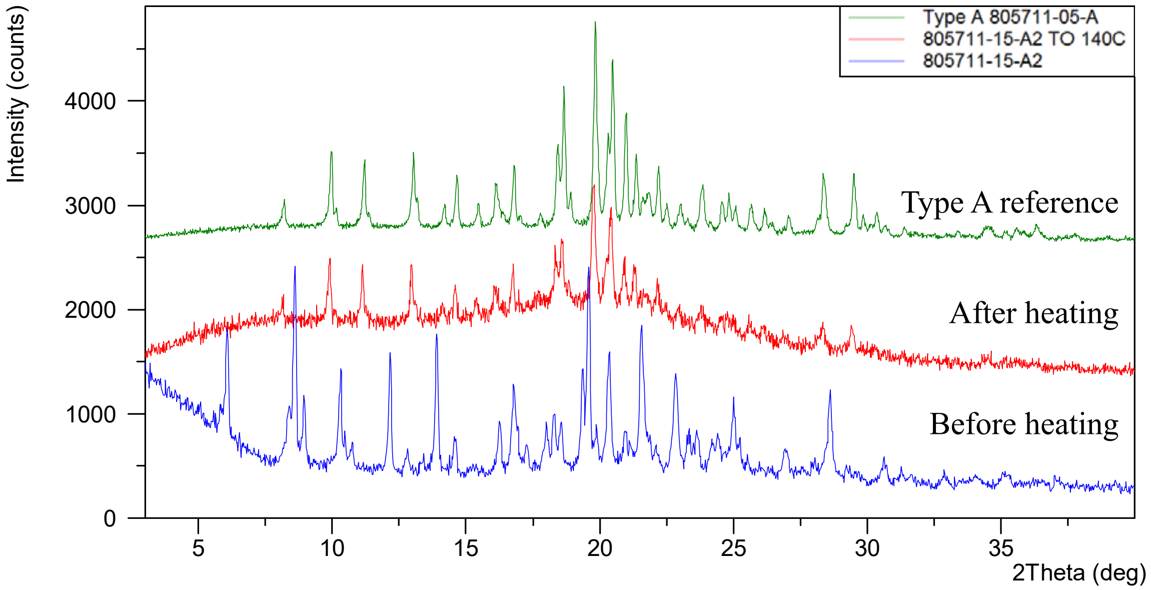

Figure 5-14 XRPD overlay of Type E (805711-15-A2) before and after heating

5.4.4 Type J

Type J sample (805711-15-A4) was obtained via interaction of Type A solids with DMSO vapor. Its XRPD pattern is shown in Figure 5-15. As per TGA and DSC data shown Figure 5-16. A two-step weight loss of 12.4% was observed up to 230 ºC in TGA and DSC result showed an endotherm at 108.5 ºC (onset temperature).

As analyzed by ^{1}H NMR result shown in Figure 5-17, a molar ratio of DMSO to freebase was determined to be 1.11 (12.2%), which was consistent with TGA weight loss. As shown in Figure 5-18, Type J converted into Type A after Type J sample (805711-15-A2) was heated to 130 ºC and cooled to RT under protection of nitrogen. From the above data, Type E is deemed to be a DMSO solvate.

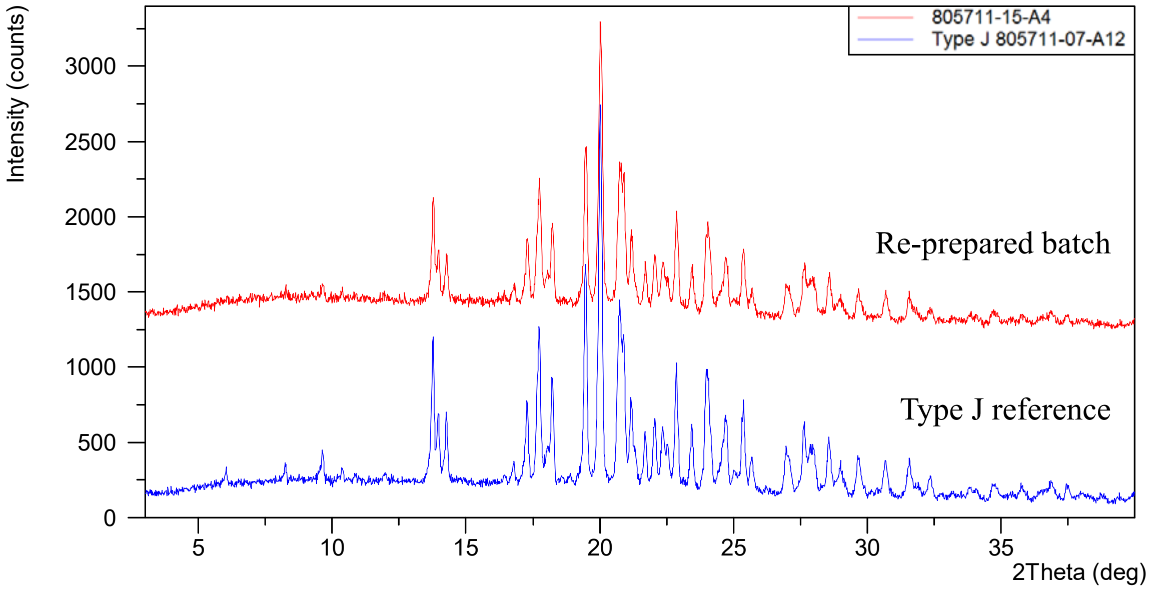

Figure 5-15 XRPD patterns of Type J (805711-07-A12) and (805711-15-A4)

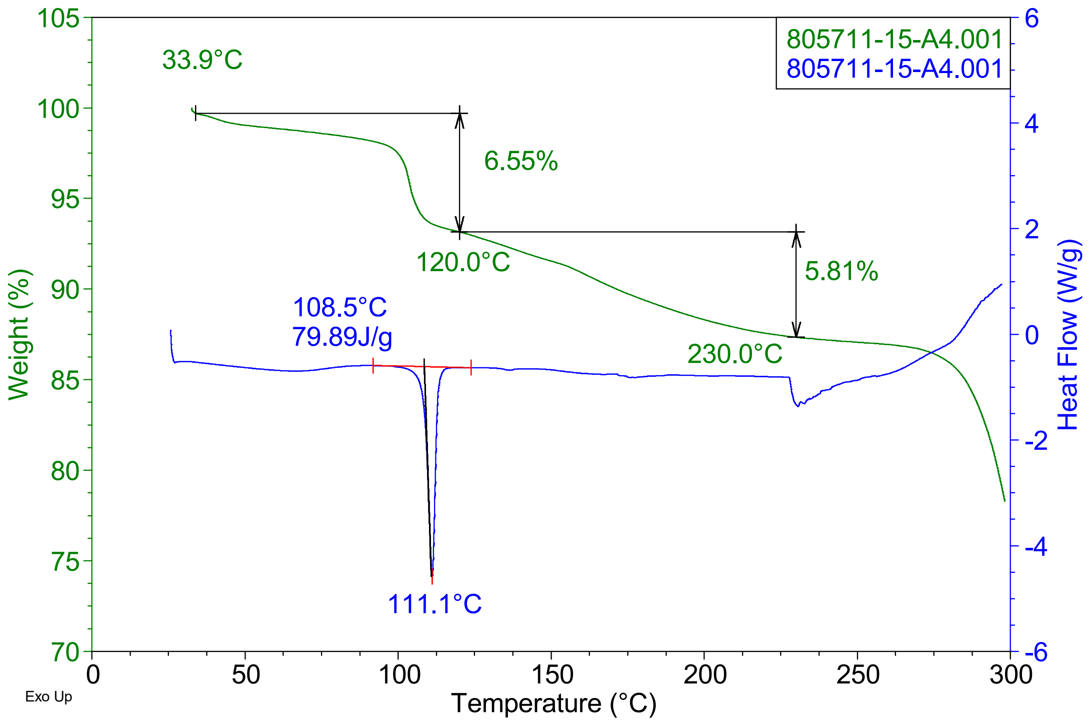

Figure 5-16 TGA/DSC curves of Type J (805711-15-A4)

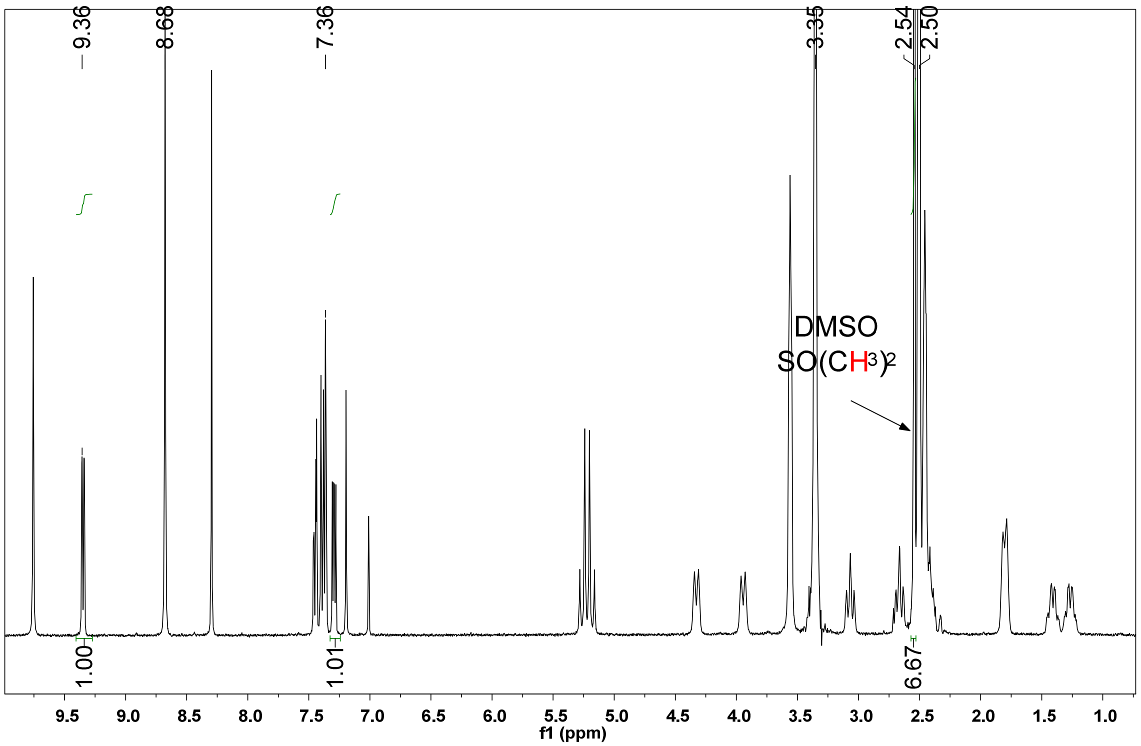

Figure 5-17 ^{1}H NMR spectrum of Type J (805711-15-A4)

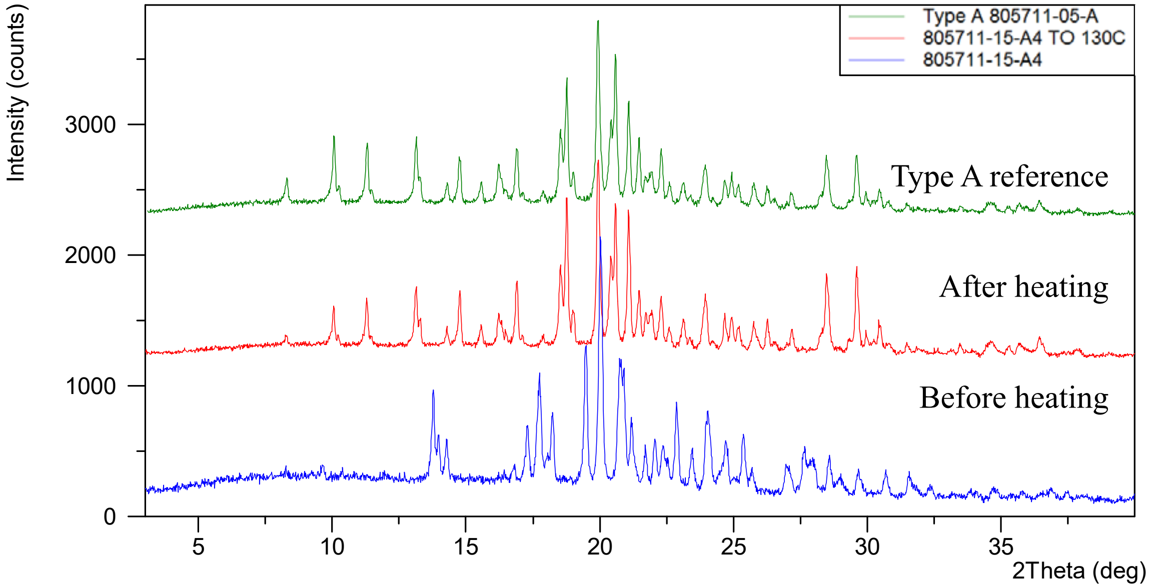

Figure 5-18 XRPD overlay of Type J (805711-15-A4) before and after heating

5.4.5 Type Q

Type Q sample (803718-29-A3) was obtained via slurry of Type M in DMC at RT. Its XRPD pattern is shown in Figure 5-19. TGA and DSC data are displayed in Figure 5-20. A weight loss of 7.0% up

to 200 ºC in TGA and DSC result showed an endotherm at 116.8 ºC (onset temperature), associated with desolvation.

As analyzed by ^{1}H NMR result shown in Figure 5-21, a molar ratio of DMC to API was determined to be 0.45 (6.1%), approximately consistent with TGA weight loss. After being heated to 125 ºC, Type Q converted into amorphous, as shown in Figure 5-22. From the above data, Type Q is speculated to be a DMC solvate.

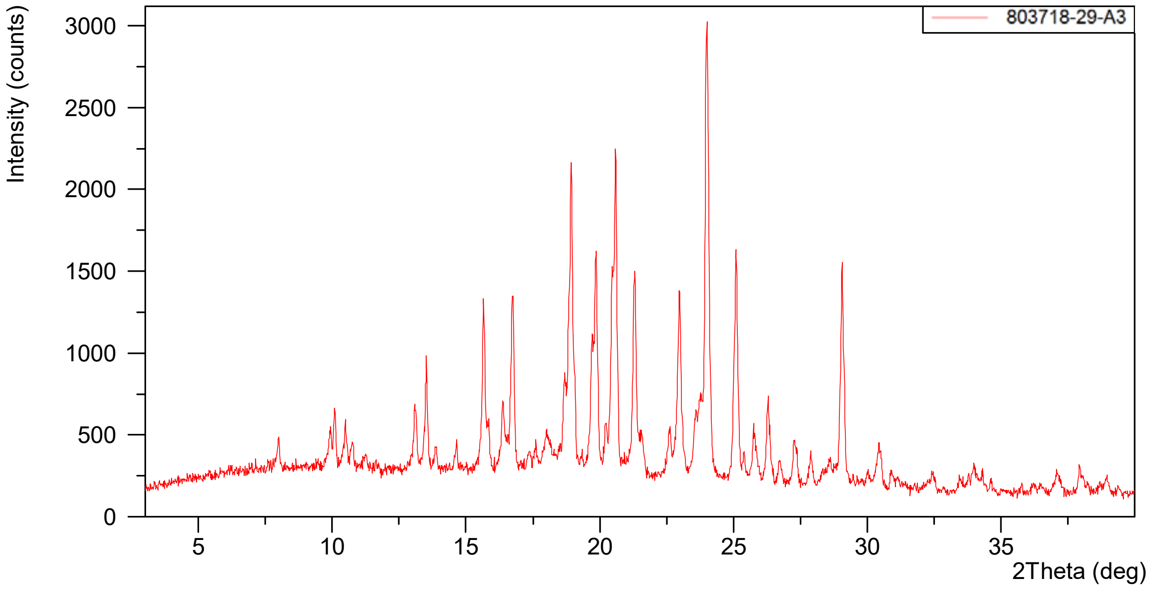

Figure 5-19 XRPD pattern of Type Q (803718-29-A3)

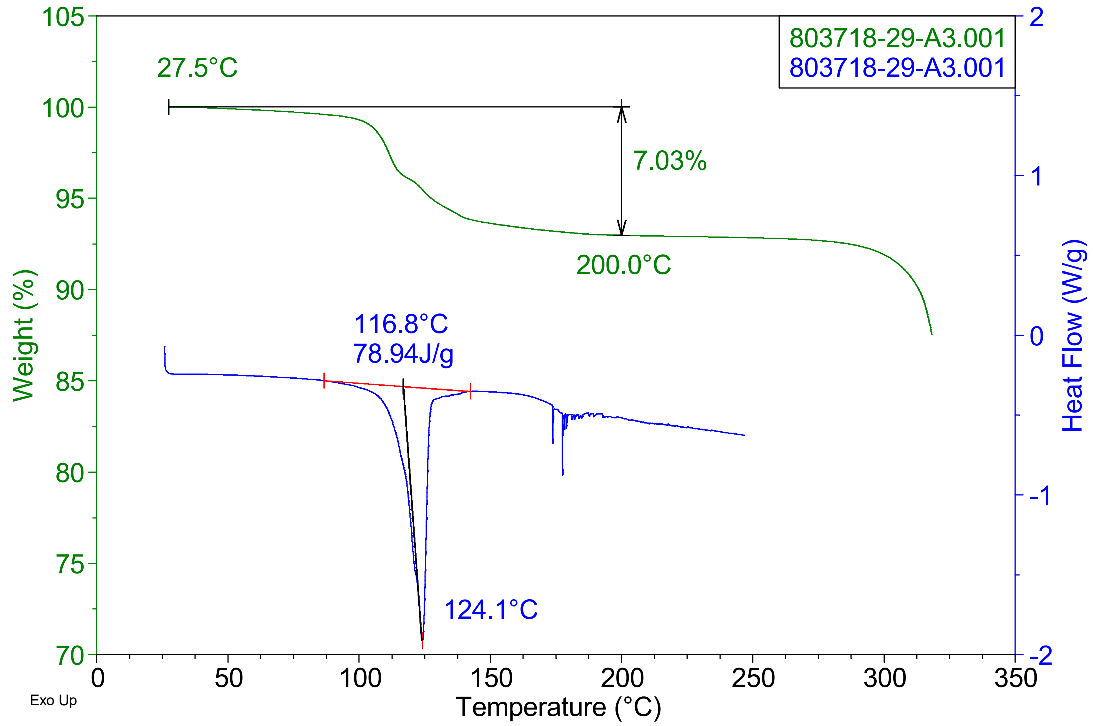

Figure 5-20 TGA/DSC curves of Type Q (803718-29-A3)

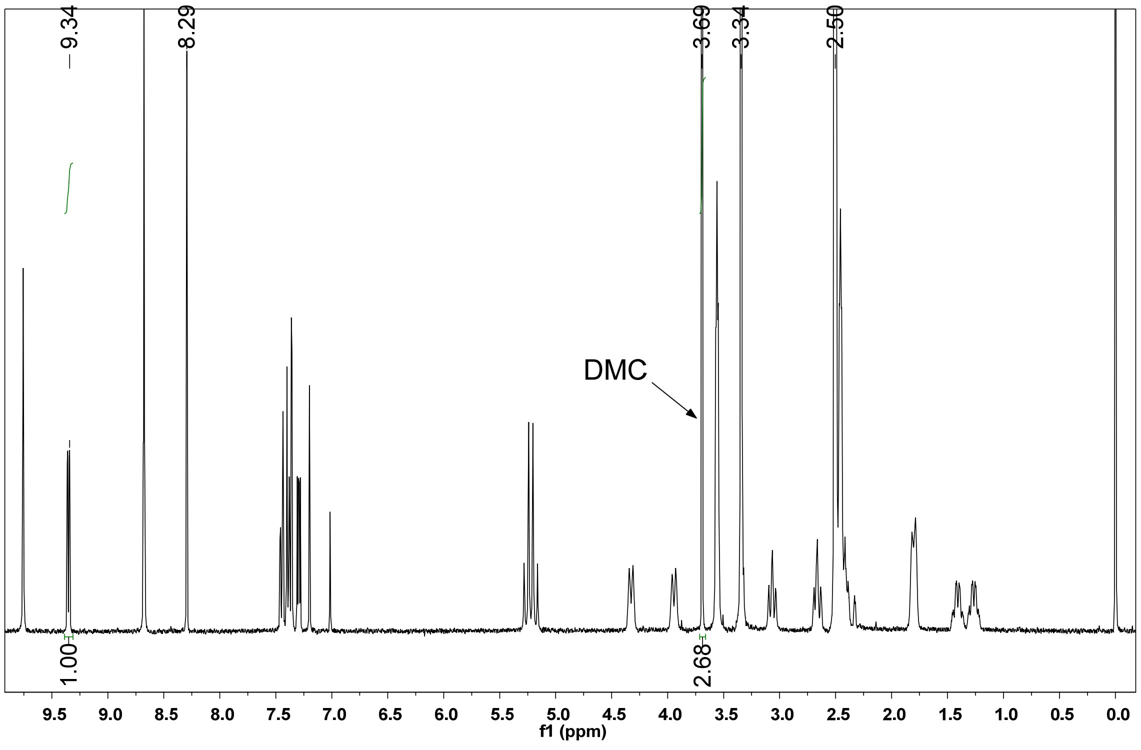

Figure 5-21 ^{1}H NMR spectrum of Type Q (803718-29-A3)

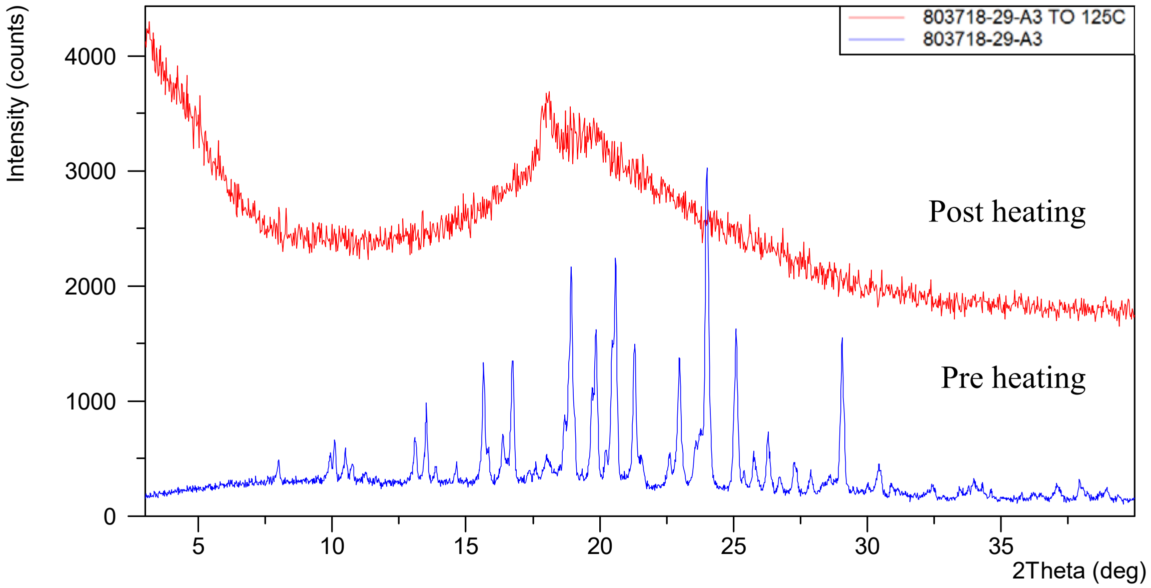

Figure 5-22 XRPD overlay of Type Q (803718-29-A3) pre and post heating

5.4.6 Type S

Type S sample (805711-12-A12) was obtained via slow evaporation in solvent mixture of 1,4-dioxane and MTBE at RT. Its XRPD pattern is shown in Figure 5-23. TGA and DSC data are displayed in Figure 5-24. A weight loss of 15.2% up to 190 ºC in TGA and DSC result showed an endotherm at 107.7 ºC (onset temperature), associated with desolvation.

As analyzed by ^{1}H NMR result shown in Figure 5-25, no MTBE was detected while a molar ratio of 1,4-dioxane to API was determined to be 0.81 (10.2%), approximately consistent with the second-step TGA weight loss. After being heated to 125 ºC, Type S converted into amorphous, as shown in Figure 5-22. From the above data, Type S is speculated to be a 1,4-dioxane solvate.

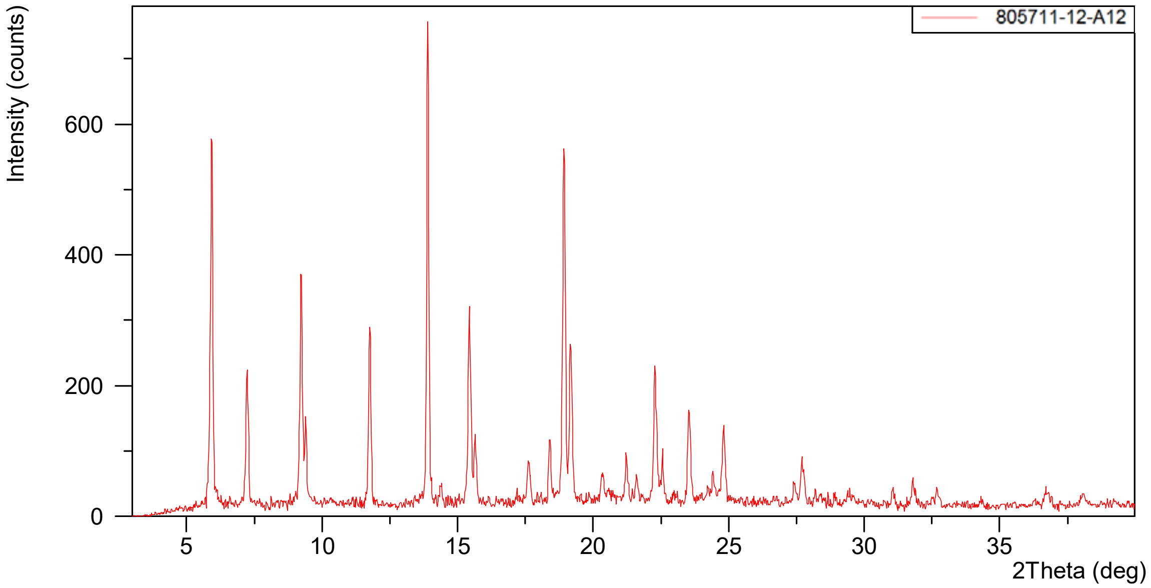

Figure 5-23 XRPD pattern of Type S (805711-12-A12)

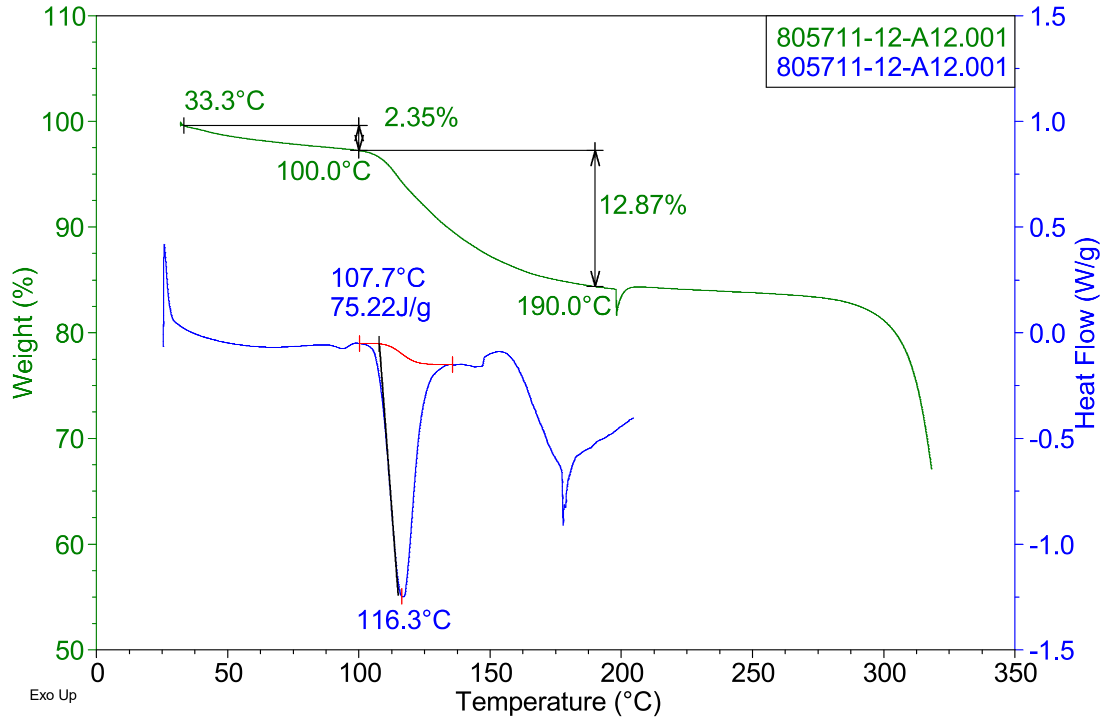

Figure 5-24 TGA/DSC curves of Type S (805711-12-A12)

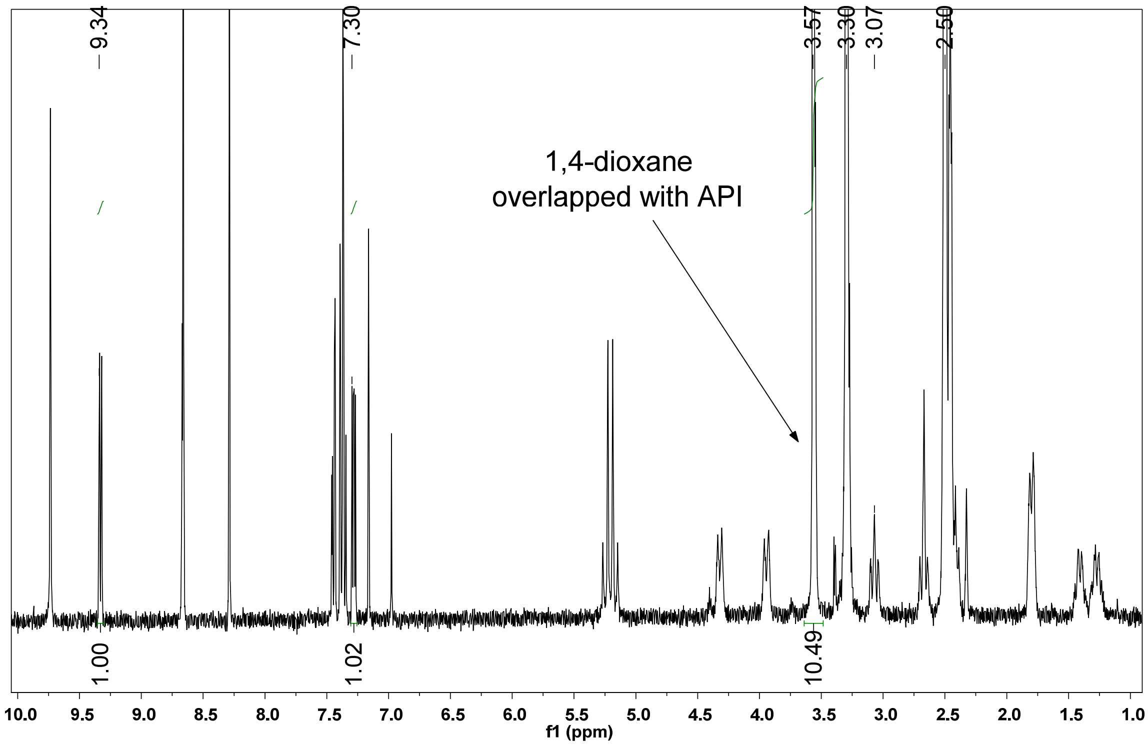

Figure 5-25 ^{1}H NMR spectrum of Type Q (803718-29-A3)

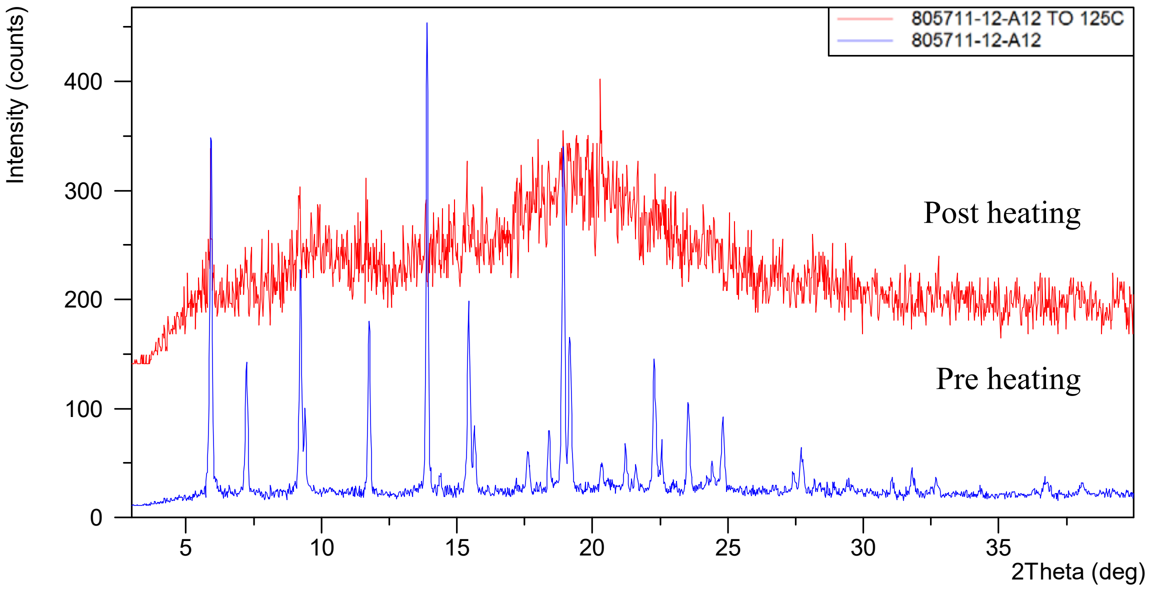

Figure 5-26 XRPD overlay of Type S (805711-12-A12) pre and post heating

5.5 Characterization of Transient and Unidentified Forms

5.5.1 Type D

Type D can only be obtained via slurry of Type A in MeOH at RT. As shown in Figure 5-27, Type D

(805711-22-A1) converted into Type G after air drying overnight, indicating Type D is a transient form at ambient conditions.

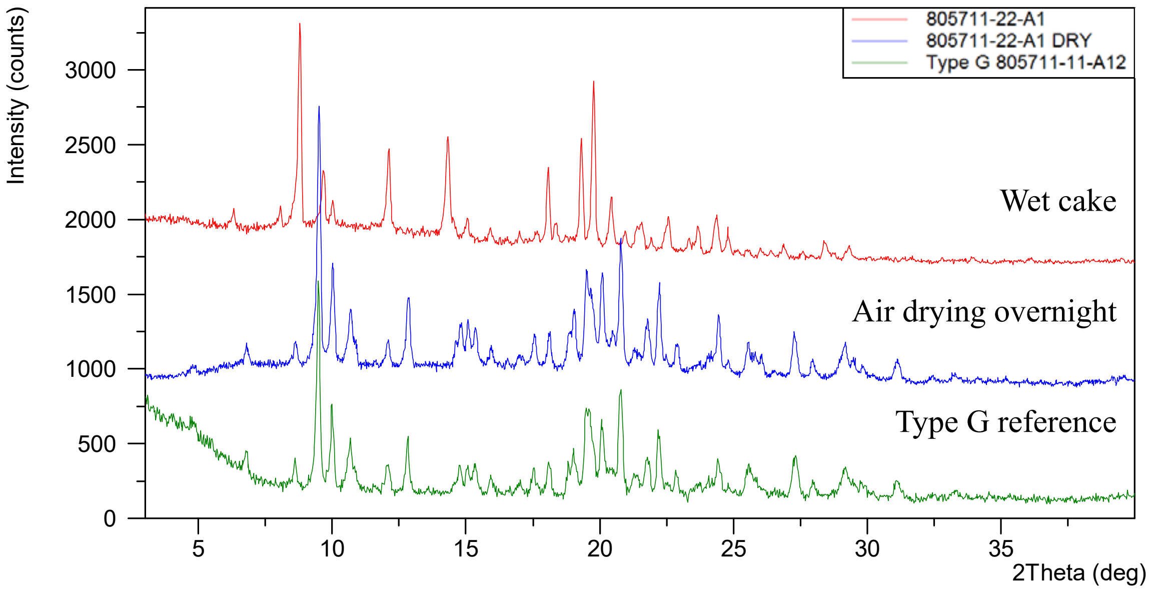

Figure 5-27 XRPD overlay of Type D (805711-22-A1) pre and post air drying

5.5.2 Type G0

Type G0 is the dehydrated form of Type G, and can only be generated *in situ* under protection of nitrogen. XRPD of Type G0 (805711-23-B) is shown in Figure 5-28. As no form change was observed after Type G was heated to 100 ºC and re-exposed to air (Figure 2-25), Type G0 is speculated to be a transient form at ambient conditions.

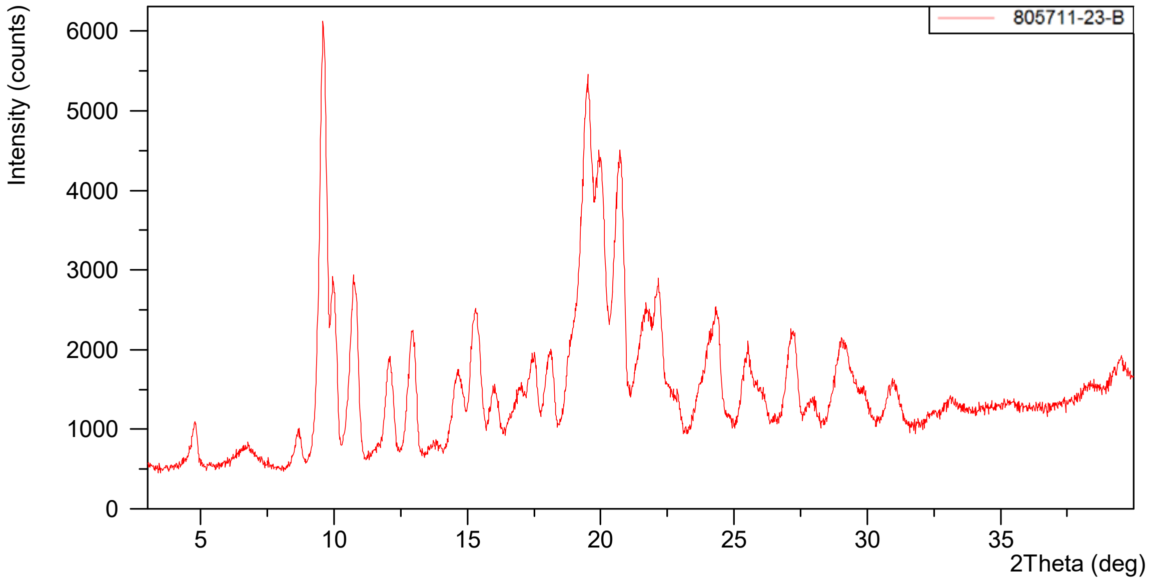

Figure 5-28 XRPD pattern of Type G0 (805711-23-B)

5.5.3 Type F

Type F can be prepared from different solvent systems. Type F sample (803718-12-A3) was obtained via slurry of Type M (803718-09-B) in ACN at RT. As shown in Figure 5-29, Type F converted into Type K after air drying for 4 days, indicating Type F is possibly a transient form at ambient conditions.

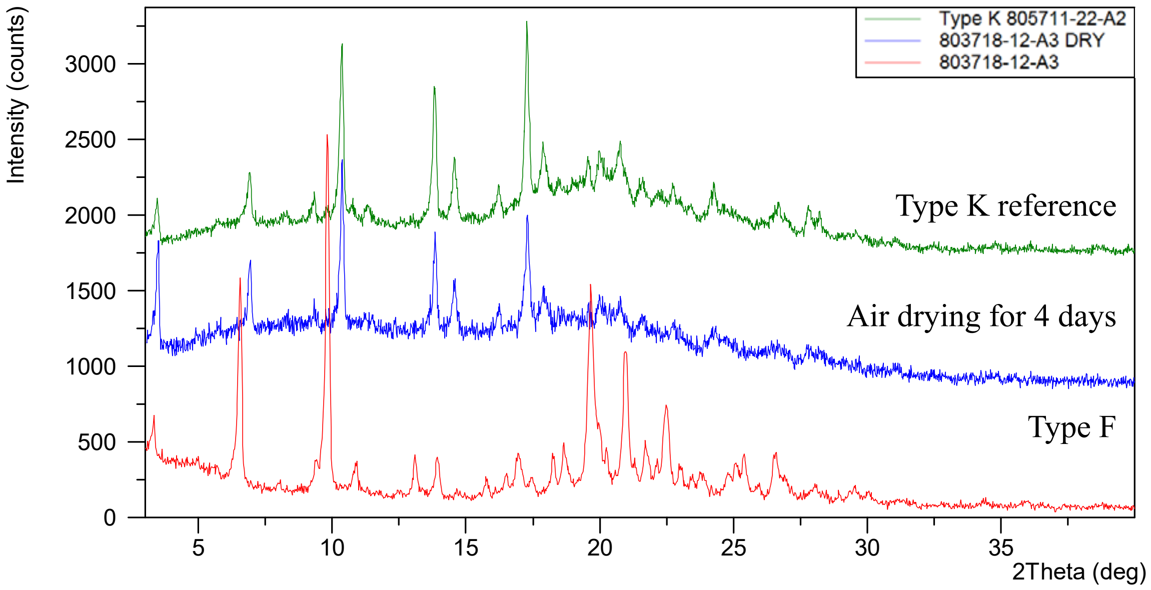

Figure 5-29 XRPD overlay of Type F (803718-12-A3) pre and post air drying

5.5.4 Type O

Type O sample (805711-41-B) was obtained via slurry of Type R (803718-32-B) in EtOH at RT. As shown in Figure 5-29, Type O converted into a mixture of Type O and P after air drying for 3 hrs, indicating Type O is a transient form at ambient conditions.

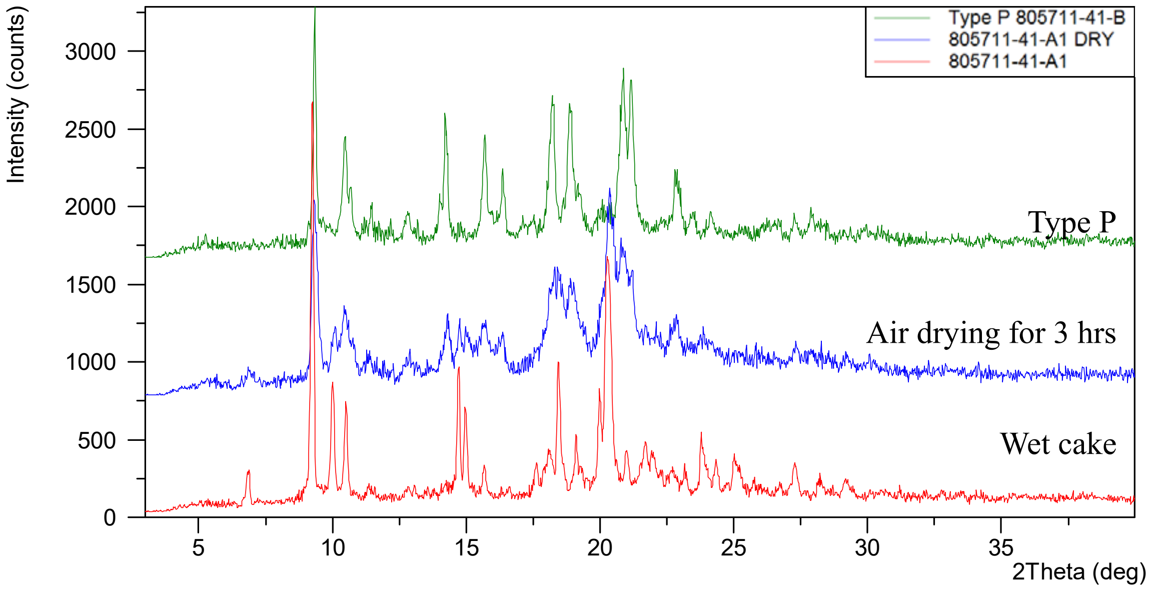

Figure 5-30 XRPD overlay of Type O (803718-41-A1) pre and post air drying

5.5.5 Type T

Type T sample (805711-46-A5) was obtained via slow cooling of MeOH/H_2O (v/v, 1:1) solution from 50 to 5 ºC at a rate of 0.1 ºC/min. As shown in Figure 5-31, Type T converted into a mixture of Type T and U after air drying for 5 mins and pure Type U after air drying overnight, indicating Type T is a transient form at ambient conditions.

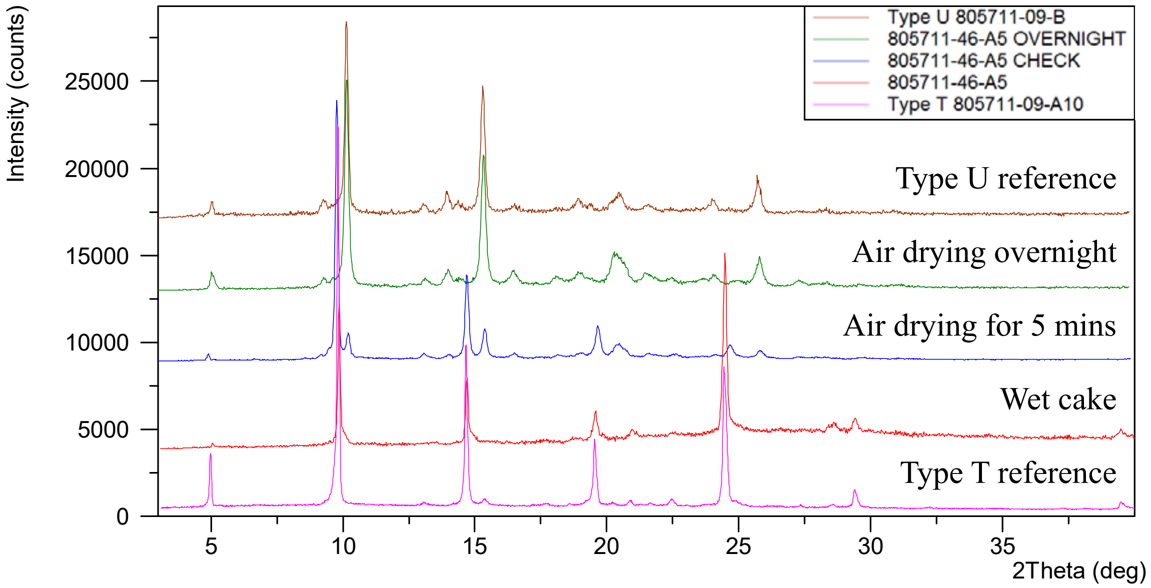

Figure 5-31 XRPD overlay of Type T (805711-46-A5) pre and post air drying

5.5.6 Type V

Type V can be crystallized from THF and 2-MeTHF solution. Type V sample (805711-41-A1) was obtained via interaction of THF solution and MTBE vapor. As shown in Figure 5-32, Type V converted into Type K during storage at ambient conditions. A mixture of Type F and K was obtained instead in the re-preparation trial via interaction of THF solution and MTBE vapor (Figure 5-33). The identity of Type V needs further investigation.

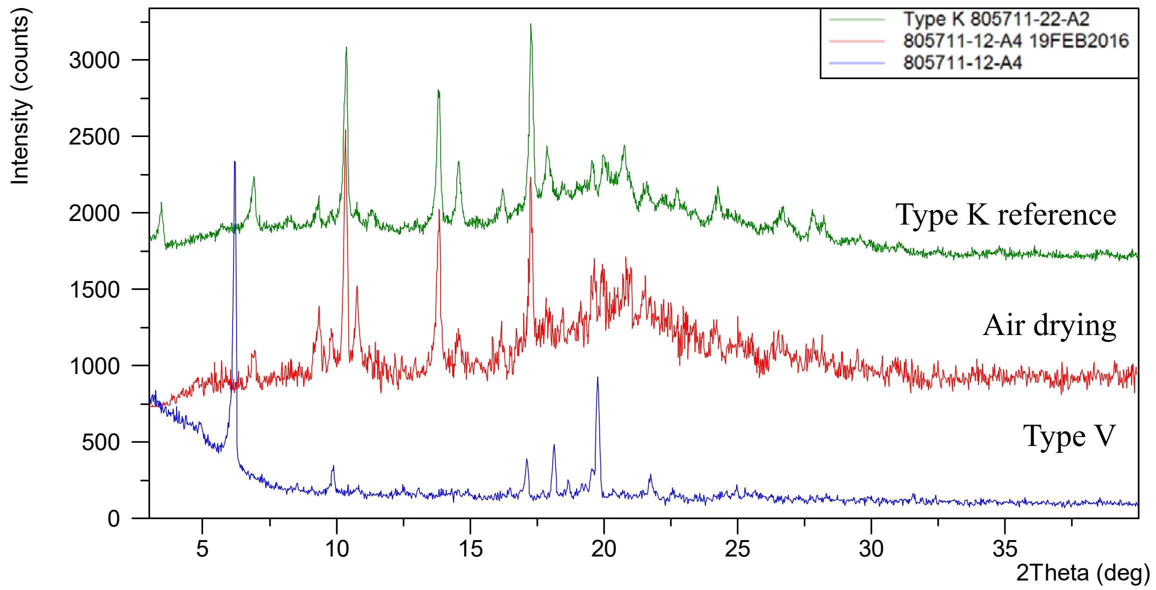

Figure 5-32 XRPD overlay of Type V (805711-12-A4) pre and post air drying

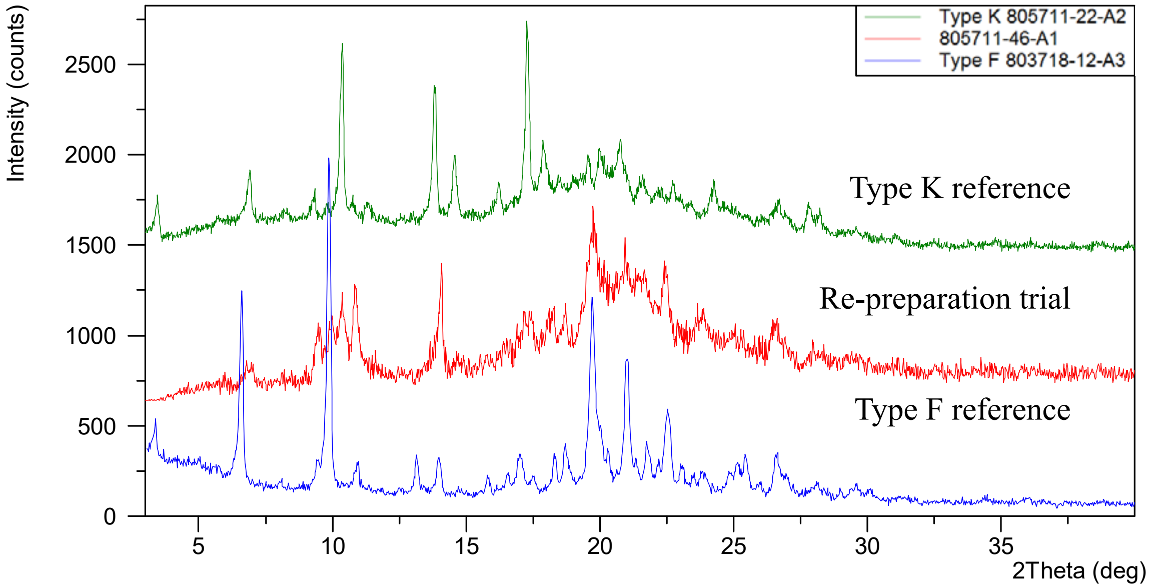

Figure 5-33 XRPD overlay of Type V re-preparation trial (805711-46-A1)

5.5.7 Type W

Type W sample (805711-08-A7) was obtained via slow evaporation of ACN/H_2O (v/v, 1:1) solution at RT. As shown in Figure 5-34, distinctive XRPD pattern was observed during storage of Type W at ambient conditions, assigned as Type X (805711-08-A7). Gel was obtained instead in the re-preparation trial. The identity of Type W needs further investigation.

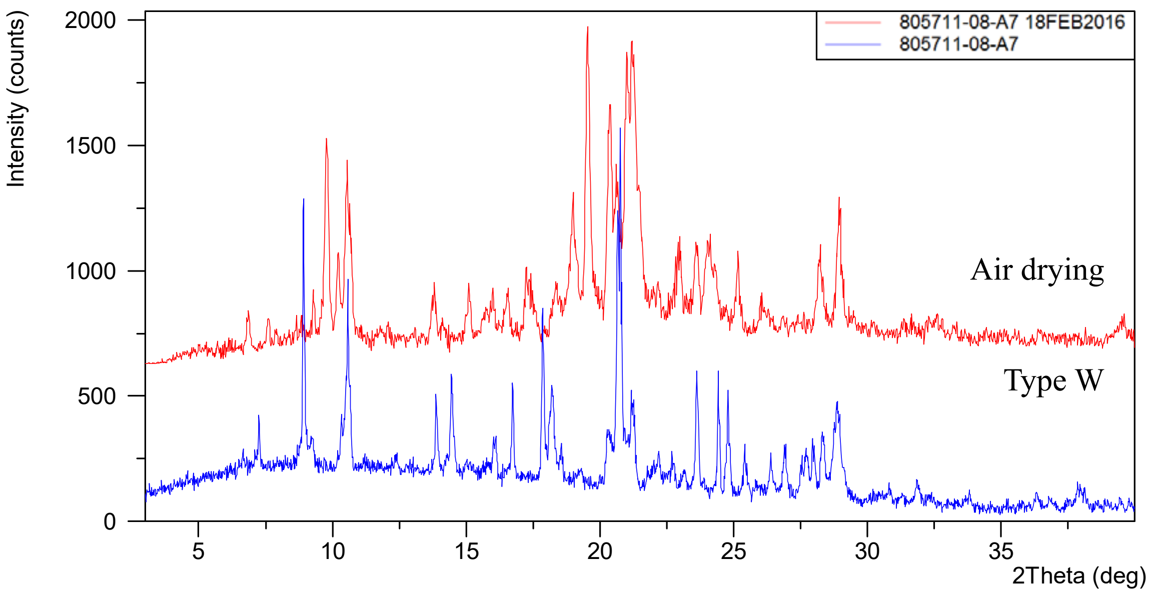

Figure 5-34 XRPD overlay of Type W (805711-08-A7) pre and post air drying

5.6 Polymorph Screening

The solubility of starting material (805711-05-A) was estimated in 18 solvents at RT (25 ± 3 ºC). Approximately 2 mg solids were added into a 3-mL glass vial. Solvents in Table 5-7 were then added step wise (100 µL per step) into the vials until the solids were dissolved or a total volume of 2 mL was reached. Results summarized in Table 5-7 were used to guide the solvent selection in polymorph screening.

Polymorph screening experiments were performed using different solution crystallization or solid transition methods. The methods utilized and crystal forms identified are summarized in Table 5-8.

Table 5-7 Approximate solubility of Type A (805711-05-A) at RT

Solvent	Solubility (mg/mL)	Solvent	Solubility (mg/mL)
MeOH*	S > 44.0	EtOAc	5.3 < S < 7.0
THF	S > 42.0	2-MeTHF	5.3 < S < 7.0
1,4-dioxane	S > 42.0	MIBK	3.8 < S < 4.8
ACN	S > 42.0	EtOH	2.3 < S < 2.5
$CHCl_3$	S > 44.0	toluene	2.2 < S < 2.9
DCM	S > 50.0	IPAc	1.7 < S < 2.0
DMSO	S > 48.0	MTBE	S < 1.1
acetone	20.0 < S < 40.0	n-heptane	S < 1.0

Solvent	Solubility (mg/mL)	Solvent	Solubility (mg/mL)
IPA	6.3 < S < 8.3	H_2O	S < 1.0

*: precipitate out during settling the clear solution

Table 5-8 Summary of polymorph screening experiments

Method	Experiment IDs	No. of Experiments	Isolated Crystal Forms
Solid vapor diffusion	805711-07	12	Type A, B, C, D, K, J, N
Anti-solvent addition	805711-13	13	Type A, C, K, I, X
Slow cooling	805711-09	10	Type A, B, F, K, V, T
Slurry at RT	805711-10	12	Type A, B, C, D, E, F, K
Slurry at 50ºC	805711-11	12	Type A, B, C, K, G, H
Solution vapor diffusion	805711-12	13	Type A, C, K, V, T
Evaporation	805711-08	8	Type A, F, I, W
Total	805711-07~13	80	Type A ~ K, N, T, V, W and X

5.6.1 Solid Vapor Diffusion

Solid vapor diffusion experiments were conducted using 12 different kinds of solvent. Approximately 8 mg of Type A sample (805711-05-A) was weighed into a 3-mL vial, which was placed into a 20-mL vial with 2 mL of relative solvent. The 20-mL vial was sealed with a cap and kept at RT allowing solvent vapor to interact with sample for 7 days. The solids were tested by XRPD and the results summarized in Table 5-9 showed that Type A, B, C, D, K, J and N were observed.

Table 5-9 Summary of solid vapor diffusion experiments

Experiment ID	Solvent	Solid Form	Experiment ID	Solvent	Solid Form
805711-07-A1	H_2O	Type C	805711-07-A7	$CHCl_3$	Clear
805711-07-A2	DCM	Clear	805711-07-A8	acetone	Type K
805711-07-A3	EtOH	Type B	805711-07-A9	EtOAc	Type A
805711-07-A4	MeOH	Type A+D	805711-07-A10	1,4-Dioxane	Type N
805711-07-A5	ACN	Type K	805711-07-A11	IPA	Type A
805711-07-A6	THF	Type K	805711-07-A12	DMSO	Type J

5.6.2 Anti-solvent Addition

A total of 13 anti-solvent addition experiments were carried out. About 10 mg of Type A sample (805711-05-A) was dissolved in 0.2-1.0 mL solvent to obtain a clear solution, and the solution was magnetically stirred, then followed by addition of 0.2 mL anti-solvent per step till precipitate appeared or the total amount of anti-solvent reached 10.0 mL. The precipitate was isolated for XRPD analysis. The clear solutions were transferred to evaporation at RT. Results summarized in Table 5-10 showed that Type A, C, I, K and X were generated.

Table 5-10 Summary of anti-solvent addition experiments

Experiment ID	Solvent	Anti-solvent	Solid Form
805711-13-A1	acetone	MTBE	Type A
805711-13-A2		n-heptane	Type I
805711-13-A3		H_2O	Type C
805711-13-A4	THF	MTBE	Type A
805711-13-A5		n-heptane	Type K
805711-13-A6		H_2O	Type C*
805711-13-A7	ACN	MTBE	Type K*
805711-13-A8		H_2O	Type C
805711-13-A9	DCM	MTBE	Type K
805711-13-A10		n-heptane	Type K
805711-13-A11		toluene	Type X**
805711-13-A12	DMSO	IPAc	Type R**
805711-13-A13		H_2O	Type C*

*: solids were obtained via slurry at 5 ºC.
**: solids were obtained via slow evaporation of clear solutions.

5.6.3 Slow Cooling

Slow cooling experiments were conducted in ten solvent systems. About 10 mg of Type A sample (805711-05-A) was suspended in 1.0 mL of solvent in a 3-mL glass vial at RT. The suspension was then heated to 50 ºC, equilibrated for 2 hrs and filtered to a new vial using a Nylon membrane (pore size of 0.45 μm). Filtrates were slowly cooled down to 5 ºC at a rate of 0.1 ºC/min. The obtained solids were kept isothermal at 5 ºC before isolated for XRPD analysis. Results summarized in Table

5-11 indicated Type A, B, F, K and T were obtained.

Table 5-11 Summary of slow cooling experiments

Experiment ID	Solvent (v/v)	Solid Form
805711-09-A1	IPA	Type F+K
805711-09-A2	EtOAc	Type A
805711-09-A3	2-MeTHF	Type V
805711-09-A4	MIBK	Clear
805711-09-A5	EtOH	Type B
805711-09-A6	toluene	Type A+C
805711-09-A7	IPAc	Clear
805711-09-A8	DCM/n-heptane(1:1)	Type K
805711-09-A9	acetone/MTBE(1:1)	Type K
805711-09-A10	MeOH/H_2O(1:1)	Type T

5.6.4 Slurry at RT

Slurry conversion experiments were conducted at RT in different solvent systems. About 15 mg of Type A sample (805711-05-A) was suspended in 0.5 mL of solvent in a 1.5-mL glass vial. After the suspension was stirred for 6 days at RT, the remaining solids were isolated for XRPD analysis. Results summarized in Table 5-12 indicated that Type A, B, C, D, E, F and K were generated.

Table 5-12 Summary of slurry conversion experiments at RT

Experiment ID	Solvent (v/v)	Solid Form
805711-10-A1	H_2O	Type C
805711-10-A2	MeOH	Type D
805711-10-A3	IPA	Type E
805711-10-A4	EtOAc	Type A
805711-10-A5	2-MeTHF	Type A
805711-10-A6	MIBK/EtOH (1:1)	Type B
805711-10-A7	EtOH	Type B
805711-10-A8	toluene	Type A
805711-10-A9	IPAc	Type A

Experiment ID	Solvent (v/v)	Solid Form
805711-10-A10	acetone/MTBE (1:1)	Type F
805711-10-A11	DCM/n-heptane (1:1)	Type F+K
805711-10-A12	MeOH/H_2O (1:1)	Type C

5.6.5 Slurry at 50 ºC

Slurry conversion experiments were also conducted at 50 ºC in different solvent systems. About 15 mg of Type A sample (805711-05-A) was suspended in 0.5 mL of solvent in a 1.5-mL glass vial. After the suspension was stirred for 6 days at 50 ºC, the remaining solids were isolated for XRPD analysis. Results summarized in Table 5-13 indicated that Type A, B, C, H, K and G were produced.

Table 5-13 Summary of slurry conversion experiments at 50 ºC

Experiment ID	Solvent (v/v)	Solid Form
805711-11-A1	H_2O	Type C
805711-11-A2	MeOH	Clear
805711-11-A3	IPA	Type K
805711-11-A4	EtOAc	Type H
805711-11-A5	2-MeTHF	Type A
805711-11-A6	MIBK/EtOH (1:1)	Clear
805711-11-A7	EtOH	Type B
805711-11-A8	toluene	Type A
805711-11-A9	IPAc	Type A
805711-11-A10	acetone/MTBE (1:1)	Type H
805711-11-A11	DCM/n-heptane (1:1)	Type K
805711-11-A12	MeOH/H_2O (1:1)	Type G

5.6.6 Solution Vapor Diffusion

Thirteen solution vapor diffusion experiments were conducted. Approximately 10 mg of Type A sample (805711-05-A) was dissolved in 1.0 of corresponding solvent to obtain a clear solution in a 3-mL vial. This solution was then placed into a 20-mL vial with 3 mL of relative anti-solvent. The 20-mL vial was sealed with a cap and kept at RT allowing sufficient time for organic vapor to interact with the solution. The precipitates were isolated for XRPD analysis. The results summarized in Table

5-14 showed that Type A, C, K, V and T were observed.

Table 5-14 Summary of solution vapor diffusion experiments

Experiment ID	Solvent	Anti-solvent	Solid Form
805711-12-A1	acetone	MTBE	Type A
805711-12-A2		n-heptane	Type K
805711-12-A3		H_2O	Type C
805711-12-A4	THF	MTBE	Type V
805711-12-A5		n-heptane	Type V
805711-12-A6		H_2O	Clear
805711-12-A7	ACN	MTBE	Type K*
805711-12-A8		toluene	Amorphous
805711-12-A9	DCM	MTBE	Type K
805711-12-A10		n-heptane	Type K
805711-12-A11		IPAc	Type K
805711-12-A12	1,4-dioxane	MTBE	Type T*
805711-12-A13	DMSO	IPAc	Gel*

*: solids were obtained via slow evaporation of clear solutions.

5.6.7 Slow Evaporation

Evaporation experiments were performed under eight conditions. Briefly, about 10 mg of Type A sample (805711-05-A) was dissolved in 1.0 or 2.0 mL of corresponding solvent in a 3-mL glass vial. The visually clear solutions were subjected to evaporation at RT to induce precipitation. The solids were isolated for XRPD analysis, and the results summarized in Table 5-15 indicated that Type A, F, K, I and W was produced.

Table 5-15 Summary of evaporation experiments

Experiment ID	Solvent (v/v)	Solid Form
805711-08-A1	acetone	Type A+I
805711-08-A2	THF	Gel
805711-08-A3	ACN	Type K
805711-08-A4	DCM	Gel
805711-08-A5	2-MeTHF	Type F
805711-08-A6	EtOAc	Type K
805711-08-A7	ACN/H_2O (1:1)	Type W
805711-08-A8	MeOH/DCM (1:1)	Gel

5.7 Notebook Reference

All data from this report can be referred to Notebook 805711, 805612, 803718, 808946 and 808956.

XXXII – Report of ssNMR data confirming the redetermination of ‘Form B’ from CSP.

Antonio DiPasquale, Genentech, Inc., 1 DNA Way, South San Francisco, CA 94080, USA

The experimental and CSP structures of XXXII were both subjected to DFT chemical shielding calculations using CASTEP within the Materials Studio 2022 environment. Resulting shielding values were subsequently compared to the experimental ^{13}C and ^{1}H chemical shifts measured using ^{13}C CPMAS and ^{1}H-^{13}C CP-HETCOR experiments. In both cases, the new CSP structure exhibited slightly better agreement with the experimental solid-state NMR data than the experimental structure solved from PXRD data.

For ^{13}C, the RMSD comparing the experimental shifts and those calculated using the CSP structure was 1.976 ppm, whereas the RMSD using the experimental PXRD structure was 2.105 ppm. For ^{1}H, the RMSD comparing the experimental shifts and those calculated using the CSP structure was 0.649 ppm, and the RMSD using the experimental PXRD structure was 0.751 ppm. In all cases, we believe the RMSD vales are somewhat higher for these structures than is often reported in the literature due to the structural complexity, dynamics present in the real crystal structure at room temperature and above, and limited of ^{1}H resolution particularly in the methylene resonances. Experimental solid-state NMR spectra and associated assignments are shown below.

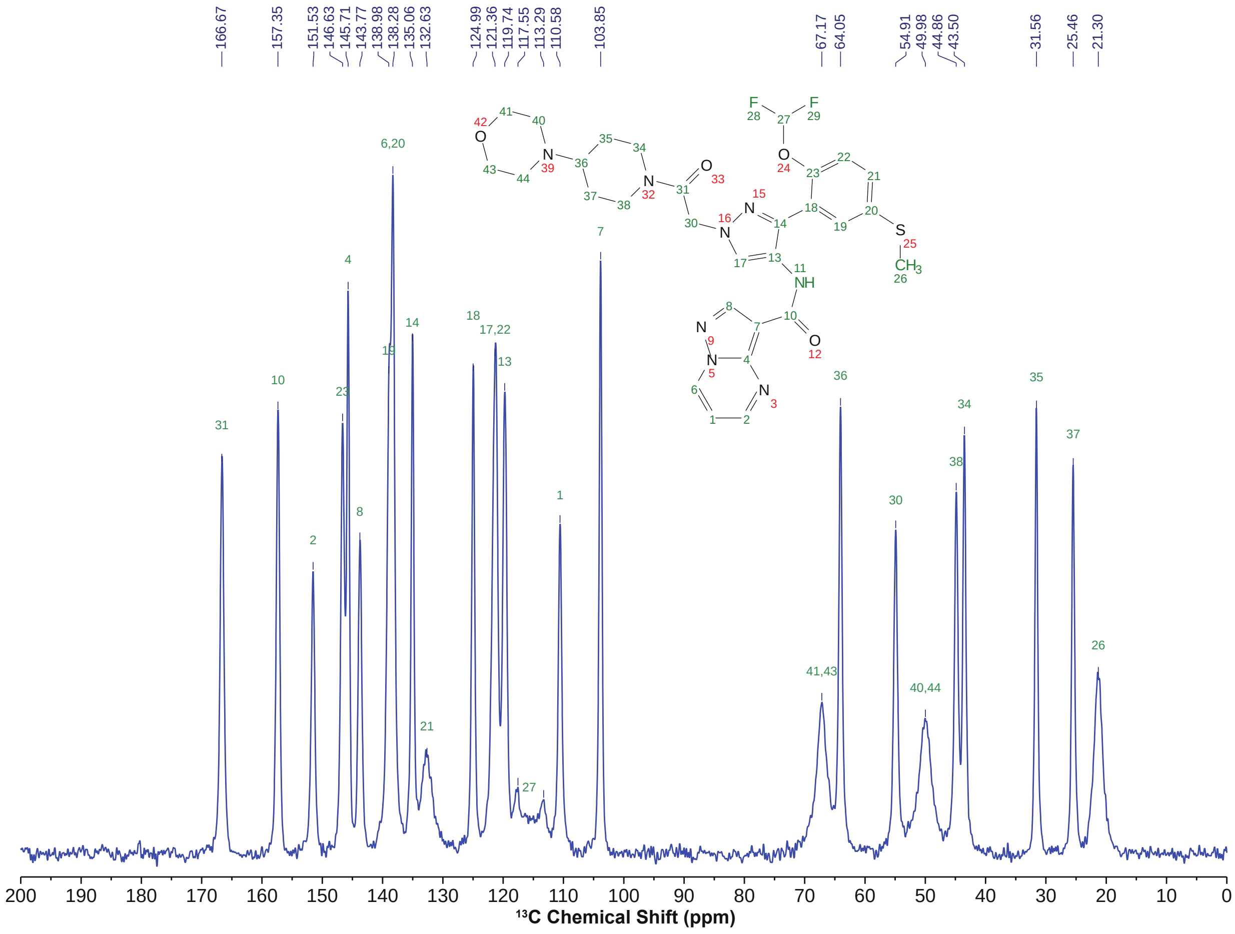

Figure 1. ^{13}C CPMAS spectrum of XXXII acquired at 11.7 T, 303 K, and MAS rate of 8 kHz.

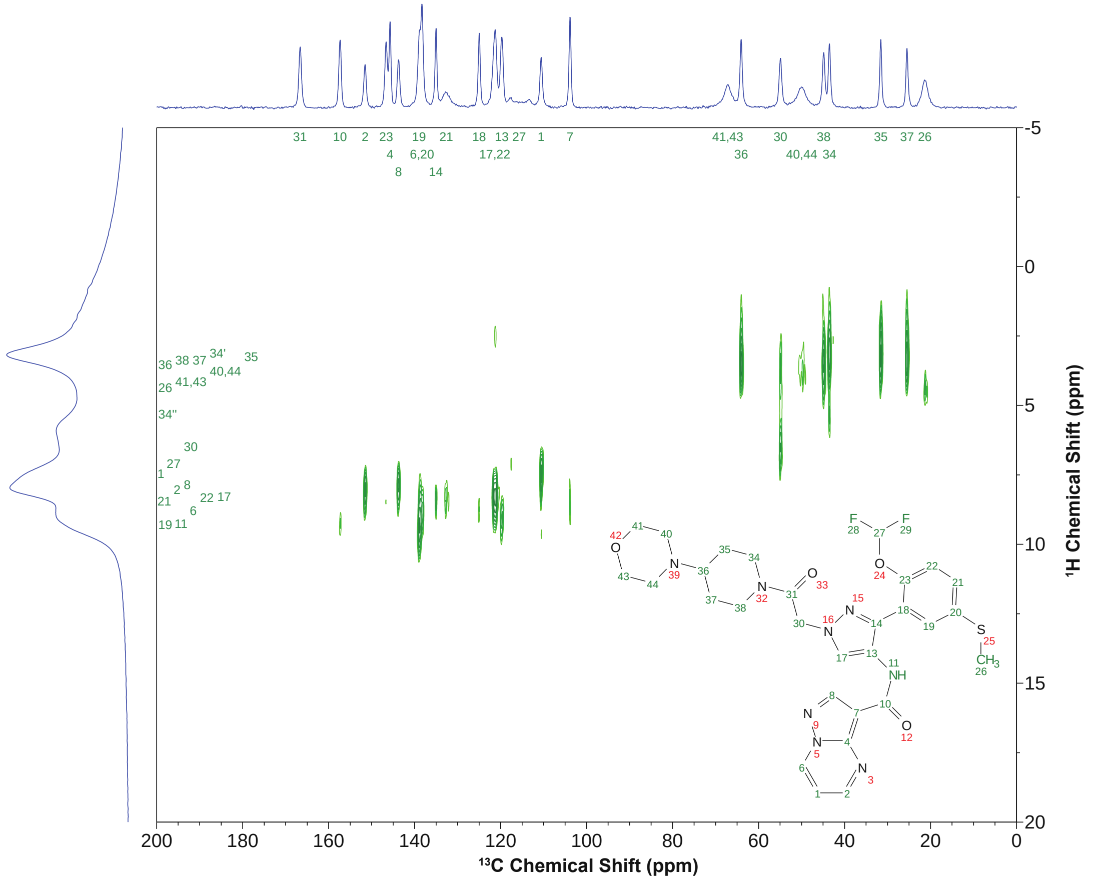

Figure 2. ^1H-^{13}C CP-HETCOR spectrum of XXXII acquired using a contact time of 500 μs at 11.7 T, 303 K, and MAS rate of 14 kHz.

8. Executive Summary - XXXIII

Two polymorphs of a morpholine salt of sulfamethoxazole have been identified. Forms I and II have been renamed Forms A and B, respectively, in the main paper. Form A is a disappearing polymorph, having been originally produced by the method which now gives Form B. A limited screen for polymorphs of the morpholine salt was performed using the solvent drop grinding method. Only Form B (II) was observed. The inability to reproduce Form A (I) precluded a determination of the relative thermodynamic stability of the two salt polymorphs.

Target XXXIII –

Polymorphism of sulfamethoxazole–morpholine salt: A crystallographic analysis

Joseph Cadden[ab], Simon J. Coles[a] and Srinivasulu Aitipamula[b]

[a]Chemistry, University of Southampton, Highfield, Southampton, SO17 1BJ, United Kingdom

[b]Formulation Technologies, Institute of Sustainability for Chemicals, Energy and Environment, 1 Pesek Road, Jurong Island, 627833, Singapore

ABSTRACT: Despite the extensive awareness of both polymorphism and multi-component systems, and their impacts on the physical and chemical properties of active pharmaceutical ingredients, studies on polymorphism in multi-component crystals has only garnered interest in recent years. Herein we report the polymorphic solvate formation of the poorly soluble antibiotic drug sulfamethoxazole with morpholine. The polymorphic nature has been confirmed using powder and single crystal X-ray diffraction. Interestingly, one of the polymorphs was identified as a disappearing polymorph—once the second polymorph was identified, it was never observed again.

1. Introduction

Many molecular crystals are known to exhibit polymorphism; the ability to exist in a number of different crystalline structures.[1] The different polymorphs of these crystals can have a host of effects on its properties, ranging from mere macroscopic properties such as colour and shape to more significant microscopic properties, for example the polymorphs of pharmaceutical compounds can possess considerably different solubilities, thermal stabilities and even bioavailability. Evidently the impacts of polymorphism are diverse, and can be unpredictable, it study and understanding is therefore of paramount importance, no more than in the area of pharmaceuticals—an industry where the properties of materials are the subject of detailed scrutiny

and regulatory control.[2] It is estimated that approximately 70% of all pharmaceutical drugs on the market have been shown to be able to exist in more than one crystalline lattice arrangement, putting polymorphism at the forefront of thoughts for many manufacturers as not just a concern but also a possibility for control and optimisation of physicochemical properties.

Sulfamethoxazole (4-amino-N-(5-methyl-1,2-oxazol-3-yl)benzene-1-sulfonamide, SMX, Figure 1) is a bacteriostatic antibiotic drug shown to be effective in treating a variety of infections, including urinary tract infections, bronchitis and prostatitis.[3] A member of the sulfonamide family, these structural analogs act through the inhibition of folic acid synthesis by competing with its structural analog para-aminobenzoic acid. As a poorly soluble BCS class IV drug, SMX has been the subject of a variety of studies for the improvement of its solubility and dissolution properties, such as micronisation, co-precipitation, and complexation with cyclodextrins.[4] However, its multi-component crystallisation potential has only recently been explored,[5-7] something that CSD analysis has revealed to as being already well studied in various other sulfonamide drugs.[8-13] The chemical structure of SMX contains multiple potential hydrogen-bonding sites, both donating and accepting, thus making multi-component crystallisation of SMX a feasible opportunity.

The objective of this manuscript is to report two polymorphs of SMX-morpholine salt. Both the polymorphs were obtained as part of our efforts to find novel solid forms of SMX for improving its physicochemical properties. Both the polymorphs were successfully characterized by single crystal X-ray diffraction and differences in their crystal structures were analysed in detail.

Materials and Methods

Materials: Sulfamethoxazole was purchased from Biotain Pharma Co., Ltd., China, while all solvents were purchased from Sigma-Aldrich, Singapore, and used as received without any further purification. Analytical grade solvents were used for the crystallization experiments.

Solvate Screening: Crystals suitable for single crystal X-ray diffraction were prepared by solvent evaporative methods at ambient condition.

SMX-Morpholine Form I: 253.3 mg (0.1 mmol) of SMX was dissolved in 5 mL of acetonitrile-morpholine (1:1) 50 °C. The resultant solution was then left at ambient conditions for solvent evaporation to occur. Large block-shaped crystals were obtained in 3 days.

SMX-Morpholine Form II: Form II crystals were found from a repeat experiment conducted to reproduce the crystals of Form I. Form II crystals were repeatedly obtained under identical conditions and adopt a distinct large prismatic morphology.

Single Crystal X-ray Diffraction: Diffraction data for the polymorphs were collected on an Agilent Technologies Dual Source Supernova, four-circle diffractometer fitted with CCD detector, with graphite monochromated Mo-Kα radiation (λ = 0.71073 Å). CrysAlisPro software[14] was used for data collection, reduction, and absorption correction using face indexing and Gaussian corrections. Structure solution and refinement were carried out using Intrinsic Phasing in SHELXT-2015[15] and refined by full-matrix least squares on F^2 using SHELXL-2015,[16] both implemented in the Olex2 software.[17] Non-hydrogen atoms in all structures were refined with anisotropic displacement parameters. Hydrogen atoms for heteroatom (N and O) were located from the difference Fourier map and refined freely, maintaining isotropic displacement parameters (U_{iso}) whilst the remaining hydrogen atoms were fixed in idealized positions with their displacement parameters riding on the values of their parent atoms. Hydrogen bond lengths were neutron normalised (for O−H, N−H and C−H at 0.983 Å, 1.009 Å and 1.008 Å respectively) using PLATON for the calculation of bond lengths and bond angles. Crystallographic data, crystal structure information, and hydrogen bond parameters for all structures presented can be found in the Supporting Information.

Results and discussion

Characterization of all possible polymorphs, identification of the stable polymorph, and design of reliable process for consistent production of the polymorphs are required for development of an active pharmaceutical ingredients.[18] In the case of SMX-morpholine salt, all our experimental attempts to reproduce Form I have failed, as both repetition of the initial experiment and alternate methods have yielded Form II only (Figure 2). Solid-state grinding was chosen as a method of evaluating the polymorph diversity in the salt. Solvent-drop grinding was performed in the presence of morpholine and trace amounts of organic solvents of different polarity. As shown in Figure 3, Form II is the only product from all the screening experiments which was evident from

the perfect match of PXRD pattern of the resulting powders with the simulated pattern of Form II, except that powders obtained from grinding with n-hexane and 1,4-dioxane shows traces of Form I. The fact that Form II is the most abundantly observed polymorph, we presume that Form I could represent a case of a disappearing polymorph.[19, 20]

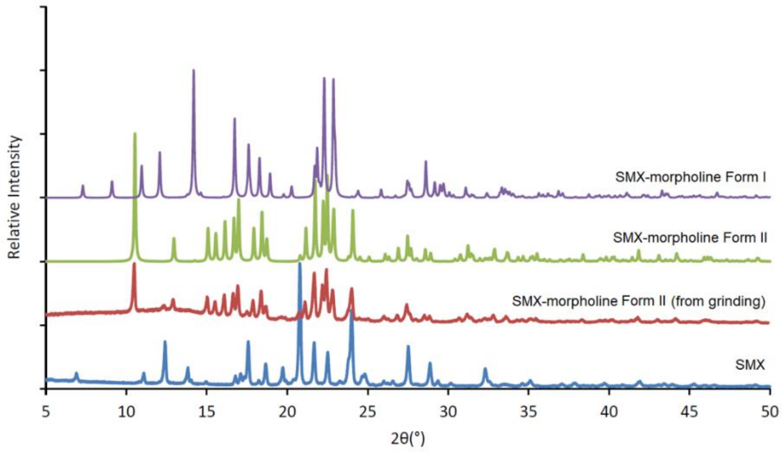

Figure 1. PXRD pattern for the solvent-drop grinding of SMX with morpholine, with reference to SMX and the simulated patterns from the crystal structures of SMX- morpholine Forms I and II.

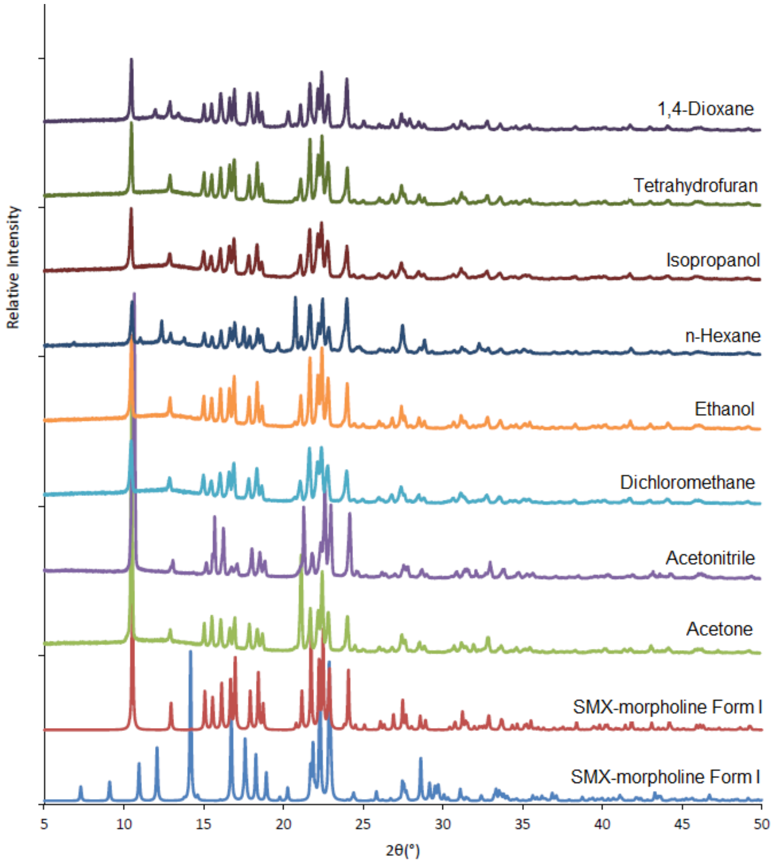

Figure 2. PXRD patterns for the solution crystallization experiments of SMX with morpholine in various solvents, with reference to the simulated patterns from the crystal structures of SMX-MOR Forms I and II, in an attempt to obtain SMX- morpholine Form I.

Crystal Structure analysis

Form I: Form I crystallizes in the monoclinic *C*2/*c* space group with each one molecule of SMX and morpholine in the crystallographic asymmetric unit (Table 1). A proton transfer occurring from the SMX sulfonamide-N2 to morpholine-N4 affords the salt form. Crystal structure is composed of dimeric units of SMX molecules of connected via N–H···N hydrogen bond (Figure 3). These dimers are further connected via two molecules of inversion-related morpholine molecules through N–H···O hydrogen bonds. This results in a chains of hydrogen bonded molecules along the crystallographic [110] direction. These hydrogen bonded chains are connected to the other similar hydrogen bonded chains via several C–H···O and C–H···N interactions (Table 2).

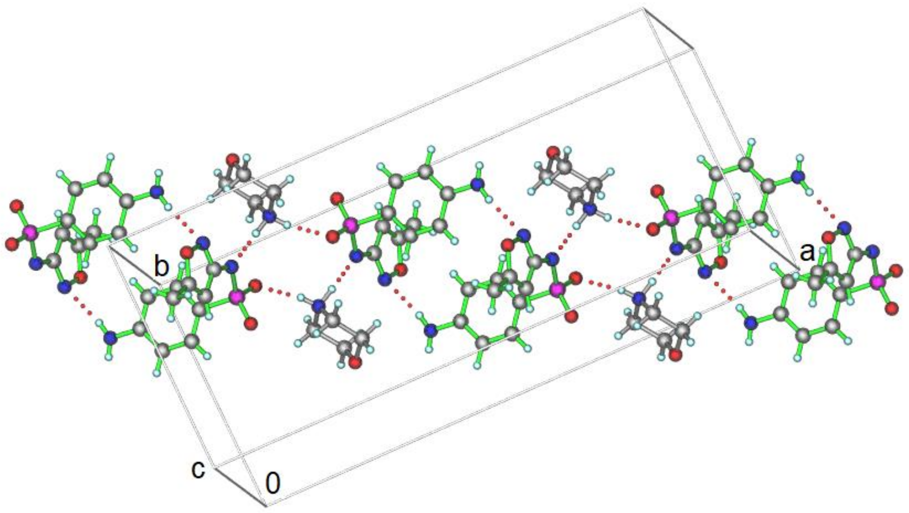

Figure 3. Hydrogen bonded chains in the crystal structure of Form I showing inversion-related molecules of SMX and morpholine forming strong hydrogen bonds. SMX and morpholine molecules were shown in green and gray colors.

Form II: The prismatic crystals of Form II belongs to orthorhombic, $Pna2_1$ space group with each one molecule of SMX and morpholine in the crystallographic asymmetric unit. An analysis of the crystal structure revealed that the hydrogen bonding in Form II is significantly different from Form I. The crystal structure of Form II contains zig-zag chains of SMX connected via morpholine molecules through MOR-$N4^+$–H···N1 SMX and MOR $N4^+$–H···N2 SMX hydrogen bonds through both the SMX isoxazole-N and SMX sulfonamide-N atoms, respectively. The ability of the protonated morpholine-N4 to donate to two separate hydrogen bonds is integral to maintaining the chains, which are arranged in a head-to-tail arrangement of neighboring SMX molecules along the crystallographic *a*-axis. Adjacent chains are linked via SMX-N3–H···O2=S-SMX hydrogen bonds and equivalent molecules of neighboring chains related by a two-fold screw axis along the crystallographic *c*-direction (Figure 4).

Understandably, differences in the interactions between components in the two polymorphs of SMX-morpholine are partially responsible for their structural changes. These interactions are partially dictated by the conformation of SMX molecules. Comparison of conformations shows how the hydrogen-bonding of Form I and Form II can be affected, with rotation about N2–C4 bond allowing for Form II-O2 to participate in hydrogen-bonding and link chains—possibly due to less steric hindrance (Figure 5).

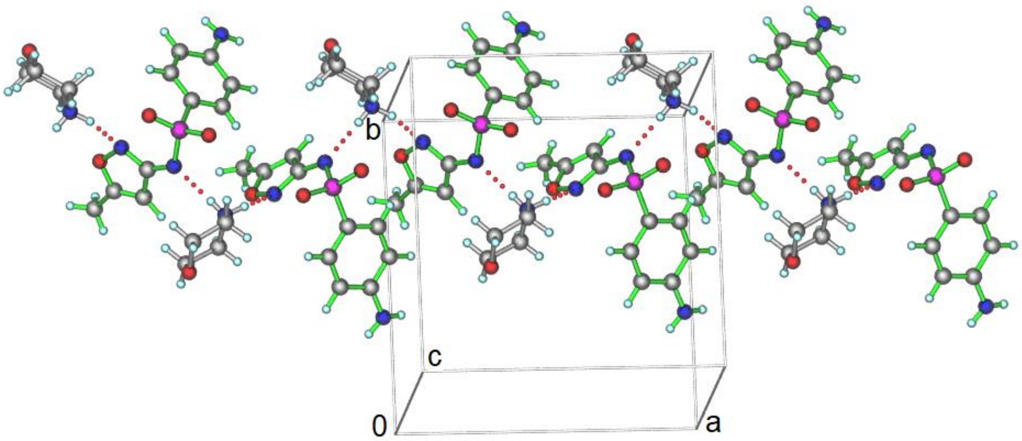

Figure 4. Hydrogen-bonding in SMX-morpholine Form II maintaining the "head-to-tail" arrangement of zig-zag chains of SMX molecules. SMX and morpholine molecules were shown in green and gray colors.

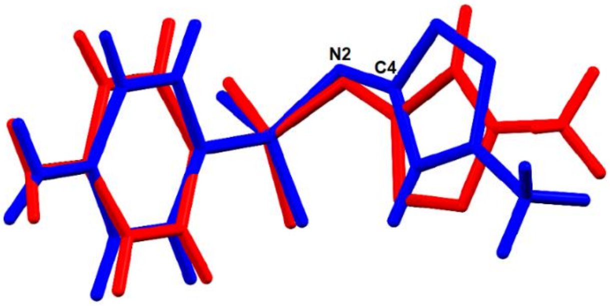

Figure 5. Overlay of SMX molecules in SMX-morpholine Form I (blue) and Form II (red) showing differing conformations of SMX, particularly the rotation about the N2–C4 bond.

Conclusion

A new pharmaceutically-acceptable salt, SMX-morpholine, was obtained as a result of initial exploratory solvate screening and fully characterized with its polymorphic nature confirmed by X-ray diffraction techniques. Proton transfer from SMX to morpholine is essential in maintaining the structure of both polymorphs of the salt, as in both cases the morpholinium ion N-atom acts as a hydrogen-bond donor to two acceptors on separate SMX molecules. Majority of our experimental attempts failed to produce Form I after Form II was observed. It is well-know that identification of reliable methods for polymorph synthesis and a thorough characterization of all the polymorphs are vital for any polymorph system. In this regard, SMX-morpholine salt is a challenging system as one of polymorph is difficult to reproduce.

Table 1. Crystal data and structure refinement for Forms I and II

Polymorph	Form I	Form II
Empirical formula	$C_{14}H_{20}N_4O_4S$	$C_{14}H_{20}N_4O_4S$
Formula weight	340.40	340.40
Temperature/K	296.47(10)	297.1(5)
Crystal system	Monoclinic	Orthorhombic
Space group	*C*2/*c*	$Pna2_1$
a/Å	24.9972(5)	11.7407(2)
b/Å	10.5875(3)	12.4207(2)
c/Å	12.9323(4)	11.3775(2)
α/°	90	90
β/°	104.502(3)	90
γ/°	90	90
Volume/$Å^3$	3313.59(15)	1659.15(5)
Z	8	4
$\rho_{calc}g/cm^3$	1.365	1.363
μ/mm^{-1}	0.221	0.220
F(000)	1440.0	720.0
Crystal size/mm^3	0.887 × 0.635 × 0.265	0.811 × 0.358 × 0.183
Radiation	Mo-Kα (λ = 0.71073)	Mo-Kα (λ = 0.71073)
2Θ range for data collection/°	6.35 to 54.956	6.56 to 54.962
Index ranges	-32 ≤ h ≤ 32, -13 ≤ k ≤ 13, -16 ≤ l ≤ 16	-15 ≤ h ≤ 15, -16 ≤ k ≤ 16, -14 ≤ l ≤ 14
Reflections collected	28650	29393
Independent reflections	3802 [R_{int} = 0.0269, R_{sigma} = 0.0146]	3797 [R_{int} = 0.0179, R_{sigma} = 0.0084]
Data/restraints/parameters	3802/0/289	3797/1/289
Goodness-of-fit on F^2	1.043	1.058
Final R indexes [I>=2σ (I)]	R_1 = 0.0358, wR_2 = 0.0941	R_1 = 0.0232, wR_2 = 0.0661
Final R indexes [all data]	R_1 = 0.0422, wR_2 = 0.0991	R_1 = 0.0235, wR_2 = 0.0664
Largest diff. peak/hole / e $Å^{-3}$	0.23/-0.27	0.15/-0.17

Table 2. Neutron normalized intermolecular interactions in the crystal structure of Forms I and II of SMX-Morpholine salt.

Crystal form	D–H···A*	H···A/Å	D···A/Å	D–H···A/°	Symmetry code
Form II	N3−H3A···N1	2.11	3.104(2)	169	1-x,1-y,1-z
	N3−H3B···O3	2.54	3.241(2)	126	1-x,y,1/2-z
	N4−H4A···N2	1.77	2.772(2)	174	x,y,z
	N4−H4B···O3	1.84	2.827(2)	165	3/2-x,1/2-y,1-z
	C13−H13A···N1	2.55	3.568(2)	157	x,y,z
	C12−H12B···O4	2.69	3.476(2)	129	3/2-x,3/2-y,1-z
	C3−H3···O2	2.58	3.112(2)	110	Intramolecular
	C6−H6···O2	2.47	2.905(2)	102	Intramolecular
Form II	N3−H3A···O2	1.98	2.977(3)	169	2-x,1-y,-1/2+z
	N3−H3B···O3	2.13	2.999(3)	143	3/2-x,-1/2+y,-1/2+z
	N4−H4A···N2	1.83	2.835(2)	179	-1/2+x,3/2-y,z
	N4−H4B···N1	1.92	2.791(2)	143	x,y,z
	C1−H1B···O4	2.60	3.507(4)	141	1/2-x,1/2+y,-1/2+z
	C10−H10···O3	2.50	2.922(3)	102	Intramolecular
	C11−H11B···O1	2.65	3.454(3)	131	1-x,1-y,1/2+z
	C14−H14B···O1	2.52	3.280(3)	126	x,y,z

*D – donor, A - acceptor

References

1. Bernstein, J., *Polymorphism In Molecular Crystals*. Clarendon, Oxford: 2002.
2. Morissette, S. L.; Soukasene, S.; Levinson, D.; Cima, M. J.; Almarsson, O., Elucidation of crystal form diversity of the HIV protease inhibitor ritonavir by high-throughput crystallization. *Proc. Natl. Acad. Sci. U. S. A.* **2003,** *100*, 2180-2184.
3. Kesimli, B.; Topacli, A.; Topacli, C., An interaction of caffeine and sulfamethoxazole: studied by IR spectroscopy and PM3 method. *J. Mol. Struct.* **2003,** *645*, 199-204.
4. Chang, Y.-P.; Tang, M.; Chen, Y.-P., Micronization of sulfamethoxazole using the supercritical anti-solvent process. *Journal of Materials Science* **2008,** *43*, 2328-2335.
5. Kumar, V.; Thaimattam, R.; Dutta, S.; Munshi, P.; Ramanan, A., Structural landscape of multicomponent solids based on sulfa drugs. *CrystEngComm* **2017,** *19*, 2914-2924.
6. Patyk-Kaźmierczak, E.; Kaźmierczak, M., Hydrate vs Anhydrate under a Pressure-(De)stabilizing Effect of the Presence of Water in Solid Forms of Sulfamethoxazole. *Crystal Growth & Design* **2021,** *21*, 6879-6888.
7. Liu, G.; Li, J.; Deng, S., Applications of Supercritical Anti-Solvent Process in Preparation of Solid Multicomponent Systems. *Pharmaceutics* **2021,** *13*.
8. Aitipamula, S.; Chow, P. S.; Tan, R. B. H., The solvates of sulfamerazine: structural, thermochemical, and desolvation studies. *CrystEngComm* **2012,** *14*, 691-699.
9. Bingham, A. L.; Hughes, D. S.; Hursthouse, M. B.; Lancaster, R. W.; Tavener, S.; Threlfall, T. L., Over one hundred solvates of sulfathiazole. *Chemical Communications (Cambridge, United Kingdom)* **2001**, 603-604.
10. Chen, H. B.; Guo, Y. W.; Wang, C. G.; Dun, J. N.; Sun, C. C., Spherical Cocrystallization-An Enabling Technology for the Development of High Dose Direct Compression Tablets of Poorly Soluble Drugs. *Crystal Growth & Design* **2019,** *19*, 2503-2510.
11. Alsubaie, M.; Aljohani, M.; Erxleben, A.; McArdle, P., Co-crystal forms of the BCS class IV drug sulfamethoxazole. *Acta Crystallographica a-Foundation and Advances* **2018,** *74*, E192-E192.
12. Huang, Y. H.; Kuminek, G.; Roy, L.; Cavanagh, K. L.; Yin, Q. X.; Rodriguez-Hornedo, N., Cocrystal Solubility Advantage Diagrams as a Means to Control Dissolution, Supersaturation, and Precipitation. *Mol. Pharm.* **2019,** *16*, 3887-3895.
13. Fu, X.; Li, J. H.; Wang, L. Y.; Wu, B.; Xu, X.; Deng, Z. W.; Zhang, H. L., Pharmaceutical crystalline complexes of sulfamethazine with saccharin: same interaction site but different ionization states. *RSC Advances* **2016,** *6*, 26474-26478.
14. Rigaku Oxford Diffraction, Rigaku Corporation, Oxford, UK, 2018. CrysAlisPro Software system, version 1.171.39.46; 2018. In.
15. Sheldrick, G., A short history of SHELX. *Acta Crystallographica Section A* **2008,** *64*, 112-122.
16. Sheldrick, G. M., Crystal structure refinement with SHELXL. *Acta Crystallogr C Struct Chem* **2015,** *71*, 3-8.
17. Dolomanov, O. V.; Bourhis, L. J.; Gildea, R. J.; Howard, J. A. K.; Puschmann, H., OLEX2: a complete structure solution, refinement and analysis program. *J. Appl. Crystallogr.* **2009,** *42*, 339-341.
18. Bauer, J.; Spanton, S.; Henry, R.; Quick, J.; Dziki, W.; Porter, W.; Morris, J., Ritonavir: An extraordinary example of conformational polymorphism. *Pharm. Res.* **2001,** *18*, 859-866.
19. Dunitz, J. D.; Bernstein, J., Disappearing Polymorphs. *Acc. Chem. Res.* **1995,** *28*, 193-200.

20. Bucar, D.-K.; Lancaster, R. W.; Bernstein, J., Disappearing Polymorphs Revisited. *Angew. Chem. Int. Ed.* **2015,** *54*, 6972-6993.
